# The Prevalence and Predictors of Experiences of Beauty in 22 Countries: An International Assessment of Aesthetic Appreciation in the Global Flourishing Study

**DOI:** 10.1007/s11482-025-10532-z

**Published:** 2026-02-02

**Authors:** Tim Lomas, R. Noah Padgett, James O. Pawelski, Christos A. Makridis, Pedro Antonio de la Rosa Fernández Pacheco, Young-Il Kim, Thomas Breedlove, Richard G. Cowden, Victor Counted, Byron R. Johnson, Tyler J. VanderWeele

**Affiliations:** 1 Harvard University, Cambridge, United States; 2 University of Pennsylvania, Philadelphia, United States; 3 Arizona State University, Tempe, United States; 4 University of Nicosia, Nicosia, Cyprus; 5 University of Navarra, Pamplona, Spain; 6 George Fox University, Newberg, United States; 7 Baylor University, Waco, United States; 8 Regents University, Augusta, United States

**Keywords:** Beauty, Aesthetics, Art, Flourishing, Global

## Abstract

Experiences of beauty (EoB) are an important aspect of wellbeing for many people. However, there is relatively little understanding of factors associated with EoB, especially cross-nationally. We thus present the most comprehensive international study to date on this topic, assessing an item in the Global Flourishing Study (GFS): “Do you regularly experience things that you consider beautiful? This may include physical beauty or abstract beauty like that found in music, art, or nature.” The GFS is a five-year (minimum) panel study investigating the predictors of flourishing across 22 countries, with this item featuring in the Wave 1 “mid-year” survey (*N* = 131,487). Besides measuring flourishing-related outcomes, the GFS assesses 15 flourishing-related factors: four demographic, eight childhood, and three that pertain to both. Overall, the strongest demographic factor (indicated by the variation among the categories in their association with EoB) was education (with EoB higher among those with more education), and the strongest childhood factor was religious service attendance (especially weekly). Even more strikingly, some childhood factors associated with EoB were those that are negative, including even abuse; while that unexpected finding does not mean appreciating such adversity as having an “upside,” it points to EoB having complex roots, including forms of suffering that potentially make people more open and sensitive to the world. The cross-cultural variation was however substantial, with most trends not universal but subject to socio-cultural dynamics. More research is needed to provide deeper understanding of this important aspect of flourishing, for which this paper is a generative foundation and impetus.

**Supplementary Information:**

The online version contains supplementary material available at 10.1007/s11482-025-10532-z.

## Introduction

Appreciation of beauty has been integral to human culture since time immemorial, with virtually all societies incorporating aesthetic considerations into important aspects of life, from religious iconography to building design (Carroll, 2004). What *constitutes* beauty is a complex question that has occupied thinkers for centuries in the philosophical field of aesthetics (Sheppard, 1987), and more recently also in areas like evolutionary psychology (Rhodes, 2006). A question that has received far less attention though is how *prevalent* experiences of beauty (EoB) are, and relatedly what factors are associated with this outcome. We explore these queries by analysing an item on EoB in the Global Flourishing Study (GFS): “Do you regularly experience things that you consider beautiful? This may include physical beauty or abstract beauty like that found in music, art, or nature.” Before turning to the study, this Introduction briefly considers, (a) the importance of beauty (hence why people should care about our findings), (b) what beauty *is*, and (c) the little relevant research that already exists on the prevalence of EoB.

### The Importance of Beauty

The importance of beauty has been celebrated by many academic fields, including most recently a new “positive humanities” paradigm (Pawelski, 2022; Tay & Pawelski, 2022). This brings together a wealth of scholarship, stretching back years, showing the value of beauty—and artistic engagement more broadly—to wellbeing across diverse settings and populations, from ethnic minority children (Khudu-Petersen, 2012) and at-risk youth (Tyson, 2002) to prisoners (Tett et al., 2012) and homeless people (Schnee, 1996). Tay, Pawelski, and colleagues have also created a conceptual model linking art engagement to myriad flourishing outcomes through the RAISE mechanisms (Reflection, Acquisition, Immersion, Socialization, Expression) (Shim et al., 2019; Tay et al., 2018; Thapa et al., 2024). Treading similar territory, advocating for a field of “positive art,” Lomas (2016) identified five classes of positive outcomes relating to artistic expression or appreciation: (1) aesthetic appreciation, with perceiving beauty being psychologically rewarding (Chatterjee, 2014); (2) facilitating elevated experiential states—such as awe, which Keltner and Haidt (2003) describe as a rarefied “spiritual emotion”—enriching people’s lives; (3) sense-making, helping people comprehend existence and find meaning in it, as exemplified by the role of religious iconography in articulating religious teachings (Jensen, 2013); (4) entertainment, whereby many people simply enjoy art and aesthetics, even when these do not necessarily serve any higher or deeper purpose (Funch, 1997); and (5) opportunities for bonding, from intimate relationships forged through shared experiences of art (Loke & Khut, 2014), to broader forms of art-related interconnectedness (Jamal, 2023).

Central to these categories is beauty, which has arguably been—at least before recent centuries—a necessary condition for characterizing something as “art” (Lomas, 2025a). The temporal caveat there is significant, since in the modern era artistic currents have emerged that refuse to engage with beauty or are even *anti*-beauty. Dadaism, for instance, originating in Geneva in 1916, described itself as “anti-art,” challenging conventions such as art having to be aesthetically pleasing and/or meaningful (Padularosa, 2019). Understood as a reaction to World War I, Dadaism rejected conventions of so-called civilised society, as crystallized in its aesthetic preferences and traditions, which the artists involved saw as implicated in the unfolding horrors of the war, and in the decades since, postmodern art forms have continued to decouple beauty from art. But for most of human history, beauty was integral to art, and moreover still is today for the many people—arguably the majority worldwide—who are still drawn to non-postmodern forms of art. Moreover, the relevance and importance of beauty extends beyond art and applies to all stimuli that people appreciate, such as natural phenomena, as our next section briefly considers.

### The Meaning of Beauty

Questions around the nature of beauty have been explored for centuries by the philosophical field of aesthetics (Sheppard, 1987) and more recently by areas like evolutionary psychology (Rhodes, 2006). It is beyond our scope to provide a comprehensive account of the relevant scholarship, so we shall just articulate a perspective on aesthetics we find helpful—while recognizing that it is not the only way of approaching the topic—namely that of philosopher Ken Wilber (1996). While myriad theories on aesthetics have been developed over the years, rather than judging these as “right” or “wrong,” Wilber suggests all have some merit, but only partially, and when considered together provide a comprehensive understanding of beauty. Wilber’s framework itself draws on semiotician C. S. Peirce (1955), whose influential theory of meaning features three inter-related parts that together constitute a “sign-system”: a sign (also called a “representamen”); an object; and an interpretant. The sign is a signifier: “something which stands to somebody for something in some respect or capacity” (p.99)—anything from a facial expression (e.g., a smile) to a weather event (e.g., a lightning bolt). The object is the phenomenon signified by the sign: a smile may signify happiness, and lightening an immanent storm. Finally, there is neither a fixed nor one-to-one correspondence between objects and signs—a given sign can potentially indicate or represent many objects, with patterns of signification changing fluidly across persons, contexts, and times—so the interpretant is someone who decodes/interprets the meaning of the sign.

Wilber draws on Peirce’s schema to understand the contribution of different theories of aesthetics, categorizing them according to where they “situate” the beauty of a phenomenon, locating this principally either in the sign, and/or object, and/or interpretant. For example, one could regard a painting as a sign, the painter’s state of mind as the object, and the viewer as the interpretant. Formalist theories locate beauty in the sign itself, whereby an artwork could be deemed inherently and objectively beautiful (Hanslick, 1986). The notion of “intrinsic” beauty can be contentious, especially given considerable cross-cultural and historical variation in aesthetic tastes (Frith et al., 2005). Many Western artistic trends, especially pre-20th Century, are characterised by qualities such as realism, vivid colour use, and harmonic proportion. Zen art by contrast, for example, is guided by other qualities which reflect the philosophy of Zen (Suzuki, 1959), including *kanso* (elegant simplicity), *fukinsei* (asymmetry, irregularity), *koko* (austere sublimity), *shizen* (naturalness), *datsuzoku* (freedom from convention), *seijaku* (stillness, tranquillity), and *yūgen* (profound depth) (Hisamatsu, 1971). But even if some ideals are culturally contingent, that does not mean all are, with attempts to find more “objective” aesthetic qualities that may hold constant across contexts. A well-known example is the “golden ratio”—even if there are popular misconceptions about how widespread its occurrence is (Markowsky, 1992)—which is found whenever the irrational number Φ (roughly 1.618) describes *both* the ratio between two quantities *and* the ratio between the larger quantity and the sum of the two. It is widely regarded as highly aesthetically pleasing, for reasons that are much debated, including that it reflects spiral-like patterns that recur in nature, from seashells to galaxies (Green, 1995). Whatever the reasons, it has been widely harnessed, in arenas from architecture to painting—with Botticelli’s “Birth of Venus” often cited as an example (Cellucci, 2015)—with the ratio purportedly helping account for the aesthetic qualities of such work.

Intentional theories by contrast, influenced by Romanticism, locate beauty more in the “object,” in the original intent, feeling, or vision of the creator (Croce, 1995). Such theories are less invested in artworks per se, placing greater interest in the artist, with art taking on meaning mainly as a manifestation of the artist’s particular genius and perspective. An interesting instance might be the following haiku by Bashō (1644–1694): “An old pond; A frog jumps in; The sound of water.” The poem itself has been celebrated as stripping language to its essence, capturing the immediate “suchness” of reality; as Barthes (1982) puts it, haiku aim for the “end of language,” enabling direct “apprehension of the thing” in itself, “awakening to the fact” of reality as it is (p.78). Yet what Zen scholars prize above all about this particular haiku is what is *represents*, namely the occasion of Bashō’s own enlightenment. Finally, reception and response theories place the emphasis on the interpretant, with beauty conferred by the viewer; Passmore (1991) even suggests “the interpreter, not the artist, creates the work” (p.34). While most people would surely not deny the role of the artist, the viewer is of course critical in “extracting” meaning and other qualities from art (and any aesthetic phenomena). One might imagine, for instance, Bashō’s haiku would be valued more highly, and seen with greater depth, by people who understand the context regarding his enlightenment and appreciate Zen and Japanese culture more broadly.

Finally, although Wilber’s framework applies principally to art, it can be extended to all phenomena, as elucidated by Lomas (2019) in proposing a paradigm of “positive semiotics,” which seeks to understand the ways phenomena could be appraised as “positive” (including as beautiful). Aligned with formalist theories of art are perspectives that emphasize qualities of beauty themselves, including in relation to natural phenomena, as noted above vis-à-vis the golden ratio (Green, 1995). Then more along the lines of intentional theories are perspectives that focus on what phenomena *represent*, and even what they might say about whatever process or entity was involved in their creation. In an exploration of different forms of “love of creation” across religious and philosophical traditions, for example, Lomas et al. (2024) suggested—drawing on Watts (1957)—that across cultures have been three main ontological perspectives regarding the creation of the cosmos: constructed artefact (fashioned by a creator God); organic process (a natural emergent unfolding); and divine play (a cosmic drama). Whatever one’s view, it is possible to understand a given instance of beauty, from a flower to a sunset, as a reflection—in addition to whatever aesthetic qualities it may possess in itself—of this underlying spiritually-inflected creative process. And aligned with reception and response theories are perspectives that focus on the role of the interpretant in determining what constitutes beauty, such as scholarship which emphasizes the role of cultural traditions and values in that regard. One example might be that although most cultures create and enjoy gardens, there is considerable variety in aesthetic norms and practices, with van den Berg and van Winsum-Westra (2010) identifying three main styles—manicured, romantic, and wild—each with many permutations.

Much more could be said regarding perceptions and standards of beauty, but this will suffice in at least pointing to the depth and complexity of such scholarship. For our purposes here though, we are not so much focused on what beauty *is* but how widely it is experienced.

### The Prevalence of Beauty

Despite the wealth of academic attention paid to beauty, there has been strikingly little empirical social scientific work on people’s *experiences* of it. A Google Scholar search in December 2025 for “experience of beauty” returned only 14,200 results, with just 136 with this phrase in the title (with only 3,130 and 26 respectively for "experiences" in the plural) . Moreover, most are not empirical scientific studies (i.e., with prevalence data), but scholarship that is more historical or philosophical, like Carruthers’ (2013) book “The experience of beauty in the Middle Ages.” Then, of the subset that *are* empirical, most are not designed to assess the *prevalence* of EoB, though they do provide insight into its dynamics: Brielmann et al. (2021) for instance studied 851 people in the US, UK, and India, and found that beauty experiences were characterized by six dimensions: intense pleasure; an impression of universality; the wish to continue the experience; exceeding expectation; perceived harmony in variety; and meaningfulness. Of particular interest here though is the *prevalence* of EoB, which has received relatively little attention. The main reason perhaps is that despite its importance, beauty tends not to be prioritized in the type of study that would allow such assessment—e.g., international surveys like the Gallup World Poll—where the focus when it comes to wellbeing is more on metrics around physical and mental health. There are *some* studies, but these are limited in their coverage and scope: Diessner et al.'s (2008) Engagement with Beauty Scale for example has 328 citations as of December 2025, but these tend to be studies with sample sizes in the hundreds (e.g., Barrows et al., 2022). So too with “appreciation of beauty and excellence”—a character strength in the Values in Action taxonomy (Peterson & Seligman, 2004)—which has been assessed psychometrically in studies with samples such as 461 (Büssing et al., 2014), 355 (Martínez-Martí et al., 2016), and 1,721 (Ruch and Gander (2022).

Moreover, most studies do not assess such experiences internationally, mostly focusing on societies labelled by Henrich et al. (2010) as “WEIRD” (Western, Educated, Industrialised, Rich, and Democratic). While one cannot simplistically classify places in a binary way as WEIRD versus non-WEIRD—since each element of the acronym is a spectrum upon which countries may be situated (Ghai, 2021)—most of the world is not *as* WEIRD as the US or Western Europe, from where a majority of research in top journals originates. This bias has various implications, as a wealth of research shows people have meaningful differences across different aspects of life related to their cultural location, as demonstrated recently by the GFS, which assesses the predictors of human flourishing across over 200,000 participants (in its first wave) from 22 diverse countries. It involves an expansive questionnaire covering myriad dimensions of flourishing (Lomas, Bradshaw et al., 2025)—based on a framework developed by VanderWeele (2017), co-PI of the GFS—together with myriad childhood and socio-demographic factors. Findings from the GFS are now being published across an extensive series of co-ordinated studies, with most focusing on a single outcome—from psychological constructs such as inner peace (Lomas, Padgett, et al., 2025a, 2025b), to social variables like social trust (Kim et al., 2025a, 2025b)—as well as reviews of multiple outcomes (VanderWeele et al., 2025). These all reveal meaningful cultural variation, both in the outcomes themselves, and the factors associated with such outcomes, with all the studies following a common analytic strategy focusing on the same 15 childhood (Padgett, Bradshaw et al., 2025a) and socio-demographic (Padgett, Bradshaw et al., 2025b) factors.

Based on such studies, one would thus expect cross-cultural variation to also apply to EoB. One might similarly expect this based on the literature, such as the differences noted above between aesthetic sensibilities across cultures. The analysis is guided by three research questions: (1) What are the distributions and descriptive statistics of key childhood and socio-demographic factors in our international sample; (2) how do mean levels of EoB order across countries; and (3) how do levels of EoB vary across childhood and socio-demographic categories? In relation to these questions, we have three hypotheses: (1) the distributions and descriptive statistics of key childhood and socio-demographic features will reveal diverse patterns across our sample; (2) mean levels of EoB will vary meaningfully across different countries; and (3) EoB will exhibit variations across different childhood and socio-demographic categories, and these differences across categories will themselves vary by country. The study design was pre-registered with the Open Science Framework on April 9th, 2025 (https://osf.io/p3t6w).

## Methods

The study design, sampling, and survey development for the GFS are described elsewhere, including overall summaries of the GFS (VanderWeele et al., 2025) and the methodology (Johnson et al., 2024; Ritter et al., 2024, 2025), the GFS questionnaire design (Cowden et al., 2024; Crabtree et al., 2021; Lomas, Bradshaw et al., 2025 ), the translation process (Case et al., 2025), the survey sampling design (Padgett, Cowden, et al., 2025) , the analytic methodology (Padgett, Bradshaw et al., 2025a, 2025b), the codebook (Markham et al., 2024), and the statistical analysis code (Padgett et al., 2024).

### Study Sample

Wave 1 of the GFS included nationally representative samples from 22 countries: Argentina, Australia, Brazil, China (the mainland, and also separately Hong Kong, a Special Administrative Region of China), Egypt, Germany, India, Indonesia, Israel, Japan, Kenya, Mexico, Nigeria, the Philippines, Poland, South Africa, Spain, Sweden, Tanzania, Turkey, the UK and US (*N* = 207,919). The countries were selected to (1) maximize coverage of the world’s population, (2) ensure geographic, cultural, and religious diversity, and (3) prioritize feasibility in Gallup’s existing data collection infrastructure. Data for Wave 1, involving an expansive 109-item questionnaire, were collected from March 2022 to January 2024, except in mainland China (March/April of 2024). Data for Wave 2, involving mostly the same items as Wave 1 (excluding the childhood and many demographic items) were collected from January 2024 to December 2024, with mainland China collected at least six months after Wave 1. Additionally, between Waves 1 and 2 was a brief “mid-year” survey involving just 14 questions, including the item on beauty we focus on here (*N* = 131,487).

### Sampling Design

The precise sampling design varied by country to ensure samples were approximately nationally representative. In most countries, local field partners implemented a probability-based face-to-face or telephone methodology to recruit panel members. Recruitment involved an intake survey gathering basic sociodemographic information and details for recontacting participants. Following recruitment, participants received invitations to participate in the annual survey via phone or online. Follow-up for Wave 2 data collection relied on the respondent-provided contact information. A minimum of three contact attempts were made on different days of the week and times of day to maximize the possibility of retention. Post-stratification and nonresponse adjustments to the Wave 1 sampling weights were performed separately within each country, using either census data or a reliable secondary source.

### Measures

The outcomes analyzed were assessed using the following measures. For additional details on the assessments see the COS GFS codebook (Markham et al., 2024) or other sources (Crabtree et al., 2021).

#### Outcome variable

EoB: “Do you regularly experience things that you consider beautiful? This may include physical beauty or abstract beauty like that found in music, art, or nature.” Response categories: yes; no; don’t know; refused to answer.

#### Childhood Antecedents

- Relationship with mother: “Please think about your relationship with your mother when you were growing up. In general, would you say that relationship was very good, somewhat good, somewhat bad, or very bad?” Responses were dichotomized to very/somewhat good versus very/somewhat bad. “Does not apply” was treated as a dichotomous control variable for respondents who did not have a mother due to death or absence.
- Relationship with father: Analogous variable used.
- Parental marital status: Response categories: married, divorced, never married, and one or both had died.
- Financial status: “Which one of these phrases comes closest to your own feelings about your family's household income when you were growing up, such as when YOU were around 12 years old?” Response categories: comfortably, got by, found it difficult, and found it very difficult.
- Abuse: “Were you ever physically or sexually abused when you were growing up?” Response categories: yes, no.
- Outsider status: “When you were growing up, did you feel like an outsider in your family?” Response categories: yes, no.
- Childhood health: “In general, how was your health when you were growing up? Was it excellent, very good, good, fair, or poor?”
- Religious attendance: “How often did YOU attend religious services or worship at a temple, mosque, shrine, church, or other religious building when YOU were around 12 years old?” Response categories: at least once/week, one-to-three times/month, less than once/month, or never.
- Childhood religious tradition/affiliation was had response categories of Christianity, Islam, Hinduism, Buddhism, Judaism, Sikhism, Baha’i, Jainism, Shinto, Taoism, Confucianism, Primal/Animist/Folk religion, Spiritism, African-Derived, some other religion, or no religion/atheist/agnostic; precise response categories varied by country.

#### Demographic Variables

- Marital status: assessed as single/never married, married, separated, divorced, widowed, and domestic partner.
- Employment: assessed as employed, self-employed, retired, student, homemaker, unemployed and searching, and other.
- Service attendance: assessed as more than once/week, once/week, one-to-three times/month, a few times/year, or never.

- Immigration status was dichotomously assessed with: “Were you born in this country, or not?”
- Education: assessed as up to 8 years, 9–15 years, and 16 + years.

#### Childhood/Demographic Variables

- Gender: assessed as male, female, or other.
- Continuous age (year of birth): classified as 18–24, 25–29, 30–39, 40–49, 50–59, 60–69, 70–79, and 80 or older.
- Immigration status: “Were you born in this country, or not?”
- Religious tradition/affiliation with categories of Christianity, Islam, Hinduism, Buddhism, Judaism, Sikhism, Baha’i, Jainism, Shinto, Taoism, Confucianism, Primal/Animist/Folk religion, Spiritism, African-Derived, some other religion, or no religion/atheist/agnostic; precise response categories varied by country (Johnson et al., 2024).
- Racial/ethnic identity assessed in some, but not all, countries, with response categories varying by country.

### Analysis

Descriptive statistics for the full sample, weighted to be nationally representative within each country, were estimated for each demographic variable. Nationally representative proportions for EoB were estimated separately for each country and ordered from highest to lowest, along with 95% confidence intervals and robust standard errors. Variation in proportions in EoB across demographic categories were estimated, with all analyses initially conducted by country (see online supplement). Primary results consisted of random effects meta-analyses of country-specific proportions of EoB in each specific demographic category (Borenstein et al., 2010; Hunter & Schmidt, 2000), along with 95% confidence intervals, standard errors, lower and upper limits of a 95% prediction interval across countries, heterogeneity (τ), and variation within particular demographic variables across countries (Mathur & VanderWeele, 2020). Forest plots of estimates are available in the online supplement. All meta-analyses were conducted in R (R Core Team, 2024) using the metafor package (Viechtbauer, 2010). Within each country, a global test of variation of outcome across levels of each particular demographic variable was conducted, and a pooled *p*-value (Wilson, 2019) across countries reported concerning evidence for variation within any country. Bonferroni corrected p-value thresholds are provided based on the number of demographic variables (Abdi, 2007; VanderWeele & Mathur, 2019). Religious affiliation/tradition and race/ethnicity were used, when available, as control variables within each country, but were not included in the meta-analyses since the availability of these response categories varied by country. As a supplementary analysis, population weighted meta-analyses were also conducted. All analyses were pre-registered with COS prior to data access (10.17605/OSF.IO/ZT84X); all code to reproduce analyses are openly available in an online repository (Padgett et al., 2024).

### Missing Data

Missing data on all variables were imputed using multivariate imputation by chained equations, and five imputed datasets were used (Sterne et al., 2009; van Buuren, 2023). To account for variation in the assessment of certain variables across countries (e.g., religious affiliation/tradition and race/ethnicity), the imputation process was conducted separately in each country. This within-country imputation approach ensured that the imputation models accurately reflected country-specific contexts and assessment methods. Sampling weights were included in the imputation model to account for specific-variable missingness that may have been related to probability of inclusion in the study.

### Accounting for Complex Sampling Design

The GFS used different sampling schemes across countries based on availability of existing panels and recruitment needs (Ritter et al., 2024). All analyses accounted for the complex survey design components by including weights, primary sampling units, and strata. Additional methodological detail, including accounting for the complex sampling design, is provided elsewhere (Padgett, Cowden et al., 2025).

## Results

Table 1 provides the distribution of descriptive statistics (weighted counts and proportions). The GFS assessed 15 socio-demographic factors: four demographic, eight childhood, and three pertaining to both (age/birth cohort, gender, and immigration status, analysed/presented below both as demographic and childhood factors).

**Table 1:** Summary statistics of mid-year data

Characteristic	*N* = 131,487^1^
Age group*, n (%)*	
1943 or earlier (current age: 80 + years)	2,739 (2.1%)
1943–1953 (current age: 70–79 years)	11,624 (8.8%)
1953–1963 (current age: 60–69 years)	19,288 (15%)
1963–1973 (current age: 50–59 years)	20,893 (16%)
1973–1983 (current age: 40–49 years)	21,886 (17%)
1983–1993 (current age: 30–39 years)	25,217 (19%)
1993–1998 (current age: 25–29 years)	12,372 (9.4%)
1998–2005 (current age: 18–24 years)	17,457 (13%)
(Missing)	11 (< 0.1%)
Gender*, n (%)*	
Male	63,490 (48%)
Female	67,477 (51%)
Other	349 (0.3%)
(Missing)	171 (0.1%)
Marital status*, n (%)*	
Single/Never been married	33,071 (25%)
Married	72,215 (55%)
Separated	2,802 (2.1%)
Divorced	7,860 (6.0%)
Widowed	6,298 (4.8%)
Domestic partner	8,612 (6.5%)
(Missing)	629 (0.5%)
Employment*, n (%)*	
Employed for an employer	51,451 (39%)
Self-employed	23,274 (18%)
Retired	20,456 (16%)
Student	7,053 (5.4%)
Homemaker	13,485 (10%)
Unemployed and looking for a job	10,125 (7.7%)
None of these/Other	5,382 (4.1%)
(Missing)	261 (0.2%)
Religious service attendance*, n (%)*	
More than once a week	16,558 (13%)
Once a week	25,368 (19%)
One to three times a month	11,925 (9.1%)
A few times a year	24,819 (19%)
Never	52,425 (40%)
(Missing)	392 (0.3%)
Education*, n (%)*	
Up to 8 years	28,969 (22%)
9–15 years	72,554 (55%)
16 + years	29,859 (23%)
(Missing)	105 (< 0.1%)
Immigration*, n (%)*	
Born in this country	124,540 (95%)
Born in another country	5,931 (4.5%)
(Missing)	1,016 (0.8%)
Religious affiliation*, n (%)*	
Christianity	63,854 (49%)
Islam	14,293 (11%)
Hinduism	6,850 (5.2%)
Buddhism	5,935 (4.5%)
Judaism	2,690 (2.0%)
Sikhism	125 (< 0.1%)
Baha’i	28 (< 0.1%)
Jainism	24 (< 0.1%)
Shinto	322 (0.2%)
Taoism	195 (0.1%)
Confucianism	43 (< 0.1%)
Primal, Animist, or Folk religion	412 (0.3%)
Spiritism	210 (0.2%)
Umbanda, Candomblé, and other African-derived religions	144 (0.1%)
Chinese folk/traditional religion	154 (0.1%)
Some other religion	1,452 (1.1%)
No religion/Atheist/Agnostic	33,862 (26%)
(Missing)	898 (0.7%)
Parent marital status	
Parents were married	100,930 (77%)
Parents were divorced	11,590 (8.8%)
Parents were never married	8,875 (6.7%)
One or both of them had died	4,980 (3.8%)
Unsure	1,128 (0.9%)
(Missing)	3,984 (3.0%)
Age 12 religious service attendance	
At least once a week	53,384 (41%)
One to three times a month	20,277 (15%)
Less than once a month	22,891 (17%)
Never	33,808 (26%)
(Missing)	1,127 (0.9%)
Relationship with mother	
Very good	81,679 (62%)
Somewhat good	34,949 (27%)
Somewhat bad	7,616 (5.8%)
Very bad	2,870 (2.2%)
(Does not apply)	3,887 (3.0%)
(Missing)	486 (0.4%)
Relationship with father	
Very good	68,326 (52%)
Somewhat good	37,295 (28%)
Somewhat bad	10,786 (8.2%)
Very bad	5,321 (4.0%)
(Does not apply)	9,000 (6.8%)
(Missing)	759 (0.6%)
Outsider growing up	
Yes	18,828 (14%)
No	110,605 (84%)
(Missing)	2,054 (1.6%)
Abuse	
Yes	19,181 (15%)
No	108,634 (83%)
(Missing)	3,672 (2.8%)
Self-rated health growing up	
Excellent	44,705 (34%)
Very good	40,873 (31%)
Good	29,774 (23%)
Fair	12,721 (9.7%)
Poor	3,103 (2.4%)
(Missing)	311 (0.2%)
Subjective financial status of family growing up	
Lived comfortably	45,758 (35%)
Got by	54,148 (41%)
Found it difficult	22,993 (17%)
Found it very difficult	8,272 (6.3%)
(Missing)	316 (0.2%)
Country	
Argentina	2,728 (2.1%)
Australia	2,533 (1.9%)
Brazil	4,044 (3.1%)
China	4,544 (3.5%)
Egypt	3,386 (2.6%)
Germany	4,163 (3.2%)
Hong Kong	706 (0.5%)
India	8,206 (6.2%)
Indonesia	2,675 (2.0%)
Israel	2,476 (1.9%)
Japan	13,886 (11%)
Kenya	9,115 (6.9%)
Mexico	2,126 (1.6%)
Nigeria	4,943 (3.8%)
Philippines	3,448 (2.6%)
Poland	4,316 (3.3%)
South Africa	1,625 (1.2%)
Spain	2,215 (1.7%)
Sweden	11,570 (8.8%)
Tanzania	6,577 (5.0%)
Turkey	659 (0.5%)
United Kingdom	3,354 (2.6%)
United States	32,192 (24%)

Country averages on EoB are reported in Table 2. The mean score is reported with associated 95% confidence intervals, country-level standard deviation, and Gini coefficient of inequality.

**Table 2:** Ordered proportions of beauty

Country	Proportion	95% CI	SE
1. South Africa	0.90	(0.88, 0.92)	0.011
2. Nigeria	0.90	(0.88, 0.91)	0.008
3. Australia	0.88	(0.86, 0.89)	0.009
4. United States	0.86	(0.85, 0.87)	0.003
5. Philippines	0.85	(0.84, 0.86)	0.007
6. Israel	0.85	(0.82, 0.88)	0.015
7. Sweden	0.84	(0.83, 0.85)	0.004
8. Germany	0.84	(0.82, 0.85)	0.007
9. China	0.83	(0.81, 0.84)	0.006
10. Argentina	0.82	(0.80, 0.85)	0.011
11. Mexico	0.82	(0.80, 0.84)	0.011
12. Spain	0.80	(0.78, 0.83)	0.012
13. Indonesia	0.79	(0.76, 0.81)	0.012
14. Kenya	0.75	(0.74, 0.77)	0.009
15. Egypt	0.74	(0.72, 0.76)	0.011
16. Brazil	0.73	(0.71, 0.75)	0.010
17. United Kingdom	0.73	(0.70, 0.75)	0.011
18. Turkey	0.72	(0.68, 0.77)	0.022
19. Poland	0.69	(0.65, 0.72)	0.017
20. India	0.62	(0.60, 0.64)	0.009
21. Tanzania	0.55	(0.53, 0.57)	0.010
22. Hong Kong	0.47	(0.42, 0.52)	0.026
23. Japan	0.44	(0.43, 0.45)	0.005

*N* = 131,487. CI, confidence interval—can be non-symmetric due to high precision and rounding; SE, standard error of proportion; and Proportion = estimated proportion of population within country that has achieved 16 + years of education (tertiary)

**Fig. 1:**
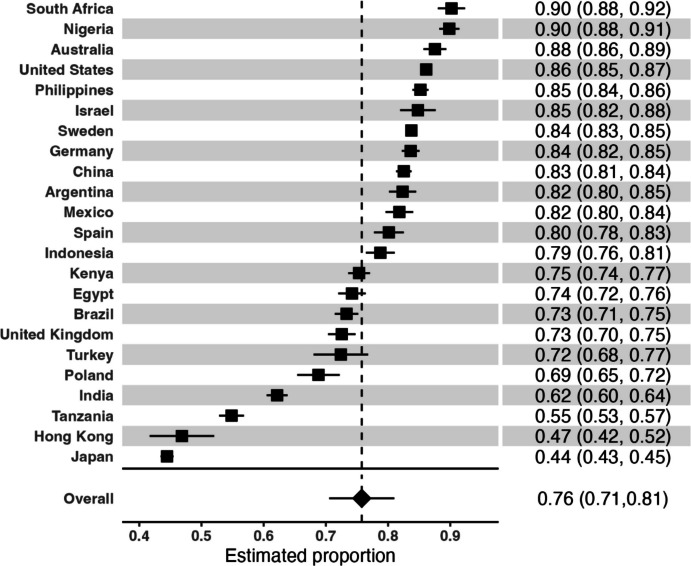
Forest plot for beauty proportions across countries

Meta-analytic estimates based on subgroups of demographic characteristics are presented in Table 3, and a Forest plot for beauty proportions across countries is shown in Figure 1.

**Table 3:** Random effects meta-analysis of beauty proportions by demographic category

					Prediction Interval				
Variable		Est	95% CI	SE^1^	LL	UL	τ	I^2^	Global *p*-value
Age group									3.01e-06***
18–24		0.80	(0.76,0.85)	0.02	0.45	0.93	0.11	96.5	
25–29		0.77	(0.71,0.83)	0.03	0.33	0.92	0.14	97.4	
30–39		0.75	(0.69,0.81)	0.03	0.34	0.91	0.15	98.8	
40–49		0.74	(0.69,0.80)	0.03	0.38	0.90	0.14	98.3	
50–59		0.74	(0.68,0.79)	0.03	0.41	0.90	0.14	98.2	
60–69		0.73	(0.67,0.79)	0.03	0.46	0.93	0.14	98.2	
70–79		0.73	(0.68,0.79)	0.03	0.43	0.92	0.13	96.7	
80 or older^ǂ^		0.75	(0.67,0.82)	0.04	0.47	0.97	0.13	92.1	
Gender									1.64e-06***
Male		0.76	(0.70,0.81)	0.03	0.38	0.90	0.13	99.2	
Female		0.76	(0.71,0.82)	0.03	0.46	0.90	0.13	99.4	
Other^ǂ^		0.77	(0.59,0.95)	0.09	0.45	0.95	0.19	86.0	
Marital status									2.87e-06***
Married		0.75	(0.70,0.81)	0.03	0.44	0.90	0.13	99.3	
Separated		0.77	(0.71,0.82)	0.03	0.50	0.94	0.11	87.1	
Divorced		0.77	(0.69,0.84)	0.04	0.37	0.98	0.17	97.3	
Widowed		0.73	(0.68,0.79)	0.03	0.46	0.88	0.12	91.5	
Domestic partner		0.77	(0.71,0.84)	0.03	0.47	0.93	0.14	97.2	
Single, never married		0.78	(0.72,0.83)	0.03	0.41	0.91	0.13	98.7	
Employment status									4.29e-06***
Employed for an employer		0.77	(0.72,0.82)	0.03	0.39	0.94	0.13	99.1	
Self-employed		0.79	(0.74,0.84)	0.03	0.53	0.91	0.12	97.9	
Retired		0.74	(0.68,0.80)	0.03	0.43	0.90	0.13	98.1	
Student		0.83	(0.79,0.88)	0.02	0.53	0.96	0.09	92.5	
Homemaker		0.74	(0.69,0.80)	0.03	0.45	0.90	0.13	96.5	
Unemployed and looking for a job		0.74	(0.68,0.80)	0.03	0.33	0.96	0.13	96.0	
None of these/other		0.73	(0.67,0.79)	0.03	0.39	0.91	0.13	92.0	
Education									2.24e-06***
Up to 8 years		0.70	(0.64,0.76)	0.03	0.34	0.90	0.13	97.7	
9–15 years		0.77	(0.72,0.82)	0.03	0.42	0.91	0.12	99.4	
16 + years		0.83	(0.79,0.88)	0.02	0.52	0.96	0.11	98.8	
Religious service attendance									3.29e-06***
> 1/week		0.82	(0.78,0.87)	0.02	0.57	0.96	0.10	95.3	
1/week		0.79	(0.75,0.83)	0.02	0.55	0.90	0.10	96.5	
1–3/month		0.78	(0.73,0.84)	0.03	0.47	0.94	0.13	96.8	
A few times a year		0.78	(0.73,0.83)	0.02	0.49	0.89	0.12	98.0	
Never		0.73	(0.67,0.78)	0.03	0.42	0.89	0.14	99.2	
Immigration status									2.23e-05***
Born in this country		0.76	(0.70,0.81)	0.03	0.45	0.90	0.13	99.6	
Born in another country		0.78	(0.72,0.85)	0.03	0.46	0.97	0.14	94.6	

^*^p <.05; **p <.007 (Bonferroni corrected threshold). (Bonferroni corrected threshold); ǂGroup is very small (< 0.1% of the observed sample) within several countries leading to large uncertainty in this estimate—be cautious about interpreting this estimate; ^1^SE Analogue = Standard Error approximated by CI Width/4; CI = confidence interval; LL = lower limits of prediction interval; UL = upper limit of prediction interval; prediction interval is the range of likely values of the estimate for a randomly selected country; τ is the standard deviation of the distribution of proportions (back-transformed from the logit-scale) across countries, which is an indicator of cross-national heterogeneity; I^2^ is an estimate of the variability in means due to heterogeneity across countries vs. sampling variability which is not uncommonly nearly 100% when there i precision in estimated proportion within country; and the global p-value corresponds to a test of the null hypothesis that there are no differences between the groups for that sociodemographic characteristic in all of the 22 countries or territories

Meta-analytic estimates of how childhood experiences predict EoB are shown in Table 4.

**Table 4:** Meta-analyzed effects of childhood predictors on experiences of beauty

Variable	Category	RR	95% CI	RR < 0.90	RR > 1.10	I^2	Global p-value
Relationship with your mother growing up	(Ref: Very bad/somewhat bad)						0.006
	Very good/somewhat good	1.01	(1.00,1.03)	0.00	0.00	9.00	
Relationship with your father growing up	(Ref: Very bad/somewhat bad)						0.380
	Very good/somewhat good	1.00	(0.99,1.01)	0.00	0.00	21.69	
Parents married to each other when you were around 12 years ol	(Ref: Parents married)						5.14e-05
	One or both of them had died	1.02	(0.99,1.04)	0.00	0.04	45.59	
	Parents were divorced	1.01	(0.99,1.03)	0.00	0.00	49.89	
	Parents were never married	1.01	(0.98,1.04)	0.00	0.04	72.28	
	Unsure	1.01	(0.97,1.06)	0.00	0.09	50.49	
Feelings about family’s household income when growing up	(Ref: Got by)						1.00e-07
	Lived comfortably	1.02	(1.02,1.03)	0.00	0.00	39.38	
	Found it difficult	0.98	(0.97,0.99)	0.00	0.00	13.90	
	Found it very difficult	0.97	(0.96,0.99)	0.00	0.00	40.07	
Physically or sexually abused when growing up	(Ref: No)						5.40e-06
		1.03	(1.01,1.04)	0.00	0.00	48.40	
Felt like an outsider in your family when growing up	(Ref: No)						0.345
	Yes	1.00	(0.99,1.01)	0.00	0.00	24.37	
Your health when growing up	(Ref: Good)						0.110
	Excellent	1.02	(1.01,1.03)	0.00	0.00	22.37	
	Very good	1.01	(1.00,1.03)	0.00	0.00	37.97	
	Fair	1.01	(0.99,1.02)	0.00	0.00	17.09	
	Poor	0.99	(0.97,1.02)	0.00	0.00	0.00	
Born in this country	(Ref: Born in this country)						2.63e-05
	Born in another country	1.03	(0.99,1.06)	0.04	0.09	66.72	
Attended religious services or worshiped around 12 years old	(Ref: Never)						7.66e-15
	At least once a week	1.07	(1.04,1.09)	0.00	0.17	75.61	
	One to three times a month	1.05	(1.02,1.08)	0.00	0.17	70.58	
	Less than once a month	1.04	(1.01,1.07)	0.00	0.13	78.27	
Year of birth (age group)	(Ref: 1998–2005; current age: 18–24)						3.79e-10
	1943 or earlier (current age: 80 + years)	0.98	(0.92,1.05)	0.23	0.14	84.41	
	1943–1953 (current age: 70–79 years)	0.94	(0.90,0.98)	0.26	0.04	73.47	
	1953–1963 (current age: 60–69 years)	0.94	(0.91,0.97)	0.30	0.00	79.18	
	1963–1973 (current age: 50–59 years)	0.95	(0.92,0.97)	0.13	0.00	68.56	
	1973–1983 (current age: 40–49 years)	0.95	(0.93,0.97)	0.09	0.00	65.19	
	1983–1993 (current age: 30–39 years)	0.96	(0.94,0.97)	0.00	0.00	58.03	
	1993–1998 (current age: 25–29 years)	0.98	(0.96,0.99)	0.00	0.00	0.00	
Race plurality (prominent race/ethnic group [0] or not [1])	Non-plurality groups	0.98	(0.96,1.00)	0.06	0.00	69.17	
FATHER_NA	Father NA flag	0.98	(0.96,0.99)	0.00	0.00	0.00	
MOTHER_NA	Mother NA flag	0.99	(0.97,1.01)	0.00	0.00	15.12	

^*^p <.05; **p <.004 (Bonferroni corrected threshold); RR = Risk Ratio;; CI = confidence interval; the estimated proportion of effects is the estimated proportion of RR above (or below) a threshold based on the calibrated effect sizes (Mathur & VanderWeele, 2020); I2 is an estimate of the variability in means due to heterogeneity across countries vs. sampling variability; the global *p*-value corresponds to the joint test of the null hypothesis that the country-specific joint parameter Wald tests (all parameters within variable groups are zero) are all null in all 22 countries; and additional details of heterogeneity of effects—including estimates of heterogeneity (τ) on the log-risk scale—are available in the forest plots of our online supplemental material

An important consideration in the childhood results is the sensitivity of estimates to unmeasured confounding, as reported in Table 5. Some were moderately robust. For example, to explain away the association between attending religious services at least weekly (compared to never) with EoB, an unmeasured confounder associated with both excellent health and higher EoB with RRs of 1.31 each, above and beyond measured covariates, could suffice, but weaker joint confounder associations could not; to shift the 95% confidence interval to include the null, an unmeasured confounder associated with both weekly service attendance and adult EoB with RRs of 1.23 each, above and beyond measured covariates, could suffice, but weaker joint confounder associations could not. However, other associations were less robust. Sensitivity of associations for country specific analyses are reported in the supplementary tables.

**Table 5:** Sensitivity to unmeasured confounding of childhood predictors

Variable	Category	E-value for Estimate	E-value for CI
Relationship with your mother growing up	(Ref: Very bad/somewhat bad)		
	Very good/somewhat good	1.13	1.02
Relationship with your father growing up	(Ref: Very bad/somewhat bad)		
	Very good/somewhat good	1.02	1.00
Parents married to each other when you were around 12 years old	(Ref: Parents married)		
	One or both of them had died	1.15	1.00
	Parents were divorced	1.09	1.00
	Parents were never married	1.11	1.00
	Unsure	1.12	1.00
Feelings about family’s household income when growing up	(Ref: Got by)		
	Lived comfortably	1.17	1.13
	Found it difficult	1.15	1.10
	Found it very difficult	1.18	1.08
Physically or sexually abused when growing up	(Ref: No)		
		1.19	1.12
Felt like an outsider in your family when growing up	(Ref: No)		
	Yes	1.05	1.00
Your health when growing up	(Ref: Good)		
	Excellent	1.16	1.11
	Very good	1.12	1.04
	Fair	1.08	1.00
	Poor	1.08	1.00
Born in this country	(Ref: Born in this country)		
	Born in another country	1.18	1.00
How often you attended religious services or worshiped when you were around 12 years old	(Ref: Never)		
	At least once a week	1.31	1.23
	One to three times a month	1.26	1.16
	Less than once a month	1.23	1.12
Year of birth (age group)	(Ref: 1998–2005; current age: 18–24)		
	1943 or earlier (current age: 80 + years)	1.13	1.00
	1943–1953 (current age: 70–79 years)	1.31	1.17
	1953–1963 (current age: 60–69 years)	1.31	1.19
	1963–1973 (current age: 50–59 years)	1.29	1.20
	1973–1983 (current age: 40–49 years)	1.28	1.20
	1983–1993 (current age: 30–39 years)	1.25	1.18
	1993–1998 (current age: 25–29 years)	1.17	1.12
Race plurality (prominent race/ethnic group [0] or not [1])	Non-plurality groups	1.17	1.04
FATHER_NA	Father NA flag	1.17	1.10
MOTHER_NA	Mother NA flag	1.09	1.00

The E-value is the minimum strength of the association an unmeasured confounder must have with both the outcome (EoB) and the predictor, above and beyond all measured covariates, for an unmeasured confounder to explain away an association (VanderWeele & Ding, 2017, p. 269–270).

Country-specific details for all the Tables above are provided in the supplementary files. In Table 6, we summarize these details, reporting the proportions (for demographic factors) or risk ratios (for childhood factors) across all categories for each country separately, as well as overall (in the first data column, labelled “All”).

**Table 6:** Country-level variation by categories

**Factor**	**Categories**	**All**	**Arg**	**Aus**	**Bra**	**Chi**	**Egy**	**Ger**	**HK**	**Indi**	**Indo**	**Isr**	**Jap**	**Ken**	**Mex**	**Nig**	**Phi**	**Pol**	**SA**	**Spa**	**Swe**	**Tan**	**Tur**	**UK**	**US**
Birth cohort/age	1998–2005 (current age: 18–24 years)	0.80	0.84	0.93	0.77	0.87	0.81	0.88	0.63	0.68	0.90	0.90	0.44	0.81	0.86	0.90	0.90	0.74	0.90	0.84	0.84	0.64	0.75	0.72	0.76
	1993–1998 (current age: 25–29 years)	0.77	0.78	0.93	0.70	0.82	0.77	0.89	0.43	0.62	0.90	0.90	0.32	0.79	0.83	0.90	0.90	0.73	0.91	0.77	0.82	0.62	0.78	0.79	0.82
	1983–1993 (current age: 30–39 years)	0.75	0.88	0.86	0.70	0.81	0.72	0.83	0.43	0.63	0.82	0.84	0.33	0.75	0.80	0.90	0.86	0.71	0.91	0.79	0.83	0.51	0.70	0.74	0.85
	1973–1983 (current age: 40–49 years)	0.74	0.81	0.86	0.72	0.81	0.71	0.82	0.47	0.64	0.75	0.85	0.38	0.71	0.81	0.91	0.84	0.69	0.87	0.82	0.83	0.46	0.79	0.71	0.85
	1963–1973 (current age: 50–59 years)	0.74	0.82	0.86	0.74	0.83	0.73	0.80	0.48	0.59	0.69	0.86	0.41	0.67	0.81	0.91	0.77	0.69	0.90	0.79	0.85	0.49	0.69	0.74	0.86
	1953–1963 (current age: 60–69 years)	0.73	0.79	0.87	0.75	0.83	0.66	0.84	0.43	0.51	0.63	0.81	0.50	0.66	0.83	0.79	0.82	0.63	0.93	0.79	0.84	0.46	0.71	0.73	0.88
	1943–1953 (current age: 70–79 years)	0.73	0.76	0.87	0.76	0.82	0.73	0.84	0.56	0.50	0.54	0.78	0.59	0.69	0.79	0.87	0.81	0.68	0.93	0.78	0.84	0.40	0.53	0.66	0.88
	1943 or earlier (current age: 80 + years)	0.75	0.83	0.87	0.77	1.00*	1.00*	0.81	*	0.55	0.70	0.80	0.64	0.46	0.67	0.97	0.53	0.65	0.77	0.92	0.87	0.412	0.83	0.83	0.88
Gender	Male	0.76	0.82	0.86	0.73	0.80	0.76	0.82	0.49	0.67	0.80	0.83	0.38	0.79	0.84	0.90	0.84	0.68	0.91	0.80	0.80	0.60	0.71	0.71	0.84
	Female	0.76	0.82	0.89	0.73	0.86	0.72	0.85	0.45	0.57	0.78	0.87	0.52	0.72	0.80	0.89	0.86	0.70	0.90	0.81	0.87	0.50	0.74	0.74	0.88
	Other	0.77	1.00*	1.00*	0.67	*	*	0.70	*	*	1.00*	*	0.37	1.00*	1.00*	1.00*	0.84	1.00*	*	0.04	0.92	*	*	0.57	0.95
Marital status	Single/Never been married	0.78	0.81	0.88	0.71	0.82	0.82	0.85	0.45	0.70	0.91	0.90	0.41	0.81	0.82	0.91	0.88	0.71	0.90	0.83	0.83	0.66	0.74	0.71	0.82
	Divorced	0.77	0.90	0.87	0.80	0.90	0.87	0.82	0.28	0.87	0.78	0.89	0.47	0.72	0.87	0.89	1.00*	0.65	0.98	0.71	0.84	0.37	0.59	0.69	0.86
	Domestic partner	0.77	0.84	0.88	0.75	0.83	*	0.92	0.51	0.45	0.74	0.88	0.56	0.77	0.78	0.95	0.89	0.65	0.94	0.83	0.83	0.51	*	0.75	0.87
	Married	0.75	0.80	0.88	0.75	0.82	0.72	0.83	0.49	0.62	0.76	0.83	0.44	0.73	0.84	0.89	0.83	0.69	0.90	0.80	0.85	0.51	0.73	0.73	0.88
	Separated	0.77	0.86	0.84	0.70	0.88	0.80	0.82	1.00*	0.46	0.73	0.83	0.46	0.72	0.79	0.94	0.77	0.67	0.83	0.73	0.84	0.53	0.57	0.83	0.88
	Widowed	0.73	0.78	0.87	0.71	0.84	0.70	0.80	0.36	0.44	0.64	0.76	0.59	0.65	0.78	0.84	0.82	0.70	0.89	0.78	0.83	0.48	0.71	0.73	0.85
Employment status	Employed for an employer	0.77	0.82	0.87	0.76	0.81	0.74	0.83	0.47	0.63	0.79	0.87	0.39	0.80	0.84	0.94	0.85	0.71	0.89	0.82	0.83	0.70	0.72	0.74	0.85
	Self-employed	0.79	0.88	0.89	0.731	0.85	0.80	0.89	0.41	0.64	0.80	0.84	0.53	0.74	0.84	0.89	0.86	0.69	0.91	0.84	0.88	0.55	0.71	0.84	0.91
	Homemaker	0.74	0.79	0.91	0.70	0.81	0.70	0.84	0.41	0.58	0.76	0.71	0.54	0.65	0.77	0.91	0.83	0.71	0.89	0.82	0.86	0.44	0.74	0.66	0.91
	None of these/Other	0.73	0.76	0.86	0.79	0.77	0.85	0.80	0.22	0.65	0.68	0.76	0.51	0.73	0.82	0.86	0.81	0.69	0.92	0.83	0.83	0.41	0.64	0.63	0.82
	Retired	0.74	0.73	0.87	0.78	0.84	0.68	0.82	0.48	0.51	0.74	0.82	0.53	0.66	0.79	0.86	0.83	0.64	0.91	0.77	0.85	0.40	0.75	0.71	0.88
	Student	0.83	0.85	0.93	0.82	0.85	0.85	0.90	0.86	0.73	0.96	0.95	0.50	0.86	0.88	0.89	0.92	0.73	0.85	0.84	0.86	0.76	0.69	0.70	0.86
	Unemployed and looking for a job	0.74	0.79	0.96	0.66	0.72	0.74	0.75	0.44	0.68	0.86	0.76	0.33	0.76	0.83	0.90	0.83	0.61	0.91	0.72	0.756	0.60	0.78	0.62	0.78
Religious service attendance	More than once a week	0.82	0.84	0.91	0.78	0.92	0.74	0.96	0.93	0.65	0.79	0.89	0.69	0.73	0.87	0.90	0.83	0.77	0.90	0.85	0.91	0.56	0.72	0.91	0.90
	Once a week	0.79	0.80	0.88	0.72	0.88	0.76	0.85	0.59	0.61	0.77	0.82	0.70	0.756	0.81	0.89	0.86	0.71	0.90	0.82	0.91	0.55	0.84	0.79	0.89
	One to three times a month	0.78	0.86	0.91	0.76	0.93	0.77	0.84	0.59	0.62	0.81	0.86	0.46	0.76	0.84	0.90	0.86	0.67	0.94	0.83	0.86	0.51	0.69	0.79	0.88
	A few times a year	0.78	0.83	0.89	0.74	0.86	0.78	0.88	0.46	0.65	0.82	0.87	0.54	0.78	0.83	0.89	0.84	0.67	0.89	0.80	0.89	0.57	0.70	0.80	0.87
	Never	0.73	0.82	0.87	0.68	0.81	0.72	0.81	0.43	0.55	0.80	0.82	0.42	0.73	0.78	0.90	0.86	0.68	0.85	0.79	0.81	0.43	0.65	0.67	0.84
Education	Up to 8	0.70	0.77	0.81	0.68	0.82	0.69	0.71	0.41	0.59	0.70	0.66	0.33	0.68	0.76	0.88	0.79	0.70	0.87	0.72	0.76	0.50	0.69	0.59	0.93
	9–15	0.77	0.84	0.86	0.75	0.83	0.79	0.81	0.46	0.70	0.88	0.82	0.42	0.80	0.82	0.91	0.87	0.67	0.91	0.79	0.82	0.71	0.73	0.71	0.84
	16 +	0.83	0.89	0.90	0.78	0.87	0.86	0.89	0.50	0.73	0.93	0.90	0.54	0.88	0.88	0.95	0.90	0.74	0.96	0.89	0.89	0.78	0.76	0.82	0.89
Immigration status	Born in this country	0.76	0.82	0.88	0.74	0.86	0.74	0.84	0.45	0.62	0.79	0.85	0.46	0.75	0.81	0.90	0.85	0.69	0.90	0.80	0.83	0.55	0.73	0.72	0.86
	Born in another country	0.78	0.92	0.86	0.46	0.88	0.83	0.84	0.65	0.58	0.88	0.83	0.42	0.74	0.97	0.94	0.73	0.71	0.86	0.80	0.87	0.44	0.71	0.80	0.86
Rel. with mother^1^	Very good/somewhat good	1.01	1.05	1.04	1.00	1.01	1.05	1.00	1.00	0.92	0.97	0.99	1.06	1.07	1.05	0.99	1.04	0.91	1.06	1.04	1.00	1.00	1.06	1.02	1.00
Rel. with father^1^	Very good/somewhat good	1.00	1.04	1.03	1.01	1.01	0.91	1.02	1.05	0.98	1.01	1.02	1.02	0.95	0.97	0.94	1.05	0.94	1.00	1.05	0.99	1.02	1.00	1.01	1.00
Parent marriage^2^	One or both of them had died	1.02	0.92	1.04	1.03	0.94	1.03	1.10	0.87	0.99	0.99	1.07	1.03	1.05	0.96	1.02	0.93	0.95	1.00	1.21	1.03	0.98	1.11	1.07	0.99
	Parents were divorced	1.01	1.04	1.02	0.97	0.92	0.91	1.00	1.01	1.07	1.00	0.99	1.08	1.00	0.87	1.02	0.93	0.93	1.03	1.06	1.02	0.99	1.10	1.02	1.02
	Parents were never married	1.01	1.01	1.00	1.01	0.97	1.18	1.00	1.12	1.00	1.18	0.83	0.96	1.00	0.97	1.00	1.03	0.95	1.00	1.04	0.99	0.98	1.35	0.99	1.00
	Unsure	1.01	0.98	1.18	0.88	0.86	1.05	1.02	1.04	1.08	0.96	0.69	0.96	1.12	0.95	1.01	1.12	0.98	0.77	0.85	1.02	0.92	0.91	0.95	1.02
Family income^3^	Lived comfortably	1.02	1.00	1.03	1.07	1.04	1.00	1.04	1.04	1.05	1.02	1.00	1.07	1.00	1.04	0.99	1.01	0.99	0.99	1.04	1.03	1.02	0.99	1.06	1.02
	Found it difficult	0.98	0.98	0.98	1.02	0.99	0.98	0.99	1.00	1.00	1.04	0.99	0.95	0.96	0.98	0.99	0.97	0.91	0.96	1.01	0.98	0.96	0.90	1.03	1.00
	Found it very difficult	0.97	0.99	1.06	1.04	0.98	0.95	0.99	1.14	0.95	1.08	0.91	0.95	0.93	1.00	1.01	0.97	1.00	0.98	0.87	1.01	0.91	0.81	0.99	0.99
Abused^4^		1.03	1.02	1.06	1.02	1.02	0.96	1.02	1.07	1.05	1.00		1.12	1.00	1.06	1.02	0.97	0.96	0.99	1.05	1.03	1.06	1.05	1.04	1.01
Outsider4		1.00	1.01	0.97	1.01	1.00	1.00	0.99	0.94	1.05	1.02	1.03	1.02	0.97	1.02	0.99	1.05	0.96	1.05	0.94	1.02	1.02	1.01	0.95	0.98
Health^5^	Excellent	1.02	1.03	1.02	1.01	0.98	1.08	1.06	1.13	1.05	1.03	0.98	1.05	1.01	0.99	1.02	1.02	1.00	0.99	1.07	1.00	1.07	0.99	1.01	1.02
	Very good	1.01	1.06	0.98	0.99	1.02	1.08	1.05	1.12	1.05	1.01	0.96	1.04	1.00	1.00	1.00	1.02	0.98	0.96	1.04	0.99	1.03	1.02	0.98	1.01
	Fair	1.01	1.03	1.02	0.99	1.02	1.11	1.92	0.93	1.01	1.04	0.96	1.02	1.02	0.99	0.99	0.97	1.00	0.99	1.02	0.97	1.05	0.87	0.95	1.02
	Poor	0.99	1.00	1.00	0.96	0.94	1.12	0.97	0.96	1.03	1.02	1.08	1.00	0.97	0.99	1.01	0.91	1.09	1.05	1.01	0.98	0.95	1.01	1.05	1.01
Religious attendance^5^	More than weekly	1.07	1.04	1.00	1.07	1.09	1.07	1.09	1.11	1.03	1.01	1.03	1.29	1.05	1.08	0.98	0.98	1.09	1.00	1.07	1.11	1.16	0.98	1.16	1.04
	1–3 month	1.05	1.02	0.98	1.07	1.12	1.09	1.05	1.08	1.03	0.92	0.99	1.20	1.04	0.99	0.94	0.98	1.09	0.99	1.07	1.07	1.14	1.01	1.14	1.04
	Less than once a month	1.04	1.04	0.96	1.03	1.03	1.05	1.07	1.01	1.02	1.09	1.03	1.15	0.99	1.04	0.93	0.94	0.98	0.96	0.99	1.09	1.19	1.03	1.16	1.02

^1^ Ref: Very bad/somewhat bad; ^2^ Ref: Married; ^3^ Ref: Got by; ^4^ Ref: No; ^5^ Ref: Good; ^6^ Ref: Never

## Discussion

The analysis sheds unique light on the cross-national childhood and demographic factors associated with EoB. All three of our main hypotheses were supported, often strikingly: (1) the distributions and descriptive statistics of key factors did reveal diverse patterns across our international sample; (2) mean levels of EoB varied meaningfully across countries; and (3) EoB exhibited variations across different childhood and demographic categories, and these differences across categories themselves varied by country. Here we touch briefly on the factors, beginning with the demographic then the childhood ones, ordered in terms of the degree of variation among their categories in their association with EoB (with greater variation implying more “impact,” even if we cannot demonstrate causality). First though, before delving into the factors, let us note the striking confirmation of hypothesis two, with considerable differences in EoB per se across countries.

### Cultural Variation

Responses to our item—“Do you regularly experience things that you consider beautiful? This may include physical beauty or abstract beauty like that found in music, art, or nature”—ranged dramatically, from 90% saying “yes” in South Africa (95% CI = 88, 92) and Nigeria (88, 91), to just 47% in Hong Kong (42, 52) and 44% (43, 45) in Japan. Besides our analysis of childhood and demographic factors, arguably the most notable contribution of our study is its cross-national coverage, given the Western-centricity of flourishing research. Our data show the folly of the tendency in fields like psychology to generalize from Western samples, especially in the US, to the rest of the world. The number of people reporting EoB in the US was 86%, and based on that figure one might conclude EoB is fairly common. However, this would overlook the fact 19 of 23 places had lower levels—Philippines (85%), Israel (85%), Sweden (84%), Germany (84%), China (83%), Argentina (82%), Mexico (82%), Spain (80%), Indonesia (79%), Kenya (75%), Egypt (74%), Brazil (73%), UK (73%), Turkey (72%), Poland (69%), India (62%), Tanzania (55%), Hong Kong (47%) and Japan (44%)—showing that in most nations EoB is not nearly as high. It would also obscure the fact that three countries managed *greater* EoB— Nigeria (90%), South Africa (90%), and Australia (88%).

Let’s briefly reflect on the highest EoB being in Nigeria and South Africa, two of the poorest places in the GFS. Although there may be several potential explanations, one is that a backdrop of pervasive social-structural vulnerabilities (e.g., high poverty, economic inequality) may prompt people to prioritize non-material sources of meaning as they try to cope with material insecurity (Frankl, 1963). To that point, analysis of Wave 1 GFS data found South Africa fared poorly on most socioeconomic indicators compared to all GFS countries combined, yet fared better on many wellbeing outcomes (e.g., peace, religious/spiritual connection) (Cowden et al., 2025a, 2025b, 2025c), and indeed, high EoB in South Africa may be related to its high religious/spiritual affiliation and engagement (Cowden et al., 2025b), which perhaps allow forms of beauty perception that may be less accessible to people of secular persuasion, such as appreciating the wonder of “creation” (Lomas et al., 2024). Likewise, despite similarly relatively high levels of poverty and corruption (Muoghalu & Abrifor, 2012; Sohn, 2020), Nigeria’s high EoB might reflect a culturally distinct framework of beauty, shaped by theology, interdependence, survival, and hope. Take the popular Nigerian religious catchphrase “What God cannot do does not exist,” meaning that no situation, however dire, is beyond divine intervention, and revealing a widespread belief in Nigeria that beauty is not the absence of pain, but rather the presence of divine possibility within it. While acknowledging the limitations and pitfalls of such sentiments, Agbedo (2025, para. 11) suggests it is “a powerful reminder of God’s limitless power and ability to transform lives.” This uplifting aesthetic allows individuals to perceive beauty not in the absence of hardship, but in the possibility of spiritual breakthrough within it (Counted et al., 2025). Other cultural frameworks such as *Wazobia—*a term used to fuse Nigeria’s three major languages and symbolize unity in diversity—perhaps also reflect a community-oriented aesthetic in which beauty is located in shared struggle and joy (Sunday Nnamdi et al., 2013). However, we note that Kenya and Tanzania share many affinities with Nigeria and South Africa—including also being relatively poor and having high religious adherence—yet have considerably lower EoB. These factors in themselves are thus apparently insufficient for engendering EoB, and additional explanations must be sought for why Nigeria and South Africa fared so well, which future work—including ideally qualitative analyses—can help uncover. With these cross-cultural considerations in mind, let’s proceed through the factors, beginning with demographic ones.

### Demographic Factors

The most strongly related factor overall—gauged by the difference between the categories in their association with EoB—was education, where EoB ranged from 70% for less than 8 years, to 77% for 9–15 years, to 83% for 16 + years. These data align with a burgeoning literature on the impact of education on flourishing, whether directly, such as teaching skills and qualities associated with wellbeing (Michalos, 2008), or indirectly, for instance by enhancing socio-economic prospects (Ilies et al., 2019). The causal dynamics are complicated though: while education may well engender EoB, people who are more attentive to beauty may also be more likely to reach higher levels of education, while a third factor (e.g., socioeconomics) may also affect both outcomes. Moreover, as with all broad trends across the GFS, there is considerable national variation, suggesting the relevance of factors differs considerably based on location. First, while most nations conform to the escalating pattern (more education is associated with higher EoB), a few do not, including notably the US, where the least educated had the *highest* levels (93%), above those with 8–15 years (84%) and 16 + years (89%). This reinforces the point above about the risks of academia relying on WEIRD places like the US and generalizing to the rest of the world, which here would mean assuming less education leads to higher EoB whereas actually the US is an exception in that regard. Other variation concerns the range of values: some countries had only a narrow difference, including just five percentage points in China, implying education has a relatively small impact on EoB there; by contrast, others have a much larger range (28 points in Tanzania), suggesting greater relevance.

The next largest variation was for employment status, led by students (83% reporting EoB), followed by the self-employed (79%), those employed for an employer (77%), homemakers, the retired, and unemployed job-seekers (all 74%), and “none of the above” (73%). In interpreting these figures, we should also consider age—the demographic variable with the third largest variation—whereby EoB peaked among those 18–24 (80%), thereafter decreasing through the categories: 25–29 (77%), 30–39 (75%), 40–49 and 50–59 (74%), and 60–69 and 70–79 (73%), albeit with a slight uptick for the tiny 80 + category (75%). Might high EoB among students be partly a function of age? An emergent literature shows that qualities related to EoB, such as “openness to experience” (Silvia et al., 2015), tend to increase during youth, stabilize and plateau into middle age, and decline with age (Schwaba, 2019), which seems evident here too. That may not be the *only* explanation; even compared to peers the same age, students—especially those that excel on qualities like creativity—may be *particularly* high in openness (Leung & Chiu, 2008). But the age-related pattern is striking, and perhaps rules out other potential explanation for high EoB among students, like the availability of free time. While many students do perhaps enjoy a surplus of free time compared to those in careers—at least in terms of their time-use being relatively unstructured and uncoerced by job-related strictures—the same would apply to retirees, who have far lower EoB. Perhaps then, as with openness, EoB risks fading over time, falling away from the naïve perceptual wonder and freshness of childhood (O’bi & Yang, 2024).

Age-related associations are moreover immune to the causal ambiguities that attend other variables (given we only have correlational data available here). With factors that are partially contingent on dynamics like personality—including marital, education, and employment status—one cannot be sure whether, (a) EoB is influenced by being in a particular category (e.g., being married), or (b) whether being in that category is influenced by EoB, or (c) whether a separate factor is driving both outcomes. With age though, while (c) cannot be ruled out, at least (b) can, and it becomes more likely EoB is indeed causally affected by age-related considerations. Once again though, these trends are not universal. Students do not have highest EoB in nine countries, with top rank instead taken by the self-employed in Argentina, Sweden, UK, and the US, those employed for an employer in Nigeria, unemployed job seekers in Australia and Turkey, homemakers in Japan, and “none of the above” in South Africa, where indeed students had the lowest EoB of everyone. Similarly, regarding age, the youngest participants did not have the highest EoB in 10 countries, which instead was those 25–29 in Germany, 30–39 in Argentina, 60–69 in South Africa and the US (where in fact students had the lowest EoB), and those 80 + in Japan, Nigeria, Spain, Sweden, Turkey, and UK. Thus, any generalizations about EoB being highest among students and those 18–24, while true across the GFS on aggregate, would be incorrect in nearly half the countries studied. Other variation includes the range within countries, where the gap between employment categories ranges from 45% in Hong Kong (41% for homemakers and the self-employed to 86% for students) to just 7% in South Africa (students at 85% to none of the above at 92%), implying employment is more consequential for EoB in the former than the latter. Likewise with age, the gap ranges from 37% in the Philippines (53% of those 80 + to 90% of those 18–29) to just 5% in Sweden (82% of those 25–29 to 87% for those 80 +), implying age has more of an impact on EoB in the former than the latter.

Next, EoB increased with the frequency of religious service attendance, being lowest among those who never attend (73%), followed by a few times a year or 1–3 times a month (78%), once a week (79%), culminating in more than once weekly (82%). This aligns with an extensive literature on the positive impact of religious attendance on flourishing (Koenig, 2009), to which we can now also add EoB. Strikingly, whereas weekly attendance versus a few times a year only has an increment of one percentage point, “more than once a week” is associated with a three point jump. Beyond religiosity per se, EoB thus seems enhanced by frequency of attendance, which on reflection is unsurprising given that many religious settings are *designed* with aesthetic qualities in mind and are appreciated in part for that reason (Farley, 2020). Regional variation again applies though. The lowest EoB was not always among never attenders, and included those attending once a week in Argentina, Indonesia, and Japan, more than once a week in the Philippines, and a few times a year in Poland. Conversely, highest EoB was not always in those attending more than once weekly, and included those attending weekly in the Philippines and Turkey, 1–3 times a month in China, and South Africa, and few times a year in Egypt, Indonesia, Kenya, and Tanzania. There was also variation in range, spanning 50% in Hong Kong (from never attenders at 43% to more than once weekly at 93%) to just 1% in Nigeria (possibly because religious views are more greatly diffused throughout the Nigerian populace than in Hong Kong).

Regarding marital status, EoB was lowest among the widowed (73%), then the married (75%), those separated, divorced, or with a domestic partner (77%), culminating in those single and never married (78%). The variation is not especially large but is still notable, though again age dynamics may be involved, given that older people are more likely to be widowed and younger people to be single. In terms of the categories themselves, there is a literature, for example, exploring the relative impact of widowhood and divorce, with a complex picture in terms of which can be more harmful (Lin & Brown, 2020), but in the case of EoB it seems the latter is more negative. Conversely, on many aspects of flourishing, people who are married consistently seem to fare better than other groups (Grover & Helliwell, 2019)—though the marriage quality matters (Chapman & Guven, 2016)—while the generally salutary effects of relationships are such that a partnership in general (whether married or not) is usually considered better than singlehood (Grundström et al., 2021). Yet there is increasing interest in singlehood, especially given the rising numbers of people opting for this, and the potential for it to bring benefits for some people (and hence be sought by some for those reasons) (Girme et al., 2023). To such literature we can add greater EoB, albeit only marginally. Once again though, note the regional variation. Single people did not have the highest EoB in 14 countries, which instead included divorcees in Argentina, Brazil, China, Egypt, India, and South Africa, those separated in the UK and US, the married in Mexico, Sweden, and the US, and those domestically partnered in Germany, Hong Kong, Nigeria, and the Philippines. Conversely, the lowest EoB included those separated (Brazil, the Philippines, South Africa), divorced (Spain, Tanzania, Turkey, UK), single (China, Japan, Sweden, US), married (China), and with a domestic partner (Poland, Sweden). Finally, there was considerable variation in range, spanning just 2% in Sweden to 43% in India (between 44% of those widowed to 87% of those divorced).

Lastly, two demographic factors had minimal impact: immigration status and sex. Of the former, the percentage reporting EoB was slightly higher among those born elsewhere (78%) than where they currently live (76%). Despite a common perception that being an immigrant presents challenges that can hinder flourishing (Rodriguez et al., 2021), immigrant mental health is often “better than expected” (Alegría et al., 2017), and may even be better than that of non-immigrants (Elshahat et al., 2022), as we found here. There were significant regional disparities though, with *non*-immigrants having higher EoB in 10 places (Australia, Brazil, India, Israel, Japan, Kenya, the Philippines, South Africa, Tanzania, and Turkey). There was also considerable variation in the differences, from the groups being equal in Germany, Spain, and the US, to 28% in Brazil (with non-immigrants doing better). While we cannot explore immigration dynamics in these countries, clearly immigrants face very different situations in various places, with resulting variation in EoB with respect to non-immigrants.

Finally, sex/gender essentially made no difference, with males and females exactly equal (76%, with the very small percentage of gender “other” on 77%). This finding is intriguing; however, the literature on sex differences in flourishing is complex and multifaceted (Lomas, 2025b), so it is difficult to judge how our finding fits into this overall picture, especially with an outcome as under-researched as EoB. Nevertheless, the lack of sex differences is itself notable. While one sometimes encounters gendered stereotypes in this arena—such as females being more interested in the “arts” and males more into the sciences (Hausmann, 2014), or conversely that males are more concerned with physical beauty (e.g., in romantic partners) than females—this was not borne out here. Despite parity overall however, differences actually were observed in most places—the largest being Japan, with females 14% higher, while the biggest lead for males was in India, at 10% higher—but with a nearly equal split in terms of advantage (hence the parity), with males being higher in nine places (Egypt, Hong Kong, India, Indonesia, Kenya, Mexico, Nigeria, South Africa, and Tanzania), and females in 12 (Australia, China, Germany, Israel, Japan, the Philippines, Poland, Spain, Sweden, Turkey UK, US). Arguably the places where males score higher tend to be more traditional patriarchal societies, while those where females score higher tend to be countries with greater gender equality. In general though, the same point applies as made in relation to immigration: while we cannot explore the gender dynamics in these countries, clearly males and females face very different situations in various places, with striking resultant variation in EoB.

### Childhood Factors

Several childhood factors have already been considered above, since these could be treated as either childhood or demographic factors, namely age, gender, and immigration status. From a childhood perspective, for example, age could be interpreted as reflecting a person’s birth cohort (as well as their current actual age). Nuances aside though, we have already dwelt on these factors, so now focus on those more strictly pertaining to childhood, beginning with the one with the greatest variation, namely attendance at religious services age 12, which we shall cover in some depth to show the nuances in the data.

Since current religious service attendance was itself a strong demographic factor, given the extent to which adult religious participation is shaped by childhood participation it is then unsurprising that childhood attendance is also impactful. Compared to people who never attended as a child, the risk ratios (RRs) rise with the frequency of attendance, from less than once a month (1.04), to 1–3 times a month (1.05), culminating in at least once weekly (1.07). Although this effect may seem modest, at the population level it is quite meaningful (especially given the relatively high baseline rates), and moreover, in some countries the effect size was considerably higher (as we consider shortly). We did not actually assess service attendance in childhood itself, but retrospective recollections about childhood, and people sometimes change their ratings of childhood experiences over time (Vuolo et al., 2014). Caution is therefore needed in interpreting our results. However, for recall bias to completely explain the associations, the effect of adult EoB on retrospective assessments of these childhood predictors would have to be at least as strong as the observed associations themselves (VanderWeele & Li, 2019). One can further examine the robustness of these associations through their E-values (VanderWeele & Ding, 2017), which measure the strength an unmeasured confounder would *need to be* to explain away the observed relationship. In the case of weekly attendance, the E-value was 1.31. This would mean an unmeasured confounder that was both, (a) related to EoB with a RR of 1.31 and (b) *simultaneously* related to childhood service attendance with a RR of 1.31, could explain the association away. However, especially given the observed RRs with other childhood variables that are so modest, it is difficult to envisage an unmeasured variable that could plausibly have an RR of 1.31 with EoB *and* childhood attendance, as this would need to be (a) higher than the largest one observed for EoB, and (b) apply also to attendance *itself*. So, the observed RR between service attendance and EoB *is* arguably robust to potential unmeasured confounding.

In terms of regional variation though, some RRs were below 1.00 in 11 places, suggesting that, in nearly half the countries studied, some amounts (e.g., less than once a month) of childhood attendance mean one is *less* likely to report EoB as an adult, including Australia, Indonesia, Israel, Kenya, Mexico, Nigeria, the Philippines, Poland, South Africa, Spain, and Turkey. In some of these, the RRs were only marginally under 1.00 and were also just in one category (such as an RR of 0.99 for those attending services 1–3 times a month in Israel), suggesting that, overall, attendance is still largely beneficial there. More notably though, three countries had an RR below 1.00 (0.98) for more than weekly attendance: Nigeria, Philippines, and Turkey. Furthermore, while Turkey had RRs above 1.00 for the other categories, they were below 1.00 in all three categories in Nigeria and Philippines—as much as 0.93 and 0.94 for those attending less than once a month in the respective countries. In these two places at least then attendance generally appears detrimental to adult EoB. We might also note the differences in range: some countries had only small RRs across all categories (e.g., Indonesia), whereas others were larger, especially Japan, with RRs of 1.15 (less than once a month), 1.12 (1–3 times a month), and 1.29 (weekly). Understanding such regional variation demands further in-depth study into the dynamics of religious activity and its consequences in particular places.

The next greatest variation was in relation to household income, where compared to people whose family merely “got by,” those who found it “difficult” or “very difficult” had respective RRs of 0.98 and 0.97, while those who “lived comfortably” had one of 1.02. This implies a socioeconomic aspect to EoB, whereby those with more disadvantaged backgrounds are less likely to report EoB as an adult, while those better off have higher EoB. These patterns align with a considerable literature indicating that economic security in childhood is associated with better long term wellbeing (Gibb et al., 2012). These associations were not uniform though: in eight places, those recalling their financial situation as difficult and/or very difficult were *more* likely to report EoB than those who got by (Australia, Brazil, Hong Kong, Indonesia, Nigeria, Spain, Sweden, and UK), while in four places those who were “comfortable” were less likely to report EoB (Nigeria, Poland, South Africa, and Turkey). Most striking was Hong Kong, where those who found it “very difficult” had an RR of 1.14. We cannot know from our data *why* this effect is observed—namely, what is special about places like Hong Kong that financial adversity in childhood seems to actually facilitate adult EoB. One could speculate that, at least in some countries, childhood adversity encourages or compels people to develop certain psychological qualities, such as emotional and perceptual sensitivity, that may subsequently give rise to EoB. But this still begs the question, namely, what is different about these countries that this effect is observed, which is something that demands more in-depth study.

Similar dynamics perhaps apply to whether people had been abused: people reporting suffering such abuse were, somewhat surprisingly, *more* likely to report EoB as an adult (RR = 1.03). We say “surprisingly” since abuse is rightly regarded as unequivocally bad in itself (e.g., morally) and in many of its consequences (e.g., on later wellbeing). Moreover, one is naturally wary of implying abuse can have any “positive” consequences, lest it imply some justification. Nevertheless, it remains possible that some forms of suffering and abuse—and, more broadly, the extensively-studied phenomena of Adverse Childhood Experiences—might sometimes, through struggle, engender certain qualities that people may even come to value. Although some research has indicated, for instance, that childhood abuse can mean subsequent issues around emotion perception and processing (McQuarrie et al., 2025), others have suggested that victims may come to be more perceptually attentive and responsive, such as having enhanced amygdala reactivity to emotional faces (van Harmelen et al., 2013). While one would not necessarily construe these latter outcomes as positives in themselves, they could plausibly also translate into greater openness/sensitivity to beauty. This idea also taps into a broader narrative around the notion that artistic people may be more likely to have suffered abuse and other afflictions, such as mental illness (Spaniol, 2001). One must also be wary of unduly reinforcing this narrative however, especially since the link here is not very strong, nor *universally* observed (although only Egypt, the Philippines, Poland, and South Africa were exceptions).

The remaining factors had dwindling levels of variation between the categories. Next was childhood health, where compared to people who recall having “good” health, those with “poor” health had marginally lower EoB (0.99), while those with “very good” and “excellent” health had marginally higher (1.01 and 1.02 respectively). It is not simply that better health means greater EoB though, since “fair” (below “good” on the five point scale) also had an RR of 1.01. This indication that childhood health has some bearing—albeit not a simple linear one—on adult EoB situates our findings within an extensive literature showing childhood health influences myriad aspects of adult life. Much of this existing longitudinal work focuses either on physical health or socio-economic status, with poor childhood health having a long-term detrimental impact (Mikkonen et al., 2018; Smith et al., 2012). To this we can now add EoB. The effect is fairly marginal though, while we reiterate that “fair” health has a better outcome than “good,” which complicates the dynamics. So does the cultural variation: having good or excellent health for instance was not always a boon, with RRs below 1.00 for one or both in Brazil, China, Israel, Mexico, Nigeria, the Philippines, South Africa, Sweden, Turkey, and the UK; conversely, poor health was not always detrimental, with RRs above 1.00 in Egypt, India, Indonesia, Israel, Nigeria, Poland, South Africa, Spain, Turkey, UK, and US (up to 1.12 in Egypt).

The next most consequential factor was parents’ marital status. As with abuse, there was marginally higher EoB in categories one would normally deem “worse” from a flourishing standpoint. Compared to what is usually taken as the optimal scenario of parents being married, the RRs were 1.01 for parents being divorced, or never married, and 1.02 for one or both parents having died. Most research into the impact of parental marital status on wellbeing has suggested marriage is usually the best situation, both in childhood itself (Størksen et al., 2005) and over the life course (Hansagi, 2000), even if in some situations (such as abusive marriages) divorce may be better (Kelly, 2000). Here though there was an ambivalent and even negative association, and perhaps similar dynamics are at play to that of abuse, in which adversity somehow engenders greater EoB. Also again though, this does not apply everywhere, with one or more of the non-marriage categories having an RR below 1.00 in Argentina, China, India, Indonesia, Israel, Japan, Mexico, the Philippines, Poland, Sweden, Tanzania, and the UK. There was also variation in range between the categories, including up to 0.26 in Egypt (RRs of 0.92 for divorced to 1.18 for never married).

Finally, also in the realm of relational dynamics, people’s relationship with their mother and father had a negligible effect, as did “feeling like an outsider” in one’s family growing up. Clearly only *some* aspects of family dynamics are more strongly relevant to subsequent EoB. Again though, that does not apply everywhere. Although a very/somewhat good relationship with mother was slightly beneficial to EoB overall (1.01), and a very/somewhat good relationship with father was neutral (1.00), in seven places a very/somewhat good relationship—either with one’s mother, father, or both—was *detrimental*, including Egypt, India, Israel, Mexico, Poland, Nigeria, and Sweden. Similarly, with feeling like an outsider, while overall this had no effect (1.00), there was considerable variation, having a negative impact (RRs below 1.00) in Australia, Germany, Hong Kong, Kenya, Nigeria, Poland, Spain, UK, and US), and a positive impact (RRs above 1.00) in Argentina, Brazil, India, Indonesia, Israel, Japan, Mexico, thePhilippines, South Africa, Sweden, Tanzania, and Turkey. As with all factors, the general trends are not universal or inevitable, and the local sociocultural dynamics matter and moreover deserve greater study.

## Conclusion

Aesthetic appreciation has always been central to human culture, with a burgeoning program of empirical research showing it to be important to flourishing in myriad ways (Lomas, 2016). However, research into who actually *has* such experiences has been relatively lacking, especially in any international sense. Hence the value of this study, which offers a unique glimpse into EoB across 22 countries and vis-à-vis 15 different predictors, four demographic (marital status, employment status, education status, current religious service attendance), eight childhood (relationship with mother and father, parental marital status, family income, abuse, feeling like an outsider, health, and religious service attendance), and three that pertain to both (age/birth cohort, sex, and immigration status). The results provide an unprecedented, and somewhat surprising, insight into factors linked to EoB. Overall, based on the highest EoB for each factor, one is more likely to experience beauty if, demographically, one is: highly educated; young; a student; attends religious services weekly; single; and lives in a country different to where one was born. Then, in terms of childhood factors, EoB is more likely if, as a child, one: attended religious services weekly; “lived comfortably”; experienced abuse; had excellent health; had one or both parents having died; and had a good relationship with one’s mother. Clearly this is an unusual profile, especially regarding childhood factors, which include several which one would normally regard—both intuitively, and based on empirical work—as negative and unconducive to wellbeing, especially abuse.

These general trends were not universal or inevitable though, with considerable cultural variation that is obscured by the process of aggregating results across all the countries. Consider that being an “outsider” appeared to have no impact, with an RR of 1.00. Yet in 21 of the 23 places it *did* have an impact, except these were fairly evenly balanced—a negative association in nine places versus a positive association in 12—meaning the overall trend appeared neutral. The overall profile elucidated above will therefore not obtain across all or even most countries. Take the top-ranked nation of Nigeria. It shares some trends with the GFS as a whole, with people more likely to experience beauty if highly educated, an immigrant, and reporting having been abused in childhood, but for the rest of the profile the trends were quite different, with EoB more likely if a person demographically was aged 80 +, was employed for an employer, attended religious services 1–3 times a month, and had a domestic partner, and if in childhood they had a bad relationship with their mother and father; their parents were divorced, the family found it very difficult financially, they did not feel like an outsider, and they never attended religious services. Given such cultural variation, one hesitates to offer any broad recommendations, since these mostly need to be tailored to the local context. As such, any stakeholders interested in promoting EoB are encouraged to consult the supplementary table for their country regarding which groups may need particular help. That said, some patterns still hold relatively consistently across countries, such as the highest EoB being among people with the most education, which was true everywhere except the US. This latter example also neatly illustrates another key point about this paper, namely the folly of basing one's conclusions about flourishing on data from the US—which often happens in Western-centric academia—since the US may often be an outlier from an international perspective.

The study of course has limitations. First, EoB was assessed using a one-item measure, which does not capture its complexity compared to multi-item scales which would have higher validity and reliability. There is always a trade-off though in survey research between depth and breadth: including multi-item scales would limit the number of constructs assessed, and on balance, any limitations of single item measures are outweighed—from the perspective of the GFS as a whole—by the value of including more constructs which together allow a more comprehensive assessment of flourishing (VanderWeele et al., 2025). Relatedly, this single EoB item could be regarded as overly broad, or—less charitably—quite confusing, as it covers all forms of beauty. Ideally, one could have numerous items, some covering different forms of beauty and some assessing their impact on different aspects of wellbeing. However, space for questions is limited in endeavours like the GFS, and given that, (a) most topics were also only assessed with a single item, (b) many relevant topics were not covered at all, and (c) it is unusual to have *any* questions on beauty in this kind of survey, we are grateful to have even this single item, imperfect as it may be. Second, caution is needed interpreting cross-national differences as these may be influenced by socio-cultural factors, such as variation in the way people interpret the word for “beauty” in their respective language (Lomas, 2022). Third, the data in this paper are cross-sectional, which precludes conclusions about directionality, though we could arguably construct a synthetic longitudinal study by retrospectively assessing childhood experiences, while also reporting *E*-values to assess the robustness of our findings to unmeasured confounding. Likewise, while the childhood predictors were assessed retrospectively, making the findings subject to recall bias, to completely explain away the associations, the effect of adult EoB on biasing retrospective assessments would need be at least as strong as the observed associations themselves (VanderWeele & Li, 2019). Overall, then, our study has shone a unique light on the demographic and childhood predictors of EoB, though as ever more research is needed to enable further insights into this important component of flourishing.

## Supplementary Information

Below is the link to the electronic supplementary material.

Supplementary file1 (PDF 18213 KB)

## Data Availability

Data is available through the Center for Open Science (https://www.cos.io/gfs-access-data).
