# Supplementary material for: The Prevalence and Predictors of Experiences of Beauty in 22 Countries: An International Assessment of Aesthetic Appreciation in the Global Flourishing Study
Source: Appl Res Qual Life. 2026 Feb 2;21(2):469–504. doi: 10.1007/s11482-025-10532-z (PMC13038709; doi:10.1007/s11482-025-10532-z)
Supplement: Supplementary file 1 — Supplementary file1 (PDF 18213 KB) [file 11482_2025_10532_MOESM1_ESM.pdf]

(Online Supplement

Important notes and caveats of results.

This online supplement to the Global Flourishing Study report on beauty has several important caveats to interpretation. Estimating the within country group proportion can be unstable if the group size is small (<1%) of the country sample size. In such cases, the uncertainty in the estimate leads to a multiple imputation adjusted degrees of freedom less than 1. This means there is not enough information to evaluate the uncertainty in the estimate. We flagged such cases with a "*".

Table S1a. Argentina: Demographic descriptive statistics

**Characteristic**	**N = 2,728**[1]
**Age group**	
1943 or earlier (current age: 80+ years)	50 (1.8%)
1943-1953 (current age: 70-79 years)	156 (5.7%)
1953-1963 (current age: 60-69 years)	289 (11%)
1963-1973 (current age: 50-59 years)	459 (17%)
1973-1983 (current age: 40-49 years)	537 (20%)
1983-1993 (current age: 30-39 years)	557 (20%)
1993-1998 (current age: 25-29 years)	259 (9.5%)
1998-2005 (current age: 18-24 years)	422 (15%)
(Missing)	0 (0%)
**Gender**	
1. Male	1,290 (47%)
2. Female	1,426 (52%)
3. Other	5 (0.2%)
(Missing)	7 (0.3%)
**Marital status**	
1. Single/Never been married	993 (36%)
2. Married	631 (23%)
3. Separated	172 (6.3%)
4. Divorced	124 (4.5%)
5. Widowed	189 (6.9%)
6. Domestic partner	608 (22%)
(Missing)	11 (0.4%)
**Employment**	
1. Employed for an employer	896 (33%)
2. Self-employed	788 (29%)
3. Retired	341 (13%)
4. Student	161 (5.9%)
5. Homemaker	256 (9.4%)
6. Unemployed and looking for a job	222 (8.1%)
7. None of these/Other	59 (2.2%)
(Missing)	4 (0.1%)
**Religious service attendance**	
1. More than once a week	207 (7.6%)
2. Once a week	310 (11%)
3. One to three times a month	160 (5.9%)
4. A few times a year	830 (30%)
5. Never	1,216 (45%)
(Missing)	5 (0.2%)
**Education**	
1. Up to 8	835 (31%)
2. 9-15	1,627 (60%)
3. 16+	265 (9.7%)
(Missing)	1 (<0.1%)
**Immigration**	
1. Born in this country	2,612 (96%)

**Characteristic**	**N = 2,728**[1]
2. Born in another country	113 (4.2%)
(Missing)	3 (<0.1%)
**Religious affiliation**	
1. Christianity	2,050 (75%)
10. Taoism	0 (0%)
11. Confucianism	0 (0%)
12. Primal, Animist, or Folk religion	3 (<0.1%)
13. Spiritism	0 (0%)
14. Umbanda, Candomblé, and other African-derived religions	0 (0%)
15. Chinese folk/traditional religion	0 (0%)
2. Islam	1 (<0.1%)
3. Hinduism	1 (<0.1%)
4. Buddhism	21 (0.8%)
5. Judaism	16 (0.6%)
6. Sikhism	0 (<0.1%)
7. Baha'i	0 (0%)
8. Jainism	0 (0%)
9. Shinto	0 (0%)
96. Some other religion	45 (1.7%)
97. No religion/Atheist/Agnostic	558 (20%)
(Missing)	32 (1.2%)
**RACE ETHNICITY**	
(Missing)	177 (6.5%)
101. Argentina: Asian	14 (0.5%)
102. Argentina: Black	21 (0.8%)
103. Argentina: Indigenous	55 (2.0%)
104. Argentina: Mestizo(a)	816 (30%)
105. Argentina: Mullato(a)	29 (1.1%)
106. Argentina: White	1,410 (52%)
9995. Prefer not to answer	151 (5.5%)
9996. Other	54 (2.0%)

[1]n (%)

Table S1b. Argentina: Childhood descriptive statistics

Characteristic	N = 2,728[1]
**Age**	43 (17)
**Year of birth**	
1943 or earlier (current age: 80+ years)	50 (1.8%)
1943-1953 (current age: 70-79 years)	156 (5.7%)
1953-1963 (current age: 60-69 years)	289 (11%)
1963-1973 (current age: 50-59 years)	459 (17%)
1973-1983 (current age: 40-49 years)	537 (20%)
1983-1993 (current age: 30-39 years)	557 (20%)
1993-1998 (current age: 25-29 years)	259 (9.5%)
1998-2005 (current age: 18-24 years)	422 (15%)
(Missing)	0 (0%)
**Gender**	
1. Male	1,290 (47%)
2. Female	1,426 (52%)
3. Other	5 (0.2%)
(Missing)	7 (0.3%)
**Parent marital status**	
1. Parents were married	1,738 (64%)
2. Parents were divorced	279 (10%)
3. Parents were never married	515 (19%)
4. One or both of them had died	93 (3.4%)
5. Unsure	70 (2.6%)
(Missing)	33 (1.2%)
**Age 12 religious service attendance**	
1. At least once a week	1,043 (38%)
2. One to three times a month	496 (18%)
3. Less than once a month	429 (16%)
4. Never	739 (27%)
(Missing)	21 (0.8%)
**Relationship with mother**	
1. Very good	1,759 (64%)
2. Somewhat good	614 (23%)
3. Somewhat bad	133 (4.9%)
4. Very bad	97 (3.6%)
97. (Does not apply)	109 (4.0%)
(Missing)	15 (0.5%)
**Relationship with father**	
1. Very good	1,471 (54%)
2. Somewhat good	615 (23%)
3. Somewhat bad	204 (7.5%)
4. Very bad	176 (6.4%)
97. (Does not apply)	253 (9.3%)
(Missing)	9 (0.3%)
**Outsider growing up**	
1. Yes	497 (18%)

Characteristic	N = 2,728[1]
2. No	2,193 (80%)
(Missing)	38 (1.4%)
**Abuse**	
1. Yes	556 (20%)
2. No	2,141 (78%)
(Missing)	31 (1.1%)
**Self-rated health growing up**	
1. Excellent	904 (33%)
2. Very good	771 (28%)
3. Good	772 (28%)
4. Fair	233 (8.5%)
5. Poor	48 (1.7%)
(Missing)	1 (<0.1%)
**Immigration status**	
1. Born in this country	2,612 (96%)
2. Born in another country	113 (4.2%)
(Missing)	3 (<0.1%)
**Subjective financial status of family growing up**	
1. Lived comfortably	803 (29%)
2. Got by	925 (34%)
3. Found it difficult	751 (28%)
4. Found it very difficult	239 (8.8%)
(Missing)	9 (0.3%)
**Religious affiliation**	
1. Christianity	2,403 (88%)
10. Taoism	2 (<0.1%)
11. Confucianism	0 (0%)
12. Primal, Animist, or Folk religion	4 (0.1%)
13. Spiritism	0 (0%)
14. Umbanda, Candomblé, and other African-derived religions	0 (0%)
15. Chinese folk/traditional religion	0 (0%)
2. Islam	0 (<0.1%)
3. Hinduism	0 (0%)
4. Buddhism	1 (<0.1%)
5. Judaism	22 (0.8%)
6. Sikhism	0 (0%)
7. Baha'i	0 (0%)
8. Jainism	0 (0%)
9. Shinto	0 (0%)
96. Some other religion	7 (0.3%)
97. No religion/Atheist/Agnostic	244 (9.0%)
(Missing)	45 (1.6%)
**Race/Ethnicity**	
(Missing)	177 (6.5%)
101. Argentina: Asian	14 (0.5%)
102. Argentina: Black	21 (0.8%)
103. Argentina: Indigenous	55 (2.0%)

Characteristic	N = 2,728[1]
104. Argentina: Mestizo(a)	816 (30%)
105. Argentina: Mullato(a)	29 (1.1%)
106. Argentina: White	1,410 (52%)
9995. Prefer not to answer	151 (5.5%)
9996. Other	54 (2.0%)

[1]Mean (SD); n (%)

Table S1c. Argentina: Proportions by demographic category

Variable	Category	Proportion	95% CI	SE	p-value
Age group	1998-2005 (current age: 18-24 years)	0.840	(0.780, 0.901)	0.031	9.76e-02
	1943 or earlier (current age: 80+ years)	0.833	(0.697, 0.970)	0.067	
	1943-1953 (current age: 70-79 years)	0.759	(0.656, 0.861)	0.052	
	1953-1963 (current age: 60-69 years)	0.792	(0.726, 0.857)	0.033	
	1963-1973 (current age: 50-59 years)	0.817	(0.766, 0.868)	0.026	
	1973-1983 (current age: 40-49 years)	0.811	(0.767, 0.855)	0.022	
	1983-1993 (current age: 30-39 years)	0.877	(0.842, 0.913)	0.018	
	1993-1998 (current age: 25-29 years)	0.788	(0.709, 0.866)	0.040	
Gender	Male	0.821	(0.789, 0.852)	0.016	< 1e-16
	Female	0.825	(0.796, 0.854)	0.015	
	Other	1.000	*	*	
Marital status	Single/Never been married	0.819	(0.782, 0.855)	0.018	6.16e-02
	Divorced	0.902	(0.845, 0.959)	0.029	
	Domestic partner	0.840	(0.799, 0.882)	0.021	
	Married	0.800	(0.755, 0.845)	0.023	
	Separated	0.863	(0.790, 0.936)	0.037	
	Widowed	0.783	(0.696, 0.871)	0.044	
Employment	Employed for an employer	0.823	(0.786, 0.860)	0.019	1.94e-03
	Homemaker	0.790	(0.713, 0.867)	0.039	
	None of these/Other	0.763	(0.610, 0.916)	0.076	
	Retired	0.735	(0.666, 0.805)	0.035	
	Self-employed	0.881	(0.850, 0.912)	0.016	
	Student	0.855	(0.776, 0.934)	0.040	
	Unemployed and looking for a job	0.787	(0.713, 0.861)	0.038	
Religious service attendance	Never	0.817	(0.786, 0.847)	0.016	7.30e-01
	A few times a year	0.833	(0.799, 0.868)	0.018	
	More than once a week	0.836	(0.757, 0.915)	0.040	
	Once a week	0.798	(0.724, 0.872)	0.038	

Variable	Category	Proportion	95% CI	SE	p-value
	One to three times a month	0.856	(0.789, 0.924)	0.034	
Education	9-15	0.838	(0.816, 0.861)	0.011	3.62e-04
	16+	0.892	(0.859, 0.925)	0.017	
	Up to 8	0.772	(0.720, 0.824)	0.026	
Immigration status	Born in this country	0.819	(0.796, 0.842)	0.012	2.98e-05
	Born in another country	0.925	(0.879, 0.971)	0.023	
Religious affiliation	No religion/Atheist/Agnostic	0.844	(0.803, 0.886)	0.021	3.41e-01
	Christianity	0.816	(0.790, 0.841)	0.013	
	Combined	0.869	(0.759, 0.980)	0.056	
	Spiritism	*	*	*	
	Umbanda, Candomblé, and other African-derived religions	*	*	*	
	Buddhism	*	*	*	
	Islam	*	*	*	
	Chinese folk/traditional religion	*	*	*	
	Hinduism	*	*	*	
	Judaism	*	*	*	
	Primal, Animist, or Folk religion	*	*	*	

Table S1d. Argentina: Childhood predictors regression analysis

Variable	Category	Risk-Ratio	logRR SE	RR 95% CI	Global p-value
Relationship with your mother growing up	(Ref: Very bad/somewhat bad)				0.342
	Very good/somewhat good	1.045	0.042	(0.96,1.13)	
Relationship with your father growing up	(Ref: Very bad/somewhat bad)				0.313
	Very good/somewhat good	1.040	0.035	(0.97,1.11)	
Parents married to each other when you were around 12 years old	(Ref: Parents married)				0.519
	One or both of them had died	0.922	0.063	(0.82,1.04)	
	Parents were divorced	1.042	0.034	(0.98,1.11)	
	Parents were never married	1.011	0.034	(0.95,1.08)	
	Unsure	0.987	0.080	(0.84,1.16)	
Feelings about familys household income when growing up	(Ref: Got by)				0.733
	Found it difficult	0.983	0.026	(0.93,1.03)	
	Found it very difficult	0.991	0.044	(0.91,1.08)	
	Lived comfortably	1.003	0.026	(0.95,1.05)	
Physically or sexually abused when growing up	(Ref: No)				0.400
		1.022	0.025	(0.97,1.07)	
Felt like an outsider in your family when growing up	(Ref: No)				0.567
	Yes	1.009	0.031	(0.95,1.07)	
Your health when growing up	(Ref: Good)				0.198
	Excellent	1.033	0.028	(0.98,1.09)	
	Fair	1.033	0.044	(0.95,1.13)	
	Poor	1.004	0.086	(0.85,1.19)	
	Very good	1.060	0.029	(1.00,1.12)	

Variable	Category	Risk-Ratio	logRR SE	RR 95% CI	Global p-value
Born in This country	(Ref: Born in this country)				2.75e-04
	Born in another country	1.109	0.028	(1.05,1.17)	
How Often You Attended Religious Services or Worshiped When You Were Around 12 Years Old	(Ref: Never)				0.349
	At least once a week	1.042	0.027	(0.99,1.10)	
	Less than once a month	1.036	0.033	(0.97,1.10)	
	One to three times a month	1.016	0.033	(0.95,1.09)	
Year of birth (age group)	(Ref: 1998-2005; current age: 18-24)				0.696
	1943 or earlier (current age: 80+ years)	1.008	0.081	(0.86,1.18)	
	1943-1953 (current age: 70-79 years)	0.938	0.058	(0.84,1.05)	
	1953-1963 (current age: 60-69 years)	0.965	0.048	(0.88,1.06)	
	1963-1973 (current age: 50-59 years)	0.988	0.043	(0.91,1.08)	
	1973-1983 (current age: 40-49 years)	0.979	0.037	(0.91,1.05)	
	1983-1993 (current age: 30-39 years)	1.041	0.038	(0.97,1.12)	
	1993-1998 (current age: 25-29 years)	0.945	0.051	(0.86,1.04)	
Religion when twelve years old	(Ref: No religion/Atheist/Agnostic)				0.066
	Christianity	0.997	0.039	(0.92,1.08)	
	Collapsed affiliations with prevalence<3%	1.153	0.065	(1.02,1.31)	
Race plurality (prominent race/ethnic group [0] or not [1])	(Ref: Plurality group)				0.226
	Non-plurality groups	1.028	0.021	(0.99,1.07)	

Table S1e. Argentina: Sensitivity to unmeasured confounding of childhood predictors

Variable	Category	E-value for Estimate	E-value for 95% CI
Relationship with your mother growing up	(Ref: Very bad/somewhat bad)		
	Very good/somewhat good	1.26	1.00
Relationship with your father growing up	(Ref: Very bad/somewhat bad)		
	Very good/somewhat good	1.24	1.00
Parents married to each other when you were around 12 years old	(Ref: Parents married)		
	One or both of them had died	1.39	1.00
	Parents were divorced	1.25	1.00
	Parents were never married	1.12	1.00
	Unsure	1.13	1.00
Feelings about familys household income when growing up	(Ref: Got by)		
	Found it difficult	1.15	1.00
	Found it very difficult	1.11	1.00
	Lived comfortably	1.05	1.00
Physically or sexually abused when growing up	(Ref: No)		
		1.17	1.00
Felt like an outsider in your family when growing up	(Ref: No)		
	Yes	1.11	1.00
Your health when growing up	(Ref: Good)		
	Excellent	1.22	1.00
	Fair	1.22	1.00
	Poor	1.07	1.00
	Very good	1.31	1.05
Born in This country	(Ref: Born in this country)		
	Born in another country	1.46	1.27
How Often You Attended Religious Services or Worshiped When You Were Around 12 Years Old	(Ref: Never)		
	At least once a week	1.25	1.00
	Less than once a month	1.23	1.00
	One to three times a month	1.15	1.00
Year of birth (age group)	(Ref: 1998-2005; current age: 18-24)		
	1943 or earlier (current age: 80+ years)	1.10	1.00
	1943-1953 (current age: 70-	1.33	1.00

Variable	Category	E-value for Estimate	E-value for 95% CI
	79 years)		
	1953-1963 (current age: 60-69 years)	1.23	1.00
	1963-1973 (current age: 50-59 years)	1.12	1.00
	1973-1983 (current age: 40-49 years)	1.17	1.00
	1983-1993 (current age: 30-39 years)	1.25	1.00
	1993-1998 (current age: 25-29 years)	1.31	1.00
Religion when twelve years old	(Ref: No religion/Atheist/Agnostic)		
	Christianity	1.05	1.00
	Collapsed affiliations with prevalence<3%	1.57	1.14
Race plurality (prominent race/ethnic group [0] or not [1])	(Ref: Plurality group)		
	Non-plurality groups	1.20	1.00

Table S2a. Australia: Demographic descriptive statistics

**Characteristic**	**N = 2,533**[1]
**Age group**	
1943 or earlier (current age: 80+ years)	112 (4.4%)
1943-1953 (current age: 70-79 years)	311 (12%)
1953-1963 (current age: 60-69 years)	380 (15%)
1963-1973 (current age: 50-59 years)	456 (18%)
1973-1983 (current age: 40-49 years)	389 (15%)
1983-1993 (current age: 30-39 years)	454 (18%)
1993-1998 (current age: 25-29 years)	206 (8.1%)
1998-2005 (current age: 18-24 years)	226 (8.9%)
(Missing)	0 (<0.1%)
**Gender**	
1. Male	1,241 (49%)
2. Female	1,264 (50%)
3. Other	24 (0.9%)
(Missing)	4 (0.2%)
**Marital status**	
1. Single/Never been married	599 (24%)
2. Married	1,152 (45%)
3. Separated	108 (4.3%)
4. Divorced	215 (8.5%)
5. Widowed	146 (5.8%)
6. Domestic partner	301 (12%)
(Missing)	12 (0.5%)
**Employment**	
1. Employed for an employer	1,271 (50%)
2. Self-employed	249 (9.8%)
3. Retired	588 (23%)
4. Student	111 (4.4%)
5. Homemaker	85 (3.4%)
6. Unemployed and looking for a job	84 (3.3%)
7. None of these/Other	140 (5.5%)
(Missing)	4 (0.1%)
**Religious service attendance**	
1. More than once a week	113 (4.5%)
2. Once a week	215 (8.5%)
3. One to three times a month	75 (3.0%)
4. A few times a year	397 (16%)
5. Never	1,727 (68%)
(Missing)	5 (0.2%)
**Education**	
1. Up to 8	55 (2.2%)
2. 9-15	1,632 (64%)
3. 16+	838 (33%)
(Missing)	8 (0.3%)
**Immigration**	
1. Born in this country	1,999 (79%)

Characteristic	N = 2,533[1]
2. Born in another country	527 (21%)
(Missing)	8 (0.3%)
**Religious affiliation**	
1. Christianity	1,026 (40%)
10. Taoism	5 (0.2%)
11. Confucianism	0 (0%)
12. Primal, Animist, or Folk religion	10 (0.4%)
13. Spiritism	0 (0%)
14. Umbanda, Candomblé, and other African-derived religions	0 (0%)
15. Chinese folk/traditional religion	0 (0%)
2. Islam	31 (1.2%)
3. Hinduism	8 (0.3%)
4. Buddhism	18 (0.7%)
5. Judaism	13 (0.5%)
6. Sikhism	5 (0.2%)
7. Baha'i	4 (0.2%)
8. Jainism	0 (0%)
9. Shinto	0 (0%)
96. Some other religion	37 (1.5%)
97. No religion/Atheist/Agnostic	1,366 (54%)
(Missing)	10 (0.4%)
**RACE ETHNICITY**	
(Missing)	9 (0.3%)
201. Australia: Aboriginal	31 (1.2%)
202. Australia: Australian	1,283 (51%)
203. Australia: Australian British/European	721 (28%)
204. Australia: Chinese	48 (1.9%)
205. Australia: Indian	21 (0.8%)
206. Australia: Japanese	0 (<0.1%)
207. Australia: Malay	10 (0.4%)
208. Australia: Sinhalese	0 (<0.1%)
209. Australia: Spanish	0 (<0.1%)
210. Australia: Sri Lankan Moor	0 (<0.1%)
211. Australia: Sri Lankan Tamil	5 (0.2%)
212. Australia: Vietnamese	6 (0.2%)
214. Australia: Russian	7 (0.3%)
216. Australia: New Zealander	63 (2.5%)
217. Australia: Other European	233 (9.2%)
9996. Other	94 (3.7%)

[1]n (%)

Table S2b. Australia: Childhood descriptive statistics

**Characteristic**	**N = 2,533**[1]
**Age**	49 (18)
**Year of birth**	
1943 or earlier (current age: 80+ years)	112 (4.4%)
1943-1953 (current age: 70-79 years)	311 (12%)
1953-1963 (current age: 60-69 years)	380 (15%)
1963-1973 (current age: 50-59 years)	456 (18%)
1973-1983 (current age: 40-49 years)	389 (15%)
1983-1993 (current age: 30-39 years)	454 (18%)
1993-1998 (current age: 25-29 years)	206 (8.1%)
1998-2005 (current age: 18-24 years)	226 (8.9%)
(Missing)	0 (<0.1%)
**Gender**	
1. Male	1,241 (49%)
2. Female	1,264 (50%)
3. Other	24 (0.9%)
(Missing)	4 (0.2%)
**Parent marital status**	
1. Parents were married	2,031 (80%)
2. Parents were divorced	318 (13%)
3. Parents were never married	106 (4.2%)
4. One or both of them had died	62 (2.5%)
5. Unsure	7 (0.3%)
(Missing)	9 (0.4%)
**Age 12 religious service attendance**	
1. At least once a week	919 (36%)
2. One to three times a month	291 (11%)
3. Less than once a month	406 (16%)
4. Never	871 (34%)
(Missing)	46 (1.8%)
**Relationship with mother**	
1. Very good	1,658 (65%)
2. Somewhat good	644 (25%)
3. Somewhat bad	142 (5.6%)
4. Very bad	67 (2.6%)
97. (Does not apply)	23 (0.9%)
(Missing)	0 (<0.1%)
**Relationship with father**	
1. Very good	1,338 (53%)
2. Somewhat good	737 (29%)
3. Somewhat bad	218 (8.6%)
4. Very bad	133 (5.3%)
97. (Does not apply)	102 (4.0%)
(Missing)	5 (0.2%)
**Outsider growing up**	
1. Yes	505 (20%)
2. No	2,009 (79%)
(Missing)	19 (0.7%)

Characteristic	N = 2,533[1]
**Abuse**	
1. Yes	659 (26%)
2. No	1,852 (73%)
(Missing)	22 (0.9%)
**Self-rated health growing up**	
1. Excellent	1,111 (44%)
2. Very good	728 (29%)
3. Good	411 (16%)
4. Fair	226 (8.9%)
5. Poor	56 (2.2%)
(Missing)	1 (<0.1%)
**Immigration status**	
1. Born in this country	1,999 (79%)
2. Born in another country	527 (21%)
(Missing)	8 (0.3%)
**Subjective financial status of family growing up**	
1. Lived comfortably	1,107 (44%)
2. Got by	1,028 (41%)
3. Found it difficult	292 (12%)
4. Found it very difficult	98 (3.9%)
(Missing)	8 (0.3%)
**Religious affiliation**	
1. Christianity	1,747 (69%)
10. Taoism	1 (<0.1%)
11. Confucianism	0 (0%)
12. Primal, Animist, or Folk religion	1 (<0.1%)
13. Spiritism	0 (0%)
14. Umbanda, Candomblé, and other African-derived religions	0 (0%)
15. Chinese folk/traditional religion	0 (0%)
2. Islam	31 (1.2%)
3. Hinduism	12 (0.5%)
4. Buddhism	1 (<0.1%)
5. Judaism	13 (0.5%)
6. Sikhism	4 (0.1%)
7. Baha'i	2 (<0.1%)
8. Jainism	0 (0%)
9. Shinto	0 (0%)
96. Some other religion	7 (0.3%)
97. No religion/Atheist/Agnostic	702 (28%)
(Missing)	13 (0.5%)
**Race/Ethnicity**	
(Missing)	9 (0.3%)
201. Australia: Aboriginal	31 (1.2%)
202. Australia: Australian	1,283 (51%)
203. Australia: Australian British/European	721 (28%)
204. Australia: Chinese	48 (1.9%)
205. Australia: Indian	21 (0.8%)

**Characteristic**	**N = 2,533**[1]
206. Australia: Japanese	0 (<0.1%)
207. Australia: Malay	10 (0.4%)
208. Australia: Sinhalese	0 (<0.1%)
209. Australia: Spanish	0 (<0.1%)
210. Australia: Sri Lankan Moor	0 (<0.1%)
211. Australia: Sri Lankan Tamil	5 (0.2%)
212. Australia: Vietnamese	6 (0.2%)
214. Australia: Russian	7 (0.3%)
216. Australia: New Zealander	63 (2.5%)
217. Australia: Other European	233 (9.2%)
9996. Other	94 (3.7%)

[1]Mean (SD); n (%)

Table S2c. Australia: Proportions by demographic category

Variable	Category	Proportion	95% CI	SE	p-value
Age group	1998-2005 (current age: 18-24 years)	0.933	(0.875, 0.991)	0.029	3.29e-01
	1943 or earlier (current age: 80+ years)	0.873	(0.796, 0.950)	0.039	
	1943-1953 (current age: 70-79 years)	0.865	(0.816, 0.914)	0.025	
	1953-1963 (current age: 60-69 years)	0.873	(0.833, 0.912)	0.020	
	1963-1973 (current age: 50-59 years)	0.856	(0.815, 0.898)	0.021	
	1973-1983 (current age: 40-49 years)	0.864	(0.816, 0.912)	0.025	
	1983-1993 (current age: 30-39 years)	0.863	(0.810, 0.916)	0.027	
	1993-1998 (current age: 25-29 years)	0.925	(0.866, 0.984)	0.030	
Gender	Male	0.859	(0.831, 0.887)	0.014	< 1e-16
	Female	0.889	(0.864, 0.914)	0.013	
	Other	1.000	*	*	
Marital status	Single/Never been married	0.875	(0.832, 0.918)	0.022	9.80e-01
	Divorced	0.866	(0.809, 0.923)	0.029	
	Domestic partner	0.881	(0.821, 0.941)	0.031	
	Married	0.879	(0.855, 0.903)	0.012	
	Separated	0.841	(0.736, 0.946)	0.053	
	Widowed	0.873	(0.800, 0.946)	0.037	
Employment	Employed for an employer	0.866	(0.839, 0.894)	0.014	1.26e-02
	Homemaker	0.911	(0.830, 0.991)	0.040	
	None of these/Other	0.861	(0.777, 0.945)	0.043	
	Retired	0.865	(0.830, 0.899)	0.018	
	Self-employed	0.889	(0.834, 0.945)	0.028	
	Student	0.930	(0.841, 1.000)	0.045	
	Unemployed and looking for a job	0.958	(0.915, 1.000)	0.022	
Religious service attendance	Never	0.868	(0.844, 0.891)	0.012	6.34e-01
	A few times a year	0.891	(0.850, 0.932)	0.021	
	More than once a week	0.914	(0.837, 0.990)	0.038	
	Once a week	0.876	(0.814, 0.938)	0.031	
	One to three times a month	0.910	(0.820, 1.000)	0.045	

Variable	Category	Proportion	95% CI	SE	p-value
Education	9-15	0.862	(0.837, 0.887)	0.013	3.35e-02
	16+	0.905	(0.882, 0.928)	0.012	
	Up to 8	0.809	(0.631, 0.986)	0.088	
Immigration status	Born in this country	0.879	(0.858, 0.899)	0.010	4.70e-01
	Born in another country	0.862	(0.821, 0.903)	0.021	
Religious affiliation	No religion/Atheist/Agnostic	0.884	(0.860, 0.909)	0.013	5.85e-01
	Christianity	0.865	(0.836, 0.893)	0.015	
	Combined	0.867	(0.771, 0.963)	0.049	
	Spiritism	*	*	*	
	Umbanda, Candomblé, and other African-derived religions	*	*	*	
	Buddhism	*	*	*	
	Islam	*	*	*	
	Chinese folk/traditional religion	*	*	*	
	Hinduism	*	*	*	
	Judaism	*	*	*	
	Primal, Animist, or Folk religion	*	*	*	

Table S2d. Australia: Childhood predictors regression analysis

Variable	Category	Risk-Ratio	logRR SE	RR 95% CI	Global p-value
Relationship with your mother growing up	(Ref: Very bad/somewhat bad)				0.369
	Very good/somewhat good	1.041	0.041	(0.96,1.13)	
Relationship with your father growing up	(Ref: Very bad/somewhat bad)				0.382
	Very good/somewhat good	1.030	0.032	(0.97,1.10)	
Parents married to each other when you were around 12 years old	(Ref: Parents married)				1.34e-05
	One or both of them had died	1.042	0.073	(0.90,1.20)	
	Parents were divorced	1.016	0.032	(0.95,1.08)	
	Parents were never married	0.999	0.065	(0.88,1.13)	
	Unsure	1.197	0.039	(1.11,1.29)	
Feelings about familys household income when growing up	(Ref: Got by)				0.404
	Found it difficult	0.977	0.033	(0.92,1.04)	
	Found it very difficult	1.055	0.045	(0.97,1.15)	
	Lived comfortably	1.026	0.020	(0.99,1.07)	
Physically or sexually abused when growing up	(Ref: No)				0.004
		1.063	0.021	(1.02,1.11)	
Felt like an outsider in your family when growing up	(Ref: No)				0.293
	Yes	0.967	0.029	(0.91,1.02)	
Your health when growing up	(Ref: Good)				0.711
	Excellent	1.023	0.028	(0.97,1.08)	
	Fair	1.023	0.039	(0.95,1.10)	
	Poor	1.002	0.078	(0.86,1.17)	
	Very good	0.978	0.030	(0.92,1.04)	
Born in This	(Ref: Born in this				0.415

Variable	Category	Risk-Ratio	logRR SE	RR 95% CI	Global p-value
country	country)				
	Born in another country	1.024	0.028	(0.97,1.08)	
How Often You Attended Religious Services or Worshiped When You Were Around 12 Years Old	(Ref: Never)				0.461
	At least once a week	1.013	0.025	(0.96,1.06)	
	Less than once a month	0.958	0.031	(0.90,1.02)	
	One to three times a month	0.975	0.036	(0.91,1.05)	
Year of birth (age group)	(Ref: 1998-2005; current age: 18-24)				0.010
	1943 or earlier (current age: 80+ years)	0.913	0.048	(0.83,1.00)	
	1943-1953 (current age: 70-79 years)	0.898	0.040	(0.83,0.97)	
	1953-1963 (current age: 60-69 years)	0.912	0.035	(0.85,0.98)	
	1963-1973 (current age: 50-59 years)	0.897	0.037	(0.83,0.96)	
	1973-1983 (current age: 40-49 years)	0.907	0.038	(0.84,0.98)	
	1983-1993 (current age: 30-39 years)	0.915	0.040	(0.85,0.99)	
	1993-1998 (current age: 25-29 years)	0.966	0.044	(0.89,1.05)	
Religion when twelve years old	(Ref: No religion/Atheist/Agnostic)				0.383
	Christianity	0.984	0.026	(0.94,1.04)	
	Collapsed affiliations with prevalence<3%	0.907	0.075	(0.78,1.05)	
Race plurality (prominent race/ethnic group [0] or not [1])	(Ref: Plurality group)				0.057
	Non-plurality groups	0.942	0.030	(0.89,1.00)	

Table S2e. Australia: Sensitivity to unmeasured confounding of childhood predictors

Variable	Category	E-value for Estimate	E-value for 95% CI
Relationship with your mother growing up	(Ref: Very bad/somewhat bad)		
	Very good/somewhat good	1.25	1.00
Relationship with your father growing up	(Ref: Very bad/somewhat bad)		
	Very good/somewhat good	1.21	1.00
Parents married to each other when you were around 12 years old	(Ref: Parents married)		
	One or both of them had died	1.25	1.00
	Parents were divorced	1.15	1.00
	Parents were never married	1.04	1.00
	Unsure	1.68	1.46
Feelings about familys household income when growing up	(Ref: Got by)		
	Found it difficult	1.18	1.00
	Found it very difficult	1.30	1.00
	Lived comfortably	1.19	1.00
Physically or sexually abused when growing up	(Ref: No)		
		1.32	1.16
Felt like an outsider in your family when growing up	(Ref: No)		
	Yes	1.22	1.00
Your health when growing up	(Ref: Good)		
	Excellent	1.18	1.00
	Fair	1.17	1.00
	Poor	1.04	1.00
	Very good	1.17	1.00
Born in This country	(Ref: Born in this country)		
	Born in another country	1.18	1.00
How Often You Attended Religious Services or Worshiped When You Were Around 12 Years Old	(Ref: Never)		
	At least once a week	1.13	1.00
	Less than once a month	1.26	1.00
	One to three times a month	1.19	1.00
Year of birth (age group)	(Ref: 1998-2005; current age: 18-24)		
	1943 or earlier (current age: 80+ years)	1.42	1.00
	1943-1953 (current age: 70-79 years)	1.47	1.21

Variable	Category	E-value for Estimate	E-value for 95% CI
	1953-1963 (current age: 60-69 years)	1.42	1.18
	1963-1973 (current age: 50-59 years)	1.47	1.23
	1973-1983 (current age: 40-49 years)	1.44	1.17
	1983-1993 (current age: 30-39 years)	1.41	1.11
	1993-1998 (current age: 25-29 years)	1.22	1.00
Religion when twelve years old	(Ref: No religion/Atheist/Agnostic)		
	Christianity	1.14	1.00
	Collapsed affiliations with prevalence<3%	1.44	1.00
Race plurality (prominent race/ethnic group [0] or not [1])	(Ref: Plurality group)		
	Non-plurality groups	1.32	1.01

Table S3a. Brazil: Demographic descriptive statistics

Characteristic	N = 4,044[1]
**Age group**	
1943 or earlier (current age: 80+ years)	54 (1.3%)
1943-1953 (current age: 70-79 years)	202 (5.0%)
1953-1963 (current age: 60-69 years)	482 (12%)
1963-1973 (current age: 50-59 years)	636 (16%)
1973-1983 (current age: 40-49 years)	784 (19%)
1983-1993 (current age: 30-39 years)	888 (22%)
1993-1998 (current age: 25-29 years)	389 (9.6%)
1998-2005 (current age: 18-24 years)	609 (15%)
(Missing)	0 (0%)
**Gender**	
1. Male	1,934 (48%)
2. Female	2,094 (52%)
3. Other	12 (0.3%)
(Missing)	3 (<0.1%)
**Marital status**	
1. Single/Never been married	1,421 (35%)
2. Married	1,415 (35%)
3. Separated	175 (4.3%)
4. Divorced	224 (5.5%)
5. Widowed	179 (4.4%)
6. Domestic partner	581 (14%)
(Missing)	48 (1.2%)
**Employment**	
1. Employed for an employer	1,089 (27%)
2. Self-employed	877 (22%)
3. Retired	526 (13%)
4. Student	213 (5.3%)
5. Homemaker	423 (10%)
6. Unemployed and looking for a job	754 (19%)
7. None of these/Other	136 (3.4%)
(Missing)	26 (0.6%)
**Religious service attendance**	
1. More than once a week	781 (19%)
2. Once a week	711 (18%)
3. One to three times a month	407 (10%)
4. A few times a year	1,142 (28%)
5. Never	983 (24%)
(Missing)	19 (0.5%)
**Education**	
1. Up to 8	1,105 (27%)
2. 9-15	2,265 (56%)
3. 16+	671 (17%)
(Missing)	3 (<0.1%)
**Immigration**	
1. Born in this country	3,944 (98%)
2. Born in another country	40 (1.0%)

**Characteristic**	**N = 4,044**[1]
(Missing)	60 (1.5%)
**Religious affiliation**	
1. Christianity	3,048 (75%)
10. Taoism	0 (<0.1%)
11. Confucianism	3 (<0.1%)
12. Primal, Animist, or Folk religion	2 (<0.1%)
13. Spiritism	210 (5.2%)
14. Umbanda, Candomblé, and other African-derived religions	144 (3.6%)
15. Chinese folk/traditional religion	0 (0%)
2. Islam	2 (<0.1%)
3. Hinduism	0 (0%)
4. Buddhism	8 (0.2%)
5. Judaism	7 (0.2%)
6. Sikhism	0 (0%)
7. Baha'i	0 (0%)
8. Jainism	0 (0%)
9. Shinto	0 (0%)
96. Some other religion	49 (1.2%)
97. No religion/Atheist/Agnostic	547 (14%)
(Missing)	25 (0.6%)
**RACE ETHNICITY**	
(Missing)	95 (2.4%)
301. Brazil: Branca	1,671 (41%)
302. Brazil: Preta	482 (12%)
303. Brazil: Parda	1,599 (40%)
304. Brazil: Amarela	75 (1.8%)
305. Brazil: Indígena	40 (1.0%)
9995. Prefer not to answer	64 (1.6%)
9996. Other	18 (0.4%)

[1]n (%)

Table S3a. Brazil: Childhood descriptive statistics

**Characteristic**	**N = 4,044**[1]
**Age**	43 (16)
**Year of birth**	
1943 or earlier (current age: 80+ years)	54 (1.3%)
1943-1953 (current age: 70-79 years)	202 (5.0%)
1953-1963 (current age: 60-69 years)	482 (12%)
1963-1973 (current age: 50-59 years)	636 (16%)
1973-1983 (current age: 40-49 years)	784 (19%)
1983-1993 (current age: 30-39 years)	888 (22%)
1993-1998 (current age: 25-29 years)	389 (9.6%)
1998-2005 (current age: 18-24 years)	609 (15%)
(Missing)	0 (0%)
**Gender**	
1. Male	1,934 (48%)
2. Female	2,094 (52%)
3. Other	12 (0.3%)
(Missing)	3 (<0.1%)
**Parent marital status**	
1. Parents were married	2,741 (68%)
2. Parents were divorced	352 (8.7%)
3. Parents were never married	650 (16%)
4. One or both of them had died	161 (4.0%)
5. Unsure	82 (2.0%)
(Missing)	57 (1.4%)
**Age 12 religious service attendance**	
1. At least once a week	2,045 (51%)
2. One to three times a month	680 (17%)
3. Less than once a month	760 (19%)
4. Never	543 (13%)
(Missing)	16 (0.4%)
**Relationship with mother**	
1. Very good	2,607 (64%)
2. Somewhat good	1,060 (26%)
3. Somewhat bad	129 (3.2%)
4. Very bad	61 (1.5%)
97. (Does not apply)	174 (4.3%)
(Missing)	13 (0.3%)
**Relationship with father**	
1. Very good	1,902 (47%)
2. Somewhat good	1,165 (29%)
3. Somewhat bad	316 (7.8%)
4. Very bad	236 (5.8%)
97. (Does not apply)	389 (9.6%)
(Missing)	36 (0.9%)
**Outsider growing up**	
1. Yes	559 (14%)
2. No	3,416 (84%)
(Missing)	69 (1.7%)

Characteristic	N = 4,044[1]
**Abuse**	
1. Yes	884 (22%)
2. No	3,104 (77%)
(Missing)	57 (1.4%)
**Self-rated health growing up**	
1. Excellent	1,512 (37%)
2. Very good	1,006 (25%)
3. Good	962 (24%)
4. Fair	481 (12%)
5. Poor	79 (2.0%)
(Missing)	4 (0.1%)
**Immigration status**	
1. Born in this country	3,944 (98%)
2. Born in another country	40 (1.0%)
(Missing)	60 (1.5%)
**Subjective financial status of family growing up**	
1. Lived comfortably	1,519 (38%)
2. Got by	1,338 (33%)
3. Found it difficult	813 (20%)
4. Found it very difficult	352 (8.7%)
(Missing)	22 (0.6%)
**Religious affiliation**	
1. Christianity	3,552 (88%)
10. Taoism	1 (<0.1%)
11. Confucianism	4 (<0.1%)
12. Primal, Animist, or Folk religion	4 (0.1%)
13. Spiritism	79 (2.0%)
14. Umbanda, Candomblé, and other African-derived religions	71 (1.8%)
15. Chinese folk/traditional religion	0 (0%)
2. Islam	5 (0.1%)
3. Hinduism	0 (<0.1%)
4. Buddhism	6 (0.2%)
5. Judaism	8 (0.2%)
6. Sikhism	0 (0%)
7. Baha'i	0 (0%)
8. Jainism	1 (<0.1%)
9. Shinto	1 (<0.1%)
96. Some other religion	19 (0.5%)
97. No religion/Atheist/Agnostic	266 (6.6%)
(Missing)	27 (0.7%)
**Race/Ethnicity**	
(Missing)	95 (2.4%)
301. Brazil: Branca	1,671 (41%)
302. Brazil: Preta	482 (12%)
303. Brazil: Parda	1,599 (40%)
304. Brazil: Amarela	75 (1.8%)
305. Brazil: Indígena	40 (1.0%)

**Characteristic**	**N = 4,044**[1]
9995. Prefer not to answer	64 (1.6%)
9996. Other	18 (0.4%)

[1]Mean (SD); n (%)

Table S3c. Brazil: Proportions by demographic category

Variable	Category	Proportion	95% CI	SE	p-value
Age group	1998-2005 (current age: 18-24 years)	0.777	(0.728, 0.827)	0.025	3.53e-01
	1943 or earlier (current age: 80+ years)	0.769	(0.606, 0.932)	0.081	
	1943-1953 (current age: 70-79 years)	0.757	(0.655, 0.858)	0.052	
	1953-1963 (current age: 60-69 years)	0.749	(0.688, 0.810)	0.031	
	1963-1973 (current age: 50-59 years)	0.744	(0.700, 0.788)	0.022	
	1973-1983 (current age: 40-49 years)	0.723	(0.683, 0.762)	0.020	
	1983-1993 (current age: 30-39 years)	0.704	(0.664, 0.745)	0.021	
	1993-1998 (current age: 25-29 years)	0.696	(0.633, 0.760)	0.032	
Gender	Male	0.734	(0.706, 0.761)	0.014	9.49e-01
	Female	0.733	(0.708, 0.759)	0.013	
	Other	0.672	(0.204, 1.000)	0.196	
Marital status	Single/Never been married	0.709	(0.675, 0.744)	0.018	1.01e-01
	Divorced	0.804	(0.742, 0.865)	0.031	
	Domestic partner	0.753	(0.709, 0.798)	0.023	
	Married	0.746	(0.716, 0.776)	0.015	
	Separated	0.695	(0.591, 0.800)	0.053	
	Widowed	0.706	(0.594, 0.817)	0.056	
Employment	Employed for an employer	0.758	(0.728, 0.787)	0.015	1.79e-03
	Homemaker	0.679	(0.613, 0.744)	0.033	
	None of these/Other	0.789	(0.682, 0.896)	0.054	
	Retired	0.783	(0.727, 0.839)	0.028	
	Self-employed	0.731	(0.692, 0.769)	0.020	
	Student	0.821	(0.741, 0.902)	0.041	
	Unemployed and looking for a job	0.662	(0.610, 0.714)	0.026	
Religious service attendance	Never	0.681	(0.639, 0.723)	0.021	1.50e-02
	A few times a year	0.744	(0.710, 0.778)	0.017	
	More than once a week	0.781	(0.737, 0.824)	0.022	
	Once a week	0.720	(0.675, 0.766)	0.023	
	One to three times	0.760	(0.706, 0.813)	0.027	

Variable	Category	Proportion	95% CI	SE	p-value
	a month				
Education	9-15	0.747	(0.723, 0.771)	0.012	9.01e-04
	16+	0.780	(0.749, 0.811)	0.016	
	Up to 8	0.676	(0.632, 0.721)	0.023	
Immigration status	Born in this country	0.736	(0.717, 0.755)	0.010	8.63e-03
	Born in another country	0.456	(0.240, 0.672)	0.107	
Religious affiliation	No religion/Atheist/Agnostic	0.743	(0.694, 0.793)	0.025	3.17e-01
	Christianity	0.726	(0.703, 0.748)	0.011	
	Combined	0.744	(0.605, 0.884)	0.070	
	Spiritism	0.769	(0.700, 0.839)	0.035	
	Umbanda, Candomblé, and other African-derived religions	0.799	(0.724, 0.874)	0.038	
	Buddhism	*	*	*	
	Islam	*	*	*	
	Chinese folk/traditional religion	*	*	*	
	Hinduism	*	*	*	
	Judaism	*	*	*	
	Primal, Animist, or Folk religion	*	*	*	

Table S3d. Brazil: Childhood predictors regression analysis

Variable	Category	Risk-Ratio	logRR SE	RR 95% CI	Global p-value
Relationship with your mother growing up	(Ref: Very bad/somewhat bad)				0.596
	Very good/somewhat good	0.995	0.038	(0.92,1.07)	
Relationship with your father growing up	(Ref: Very bad/somewhat bad)				0.561
	Very good/somewhat good	1.009	0.028	(0.95,1.07)	
Parents married to each other when you were around 12 years old	(Ref: Parents married)				0.295
	One or both of them had died	1.034	0.057	(0.92,1.16)	
	Parents were divorced	0.973	0.036	(0.91,1.04)	
	Parents were never married	1.012	0.031	(0.95,1.07)	
	Unsure	0.875	0.072	(0.76,1.01)	
Feelings about familys household income when growing up	(Ref: Got by)				0.013
	Found it difficult	1.017	0.028	(0.96,1.07)	
	Found it very difficult	1.041	0.038	(0.97,1.12)	
	Lived comfortably	1.069	0.023	(1.02,1.12)	
Physically or sexually abused when growing up	(Ref: No)				0.470
		1.017	0.025	(0.97,1.07)	
Felt like an outsider in your family when growing up	(Ref: No)				0.554
	Yes	1.010	0.029	(0.95,1.07)	
Your health when growing up	(Ref: Good)				0.778
	Excellent	1.012	0.025	(0.96,1.06)	
	Fair	0.989	0.035	(0.92,1.06)	
	Poor	0.961	0.084	(0.82,1.13)	
	Very good	0.992	0.028	(0.94,1.05)	
Born in This	(Ref: Born in this				0.005

Variable	Category	Risk-Ratio	logRR SE	RR 95% CI	Global p-value
country	country)				
	Born in another country	0.741	0.106	(0.60,0.91)	
How Often You Attended Religious Services or Worshiped When You Were Around 12 Years Old	(Ref: Never)				0.108
	At least once a week	1.068	0.033	(1.00,1.14)	
	Less than once a month	1.034	0.038	(0.96,1.11)	
	One to three times a month	1.072	0.039	(0.99,1.16)	
Year of birth (age group)	(Ref: 1998-2005; current age: 18-24)				0.254
	1943 or earlier (current age: 80+ years)	1.023	0.079	(0.88,1.19)	
	1943-1953 (current age: 70-79 years)	0.972	0.060	(0.86,1.09)	
	1953-1963 (current age: 60-69 years)	0.981	0.042	(0.90,1.07)	
	1963-1973 (current age: 50-59 years)	0.978	0.036	(0.91,1.05)	
	1973-1983 (current age: 40-49 years)	0.953	0.034	(0.89,1.02)	
	1983-1993 (current age: 30-39 years)	0.939	0.033	(0.88,1.00)	
	1993-1998 (current age: 25-29 years)	0.926	0.040	(0.86,1.00)	
Religion when twelve years old	(Ref: No religion/Atheist/Agnostic)				0.166
	Christianity	0.940	0.040	(0.87,1.02)	
	Collapsed affiliations with prevalence<3%	0.917	0.057	(0.82,1.02)	
Race plurality (prominent race/ethnic group [0] or not [1])	(Ref: Plurality group)				0.418
	Non-plurality groups	0.983	0.020	(0.95,1.02)	

Table S3e. Brazil: Sensitivity to unmeasured confounding of childhood predictors

Variable	Category	E-value for Estimate	E-value for 95% CI
Relationship with your mother growing up	(Ref: Very bad/somewhat bad)		
	Very good/somewhat good	1.08	1.00
Relationship with your father growing up	(Ref: Very bad/somewhat bad)		
	Very good/somewhat good	1.11	1.00
Parents married to each other when you were around 12 years old	(Ref: Parents married)		
	One or both of them had died	1.22	1.00
	Parents were divorced	1.20	1.00
	Parents were never married	1.12	1.00
	Unsure	1.55	1.00
Feelings about familys household income when growing up	(Ref: Got by)		
	Found it difficult	1.15	1.00
	Found it very difficult	1.25	1.00
	Lived comfortably	1.34	1.17
Physically or sexually abused when growing up	(Ref: No)		
		1.15	1.00
Felt like an outsider in your family when growing up	(Ref: No)		
	Yes	1.11	1.00
Your health when growing up	(Ref: Good)		
	Excellent	1.12	1.00
	Fair	1.11	1.00
	Poor	1.25	1.00
	Very good	1.10	1.00
Born in This country	(Ref: Born in this country)		
	Born in another country	2.04	1.42
How Often You Attended Religious Services or Worshiped When You Were Around 12 Years Old	(Ref: Never)		
	At least once a week	1.34	1.03
	Less than once a month	1.22	1.00
	One to three times a month	1.35	1.00
Year of birth (age group)	(Ref: 1998-2005; current age: 18-24)		
	1943 or earlier (current age: 80+ years)	1.18	1.00
	1943-1953 (current age: 70-79 years)	1.20	1.00

Variable	Category	E-value for Estimate	E-value for 95% CI
	1953-1963 (current age: 60-69 years)	1.16	1.00
	1963-1973 (current age: 50-59 years)	1.17	1.00
	1973-1983 (current age: 40-49 years)	1.28	1.00
	1983-1993 (current age: 30-39 years)	1.33	1.00
	1993-1998 (current age: 25-29 years)	1.37	1.00
Religion when twelve years old	(Ref: No religion/Atheist/Agnostic)		
	Christianity	1.32	1.00
	Collapsed affiliations with prevalence<3%	1.41	1.00
Race plurality (prominent race/ethnic group [0] or not [1])	(Ref: Plurality group)		
	Non-plurality groups	1.15	1.00

Table S4a. China: Demographic descriptive statistics

**Characteristic**	**N = 4,544**[1]
**Age group**	
1943 or earlier (current age: 80+ years)	2 (<0.1%)
1943-1953 (current age: 70-79 years)	133 (2.9%)
1953-1963 (current age: 60-69 years)	865 (19%)
1963-1973 (current age: 50-59 years)	770 (17%)
1973-1983 (current age: 40-49 years)	917 (20%)
1983-1993 (current age: 30-39 years)	822 (18%)
1993-1998 (current age: 25-29 years)	549 (12%)
1998-2005 (current age: 18-24 years)	485 (11%)
(Missing)	0 (0%)
**Gender**	
1. Male	2,300 (51%)
2. Female	2,244 (49%)
3. Other	0 (0%)
(Missing)	0 (0%)
**Marital status**	
1. Single/Never been married	911 (20%)
2. Married	3,214 (71%)
3. Separated	32 (0.7%)
4. Divorced	107 (2.4%)
5. Widowed	145 (3.2%)
6. Domestic partner	132 (2.9%)
(Missing)	3 (<0.1%)
**Employment**	
1. Employed for an employer	1,728 (38%)
2. Self-employed	1,170 (26%)
3. Retired	833 (18%)
4. Student	180 (4.0%)
5. Homemaker	265 (5.8%)
6. Unemployed and looking for a job	147 (3.2%)
7. None of these/Other	222 (4.9%)
(Missing)	0 (0%)
**Religious service attendance**	
1. More than once a week	47 (1.0%)
2. Once a week	170 (3.7%)
3. One to three times a month	267 (5.9%)
4. A few times a year	528 (12%)
5. Never	3,529 (78%)
(Missing)	4 (<0.1%)
**Education**	
1. Up to 8	2,925 (64%)
2. 9-15	1,191 (26%)
3. 16+	428 (9.4%)
(Missing)	0 (0%)
**Immigration**	
1. Born in this country	4,531 (100%)
2. Born in another country	8 (0.2%)

Characteristic	N = 4,544[1]
(Missing)	4 (<0.1%)
**Religious affiliation**	
1. Christianity	126 (2.8%)
10. Taoism	110 (2.4%)
11. Confucianism	13 (0.3%)
12. Primal, Animist, or Folk religion	19 (0.4%)
13. Spiritism	0 (0%)
14. Umbanda, Candomblé, and other African-derived religions	0 (0%)
15. Chinese folk/traditional religion	124 (2.7%)
2. Islam	17 (0.4%)
3. Hinduism	0 (0%)
4. Buddhism	755 (17%)
5. Judaism	2 (<0.1%)
6. Sikhism	0 (0%)
7. Baha'i	0 (0%)
8. Jainism	0 (0%)
9. Shinto	5 (0.1%)
96. Some other religion	0 (0%)
97. No religion/Atheist/Agnostic	3,361 (74%)
(Missing)	13 (0.3%)
**RACE ETHNICITY**	
(Missing)	4,544 (100%)

[1]n (%)

Table S4b. China: Childhood descriptive statistics

**Characteristic**	**N = 4,544**[1]
**Age**	45 (15)
**Year of birth**	
1943 or earlier (current age: 80+ years)	2 (<0.1%)
1943-1953 (current age: 70-79 years)	133 (2.9%)
1953-1963 (current age: 60-69 years)	865 (19%)
1963-1973 (current age: 50-59 years)	770 (17%)
1973-1983 (current age: 40-49 years)	917 (20%)
1983-1993 (current age: 30-39 years)	822 (18%)
1993-1998 (current age: 25-29 years)	549 (12%)
1998-2005 (current age: 18-24 years)	485 (11%)
(Missing)	0 (0%)
**Gender**	
1. Male	2,300 (51%)
2. Female	2,244 (49%)
3. Other	0 (0%)
(Missing)	0 (0%)
**Parent marital status**	
1. Parents were married	4,236 (93%)
2. Parents were divorced	109 (2.4%)
3. Parents were never married	28 (0.6%)
4. One or both of them had died	121 (2.7%)
5. Unsure	49 (1.1%)
(Missing)	1 (<0.1%)
**Age 12 religious service attendance**	
1. At least once a week	79 (1.7%)
2. One to three times a month	288 (6.3%)
3. Less than once a month	560 (12%)
4. Never	3,615 (80%)
(Missing)	2 (<0.1%)
**Relationship with mother**	
1. Very good	1,884 (41%)
2. Somewhat good	2,116 (47%)
3. Somewhat bad	174 (3.8%)
4. Very bad	24 (0.5%)
97. (Does not apply)	347 (7.6%)
(Missing)	0 (0%)
**Relationship with father**	
1. Very good	1,502 (33%)
2. Somewhat good	2,131 (47%)
3. Somewhat bad	384 (8.4%)
4. Very bad	44 (1.0%)
97. (Does not apply)	483 (11%)
(Missing)	1 (<0.1%)
**Outsider growing up**	
1. Yes	545 (12%)
2. No	3,927 (86%)
(Missing)	71 (1.6%)

Characteristic	N = 4,544[1]
**Abuse**	
1. Yes	241 (5.3%)
2. No	4,301 (95%)
(Missing)	2 (<0.1%)
**Self-rated health growing up**	
1. Excellent	666 (15%)
2. Very good	1,748 (38%)
3. Good	1,501 (33%)
4. Fair	588 (13%)
5. Poor	41 (0.9%)
(Missing)	0 (0%)
**Immigration status**	
1. Born in this country	4,531 (100%)
2. Born in another country	8 (0.2%)
(Missing)	4 (<0.1%)
**Subjective financial status of family growing up**	
1. Lived comfortably	1,249 (27%)
2. Got by	2,460 (54%)
3. Found it difficult	668 (15%)
4. Found it very difficult	166 (3.6%)
(Missing)	1 (<0.1%)
**Religious affiliation**	
1. Christianity	101 (2.2%)
10. Taoism	93 (2.1%)
11. Confucianism	8 (0.2%)
12. Primal, Animist, or Folk religion	12 (0.3%)
13. Spiritism	0 (0%)
14. Umbanda, Candomblé, and other African-derived religions	0 (0%)
15. Chinese folk/traditional religion	127 (2.8%)
2. Islam	16 (0.3%)
3. Hinduism	0 (0%)
4. Buddhism	608 (13%)
5. Judaism	0 (0%)
6. Sikhism	0 (0%)
7. Baha'i	0 (0%)
8. Jainism	0 (0%)
9. Shinto	6 (0.1%)
96. Some other religion	1 (<0.1%)
97. No religion/Atheist/Agnostic	3,551 (78%)
(Missing)	20 (0.4%)
**Race/Ethnicity**	
(Missing)	4,544 (100%)

[1]Mean (SD); n (%)

Table S4c. China: Proportions by demographic category

Variable	Category	Proportion	95% CI	SE	p-value
Age group	1998-2005 (current age: 18-24 years)	0.870	(0.839, 0.902)	0.016	< 1e-16
	1943 or earlier (current age: 80+ years)	1.000	*	*	
	1943-1953 (current age: 70-79 years)	0.817	(0.732, 0.902)	0.043	
	1953-1963 (current age: 60-69 years)	0.830	(0.799, 0.860)	0.016	
	1963-1973 (current age: 50-59 years)	0.832	(0.802, 0.862)	0.015	
	1973-1983 (current age: 40-49 years)	0.812	(0.785, 0.839)	0.014	
	1983-1993 (current age: 30-39 years)	0.807	(0.778, 0.836)	0.015	
	1993-1998 (current age: 25-29 years)	0.820	(0.785, 0.855)	0.018	
Gender	Male	0.795	(0.775, 0.814)	0.010	6.38e-07
	Female	0.857	(0.842, 0.872)	0.008	
	Other	*	*	*	
Marital status	Single/Never been married	0.822	(0.797, 0.848)	0.013	2.19e-01
	Divorced	0.905	(0.841, 0.970)	0.032	
	Domestic partner	0.827	(0.758, 0.896)	0.035	
	Married	0.822	(0.807, 0.838)	0.008	
	Separated	0.876	(0.748, 1.000)	0.062	
	Widowed	0.836	(0.764, 0.908)	0.036	
Employment	Employed for an employer	0.813	(0.792, 0.834)	0.011	1.15e-03
	Homemaker	0.814	(0.769, 0.859)	0.023	
	None of these/Other	0.773	(0.723, 0.823)	0.025	
	Retired	0.843	(0.815, 0.871)	0.014	
	Self-employed	0.852	(0.828, 0.876)	0.012	
	Student	0.854	(0.804, 0.903)	0.025	
	Unemployed and looking for a job	0.721	(0.651, 0.790)	0.035	
Religious service attendance	Never	0.808	(0.793, 0.822)	0.007	1.90e-12
	A few times a year	0.860	(0.829, 0.891)	0.016	
	More than once a week	0.922	(0.840, 1.000)	0.041	
	Once a week	0.883	(0.818, 0.948)	0.033	
	One to three times a month	0.933	(0.902, 0.965)	0.016	
Education	9-15	0.835	(0.805, 0.864)	0.015	6.97e-03
	16+	0.865	(0.837, 0.892)	0.014	

Variable	Category	Proportion	95% CI	SE	p-value
	Up to 8	0.816	(0.801, 0.830)	0.007	
Immigration status	Born in this country	0.825	(0.813, 0.838)	0.006	6.32e-01
	Born in another country	0.880	(0.571, 1.000)	0.115	
Religious affiliation	No religion/Atheist/Agnostic	0.811	(0.796, 0.825)	0.008	8.03e-05
	Christianity	*	*	*	
	Combined	0.875	(0.841, 0.908)	0.017	
	Spiritism	*	*	*	
	Umbanda, Candomblé, and other African-derived religions	*	*	*	
	Buddhism	0.863	(0.835, 0.892)	0.014	
	Islam	*	*	*	
	Chinese folk/traditional religion	*	*	*	
	Hinduism	*	*	*	
	Judaism	*	*	*	
	Primal, Animist, or Folk religion	*	*	*	

Table S4d. China: Childhood predictors regression analysis

Variable	Category	Risk-Ratio	logRR SE	RR 95% CI	Global p-value
Relationship with your mother growing up	(Ref: Very bad/somewhat bad)				0.501
	Very good/somewhat good	1.014	0.024	(0.97,1.06)	
Relationship with your father growing up	(Ref: Very bad/somewhat bad)				0.476
	Very good/somewhat good	1.014	0.021	(0.97,1.06)	
Parents married to each other when you were around 12 years old	(Ref: Parents married)				0.153
	One or both of them had died	0.943	0.043	(0.87,1.03)	
	Parents were divorced	0.923	0.042	(0.85,1.00)	
	Parents were never married	0.970	0.082	(0.83,1.14)	
	Unsure	0.855	0.090	(0.72,1.02)	
Feelings about familys household income when growing up	(Ref: Got by)				0.059
	Found it difficult	0.993	0.019	(0.96,1.03)	
	Found it very difficult	0.982	0.034	(0.92,1.05)	
	Lived comfortably	1.037	0.015	(1.01,1.07)	
Physically or sexually abused when growing up	(Ref: No)				0.452
		1.020	0.027	(0.97,1.08)	
Felt like an outsider in your family when growing up	(Ref: No)				0.613
	Yes	1.000	0.019	(0.96,1.04)	
Your health when growing up	(Ref: Good)				0.512
	Excellent	0.978	0.022	(0.94,1.02)	
	Fair	1.020	0.021	(0.98,1.06)	
	Poor	0.941	0.077	(0.81,1.09)	
	Very good	1.018	0.016	(0.99,1.05)	
Born in This	(Ref: Born in this				0.602

Variable	Category	Risk-Ratio	logRR SE	RR 95% CI	Global p-value
country	country)				
	Born in another country	1.010	0.110	(0.81,1.25)	
How Often You Attended Religious Services or Worshiped When You Were Around 12 Years Old	(Ref: Never)				0.001
	At least once a week	1.098	0.040	(1.02,1.19)	
	Less than once a month	1.029	0.024	(0.98,1.08)	
	One to three times a month	1.113	0.030	(1.05,1.18)	
Year of birth (age group)	(Ref: 1998-2005; current age: 18-24)				0.005
	1943 or earlier (current age: 80+ years)	1.278	0.074	(1.11,1.48)	
	1943-1953 (current age: 70-79 years)	0.962	0.046	(0.88,1.05)	
	1953-1963 (current age: 60-69 years)	0.968	0.023	(0.92,1.01)	
	1963-1973 (current age: 50-59 years)	0.969	0.022	(0.93,1.01)	
	1973-1983 (current age: 40-49 years)	0.946	0.021	(0.91,0.99)	
	1983-1993 (current age: 30-39 years)	0.941	0.022	(0.90,0.98)	
	1993-1998 (current age: 25-29 years)	0.951	0.024	(0.91,1.00)	
Religion when twelve years old	(Ref:)				0.712
	Buddhism	1.005	0.026	(0.96,1.06)	
	Collapsed affiliations with prevalence<3%	1.002	0.028	(0.95,1.06)	

Table S4e. China: Sensitivity to unmeasured confounding of childhood predictors

Variable	Category	E-value for Estimate	E-value for 95% CI
Relationship with your mother growing up	(Ref: Very bad/somewhat bad)		
	Very good/somewhat good	1.13	1.00
Relationship with your father growing up	(Ref: Very bad/somewhat bad)		
	Very good/somewhat good	1.13	1.00
Parents married to each other when you were around 12 years old	(Ref: Parents married)		
	One or both of them had died	1.31	1.00
	Parents were divorced	1.38	1.00
	Parents were never married	1.21	1.00
	Unsure	1.62	1.00
Feelings about familys household income when growing up	(Ref: Got by)		
	Found it difficult	1.09	1.00
	Found it very difficult	1.16	1.00
	Lived comfortably	1.23	1.09
Physically or sexually abused when growing up	(Ref: No)		
		1.16	1.00
Felt like an outsider in your family when growing up	(Ref: No)		
	Yes	1.02	1.00
Your health when growing up	(Ref: Good)		
	Excellent	1.18	1.00
	Fair	1.16	1.00
	Poor	1.32	1.00
	Very good	1.15	1.00
Born in This country	(Ref: Born in this country)		
	Born in another country	1.11	1.00
How Often You Attended Religious Services or Worshiped When You Were Around 12 Years Old	(Ref: Never)		
	At least once a week	1.43	1.14
	Less than once a month	1.20	1.00
	One to three times a month	1.47	1.27
Year of birth (age group)	(Ref: 1998-2005; current age: 18-24)		
	1943 or earlier (current age: 80+ years)	1.87	1.45
	1943-1953 (current age: 70-79 years)	1.24	1.00

Variable	Category	E-value for Estimate	E-value for 95% CI
	1953-1963 (current age: 60-69 years)	1.22	1.00
	1963-1973 (current age: 50-59 years)	1.21	1.00
	1973-1983 (current age: 40-49 years)	1.30	1.13
	1983-1993 (current age: 30-39 years)	1.32	1.16
	1993-1998 (current age: 25-29 years)	1.28	1.06
Religion when twelve years old	(Ref:)		
	Buddhism	1.07	1.00
	Collapsed affiliations with prevalence<3%	1.05	1.00

Table S5a. Egypt: Demographic descriptive statistics

Characteristic	N = 3,386[1]
**Age group**	
1943 or earlier (current age: 80+ years)	3 (<0.1%)
1943-1953 (current age: 70-79 years)	30 (0.9%)
1953-1963 (current age: 60-69 years)	274 (8.1%)
1963-1973 (current age: 50-59 years)	410 (12%)
1973-1983 (current age: 40-49 years)	577 (17%)
1983-1993 (current age: 30-39 years)	836 (25%)
1993-1998 (current age: 25-29 years)	419 (12%)
1998-2005 (current age: 18-24 years)	836 (25%)
(Missing)	0 (0%)
**Gender**	
1. Male	1,745 (52%)
2. Female	1,641 (48%)
3. Other	0 (0%)
(Missing)	0 (<0.1%)
**Marital status**	
1. Single/Never been married	791 (23%)
2. Married	2,316 (68%)
3. Separated	29 (0.9%)
4. Divorced	71 (2.1%)
5. Widowed	174 (5.1%)
6. Domestic partner	0 (0%)
(Missing)	6 (0.2%)
**Employment**	
1. Employed for an employer	925 (27%)
2. Self-employed	618 (18%)
3. Retired	173 (5.1%)
4. Student	281 (8.3%)
5. Homemaker	1,205 (36%)
6. Unemployed and looking for a job	169 (5.0%)
7. None of these/Other	14 (0.4%)
(Missing)	0 (<0.1%)
**Religious service attendance**	
1. More than once a week	620 (18%)
2. Once a week	666 (20%)
3. One to three times a month	267 (7.9%)
4. A few times a year	364 (11%)
5. Never	1,459 (43%)
(Missing)	10 (0.3%)
**Education**	
1. Up to 8	1,886 (56%)
2. 9-15	1,077 (32%)
3. 16+	423 (12%)
(Missing)	1 (<0.1%)
**Immigration**	
1. Born in this country	3,375 (100%)
2. Born in another country	10 (0.3%)

Characteristic	N = 3,386[1]
(Missing)	1 (<0.1%)
**Religious affiliation**	
1. Christianity	81 (2.4%)
10. Taoism	0 (<0.1%)
11. Confucianism	0 (0%)
12. Primal, Animist, or Folk religion	0 (0%)
13. Spiritism	0 (0%)
14. Umbanda, Candomblé, and other African-derived religions	0 (0%)
15. Chinese folk/traditional religion	0 (0%)
2. Islam	3,303 (98%)
3. Hinduism	0 (0%)
4. Buddhism	0 (0%)
5. Judaism	0 (0%)
6. Sikhism	0 (0%)
7. Baha'i	0 (0%)
8. Jainism	0 (0%)
9. Shinto	0 (0%)
96. Some other religion	0 (0%)
97. No religion/Atheist/Agnostic	0 (0%)
(Missing)	1 (<0.1%)
**RACE ETHNICITY**	
(Missing)	78 (2.3%)
401. Egypt: Arab	3,282 (97%)
402. Egypt: Turkish	4 (0.1%)
403. Egypt: Greek	1 (<0.1%)
405. Egypt: Bedouin Arab	4 (0.1%)
407. Egypt: Nubian	17 (0.5%)

[1]n (%)

Table S5b. Egypt: Childhood descriptive statistics

**Characteristic**	**N = 3,386**[1]
**Age**	37 (14)
**Year of birth**	
1943 or earlier (current age: 80+ years)	3 (<0.1%)
1943-1953 (current age: 70-79 years)	30 (0.9%)
1953-1963 (current age: 60-69 years)	274 (8.1%)
1963-1973 (current age: 50-59 years)	410 (12%)
1973-1983 (current age: 40-49 years)	577 (17%)
1983-1993 (current age: 30-39 years)	836 (25%)
1993-1998 (current age: 25-29 years)	419 (12%)
1998-2005 (current age: 18-24 years)	836 (25%)
(Missing)	0 (0%)
**Gender**	
1. Male	1,745 (52%)
2. Female	1,641 (48%)
3. Other	0 (0%)
(Missing)	0 (<0.1%)
**Parent marital status**	
1. Parents were married	2,897 (86%)
2. Parents were divorced	107 (3.1%)
3. Parents were never married	4 (0.1%)
4. One or both of them had died	339 (10.0%)
5. Unsure	28 (0.8%)
(Missing)	12 (0.3%)
**Age 12 religious service attendance**	
1. At least once a week	1,649 (49%)
2. One to three times a month	435 (13%)
3. Less than once a month	446 (13%)
4. Never	817 (24%)
(Missing)	39 (1.2%)
**Relationship with mother**	
1. Very good	2,966 (88%)
2. Somewhat good	347 (10%)
3. Somewhat bad	14 (0.4%)
4. Very bad	6 (0.2%)
97. (Does not apply)	53 (1.6%)
(Missing)	0 (0%)
**Relationship with father**	
1. Very good	2,648 (78%)
2. Somewhat good	492 (15%)
3. Somewhat bad	53 (1.6%)
4. Very bad	25 (0.7%)
97. (Does not apply)	157 (4.6%)
(Missing)	12 (0.3%)
**Outsider growing up**	
1. Yes	182 (5.4%)
2. No	3,193 (94%)
(Missing)	11 (0.3%)

Characteristic	N = 3,386[1]
**Abuse**	
1. Yes	278 (8.2%)
2. No	3,092 (91%)
(Missing)	16 (0.5%)
**Self-rated health growing up**	
1. Excellent	1,885 (56%)
2. Very good	882 (26%)
3. Good	343 (10%)
4. Fair	199 (5.9%)
5. Poor	76 (2.3%)
(Missing)	1 (<0.1%)
**Immigration status**	
1. Born in this country	3,375 (100%)
2. Born in another country	10 (0.3%)
(Missing)	1 (<0.1%)
**Subjective financial status of family growing up**	
1. Lived comfortably	923 (27%)
2. Got by	1,650 (49%)
3. Found it difficult	630 (19%)
4. Found it very difficult	183 (5.4%)
(Missing)	0 (<0.1%)
**Religious affiliation**	
1. Christianity	84 (2.5%)
10. Taoism	0 (<0.1%)
11. Confucianism	0 (0%)
12. Primal, Animist, or Folk religion	0 (0%)
13. Spiritism	0 (0%)
14. Umbanda, Candomblé, and other African-derived religions	0 (0%)
15. Chinese folk/traditional religion	0 (0%)
2. Islam	3,301 (98%)
3. Hinduism	0 (0%)
4. Buddhism	0 (0%)
5. Judaism	0 (0%)
6. Sikhism	0 (0%)
7. Baha'i	0 (0%)
8. Jainism	0 (<0.1%)
9. Shinto	0 (0%)
96. Some other religion	0 (0%)
97. No religion/Atheist/Agnostic	0 (0%)
(Missing)	0 (0%)
**Race/Ethnicity**	
(Missing)	78 (2.3%)
401. Egypt: Arab	3,282 (97%)
402. Egypt: Turkish	4 (0.1%)
403. Egypt: Greek	1 (<0.1%)
405. Egypt: Bedouin Arab	4 (0.1%)
407. Egypt: Nubian	17 (0.5%)

Characteristic	N = 3,386[1]

[1]Mean (SD); n (%)

Table S5c. Egypt: Proportions by demographic category

Variable	Category	Proportion	95% CI	SE	p-value
Age group	1998-2005 (current age: 18-24 years)	0.811	(0.770, 0.851)	0.021	< 1e-16
	1943 or earlier (current age: 80+ years)	1.000	*	*	
	1943-1953 (current age: 70-79 years)	0.726	(0.476, 0.976)	0.120	
	1953-1963 (current age: 60-69 years)	0.661	(0.568, 0.754)	0.047	
	1963-1973 (current age: 50-59 years)	0.727	(0.673, 0.781)	0.028	
	1973-1983 (current age: 40-49 years)	0.707	(0.662, 0.753)	0.023	
	1983-1993 (current age: 30-39 years)	0.718	(0.679, 0.757)	0.020	
	1993-1998 (current age: 25-29 years)	0.767	(0.714, 0.820)	0.027	
Gender	Male	0.759	(0.730, 0.788)	0.015	5.22e-02
	Female	0.724	(0.697, 0.751)	0.014	
	Other	*	*	*	
Marital status	Single/Never been married	0.815	(0.773, 0.857)	0.021	1.39e-04
	Divorced	0.867	(0.764, 0.969)	0.051	
	Domestic partner	*	*	*	
	Married	0.716	(0.690, 0.741)	0.013	
	Separated	0.800	(0.602, 0.999)	0.096	
	Widowed	0.698	(0.600, 0.797)	0.050	
Employment	Employed for an employer	0.743	(0.704, 0.781)	0.020	5.41e-05
	Homemaker	0.697	(0.662, 0.732)	0.018	
	None of these/Other	0.846	(0.545, 1.000)	0.117	
	Retired	0.678	(0.577, 0.779)	0.051	
	Self-employed	0.797	(0.754, 0.839)	0.022	
	Student	0.847	(0.787, 0.906)	0.030	
	Unemployed and looking for a job	0.741	(0.655, 0.827)	0.044	
Religious service attendance	Never	0.721	(0.689, 0.752)	0.016	3.74e-01
	A few times a year	0.777	(0.723, 0.830)	0.027	
	More than once a week	0.739	(0.690, 0.787)	0.025	
	Once a week	0.761	(0.712, 0.809)	0.025	
	One to three times a month	0.771	(0.697, 0.845)	0.038	
Education	9-15	0.792	(0.764, 0.819)	0.014	4.80e-09
	16+	0.855	(0.806, 0.904)	0.025	

Variable	Category	Proportion	95% CI	SE	p-value
	Up to 8	0.688	(0.656, 0.721)	0.016	
Immigration status	Born in this country	0.742	(0.720, 0.764)	0.011	5.76e-01
	Born in another country	0.829	(0.449, 1.000)	0.154	
Religious affiliation	No religion/Atheist/Agnostic	*	*	*	3.94e-01
	Christianity	*	*	*	
	Combined	0.686	(0.550, 0.821)	0.068	
	Spiritism	*	*	*	
	Umbanda, Candomblé, and other African-derived religions	*	*	*	
	Buddhism	*	*	*	
	Islam	0.743	(0.722, 0.765)	0.011	
	Chinese folk/traditional religion	*	*	*	
	Hinduism	*	*	*	
	Judaism	*	*	*	
	Primal, Animist, or Folk religion	*	*	*	

Table S5d. Egypt: Childhood predictors regression analysis

Variable	Category	Risk-Ratio	logRR SE	RR 95% CI	Global p-value
Relationship with your mother growing up	(Ref: Very bad/somewhat bad)				0.535
	Very good/somewhat good	1.049	0.108	(0.85,1.30)	
Relationship with your father growing up	(Ref: Very bad/somewhat bad)				0.048
	Very good/somewhat good	0.907	0.048	(0.82,1.00)	
Parents married to each other when you were around 12 years old	(Ref: Parents married)				0.215
	One or both of them had died	1.029	0.037	(0.96,1.11)	
	Parents were divorced	0.915	0.066	(0.80,1.04)	
	Parents were never married	1.184	0.087	(1.00,1.41)	
	Unsure	1.050	0.100	(0.86,1.28)	
Feelings about familys household income when growing up	(Ref: Got by)				0.527
	Found it difficult	0.981	0.026	(0.93,1.03)	
	Found it very difficult	0.950	0.042	(0.88,1.03)	
	Lived comfortably	0.996	0.023	(0.95,1.04)	
Physically or sexually abused when growing up	(Ref: No)				0.249
		0.957	0.035	(0.89,1.02)	
Felt like an outsider in your family when growing up	(Ref: No)				0.596
	Yes	0.995	0.043	(0.91,1.08)	
Your health when growing up	(Ref: Good)				0.096
	Excellent	1.075	0.039	(0.99,1.16)	
	Fair	1.106	0.054	(0.99,1.23)	
	Poor	1.117	0.072	(0.97,1.28)	
	Very good	1.076	0.039	(1.00,1.16)	
Born in This	(Ref: Born in this				0.555

Variable	Category	Risk-Ratio	logRR SE	RR 95% CI	Global p-value
country	country)				
	Born in another country	1.059	0.162	(0.77,1.46)	
How Often You Attended Religious Services or Worshiped When You Were Around 12 Years Old	(Ref: Never)				0.012
	At least once a week	1.072	0.026	(1.02,1.13)	
	Less than once a month	1.047	0.037	(0.97,1.13)	
	One to three times a month	1.087	0.032	(1.02,1.16)	
Year of birth (age group)	(Ref: 1998-2005; current age: 18-24)				4.44e-11
	1943 or earlier (current age: 80+ years)	1.251	0.032	(1.17,1.33)	
	1943-1953 (current age: 70-79 years)	0.930	0.127	(0.72,1.19)	
	1953-1963 (current age: 60-69 years)	0.868	0.050	(0.79,0.96)	
	1963-1973 (current age: 50-59 years)	0.929	0.034	(0.87,0.99)	
	1973-1983 (current age: 40-49 years)	0.907	0.029	(0.86,0.96)	
	1983-1993 (current age: 30-39 years)	0.914	0.029	(0.86,0.97)	
	1993-1998 (current age: 25-29 years)	0.953	0.034	(0.89,1.02)	
Religion when twelve years old	(Ref: Islam)				0.350
	Collapsed affiliations with prevalence<3%	0.934	0.067	(0.82,1.06)	
Race plurality (prominent race/ethnic group [0] or not [1])	(Ref: Plurality group)				0.494
	Non-plurality groups	0.965	0.059	(0.86,1.08)	

Table S5e. Egypt: Sensitivity to unmeasured confounding of childhood predictors

Variable	Category	E-value for Estimate	E-value for 95% CI
Relationship with your mother growing up	(Ref: Very bad/somewhat bad)		
	Very good/somewhat good	1.28	1.00
Relationship with your father growing up	(Ref: Very bad/somewhat bad)		
	Very good/somewhat good	1.44	1.06
Parents married to each other when you were around 12 years old	(Ref: Parents married)		
	One or both of them had died	1.20	1.00
	Parents were divorced	1.41	1.00
	Parents were never married	1.65	1.00
	Unsure	1.28	1.00
Feelings about familys household income when growing up	(Ref: Got by)		
	Found it difficult	1.16	1.00
	Found it very difficult	1.29	1.00
	Lived comfortably	1.07	1.00
Physically or sexually abused when growing up	(Ref: No)		
		1.26	1.00
Felt like an outsider in your family when growing up	(Ref: No)		
	Yes	1.08	1.00
Your health when growing up	(Ref: Good)		
	Excellent	1.36	1.00
	Fair	1.45	1.00
	Poor	1.48	1.00
	Very good	1.36	1.00
Born in This country	(Ref: Born in this country)		
	Born in another country	1.31	1.00
How Often You Attended Religious Services or Worshiped When You Were Around 12 Years Old	(Ref: Never)		
	At least once a week	1.35	1.16
	Less than once a month	1.27	1.00
	One to three times a month	1.39	1.17
Year of birth (age group)	(Ref: 1998-2005; current age: 18-24)		
	1943 or earlier (current age: 80+ years)	1.81	1.63
	1943-1953 (current age: 70-79 years)	1.36	1.00

Variable	Category	E-value for Estimate	E-value for 95% CI
	1953-1963 (current age: 60-69 years)	1.57	1.26
	1963-1973 (current age: 50-59 years)	1.36	1.09
	1973-1983 (current age: 40-49 years)	1.44	1.25
	1983-1993 (current age: 30-39 years)	1.41	1.22
	1993-1998 (current age: 25-29 years)	1.28	1.00
Religion when twelve years old	(Ref: Islam)		
	Collapsed affiliations with prevalence<3%	1.34	1.00
Race plurality (prominent race/ethnic group [0] or not [1])	(Ref: Plurality group)		
	Non-plurality groups	1.23	1.00

Table S6a. Germany: Demographic descriptive statistics

**Characteristic**	**N = 4,163**[1]
**Age group**	
1943 or earlier (current age: 80+ years)	65 (1.6%)
1943-1953 (current age: 70-79 years)	509 (12%)
1953-1963 (current age: 60-69 years)	826 (20%)
1963-1973 (current age: 50-59 years)	762 (18%)
1973-1983 (current age: 40-49 years)	678 (16%)
1983-1993 (current age: 30-39 years)	582 (14%)
1993-1998 (current age: 25-29 years)	339 (8.1%)
1998-2005 (current age: 18-24 years)	402 (9.7%)
(Missing)	0 (0%)
**Gender**	
1. Male	2,020 (49%)
2. Female	2,134 (51%)
3. Other	5 (0.1%)
(Missing)	5 (0.1%)
**Marital status**	
1. Single/Never been married	1,222 (29%)
2. Married	2,123 (51%)
3. Separated	68 (1.6%)
4. Divorced	354 (8.5%)
5. Widowed	154 (3.7%)
6. Domestic partner	225 (5.4%)
(Missing)	16 (0.4%)
**Employment**	
1. Employed for an employer	2,269 (54%)
2. Self-employed	272 (6.5%)
3. Retired	987 (24%)
4. Student	297 (7.1%)
5. Homemaker	125 (3.0%)
6. Unemployed and looking for a job	117 (2.8%)
7. None of these/Other	90 (2.2%)
(Missing)	6 (0.1%)
**Religious service attendance**	
1. More than once a week	79 (1.9%)
2. Once a week	190 (4.6%)
3. One to three times a month	228 (5.5%)
4. A few times a year	1,068 (26%)
5. Never	2,588 (62%)
(Missing)	9 (0.2%)
**Education**	
1. Up to 8	61 (1.5%)
2. 9-15	2,790 (67%)
3. 16+	1,309 (31%)
(Missing)	3 (<0.1%)
**Immigration**	
1. Born in this country	3,864 (93%)
2. Born in another country	272 (6.5%)

Characteristic	N = 4,163[1]
(Missing)	27 (0.6%)
**Religious affiliation**	
1. Christianity	2,338 (56%)
10. Taoism	0 (<0.1%)
11. Confucianism	2 (<0.1%)
12. Primal, Animist, or Folk religion	14 (0.3%)
13. Spiritism	0 (0%)
14. Umbanda, Candomblé, and other African-derived religions	0 (0%)
15. Chinese folk/traditional religion	0 (0%)
2. Islam	117 (2.8%)
3. Hinduism	9 (0.2%)
4. Buddhism	23 (0.5%)
5. Judaism	7 (0.2%)
6. Sikhism	4 (0.1%)
7. Baha'i	2 (<0.1%)
8. Jainism	0 (0%)
9. Shinto	3 (<0.1%)
96. Some other religion	25 (0.6%)
97. No religion/Atheist/Agnostic	1,580 (38%)
(Missing)	39 (0.9%)

[1] n (%)

Table S6a. Germany: Childhood descriptive statistics

**Characteristic**	**N = 4,163**[1]
**Age**	49 (17)
**Year of birth**	
1943 or earlier (current age: 80+ years)	65 (1.6%)
1943-1953 (current age: 70-79 years)	509 (12%)
1953-1963 (current age: 60-69 years)	826 (20%)
1963-1973 (current age: 50-59 years)	762 (18%)
1973-1983 (current age: 40-49 years)	678 (16%)
1983-1993 (current age: 30-39 years)	582 (14%)
1993-1998 (current age: 25-29 years)	339 (8.1%)
1998-2005 (current age: 18-24 years)	402 (9.7%)
(Missing)	0 (0%)
**Gender**	
1. Male	2,020 (49%)
2. Female	2,134 (51%)
3. Other	5 (0.1%)
(Missing)	5 (0.1%)
**Parent marital status**	
1. Parents were married	3,412 (82%)
2. Parents were divorced	362 (8.7%)
3. Parents were never married	249 (6.0%)
4. One or both of them had died	95 (2.3%)
5. Unsure	38 (0.9%)
(Missing)	7 (0.2%)
**Age 12 religious service attendance**	
1. At least once a week	847 (20%)
2. One to three times a month	849 (20%)
3. Less than once a month	1,268 (30%)
4. Never	1,185 (28%)
(Missing)	14 (0.3%)
**Relationship with mother**	
1. Very good	2,191 (53%)
2. Somewhat good	1,447 (35%)
3. Somewhat bad	291 (7.0%)
4. Very bad	83 (2.0%)
97. (Does not apply)	137 (3.3%)
(Missing)	14 (0.3%)
**Relationship with father**	
1. Very good	1,916 (46%)
2. Somewhat good	1,389 (33%)
3. Somewhat bad	364 (8.7%)
4. Very bad	175 (4.2%)
97. (Does not apply)	301 (7.2%)
(Missing)	18 (0.4%)
**Outsider growing up**	
1. Yes	483 (12%)
2. No	3,607 (87%)
(Missing)	72 (1.7%)

Characteristic	N = 4,163[1]
**Abuse**	
1. Yes	479 (12%)
2. No	3,666 (88%)
(Missing)	18 (0.4%)
**Self-rated health growing up**	
1. Excellent	1,123 (27%)
2. Very good	1,663 (40%)
3. Good	1,060 (25%)
4. Fair	262 (6.3%)
5. Poor	42 (1.0%)
(Missing)	13 (0.3%)
**Immigration status**	
1. Born in this country	3,864 (93%)
2. Born in another country	272 (6.5%)
(Missing)	27 (0.6%)
**Subjective financial status of family growing up**	
1. Lived comfortably	1,333 (32%)
2. Got by	2,000 (48%)
3. Found it difficult	664 (16%)
4. Found it very difficult	152 (3.6%)
(Missing)	15 (0.4%)
**Religious affiliation**	
1. Christianity	2,835 (68%)
10. Taoism	0 (0%)
11. Confucianism	6 (0.1%)
12. Primal, Animist, or Folk religion	15 (0.4%)
13. Spiritism	0 (0%)
14. Umbanda, Candomblé, and other African-derived religions	0 (0%)
15. Chinese folk/traditional religion	0 (0%)
2. Islam	109 (2.6%)
3. Hinduism	9 (0.2%)
4. Buddhism	5 (0.1%)
5. Judaism	3 (<0.1%)
6. Sikhism	6 (0.1%)
7. Baha'i	1 (<0.1%)
8. Jainism	0 (0%)
9. Shinto	0 (0%)
96. Some other religion	36 (0.9%)
97. No religion/Atheist/Agnostic	1,110 (27%)
(Missing)	29 (0.7%)

[1]Mean (SD); n (%)

Table S6c. Germany: Proportions by demographic category

Variable	Category	Proportion	95% CI	SE	p-value
Age group	1998-2005 (current age: 18-24 years)	0.883	(0.837, 0.929)	0.023	5.54e-02
	1943 or earlier (current age: 80+ years)	0.811	(0.669, 0.953)	0.071	
	1943-1953 (current age: 70-79 years)	0.841	(0.804, 0.878)	0.019	
	1953-1963 (current age: 60-69 years)	0.835	(0.807, 0.864)	0.015	
	1963-1973 (current age: 50-59 years)	0.803	(0.769, 0.838)	0.018	
	1973-1983 (current age: 40-49 years)	0.824	(0.785, 0.862)	0.020	
	1983-1993 (current age: 30-39 years)	0.830	(0.792, 0.869)	0.020	
	1993-1998 (current age: 25-29 years)	0.890	(0.846, 0.935)	0.023	
Gender	Male	0.822	(0.802, 0.843)	0.010	1.40e-01
	Female	0.850	(0.830, 0.869)	0.010	
	Other	0.701	*	*	
Marital status	Single/Never been married	0.848	(0.821, 0.874)	0.013	8.70e-03
	Divorced	0.820	(0.779, 0.861)	0.021	
	Domestic partner	0.917	(0.872, 0.962)	0.023	
	Married	0.827	(0.806, 0.848)	0.011	
	Separated	0.820	(0.703, 0.937)	0.059	
	Widowed	0.804	(0.729, 0.878)	0.038	
Employment	Employed for an employer	0.834	(0.814, 0.854)	0.010	1.20e-02
	Homemaker	0.840	(0.771, 0.909)	0.035	
	None of these/Other	0.803	(0.711, 0.896)	0.046	
	Retired	0.821	(0.793, 0.848)	0.014	
	Self-employed	0.886	(0.832, 0.940)	0.027	
	Student	0.902	(0.851, 0.953)	0.026	
	Unemployed and looking for a job	0.750	(0.672, 0.829)	0.040	
Religious service attendance	Never	0.812	(0.793, 0.830)	0.009	3.43e-09
	A few times a year	0.883	(0.859, 0.907)	0.012	
	More than once a week	0.959	(0.912, 1.000)	0.023	
	Once a week	0.850	(0.774, 0.925)	0.038	
	One to three times	0.843	(0.776, 0.910)	0.034	

Variable	Category	Proportion	95% CI	SE	p-value
	a month				
Education	9-15	0.814	(0.795, 0.832)	0.009	4.54e-08
	16+	0.891	(0.869, 0.912)	0.011	
	Up to 8	0.709	(0.584, 0.833)	0.062	
Immigration status	Born in this country	0.836	(0.821, 0.850)	0.007	7.82e-01
	Born in another country	0.844	(0.786, 0.902)	0.029	
Religious affiliation	No religion/Atheist/Agnostic	0.839	(0.817, 0.862)	0.012	9.32e-01
	Christianity	0.834	(0.816, 0.852)	0.009	
	Combined	0.838	(0.761, 0.916)	0.039	
	Spiritism	*	*	*	
	Umbanda, Candomblé, and other African-derived religions	*	*	*	
	Buddhism	*	*	*	
	Islam	*	*	*	
	Chinese folk/traditional religion	*	*	*	
	Hinduism	*	*	*	
	Judaism	*	*	*	
	Primal, Animist, or Folk religion	*	*	*	

Table S6d. Germany: Childhood predictors regression analysis

Variable	Category	Risk-Ratio	logRR SE	RR 95% CI	Global p-value
Relationship with your mother growing up	(Ref: Very bad/somewhat bad)				0.594
	Very good/somewhat good	0.997	0.025	(0.95,1.05)	
Relationship with your father growing up	(Ref: Very bad/somewhat bad)				0.399
	Very good/somewhat good	1.021	0.023	(0.98,1.07)	
Parents married to each other when you were around 12 years old	(Ref: Parents married)				0.021
	One or both of them had died	1.104	0.035	(1.03,1.18)	
	Parents were divorced	0.997	0.029	(0.94,1.06)	
	Parents were never married	1.003	0.031	(0.94,1.07)	
	Unsure	1.016	0.079	(0.87,1.19)	
Feelings about familys household income when growing up	(Ref: Got by)				0.031
	Found it difficult	0.993	0.022	(0.95,1.04)	
	Found it very difficult	0.988	0.042	(0.91,1.07)	
	Lived comfortably	1.044	0.017	(1.01,1.08)	
Physically or sexually abused when growing up	(Ref: No)				0.466
		1.017	0.024	(0.97,1.07)	
Felt like an outsider in your family when growing up	(Ref: No)				0.519
	Yes	0.988	0.023	(0.95,1.03)	
Your health when growing up	(Ref: Good)				0.016
	Excellent	1.058	0.021	(1.02,1.10)	
	Fair	1.023	0.030	(0.96,1.08)	

Variable	Category	Risk-Ratio	logRR SE	RR 95% CI	Global p-value
	Poor	0.973	0.073	(0.84,1.12)	
	Very good	1.054	0.020	(1.01,1.10)	
Born in This country	(Ref: Born in this country)				0.505
	Born in another country	1.017	0.031	(0.96,1.08)	
How Often You Attended Religious Services or Worshiped When You Were Around 12 Years Old	(Ref: Never)				2.04e-04
	At least once a week	1.092	0.022	(1.05,1.14)	
	Less than once a month	1.070	0.021	(1.03,1.11)	
	One to three times a month	1.049	0.025	(1.00,1.10)	
Year of birth (age group)	(Ref: 1998-2005; current age: 18-24)				0.233
	1943 or earlier (current age: 80+ years)	0.935	0.076	(0.81,1.08)	
	1943-1953 (current age: 70-79 years)	0.969	0.032	(0.91,1.03)	
	1953-1963 (current age: 60-69 years)	0.967	0.029	(0.91,1.02)	
	1963-1973 (current age: 50-59 years)	0.939	0.031	(0.88,1.00)	
	1973-1983 (current age: 40-49 years)	0.954	0.031	(0.90,1.01)	
	1983-1993 (current age: 30-39 years)	0.956	0.031	(0.90,1.01)	
	1993-1998 (current age: 25-29 years)	1.009	0.033	(0.95,1.08)	
Religion when twelve years old	(Ref: No religion/Atheist/Agnostic)				0.318
	Christianity	0.974	0.020	(0.94,1.01)	
	Collapsed affiliations with prevalence<3%	0.949	0.048	(0.86,1.04)	

Table S6e. Germany: Sensitivity to unmeasured confounding of childhood predictors

Variable	Category	E-value for Estimate	E-value for 95% CI
Relationship with your mother growing up	(Ref: Very bad/somewhat bad)		
	Very good/somewhat good	1.06	1.00
Relationship with your father growing up	(Ref: Very bad/somewhat bad)		
	Very good/somewhat good	1.17	1.00
Parents married to each other when you were around 12 years old	(Ref: Parents married)		
	One or both of them had died	1.44	1.21
	Parents were divorced	1.05	1.00
	Parents were never married	1.06	1.00
	Unsure	1.14	1.00
Feelings about familys household income when growing up	(Ref: Got by)		
	Found it difficult	1.09	1.00
	Found it very difficult	1.12	1.00
	Lived comfortably	1.26	1.11
Physically or sexually abused when growing up	(Ref: No)		
		1.15	1.00
Felt like an outsider in your family when growing up	(Ref: No)		
	Yes	1.12	1.00
Your health when growing up	(Ref: Good)		
	Excellent	1.31	1.14
	Fair	1.18	1.00
	Poor	1.20	1.00
	Very good	1.29	1.13
Born in This country	(Ref: Born in this country)		
	Born in another country	1.15	1.00
How Often You Attended Religious Services or Worshiped When You Were Around 12 Years Old	(Ref: Never)		
	At least once a week	1.41	1.26
	Less than once a month	1.34	1.20
	One to three times a month	1.28	1.00
Year of birth (age group)	(Ref: 1998-2005; current age: 18-24)		
	1943 or earlier (current age: 80+ years)	1.34	1.00
	1943-1953 (current age: 70-79 years)	1.21	1.00

Variable	Category	E-value for Estimate	E-value for 95% CI
	1953-1963 (current age: 60-69 years)	1.22	1.00
	1963-1973 (current age: 50-59 years)	1.33	1.06
	1973-1983 (current age: 40-49 years)	1.27	1.00
	1983-1993 (current age: 30-39 years)	1.27	1.00
	1993-1998 (current age: 25-29 years)	1.10	1.00
Religion when twelve years old	(Ref: No religion/Atheist/Agnostic)		
	Christianity	1.19	1.00
	Collapsed affiliations with prevalence<3%	1.29	1.00

Table S7a. Hong Kong: Demographic descriptive statistics

**Characteristic**	**N = 706**[1]
**Age group**	
1943 or earlier (current age: 80+ years)	0 (0%)
1943-1953 (current age: 70-79 years)	48 (6.8%)
1953-1963 (current age: 60-69 years)	135 (19%)
1963-1973 (current age: 50-59 years)	141 (20%)
1973-1983 (current age: 40-49 years)	156 (22%)
1983-1993 (current age: 30-39 years)	150 (21%)
1993-1998 (current age: 25-29 years)	33 (4.6%)
1998-2005 (current age: 18-24 years)	44 (6.2%)
(Missing)	0 (0%)
**Gender**	
1. Male	311 (44%)
2. Female	395 (56%)
3. Other	0 (0%)
(Missing)	0 (0%)
**Marital status**	
1. Single/Never been married	234 (33%)
2. Married	424 (60%)
3. Separated	3 (0.4%)
4. Divorced	21 (3.0%)
5. Widowed	16 (2.3%)
6. Domestic partner	8 (1.2%)
(Missing)	0 (0%)
**Employment**	
1. Employed for an employer	499 (71%)
2. Self-employed	24 (3.4%)
3. Retired	95 (13%)
4. Student	10 (1.4%)
5. Homemaker	42 (5.9%)
6. Unemployed and looking for a job	23 (3.3%)
7. None of these/Other	10 (1.5%)
(Missing)	3 (0.4%)
**Religious service attendance**	
1. More than once a week	12 (1.8%)
2. Once a week	56 (8.0%)
3. One to three times a month	60 (8.5%)
4. A few times a year	147 (21%)
5. Never	430 (61%)
(Missing)	0 (0%)
**Education**	
1. Up to 8	49 (6.9%)
2. 9-15	505 (72%)
3. 16+	152 (22%)
(Missing)	0 (0%)

Characteristic	N = 706[1]
**Immigration**	
1. Born in this country	624 (88%)
2. Born in another country	62 (8.8%)
(Missing)	20 (2.8%)
**Religious affiliation**	
1. Christianity	144 (20%)
10. Taoism	25 (3.5%)
11. Confucianism	1 (<0.1%)
12. Primal, Animist, or Folk religion	5 (0.7%)
13. Spiritism	0 (0%)
14. Umbanda, Candomblé, and other African-derived religions	0 (0%)
15. Chinese folk/traditional religion	30 (4.2%)
2. Islam	3 (0.5%)
3. Hinduism	1 (0.2%)
4. Buddhism	54 (7.7%)
5. Judaism	0 (0%)
6. Sikhism	0 (0%)
7. Baha'i	0 (0%)
8. Jainism	0 (0%)
9. Shinto	1 (<0.1%)
96. Some other religion	0 (0%)
97. No religion/Atheist/Agnostic	440 (62%)
(Missing)	2 (0.3%)
**RACE ETHNICITY**	
2401. Hong Kong: Chinese (Cantonese)	494 (70%)
2402. Hong Kong: Chinese (Chaoshan)	47 (6.7%)
2403. Hong Kong: Chinese (Fujianese)	15 (2.2%)
2404. Hong Kong: Chinese (Hakka)	22 (3.0%)
2405. Hong Kong: Chinese (Shanghainese)	24 (3.4%)
2406. Hong Kong: Chinese (Other ethnicity)	56 (8.0%)
2407. Hong Kong: East Asian (Korean, Japanese)	1 (0.1%)
2408. Hong Kong: Southeast Asian (Filipino, Indonesian, Thailand)	1 (<0.1%)
2409. Hong Kong: South Asian (Indian, Nepalese, Pakistani)	3 (0.4%)
2410. Hong Kong: Taiwanese	1 (0.1%)
2411. Hong Kong: White	2 (0.4%)
9995. Prefer not to answer	36 (5.1%)
9996. Other	3 (0.4%)

[1]n (%)

Table S7b. Hong Kong: Childhood descriptive statistics

**Characteristic**	**N = 706**[1]
**Age**	48 (15)
**Year of birth**	
1943 or earlier (current age: 80+ years)	0 (0%)
1943-1953 (current age: 70-79 years)	48 (6.8%)
1953-1963 (current age: 60-69 years)	135 (19%)
1963-1973 (current age: 50-59 years)	141 (20%)
1973-1983 (current age: 40-49 years)	156 (22%)
1983-1993 (current age: 30-39 years)	150 (21%)
1993-1998 (current age: 25-29 years)	33 (4.6%)
1998-2005 (current age: 18-24 years)	44 (6.2%)
(Missing)	0 (0%)
**Gender**	
1. Male	311 (44%)
2. Female	395 (56%)
3. Other	0 (0%)
(Missing)	0 (0%)
**Parent marital status**	
1. Parents were married	644 (91%)
2. Parents were divorced	20 (2.9%)
3. Parents were never married	13 (1.9%)
4. One or both of them had died	18 (2.6%)
5. Unsure	10 (1.4%)
(Missing)	0 (0%)
**Age 12 religious service attendance**	
1. At least once a week	58 (8.2%)
2. One to three times a month	51 (7.2%)
3. Less than once a month	206 (29%)
4. Never	392 (55%)
(Missing)	0 (0%)
**Relationship with mother**	
1. Very good	208 (29%)
2. Somewhat good	282 (40%)
3. Somewhat bad	101 (14%)
4. Very bad	23 (3.3%)
97. (Does not apply)	91 (13%)
(Missing)	0 (<0.1%)
**Relationship with father**	
1. Very good	155 (22%)
2. Somewhat good	271 (38%)
3. Somewhat bad	110 (16%)
4. Very bad	43 (6.0%)
97. (Does not apply)	127 (18%)
(Missing)	0 (0%)
**Outsider growing up**	
1. Yes	102 (14%)
2. No	590 (84%)
(Missing)	14 (2.0%)

Characteristic	N = 706[1]
**Abuse**	
1. Yes	58 (8.3%)
2. No	648 (92%)
(Missing)	0 (0%)
**Self-rated health growing up**	
1. Excellent	49 (6.9%)
2. Very good	212 (30%)
3. Good	263 (37%)
4. Fair	150 (21%)
5. Poor	30 (4.3%)
(Missing)	1 (0.2%)
**Immigration status**	
1. Born in this country	624 (88%)
2. Born in another country	62 (8.8%)
(Missing)	20 (2.8%)
**Subjective financial status of family growing up**	
1. Lived comfortably	91 (13%)
2. Got by	400 (57%)
3. Found it difficult	180 (25%)
4. Found it very difficult	32 (4.5%)
(Missing)	3 (0.4%)
**Religious affiliation**	
1. Christianity	125 (18%)
10. Taoism	20 (2.8%)
11. Confucianism	0 (0%)
12. Primal, Animist, or Folk religion	3 (0.4%)
13. Spiritism	0 (0%)
14. Umbanda, Candomblé, and other African-derived religions	0 (0%)
15. Chinese folk/traditional religion	26 (3.7%)
2. Islam	3 (0.5%)
3. Hinduism	1 (0.2%)
4. Buddhism	51 (7.2%)
5. Judaism	1 (<0.1%)
6. Sikhism	0 (0%)
7. Baha'i	0 (0%)
8. Jainism	0 (<0.1%)
9. Shinto	0 (0%)
96. Some other religion	4 (0.6%)
97. No religion/Atheist/Agnostic	471 (67%)
(Missing)	0 (0%)
**Race/Ethnicity**	
2401. Hong Kong: Chinese (Cantonese)	494 (70%)
2402. Hong Kong: Chinese (Chaoshan)	47 (6.7%)
2403. Hong Kong: Chinese (Fujianese)	15 (2.2%)
2404. Hong Kong: Chinese (Hakka)	22 (3.0%)
2405. Hong Kong: Chinese (Shanghainese)	24 (3.4%)
2406. Hong Kong: Chinese (Other ethnicity)	56 (8.0%)

Characteristic	N = 706[1]
2407. Hong Kong: East Asian (Korean, Japanese)	1 (0.1%)
2408. Hong Kong: Southeast Asian (Filipino, Indonesian, Thailand)	1 (<0.1%)
2409. Hong Kong: South Asian (Indian, Nepalese, Pakistani)	3 (0.4%)
2410. Hong Kong: Taiwanese	1 (0.1%)
2411. Hong Kong: White	2 (0.4%)
9995. Prefer not to answer	36 (5.1%)
9996. Other	3 (0.4%)

[1]Mean (SD); n (%)

Table S7c. Hong Kong: Proportions by demographic category

Variable	Category	Proportion	95% CI	SE	p-value
Age group	1998-2005 (current age: 18-24 years)	0.634	(0.374, 0.895)	0.128	7.68e-01
	1943 or earlier (current age: 80+ years)	*	*	*	
	1943-1953 (current age: 70-79 years)	0.561	(0.277, 0.845)	0.140	
	1953-1963 (current age: 60-69 years)	0.428	(0.276, 0.580)	0.077	
	1963-1973 (current age: 50-59 years)	0.475	(0.378, 0.573)	0.049	
	1973-1983 (current age: 40-49 years)	0.473	(0.396, 0.550)	0.039	
	1983-1993 (current age: 30-39 years)	0.425	(0.325, 0.525)	0.051	
	1993-1998 (current age: 25-29 years)	0.428	(0.196, 0.660)	0.112	
Gender	Male	0.492	(0.410, 0.574)	0.042	4.21e-01
	Female	0.450	(0.383, 0.516)	0.034	
	Other	*	*	*	
Marital status	Single/Never been married	0.451	(0.360, 0.543)	0.046	< 1e-16
	Divorced	0.284	(0.041, 0.527)	0.112	
	Domestic partner	0.514	(0.000, 1.000)	0.138	
	Married	0.487	(0.420, 0.553)	0.034	
	Separated	1.000	*	*	
	Widowed	0.364	(0.000, 0.809)	0.192	
Employment	Employed for an employer	0.472	(0.417, 0.527)	0.028	9.54e-03
	Homemaker	0.413	(0.141, 0.684)	0.132	
	None of these/Other	0.224	(0.000, 0.935)	0.144	
	Retired	0.484	(0.293, 0.675)	0.096	
	Self-employed	0.409	(0.121, 0.697)	0.135	
	Student	0.861	(0.000, 1.000)	0.106	
	Unemployed and looking for a job	0.444	(0.102, 0.786)	0.159	
Religious service attendance	Never	0.426	(0.360, 0.492)	0.034	5.70e-09
	A few times a year	0.459	(0.344, 0.574)	0.058	
	More than once a week	0.931	(0.756, 1.000)	0.069	
	Once a week	0.586	(0.429, 0.743)	0.078	
	One to three times	0.590	(0.386, 0.794)	0.101	

Variable	Category	Proportion	95% CI	SE	p-value
	a month				
Education	9-15	0.464	(0.402, 0.525)	0.031	6.79e-01
	16+	0.502	(0.415, 0.589)	0.044	
	Up to 8	0.414	(0.107, 0.722)	0.152	
Immigration status	Born in this country	0.450	(0.397, 0.504)	0.027	1.28e-02
	Born in another country	0.649	(0.469, 0.829)	0.089	
Religious affiliation	No religion/Atheist/Agnostic	0.425	(0.362, 0.488)	0.032	1.36e-01
	Christianity	0.542	(0.435, 0.650)	0.055	
	Combined	0.585	(0.336, 0.834)	0.121	
	Spiritism	*	*	*	
	Umbanda, Candomblé, and other African-derived religions	*	*	*	
	Buddhism	0.440	(0.228, 0.651)	0.105	
	Islam	*	*	*	
	Chinese folk/traditional religion	0.669	(0.329, 1.000)	0.161	
	Hinduism	*	*	*	
	Judaism	*	*	*	
	Primal, Animist, or Folk religion	*	*	*	

Table S7d. Hong Kong: Childhood predictors regression analysis

Variable	Category	Risk-Ratio	logRR SE	RR 95% CI	Global p-value
Relationship with your mother growing up	(Ref: Very bad/somewhat bad)				0.610
	Very good/somewhat good	1.002	0.071	(0.87,1.15)	
Relationship with your father growing up	(Ref: Very bad/somewhat bad)				0.423
	Very good/somewhat good	1.054	0.064	(0.93,1.19)	
Parents married to each other when you were around 12 years old	(Ref: Parents married)				0.715
	One or both of them had died	0.867	0.165	(0.63,1.20)	
	Parents were divorced	1.005	0.148	(0.75,1.34)	
	Parents were never married	1.117	0.137	(0.85,1.46)	
	Unsure	1.041	0.196	(0.71,1.53)	
Feelings about familys household income when growing up	(Ref: Got by)				0.674
	Found it difficult	1.002	0.063	(0.89,1.13)	
	Found it very difficult	1.139	0.138	(0.87,1.49)	
	Lived comfortably	1.024	0.075	(0.88,1.19)	
Physically or sexually abused when growing up	(Ref: No)				0.475
		1.064	0.093	(0.89,1.28)	
Felt like an outsider in your family when growing up	(Ref: No)				0.401
	Yes	0.941	0.069	(0.82,1.08)	
Your health when growing up	(Ref: Good)				0.286
	Excellent	1.132	0.105	(0.92,1.39)	
	Fair	0.933	0.072	(0.81,1.07)	
	Poor	0.955	0.141	(0.72,1.26)	
	Very good	1.118	0.062	(0.99,1.26)	
Born in This	(Ref: Born in this				0.017

Variable	Category	Risk-Ratio	logRR SE	RR 95% CI	Global p-value
country	country)				
	Born in another country	1.248	0.091	(1.04,1.49)	
How Often You Attended Religious Services or Worshiped When You Were Around 12 Years Old	(Ref: Never)				0.621
	At least once a week	1.106	0.111	(0.89,1.37)	
	Less than once a month	1.013	0.060	(0.90,1.14)	
	One to three times a month	1.076	0.097	(0.89,1.30)	
Year of birth (age group)	(Ref: 1998-2005; current age: 18-24)				0.225
	1943-1953 (current age: 70-79 years)	0.874	0.176	(0.62,1.23)	
	1953-1963 (current age: 60-69 years)	0.801	0.138	(0.61,1.05)	
	1963-1973 (current age: 50-59 years)	0.847	0.122	(0.67,1.08)	
	1973-1983 (current age: 40-49 years)	0.846	0.118	(0.67,1.07)	
	1983-1993 (current age: 30-39 years)	0.801	0.121	(0.63,1.02)	
	1993-1998 (current age: 25-29 years)	0.833	0.147	(0.62,1.11)	
Religion when twelve years old	(Ref: No religion/Atheist/Agnostic)				0.766
	Buddhism	1.055	0.127	(0.82,1.35)	
	Chinese folk/traditional religion	1.052	0.133	(0.81,1.37)	
	Christianity	1.002	0.078	(0.86,1.17)	
	Collapsed affiliations with prevalence<3%	1.111	0.145	(0.84,1.48)	

Table S7e. Hong Kong: Sensitivity to unmeasured confounding of childhood predictors

Variable	Category	E-value for Estimate	E-value for 95% CI
Relationship with your mother growing up	(Ref: Very bad/somewhat bad)		
	Very good/somewhat good	1.05	1.00
Relationship with your father growing up	(Ref: Very bad/somewhat bad)		
	Very good/somewhat good	1.29	1.00
Parents married to each other when you were around 12 years old	(Ref: Parents married)		
	One or both of them had died	1.57	1.00
	Parents were divorced	1.08	1.00
	Parents were never married	1.48	1.00
	Unsure	1.25	1.00
Feelings about familys household income when growing up	(Ref: Got by)		
	Found it difficult	1.04	1.00
	Found it very difficult	1.54	1.00
	Lived comfortably	1.18	1.00
Physically or sexually abused when growing up	(Ref: No)		
		1.32	1.00
Felt like an outsider in your family when growing up	(Ref: No)		
	Yes	1.32	1.00
Your health when growing up	(Ref: Good)		
	Excellent	1.52	1.00
	Fair	1.35	1.00
	Poor	1.27	1.00
	Very good	1.48	1.00
Born in This country	(Ref: Born in this country)		
	Born in another country	1.80	1.25
How Often You Attended Religious Services or Worshiped When You Were Around 12 Years Old	(Ref: Never)		
	At least once a week	1.45	1.00
	Less than once a month	1.13	1.00
	One to three times a month	1.36	1.00
Year of birth (age group)	(Ref: 1998-2005; current age: 18-24)		
	1943-1953 (current age: 70-79 years)	1.55	1.00
	1953-1963 (current age: 60-69 years)	1.81	1.00

Variable	Category	E-value for Estimate	E-value for 95% CI
	1963-1973 (current age: 50-59 years)	1.64	1.00
	1973-1983 (current age: 40-49 years)	1.65	1.00
	1983-1993 (current age: 30-39 years)	1.81	1.00
	1993-1998 (current age: 25-29 years)	1.69	1.00
Religion when twelve years old	(Ref: No religion/Atheist/Agnostic)		
	Buddhism	1.30	1.00
	Chinese folk/traditional religion	1.29	1.00
	Christianity	1.05	1.00
	Collapsed affiliations with prevalence<3%	1.46	1.00

Table S8a. India: Demographic descriptive statistics

**Characteristic**	**N = 8,206**[1]
**Age group**	
1943 or earlier (current age: 80+ years)	39 (0.5%)
1943-1953 (current age: 70-79 years)	208 (2.5%)
1953-1963 (current age: 60-69 years)	800 (9.8%)
1963-1973 (current age: 50-59 years)	936 (11%)
1973-1983 (current age: 40-49 years)	1,406 (17%)
1983-1993 (current age: 30-39 years)	1,919 (23%)
1993-1998 (current age: 25-29 years)	982 (12%)
1998-2005 (current age: 18-24 years)	1,915 (23%)
(Missing)	0 (0%)
**Gender**	
1. Male	4,207 (51%)
2. Female	3,999 (49%)
3. Other	0 (0%)
(Missing)	0 (0%)
**Marital status**	
1. Single/Never been married	1,450 (18%)
2. Married	6,216 (76%)
3. Separated	32 (0.4%)
4. Divorced	14 (0.2%)
5. Widowed	274 (3.3%)
6. Domestic partner	181 (2.2%)
(Missing)	39 (0.5%)
**Employment**	
1. Employed for an employer	1,690 (21%)
2. Self-employed	2,185 (27%)
3. Retired	164 (2.0%)
4. Student	362 (4.4%)
5. Homemaker	2,688 (33%)
6. Unemployed and looking for a job	610 (7.4%)
7. None of these/Other	476 (5.8%)
(Missing)	30 (0.4%)
**Religious service attendance**	
1. More than once a week	1,947 (24%)
2. Once a week	1,997 (24%)
3. One to three times a month	1,803 (22%)
4. A few times a year	1,277 (16%)
5. Never	1,137 (14%)
(Missing)	44 (0.5%)
**Education**	
1. Up to 8	5,952 (73%)
2. 9-15	1,842 (22%)
3. 16+	410 (5.0%)
(Missing)	2 (<0.1%)
**Immigration**	
1. Born in this country	8,118 (99%)
2. Born in another country	77 (0.9%)

**Characteristic**	**N = 8,206**[1]
(Missing)	11 (0.1%)
**Religious affiliation**	
1. Christianity	191 (2.3%)
10. Taoism	0 (0%)
11. Confucianism	0 (0%)
12. Primal, Animist, or Folk religion	19 (0.2%)
13. Spiritism	0 (0%)
14. Umbanda, Candomblé, and other African-derived religions	0 (0%)
15. Chinese folk/traditional religion	0 (0%)
2. Islam	1,027 (13%)
3. Hinduism	6,640 (81%)
4. Buddhism	138 (1.7%)
5. Judaism	0 (0%)
6. Sikhism	86 (1.0%)
7. Baha'i	0 (0%)
8. Jainism	8 (<0.1%)
9. Shinto	2 (<0.1%)
96. Some other religion	45 (0.5%)
97. No religion/Atheist/Agnostic	13 (0.2%)
(Missing)	37 (0.4%)
**RACE ETHNICITY**	
(Missing)	163 (2.0%)
601. India: General	2,239 (27%)
602. India: Other backward caste	2,744 (33%)
603. India: Schedule caste	2,330 (28%)
604. India: Schedule tribe	730 (8.9%)

[1]n (%)

Table S8b. India: Childhood descriptive statistics

**Characteristic**	**N = 8,206**[1]
**Age**	38 (15)
**Year of birth**	
1943 or earlier (current age: 80+ years)	39 (0.5%)
1943-1953 (current age: 70-79 years)	208 (2.5%)
1953-1963 (current age: 60-69 years)	800 (9.8%)
1963-1973 (current age: 50-59 years)	936 (11%)
1973-1983 (current age: 40-49 years)	1,406 (17%)
1983-1993 (current age: 30-39 years)	1,919 (23%)
1993-1998 (current age: 25-29 years)	982 (12%)
1998-2005 (current age: 18-24 years)	1,915 (23%)
(Missing)	0 (0%)
**Gender**	
1. Male	4,207 (51%)
2. Female	3,999 (49%)
3. Other	0 (0%)
(Missing)	0 (0%)
**Parent marital status**	
1. Parents were married	3,564 (43%)
2. Parents were divorced	151 (1.8%)
3. Parents were never married	708 (8.6%)
4. One or both of them had died	600 (7.3%)
5. Unsure	24 (0.3%)
(Missing)	3,159 (38%)
**Age 12 religious service attendance**	
1. At least once a week	3,451 (42%)
2. One to three times a month	1,881 (23%)
3. Less than once a month	1,731 (21%)
4. Never	933 (11%)
(Missing)	210 (2.6%)
**Relationship with mother**	
1. Very good	7,369 (90%)
2. Somewhat good	488 (5.9%)
3. Somewhat bad	56 (0.7%)
4. Very bad	44 (0.5%)
97. (Does not apply)	206 (2.5%)
(Missing)	44 (0.5%)
**Relationship with father**	
1. Very good	7,025 (86%)
2. Somewhat good	628 (7.7%)
3. Somewhat bad	80 (1.0%)
4. Very bad	59 (0.7%)
97. (Does not apply)	333 (4.1%)
(Missing)	81 (1.0%)
**Outsider growing up**	
1. Yes	1,275 (16%)
2. No	6,892 (84%)
(Missing)	38 (0.5%)

Characteristic	N = 8,206[1]
**Abuse**	
1. Yes	980 (12%)
2. No	6,712 (82%)
(Missing)	515 (6.3%)
**Self-rated health growing up**	
1. Excellent	1,420 (17%)
2. Very good	2,439 (30%)
3. Good	2,553 (31%)
4. Fair	1,472 (18%)
5. Poor	294 (3.6%)
(Missing)	28 (0.3%)
**Immigration status**	
1. Born in this country	8,118 (99%)
2. Born in another country	77 (0.9%)
(Missing)	11 (0.1%)
**Subjective financial status of family growing up**	
1. Lived comfortably	3,057 (37%)
2. Got by	1,985 (24%)
3. Found it difficult	1,736 (21%)
4. Found it very difficult	1,383 (17%)
(Missing)	45 (0.5%)
**Religious affiliation**	
1. Christianity	152 (1.8%)
10. Taoism	0 (0%)
11. Confucianism	0 (0%)
12. Primal, Animist, or Folk religion	17 (0.2%)
13. Spiritism	0 (0%)
14. Umbanda, Candomblé, and other African-derived religions	0 (0%)
15. Chinese folk/traditional religion	0 (0%)
2. Islam	1,025 (12%)
3. Hinduism	6,687 (81%)
4. Buddhism	110 (1.3%)
5. Judaism	0 (0%)
6. Sikhism	82 (1.0%)
7. Baha'i	0 (0%)
8. Jainism	7 (<0.1%)
9. Shinto	2 (<0.1%)
96. Some other religion	37 (0.5%)
97. No religion/Atheist/Agnostic	8 (<0.1%)
(Missing)	80 (1.0%)
**Race/Ethnicity**	
(Missing)	163 (2.0%)
601. India: General	2,239 (27%)
602. India: Other backward caste	2,744 (33%)
603. India: Schedule caste	2,330 (28%)
604. India: Schedule tribe	730 (8.9%)

[1]Mean (SD); n (%)

Table S8c. Proportions by demographic category for India

Variable	Category	Proportion	95% CI	SE	p-value
Age group	1998-2005 (current age: 18-24 years)	0.681	(0.644, 0.718)	0.019	1.67e-06
	1943 or earlier (current age: 80+ years)	0.549	(0.303, 0.795)	0.120	
	1943-1953 (current age: 70-79 years)	0.501	(0.403, 0.598)	0.049	
	1953-1963 (current age: 60-69 years)	0.507	(0.450, 0.564)	0.029	
	1963-1973 (current age: 50-59 years)	0.586	(0.541, 0.630)	0.023	
	1973-1983 (current age: 40-49 years)	0.641	(0.610, 0.671)	0.016	
	1983-1993 (current age: 30-39 years)	0.627	(0.600, 0.654)	0.014	
	1993-1998 (current age: 25-29 years)	0.624	(0.588, 0.660)	0.018	
Gender	Male	0.668	(0.647, 0.689)	0.011	5.08e-10
	Female	0.573	(0.549, 0.596)	0.012	
	Other	*	*	*	
Marital status	Single/Never been married	0.698	(0.659, 0.738)	0.020	1.57e-09
	Divorced	0.871	(0.665, 1.000)	0.083	
	Domestic partner	0.452	(0.351, 0.553)	0.051	
	Married	0.617	(0.599, 0.635)	0.009	
	Separated	0.461	(0.186, 0.736)	0.133	
	Widowed	0.436	(0.345, 0.527)	0.046	
Employment	Employed for an employer	0.630	(0.596, 0.664)	0.017	3.09e-06
	Homemaker	0.575	(0.547, 0.604)	0.014	
	None of these/Other	0.645	(0.574, 0.716)	0.036	
	Retired	0.510	(0.407, 0.614)	0.052	
	Self-employed	0.643	(0.613, 0.672)	0.015	
	Student	0.728	(0.669, 0.787)	0.030	
	Unemployed and looking for a job	0.675	(0.621, 0.729)	0.028	
Religious service attendance	Never	0.551	(0.509, 0.594)	0.022	2.70e-03
	A few times a year	0.654	(0.617, 0.692)	0.019	
	More than once a week	0.654	(0.622, 0.687)	0.017	
	Once a week	0.613	(0.580, 0.645)	0.017	
	One to three times a month	0.617	(0.583, 0.651)	0.017	
Education	9-15	0.704	(0.680, 0.728)	0.012	6.66e-16
	16+	0.729	(0.685, 0.773)	0.022	

Variable	Category	Proportion	95% CI	SE	p-value
	Up to 8	0.588	(0.568, 0.609)	0.010	
Immigration status	Born in this country	0.622	(0.605, 0.639)	0.009	5.42e-01
	Born in another country	0.577	(0.428, 0.725)	0.074	
Religious affiliation	No religion/Atheist/Agnostic	*	*	*	5.40e-05
	Christianity	*	*	*	
	Combined	0.571	(0.517, 0.624)	0.027	
	Spiritism	*	*	*	
	Umbanda, Candomblé, and other African-derived religions	*	*	*	
	Buddhism	*	*	*	
	Islam	0.535	(0.486, 0.584)	0.025	
	Chinese folk/traditional religion	*	*	*	
	Hinduism	0.639	(0.621, 0.657)	0.009	
	Judaism	*	*	*	
	Primal, Animist, or Folk religion	*	*	*	

Table S8d. India: Childhood predictors regression analysis

Variable	Category	Risk-Ratio	logRR SE	RR 95% CI	Global p-value
Relationship with your mother growing up	(Ref: Very bad/somewhat bad)				0.114
	Very good/somewhat good	0.923	0.048	(0.84,1.01)	
Relationship with your father growing up	(Ref: Very bad/somewhat bad)				0.546
	Very good/somewhat good	0.981	0.047	(0.89,1.08)	
Parents married to each other when you were around 12 years old	(Ref: Parents married)				0.457
	One or both of them had died	0.987	0.029	(0.93,1.04)	
	Parents were divorced	1.073	0.044	(0.98,1.17)	
	Parents were never married	0.996	0.028	(0.94,1.05)	
	Unsure	1.078	0.086	(0.91,1.28)	
Feelings about familys household income when growing up	(Ref: Got by)				0.054
	Found it difficult	1.002	0.022	(0.96,1.05)	
	Found it very difficult	0.952	0.026	(0.90,1.00)	
	Lived comfortably	1.045	0.019	(1.01,1.08)	
Physically or sexually abused when growing up	(Ref: No)				0.058
		1.048	0.024	(1.00,1.10)	
Felt like an outsider in your family when growing up	(Ref: No)				0.031
	Yes	1.049	0.022	(1.01,1.09)	
Your health when growing up	(Ref: Good)				0.073
	Excellent	1.050	0.024	(1.00,1.10)	
	Fair	1.016	0.023	(0.97,1.06)	
	Poor	1.025	0.042	(0.95,1.11)	
	Very good	1.046	0.020	(1.01,1.09)	
Born in This	(Ref: Born in this				0.582

Variable	Category	Risk-Ratio	logRR SE	RR 95% CI	Global p-value
country	country)				
	Born in another country	0.985	0.073	(0.85,1.14)	
How Often You Attended Religious Services or Worshiped When You Were Around 12 Years Old	(Ref: Never)				0.494
	At least once a week	1.028	0.025	(0.98,1.08)	
	Less than once a month	1.016	0.027	(0.96,1.07)	
	One to three times a month	1.027	0.027	(0.97,1.08)	
Year of birth (age group)	(Ref: 1998-2005; current age: 18-24)				4.65e-05
	1943 or earlier (current age: 80+ years)	0.874	0.118	(0.69,1.10)	
	1943-1953 (current age: 70-79 years)	0.847	0.052	(0.76,0.94)	
	1953-1963 (current age: 60-69 years)	0.860	0.034	(0.81,0.92)	
	1963-1973 (current age: 50-59 years)	0.918	0.029	(0.87,0.97)	
	1973-1983 (current age: 40-49 years)	0.967	0.024	(0.92,1.01)	
	1983-1993 (current age: 30-39 years)	0.957	0.023	(0.91,1.00)	
	1993-1998 (current age: 25-29 years)	0.943	0.025	(0.90,0.99)	
Religion when twelve years old	(Ref: Hinduism)				2.68e-05
	Collapsed affiliations with prevalence<3%	0.947	0.031	(0.89,1.01)	
	Islam	0.898	0.025	(0.86,0.94)	
Race plurality (prominent race/ethnic group [0] or not [1])	(Ref: Plurality group)				0.326
	Non-plurality groups	0.982	0.017	(0.95,1.02)	

Table S8e. India: Sensitivity to unmeasured confounding of childhood predictors

Variable	Category	E-value for Estimate	E-value for 95% CI
Relationship with your mother growing up	(Ref: Very bad/somewhat bad)		
	Very good/somewhat good	1.38	1.00
Relationship with your father growing up	(Ref: Very bad/somewhat bad)		
	Very good/somewhat good	1.16	1.00
Parents married to each other when you were around 12 years old	(Ref: Parents married)		
	One or both of them had died	1.13	1.00
	Parents were divorced	1.35	1.00
	Parents were never married	1.07	1.00
	Unsure	1.37	1.00
Feelings about familys household income when growing up	(Ref: Got by)		
	Found it difficult	1.05	1.00
	Found it very difficult	1.28	1.00
	Lived comfortably	1.26	1.09
Physically or sexually abused when growing up	(Ref: No)		
		1.27	1.00
Felt like an outsider in your family when growing up	(Ref: No)		
	Yes	1.28	1.08
Your health when growing up	(Ref: Good)		
	Excellent	1.28	1.04
	Fair	1.15	1.00
	Poor	1.19	1.00
	Very good	1.27	1.09
Born in This country	(Ref: Born in this country)		
	Born in another country	1.14	1.00
How Often You Attended Religious Services or Worshiped When You Were Around 12 Years Old	(Ref: Never)		
	At least once a week	1.20	1.00
	Less than once a month	1.14	1.00
	One to three times a month	1.20	1.00
Year of birth (age group)	(Ref: 1998-2005; current age: 18-24)		
	1943 or earlier (current age:	1.55	1.00

Variable	Category	E-value for Estimate	E-value for 95% CI
	80+ years)		
	1943-1953 (current age: 70-79 years)	1.64	1.33
	1953-1963 (current age: 60-69 years)	1.60	1.40
	1963-1973 (current age: 50-59 years)	1.40	1.20
	1973-1983 (current age: 40-49 years)	1.22	1.00
	1983-1993 (current age: 30-39 years)	1.26	1.00
	1993-1998 (current age: 25-29 years)	1.31	1.11
Religion when twelve years old	(Ref: Hinduism)		
	Collapsed affiliations with prevalence<3%	1.30	1.00
	Islam	1.47	1.31
Race plurality (prominent race/ethnic group [0] or not [1])	(Ref: Plurality group)		
	Non-plurality groups	1.16	1.00

Table S9a. Indonesia: Demographic descriptive statistics

Characteristic	N = 2,675[1]
**Age group**	
1943 or earlier (current age: 80+ years)	11 (0.4%)
1943-1953 (current age: 70-79 years)	34 (1.3%)
1953-1963 (current age: 60-69 years)	196 (7.3%)
1963-1973 (current age: 50-59 years)	444 (17%)
1973-1983 (current age: 40-49 years)	546 (20%)
1983-1993 (current age: 30-39 years)	598 (22%)
1993-1998 (current age: 25-29 years)	322 (12%)
1998-2005 (current age: 18-24 years)	523 (20%)
(Missing)	0 (0%)
**Gender**	
1. Male	1,342 (50%)
2. Female	1,329 (50%)
3. Other	1 (<0.1%)
(Missing)	3 (0.1%)
**Marital status**	
1. Single/Never been married	592 (22%)
2. Married	1,794 (67%)
3. Separated	19 (0.7%)
4. Divorced	70 (2.6%)
5. Widowed	185 (6.9%)
6. Domestic partner	6 (0.2%)
(Missing)	8 (0.3%)
**Employment**	
1. Employed for an employer	453 (17%)
2. Self-employed	932 (35%)
3. Retired	25 (0.9%)
4. Student	129 (4.8%)
5. Homemaker	773 (29%)
6. Unemployed and looking for a job	203 (7.6%)
7. None of these/Other	155 (5.8%)
(Missing)	4 (0.2%)
**Religious service attendance**	
1. More than once a week	981 (37%)
2. Once a week	1,015 (38%)
3. One to three times a month	313 (12%)
4. A few times a year	246 (9.2%)
5. Never	113 (4.2%)
(Missing)	6 (0.2%)
**Education**	
1. Up to 8	1,390 (52%)
2. 9-15	1,169 (44%)
3. 16+	115 (4.3%)
(Missing)	1 (<0.1%)
**Immigration**	
1. Born in this country	2,664 (100%)
2. Born in another country	11 (0.4%)

**Characteristic**	**N = 2,675**[1]
(Missing)	0 (0%)
**Religious affiliation**	
1. Christianity	205 (7.6%)
10. Taoism	1 (<0.1%)
11. Confucianism	0 (0%)
12. Primal, Animist, or Folk religion	0 (0%)
13. Spiritism	0 (0%)
14. Umbanda, Candomblé, and other African-derived religions	0 (0%)
15. Chinese folk/traditional religion	0 (0%)
2. Islam	2,434 (91%)
3. Hinduism	31 (1.2%)
4. Buddhism	1 (<0.1%)
5. Judaism	0 (0%)
6. Sikhism	0 (0%)
7. Baha'i	0 (0%)
8. Jainism	0 (0%)
9. Shinto	0 (0%)
96. Some other religion	3 (<0.1%)
97. No religion/Atheist/Agnostic	0 (0%)
(Missing)	0 (0%)
**RACE ETHNICITY**	
(Missing)	16 (0.6%)
701. Indonesia: Banjar/Melayu Banjar	138 (5.2%)
702. Indonesia: Betawi	67 (2.5%)
703. Indonesia: Bugis	93 (3.5%)
704. Indonesia: Jawa	1,043 (39%)
705. Indonesia: Madura	109 (4.1%)
706. Indonesia: Minangkabau	117 (4.4%)
707. Indonesia: Sunda/Parahyangan	493 (18%)
708. Indonesia: Bali	30 (1.1%)
709. Indonesia: Batak	55 (2.0%)
710. Indonesia: Makasar	31 (1.2%)
9996. Other	483 (18%)

[1]n (%)

Table S9b. Indonesia: Childhood descriptive statistics

**Characteristic**	**N = 2,675**[1]
**Age**	39 (14)
**Year of birth**	
1943 or earlier (current age: 80+ years)	11 (0.4%)
1943-1953 (current age: 70-79 years)	34 (1.3%)
1953-1963 (current age: 60-69 years)	196 (7.3%)
1963-1973 (current age: 50-59 years)	444 (17%)
1973-1983 (current age: 40-49 years)	546 (20%)
1983-1993 (current age: 30-39 years)	598 (22%)
1993-1998 (current age: 25-29 years)	322 (12%)
1998-2005 (current age: 18-24 years)	523 (20%)
(Missing)	0 (0%)
**Gender**	
1. Male	1,342 (50%)
2. Female	1,329 (50%)
3. Other	1 (<0.1%)
(Missing)	3 (0.1%)
**Parent marital status**	
1. Parents were married	2,032 (76%)
2. Parents were divorced	219 (8.2%)
3. Parents were never married	18 (0.7%)
4. One or both of them had died	352 (13%)
5. Unsure	30 (1.1%)
(Missing)	24 (0.9%)
**Age 12 religious service attendance**	
1. At least once a week	2,042 (76%)
2. One to three times a month	390 (15%)
3. Less than once a month	115 (4.3%)
4. Never	111 (4.1%)
(Missing)	17 (0.6%)
**Relationship with mother**	
1. Very good	2,363 (88%)
2. Somewhat good	245 (9.2%)
3. Somewhat bad	20 (0.8%)
4. Very bad	12 (0.4%)
97. (Does not apply)	19 (0.7%)
(Missing)	16 (0.6%)
**Relationship with father**	
1. Very good	2,290 (86%)
2. Somewhat good	283 (11%)
3. Somewhat bad	26 (1.0%)
4. Very bad	16 (0.6%)
97. (Does not apply)	35 (1.3%)
(Missing)	25 (0.9%)
**Outsider growing up**	
1. Yes	145 (5.4%)
2. No	2,525 (94%)
(Missing)	4 (0.2%)

Characteristic	N = 2,675[1]
**Abuse**	
1. Yes	221 (8.3%)
2. No	2,442 (91%)
(Missing)	11 (0.4%)
**Self-rated health growing up**	
1. Excellent	441 (16%)
2. Very good	725 (27%)
3. Good	967 (36%)
4. Fair	524 (20%)
5. Poor	18 (0.7%)
(Missing)	0 (0%)
**Immigration status**	
1. Born in this country	2,664 (100%)
2. Born in another country	11 (0.4%)
(Missing)	0 (0%)
**Subjective financial status of family growing up**	
1. Lived comfortably	1,161 (43%)
2. Got by	1,217 (45%)
3. Found it difficult	191 (7.1%)
4. Found it very difficult	104 (3.9%)
(Missing)	2 (<0.1%)
**Religious affiliation**	
1. Christianity	210 (7.9%)
10. Taoism	0 (0%)
11. Confucianism	0 (0%)
12. Primal, Animist, or Folk religion	0 (<0.1%)
13. Spiritism	0 (0%)
14. Umbanda, Candomblé, and other African-derived religions	0 (0%)
15. Chinese folk/traditional religion	0 (0%)
2. Islam	2,426 (91%)
3. Hinduism	31 (1.2%)
4. Buddhism	5 (0.2%)
5. Judaism	0 (0%)
6. Sikhism	0 (0%)
7. Baha'i	0 (0%)
8. Jainism	0 (0%)
9. Shinto	0 (0%)
96. Some other religion	0 (0%)
97. No religion/Atheist/Agnostic	2 (<0.1%)
(Missing)	0 (<0.1%)
**Race/Ethnicity**	
(Missing)	16 (0.6%)
701. Indonesia: Banjar/Melayu Banjar	138 (5.2%)
702. Indonesia: Betawi	67 (2.5%)
703. Indonesia: Bugis	93 (3.5%)
704. Indonesia: Jawa	1,043 (39%)
705. Indonesia: Madura	109 (4.1%)

**Characteristic**	**N = 2,675**[1]
706. Indonesia: Minangkabau	117 (4.4%)
707. Indonesia: Sunda/Parahyangan	493 (18%)
708. Indonesia: Bali	30 (1.1%)
709. Indonesia: Batak	55 (2.0%)
710. Indonesia: Makasar	31 (1.2%)
9996. Other	483 (18%)

[1]Mean (SD); n (%)

Table S9c. Indonesia: Proportions by demographic category

Variable	Category	Proportion	95% CI	SE	p-value
Age group	1998-2005 (current age: 18-24 years)	0.895	(0.851, 0.940)	0.023	6.76e-09
	1943 or earlier (current age: 80+ years)	0.695	(0.000, 1.000)	0.246	
	1943-1953 (current age: 70-79 years)	0.540	(0.246, 0.835)	0.143	
	1953-1963 (current age: 60-69 years)	0.628	(0.504, 0.751)	0.062	
	1963-1973 (current age: 50-59 years)	0.687	(0.613, 0.761)	0.038	
	1973-1983 (current age: 40-49 years)	0.746	(0.692, 0.801)	0.028	
	1983-1993 (current age: 30-39 years)	0.819	(0.780, 0.859)	0.020	
	1993-1998 (current age: 25-29 years)	0.889	(0.843, 0.934)	0.023	
Gender	Male	0.800	(0.767, 0.834)	0.017	< 1e-16
	Female	0.775	(0.743, 0.806)	0.016	
	Other	1.000	*	*	
Marital status	Single/Never been married	0.907	(0.873, 0.941)	0.017	5.75e-09
	Divorced	0.781	(0.651, 0.912)	0.065	
	Domestic partner	0.740	*	*	
	Married	0.764	(0.734, 0.793)	0.015	
	Separated	0.733	(0.495, 0.971)	0.108	
	Widowed	0.644	(0.522, 0.767)	0.062	
Employment	Employed for an employer	0.788	(0.728, 0.848)	0.031	1.15e-12
	Homemaker	0.755	(0.712, 0.799)	0.022	
	None of these/Other	0.680	(0.558, 0.802)	0.062	
	Retired	0.737	(0.549, 0.925)	0.088	
	Self-employed	0.795	(0.755, 0.834)	0.020	
	Student	0.959	(0.918, 1.000)	0.021	
	Unemployed and looking for a job	0.857	(0.789, 0.925)	0.035	
Religious service attendance	Never	0.795	(0.681, 0.909)	0.057	5.14e-01
	A few times a year	0.820	(0.756, 0.883)	0.032	
	More than once a week	0.794	(0.755, 0.834)	0.020	
	Once a week	0.765	(0.728, 0.802)	0.019	
	One to three times a month	0.813	(0.748, 0.878)	0.033	
Education	9-15	0.882	(0.864, 0.901)	0.009	< 1e-16
	16+	0.925	(0.886, 0.963)	0.019	

Variable	Category	Proportion	95% CI	SE	p-value
	Up to 8	0.696	(0.657, 0.736)	0.020	
Immigration status	Born in this country	0.787	(0.764, 0.810)	0.012	4.26e-01
	Born in another country	0.877	(0.613, 1.000)	0.112	
Religious affiliation	No religion/Atheist/Agnostic	*	*	*	9.05e-01
	Christianity	0.801	(0.724, 0.879)	0.039	
	Combined	0.806	(0.619, 0.993)	0.092	
	Spiritism	*	*	*	
	Umbanda, Candomblé, and other African-derived religions	*	*	*	
	Buddhism	*	*	*	
	Islam	0.786	(0.762, 0.811)	0.012	
	Chinese folk/traditional religion	*	*	*	
	Hinduism	*	*	*	
	Judaism	*	*	*	
	Primal, Animist, or Folk religion	*	*	*	

Table S9d. Indonesia: Childhood predictors regression analysis

Variable	Category	Risk-Ratio	logRR SE	RR 95% CI	Global p-value
Relationship with your mother growing up	(Ref: Very bad/somewhat bad)				0.556
	Very good/somewhat good	0.969	0.089	(0.81,1.15)	
Relationship with your father growing up	(Ref: Very bad/somewhat bad)				0.598
	Very good/somewhat good	1.007	0.063	(0.89,1.14)	
Parents married to each other when you were around 12 years old	(Ref: Parents married)				0.645
	One or both of them had died	0.994	0.040	(0.92,1.08)	
	Parents were divorced	1.002	0.039	(0.93,1.08)	
	Parents were never married	1.182	0.127	(0.92,1.52)	
	Unsure	0.959	0.125	(0.75,1.23)	
Feelings about familys household income when growing up	(Ref: Got by)				0.435
	Found it difficult	1.040	0.049	(0.95,1.14)	
	Found it very difficult	1.084	0.068	(0.95,1.24)	
	Lived comfortably	1.024	0.022	(0.98,1.07)	
Physically or sexually abused when growing up	(Ref: No)				0.606
		0.997	0.046	(0.91,1.09)	
Felt like an outsider in your family when growing up	(Ref: No)				0.544
	Yes	1.022	0.053	(0.92,1.13)	
Your health when growing up	(Ref: Good)				0.644
	Excellent	1.030	0.033	(0.97,1.10)	
	Fair	1.036	0.033	(0.97,1.10)	
	Poor	1.024	0.140	(0.78,1.35)	
	Very good	1.013	0.030	(0.96,1.07)	
Born in This	(Ref: Born in this				0.538

Variable	Category	Risk-Ratio	logRR SE	RR 95% CI	Global p-value
country	country)				
	Born in another country	1.056	0.128	(0.82,1.36)	
How Often You Attended Religious Services or Worshiped When You Were Around 12 Years Old	(Ref: Never)				0.439
	At least once a week	1.008	0.061	(0.89,1.14)	
	Less than once a month	1.087	0.070	(0.95,1.25)	
	One to three times a month	0.915	0.071	(0.80,1.05)	
Year of birth (age group)	(Ref: 1998-2005; current age: 18-24)				1.32e-05
	1943 or earlier (current age: 80+ years)	0.773	0.252	(0.47,1.27)	
	1943-1953 (current age: 70-79 years)	0.680	0.141	(0.52,0.90)	
	1953-1963 (current age: 60-69 years)	0.758	0.067	(0.66,0.87)	
	1963-1973 (current age: 50-59 years)	0.806	0.045	(0.74,0.88)	
	1973-1983 (current age: 40-49 years)	0.860	0.035	(0.80,0.92)	
	1983-1993 (current age: 30-39 years)	0.927	0.029	(0.88,0.98)	
	1993-1998 (current age: 25-29 years)	0.995	0.030	(0.94,1.06)	
Religion when twelve years old	(Ref: Islam)				0.448
	Christianity	1.054	0.047	(0.96,1.16)	
	Collapsed affiliations with prevalence<3%	1.069	0.100	(0.88,1.30)	
Race plurality (prominent race/ethnic group [0] or not [1])	(Ref: Plurality group)				0.001
	Non-plurality groups	0.932	0.022	(0.89,0.97)	

Table S9e. Indonesia: Sensitivity to unmeasured confounding of childhood predictors

Variable	Category	E-value for Estimate	E-value for 95% CI
Relationship with your mother growing up	(Ref: Very bad/somewhat bad)		
	Very good/somewhat good	1.21	1.00
Relationship with your father growing up	(Ref: Very bad/somewhat bad)		
	Very good/somewhat good	1.09	1.00
Parents married to each other when you were around 12 years old	(Ref: Parents married)		
	One or both of them had died	1.08	1.00
	Parents were divorced	1.04	1.00
	Parents were never married	1.65	1.00
	Unsure	1.25	1.00
Feelings about familys household income when growing up	(Ref: Got by)		
	Found it difficult	1.24	1.00
	Found it very difficult	1.38	1.00
	Lived comfortably	1.18	1.00
Physically or sexually abused when growing up	(Ref: No)		
		1.06	1.00
Felt like an outsider in your family when growing up	(Ref: No)		
	Yes	1.17	1.00
Your health when growing up	(Ref: Good)		
	Excellent	1.21	1.00
	Fair	1.23	1.00
	Poor	1.18	1.00
	Very good	1.13	1.00
Born in This country	(Ref: Born in this country)		
	Born in another country	1.30	1.00
How Often You Attended Religious Services or Worshiped When You Were Around 12 Years Old	(Ref: Never)		
	At least once a week	1.10	1.00
	Less than once a month	1.39	1.00
	One to three times a month	1.41	1.00
Year of birth (age group)	(Ref: 1998-2005; current age: 18-24)		
	1943 or earlier (current age: 80+ years)	1.91	1.00
	1943-1953 (current age: 70-79 years)	2.30	1.48

Variable	Category	E-value for Estimate	E-value for 95% CI
	1953-1963 (current age: 60-69 years)	1.97	1.58
	1963-1973 (current age: 50-59 years)	1.79	1.53
	1973-1983 (current age: 40-49 years)	1.60	1.39
	1983-1993 (current age: 30-39 years)	1.37	1.16
	1993-1998 (current age: 25-29 years)	1.07	1.00
Religion when twelve years old	(Ref: Islam)		
	Christianity	1.29	1.00
	Collapsed affiliations with prevalence<3%	1.34	1.00
Race plurality (prominent race/ethnic group [0] or not [1])	(Ref: Plurality group)		
	Non-plurality groups	1.35	1.20

Table S10a. Israel: Demographic descriptive statistics

**Characteristic**	**N = 2,476**[1]
**Age group**	
1943 or earlier (current age: 80+ years)	59 (2.4%)
1943-1953 (current age: 70-79 years)	233 (9.4%)
1953-1963 (current age: 60-69 years)	310 (13%)
1963-1973 (current age: 50-59 years)	331 (13%)
1973-1983 (current age: 40-49 years)	415 (17%)
1983-1993 (current age: 30-39 years)	442 (18%)
1993-1998 (current age: 25-29 years)	271 (11%)
1998-2005 (current age: 18-24 years)	415 (17%)
(Missing)	0 (0%)
**Gender**	
1. Male	1,205 (49%)
2. Female	1,270 (51%)
3. Other	0 (0%)
(Missing)	0 (<0.1%)
**Marital status**	
1. Single/Never been married	606 (24%)
2. Married	1,331 (54%)
3. Separated	24 (1.0%)
4. Divorced	194 (7.8%)
5. Widowed	153 (6.2%)
6. Domestic partner	123 (5.0%)
(Missing)	45 (1.8%)
**Employment**	
1. Employed for an employer	1,186 (48%)
2. Self-employed	262 (11%)
3. Retired	406 (16%)
4. Student	275 (11%)
5. Homemaker	166 (6.7%)
6. Unemployed and looking for a job	98 (3.9%)
7. None of these/Other	77 (3.1%)
(Missing)	6 (0.3%)
**Religious service attendance**	
1. More than once a week	490 (20%)
2. Once a week	349 (14%)
3. One to three times a month	250 (10%)
4. A few times a year	614 (25%)
5. Never	768 (31%)
(Missing)	6 (0.2%)
**Education**	
1. Up to 8	161 (6.5%)
2. 9-15	1,072 (43%)
3. 16+	1,241 (50%)
(Missing)	2 (<0.1%)
**Immigration**	
1. Born in this country	1,915 (77%)
2. Born in another country	556 (22%)

Characteristic	N = 2,476[1]
(Missing)	5 (0.2%)
**Religious affiliation**	
1. Christianity	29 (1.2%)
10. Taoism	0 (0%)
11. Confucianism	0 (0%)
12. Primal, Animist, or Folk religion	0 (0%)
13. Spiritism	0 (0%)
14. Umbanda, Candomblé, and other African-derived religions	0 (0%)
15. Chinese folk/traditional religion	0 (0%)
2. Islam	451 (18%)
3. Hinduism	0 (0%)
4. Buddhism	0 (0%)
5. Judaism	1,946 (79%)
6. Sikhism	0 (0%)
7. Baha'i	2 (<0.1%)
8. Jainism	0 (0%)
9. Shinto	0 (0%)
96. Some other religion	3 (0.1%)
97. No religion/Atheist/Agnostic	43 (1.7%)
(Missing)	3 (0.1%)
**RACE ETHNICITY**	
(Missing)	17 (0.7%)
801. Israel: Jewish	1,961 (79%)
802. Israel: Arab	475 (19%)
9996. Other	22 (0.9%)

[1]n (%)

Table S10b. Israel: Childhood descriptive statistics

Characteristic	N = 2,476[1]
**Age**	44 (18)
**Year of birth**	
1943 or earlier (current age: 80+ years)	59 (2.4%)
1943-1953 (current age: 70-79 years)	233 (9.4%)
1953-1963 (current age: 60-69 years)	310 (13%)
1963-1973 (current age: 50-59 years)	331 (13%)
1973-1983 (current age: 40-49 years)	415 (17%)
1983-1993 (current age: 30-39 years)	442 (18%)
1993-1998 (current age: 25-29 years)	271 (11%)
1998-2005 (current age: 18-24 years)	415 (17%)
(Missing)	0 (0%)
**Gender**	
1. Male	1,205 (49%)
2. Female	1,270 (51%)
3. Other	0 (0%)
(Missing)	0 (<0.1%)
**Parent marital status**	
1. Parents were married	2,166 (87%)
2. Parents were divorced	186 (7.5%)
3. Parents were never married	18 (0.7%)
4. One or both of them had died	77 (3.1%)
5. Unsure	9 (0.4%)
(Missing)	19 (0.8%)
**Age 12 religious service attendance**	
1. At least once a week	588 (24%)
2. One to three times a month	293 (12%)
3. Less than once a month	552 (22%)
4. Never	1,031 (42%)
(Missing)	11 (0.5%)
**Relationship with mother**	
1. Very good	1,835 (74%)
2. Somewhat good	512 (21%)
3. Somewhat bad	75 (3.0%)
4. Very bad	10 (0.4%)
97. (Does not apply)	33 (1.4%)
(Missing)	12 (0.5%)
**Relationship with father**	
1. Very good	1,571 (63%)
2. Somewhat good	578 (23%)
3. Somewhat bad	172 (6.9%)
4. Very bad	19 (0.7%)
97. (Does not apply)	122 (4.9%)
(Missing)	14 (0.6%)
**Outsider growing up**	
1. Yes	226 (9.1%)
2. No	2,205 (89%)
(Missing)	45 (1.8%)

**Characteristic**	**N = 2,476**[1]
**Abuse**	
1. Yes	0 (0%)
2. No	0 (0%)
(Missing)	2,476 (100%)
**Self-rated health growing up**	
1. Excellent	1,193 (48%)
2. Very good	884 (36%)
3. Good	323 (13%)
4. Fair	72 (2.9%)
5. Poor	1 (<0.1%)
(Missing)	3 (0.1%)
**Immigration status**	
1. Born in this country	1,915 (77%)
2. Born in another country	556 (22%)
(Missing)	5 (0.2%)
**Subjective financial status of family growing up**	
1. Lived comfortably	599 (24%)
2. Got by	1,255 (51%)
3. Found it difficult	454 (18%)
4. Found it very difficult	159 (6.4%)
(Missing)	9 (0.4%)
**Religious affiliation**	
1. Christianity	44 (1.8%)
10. Taoism	0 (0%)
11. Confucianism	0 (0%)
12. Primal, Animist, or Folk religion	0 (0%)
13. Spiritism	0 (0%)
14. Umbanda, Candomblé, and other African-derived religions	0 (0%)
15. Chinese folk/traditional religion	0 (0%)
2. Islam	445 (18%)
3. Hinduism	0 (0%)
4. Buddhism	0 (0%)
5. Judaism	1,933 (78%)
6. Sikhism	0 (0%)
7. Baha'i	1 (<0.1%)
8. Jainism	0 (0%)
9. Shinto	0 (0%)
96. Some other religion	3 (0.1%)
97. No religion/Atheist/Agnostic	45 (1.8%)
(Missing)	5 (0.2%)
**Race/Ethnicity**	
(Missing)	17 (0.7%)
801. Israel: Jewish	1,961 (79%)
802. Israel: Arab	475 (19%)
9996. Other	22 (0.9%)

[1]Mean (SD); n (%)

Table S10c. Israel: Proportions by demographic category

Variable	Category	Proportion	95% CI	SE	p-value
Age group	1998-2005 (current age: 18-24 years)	0.899	(0.849, 0.950)	0.026	1.02e-01
	1943 or earlier (current age: 80+ years)	0.801	(0.670, 0.932)	0.065	
	1943-1953 (current age: 70-79 years)	0.775	(0.698, 0.852)	0.039	
	1953-1963 (current age: 60-69 years)	0.812	(0.741, 0.882)	0.036	
	1963-1973 (current age: 50-59 years)	0.859	(0.806, 0.912)	0.027	
	1973-1983 (current age: 40-49 years)	0.847	(0.805, 0.889)	0.021	
	1983-1993 (current age: 30-39 years)	0.837	(0.792, 0.883)	0.023	
	1993-1998 (current age: 25-29 years)	0.889	(0.832, 0.945)	0.029	
Gender	Male	0.827	(0.794, 0.860)	0.017	3.80e-02
	Female	0.868	(0.832, 0.904)	0.018	
	Other	*	*	*	
Marital status	Single/Never been married	0.896	(0.856, 0.937)	0.021	2.85e-03
	Divorced	0.886	(0.827, 0.944)	0.030	
	Domestic partner	0.880	(0.785, 0.975)	0.048	
	Married	0.828	(0.792, 0.864)	0.018	
	Separated	0.828	(0.662, 0.994)	0.077	
	Widowed	0.763	(0.658, 0.868)	0.053	
Employment	Employed for an employer	0.867	(0.837, 0.897)	0.015	8.04e-05
	Homemaker	0.709	(0.569, 0.850)	0.071	
	None of these/Other	0.762	(0.626, 0.897)	0.068	
	Retired	0.823	(0.763, 0.884)	0.031	
	Self-employed	0.841	(0.778, 0.903)	0.032	
	Student	0.948	(0.914, 0.982)	0.017	
	Unemployed and looking for a job	0.760	(0.605, 0.915)	0.078	
Religious service attendance	Never	0.818	(0.780, 0.856)	0.019	4.47e-03
	A few times a year	0.871	(0.830, 0.913)	0.021	
	More than once a week	0.889	(0.852, 0.926)	0.019	
	Once a week	0.818	(0.750, 0.885)	0.034	
	One to three times	0.845	(0.784, 0.905)	0.031	

Variable	Category	Proportion	95% CI	SE	p-value
	a month				
Education	9-15	0.822	(0.781, 0.864)	0.021	2.49e-04
	16+	0.896	(0.872, 0.920)	0.012	
	Up to 8	0.650	(0.484, 0.816)	0.084	
Immigration status	Born in this country	0.853	(0.821, 0.886)	0.017	3.63e-01
	Born in another country	0.830	(0.783, 0.877)	0.024	
Religious affiliation	No religion/Atheist/Agnostic	*	*	*	1.16e-04
	Christianity	*	*	*	
	Combined	0.799	(0.659, 0.939)	0.070	
	Spiritism	*	*	*	
	Umbanda, Candomblé, and other African-derived religions	*	*	*	
	Buddhism	*	*	*	
	Islam	0.672	(0.575, 0.769)	0.049	
	Chinese folk/traditional religion	*	*	*	
	Hinduism	*	*	*	
	Judaism	0.891	(0.865, 0.916)	0.013	
	Primal, Animist, or Folk religion	*	*	*	

Table S10d. Israel: Childhood predictors regression analysis

Variable	Category	Risk-Ratio	logRR SE	RR 95% CI	Global p-value
Relationship with your mother growing up	(Ref: Very bad/somewhat bad)				0.591
	Very good/somewhat good	0.993	0.043	(0.91,1.08)	
Relationship with your father growing up	(Ref: Very bad/somewhat bad)				0.550
	Very good/somewhat good	1.016	0.042	(0.94,1.10)	
Parents married to each other when you were around 12 years old	(Ref: Parents married)				0.197
	One or both of them had died	1.070	0.053	(0.96,1.19)	
	Parents were divorced	0.993	0.045	(0.91,1.09)	
	Parents were never married	0.832	0.118	(0.66,1.05)	
	Unsure	0.692	0.196	(0.47,1.02)	
Feelings about familys household income when growing up	(Ref: Got by)				0.158
	Found it difficult	0.997	0.027	(0.95,1.05)	
	Found it very difficult	0.914	0.044	(0.84,1.00)	
	Lived comfortably	1.005	0.019	(0.97,1.04)	
Felt like an outsider in your family when growing up	(Ref: No)				0.439
	Yes	1.025	0.031	(0.96,1.09)	
Your health when growing up	(Ref: Good)				0.291
	Excellent	0.975	0.028	(0.92,1.03)	
	Fair	0.964	0.045	(0.88,1.05)	
	Poor	1.078	0.054	(0.97,1.20)	
	Very good	0.957	0.026	(0.91,1.01)	
Born in This country	(Ref: Born in this country)				0.518
	Born in another country	0.986	0.028	(0.93,1.04)	
How Often You	(Ref: Never)				0.501

Variable	Category	Risk-Ratio	logRR SE	RR 95% CI	Global p-value
Attended Religious Services or Worshiped When You Were Around 12 Years Old					
	At least once a week	1.031	0.028	(0.98,1.09)	
	Less than once a month	1.027	0.024	(0.98,1.08)	
	One to three times a month	0.991	0.037	(0.92,1.07)	
Year of birth (age group)	(Ref: 1998-2005; current age: 18-24)				0.026
	1943 or earlier (current age: 80+ years)	0.912	0.082	(0.78,1.07)	
	1943-1953 (current age: 70-79 years)	0.869	0.049	(0.79,0.96)	
	1953-1963 (current age: 60-69 years)	0.912	0.042	(0.84,0.99)	
	1963-1973 (current age: 50-59 years)	0.951	0.040	(0.88,1.03)	
	1973-1983 (current age: 40-49 years)	0.951	0.032	(0.89,1.01)	
	1983-1993 (current age: 30-39 years)	0.948	0.035	(0.88,1.02)	
	1993-1998 (current age: 25-29 years)	0.989	0.039	(0.92,1.07)	
Religion when twelve years old	(Ref: Judaism)				0.197
	Collapsed affiliations with prevalence<3%	1.083	0.046	(0.99,1.18)	
	Islam	0.970	0.068	(0.85,1.11)	
Race plurality (prominent race/ethnic group [0] or not [1])	(Ref: Plurality group)				1.42e-04
	Non-plurality groups	0.825	0.051	(0.75,0.91)	

Table S10e. Israel: Sensitivity to unmeasured confounding of childhood predictors

Variable	Category	E-value for Estimate	E-value for 95% CI
Relationship with your mother growing up	(Ref: Very bad/somewhat bad)		
	Very good/somewhat good	1.09	1.00
Relationship with your father growing up	(Ref: Very bad/somewhat bad)		
	Very good/somewhat good	1.14	1.00
Parents married to each other when you were around 12 years old	(Ref: Parents married)		
	One or both of them had died	1.34	1.00
	Parents were divorced	1.09	1.00
	Parents were never married	1.69	1.00
	Unsure	2.25	1.00
Feelings about familys household income when growing up	(Ref: Got by)		
	Found it difficult	1.06	1.00
	Found it very difficult	1.41	1.06
	Lived comfortably	1.07	1.00
Felt like an outsider in your family when growing up	(Ref: No)		
	Yes	1.18	1.00
Your health when growing up	(Ref: Good)		
	Excellent	1.19	1.00
	Fair	1.23	1.00
	Poor	1.37	1.00
	Very good	1.26	1.00
Born in This country	(Ref: Born in this country)		
	Born in another country	1.14	1.00
How Often You Attended Religious Services or Worshiped When You Were Around 12 Years Old	(Ref: Never)		
	At least once a week	1.21	1.00
	Less than once a month	1.19	1.00
	One to three times a month	1.10	1.00
Year of birth (age group)	(Ref: 1998-2005; current age: 18-24)		
	1943 or earlier (current age: 80+ years)	1.42	1.00
	1943-1953 (current age: 70-79 years)	1.57	1.26
	1953-1963 (current age: 60-69 years)	1.42	1.11
	1963-1973 (current age: 50-	1.28	1.00

Variable	Category	E-value for Estimate	E-value for 95% CI
	59 years)		
	1973-1983 (current age: 40-49 years)	1.28	1.00
	1983-1993 (current age: 30-39 years)	1.30	1.00
	1993-1998 (current age: 25-29 years)	1.12	1.00
Religion when twelve years old	(Ref: Judaism)		
	Collapsed affiliations with prevalence<3%	1.38	1.00
	Islam	1.21	1.00
Race plurality (prominent race/ethnic group [0] or not [1])	(Ref: Plurality group)		
	Non-plurality groups	1.72	1.43

Table S11a. Japan: Demographic descriptive statistics

Characteristic	N = 13,886[1]
**Age group**	
1943 or earlier (current age: 80+ years)	302 (2.2%)
1943-1953 (current age: 70-79 years)	2,394 (17%)
1953-1963 (current age: 60-69 years)	2,734 (20%)
1963-1973 (current age: 50-59 years)	2,513 (18%)
1973-1983 (current age: 40-49 years)	2,363 (17%)
1983-1993 (current age: 30-39 years)	1,959 (14%)
1993-1998 (current age: 25-29 years)	527 (3.8%)
1998-2005 (current age: 18-24 years)	1,094 (7.9%)
(Missing)	0 (0%)
**Gender**	
1. Male	6,536 (47%)
2. Female	7,301 (53%)
3. Other	16 (0.1%)
(Missing)	33 (0.2%)
**Marital status**	
1. Single/Never been married	3,324 (24%)
2. Married	8,079 (58%)
3. Separated	114 (0.8%)
4. Divorced	1,475 (11%)
5. Widowed	770 (5.5%)
6. Domestic partner	79 (0.6%)
(Missing)	44 (0.3%)
**Employment**	
1. Employed for an employer	7,478 (54%)
2. Self-employed	1,165 (8.4%)
3. Retired	1,718 (12%)
4. Student	351 (2.5%)
5. Homemaker	803 (5.8%)
6. Unemployed and looking for a job	408 (2.9%)
7. None of these/Other	1,945 (14%)
(Missing)	19 (0.1%)
**Religious service attendance**	
1. More than once a week	201 (1.4%)
2. Once a week	199 (1.4%)
3. One to three times a month	513 (3.7%)
4. A few times a year	2,083 (15%)
5. Never	10,813 (78%)
(Missing)	77 (0.6%)
**Education**	
1. Up to 8	371 (2.7%)
2. 9-15	10,167 (73%)
3. 16+	3,347 (24%)
(Missing)	0 (0%)
**Immigration**	
1. Born in this country	13,317 (96%)
2. Born in another country	89 (0.6%)

**Characteristic**	**N = 13,886**[1]
(Missing)	481 (3.5%)
**Religious affiliation**	
1. Christianity	248 (1.8%)
10. Taoism	4 (<0.1%)
11. Confucianism	10 (<0.1%)
12. Primal, Animist, or Folk religion	8 (<0.1%)
13. Spiritism	0 (0%)
14. Umbanda, Candomblé, and other African-derived religions	0 (0%)
15. Chinese folk/traditional religion	0 (0%)
2. Islam	2 (<0.1%)
3. Hinduism	3 (<0.1%)
4. Buddhism	4,530 (33%)
5. Judaism	0 (<0.1%)
6. Sikhism	5 (<0.1%)
7. Baha'i	0 (0%)
8. Jainism	3 (<0.1%)
9. Shinto	298 (2.1%)
96. Some other religion	29 (0.2%)
97. No religion/Atheist/Agnostic	8,496 (61%)
(Missing)	251 (1.8%)

[1]n (%)

Table S11b. Japan: Childhood descriptive statistics

**Characteristic**	**N = 13,886**[1]
**Age**	52 (17)
**Year of birth**	
1943 or earlier (current age: 80+ years)	302 (2.2%)
1943-1953 (current age: 70-79 years)	2,394 (17%)
1953-1963 (current age: 60-69 years)	2,734 (20%)
1963-1973 (current age: 50-59 years)	2,513 (18%)
1973-1983 (current age: 40-49 years)	2,363 (17%)
1983-1993 (current age: 30-39 years)	1,959 (14%)
1993-1998 (current age: 25-29 years)	527 (3.8%)
1998-2005 (current age: 18-24 years)	1,094 (7.9%)
(Missing)	0 (0%)
**Gender**	
1. Male	6,536 (47%)
2. Female	7,301 (53%)
3. Other	16 (0.1%)
(Missing)	33 (0.2%)
**Parent marital status**	
1. Parents were married	12,053 (87%)
2. Parents were divorced	752 (5.4%)
3. Parents were never married	385 (2.8%)
4. One or both of them had died	484 (3.5%)
5. Unsure	189 (1.4%)
(Missing)	23 (0.2%)
**Age 12 religious service attendance**	
1. At least once a week	263 (1.9%)
2. One to three times a month	550 (4.0%)
3. Less than once a month	3,367 (24%)
4. Never	9,640 (69%)
(Missing)	66 (0.5%)
**Relationship with mother**	
1. Very good	3,809 (27%)
2. Somewhat good	6,437 (46%)
3. Somewhat bad	1,819 (13%)
4. Very bad	535 (3.9%)
97. (Does not apply)	1,246 (9.0%)
(Missing)	41 (0.3%)
**Relationship with father**	
1. Very good	2,797 (20%)
2. Somewhat good	6,157 (44%)
3. Somewhat bad	2,313 (17%)
4. Very bad	875 (6.3%)
97. (Does not apply)	1,705 (12%)
(Missing)	39 (0.3%)
**Outsider growing up**	
1. Yes	1,294 (9.3%)
2. No	11,635 (84%)
(Missing)	957 (6.9%)

Characteristic	N = 13,886[1]
**Abuse**	
1. Yes	974 (7.0%)
2. No	12,852 (93%)
(Missing)	60 (0.4%)
**Self-rated health growing up**	
1. Excellent	1,810 (13%)
2. Very good	4,817 (35%)
3. Good	4,552 (33%)
4. Fair	2,162 (16%)
5. Poor	493 (3.6%)
(Missing)	52 (0.4%)
**Immigration status**	
1. Born in this country	13,317 (96%)
2. Born in another country	89 (0.6%)
(Missing)	481 (3.5%)
**Subjective financial status of family growing up**	
1. Lived comfortably	5,548 (40%)
2. Got by	5,978 (43%)
3. Found it difficult	1,638 (12%)
4. Found it very difficult	684 (4.9%)
(Missing)	37 (0.3%)
**Religious affiliation**	
1. Christianity	221 (1.6%)
10. Taoism	6 (<0.1%)
11. Confucianism	16 (0.1%)
12. Primal, Animist, or Folk religion	4 (<0.1%)
13. Spiritism	0 (0%)
14. Umbanda, Candomblé, and other African-derived religions	0 (0%)
15. Chinese folk/traditional religion	0 (0%)
2. Islam	0 (0%)
3. Hinduism	5 (<0.1%)
4. Buddhism	4,421 (32%)
5. Judaism	0 (0%)
6. Sikhism	0 (0%)
7. Baha'i	4 (<0.1%)
8. Jainism	1 (<0.1%)
9. Shinto	238 (1.7%)
96. Some other religion	28 (0.2%)
97. No religion/Atheist/Agnostic	8,817 (63%)
(Missing)	126 (0.9%)

[1]Mean (SD); n (%)

Table S11c. Japan: Proportions by demographic category

Variable	Category	Proportion	95% CI	SE	p-value
Age group	1998-2005 (current age: 18-24 years)	0.440	(0.392, 0.489)	0.025	< 1e-16
	1943 or earlier (current age: 80+ years)	0.636	(0.576, 0.695)	0.030	
	1943-1953 (current age: 70-79 years)	0.586	(0.564, 0.607)	0.011	
	1953-1963 (current age: 60-69 years)	0.498	(0.477, 0.519)	0.011	
	1963-1973 (current age: 50-59 years)	0.405	(0.384, 0.427)	0.011	
	1973-1983 (current age: 40-49 years)	0.379	(0.354, 0.404)	0.013	
	1983-1993 (current age: 30-39 years)	0.334	(0.304, 0.363)	0.015	
	1993-1998 (current age: 25-29 years)	0.317	(0.261, 0.373)	0.028	
Gender	Male	0.378	(0.364, 0.392)	0.007	< 1e-16
	Female	0.505	(0.490, 0.519)	0.007	
	Other	0.365	(0.046, 0.683)	0.144	
Marital status	Single/Never been married	0.409	(0.388, 0.429)	0.010	2.68e-10
	Divorced	0.466	(0.431, 0.501)	0.018	
	Domestic partner	0.545	(0.393, 0.697)	0.076	
	Married	0.440	(0.427, 0.453)	0.007	
	Separated	0.464	(0.341, 0.587)	0.062	
	Widowed	0.593	(0.545, 0.641)	0.024	
Employment	Employed for an employer	0.388	(0.373, 0.403)	0.008	< 1e-16
	Homemaker	0.541	(0.507, 0.575)	0.017	
	None of these/Other	0.510	(0.486, 0.533)	0.012	
	Retired	0.531	(0.506, 0.556)	0.013	
	Self-employed	0.531	(0.496, 0.566)	0.018	
	Student	0.499	(0.414, 0.584)	0.043	
	Unemployed and looking for a job	0.325	(0.279, 0.370)	0.023	
Religious service attendance	Never	0.416	(0.405, 0.428)	0.006	< 1e-16
	A few times a year	0.540	(0.513, 0.567)	0.014	
	More than once a week	0.688	(0.616, 0.761)	0.037	
	Once a week	0.699	(0.615, 0.783)	0.043	
	One to three times a month	0.458	(0.403, 0.513)	0.028	
Education	9-15	0.416	(0.405, 0.428)	0.006	< 1e-16
	16+	0.544	(0.522, 0.566)	0.011	

Variable	Category	Proportion	95% CI	SE	p-value
	Up to 8	0.327	(0.265, 0.388)	0.031	
Immigration status	Born in this country	0.445	(0.434, 0.455)	0.005	6.94e-01
	Born in another country	0.421	(0.274, 0.567)	0.073	
Religious affiliation	No religion/Atheist/Agnostic	0.396	(0.383, 0.409)	0.007	< 1e-16
	Christianity	*	*	*	
	Combined	0.640	(0.593, 0.687)	0.024	
	Spiritism	*	*	*	
	Umbanda, Candomblé, and other African-derived religions	*	*	*	
	Buddhism	0.509	(0.492, 0.527)	0.009	
	Islam	*	*	*	
	Chinese folk/traditional religion	*	*	*	
	Hinduism	*	*	*	
	Judaism	*	*	*	
	Primal, Animist, or Folk religion	*	*	*	

Table S11d. Japan: Childhood predictors regression analysis

Variable	Category	Risk-Ratio	logRR SE	RR 95% CI	Global p-value
Relationship with your mother growing up	(Ref: Very bad/somewhat bad)				2.40e-04
	Very good/somewhat good	1.057	0.015	(1.03,1.09)	
Relationship with your father growing up	(Ref: Very bad/somewhat bad)				0.251
	Very good/somewhat good	1.018	0.014	(0.99,1.05)	
Parents married to each other when you were around 12 years old	(Ref: Parents married)				0.003
	One or both of them had died	1.025	0.028	(0.97,1.08)	
	Parents were divorced	1.084	0.024	(1.03,1.14)	
	Parents were never married	0.958	0.031	(0.90,1.02)	
	Unsure	0.962	0.041	(0.89,1.04)	
Feelings about familys household income when growing up	(Ref: Got by)				4.35e-09
	Found it difficult	0.954	0.016	(0.92,0.98)	
	Found it very difficult	0.948	0.025	(0.90,1.00)	
	Lived comfortably	1.072	0.012	(1.05,1.10)	
Physically or sexually abused when growing up	(Ref: No)				2.45e-07
		1.119	0.022	(1.07,1.17)	
Felt like an outsider in your family when growing up	(Ref: No)				0.429
	Yes	1.016	0.019	(0.98,1.06)	
Your health when growing up	(Ref: Good)				0.009
	Excellent	1.048	0.018	(1.01,1.09)	
	Fair	1.020	0.015	(0.99,1.05)	
	Poor	1.000	0.027	(0.95,1.05)	
	Very good	1.038	0.012	(1.01,1.06)	
Born in This	(Ref: Born in this				0.575

Variable	Category	Risk-Ratio	logRR SE	RR 95% CI	Global p-value
country	country)				
	Born in another country	0.983	0.069	(0.86,1.13)	
How Often You Attended Religious Services or Worshiped When You Were Around 12 Years Old	(Ref: Never)				6.66e-16
	At least once a week	1.287	0.035	(1.20,1.38)	
	Less than once a month	1.151	0.012	(1.12,1.18)	
	One to three times a month	1.203	0.027	(1.14,1.27)	
Year of birth (age group)	(Ref: 1998-2005; current age: 18-24)				1.77e-06
	1943 or earlier (current age: 80+ years)	1.201	0.040	(1.11,1.30)	
	1943-1953 (current age: 70-79 years)	1.148	0.027	(1.09,1.21)	
	1953-1963 (current age: 60-69 years)	1.058	0.026	(1.00,1.11)	
	1963-1973 (current age: 50-59 years)	0.974	0.027	(0.92,1.03)	
	1973-1983 (current age: 40-49 years)	0.938	0.027	(0.89,0.99)	
	1983-1993 (current age: 30-39 years)	0.908	0.028	(0.86,0.96)	
	1993-1998 (current age: 25-29 years)	0.894	0.036	(0.83,0.96)	
Religion when twelve years old	(Ref: No religion/Atheist/Agnostic)				0.003
	Buddhism	1.033	0.012	(1.01,1.06)	
	Collapsed affiliations with prevalence<3%	1.092	0.029	(1.03,1.16)	

Table S11e. Japan: Sensitivity to unmeasured confounding of childhood predictors

Variable	Category	E-value for Estimate	E-value for 95% CI
Relationship with your mother growing up	(Ref: Very bad/somewhat bad)		
	Very good/somewhat good	1.30	1.19
Relationship with your father growing up	(Ref: Very bad/somewhat bad)		
	Very good/somewhat good	1.15	1.00
Parents married to each other when you were around 12 years old	(Ref: Parents married)		
	One or both of them had died	1.19	1.00
	Parents were divorced	1.38	1.22
	Parents were never married	1.26	1.00
	Unsure	1.24	1.00
Feelings about familys household income when growing up	(Ref: Got by)		
	Found it difficult	1.27	1.14
	Found it very difficult	1.29	1.07
	Lived comfortably	1.35	1.27
Physically or sexually abused when growing up	(Ref: No)		
		1.48	1.35
Felt like an outsider in your family when growing up	(Ref: No)		
	Yes	1.14	1.00
Your health when growing up	(Ref: Good)		
	Excellent	1.27	1.13
	Fair	1.16	1.00
	Poor	1.02	1.00
	Very good	1.24	1.13
Born in This country	(Ref: Born in this country)		
	Born in another country	1.15	1.00
How Often You Attended Religious Services or Worshiped When You Were Around 12 Years Old	(Ref: Never)		
	At least once a week	1.89	1.69
	Less than once a month	1.57	1.49
	One to three times a month	1.70	1.54
Year of birth (age group)	(Ref: 1998-2005; current age: 18-24)		
	1943 or earlier (current age: 80+ years)	1.69	1.46
	1943-1953 (current age: 70-79 years)	1.56	1.40

Variable	Category	E-value for Estimate	E-value for 95% CI
	1953-1963 (current age: 60-69 years)	1.31	1.07
	1963-1973 (current age: 50-59 years)	1.19	1.00
	1973-1983 (current age: 40-49 years)	1.33	1.12
	1983-1993 (current age: 30-39 years)	1.44	1.26
	1993-1998 (current age: 25-29 years)	1.48	1.26
Religion when twelve years old	(Ref: No religion/Atheist/Agnostic)		
	Buddhism	1.22	1.11
	Collapsed affiliations with prevalence<3%	1.41	1.21

Table S12a. Kenya: Demographic descriptive statistics

Characteristic	N = 9,115[1]
**Age group**	
1943 or earlier (current age: 80+ years)	42 (0.5%)
1943-1953 (current age: 70-79 years)	253 (2.8%)
1953-1963 (current age: 60-69 years)	564 (6.2%)
1963-1973 (current age: 50-59 years)	820 (9.0%)
1973-1983 (current age: 40-49 years)	1,313 (14%)
1983-1993 (current age: 30-39 years)	1,908 (21%)
1993-1998 (current age: 25-29 years)	1,503 (16%)
1998-2005 (current age: 18-24 years)	2,709 (30%)
(Missing)	5 (<0.1%)
**Gender**	
1. Male	4,437 (49%)
2. Female	4,672 (51%)
3. Other	0 (<0.1%)
(Missing)	5 (<0.1%)
**Marital status**	
1. Single/Never been married	3,034 (33%)
2. Married	5,150 (57%)
3. Separated	363 (4.0%)
4. Divorced	72 (0.8%)
5. Widowed	350 (3.8%)
6. Domestic partner	119 (1.3%)
(Missing)	27 (0.3%)
**Employment**	
1. Employed for an employer	1,093 (12%)
2. Self-employed	2,805 (31%)
3. Retired	223 (2.4%)
4. Student	1,072 (12%)
5. Homemaker	1,219 (13%)
6. Unemployed and looking for a job	2,592 (28%)
7. None of these/Other	105 (1.2%)
(Missing)	6 (<0.1%)
**Religious service attendance**	
1. More than once a week	2,191 (24%)
2. Once a week	4,896 (54%)
3. One to three times a month	999 (11%)
4. A few times a year	673 (7.4%)
5. Never	345 (3.8%)
(Missing)	11 (0.1%)
**Education**	
1. Up to 8	3,824 (42%)
2. 9-15	4,700 (52%)
3. 16+	585 (6.4%)
(Missing)	5 (<0.1%)
**Immigration**	
1. Born in this country	9,025 (99%)
2. Born in another country	88 (1.0%)

**Characteristic**	**N = 9,115**[1]
(Missing)	2 (<0.1%)
**Religious affiliation**	
1. Christianity	8,284 (91%)
10. Taoism	0 (0%)
11. Confucianism	0 (0%)
12. Primal, Animist, or Folk religion	6 (<0.1%)
13. Spiritism	0 (0%)
14. Umbanda, Candomblé, and other African-derived religions	0 (0%)
15. Chinese folk/traditional religion	0 (0%)
2. Islam	725 (8.0%)
3. Hinduism	0 (0%)
4. Buddhism	1 (<0.1%)
5. Judaism	4 (<0.1%)
6. Sikhism	0 (0%)
7. Baha'i	1 (<0.1%)
8. Jainism	0 (0%)
9. Shinto	0 (0%)
96. Some other religion	5 (<0.1%)
97. No religion/Atheist/Agnostic	83 (0.9%)
(Missing)	8 (<0.1%)
**RACE ETHNICITY**	
(Missing)	21 (0.2%)
1001. Kenya: Luhya	1,535 (17%)
1002. Kenya: Luo	935 (10%)
1003. Kenya: Kalenjin	1,093 (12%)
1004. Kenya: Kamba	1,027 (11%)
1005. Kenya: Kikuyu	1,657 (18%)
1006. Kenya: Kisii	604 (6.6%)
1007. Kenya: Maasai	175 (1.9%)
1008. Kenya: Meru	541 (5.9%)
1009. Kenya: Kenyan Somali/Somali	304 (3.3%)
1010. Kenya: Miji Kenda tribes	581 (6.4%)
1011. Kenya: Embu	166 (1.8%)
9996. Other	476 (5.2%)

[1]n (%)

Table S12b. Kenya: Childhood descriptive statistics

Characteristic	N = 9,115[1]
**Age**	35 (15)
Unknown	5
**Year of birth**	
1943 or earlier (current age: 80+ years)	42 (0.5%)
1943-1953 (current age: 70-79 years)	253 (2.8%)
1953-1963 (current age: 60-69 years)	564 (6.2%)
1963-1973 (current age: 50-59 years)	820 (9.0%)
1973-1983 (current age: 40-49 years)	1,313 (14%)
1983-1993 (current age: 30-39 years)	1,908 (21%)
1993-1998 (current age: 25-29 years)	1,503 (16%)
1998-2005 (current age: 18-24 years)	2,709 (30%)
(Missing)	5 (<0.1%)
**Gender**	
1. Male	4,437 (49%)
2. Female	4,672 (51%)
3. Other	0 (<0.1%)
(Missing)	5 (<0.1%)
**Parent marital status**	
1. Parents were married	7,394 (81%)
2. Parents were divorced	567 (6.2%)
3. Parents were never married	546 (6.0%)
4. One or both of them had died	384 (4.2%)
5. Unsure	74 (0.8%)
(Missing)	151 (1.7%)
**Age 12 religious service attendance**	
1. At least once a week	7,340 (81%)
2. One to three times a month	1,324 (15%)
3. Less than once a month	215 (2.4%)
4. Never	169 (1.9%)
(Missing)	68 (0.7%)
**Relationship with mother**	
1. Very good	7,559 (83%)
2. Somewhat good	1,131 (12%)
3. Somewhat bad	110 (1.2%)
4. Very bad	90 (1.0%)
97. (Does not apply)	177 (1.9%)
(Missing)	49 (0.5%)
**Relationship with father**	
1. Very good	6,370 (70%)
2. Somewhat good	1,525 (17%)
3. Somewhat bad	179 (2.0%)
4. Very bad	165 (1.8%)
97. (Does not apply)	765 (8.4%)
(Missing)	112 (1.2%)
**Outsider growing up**	
1. Yes	972 (11%)
2. No	8,100 (89%)

Characteristic	N = 9,115[1]
(Missing)	43 (0.5%)
**Abuse**	
1. Yes	1,028 (11%)
2. No	8,052 (88%)
(Missing)	35 (0.4%)
**Self-rated health growing up**	
1. Excellent	3,559 (39%)
2. Very good	2,086 (23%)
3. Good	2,033 (22%)
4. Fair	1,119 (12%)
5. Poor	302 (3.3%)
(Missing)	17 (0.2%)
**Immigration status**	
1. Born in this country	9,025 (99%)
2. Born in another country	88 (1.0%)
(Missing)	2 (<0.1%)
**Subjective financial status of family growing up**	
1. Lived comfortably	2,385 (26%)
2. Got by	2,619 (29%)
3. Found it difficult	3,310 (36%)
4. Found it very difficult	788 (8.6%)
(Missing)	13 (0.1%)
**Religious affiliation**	
1. Christianity	8,301 (91%)
10. Taoism	0 (0%)
11. Confucianism	0 (0%)
12. Primal, Animist, or Folk religion	11 (0.1%)
13. Spiritism	0 (0%)
14. Umbanda, Candomblé, and other African-derived religions	0 (0%)
15. Chinese folk/traditional religion	0 (0%)
2. Islam	730 (8.0%)
3. Hinduism	0 (0%)
4. Buddhism	5 (<0.1%)
5. Judaism	5 (<0.1%)
6. Sikhism	0 (<0.1%)
7. Baha'i	4 (<0.1%)
8. Jainism	0 (0%)
9. Shinto	0 (0%)
96. Some other religion	1 (<0.1%)
97. No religion/Atheist/Agnostic	50 (0.6%)
(Missing)	8 (<0.1%)
**Race/Ethnicity**	
(Missing)	21 (0.2%)
1001. Kenya: Luhya	1,535 (17%)
1002. Kenya: Luo	935 (10%)
1003. Kenya: Kalenjin	1,093 (12%)
1004. Kenya: Kamba	1,027 (11%)

**Characteristic**	**N = 9,115**[1]
1005. Kenya: Kikuyu	1,657 (18%)
1006. Kenya: Kisii	604 (6.6%)
1007. Kenya: Maasai	175 (1.9%)
1008. Kenya: Meru	541 (5.9%)
1009. Kenya: Kenyan Somali/Somali	304 (3.3%)
1010. Kenya: Miji Kenda tribes	581 (6.4%)
1011. Kenya: Embu	166 (1.8%)
9996. Other	476 (5.2%)

[1]Mean (SD); n (%)

Table S12c. Kenya: Proportions by demographic category

Variable	Category	Proportion	95% CI	SE	p-value
Age group	1998-2005 (current age: 18-24 years)	0.813	(0.793, 0.832)	0.010	5.22e-11
	1943 or earlier (current age: 80+ years)	0.459	(0.212, 0.707)	0.121	
	1943-1953 (current age: 70-79 years)	0.686	(0.593, 0.779)	0.047	
	1953-1963 (current age: 60-69 years)	0.658	(0.596, 0.721)	0.032	
	1963-1973 (current age: 50-59 years)	0.668	(0.615, 0.720)	0.027	
	1973-1983 (current age: 40-49 years)	0.707	(0.672, 0.743)	0.018	
	1983-1993 (current age: 30-39 years)	0.753	(0.728, 0.779)	0.013	
	1993-1998 (current age: 25-29 years)	0.788	(0.764, 0.811)	0.012	
Gender	Male	0.785	(0.765, 0.805)	0.010	< 1e-16
	Female	0.723	(0.703, 0.744)	0.010	
	Other	1.000	*	*	
Marital status	Single/Never been married	0.813	(0.794, 0.831)	0.009	3.23e-10
	Divorced	0.719	(0.599, 0.838)	0.060	
	Domestic partner	0.768	(0.660, 0.877)	0.055	
	Married	0.728	(0.706, 0.750)	0.011	
	Separated	0.722	(0.667, 0.777)	0.028	
	Widowed	0.646	(0.564, 0.728)	0.042	
Employment	Employed for an employer	0.802	(0.772, 0.831)	0.015	2.22e-16
	Homemaker	0.646	(0.605, 0.687)	0.021	
	None of these/Other	0.730	(0.620, 0.840)	0.056	
	Retired	0.657	(0.565, 0.750)	0.047	
	Self-employed	0.740	(0.715, 0.766)	0.013	
	Student	0.858	(0.833, 0.884)	0.013	
	Unemployed and looking for a job	0.763	(0.741, 0.785)	0.011	
Religious service attendance	Never	0.728	(0.665, 0.791)	0.032	4.20e-01
	A few times a year	0.777	(0.736, 0.817)	0.021	
	More than once a week	0.734	(0.701, 0.766)	0.016	
	Once a week	0.759	(0.739, 0.780)	0.010	
	One to three times a month	0.760	(0.724, 0.796)	0.018	
Education	9-15	0.802	(0.784, 0.819)	0.009	< 1e-16
	16+	0.879	(0.848, 0.909)	0.016	

Variable	Category	Proportion	95% CI	SE	p-value
	Up to 8	0.675	(0.647, 0.703)	0.014	
Immigration status	Born in this country	0.753	(0.736, 0.771)	0.009	8.31e-01
	Born in another country	0.742	(0.634, 0.850)	0.054	
Religious affiliation	No religion/Atheist/Agnostic	*	*	*	1.75e-01
	Christianity	0.758	(0.740, 0.776)	0.009	
	Combined	0.755	(0.667, 0.843)	0.044	
	Spiritism	*	*	*	
	Umbanda, Candomblé, and other African-derived religions	*	*	*	
	Buddhism	*	*	*	
	Islam	0.698	(0.637, 0.759)	0.031	
	Chinese folk/traditional religion	*	*	*	
	Hinduism	*	*	*	
	Judaism	*	*	*	
	Primal, Animist, or Folk religion	*	*	*	

Table S12d. Kenya: Childhood predictors regression analysis

Variable	Category	Risk-Ratio	logRR SE	RR 95% CI	Global p-value
Relationship with your mother growing up	(Ref: Very bad/somewhat bad)				0.117
	Very good/somewhat good	1.071	0.041	(0.99,1.16)	
Relationship with your father growing up	(Ref: Very bad/somewhat bad)				0.045
	Very good/somewhat good	0.949	0.025	(0.90,1.00)	
Parents married to each other when you were around 12 years old	(Ref: Parents married)				0.038
	One or both of them had died	1.046	0.028	(0.99,1.10)	
	Parents were divorced	1.004	0.023	(0.96,1.05)	
	Parents were never married	1.004	0.019	(0.97,1.04)	
	Unsure	1.117	0.042	(1.03,1.21)	
Feelings about familys household income when growing up	(Ref: Got by)				0.004
	Found it difficult	0.962	0.015	(0.94,0.99)	
	Found it very difficult	0.931	0.023	(0.89,0.97)	
	Lived comfortably	1.004	0.014	(0.98,1.03)	
Physically or sexually abused when growing up	(Ref: No)				0.613
		1.000	0.019	(0.96,1.04)	
Felt like an outsider in your family when growing up	(Ref: No)				0.176
	Yes	0.971	0.020	(0.93,1.01)	
Your health when growing up	(Ref: Good)				0.605
	Excellent	1.012	0.016	(0.98,1.04)	
	Fair	1.021	0.019	(0.98,1.06)	
	Poor	0.966	0.038	(0.90,1.04)	
	Very good	1.003	0.018	(0.97,1.04)	
Born in This	(Ref: Born in this				0.570

Variable	Category	Risk-Ratio	logRR SE	RR 95% CI	Global p-value
country	country)				
	Born in another country	0.985	0.054	(0.89,1.10)	
How Often You Attended Religious Services or Worshiped When You Were Around 12 Years Old	(Ref: Never)				0.644
	At least once a week	1.045	0.053	(0.94,1.16)	
	Less than once a month	0.991	0.066	(0.87,1.13)	
	One to three times a month	1.041	0.055	(0.94,1.16)	
Year of birth (age group)	(Ref: 1998-2005; current age: 18-24)				2.21e-06
	1943 or earlier (current age: 80+ years)	0.703	0.116	(0.56,0.88)	
	1943-1953 (current age: 70-79 years)	0.886	0.048	(0.81,0.97)	
	1953-1963 (current age: 60-69 years)	0.863	0.032	(0.81,0.92)	
	1963-1973 (current age: 50-59 years)	0.874	0.027	(0.83,0.92)	
	1973-1983 (current age: 40-49 years)	0.910	0.019	(0.88,0.94)	
	1983-1993 (current age: 30-39 years)	0.948	0.014	(0.92,0.97)	
	1993-1998 (current age: 25-29 years)	0.978	0.014	(0.95,1.01)	
Religion when twelve years old	(Ref: Christianity)				0.029
	Collapsed affiliations with prevalence<3%	1.017	0.064	(0.90,1.15)	
	Islam	0.925	0.031	(0.87,0.98)	
Race plurality (prominent race/ethnic group [0] or not [1])	(Ref: Plurality group)				0.612
	Non-plurality groups	1.000	0.023	(0.95,1.05)	

Table S12e. Kenya: Sensitivity to unmeasured confounding of childhood predictors

Variable	Category	E-value for Estimate	E-value for 95% CI
Relationship with your mother growing up	(Ref: Very bad/somewhat bad)		
	Very good/somewhat good	1.35	1.00
Relationship with your father growing up	(Ref: Very bad/somewhat bad)		
	Very good/somewhat good	1.29	1.05
Parents married to each other when you were around 12 years old	(Ref: Parents married)		
	One or both of them had died	1.26	1.00
	Parents were divorced	1.06	1.00
	Parents were never married	1.06	1.00
	Unsure	1.48	1.20
Feelings about familys household income when growing up	(Ref: Got by)		
	Found it difficult	1.24	1.11
	Found it very difficult	1.36	1.19
	Lived comfortably	1.06	1.00
Physically or sexually abused when growing up	(Ref: No)		
		1.01	1.00
Felt like an outsider in your family when growing up	(Ref: No)		
	Yes	1.21	1.00
Your health when growing up	(Ref: Good)		
	Excellent	1.12	1.00
	Fair	1.17	1.00
	Poor	1.23	1.00
	Very good	1.06	1.00
Born in This country	(Ref: Born in this country)		
	Born in another country	1.14	1.00
How Often You Attended Religious Services or Worshiped When You Were Around 12 Years Old	(Ref: Never)		
	At least once a week	1.26	1.00
	Less than once a month	1.10	1.00
	One to three times a month	1.25	1.00
Year of birth (age group)	(Ref: 1998-2005; current age: 18-24)		
	1943 or earlier (current age: 80+ years)	2.20	1.52
	1943-1953 (current age: 70-79 years)	1.51	1.19

Variable	Category	E-value for Estimate	E-value for 95% CI
	1953-1963 (current age: 60-69 years)	1.59	1.40
	1963-1973 (current age: 50-59 years)	1.55	1.39
	1973-1983 (current age: 40-49 years)	1.43	1.31
	1983-1993 (current age: 30-39 years)	1.29	1.19
	1993-1998 (current age: 25-29 years)	1.18	1.00
Religion when twelve years old	(Ref: Christianity)		
	Collapsed affiliations with prevalence<3%	1.15	1.00
	Islam	1.38	1.15
Race plurality (prominent race/ethnic group [0] or not [1])	(Ref: Plurality group)		
	Non-plurality groups	1.02	1.00

Table S13a. Mexico: Demographic descriptive statistics

**Characteristic**	**N = 2,126**[1]
**Age group**	
1943 or earlier (current age: 80+ years)	26 (1.2%)
1943-1953 (current age: 70-79 years)	98 (4.6%)
1953-1963 (current age: 60-69 years)	214 (10%)
1963-1973 (current age: 50-59 years)	325 (15%)
1973-1983 (current age: 40-49 years)	351 (17%)
1983-1993 (current age: 30-39 years)	483 (23%)
1993-1998 (current age: 25-29 years)	219 (10%)
1998-2005 (current age: 18-24 years)	410 (19%)
(Missing)	0 (0%)
**Gender**	
1. Male	1,004 (47%)
2. Female	1,117 (53%)
3. Other	1 (<0.1%)
(Missing)	4 (0.2%)
**Marital status**	
1. Single/Never been married	556 (26%)
2. Married	744 (35%)
3. Separated	139 (6.5%)
4. Divorced	91 (4.3%)
5. Widowed	121 (5.7%)
6. Domestic partner	452 (21%)
(Missing)	23 (1.1%)
**Employment**	
1. Employed for an employer	684 (32%)
2. Self-employed	399 (19%)
3. Retired	128 (6.0%)
4. Student	115 (5.4%)
5. Homemaker	538 (25%)
6. Unemployed and looking for a job	178 (8.4%)
7. None of these/Other	60 (2.8%)
(Missing)	24 (1.1%)
**Religious service attendance**	
1. More than once a week	220 (10%)
2. Once a week	451 (21%)
3. One to three times a month	234 (11%)
4. A few times a year	774 (36%)
5. Never	434 (20%)
(Missing)	14 (0.6%)
**Education**	
1. Up to 8	487 (23%)
2. 9-15	1,204 (57%)
3. 16+	435 (20%)
(Missing)	0 (0%)
**Immigration**	
1. Born in this country	2,068 (97%)
2. Born in another country	37 (1.7%)

**Characteristic**	**N = 2,126**[1]
(Missing)	21 (1.0%)
**Religious affiliation**	
1. Christianity	1,784 (84%)
10. Taoism	0 (<0.1%)
11. Confucianism	0 (0%)
12. Primal, Animist, or Folk religion	10 (0.5%)
13. Spiritism	0 (0%)
14. Umbanda, Candomblé, and other African-derived religions	0 (0%)
15. Chinese folk/traditional religion	0 (0%)
2. Islam	0 (0%)
3. Hinduism	1 (<0.1%)
4. Buddhism	2 (<0.1%)
5. Judaism	2 (<0.1%)
6. Sikhism	0 (0%)
7. Baha'i	0 (0%)
8. Jainism	0 (0%)
9. Shinto	0 (<0.1%)
96. Some other religion	16 (0.8%)
97. No religion/Atheist/Agnostic	285 (13%)
(Missing)	26 (1.2%)
**RACE ETHNICITY**	
(Missing)	121 (5.7%)
1101. Mexico: White	414 (19%)
1102. Mexico: Mestizo	1,070 (50%)
1103. Mexico: Indigenous	214 (10%)
1104. Mexico: Black	27 (1.3%)
1105. Mexico: Mulatto	28 (1.3%)
9995. Prefer not to answer	116 (5.5%)
9996. Other	137 (6.5%)

[1]n (%)

Table S13b. Mexico: Childhood descriptive statistics

Characteristic	N = 2,126[1]
**Age**	41 (16)
**Year of birth**	
1943 or earlier (current age: 80+ years)	26 (1.2%)
1943-1953 (current age: 70-79 years)	98 (4.6%)
1953-1963 (current age: 60-69 years)	214 (10%)
1963-1973 (current age: 50-59 years)	325 (15%)
1973-1983 (current age: 40-49 years)	351 (17%)
1983-1993 (current age: 30-39 years)	483 (23%)
1993-1998 (current age: 25-29 years)	219 (10%)
1998-2005 (current age: 18-24 years)	410 (19%)
(Missing)	0 (0%)
**Gender**	
1. Male	1,004 (47%)
2. Female	1,117 (53%)
3. Other	1 (<0.1%)
(Missing)	4 (0.2%)
**Parent marital status**	
1. Parents were married	1,509 (71%)
2. Parents were divorced	97 (4.5%)
3. Parents were never married	344 (16%)
4. One or both of them had died	75 (3.5%)
5. Unsure	42 (2.0%)
(Missing)	59 (2.8%)
**Age 12 religious service attendance**	
1. At least once a week	929 (44%)
2. One to three times a month	409 (19%)
3. Less than once a month	407 (19%)
4. Never	355 (17%)
(Missing)	26 (1.2%)
**Relationship with mother**	
1. Very good	1,422 (67%)
2. Somewhat good	503 (24%)
3. Somewhat bad	71 (3.3%)
4. Very bad	42 (2.0%)
97. (Does not apply)	61 (2.9%)
(Missing)	27 (1.3%)
**Relationship with father**	
1. Very good	1,119 (53%)
2. Somewhat good	558 (26%)
3. Somewhat bad	143 (6.7%)
4. Very bad	116 (5.5%)
97. (Does not apply)	174 (8.2%)
(Missing)	15 (0.7%)
**Outsider growing up**	
1. Yes	295 (14%)
2. No	1,796 (84%)
(Missing)	35 (1.7%)

**Characteristic**	**N = 2,126**[1]
**Abuse**	
1. Yes	385 (18%)
2. No	1,699 (80%)
(Missing)	42 (2.0%)
**Self-rated health growing up**	
1. Excellent	662 (31%)
2. Very good	505 (24%)
3. Good	618 (29%)
4. Fair	290 (14%)
5. Poor	45 (2.1%)
(Missing)	6 (0.3%)
**Immigration status**	
1. Born in this country	2,068 (97%)
2. Born in another country	37 (1.7%)
(Missing)	21 (1.0%)
**Subjective financial status of family growing up**	
1. Lived comfortably	650 (31%)
2. Got by	663 (31%)
3. Found it difficult	651 (31%)
4. Found it very difficult	144 (6.8%)
(Missing)	17 (0.8%)
**Religious affiliation**	
1. Christianity	1,981 (93%)
10. Taoism	0 (0%)
11. Confucianism	0 (0%)
12. Primal, Animist, or Folk religion	1 (<0.1%)
13. Spiritism	0 (0%)
14. Umbanda, Candomblé, and other African-derived religions	0 (0%)
15. Chinese folk/traditional religion	0 (0%)
2. Islam	1 (<0.1%)
3. Hinduism	1 (<0.1%)
4. Buddhism	0 (0%)
5. Judaism	6 (0.3%)
6. Sikhism	1 (<0.1%)
7. Baha'i	0 (0%)
8. Jainism	0 (0%)
9. Shinto	0 (0%)
96. Some other religion	4 (0.2%)
97. No religion/Atheist/Agnostic	106 (5.0%)
(Missing)	26 (1.2%)
**Race/Ethnicity**	
(Missing)	121 (5.7%)
1101. Mexico: White	414 (19%)
1102. Mexico: Mestizo	1,070 (50%)
1103. Mexico: Indigenous	214 (10%)
1104. Mexico: Black	27 (1.3%)
1105. Mexico: Mulatto	28 (1.3%)

Characteristic	N = 2,126[1]
9995. Prefer not to answer	116 (5.5%)
9996. Other	137 (6.5%)

[1]Mean (SD); n (%)

Table S13c. Mexico: Proportions by demographic category

Variable	Category	Proportion	95% CI	SE	p-value
Age group	1998-2005 (current age: 18-24 years)	0.862	(0.809, 0.916)	0.027	5.60e-01
	1943 or earlier (current age: 80+ years)	0.669	(0.454, 0.884)	0.101	
	1943-1953 (current age: 70-79 years)	0.794	(0.698, 0.890)	0.048	
	1953-1963 (current age: 60-69 years)	0.825	(0.763, 0.886)	0.031	
	1963-1973 (current age: 50-59 years)	0.814	(0.767, 0.862)	0.024	
	1973-1983 (current age: 40-49 years)	0.811	(0.762, 0.860)	0.025	
	1983-1993 (current age: 30-39 years)	0.795	(0.744, 0.846)	0.026	
	1993-1998 (current age: 25-29 years)	0.825	(0.754, 0.896)	0.036	
Gender	Male	0.835	(0.802, 0.868)	0.017	< 1e-16
	Female	0.803	(0.774, 0.832)	0.015	
	Other	1.000	*	*	
Marital status	Single/Never been married	0.822	(0.775, 0.869)	0.024	2.65e-01
	Divorced	0.868	(0.791, 0.946)	0.039	
	Domestic partner	0.784	(0.730, 0.838)	0.027	
	Married	0.843	(0.809, 0.876)	0.017	
	Separated	0.785	(0.703, 0.866)	0.041	
	Widowed	0.778	(0.681, 0.876)	0.049	
Employment	Employed for an employer	0.835	(0.798, 0.871)	0.019	2.51e-01
	Homemaker	0.770	(0.724, 0.817)	0.024	
	None of these/Other	0.816	(0.685, 0.947)	0.065	
	Retired	0.790	(0.704, 0.877)	0.043	
	Self-employed	0.841	(0.792, 0.891)	0.025	
	Student	0.881	(0.788, 0.974)	0.047	
	Unemployed and looking for a job	0.827	(0.744, 0.910)	0.042	
Religious service attendance	Never	0.778	(0.727, 0.829)	0.026	1.09e-01
	A few times a year	0.828	(0.793, 0.863)	0.018	
	More than once a week	0.872	(0.818, 0.926)	0.028	
	Once a week	0.805	(0.751, 0.858)	0.027	
	One to three times a month	0.836	(0.770, 0.902)	0.033	
Education	9-15	0.822	(0.792, 0.852)	0.015	4.44e-04
	16+	0.879	(0.843, 0.916)	0.019	

Variable	Category	Proportion	95% CI	SE	p-value
	Up to 8	0.755	(0.705, 0.805)	0.025	
Immigration status	Born in this country	0.815	(0.793, 0.838)	0.011	2.04e-09
	Born in another country	0.969	(0.920, 1.000)	0.024	
Religious affiliation	No religion/Atheist/Agnostic	0.773	(0.704, 0.842)	0.035	< 1e-16
	Christianity	0.822	(0.799, 0.846)	0.012	
	Combined	0.988	(0.957, 1.000)	0.015	
	Spiritism	*	*	*	
	Umbanda, Candomblé, and other African-derived religions	*	*	*	
	Buddhism	*	*	*	
	Islam	*	*	*	
	Chinese folk/traditional religion	*	*	*	
	Hinduism	*	*	*	
	Judaism	*	*	*	
	Primal, Animist, or Folk religion	*	*	*	

Table S13d. Mexico: Childhood predictors regression analysis

Variable	Category	Risk-Ratio	logRR SE	RR 95% CI	Global p-value
Relationship with your mother growing up	(Ref: Very bad/somewhat bad)				0.326
	Very good/somewhat good	1.053	0.048	(0.96,1.16)	
Relationship with your father growing up	(Ref: Very bad/somewhat bad)				0.392
	Very good/somewhat good	0.965	0.040	(0.89,1.04)	
Parents married to each other when you were around 12 years old	(Ref: Parents married)				0.099
	One or both of them had died	0.960	0.067	(0.84,1.10)	
	Parents were divorced	0.868	0.061	(0.77,0.98)	
	Parents were never married	0.974	0.035	(0.91,1.04)	
	Unsure	0.951	0.085	(0.80,1.12)	
Feelings about familys household income when growing up	(Ref: Got by)				0.488
	Found it difficult	0.978	0.029	(0.92,1.03)	
	Found it very difficult	1.002	0.043	(0.92,1.09)	
	Lived comfortably	1.037	0.027	(0.98,1.09)	
Physically or sexually abused when growing up	(Ref: No)				0.024
		1.062	0.026	(1.01,1.12)	
Felt like an outsider in your family when growing up	(Ref: No)				0.497
	Yes	1.019	0.031	(0.96,1.08)	
Your health when growing up	(Ref: Good)				0.823
	Excellent	0.994	0.028	(0.94,1.05)	
	Fair	0.987	0.037	(0.92,1.06)	
	Poor	0.993	0.088	(0.83,1.18)	
	Very good	1.004	0.029	(0.95,1.06)	
Born in This	(Ref: Born in this				1.15e-06

Variable	Category	Risk-Ratio	logRR SE	RR 95% CI	Global p-value
country	country)				
	Born in another country	1.169	0.032	(1.10,1.25)	
How Often You Attended Religious Services or Worshiped When You Were Around 12 Years Old	(Ref: Never)				0.051
	At least once a week	1.083	0.033	(1.02,1.15)	
	Less than once a month	1.037	0.038	(0.96,1.12)	
	One to three times a month	0.988	0.041	(0.91,1.07)	
Year of birth (age group)	(Ref: 1998-2005; current age: 18-24)				0.290
	1943 or earlier (current age: 80+ years)	0.829	0.107	(0.67,1.02)	
	1943-1953 (current age: 70-79 years)	0.947	0.054	(0.85,1.05)	
	1953-1963 (current age: 60-69 years)	0.975	0.042	(0.90,1.06)	
	1963-1973 (current age: 50-59 years)	0.955	0.034	(0.89,1.02)	
	1973-1983 (current age: 40-49 years)	0.952	0.036	(0.89,1.02)	
	1983-1993 (current age: 30-39 years)	0.941	0.036	(0.88,1.01)	
	1993-1998 (current age: 25-29 years)	0.960	0.044	(0.88,1.05)	
Religion when twelve years old	(Ref: No religion/Atheist/Agnostic)				0.199
	Christianity	0.930	0.043	(0.85,1.01)	
	Collapsed affiliations with prevalence<3%	0.798	0.238	(0.50,1.27)	
Race plurality (prominent race/ethnic group [0] or not [1])	(Ref: Plurality group)				0.188
	Non-plurality groups	0.969	0.022	(0.93,1.01)	

Table S13e. Mexico: Sensitivity to unmeasured confounding of childhood predictors

Variable	Category	E-value for Estimate	E-value for 95% CI
Relationship with your mother growing up	(Ref: Very bad/somewhat bad)		
	Very good/somewhat good	1.29	1.00
Relationship with your father growing up	(Ref: Very bad/somewhat bad)		
	Very good/somewhat good	1.23	1.00
Parents married to each other when you were around 12 years old	(Ref: Parents married)		
	One or both of them had died	1.25	1.00
	Parents were divorced	1.57	1.17
	Parents were never married	1.19	1.00
	Unsure	1.28	1.00
Feelings about familys household income when growing up	(Ref: Got by)		
	Found it difficult	1.17	1.00
	Found it very difficult	1.05	1.00
	Lived comfortably	1.23	1.00
Physically or sexually abused when growing up	(Ref: No)		
		1.32	1.10
Felt like an outsider in your family when growing up	(Ref: No)		
	Yes	1.16	1.00
Your health when growing up	(Ref: Good)		
	Excellent	1.08	1.00
	Fair	1.13	1.00
	Poor	1.09	1.00
	Very good	1.07	1.00
Born in This country	(Ref: Born in this country)		
	Born in another country	1.61	1.43
How Often You Attended Religious Services or Worshiped When You Were Around 12 Years Old	(Ref: Never)		
	At least once a week	1.38	1.14
	Less than once a month	1.23	1.00
	One to three times a month	1.12	1.00
Year of birth (age group)	(Ref: 1998-2005; current age: 18-24)		
	1943 or earlier (current age: 80+ years)	1.71	1.00
	1943-1953 (current age: 70-79 years)	1.30	1.00

Variable	Category	E-value for Estimate	E-value for 95% CI
	1953-1963 (current age: 60-69 years)	1.19	1.00
	1963-1973 (current age: 50-59 years)	1.27	1.00
	1973-1983 (current age: 40-49 years)	1.28	1.00
	1983-1993 (current age: 30-39 years)	1.32	1.00
	1993-1998 (current age: 25-29 years)	1.25	1.00
Religion when twelve years old	(Ref: No religion/Atheist/Agnostic)		
	Christianity	1.36	1.00
	Collapsed affiliations with prevalence<3%	1.82	1.00
Race plurality (prominent race/ethnic group [0] or not [1])	(Ref: Plurality group)		
	Non-plurality groups	1.21	1.00

Table S14a. Nigeria: Demographic descriptive statistics

**Characteristic**	**N = 4,943**[1]
**Age group**	
1943 or earlier (current age: 80+ years)	36 (0.7%)
1943-1953 (current age: 70-79 years)	93 (1.9%)
1953-1963 (current age: 60-69 years)	195 (3.9%)
1963-1973 (current age: 50-59 years)	412 (8.3%)
1973-1983 (current age: 40-49 years)	703 (14%)
1983-1993 (current age: 30-39 years)	1,379 (28%)
1993-1998 (current age: 25-29 years)	899 (18%)
1998-2005 (current age: 18-24 years)	1,227 (25%)
(Missing)	0 (0%)
**Gender**	
1. Male	2,471 (50%)
2. Female	2,472 (50%)
3. Other	0 (<0.1%)
(Missing)	0 (0%)
**Marital status**	
1. Single/Never been married	1,757 (36%)
2. Married	2,858 (58%)
3. Separated	89 (1.8%)
4. Divorced	51 (1.0%)
5. Widowed	155 (3.1%)
6. Domestic partner	7 (0.2%)
(Missing)	26 (0.5%)
**Employment**	
1. Employed for an employer	496 (10%)
2. Self-employed	2,770 (56%)
3. Retired	112 (2.3%)
4. Student	512 (10%)
5. Homemaker	370 (7.5%)
6. Unemployed and looking for a job	504 (10%)
7. None of these/Other	173 (3.5%)
(Missing)	6 (0.1%)
**Religious service attendance**	
1. More than once a week	2,967 (60%)
2. Once a week	1,344 (27%)
3. One to three times a month	378 (7.6%)
4. A few times a year	178 (3.6%)
5. Never	64 (1.3%)
(Missing)	12 (0.3%)
**Education**	
1. Up to 8	1,858 (38%)
2. 9-15	2,998 (61%)
3. 16+	86 (1.7%)
(Missing)	2 (<0.1%)
**Immigration**	
1. Born in this country	4,909 (99%)
2. Born in another country	33 (0.7%)

Characteristic	N = 4,943[1]
(Missing)	0 (<0.1%)
**Religious affiliation**	
1. Christianity	2,476 (50%)
10. Taoism	0 (0%)
11. Confucianism	0 (<0.1%)
12. Primal, Animist, or Folk religion	23 (0.5%)
13. Spiritism	0 (0%)
14. Umbanda, Candomblé, and other African-derived religions	0 (0%)
15. Chinese folk/traditional religion	0 (0%)
2. Islam	2,425 (49%)
3. Hinduism	0 (0%)
4. Buddhism	0 (0%)
5. Judaism	0 (0%)
6. Sikhism	0 (0%)
7. Baha'i	0 (0%)
8. Jainism	0 (0%)
9. Shinto	1 (<0.1%)
96. Some other religion	0 (0%)
97. No religion/Atheist/Agnostic	12 (0.2%)
(Missing)	7 (0.1%)
**RACE ETHNICITY**	
(Missing)	2 (<0.1%)
1201. Nigeria: Hausa	1,730 (35%)
1202. Nigeria: Yoruba	874 (18%)
1203. Nigeria: Igbo (Ibo)	774 (16%)
1204. Nigeria: Edo	75 (1.5%)
1205. Nigeria: Urhobo	34 (0.7%)
1206. Nigeria: Fulani	188 (3.8%)
1207. Nigeria: Kanuri	27 (0.5%)
1208. Nigeria: Tiv	140 (2.8%)
1209. Nigeria: Efik	37 (0.7%)
1210. Nigeria: Ijaw	79 (1.6%)
1211. Nigeria: Igala	62 (1.3%)
1212. Nigeria: Ibibio	135 (2.7%)
1213. Nigeria: Idoma	46 (0.9%)
9996. Other	739 (15%)

[1]n (%)

Table S14b. Nigeria: Childhood descriptive statistics

Characteristic	N = 4,943[1]
**Age**	35 (14)
**Year of birth**	
1943 or earlier (current age: 80+ years)	36 (0.7%)
1943-1953 (current age: 70-79 years)	93 (1.9%)
1953-1963 (current age: 60-69 years)	195 (3.9%)
1963-1973 (current age: 50-59 years)	412 (8.3%)
1973-1983 (current age: 40-49 years)	703 (14%)
1983-1993 (current age: 30-39 years)	1,379 (28%)
1993-1998 (current age: 25-29 years)	899 (18%)
1998-2005 (current age: 18-24 years)	1,227 (25%)
(Missing)	0 (0%)
**Gender**	
1. Male	2,471 (50%)
2. Female	2,472 (50%)
3. Other	0 (<0.1%)
(Missing)	0 (0%)
**Parent marital status**	
1. Parents were married	4,061 (82%)
2. Parents were divorced	212 (4.3%)
3. Parents were never married	251 (5.1%)
4. One or both of them had died	326 (6.6%)
5. Unsure	39 (0.8%)
(Missing)	54 (1.1%)
**Age 12 religious service attendance**	
1. At least once a week	4,301 (87%)
2. One to three times a month	423 (8.6%)
3. Less than once a month	88 (1.8%)
4. Never	95 (1.9%)
(Missing)	36 (0.7%)
**Relationship with mother**	
1. Very good	4,315 (87%)
2. Somewhat good	483 (9.8%)
3. Somewhat bad	47 (1.0%)
4. Very bad	13 (0.3%)
97. (Does not apply)	74 (1.5%)
(Missing)	9 (0.2%)
**Relationship with father**	
1. Very good	4,020 (81%)
2. Somewhat good	679 (14%)
3. Somewhat bad	60 (1.2%)
4. Very bad	30 (0.6%)
97. (Does not apply)	136 (2.7%)
(Missing)	19 (0.4%)
**Outsider growing up**	
1. Yes	503 (10%)
2. No	4,369 (88%)
(Missing)	71 (1.4%)

Characteristic	N = 4,943[1]
**Abuse**	
1. Yes	631 (13%)
2. No	4,251 (86%)
(Missing)	62 (1.2%)
**Self-rated health growing up**	
1. Excellent	1,926 (39%)
2. Very good	1,890 (38%)
3. Good	842 (17%)
4. Fair	193 (3.9%)
5. Poor	83 (1.7%)
(Missing)	10 (0.2%)
**Immigration status**	
1. Born in this country	4,909 (99%)
2. Born in another country	33 (0.7%)
(Missing)	0 (<0.1%)
**Subjective financial status of family growing up**	
1. Lived comfortably	1,622 (33%)
2. Got by	1,724 (35%)
3. Found it difficult	1,168 (24%)
4. Found it very difficult	405 (8.2%)
(Missing)	24 (0.5%)
**Religious affiliation**	
1. Christianity	2,476 (50%)
10. Taoism	0 (0%)
11. Confucianism	1 (<0.1%)
12. Primal, Animist, or Folk religion	8 (0.2%)
13. Spiritism	0 (0%)
14. Umbanda, Candomblé, and other African-derived religions	0 (0%)
15. Chinese folk/traditional religion	0 (0%)
2. Islam	2,436 (49%)
3. Hinduism	0 (0%)
4. Buddhism	0 (0%)
5. Judaism	0 (0%)
6. Sikhism	0 (0%)
7. Baha'i	0 (0%)
8. Jainism	0 (0%)
9. Shinto	0 (0%)
96. Some other religion	0 (0%)
97. No religion/Atheist/Agnostic	15 (0.3%)
(Missing)	8 (0.2%)
**Race/Ethnicity**	
(Missing)	2 (<0.1%)
1201. Nigeria: Hausa	1,730 (35%)
1202. Nigeria: Yoruba	874 (18%)
1203. Nigeria: Igbo (Ibo)	774 (16%)
1204. Nigeria: Edo	75 (1.5%)
1205. Nigeria: Urhobo	34 (0.7%)

Characteristic	N = 4,943[1]
1206. Nigeria: Fulani	188 (3.8%)
1207. Nigeria: Kanuri	27 (0.5%)
1208. Nigeria: Tiv	140 (2.8%)
1209. Nigeria: Efik	37 (0.7%)
1210. Nigeria: Ijaw	79 (1.6%)
1211. Nigeria: Igala	62 (1.3%)
1212. Nigeria: Ibibio	135 (2.7%)
1213. Nigeria: Idoma	46 (0.9%)
9996. Other	739 (15%)

[1]Mean (SD); n (%)

Table S14c. Nigeria: Proportions by demographic category

Variable	Category	Proportion	95% CI	SE	p-value
Age group	1998-2005 (current age: 18-24 years)	0.904	(0.875, 0.932)	0.015	1.34e-01
	1943 or earlier (current age: 80+ years)	0.972	(0.909, 1.000)	0.030	
	1943-1953 (current age: 70-79 years)	0.871	(0.760, 0.983)	0.056	
	1953-1963 (current age: 60-69 years)	0.793	(0.692, 0.894)	0.051	
	1963-1973 (current age: 50-59 years)	0.908	(0.855, 0.961)	0.027	
	1973-1983 (current age: 40-49 years)	0.906	(0.873, 0.940)	0.017	
	1983-1993 (current age: 30-39 years)	0.899	(0.878, 0.920)	0.010	
	1993-1998 (current age: 25-29 years)	0.903	(0.874, 0.931)	0.015	
Gender	Male	0.903	(0.883, 0.924)	0.011	< 1e-16
	Female	0.893	(0.873, 0.914)	0.010	
	Other	1.000	*	*	
Marital status	Single/Never been married	0.909	(0.890, 0.929)	0.010	< 1e-16
	Divorced	0.889	(0.781, 0.996)	0.053	
	Domestic partner	0.952	*	*	
	Married	0.893	(0.872, 0.915)	0.011	
	Separated	0.943	(0.879, 1.000)	0.032	
	Widowed	0.844	(0.759, 0.929)	0.043	
Employment	Employed for an employer	0.943	(0.920, 0.967)	0.012	4.18e-02
	Homemaker	0.905	(0.854, 0.957)	0.026	
	None of these/Other	0.857	(0.762, 0.951)	0.048	
	Retired	0.875	(0.770, 0.980)	0.053	
	Self-employed	0.893	(0.873, 0.914)	0.010	
	Student	0.894	(0.851, 0.936)	0.022	
	Unemployed and looking for a job	0.901	(0.863, 0.939)	0.019	
Religious service attendance	Never	0.899	(0.785, 1.000)	0.057	9.12e-01
	A few times a year	0.885	(0.818, 0.952)	0.034	
	More than once a week	0.902	(0.881, 0.923)	0.011	
	Once a week	0.891	(0.868, 0.915)	0.012	
	One to three times a month	0.903	(0.858, 0.947)	0.023	
Education	9-15	0.910	(0.896, 0.924)	0.007	5.98e-03
	16+	0.949	(0.918, 0.980)	0.016	

Variable	Category	Proportion	95% CI	SE	p-value
	Up to 8	0.878	(0.846, 0.909)	0.016	
Immigration status	Born in this country	0.898	(0.882, 0.914)	0.008	1.89e-01
	Born in another country	0.941	(0.878, 1.000)	0.031	
Religious affiliation	No religion/Atheist/Agnostic	*	*	*	3.61e-02
	Christianity	0.917	(0.902, 0.932)	0.008	
	Combined	0.947	(0.879, 1.000)	0.033	
	Spiritism	*	*	*	
	Umbanda, Candomblé, and other African-derived religions	*	*	*	
	Buddhism	*	*	*	
	Islam	0.879	(0.851, 0.907)	0.014	
	Chinese folk/traditional religion	*	*	*	
	Hinduism	*	*	*	
	Judaism	*	*	*	
	Primal, Animist, or Folk religion	*	*	*	

Table S14d. Nigeria: Childhood predictors regression analysis

Variable	Category	Risk-Ratio	logRR SE	RR 95% CI	Global p-value
Relationship with your mother growing up	(Ref: Very bad/somewhat bad)				0.533
	Very good/somewhat good	0.987	0.030	(0.93,1.05)	
Relationship with your father growing up	(Ref: Very bad/somewhat bad)				0.024
	Very good/somewhat good	0.942	0.026	(0.90,0.99)	
Parents married to each other when you were around 12 years old	(Ref: Parents married)				0.701
	One or both of them had died	1.019	0.024	(0.97,1.07)	
	Parents were divorced	1.023	0.025	(0.97,1.07)	
	Parents were never married	1.006	0.025	(0.96,1.06)	
	Unsure	1.010	0.036	(0.94,1.08)	
Feelings about familys household income when growing up	(Ref: Got by)				0.648
	Found it difficult	0.989	0.015	(0.96,1.02)	
	Found it very difficult	1.008	0.024	(0.96,1.06)	
	Lived comfortably	0.990	0.015	(0.96,1.02)	
Physically or sexually abused when growing up	(Ref: No)				0.127
		1.024	0.015	(1.00,1.05)	
Felt like an outsider in your family when growing up	(Ref: No)				0.486
	Yes	0.985	0.023	(0.94,1.03)	
Your health when growing up	(Ref: Good)				0.726
	Excellent	1.020	0.019	(0.98,1.06)	
	Fair	0.987	0.037	(0.92,1.06)	
	Poor	1.007	0.060	(0.90,1.13)	
	Very good	1.004	0.019	(0.97,1.04)	
Born in This	(Ref: Born in this				0.267

Variable	Category	Risk-Ratio	logRR SE	RR 95% CI	Global p-value
country	country)				
	Born in another country	1.053	0.042	(0.97,1.14)	
How Often You Attended Religious Services or Worshiped When You Were Around 12 Years Old	(Ref: Never)				0.472
	At least once a week	0.976	0.044	(0.90,1.06)	
	Less than once a month	0.929	0.064	(0.82,1.05)	
	One to three times a month	0.944	0.053	(0.85,1.05)	
Year of birth (age group)	(Ref: 1998-2005; current age: 18-24)				0.141
	1943 or earlier (current age: 80+ years)	1.041	0.034	(0.97,1.11)	
	1943-1953 (current age: 70-79 years)	0.952	0.055	(0.85,1.06)	
	1953-1963 (current age: 60-69 years)	0.884	0.051	(0.80,0.98)	
	1963-1973 (current age: 50-59 years)	0.994	0.029	(0.94,1.05)	
	1973-1983 (current age: 40-49 years)	0.995	0.020	(0.96,1.03)	
	1983-1993 (current age: 30-39 years)	0.993	0.017	(0.96,1.03)	
	1993-1998 (current age: 25-29 years)	1.000	0.019	(0.96,1.04)	
Religion when twelve years old	(Ref: Christianity)				0.355
	Collapsed affiliations with prevalence<3%	1.042	0.031	(0.98,1.11)	
	Islam	0.984	0.020	(0.95,1.02)	
Race plurality (prominent race/ethnic group [0] or not [1])	(Ref: Plurality group)				0.166
	Non-plurality groups	1.034	0.022	(0.99,1.08)	

Table S14e. Nigeria: Sensitivity to unmeasured confounding of childhood predictors

Variable	Category	E-value for Estimate	E-value for 95% CI
Relationship with your mother growing up	(Ref: Very bad/somewhat bad)		
	Very good/somewhat good	1.13	1.00
Relationship with your father growing up	(Ref: Very bad/somewhat bad)		
	Very good/somewhat good	1.32	1.10
Parents married to each other when you were around 12 years old	(Ref: Parents married)		
	One or both of them had died	1.16	1.00
	Parents were divorced	1.18	1.00
	Parents were never married	1.09	1.00
	Unsure	1.11	1.00
Feelings about familys household income when growing up	(Ref: Got by)		
	Found it difficult	1.12	1.00
	Found it very difficult	1.10	1.00
	Lived comfortably	1.11	1.00
Physically or sexually abused when growing up	(Ref: No)		
		1.18	1.00
Felt like an outsider in your family when growing up	(Ref: No)		
	Yes	1.14	1.00
Your health when growing up	(Ref: Good)		
	Excellent	1.16	1.00
	Fair	1.13	1.00
	Poor	1.09	1.00
	Very good	1.07	1.00
Born in This country	(Ref: Born in this country)		
	Born in another country	1.29	1.00
How Often You Attended Religious Services or Worshiped When You Were Around 12 Years Old	(Ref: Never)		
	At least once a week	1.18	1.00
	Less than once a month	1.36	1.00
	One to three times a month	1.31	1.00
Year of birth (age group)	(Ref: 1998-2005; current age: 18-24)		
	1943 or earlier (current age: 80+ years)	1.25	1.00
	1943-1953 (current age: 70-79 years)	1.28	1.00

Variable	Category	E-value for Estimate	E-value for 95% CI
	1953-1963 (current age: 60-69 years)	1.52	1.18
	1963-1973 (current age: 50-59 years)	1.08	1.00
	1973-1983 (current age: 40-49 years)	1.07	1.00
	1983-1993 (current age: 30-39 years)	1.09	1.00
	1993-1998 (current age: 25-29 years)	1.02	1.00
Religion when twelve years old	(Ref: Christianity)		
	Collapsed affiliations with prevalence<3%	1.25	1.00
	Islam	1.14	1.00
Race plurality (prominent race/ethnic group [0] or not [1])	(Ref: Plurality group)		
	Non-plurality groups	1.22	1.00

Table S15a. Philippines: Demographic descriptive statistics

**Characteristic**	**N = 3,448**[1]
**Age group**	
1943 or earlier (current age: 80+ years)	14 (0.4%)
1943-1953 (current age: 70-79 years)	87 (2.5%)
1953-1963 (current age: 60-69 years)	309 (9.0%)
1963-1973 (current age: 50-59 years)	454 (13%)
1973-1983 (current age: 40-49 years)	618 (18%)
1983-1993 (current age: 30-39 years)	773 (22%)
1993-1998 (current age: 25-29 years)	424 (12%)
1998-2005 (current age: 18-24 years)	770 (22%)
(Missing)	0 (0%)
**Gender**	
1. Male	1,651 (48%)
2. Female	1,786 (52%)
3. Other	8 (0.2%)
(Missing)	3 (<0.1%)
**Marital status**	
1. Single/Never been married	827 (24%)
2. Married	1,526 (44%)
3. Separated	165 (4.8%)
4. Divorced	7 (0.2%)
5. Widowed	166 (4.8%)
6. Domestic partner	747 (22%)
(Missing)	10 (0.3%)
**Employment**	
1. Employed for an employer	877 (25%)
2. Self-employed	842 (24%)
3. Retired	100 (2.9%)
4. Student	415 (12%)
5. Homemaker	714 (21%)
6. Unemployed and looking for a job	428 (12%)
7. None of these/Other	72 (2.1%)
(Missing)	0 (0%)
**Religious service attendance**	
1. More than once a week	550 (16%)
2. Once a week	1,270 (37%)
3. One to three times a month	881 (26%)
4. A few times a year	609 (18%)
5. Never	137 (4.0%)
(Missing)	0 (0%)
**Education**	
1. Up to 8	824 (24%)
2. 9-15	2,404 (70%)
3. 16+	219 (6.4%)
(Missing)	0 (0%)
**Immigration**	
1. Born in this country	3,445 (100%)
2. Born in another country	3 (<0.1%)

Characteristic	N = 3,448[1]
(Missing)	0 (0%)
**Religious affiliation**	
1. Christianity	3,170 (92%)
10. Taoism	0 (0%)
11. Confucianism	0 (0%)
12. Primal, Animist, or Folk religion	1 (<0.1%)
13. Spiritism	0 (0%)
14. Umbanda, Candomblé, and other African-derived religions	0 (0%)
15. Chinese folk/traditional religion	0 (0%)
2. Islam	224 (6.5%)
3. Hinduism	0 (0%)
4. Buddhism	3 (<0.1%)
5. Judaism	4 (0.1%)
6. Sikhism	0 (0%)
7. Baha'i	1 (<0.1%)
8. Jainism	0 (0%)
9. Shinto	0 (0%)
96. Some other religion	19 (0.5%)
97. No religion/Atheist/Agnostic	19 (0.5%)
(Missing)	6 (0.2%)
**RACE ETHNICITY**	
(Missing)	1 (<0.1%)
1301. Philippines: Tagalog	1,111 (32%)
1302. Philippines: Cebuano	413 (12%)
1303. Philippines: Ilocano/Ilokano	299 (8.7%)
1304. Philippines: Visayan/Bisaya	430 (12%)
1305. Philippines: Ilonggo/Hiligaynon	289 (8.4%)
1306. Philippines: Bicolano/Bikolano	191 (5.5%)
1307. Philippines: Waray	151 (4.4%)
1308. Philippines: Tausug	69 (2.0%)
1309. Philippines: Maranao	34 (1.0%)
1310. Philippines: Maguindanaoan	65 (1.9%)
1311. Philippines: Chinese-Filipino	4 (0.1%)
1312. Philippines: Kapampangan	70 (2.0%)
1313. Philippines: Pangasinense	69 (2.0%)
1314. Philippines: Zamboangueno	30 (0.9%)
1316. Philippines: Masbateno	32 (0.9%)
1317. Philippines: Aeta	0 (<0.1%)
1318. Philippines: Igorot	29 (0.8%)
1320. Philippines: Badjao	1 (<0.1%)
9996. Other	160 (4.6%)

[1]n (%)

Table S15b. Philippines: Childhood descriptive statistics

Characteristic	N = 3,448[1]
**Age**	39 (15)
**Year of birth**	
1943 or earlier (current age: 80+ years)	14 (0.4%)
1943-1953 (current age: 70-79 years)	87 (2.5%)
1953-1963 (current age: 60-69 years)	309 (9.0%)
1963-1973 (current age: 50-59 years)	454 (13%)
1973-1983 (current age: 40-49 years)	618 (18%)
1983-1993 (current age: 30-39 years)	773 (22%)
1993-1998 (current age: 25-29 years)	424 (12%)
1998-2005 (current age: 18-24 years)	770 (22%)
(Missing)	0 (0%)
**Gender**	
1. Male	1,651 (48%)
2. Female	1,786 (52%)
3. Other	8 (0.2%)
(Missing)	3 (<0.1%)
**Parent marital status**	
1. Parents were married	2,999 (87%)
2. Parents were divorced	37 (1.1%)
3. Parents were never married	336 (9.7%)
4. One or both of them had died	35 (1.0%)
5. Unsure	25 (0.7%)
(Missing)	17 (0.5%)
**Age 12 religious service attendance**	
1. At least once a week	1,602 (46%)
2. One to three times a month	1,101 (32%)
3. Less than once a month	572 (17%)
4. Never	137 (4.0%)
(Missing)	37 (1.1%)
**Relationship with mother**	
1. Very good	2,166 (63%)
2. Somewhat good	1,114 (32%)
3. Somewhat bad	86 (2.5%)
4. Very bad	25 (0.7%)
97. (Does not apply)	36 (1.0%)
(Missing)	21 (0.6%)
**Relationship with father**	
1. Very good	2,234 (65%)
2. Somewhat good	916 (27%)
3. Somewhat bad	111 (3.2%)
4. Very bad	39 (1.1%)
97. (Does not apply)	77 (2.2%)
(Missing)	70 (2.0%)
**Outsider growing up**	
1. Yes	262 (7.6%)
2. No	3,177 (92%)
(Missing)	9 (0.3%)

Characteristic	N = 3,448[1]
**Abuse**	
1. Yes	284 (8.2%)
2. No	3,163 (92%)
(Missing)	0 (<0.1%)
**Self-rated health growing up**	
1. Excellent	644 (19%)
2. Very good	359 (10%)
3. Good	1,448 (42%)
4. Fair	817 (24%)
5. Poor	179 (5.2%)
(Missing)	0 (0%)
**Immigration status**	
1. Born in this country	3,445 (100%)
2. Born in another country	3 (<0.1%)
(Missing)	0 (0%)
**Subjective financial status of family growing up**	
1. Lived comfortably	601 (17%)
2. Got by	1,929 (56%)
3. Found it difficult	711 (21%)
4. Found it very difficult	206 (6.0%)
(Missing)	1 (<0.1%)
**Religious affiliation**	
1. Christianity	3,211 (93%)
10. Taoism	0 (0%)
11. Confucianism	0 (0%)
12. Primal, Animist, or Folk religion	3 (<0.1%)
13. Spiritism	0 (0%)
14. Umbanda, Candomblé, and other African-derived religions	0 (0%)
15. Chinese folk/traditional religion	0 (0%)
2. Islam	206 (6.0%)
3. Hinduism	0 (0%)
4. Buddhism	1 (<0.1%)
5. Judaism	0 (0%)
6. Sikhism	4 (0.1%)
7. Baha'i	1 (<0.1%)
8. Jainism	0 (0%)
9. Shinto	0 (0%)
96. Some other religion	6 (0.2%)
97. No religion/Atheist/Agnostic	6 (0.2%)
(Missing)	10 (0.3%)
**Race/Ethnicity**	
(Missing)	1 (<0.1%)
1301. Philippines: Tagalog	1,111 (32%)
1302. Philippines: Cebuano	413 (12%)
1303. Philippines: Ilocano/Ilokano	299 (8.7%)
1304. Philippines: Visayan/Bisaya	430 (12%)
1305. Philippines: Ilonggo/Hiligaynon	289 (8.4%)

Characteristic	N = 3,448[1]
1306. Philippines: Bicolano/Bikolano	191 (5.5%)
1307. Philippines: Waray	151 (4.4%)
1308. Philippines: Tausug	69 (2.0%)
1309. Philippines: Maranao	34 (1.0%)
1310. Philippines: Maguindanaoan	65 (1.9%)
1311. Philippines: Chinese-Filipino	4 (0.1%)
1312. Philippines: Kapampangan	70 (2.0%)
1313. Philippines: Pangasinense	69 (2.0%)
1314. Philippines: Zamboangueno	30 (0.9%)
1316. Philippines: Masbateno	32 (0.9%)
1317. Philippines: Aeta	0 (<0.1%)
1318. Philippines: Igorot	29 (0.8%)
1320. Philippines: Badjao	1 (<0.1%)
9996. Other	160 (4.6%)

[1]Mean (SD); n (%)

Table S15c. Philippines: Proportions by demographic category

Variable	Category	Proportion	95% CI	SE	p-value
Age group	1998-2005 (current age: 18-24 years)	0.903	(0.874, 0.932)	0.015	4.26e-07
	1943 or earlier (current age: 80+ years)	0.525	(0.157, 0.894)	0.136	
	1943-1953 (current age: 70-79 years)	0.807	(0.713, 0.900)	0.047	
	1953-1963 (current age: 60-69 years)	0.816	(0.762, 0.870)	0.028	
	1963-1973 (current age: 50-59 years)	0.770	(0.728, 0.812)	0.021	
	1973-1983 (current age: 40-49 years)	0.840	(0.808, 0.872)	0.016	
	1983-1993 (current age: 30-39 years)	0.859	(0.833, 0.885)	0.013	
	1993-1998 (current age: 25-29 years)	0.898	(0.859, 0.938)	0.020	
Gender	Male	0.842	(0.821, 0.863)	0.011	4.00e-01
	Female	0.861	(0.845, 0.878)	0.009	
	Other	0.836	(0.469, 1.000)	0.116	
Marital status	Single/Never been married	0.881	(0.850, 0.912)	0.016	< 1e-16
	Divorced	1.000	*	*	
	Domestic partner	0.894	(0.871, 0.917)	0.012	
	Married	0.828	(0.806, 0.849)	0.011	
	Separated	0.771	(0.698, 0.844)	0.037	
	Widowed	0.817	(0.758, 0.877)	0.030	
Employment	Employed for an employer	0.849	(0.822, 0.876)	0.014	5.68e-03
	Homemaker	0.831	(0.803, 0.859)	0.014	
	None of these/Other	0.808	(0.695, 0.920)	0.056	
	Retired	0.832	(0.736, 0.927)	0.048	
	Self-employed	0.857	(0.829, 0.884)	0.014	
	Student	0.920	(0.886, 0.955)	0.017	
	Unemployed and looking for a job	0.830	(0.782, 0.879)	0.025	
Religious service attendance	Never	0.850	(0.773, 0.927)	0.039	5.93e-01
	A few times a year	0.838	(0.803, 0.873)	0.018	
	More than once a week	0.833	(0.798, 0.868)	0.018	
	Once a week	0.862	(0.841, 0.884)	0.011	
	One to three times a month	0.859	(0.832, 0.886)	0.014	
Education	9-15	0.869	(0.853, 0.884)	0.008	5.69e-05
	16+	0.895	(0.851, 0.938)	0.022	

Variable	Category	Proportion	95% CI	SE	p-value
	Up to 8	0.792	(0.760, 0.824)	0.016	
Immigration status	Born in this country	0.852	(0.839, 0.865)	0.007	6.19e-01
	Born in another country	0.726	*	*	
Religious affiliation	No religion/Atheist/Agnostic	*	*	*	3.28e-03
	Christianity	0.851	(0.837, 0.865)	0.007	
	Combined	0.952	(0.894, 1.000)	0.029	
	Spiritism	*	*	*	
	Umbanda, Candomblé, and other African-derived religions	*	*	*	
	Buddhism	*	*	*	
	Islam	0.849	(0.804, 0.893)	0.023	
	Chinese folk/traditional religion	*	*	*	
	Hinduism	*	*	*	
	Judaism	*	*	*	
	Primal, Animist, or Folk religion	*	*	*	

Table S15d. Philippines: Childhood predictors regression analysis

Variable	Category	Risk-Ratio	logRR SE	RR 95% CI	Global p-value
Relationship with your mother growing up	(Ref: Very bad/somewhat bad)				0.378
	Very good/somewhat good	1.038	0.039	(0.96,1.12)	
Relationship with your father growing up	(Ref: Very bad/somewhat bad)				0.187
	Very good/somewhat good	1.047	0.032	(0.98,1.11)	
Parents married to each other when you were around 12 years old	(Ref: Parents married)				6.25e-06
	One or both of them had died	0.928	0.073	(0.80,1.07)	
	Parents were divorced	0.927	0.082	(0.79,1.09)	
	Parents were never married	1.030	0.021	(0.99,1.07)	
	Unsure	1.117	0.023	(1.07,1.17)	
Feelings about familys household income when growing up	(Ref: Got by)				0.325
	Found it difficult	0.965	0.022	(0.93,1.01)	
	Found it very difficult	0.971	0.033	(0.91,1.04)	
	Lived comfortably	1.011	0.019	(0.98,1.05)	
Physically or sexually abused when growing up	(Ref: No)				0.380
		0.972	0.031	(0.92,1.03)	
Felt like an outsider in your family when growing up	(Ref: No)				0.043
	Yes	1.048	0.022	(1.00,1.09)	
Your health when growing up	(Ref: Good)				0.076
	Excellent	1.020	0.018	(0.98,1.06)	
	Fair	0.964	0.020	(0.93,1.00)	
	Poor	0.911	0.041	(0.84,0.99)	
	Very good	1.022	0.022	(0.98,1.07)	
Born in This	(Ref: Born in this				0.446

Variable	Category	Risk-Ratio	logRR SE	RR 95% CI	Global p-value
country	country)				
	Born in another country	0.824	0.256	(0.50,1.36)	
How Often You Attended Religious Services or Worshiped When You Were Around 12 Years Old	(Ref: Never)				0.324
	At least once a week	0.981	0.035	(0.92,1.05)	
	Less than once a month	0.939	0.038	(0.87,1.01)	
	One to three times a month	0.976	0.034	(0.91,1.04)	
Year of birth (age group)	(Ref: 1998-2005; current age: 18-24)				3.27e-04
	1943 or earlier (current age: 80+ years)	0.707	0.132	(0.55,0.92)	
	1943-1953 (current age: 70-79 years)	0.920	0.048	(0.84,1.01)	
	1953-1963 (current age: 60-69 years)	0.935	0.030	(0.88,0.99)	
	1963-1973 (current age: 50-59 years)	0.894	0.027	(0.85,0.94)	
	1973-1983 (current age: 40-49 years)	0.953	0.023	(0.91,1.00)	
	1983-1993 (current age: 30-39 years)	0.966	0.021	(0.93,1.01)	
	1993-1998 (current age: 25-29 years)	0.996	0.025	(0.95,1.04)	
Religion when twelve years old	(Ref: Christianity)				7.55e-15
	Collapsed affiliations with prevalence<3%	1.185	0.021	(1.14,1.24)	
	Islam	1.001	0.025	(0.95,1.05)	
Race plurality (prominent race/ethnic group [0] or not [1])	(Ref: Plurality group)				0.139
	Non-plurality groups	0.975	0.016	(0.95,1.01)	

Table S15e. Philippines: Sensitivity to unmeasured confounding of childhood predictors

Variable	Category	E-value for Estimate	E-value for 95% CI
Relationship with your mother growing up	(Ref: Very bad/somewhat bad)		
	Very good/somewhat good	1.24	1.00
Relationship with your father growing up	(Ref: Very bad/somewhat bad)		
	Very good/somewhat good	1.27	1.00
Parents married to each other when you were around 12 years old	(Ref: Parents married)		
	One or both of them had died	1.37	1.00
	Parents were divorced	1.37	1.00
	Parents were never married	1.21	1.00
	Unsure	1.48	1.34
Feelings about familys household income when growing up	(Ref: Got by)		
	Found it difficult	1.23	1.00
	Found it very difficult	1.20	1.00
	Lived comfortably	1.12	1.00
Physically or sexually abused when growing up	(Ref: No)		
		1.20	1.00
Felt like an outsider in your family when growing up	(Ref: No)		
	Yes	1.27	1.05
Your health when growing up	(Ref: Good)		
	Excellent	1.16	1.00
	Fair	1.24	1.00
	Poor	1.43	1.13
	Very good	1.17	1.00
Born in This country	(Ref: Born in this country)		
	Born in another country	1.72	1.00
How Often You Attended Religious Services or Worshiped When You Were Around 12 Years Old	(Ref: Never)		
	At least once a week	1.16	1.00
	Less than once a month	1.33	1.00
	One to three times a month	1.18	1.00
Year of birth (age group)	(Ref: 1998-2005; current age: 18-24)		
	1943 or earlier (current age: 80+ years)	2.18	1.41
	1943-1953 (current age: 70-79 years)	1.39	1.00

Variable	Category	E-value for Estimate	E-value for 95% CI
	1953-1963 (current age: 60-69 years)	1.34	1.10
	1963-1973 (current age: 50-59 years)	1.48	1.31
	1973-1983 (current age: 40-49 years)	1.28	1.06
	1983-1993 (current age: 30-39 years)	1.22	1.00
	1993-1998 (current age: 25-29 years)	1.07	1.00
Religion when twelve years old	(Ref: Christianity)		
	Collapsed affiliations with prevalence<3%	1.65	1.53
	Islam	1.03	1.00
Race plurality (prominent race/ethnic group [0] or not [1])	(Ref: Plurality group)		
	Non-plurality groups	1.19	1.00

Table S16a. Poland: Demographic descriptive statistics

Characteristic	N = 4,316[1]
**Age group**	
1943 or earlier (current age: 80+ years)	44 (1.0%)
1943-1953 (current age: 70-79 years)	319 (7.4%)
1953-1963 (current age: 60-69 years)	859 (20%)
1963-1973 (current age: 50-59 years)	654 (15%)
1973-1983 (current age: 40-49 years)	792 (18%)
1983-1993 (current age: 30-39 years)	862 (20%)
1993-1998 (current age: 25-29 years)	330 (7.7%)
1998-2005 (current age: 18-24 years)	455 (11%)
(Missing)	0 (<0.1%)
**Gender**	
1. Male	2,024 (47%)
2. Female	2,276 (53%)
3. Other	4 (<0.1%)
(Missing)	11 (0.3%)
**Marital status**	
1. Single/Never been married	849 (20%)
2. Married	2,572 (60%)
3. Separated	32 (0.7%)
4. Divorced	205 (4.7%)
5. Widowed	400 (9.3%)
6. Domestic partner	204 (4.7%)
(Missing)	55 (1.3%)
**Employment**	
1. Employed for an employer	2,397 (56%)
2. Self-employed	232 (5.4%)
3. Retired	1,074 (25%)
4. Student	244 (5.7%)
5. Homemaker	165 (3.8%)
6. Unemployed and looking for a job	144 (3.3%)
7. None of these/Other	48 (1.1%)
(Missing)	12 (0.3%)
**Religious service attendance**	
1. More than once a week	122 (2.8%)
2. Once a week	1,578 (37%)
3. One to three times a month	895 (21%)
4. A few times a year	1,079 (25%)
5. Never	622 (14%)
(Missing)	20 (0.5%)
**Education**	
1. Up to 8	328 (7.6%)
2. 9-15	2,777 (64%)
3. 16+	1,211 (28%)
(Missing)	0 (0%)
**Immigration**	
1. Born in this country	4,287 (99%)
2. Born in another country	27 (0.6%)

**Characteristic**	**N = 4,316**[1]
(Missing)	1 (<0.1%)
**Religious affiliation**	
1. Christianity	3,867 (90%)
10. Taoism	0 (0%)
11. Confucianism	0 (0%)
12. Primal, Animist, or Folk religion	2 (<0.1%)
13. Spiritism	0 (0%)
14. Umbanda, Candomblé, and other African-derived religions	0 (0%)
15. Chinese folk/traditional religion	0 (0%)
2. Islam	1 (<0.1%)
3. Hinduism	0 (0%)
4. Buddhism	1 (<0.1%)
5. Judaism	0 (0%)
6. Sikhism	1 (<0.1%)
7. Baha'i	0 (0%)
8. Jainism	0 (0%)
9. Shinto	0 (0%)
96. Some other religion	0 (0%)
97. No religion/Atheist/Agnostic	426 (9.9%)
(Missing)	17 (0.4%)
**RACE ETHNICITY**	
(Missing)	2 (<0.1%)
1401. Poland: Polish	4,295 (100%)
1402. Poland: German	1 (<0.1%)
1403. Poland: Belarussian	2 (<0.1%)
1404. Poland: Ukrainian	7 (0.2%)
1409. Poland: Silesia	7 (0.2%)
9996. Other	1 (<0.1%)

[1]n (%)

Table S16b. Poland: Childhood descriptive statistics

**Characteristic**	**N = 4,316**[1]
**Age**	47 (17)
**Year of birth**	
1943 or earlier (current age: 80+ years)	44 (1.0%)
1943-1953 (current age: 70-79 years)	319 (7.4%)
1953-1963 (current age: 60-69 years)	859 (20%)
1963-1973 (current age: 50-59 years)	654 (15%)
1973-1983 (current age: 40-49 years)	792 (18%)
1983-1993 (current age: 30-39 years)	862 (20%)
1993-1998 (current age: 25-29 years)	330 (7.7%)
1998-2005 (current age: 18-24 years)	455 (11%)
(Missing)	0 (<0.1%)
**Gender**	
1. Male	2,024 (47%)
2. Female	2,276 (53%)
3. Other	4 (<0.1%)
(Missing)	11 (0.3%)
**Parent marital status**	
1. Parents were married	3,915 (91%)
2. Parents were divorced	219 (5.1%)
3. Parents were never married	54 (1.3%)
4. One or both of them had died	86 (2.0%)
5. Unsure	35 (0.8%)
(Missing)	7 (0.2%)
**Age 12 religious service attendance**	
1. At least once a week	2,108 (49%)
2. One to three times a month	1,106 (26%)
3. Less than once a month	796 (18%)
4. Never	123 (2.9%)
(Missing)	183 (4.2%)
**Relationship with mother**	
1. Very good	2,205 (51%)
2. Somewhat good	1,962 (45%)
3. Somewhat bad	92 (2.1%)
4. Very bad	15 (0.4%)
97. (Does not apply)	14 (0.3%)
(Missing)	28 (0.6%)
**Relationship with father**	
1. Very good	1,879 (44%)
2. Somewhat good	2,029 (47%)
3. Somewhat bad	181 (4.2%)
4. Very bad	30 (0.7%)
97. (Does not apply)	144 (3.3%)
(Missing)	53 (1.2%)
**Outsider growing up**	
1. Yes	186 (4.3%)
2. No	4,067 (94%)
(Missing)	63 (1.5%)

Characteristic	N = 4,316[1]
**Abuse**	
1. Yes	130 (3.0%)
2. No	4,171 (97%)
(Missing)	14 (0.3%)
**Self-rated health growing up**	
1. Excellent	1,392 (32%)
2. Very good	2,116 (49%)
3. Good	614 (14%)
4. Fair	139 (3.2%)
5. Poor	50 (1.2%)
(Missing)	5 (0.1%)
**Immigration status**	
1. Born in this country	4,287 (99%)
2. Born in another country	27 (0.6%)
(Missing)	1 (<0.1%)
**Subjective financial status of family growing up**	
1. Lived comfortably	596 (14%)
2. Got by	2,598 (60%)
3. Found it difficult	906 (21%)
4. Found it very difficult	199 (4.6%)
(Missing)	17 (0.4%)
**Religious affiliation**	
1. Christianity	4,111 (95%)
10. Taoism	0 (0%)
11. Confucianism	0 (0%)
12. Primal, Animist, or Folk religion	0 (0%)
13. Spiritism	0 (0%)
14. Umbanda, Candomblé, and other African-derived religions	0 (0%)
15. Chinese folk/traditional religion	0 (0%)
2. Islam	0 (0%)
3. Hinduism	0 (0%)
4. Buddhism	0 (0%)
5. Judaism	0 (0%)
6. Sikhism	1 (<0.1%)
7. Baha'i	0 (0%)
8. Jainism	0 (0%)
9. Shinto	0 (0%)
96. Some other religion	0 (0%)
97. No religion/Atheist/Agnostic	197 (4.6%)
(Missing)	7 (0.2%)
**Race/Ethnicity**	
(Missing)	2 (<0.1%)
1401. Poland: Polish	4,295 (100%)
1402. Poland: German	1 (<0.1%)
1403. Poland: Belarussian	2 (<0.1%)
1404. Poland: Ukrainian	7 (0.2%)
1409. Poland: Silesia	7 (0.2%)

**Characteristic**	**N = 4,316**[1]
9996. Other	1 (<0.1%)

[1]Mean (SD); n (%)

Table S16c. Poland: Proportions by demographic category

Variable	Category	Proportion	95% CI	SE	p-value
Age group	1998-2005 (current age: 18-24 years)	0.737	(0.660, 0.814)	0.039	3.01e-01
	1943 or earlier (current age: 80+ years)	0.651	(0.433, 0.868)	0.107	
	1943-1953 (current age: 70-79 years)	0.675	(0.588, 0.761)	0.044	
	1953-1963 (current age: 60-69 years)	0.633	(0.580, 0.686)	0.027	
	1963-1973 (current age: 50-59 years)	0.685	(0.636, 0.733)	0.025	
	1973-1983 (current age: 40-49 years)	0.691	(0.640, 0.742)	0.026	
	1983-1993 (current age: 30-39 years)	0.712	(0.666, 0.758)	0.023	
	1993-1998 (current age: 25-29 years)	0.718	(0.647, 0.790)	0.036	
Gender	Male	0.675	(0.634, 0.715)	0.021	< 1e-16
	Female	0.699	(0.660, 0.738)	0.020	
	Other	1.000	*	*	
Marital status	Single/Never been married	0.713	(0.651, 0.775)	0.032	7.65e-01
	Divorced	0.651	(0.547, 0.754)	0.052	
	Domestic partner	0.647	(0.539, 0.756)	0.055	
	Married	0.685	(0.650, 0.720)	0.018	
	Separated	0.666	(0.474, 0.857)	0.092	
	Widowed	0.697	(0.623, 0.771)	0.038	
Employment	Employed for an employer	0.709	(0.673, 0.745)	0.018	1.82e-01
	Homemaker	0.710	(0.592, 0.828)	0.060	
	None of these/Other	0.688	(0.499, 0.877)	0.093	
	Retired	0.641	(0.587, 0.695)	0.027	
	Self-employed	0.686	(0.615, 0.757)	0.036	
	Student	0.726	(0.628, 0.823)	0.050	
	Unemployed and looking for a job	0.605	(0.444, 0.765)	0.081	
Religious service attendance	Never	0.675	(0.597, 0.754)	0.040	5.38e-01
	A few times a year	0.667	(0.612, 0.722)	0.028	
	More than once a week	0.773	(0.654, 0.891)	0.060	
	Once a week	0.709	(0.656, 0.762)	0.027	
	One to three times a month	0.674	(0.629, 0.719)	0.023	
Education	9-15	0.666	(0.629, 0.703)	0.019	2.76e-03
	16+	0.737	(0.697, 0.777)	0.020	

Variable	Category	Proportion	95% CI	SE	p-value
	Up to 8	0.696	(0.572, 0.820)	0.063	
Immigration status	Born in this country	0.688	(0.654, 0.722)	0.017	8.66e-01
	Born in another country	0.705	(0.484, 0.925)	0.107	
Religious affiliation	No religion/Atheist/Agnostic	0.768	(0.712, 0.823)	0.028	1.12e-02
	Christianity	0.679	(0.643, 0.715)	0.018	
	Combined	0.768	(0.000, 1.000)	0.164	
	Spiritism	*	*	*	
	Umbanda, Candomblé, and other African-derived religions	*	*	*	
	Buddhism	*	*	*	
	Islam	*	*	*	
	Chinese folk/traditional religion	*	*	*	
	Hinduism	*	*	*	
	Judaism	*	*	*	
	Primal, Animist, or Folk religion	*	*	*	

Table S16d. Poland: Childhood predictors regression analysis

Variable	Category	Risk-Ratio	logRR SE	RR 95% CI	Global p-value
Relationship with your mother growing up	(Ref: Very bad/somewhat bad)				0.104
	Very good/somewhat good	0.916	0.051	(0.83,1.01)	
Relationship with your father growing up	(Ref: Very bad/somewhat bad)				0.168
	Very good/somewhat good	0.938	0.043	(0.86,1.02)	
Parents married to each other when you were around 12 years old	(Ref: Parents married)				0.348
	One or both of them had died	0.952	0.076	(0.82,1.11)	
	Parents were divorced	0.930	0.041	(0.86,1.01)	
	Parents were never married	0.947	0.097	(0.78,1.14)	
	Unsure	0.979	0.097	(0.81,1.18)	
Feelings about familys household income when growing up	(Ref: Got by)				0.004
	Found it difficult	0.914	0.028	(0.87,0.97)	
	Found it very difficult	1.001	0.057	(0.90,1.12)	
	Lived comfortably	0.994	0.029	(0.94,1.05)	
Physically or sexually abused when growing up	(Ref: No)				0.477
		0.962	0.059	(0.86,1.08)	
Felt like an outsider in your family when growing up	(Ref: No)				0.392
	Yes	0.955	0.050	(0.87,1.05)	
Your health when growing up	(Ref: Good)				0.745
	Excellent	0.999	0.033	(0.94,1.07)	
	Fair	0.995	0.060	(0.88,1.12)	
	Poor	1.092	0.098	(0.90,1.32)	
	Very good	0.983	0.030	(0.93,1.04)	
Born in This	(Ref: Born in this				0.580

Variable	Category	Risk-Ratio	logRR SE	RR 95% CI	Global p-value
country	country)				
	Born in another country	1.027	0.118	(0.81,1.29)	
How Often You Attended Religious Services or Worshiped When You Were Around 12 Years Old	(Ref: Never)				0.404
	At least once a week	1.086	0.067	(0.95,1.24)	
	Less than once a month	0.976	0.063	(0.86,1.10)	
	One to three times a month	1.086	0.063	(0.96,1.23)	
Year of birth (age group)	(Ref: 1998-2005; current age: 18-24)				0.061
	1943 or earlier (current age: 80+ years)	0.924	0.108	(0.75,1.14)	
	1943-1953 (current age: 70-79 years)	0.919	0.055	(0.83,1.02)	
	1953-1963 (current age: 60-69 years)	0.895	0.042	(0.82,0.97)	
	1963-1973 (current age: 50-59 years)	0.941	0.042	(0.87,1.02)	
	1973-1983 (current age: 40-49 years)	0.947	0.038	(0.88,1.02)	
	1983-1993 (current age: 30-39 years)	0.968	0.037	(0.90,1.04)	
	1993-1998 (current age: 25-29 years)	0.969	0.042	(0.89,1.05)	
Religion when twelve years old	(Ref: No religion/Atheist/Agnostic)				1.76e-06
	Christianity	0.931	0.047	(0.85,1.02)	
	Collapsed affiliations with prevalence<3%	0.528	0.119	(0.42,0.67)	
Race plurality (prominent race/ethnic group [0] or not [1])	(Ref: Plurality group)				0.605
	Non-plurality groups	1.014	0.207	(0.68,1.52)	

Table S16e. Poland: Sensitivity to unmeasured confounding of childhood predictors

Variable	Category	E-value for Estimate	E-value for 95% CI
Relationship with your mother growing up	(Ref: Very bad/somewhat bad)		
	Very good/somewhat good	1.41	1.00
Relationship with your father growing up	(Ref: Very bad/somewhat bad)		
	Very good/somewhat good	1.33	1.00
Parents married to each other when you were around 12 years old	(Ref: Parents married)		
	One or both of them had died	1.28	1.00
	Parents were divorced	1.36	1.00
	Parents were never married	1.30	1.00
	Unsure	1.17	1.00
Feelings about familys household income when growing up	(Ref: Got by)		
	Found it difficult	1.41	1.23
	Found it very difficult	1.03	1.00
	Lived comfortably	1.08	1.00
Physically or sexually abused when growing up	(Ref: No)		
		1.24	1.00
Felt like an outsider in your family when growing up	(Ref: No)		
	Yes	1.27	1.00
Your health when growing up	(Ref: Good)		
	Excellent	1.03	1.00
	Fair	1.08	1.00
	Poor	1.41	1.00
	Very good	1.15	1.00
Born in This country	(Ref: Born in this country)		
	Born in another country	1.19	1.00
How Often You Attended Religious Services or Worshiped When You Were Around 12 Years Old	(Ref: Never)		
	At least once a week	1.39	1.00
	Less than once a month	1.18	1.00
	One to three times a month	1.39	1.00
Year of birth (age group)	(Ref: 1998-2005; current age: 18-24)		
	1943 or earlier (current age: 80+ years)	1.38	1.00
	1943-1953 (current age: 70-79 years)	1.40	1.00

Variable	Category	E-value for Estimate	E-value for 95% CI
	1953-1963 (current age: 60-69 years)	1.48	1.20
	1963-1973 (current age: 50-59 years)	1.32	1.00
	1973-1983 (current age: 40-49 years)	1.30	1.00
	1983-1993 (current age: 30-39 years)	1.22	1.00
	1993-1998 (current age: 25-29 years)	1.22	1.00
Religion when twelve years old	(Ref: No religion/Atheist/Agnostic)		
	Christianity	1.36	1.00
	Collapsed affiliations with prevalence<3%	3.19	2.35
Race plurality (prominent race/ethnic group [0] or not [1])	(Ref: Plurality group)		
	Non-plurality groups	1.13	1.00

Table S17a. South Africa: Demographic descriptive statistics

**Characteristic**	**N = 1,625**[1]
**Age group**	
1943 or earlier (current age: 80+ years)	12 (0.7%)
1943-1953 (current age: 70-79 years)	65 (4.0%)
1953-1963 (current age: 60-69 years)	105 (6.5%)
1963-1973 (current age: 50-59 years)	212 (13%)
1973-1983 (current age: 40-49 years)	283 (17%)
1983-1993 (current age: 30-39 years)	406 (25%)
1993-1998 (current age: 25-29 years)	223 (14%)
1998-2005 (current age: 18-24 years)	316 (19%)
(Missing)	3 (0.2%)
**Gender**	
1. Male	777 (48%)
2. Female	846 (52%)
3. Other	0 (0%)
(Missing)	2 (0.1%)
**Marital status**	
1. Single/Never been married	984 (61%)
2. Married	331 (20%)
3. Separated	31 (1.9%)
4. Divorced	21 (1.3%)
5. Widowed	85 (5.2%)
6. Domestic partner	164 (10%)
(Missing)	9 (0.6%)
**Employment**	
1. Employed for an employer	329 (20%)
2. Self-employed	239 (15%)
3. Retired	123 (7.6%)
4. Student	123 (7.5%)
5. Homemaker	99 (6.1%)
6. Unemployed and looking for a job	665 (41%)
7. None of these/Other	45 (2.8%)
(Missing)	2 (0.1%)
**Religious service attendance**	
1. More than once a week	280 (17%)
2. Once a week	551 (34%)
3. One to three times a month	338 (21%)
4. A few times a year	244 (15%)
5. Never	209 (13%)
(Missing)	4 (0.2%)
**Education**	
1. Up to 8	419 (26%)
2. 9-15	1,106 (68%)
3. 16+	98 (6.0%)
(Missing)	2 (0.1%)
**Immigration**	
1. Born in this country	1,536 (95%)
2. Born in another country	89 (5.5%)

**Characteristic**	**N = 1,625**[1]
(Missing)	0 (0%)
**Religious affiliation**	
1. Christianity	1,337 (82%)
10. Taoism	1 (<0.1%)
11. Confucianism	0 (0%)
12. Primal, Animist, or Folk religion	73 (4.5%)
13. Spiritism	0 (0%)
14. Umbanda, Candomblé, and other African-derived religions	0 (0%)
15. Chinese folk/traditional religion	0 (0%)
2. Islam	33 (2.0%)
3. Hinduism	0 (0%)
4. Buddhism	9 (0.6%)
5. Judaism	0 (0%)
6. Sikhism	0 (0%)
7. Baha'i	0 (0%)
8. Jainism	3 (0.2%)
9. Shinto	2 (0.1%)
96. Some other religion	1 (<0.1%)
97. No religion/Atheist/Agnostic	154 (9.5%)
(Missing)	12 (0.7%)
**RACE ETHNICITY**	
(Missing)	3 (0.2%)
1601. South Africa: Black	1,484 (91%)
1602. South Africa: Asian/Indian	2 (0.1%)
1603. South Africa: Colored	127 (7.8%)
1604. South Africa: White	8 (0.5%)
9996. Other	1 (<0.1%)

[1]n (%)

Table S17b. South Africa: Demographic descriptive statistics

**Characteristic**	**N = 1,625**[1]
**Age**	39 (15)
Unknown	3
**Year of birth**	
1943 or earlier (current age: 80+ years)	12 (0.7%)
1943-1953 (current age: 70-79 years)	65 (4.0%)
1953-1963 (current age: 60-69 years)	105 (6.5%)
1963-1973 (current age: 50-59 years)	212 (13%)
1973-1983 (current age: 40-49 years)	283 (17%)
1983-1993 (current age: 30-39 years)	406 (25%)
1993-1998 (current age: 25-29 years)	223 (14%)
1998-2005 (current age: 18-24 years)	316 (19%)
(Missing)	3 (0.2%)
**Gender**	
1. Male	777 (48%)
2. Female	846 (52%)
3. Other	0 (0%)
(Missing)	2 (0.1%)
**Parent marital status**	
1. Parents were married	806 (50%)
2. Parents were divorced	79 (4.9%)
3. Parents were never married	566 (35%)
4. One or both of them had died	89 (5.5%)
5. Unsure	11 (0.7%)
(Missing)	73 (4.5%)
**Age 12 religious service attendance**	
1. At least once a week	1,026 (63%)
2. One to three times a month	338 (21%)
3. Less than once a month	112 (6.9%)
4. Never	132 (8.1%)
(Missing)	17 (1.0%)
**Relationship with mother**	
1. Very good	1,335 (82%)
2. Somewhat good	161 (9.9%)
3. Somewhat bad	23 (1.4%)
4. Very bad	19 (1.2%)
97. (Does not apply)	72 (4.4%)
(Missing)	16 (1.0%)
**Relationship with father**	
1. Very good	968 (60%)
2. Somewhat good	210 (13%)
3. Somewhat bad	54 (3.3%)
4. Very bad	104 (6.4%)
97. (Does not apply)	225 (14%)
(Missing)	63 (3.9%)
**Outsider growing up**	
1. Yes	254 (16%)
2. No	1,368 (84%)

Characteristic	N = 1,625[1]
(Missing)	4 (0.2%)
**Abuse**	
1. Yes	301 (19%)
2. No	1,298 (80%)
(Missing)	26 (1.6%)
**Self-rated health growing up**	
1. Excellent	764 (47%)
2. Very good	358 (22%)
3. Good	197 (12%)
4. Fair	161 (9.9%)
5. Poor	132 (8.2%)
(Missing)	12 (0.8%)
**Immigration status**	
1. Born in this country	1,536 (95%)
2. Born in another country	89 (5.5%)
(Missing)	0 (0%)
**Subjective financial status of family growing up**	
1. Lived comfortably	640 (39%)
2. Got by	516 (32%)
3. Found it difficult	265 (16%)
4. Found it very difficult	201 (12%)
(Missing)	3 (0.2%)
**Religious affiliation**	
1. Christianity	1,450 (89%)
10. Taoism	1 (<0.1%)
11. Confucianism	0 (0%)
12. Primal, Animist, or Folk religion	56 (3.5%)
13. Spiritism	0 (0%)
14. Umbanda, Candomblé, and other African-derived religions	0 (0%)
15. Chinese folk/traditional religion	0 (0%)
2. Islam	26 (1.6%)
3. Hinduism	1 (<0.1%)
4. Buddhism	10 (0.6%)
5. Judaism	0 (0%)
6. Sikhism	0 (0%)
7. Baha'i	0 (0%)
8. Jainism	0 (0%)
9. Shinto	2 (0.1%)
96. Some other religion	4 (0.2%)
97. No religion/Atheist/Agnostic	60 (3.7%)
(Missing)	14 (0.9%)
**Race/Ethnicity**	
(Missing)	3 (0.2%)
1601. South Africa: Black	1,484 (91%)
1602. South Africa: Asian/Indian	2 (0.1%)
1603. South Africa: Colored	127 (7.8%)
1604. South Africa: White	8 (0.5%)

**Characteristic**	**N = 1,625**[1]
9996. Other	1 (<0.1%)

[1]Mean (SD); n (%)

Table S17c. South Africa: Proportions by demographic category

Variable	Category	Proportion	95% CI	SE	p-value
Age group	1998-2005 (current age: 18-24 years)	0.904	(0.854, 0.953)	0.025	6.71e-01
	1943 or earlier (current age: 80+ years)	0.765	(0.000, 1.000)	0.202	
	1943-1953 (current age: 70-79 years)	0.929	(0.839, 1.000)	0.045	
	1953-1963 (current age: 60-69 years)	0.934	(0.883, 0.985)	0.026	
	1963-1973 (current age: 50-59 years)	0.900	(0.836, 0.964)	0.032	
	1973-1983 (current age: 40-49 years)	0.869	(0.813, 0.926)	0.029	
	1983-1993 (current age: 30-39 years)	0.909	(0.876, 0.943)	0.017	
	1993-1998 (current age: 25-29 years)	0.913	(0.866, 0.960)	0.024	
Gender	Male	0.905	(0.872, 0.938)	0.017	7.71e-01
	Female	0.899	(0.875, 0.924)	0.012	
	Other	*	*	*	
Marital status	Single/Never been married	0.898	(0.870, 0.926)	0.014	2.44e-02
	Divorced	0.979	(0.932, 1.000)	0.022	
	Domestic partner	0.935	(0.877, 0.992)	0.029	
	Married	0.903	(0.851, 0.954)	0.026	
	Separated	0.829	(0.662, 0.996)	0.081	
	Widowed	0.887	(0.801, 0.972)	0.043	
Employment	Employed for an employer	0.893	(0.843, 0.943)	0.025	9.04e-01
	Homemaker	0.892	(0.814, 0.970)	0.039	
	None of these/Other	0.922	(0.830, 1.000)	0.045	
	Retired	0.908	(0.824, 0.992)	0.042	
	Self-employed	0.914	(0.871, 0.956)	0.022	
	Student	0.851	(0.752, 0.949)	0.050	
	Unemployed and looking for a job	0.911	(0.879, 0.942)	0.016	
Religious service attendance	Never	0.852	(0.776, 0.927)	0.038	7.18e-02
	A few times a year	0.893	(0.838, 0.947)	0.028	
	More than once a week	0.900	(0.848, 0.953)	0.027	
	Once a week	0.903	(0.864, 0.942)	0.020	
	One to three times a month	0.939	(0.910, 0.969)	0.015	
Education	9-15	0.910	(0.888, 0.931)	0.011	2.96e-02
	16+	0.959	(0.917, 1.000)	0.021	

Variable	Category	Proportion	95% CI	SE	p-value
	Up to 8	0.868	(0.810, 0.926)	0.029	
Immigration status	Born in this country	0.904	(0.883, 0.926)	0.011	5.07e-01
	Born in another country	0.861	(0.732, 0.991)	0.065	
Religious affiliation	No religion/Atheist/Agnostic	0.887	(0.811, 0.963)	0.038	6.32e-01
	Christianity	0.904	(0.882, 0.926)	0.011	
	Combined	0.943	(0.859, 1.000)	0.042	
	Spiritism	*	*	*	
	Umbanda, Candomblé, and other African-derived religions	*	*	*	
	Buddhism	*	*	*	
	Islam	*	*	*	
	Chinese folk/traditional religion	*	*	*	
	Hinduism	*	*	*	
	Judaism	*	*	*	
	Primal, Animist, or Folk religion	0.870	(0.759, 0.981)	0.056	

Table S17d. South Africa: Childhood predictors regression analysis

Variable	Category	Risk-Ratio	logRR SE	RR 95% CI	Global p-value
Relationship with your mother growing up	(Ref: Very bad/somewhat bad)				0.257
	Very good/somewhat good	1.065	0.050	(0.96,1.18)	
Relationship with your father growing up	(Ref: Very bad/somewhat bad)				0.597
	Very good/somewhat good	1.004	0.032	(0.94,1.07)	
Parents married to each other when you were around 12 years old	(Ref: Parents married)				0.605
	One or both of them had died	1.000	0.055	(0.90,1.11)	
	Parents were divorced	1.031	0.046	(0.94,1.13)	
	Parents were never married	1.002	0.022	(0.96,1.05)	
	Unsure	0.771	0.191	(0.53,1.12)	
Feelings about familys household income when growing up	(Ref: Got by)				0.521
	Found it difficult	0.959	0.033	(0.90,1.02)	
	Found it very difficult	0.976	0.041	(0.90,1.06)	
	Lived comfortably	0.988	0.024	(0.94,1.04)	
Physically or sexually abused when growing up	(Ref: No)				0.527
		0.985	0.031	(0.93,1.05)	
Felt like an outsider in your family when growing up	(Ref: No)				0.060
	Yes	1.046	0.023	(1.00,1.09)	
Your health when growing up	(Ref: Good)				0.571
	Excellent	0.989	0.031	(0.93,1.05)	
	Fair	0.989	0.045	(0.90,1.08)	
	Poor	1.046	0.038	(0.97,1.13)	
	Very good	0.962	0.035	(0.90,1.03)	
Born in This	(Ref: Born in this				0.477

Variable	Category	Risk-Ratio	logRR SE	RR 95% CI	Global p-value
country	country)				
	Born in another country	0.960	0.062	(0.85,1.08)	
How Often You Attended Religious Services or Worshiped When You Were Around 12 Years Old	(Ref: Never)				0.762
	At least once a week	0.999	0.058	(0.89,1.12)	
	Less than once a month	0.963	0.072	(0.84,1.11)	
	One to three times a month	0.994	0.062	(0.88,1.12)	
Year of birth (age group)	(Ref: 1998-2005; current age: 18-24)				0.831
	1943 or earlier (current age: 80+ years)	0.872	0.208	(0.58,1.31)	
	1943-1953 (current age: 70-79 years)	1.019	0.053	(0.92,1.13)	
	1953-1963 (current age: 60-69 years)	1.028	0.039	(0.95,1.11)	
	1963-1973 (current age: 50-59 years)	0.992	0.042	(0.91,1.08)	
	1973-1983 (current age: 40-49 years)	0.967	0.035	(0.90,1.04)	
	1983-1993 (current age: 30-39 years)	1.004	0.030	(0.95,1.06)	
	1993-1998 (current age: 25-29 years)	1.010	0.034	(0.94,1.08)	
Religion when twelve years old	(Ref: No religion/Atheist/Agnostic)				0.662
	Christianity	1.001	0.060	(0.89,1.13)	
	Collapsed affiliations with prevalence<3%	1.066	0.070	(0.93,1.22)	
	Primal, Animist, or Folk religion	0.961	0.077	(0.83,1.12)	
Race plurality (prominent race/ethnic group [0] or not [1])	(Ref: Plurality group)				0.608
	Non-plurality groups	1.002	0.036	(0.93,1.08)	

Table S17e. South Africa: Sensitivity to unmeasured confounding of childhood predictors

Variable	Category	E-value for Estimate	E-value for 95% CI
Relationship with your mother growing up	(Ref: Very bad/somewhat bad)		
	Very good/somewhat good	1.33	1.00
Relationship with your father growing up	(Ref: Very bad/somewhat bad)		
	Very good/somewhat good	1.07	1.00
Parents married to each other when you were around 12 years old	(Ref: Parents married)		
	One or both of them had died	1.00	1.00
	Parents were divorced	1.21	1.00
	Parents were never married	1.04	1.00
	Unsure	1.92	1.00
Feelings about familys household income when growing up	(Ref: Got by)		
	Found it difficult	1.25	1.00
	Found it very difficult	1.18	1.00
	Lived comfortably	1.12	1.00
Physically or sexually abused when growing up	(Ref: No)		
		1.14	1.00
Felt like an outsider in your family when growing up	(Ref: No)		
	Yes	1.27	1.00
Your health when growing up	(Ref: Good)		
	Excellent	1.12	1.00
	Fair	1.12	1.00
	Poor	1.26	1.00
	Very good	1.24	1.00
Born in This country	(Ref: Born in this country)		
	Born in another country	1.25	1.00
How Often You Attended Religious Services or Worshiped When You Were Around 12 Years Old	(Ref: Never)		
	At least once a week	1.04	1.00
	Less than once a month	1.24	1.00
	One to three times a month	1.08	1.00
Year of birth (age group)	(Ref: 1998-2005; current age: 18-24)		
	1943 or earlier (current age: 80+ years)	1.56	1.00
	1943-1953 (current age: 70-79 years)	1.16	1.00

Variable	Category	E-value for Estimate	E-value for 95% CI
	1953-1963 (current age: 60-69 years)	1.20	1.00
	1963-1973 (current age: 50-59 years)	1.10	1.00
	1973-1983 (current age: 40-49 years)	1.22	1.00
	1983-1993 (current age: 30-39 years)	1.07	1.00
	1993-1998 (current age: 25-29 years)	1.11	1.00
Religion when twelve years old	(Ref: No religion/Atheist/Agnostic)		
	Christianity	1.03	1.00
	Collapsed affiliations with prevalence<3%	1.33	1.00
	Primal, Animist, or Folk religion	1.25	1.00
Race plurality (prominent race/ethnic group [0] or not [1])	(Ref: Plurality group)		
	Non-plurality groups	1.04	1.00

Table S18a. Spain: Demographic descriptive statistics

**Characteristic**	**N = 2,215**[1]
**Age group**	
1943 or earlier (current age: 80+ years)	10 (0.4%)
1943-1953 (current age: 70-79 years)	133 (6.0%)
1953-1963 (current age: 60-69 years)	362 (16%)
1963-1973 (current age: 50-59 years)	451 (20%)
1973-1983 (current age: 40-49 years)	524 (24%)
1983-1993 (current age: 30-39 years)	380 (17%)
1993-1998 (current age: 25-29 years)	152 (6.9%)
1998-2005 (current age: 18-24 years)	203 (9.2%)
(Missing)	0 (0%)
**Gender**	
1. Male	1,105 (50%)
2. Female	1,102 (50%)
3. Other	0 (<0.1%)
(Missing)	8 (0.4%)
**Marital status**	
1. Single/Never been married	622 (28%)
2. Married	1,060 (48%)
3. Separated	56 (2.5%)
4. Divorced	188 (8.5%)
5. Widowed	64 (2.9%)
6. Domestic partner	214 (9.7%)
(Missing)	10 (0.4%)
**Employment**	
1. Employed for an employer	1,103 (50%)
2. Self-employed	158 (7.1%)
3. Retired	393 (18%)
4. Student	155 (7.0%)
5. Homemaker	140 (6.3%)
6. Unemployed and looking for a job	227 (10%)
7. None of these/Other	34 (1.5%)
(Missing)	5 (0.2%)
**Religious service attendance**	
1. More than once a week	86 (3.9%)
2. Once a week	231 (10%)
3. One to three times a month	125 (5.7%)
4. A few times a year	696 (31%)
5. Never	1,069 (48%)
(Missing)	7 (0.3%)
**Education**	
1. Up to 8	268 (12%)
2. 9-15	1,443 (65%)
3. 16+	504 (23%)
(Missing)	0 (0%)
**Immigration**	
1. Born in this country	1,948 (88%)
2. Born in another country	258 (12%)

**Characteristic**	**N = 2,215**[1]
(Missing)	9 (0.4%)
**Religious affiliation**	
1. Christianity	1,350 (61%)
10. Taoism	0 (0%)
11. Confucianism	1 (<0.1%)
12. Primal, Animist, or Folk religion	2 (<0.1%)
13. Spiritism	0 (0%)
14. Umbanda, Candomblé, and other African-derived religions	0 (0%)
15. Chinese folk/traditional religion	0 (0%)
2. Islam	39 (1.8%)
3. Hinduism	1 (<0.1%)
4. Buddhism	9 (0.4%)
5. Judaism	0 (<0.1%)
6. Sikhism	0 (<0.1%)
7. Baha'i	1 (<0.1%)
8. Jainism	0 (0%)
9. Shinto	0 (0%)
96. Some other religion	13 (0.6%)
97. No religion/Atheist/Agnostic	778 (35%)
(Missing)	21 (0.9%)

[1] n (%)

Table S18b. Spain: Childhood descriptive statistics

**Characteristic**	**N = 2,215**[1]
**Age**	47 (15)
**Year of birth**	
1943 or earlier (current age: 80+ years)	10 (0.4%)
1943-1953 (current age: 70-79 years)	133 (6.0%)
1953-1963 (current age: 60-69 years)	362 (16%)
1963-1973 (current age: 50-59 years)	451 (20%)
1973-1983 (current age: 40-49 years)	524 (24%)
1983-1993 (current age: 30-39 years)	380 (17%)
1993-1998 (current age: 25-29 years)	152 (6.9%)
1998-2005 (current age: 18-24 years)	203 (9.2%)
(Missing)	0 (0%)
**Gender**	
1. Male	1,105 (50%)
2. Female	1,102 (50%)
3. Other	0 (<0.1%)
(Missing)	8 (0.4%)
**Parent marital status**	
1. Parents were married	1,941 (88%)
2. Parents were divorced	122 (5.5%)
3. Parents were never married	87 (3.9%)
4. One or both of them had died	37 (1.7%)
5. Unsure	18 (0.8%)
(Missing)	10 (0.4%)
**Age 12 religious service attendance**	
1. At least once a week	800 (36%)
2. One to three times a month	393 (18%)
3. Less than once a month	466 (21%)
4. Never	537 (24%)
(Missing)	18 (0.8%)
**Relationship with mother**	
1. Very good	1,571 (71%)
2. Somewhat good	447 (20%)
3. Somewhat bad	94 (4.2%)
4. Very bad	28 (1.3%)
97. (Does not apply)	66 (3.0%)
(Missing)	10 (0.4%)
**Relationship with father**	
1. Very good	1,408 (64%)
2. Somewhat good	515 (23%)
3. Somewhat bad	116 (5.2%)
4. Very bad	69 (3.1%)
97. (Does not apply)	94 (4.3%)
(Missing)	13 (0.6%)
**Outsider growing up**	
1. Yes	223 (10%)
2. No	1,967 (89%)
(Missing)	25 (1.1%)

Characteristic	N = 2,215[1]
**Abuse**	
1. Yes	240 (11%)
2. No	1,967 (89%)
(Missing)	8 (0.4%)
**Self-rated health growing up**	
1. Excellent	869 (39%)
2. Very good	789 (36%)
3. Good	434 (20%)
4. Fair	82 (3.7%)
5. Poor	31 (1.4%)
(Missing)	10 (0.4%)
**Immigration status**	
1. Born in this country	1,948 (88%)
2. Born in another country	258 (12%)
(Missing)	9 (0.4%)
**Subjective financial status of family growing up**	
1. Lived comfortably	694 (31%)
2. Got by	1,015 (46%)
3. Found it difficult	455 (21%)
4. Found it very difficult	44 (2.0%)
(Missing)	7 (0.3%)
**Religious affiliation**	
1. Christianity	1,851 (84%)
10. Taoism	0 (0%)
11. Confucianism	1 (<0.1%)
12. Primal, Animist, or Folk religion	0 (<0.1%)
13. Spiritism	0 (0%)
14. Umbanda, Candomblé, and other African-derived religions	0 (0%)
15. Chinese folk/traditional religion	0 (0%)
2. Islam	31 (1.4%)
3. Hinduism	0 (<0.1%)
4. Buddhism	1 (<0.1%)
5. Judaism	0 (<0.1%)
6. Sikhism	0 (0%)
7. Baha'i	0 (0%)
8. Jainism	0 (0%)
9. Shinto	0 (0%)
96. Some other religion	6 (0.3%)
97. No religion/Atheist/Agnostic	311 (14%)
(Missing)	13 (0.6%)

[1]Mean (SD); n (%)

Table S18c. Spain: Proportions by demographic category

Variable	Category	Proportion	95% CI	SE	p-value
Age group	1998-2005 (current age: 18-24 years)	0.840	(0.745, 0.935)	0.048	6.76e-01
	1943 or earlier (current age: 80+ years)	0.917	*	*	
	1943-1953 (current age: 70-79 years)	0.775	(0.639, 0.912)	0.069	
	1953-1963 (current age: 60-69 years)	0.793	(0.723, 0.863)	0.036	
	1963-1973 (current age: 50-59 years)	0.787	(0.744, 0.831)	0.022	
	1973-1983 (current age: 40-49 years)	0.822	(0.784, 0.861)	0.020	
	1983-1993 (current age: 30-39 years)	0.793	(0.741, 0.845)	0.026	
	1993-1998 (current age: 25-29 years)	0.774	(0.669, 0.879)	0.053	
Gender	Male	0.795	(0.759, 0.832)	0.018	< 1e-16
	Female	0.808	(0.776, 0.839)	0.016	
	Other	0.037	*	*	
Marital status	Single/Never been married	0.825	(0.780, 0.870)	0.023	3.50e-01
	Divorced	0.711	(0.601, 0.821)	0.056	
	Domestic partner	0.832	(0.773, 0.891)	0.030	
	Married	0.802	(0.770, 0.834)	0.017	
	Separated	0.730	(0.548, 0.912)	0.091	
	Widowed	0.781	(0.630, 0.932)	0.075	
Employment	Employed for an employer	0.815	(0.787, 0.844)	0.015	1.65e-01
	Homemaker	0.817	(0.729, 0.906)	0.045	
	None of these/Other	0.825	(0.534, 1.000)	0.135	
	Retired	0.768	(0.697, 0.838)	0.036	
	Self-employed	0.838	(0.763, 0.912)	0.038	
	Student	0.842	(0.730, 0.954)	0.057	
	Unemployed and looking for a job	0.724	(0.645, 0.802)	0.040	
Religious service attendance	Never	0.792	(0.757, 0.826)	0.018	6.31e-01
	A few times a year	0.797	(0.755, 0.838)	0.021	
	More than once a week	0.854	(0.757, 0.951)	0.049	
	Once a week	0.822	(0.735, 0.909)	0.044	
	One to three times a month	0.834	(0.752, 0.917)	0.041	
Education	9-15	0.785	(0.757, 0.814)	0.014	4.07e-06
	16+	0.889	(0.854, 0.923)	0.018	

Variable	Category	Proportion	95% CI	SE	p-value
	Up to 8	0.723	(0.616, 0.830)	0.054	
Immigration status	Born in this country	0.802	(0.777, 0.828)	0.013	8.45e-01
	Born in another country	0.795	(0.724, 0.866)	0.036	
Religious affiliation	No religion/Atheist/Agnostic	0.795	(0.753, 0.838)	0.022	5.07e-01
	Christianity	0.809	(0.779, 0.838)	0.015	
	Combined	0.722	(0.561, 0.882)	0.080	
	Spiritism	*	*	*	
	Umbanda, Candomblé, and other African-derived religions	*	*	*	
	Buddhism	*	*	*	
	Islam	*	*	*	
	Chinese folk/traditional religion	*	*	*	
	Hinduism	*	*	*	
	Judaism	*	*	*	
	Primal, Animist, or Folk religion	*	*	*	

Table S18d. Spain: Childhood predictors regression analysis

Variable	Category	Risk-Ratio	logRR SE	RR 95% CI	Global p-value
Relationship with your mother growing up	(Ref: Very bad/somewhat bad)				0.461
	Very good/somewhat good	1.038	0.053	(0.94,1.15)	
Relationship with your father growing up	(Ref: Very bad/somewhat bad)				0.319
	Very good/somewhat good	1.052	0.046	(0.96,1.15)	
Parents married to each other when you were around 12 years old	(Ref: Parents married)				3.91e-05
	One or both of them had died	1.213	0.044	(1.11,1.32)	
	Parents were divorced	1.060	0.059	(0.95,1.19)	
	Parents were never married	1.037	0.057	(0.93,1.16)	
	Unsure	0.850	0.200	(0.57,1.26)	
Feelings about familys household income when growing up	(Ref: Got by)				0.354
	Found it difficult	1.005	0.033	(0.94,1.07)	
	Found it very difficult	0.874	0.098	(0.72,1.06)	
	Lived comfortably	1.036	0.026	(0.98,1.09)	
Physically or sexually abused when growing up	(Ref: No)				0.276
		1.049	0.040	(0.97,1.13)	
Felt like an outsider in your family when growing up	(Ref: No)				0.277
	Yes	0.941	0.050	(0.85,1.04)	
Your health when growing up	(Ref: Good)				0.273
	Excellent	1.067	0.034	(1.00,1.14)	
	Fair	1.017	0.073	(0.88,1.17)	
	Poor	1.009	0.106	(0.82,1.24)	
	Very good	1.035	0.035	(0.97,1.11)	
Born in This	(Ref: Born in this				0.496

Variable	Category	Risk-Ratio	logRR SE	RR 95% CI	Global p-value
country	country)				
	Born in another country	0.977	0.039	(0.91,1.05)	
How Often You Attended Religious Services or Worshiped When You Were Around 12 Years Old	(Ref: Never)				0.093
	At least once a week	1.074	0.034	(1.00,1.15)	
	Less than once a month	0.986	0.038	(0.92,1.06)	
	One to three times a month	1.066	0.036	(0.99,1.14)	
Year of birth (age group)	(Ref: 1998-2005; current age: 18-24)				0.539
	1943 or earlier (current age: 80+ years)	1.034	0.095	(0.86,1.24)	
	1943-1953 (current age: 70-79 years)	0.903	0.081	(0.77,1.06)	
	1953-1963 (current age: 60-69 years)	0.934	0.058	(0.83,1.05)	
	1963-1973 (current age: 50-59 years)	0.934	0.052	(0.84,1.03)	
	1973-1983 (current age: 40-49 years)	0.974	0.051	(0.88,1.08)	
	1983-1993 (current age: 30-39 years)	0.943	0.054	(0.85,1.05)	
	1993-1998 (current age: 25-29 years)	0.931	0.073	(0.81,1.08)	
Religion when twelve years old	(Ref: No religion/Atheist/Agnostic)				0.383
	Christianity	1.003	0.038	(0.93,1.08)	
	Collapsed affiliations with prevalence<3%	0.851	0.117	(0.68,1.07)	

Table S18e. Spain: Sensitivity to unmeasured confounding of childhood predictors

Variable	Category	E-value for Estimate	E-value for 95% CI
Relationship with your mother growing up	(Ref: Very bad/somewhat bad)		
	Very good/somewhat good	1.24	1.00
Relationship with your father growing up	(Ref: Very bad/somewhat bad)		
	Very good/somewhat good	1.29	1.00
Parents married to each other when you were around 12 years old	(Ref: Parents married)		
	One or both of them had died	1.72	1.47
	Parents were divorced	1.31	1.00
	Parents were never married	1.23	1.00
	Unsure	1.63	1.00
Feelings about familys household income when growing up	(Ref: Got by)		
	Found it difficult	1.08	1.00
	Found it very difficult	1.55	1.00
	Lived comfortably	1.23	1.00
Physically or sexually abused when growing up	(Ref: No)		
		1.28	1.00
Felt like an outsider in your family when growing up	(Ref: No)		
	Yes	1.32	1.00
Your health when growing up	(Ref: Good)		
	Excellent	1.34	1.00
	Fair	1.15	1.00
	Poor	1.10	1.00
	Very good	1.22	1.00
Born in This country	(Ref: Born in this country)		
	Born in another country	1.18	1.00
How Often You Attended Religious Services or Worshiped When You Were Around 12 Years Old	(Ref: Never)		
	At least once a week	1.36	1.07
	Less than once a month	1.13	1.00
	One to three times a month	1.33	1.00
Year of birth (age group)	(Ref: 1998-2005; current age: 18-24)		
	1943 or earlier (current age: 80+ years)	1.22	1.00
	1943-1953 (current age: 70-79 years)	1.45	1.00

Variable	Category	E-value for Estimate	E-value for 95% CI
	1953-1963 (current age: 60-69 years)	1.35	1.00
	1963-1973 (current age: 50-59 years)	1.35	1.00
	1973-1983 (current age: 40-49 years)	1.19	1.00
	1983-1993 (current age: 30-39 years)	1.32	1.00
	1993-1998 (current age: 25-29 years)	1.36	1.00
Religion when twelve years old	(Ref: No religion/Atheist/Agnostic)		
	Christianity	1.06	1.00
	Collapsed affiliations with prevalence<3%	1.63	1.00

Table S19a. Sweden: Demographic descriptive statistics

**Characteristic**	**N = 11,570**[1]
**Age group**	
1943 or earlier (current age: 80+ years)	448 (3.9%)
1943-1953 (current age: 70-79 years)	1,716 (15%)
1953-1963 (current age: 60-69 years)	1,657 (14%)
1963-1973 (current age: 50-59 years)	1,945 (17%)
1973-1983 (current age: 40-49 years)	1,808 (16%)
1983-1993 (current age: 30-39 years)	1,935 (17%)
1993-1998 (current age: 25-29 years)	981 (8.5%)
1998-2005 (current age: 18-24 years)	1,080 (9.3%)
(Missing)	0 (0%)
**Gender**	
1. Male	5,680 (49%)
2. Female	5,855 (51%)
3. Other	24 (0.2%)
(Missing)	12 (0.1%)
**Marital status**	
1. Single/Never been married	2,979 (26%)
2. Married	4,864 (42%)
3. Separated	317 (2.7%)
4. Divorced	628 (5.4%)
5. Widowed	330 (2.9%)
6. Domestic partner	2,403 (21%)
(Missing)	50 (0.4%)
**Employment**	
1. Employed for an employer	6,123 (53%)
2. Self-employed	842 (7.3%)
3. Retired	2,969 (26%)
4. Student	1,014 (8.8%)
5. Homemaker	62 (0.5%)
6. Unemployed and looking for a job	247 (2.1%)
7. None of these/Other	301 (2.6%)
(Missing)	11 (<0.1%)
**Religious service attendance**	
1. More than once a week	166 (1.4%)
2. Once a week	279 (2.4%)
3. One to three times a month	339 (2.9%)
4. A few times a year	2,981 (26%)
5. Never	7,773 (67%)
(Missing)	32 (0.3%)
**Education**	
1. Up to 8	194 (1.7%)
2. 9-15	8,409 (73%)
3. 16+	2,967 (26%)
(Missing)	0 (0%)
**Immigration**	
1. Born in this country	10,738 (93%)
2. Born in another country	763 (6.6%)

**Characteristic**	**N = 11,570**[1]
(Missing)	69 (0.6%)
**Religious affiliation**	
1. Christianity	6,352 (55%)
10. Taoism	3 (<0.1%)
11. Confucianism	0 (0%)
12. Primal, Animist, or Folk religion	59 (0.5%)
13. Spiritism	0 (0%)
14. Umbanda, Candomblé, and other African-derived religions	0 (0%)
15. Chinese folk/traditional religion	0 (0%)
2. Islam	257 (2.2%)
3. Hinduism	12 (0.1%)
4. Buddhism	74 (0.6%)
5. Judaism	41 (0.4%)
6. Sikhism	0 (0%)
7. Baha'i	2 (<0.1%)
8. Jainism	0 (0%)
9. Shinto	0 (<0.1%)
96. Some other religion	146 (1.3%)
97. No religion/Atheist/Agnostic	4,560 (39%)
(Missing)	62 (0.5%)

[1]n (%)

Table S19b. Sweden: Childhood descriptive statistics

**Characteristic**	**N = 11,570**[1]
**Age**	49 (18)
**Year of birth**	
1943 or earlier (current age: 80+ years)	448 (3.9%)
1943-1953 (current age: 70-79 years)	1,716 (15%)
1953-1963 (current age: 60-69 years)	1,657 (14%)
1963-1973 (current age: 50-59 years)	1,945 (17%)
1973-1983 (current age: 40-49 years)	1,808 (16%)
1983-1993 (current age: 30-39 years)	1,935 (17%)
1993-1998 (current age: 25-29 years)	981 (8.5%)
1998-2005 (current age: 18-24 years)	1,080 (9.3%)
(Missing)	0 (0%)
**Gender**	
1. Male	5,680 (49%)
2. Female	5,855 (51%)
3. Other	24 (0.2%)
(Missing)	12 (0.1%)
**Parent marital status**	
1. Parents were married	8,354 (72%)
2. Parents were divorced	1,463 (13%)
3. Parents were never married	1,384 (12%)
4. One or both of them had died	269 (2.3%)
5. Unsure	58 (0.5%)
(Missing)	42 (0.4%)
**Age 12 religious service attendance**	
1. At least once a week	691 (6.0%)
2. One to three times a month	996 (8.6%)
3. Less than once a month	4,715 (41%)
4. Never	5,128 (44%)
(Missing)	39 (0.3%)
**Relationship with mother**	
1. Very good	6,661 (58%)
2. Somewhat good	3,523 (30%)
3. Somewhat bad	917 (7.9%)
4. Very bad	287 (2.5%)
97. (Does not apply)	164 (1.4%)
(Missing)	17 (0.1%)
**Relationship with father**	
1. Very good	5,388 (47%)
2. Somewhat good	3,827 (33%)
3. Somewhat bad	1,252 (11%)
4. Very bad	550 (4.8%)
97. (Does not apply)	542 (4.7%)
(Missing)	12 (0.1%)
**Outsider growing up**	
1. Yes	1,429 (12%)
2. No	10,006 (86%)
(Missing)	135 (1.2%)

Characteristic	N = 11,570[1]
**Abuse**	
1. Yes	1,752 (15%)
2. No	9,786 (85%)
(Missing)	32 (0.3%)
**Self-rated health growing up**	
1. Excellent	4,308 (37%)
2. Very good	3,941 (34%)
3. Good	2,101 (18%)
4. Fair	870 (7.5%)
5. Poor	328 (2.8%)
(Missing)	22 (0.2%)
**Immigration status**	
1. Born in this country	10,738 (93%)
2. Born in another country	763 (6.6%)
(Missing)	69 (0.6%)
**Subjective financial status of family growing up**	
1. Lived comfortably	4,524 (39%)
2. Got by	5,971 (52%)
3. Found it difficult	954 (8.2%)
4. Found it very difficult	105 (0.9%)
(Missing)	17 (0.1%)
**Religious affiliation**	
1. Christianity	8,175 (71%)
10. Taoism	0 (0%)
11. Confucianism	1 (<0.1%)
12. Primal, Animist, or Folk religion	23 (0.2%)
13. Spiritism	0 (0%)
14. Umbanda, Candomblé, and other African-derived religions	0 (0%)
15. Chinese folk/traditional religion	0 (0%)
2. Islam	270 (2.3%)
3. Hinduism	7 (<0.1%)
4. Buddhism	25 (0.2%)
5. Judaism	37 (0.3%)
6. Sikhism	3 (<0.1%)
7. Baha'i	2 (<0.1%)
8. Jainism	0 (0%)
9. Shinto	0 (0%)
96. Some other religion	52 (0.5%)
97. No religion/Atheist/Agnostic	2,953 (26%)
(Missing)	21 (0.2%)

[1]Mean (SD); n (%)

Table S19c. Sweden: Proportions by demographic category

Variable	Category	Proportion	95% CI	SE	p-value
Age group	1998-2005 (current age: 18-24 years)	0.841	(0.813, 0.869)	0.014	2.51e-01
	1943 or earlier (current age: 80+ years)	0.874	(0.842, 0.905)	0.016	
	1943-1953 (current age: 70-79 years)	0.840	(0.820, 0.859)	0.010	
	1953-1963 (current age: 60-69 years)	0.843	(0.825, 0.862)	0.009	
	1963-1973 (current age: 50-59 years)	0.845	(0.826, 0.863)	0.009	
	1973-1983 (current age: 40-49 years)	0.829	(0.808, 0.850)	0.011	
	1983-1993 (current age: 30-39 years)	0.830	(0.811, 0.850)	0.010	
	1993-1998 (current age: 25-29 years)	0.818	(0.789, 0.847)	0.015	
Gender	Male	0.803	(0.790, 0.815)	0.006	< 1e-16
	Female	0.871	(0.861, 0.881)	0.005	
	Other	0.915	(0.803, 1.000)	0.053	
Marital status	Single/Never been married	0.827	(0.810, 0.844)	0.009	2.79e-01
	Divorced	0.842	(0.811, 0.872)	0.015	
	Domestic partner	0.828	(0.811, 0.845)	0.009	
	Married	0.848	(0.837, 0.860)	0.006	
	Separated	0.839	(0.796, 0.882)	0.022	
	Widowed	0.829	(0.786, 0.873)	0.022	
Employment	Employed for an employer	0.827	(0.816, 0.838)	0.006	4.65e-04
	Homemaker	0.860	(0.758, 0.961)	0.051	
	None of these/Other	0.825	(0.776, 0.873)	0.025	
	Retired	0.846	(0.832, 0.861)	0.007	
	Self-employed	0.879	(0.849, 0.908)	0.015	
	Student	0.862	(0.835, 0.888)	0.013	
	Unemployed and looking for a job	0.755	(0.698, 0.812)	0.029	
Religious service attendance	Never	0.814	(0.804, 0.824)	0.005	1.11e-16
	A few times a year	0.885	(0.871, 0.899)	0.007	
	More than once a week	0.909	(0.849, 0.969)	0.030	
	Once a week	0.905	(0.862, 0.948)	0.022	
	One to three times a month	0.860	(0.816, 0.905)	0.023	
Education	9-15	0.820	(0.810, 0.830)	0.005	< 1e-16
	16+	0.893	(0.880, 0.905)	0.006	

Variable	Category	Proportion	95% CI	SE	p-value
	Up to 8	0.756	(0.684, 0.829)	0.037	
Immigration status	Born in this country	0.835	(0.827, 0.844)	0.004	4.79e-02
	Born in another country	0.866	(0.837, 0.895)	0.015	
Religious affiliation	No religion/Atheist/Agnostic	0.832	(0.820, 0.843)	0.006	3.66e-01
	Christianity	0.840	(0.829, 0.851)	0.005	
	Combined	0.857	(0.816, 0.898)	0.021	
	Spiritism	*	*	*	
	Umbanda, Candomblé, and other African-derived religions	*	*	*	
	Buddhism	*	*	*	
	Islam	*	*	*	
	Chinese folk/traditional religion	*	*	*	
	Hinduism	*	*	*	
	Judaism	*	*	*	
	Primal, Animist, or Folk religion	*	*	*	

Table S19d. Sweden: Childhood predictors regression analysis

Variable	Category	Risk-Ratio	logRR SE	RR 95% CI	Global p-value
Relationship with your mother growing up	(Ref: Very bad/somewhat bad)				0.567
	Very good/somewhat good	1.004	0.014	(0.98,1.03)	
Relationship with your father growing up	(Ref: Very bad/somewhat bad)				0.428
	Very good/somewhat good	0.990	0.012	(0.97,1.01)	
Parents married to each other when you were around 12 years old	(Ref: Parents married)				0.521
	One or both of them had died	1.027	0.028	(0.97,1.08)	
	Parents were divorced	1.018	0.013	(0.99,1.04)	
	Parents were never married	0.996	0.014	(0.97,1.02)	
	Unsure	1.023	0.053	(0.92,1.13)	
Feelings about familys household income when growing up	(Ref: Got by)				0.006
	Found it difficult	0.981	0.016	(0.95,1.01)	
	Found it very difficult	1.014	0.038	(0.94,1.09)	
	Lived comfortably	1.028	0.009	(1.01,1.05)	
Physically or sexually abused when growing up	(Ref: No)				0.005
		1.032	0.011	(1.01,1.06)	
Felt like an outsider in your family when growing up	(Ref: No)				0.173
	Yes	1.020	0.014	(0.99,1.05)	
Your health when growing up	(Ref: Good)				0.489
	Excellent	1.003	0.012	(0.98,1.03)	
	Fair	0.977	0.017	(0.94,1.01)	
	Poor	0.980	0.026	(0.93,1.03)	
	Very good	0.986	0.012	(0.96,1.01)	
Born in This	(Ref: Born in this				0.113

Variable	Category	Risk-Ratio	logRR SE	RR 95% CI	Global p-value
country	country)				
	Born in another country	1.027	0.016	(1.00,1.06)	
How Often You Attended Religious Services or Worshiped When You Were Around 12 Years Old	(Ref: Never)				6.66e-16
	At least once a week	1.113	0.016	(1.08,1.15)	
	Less than once a month	1.087	0.009	(1.07,1.11)	
	One to three times a month	1.074	0.015	(1.04,1.11)	
Year of birth (age group)	(Ref: 1998-2005; current age: 18-24)				0.652
	1943 or earlier (current age: 80+ years)	1.037	0.023	(0.99,1.08)	
	1943-1953 (current age: 70-79 years)	1.007	0.019	(0.97,1.05)	
	1953-1963 (current age: 60-69 years)	1.011	0.018	(0.98,1.05)	
	1963-1973 (current age: 50-59 years)	1.010	0.018	(0.97,1.05)	
	1973-1983 (current age: 40-49 years)	0.995	0.018	(0.96,1.03)	
	1983-1993 (current age: 30-39 years)	1.000	0.018	(0.97,1.04)	
	1993-1998 (current age: 25-29 years)	0.982	0.021	(0.94,1.02)	
Religion when twelve years old	(Ref: No religion/Atheist/Agnostic)				0.259
	Christianity	0.985	0.010	(0.97,1.01)	
	Collapsed affiliations with prevalence<3%	0.966	0.029	(0.91,1.02)	

Table S19e. Sweden: Sensitivity to unmeasured confounding of childhood predictors

Variable	Category	E-value for Estimate	E-value for 95% CI
Relationship with your mother growing up	(Ref: Very bad/somewhat bad)		
	Very good/somewhat good	1.07	1.00
Relationship with your father growing up	(Ref: Very bad/somewhat bad)		
	Very good/somewhat good	1.11	1.00
Parents married to each other when you were around 12 years old	(Ref: Parents married)		
	One or both of them had died	1.19	1.00
	Parents were divorced	1.15	1.00
	Parents were never married	1.07	1.00
	Unsure	1.18	1.00
Feelings about familys household income when growing up	(Ref: Got by)		
	Found it difficult	1.16	1.00
	Found it very difficult	1.13	1.00
	Lived comfortably	1.20	1.11
Physically or sexually abused when growing up	(Ref: No)		
		1.21	1.11
Felt like an outsider in your family when growing up	(Ref: No)		
	Yes	1.16	1.00
Your health when growing up	(Ref: Good)		
	Excellent	1.06	1.00
	Fair	1.18	1.00
	Poor	1.16	1.00
	Very good	1.13	1.00
Born in This country	(Ref: Born in this country)		
	Born in another country	1.19	1.00
How Often You Attended Religious Services or Worshiped When You Were Around 12 Years Old	(Ref: Never)		
	At least once a week	1.47	1.37
	Less than once a month	1.39	1.34
	One to three times a month	1.36	1.25
Year of birth (age group)	(Ref: 1998-2005; current age: 18-24)		
	1943 or earlier (current age: 80+ years)	1.23	1.00
	1943-1953 (current age: 70-79 years)	1.09	1.00

Variable	Category	E-value for Estimate	E-value for 95% CI
	1953-1963 (current age: 60-69 years)	1.12	1.00
	1963-1973 (current age: 50-59 years)	1.11	1.00
	1973-1983 (current age: 40-49 years)	1.08	1.00
	1983-1993 (current age: 30-39 years)	1.01	1.00
	1993-1998 (current age: 25-29 years)	1.16	1.00
Religion when twelve years old	(Ref: No religion/Atheist/Agnostic)		
	Christianity	1.14	1.00
	Collapsed affiliations with prevalence<3%	1.23	1.00

Table S20a. Tanzania: Demographic descriptive statistics

Characteristic	N = 6,577[1]
**Age group**	
1943 or earlier (current age: 80+ years)	62 (0.9%)
1943-1953 (current age: 70-79 years)	195 (3.0%)
1953-1963 (current age: 60-69 years)	389 (5.9%)
1963-1973 (current age: 50-59 years)	618 (9.4%)
1973-1983 (current age: 40-49 years)	1,058 (16%)
1983-1993 (current age: 30-39 years)	1,446 (22%)
1993-1998 (current age: 25-29 years)	924 (14%)
1998-2005 (current age: 18-24 years)	1,884 (29%)
(Missing)	2 (<0.1%)
**Gender**	
1. Male	3,104 (47%)
2. Female	3,473 (53%)
3. Other	0 (0%)
(Missing)	0 (0%)
**Marital status**	
1. Single/Never been married	1,745 (27%)
2. Married	3,982 (61%)
3. Separated	274 (4.2%)
4. Divorced	72 (1.1%)
5. Widowed	302 (4.6%)
6. Domestic partner	196 (3.0%)
(Missing)	6 (<0.1%)
**Employment**	
1. Employed for an employer	341 (5.2%)
2. Self-employed	3,321 (50%)
3. Retired	86 (1.3%)
4. Student	257 (3.9%)
5. Homemaker	1,296 (20%)
6. Unemployed and looking for a job	1,138 (17%)
7. None of these/Other	133 (2.0%)
(Missing)	5 (<0.1%)
**Religious service attendance**	
1. More than once a week	1,825 (28%)
2. Once a week	3,134 (48%)
3. One to three times a month	766 (12%)
4. A few times a year	628 (9.5%)
5. Never	224 (3.4%)
(Missing)	0 (<0.1%)
**Education**	
1. Up to 8	4,966 (76%)
2. 9-15	1,545 (23%)
3. 16+	66 (1.0%)
(Missing)	0 (<0.1%)
**Immigration**	
1. Born in this country	6,562 (100%)
2. Born in another country	14 (0.2%)

Characteristic	N = 6,577[1]
(Missing)	2 (<0.1%)
**Religious affiliation**	
1. Christianity	4,051 (62%)
10. Taoism	1 (<0.1%)
11. Confucianism	0 (0%)
12. Primal, Animist, or Folk religion	10 (0.1%)
13. Spiritism	0 (0%)
14. Umbanda, Candomblé, and other African-derived religions	0 (0%)
15. Chinese folk/traditional religion	0 (0%)
2. Islam	2,327 (35%)
3. Hinduism	0 (0%)
4. Buddhism	0 (0%)
5. Judaism	0 (0%)
6. Sikhism	0 (0%)
7. Baha'i	0 (0%)
8. Jainism	0 (0%)
9. Shinto	0 (0%)
96. Some other religion	0 (0%)
97. No religion/Atheist/Agnostic	174 (2.6%)
(Missing)	13 (0.2%)
**RACE ETHNICITY**	
(Missing)	2 (<0.1%)
1801. Tanzania: African	6,562 (100%)
1802. Tanzania: Indian	2 (<0.1%)
1803. Tanzania: Arab	11 (0.2%)

[1]n (%)

Table S20b. Tanzania: Childhood descriptive statistics

**Characteristic**	**N = 6,577**[1]
**Age**	36 (15)
Unknown	2
**Year of birth**	
1943 or earlier (current age: 80+ years)	62 (0.9%)
1943-1953 (current age: 70-79 years)	195 (3.0%)
1953-1963 (current age: 60-69 years)	389 (5.9%)
1963-1973 (current age: 50-59 years)	618 (9.4%)
1973-1983 (current age: 40-49 years)	1,058 (16%)
1983-1993 (current age: 30-39 years)	1,446 (22%)
1993-1998 (current age: 25-29 years)	924 (14%)
1998-2005 (current age: 18-24 years)	1,884 (29%)
(Missing)	2 (<0.1%)
**Gender**	
1. Male	3,104 (47%)
2. Female	3,473 (53%)
3. Other	0 (0%)
(Missing)	0 (0%)
**Parent marital status**	
1. Parents were married	5,035 (77%)
2. Parents were divorced	514 (7.8%)
3. Parents were never married	585 (8.9%)
4. One or both of them had died	241 (3.7%)
5. Unsure	18 (0.3%)
(Missing)	184 (2.8%)
**Age 12 religious service attendance**	
1. At least once a week	4,020 (61%)
2. One to three times a month	1,738 (26%)
3. Less than once a month	247 (3.8%)
4. Never	446 (6.8%)
(Missing)	127 (1.9%)
**Relationship with mother**	
1. Very good	5,594 (85%)
2. Somewhat good	611 (9.3%)
3. Somewhat bad	60 (0.9%)
4. Very bad	60 (0.9%)
97. (Does not apply)	210 (3.2%)
(Missing)	42 (0.6%)
**Relationship with father**	
1. Very good	4,931 (75%)
2. Somewhat good	804 (12%)
3. Somewhat bad	146 (2.2%)
4. Very bad	202 (3.1%)
97. (Does not apply)	396 (6.0%)
(Missing)	98 (1.5%)
**Outsider growing up**	
1. Yes	493 (7.5%)
2. No	6,066 (92%)

Characteristic	N = 6,577[1]
(Missing)	18 (0.3%)
**Abuse**	
1. Yes	557 (8.5%)
2. No	6,001 (91%)
(Missing)	19 (0.3%)
**Self-rated health growing up**	
1. Excellent	1,776 (27%)
2. Very good	1,486 (23%)
3. Good	2,115 (32%)
4. Fair	839 (13%)
5. Poor	324 (4.9%)
(Missing)	37 (0.6%)
**Immigration status**	
1. Born in this country	6,562 (100%)
2. Born in another country	14 (0.2%)
(Missing)	2 (<0.1%)
**Subjective financial status of family growing up**	
1. Lived comfortably	1,954 (30%)
2. Got by	2,059 (31%)
3. Found it difficult	1,940 (29%)
4. Found it very difficult	594 (9.0%)
(Missing)	30 (0.5%)
**Religious affiliation**	
1. Christianity	4,056 (62%)
10. Taoism	0 (0%)
11. Confucianism	0 (0%)
12. Primal, Animist, or Folk religion	10 (0.2%)
13. Spiritism	0 (0%)
14. Umbanda, Candomblé, and other African-derived religions	0 (0%)
15. Chinese folk/traditional religion	0 (0%)
2. Islam	2,237 (34%)
3. Hinduism	0 (0%)
4. Buddhism	0 (0%)
5. Judaism	0 (0%)
6. Sikhism	0 (0%)
7. Baha'i	0 (0%)
8. Jainism	0 (0%)
9. Shinto	0 (0%)
96. Some other religion	0 (0%)
97. No religion/Atheist/Agnostic	269 (4.1%)
(Missing)	5 (<0.1%)
**Race/Ethnicity**	
(Missing)	2 (<0.1%)
1801. Tanzania: African	6,562 (100%)
1802. Tanzania: Indian	2 (<0.1%)
1803. Tanzania: Arab	11 (0.2%)

[1]Mean (SD); n (%)

Table S20c. Tanzania: Proportions by demographic category

Variable	Category	Proportion	95% CI	SE	p-value
Age group	1998-2005 (current age: 18-24 years)	0.644	(0.612, 0.677)	0.017	5.33e-15
	1943 or earlier (current age: 80+ years)	0.419	(0.251, 0.587)	0.084	
	1943-1953 (current age: 70-79 years)	0.401	(0.300, 0.502)	0.051	
	1953-1963 (current age: 60-69 years)	0.457	(0.392, 0.522)	0.033	
	1963-1973 (current age: 50-59 years)	0.488	(0.445, 0.532)	0.022	
	1973-1983 (current age: 40-49 years)	0.464	(0.427, 0.501)	0.019	
	1983-1993 (current age: 30-39 years)	0.514	(0.482, 0.547)	0.016	
	1993-1998 (current age: 25-29 years)	0.620	(0.579, 0.662)	0.021	
Gender	Male	0.597	(0.572, 0.622)	0.013	1.33e-08
	Female	0.505	(0.480, 0.530)	0.013	
	Other	*	*	*	
Marital status	Single/Never been married	0.656	(0.626, 0.686)	0.015	6.13e-12
	Divorced	0.370	(0.237, 0.502)	0.066	
	Domestic partner	0.511	(0.426, 0.596)	0.043	
	Married	0.513	(0.488, 0.537)	0.012	
	Separated	0.527	(0.458, 0.597)	0.035	
	Widowed	0.480	(0.403, 0.557)	0.039	
Employment	Employed for an employer	0.697	(0.638, 0.756)	0.030	< 1e-16
	Homemaker	0.444	(0.406, 0.481)	0.019	
	None of these/Other	0.412	(0.310, 0.515)	0.052	
	Retired	0.404	(0.294, 0.515)	0.055	
	Self-employed	0.550	(0.523, 0.576)	0.013	
	Student	0.757	(0.687, 0.827)	0.035	
	Unemployed and looking for a job	0.598	(0.561, 0.636)	0.019	
Religious service attendance	Never	0.434	(0.352, 0.515)	0.041	1.69e-02
	A few times a year	0.574	(0.525, 0.623)	0.025	
	More than once a week	0.562	(0.528, 0.596)	0.017	
	Once a week	0.552	(0.525, 0.579)	0.014	
	One to three times a month	0.514	(0.470, 0.558)	0.023	
Education	9-15	0.709	(0.683, 0.734)	0.013	< 1e-16
	16+	0.779	(0.673, 0.886)	0.053	

Variable	Category	Proportion	95% CI	SE	p-value
	Up to 8	0.495	(0.472, 0.519)	0.012	
Immigration status	Born in this country	0.548	(0.529, 0.568)	0.010	5.27e-01
	Born in another country	0.443	(0.066, 0.820)	0.167	
Religious affiliation	No religion/Atheist/Agnostic	*	*	*	7.52e-05
	Christianity	0.580	(0.556, 0.604)	0.012	
	Combined	0.447	(0.329, 0.566)	0.060	
	Spiritism	*	*	*	
	Umbanda, Candomblé, and other African-derived religions	*	*	*	
	Buddhism	*	*	*	
	Islam	0.501	(0.470, 0.533)	0.016	
	Chinese folk/traditional religion	*	*	*	
	Hinduism	*	*	*	
	Judaism	*	*	*	
	Primal, Animist, or Folk religion	*	*	*	

Table S20d. Tanzania: Childhood predictors regression analysis

Variable	Category	Risk-Ratio	logRR SE	RR 95% CI	Global p-value
Relationship with your mother growing up	(Ref: Very bad/somewhat bad)				0.596
	Very good/somewhat good	1.006	0.045	(0.92,1.10)	
Relationship with your father growing up	(Ref: Very bad/somewhat bad)				0.499
	Very good/somewhat good	1.021	0.035	(0.95,1.09)	
Parents married to each other when you were around 12 years old	(Ref: Parents married)				0.733
	One or both of them had died	0.978	0.040	(0.90,1.06)	
	Parents were divorced	0.992	0.031	(0.93,1.06)	
	Parents were never married	0.981	0.029	(0.93,1.04)	
	Unsure	0.916	0.146	(0.69,1.22)	
Feelings about familys household income when growing up	(Ref: Got by)				0.002
	Found it difficult	0.964	0.021	(0.93,1.00)	
	Found it very difficult	0.905	0.029	(0.85,0.96)	
	Lived comfortably	1.024	0.019	(0.99,1.06)	
Physically or sexually abused when growing up	(Ref: No)				0.045
		1.064	0.030	(1.00,1.13)	
Felt like an outsider in your family when growing up	(Ref: No)				0.506
	Yes	1.015	0.027	(0.96,1.07)	
Your health when growing up	(Ref: Good)				0.012
	Excellent	1.072	0.024	(1.02,1.12)	
	Fair	1.054	0.025	(1.00,1.11)	
	Poor	0.947	0.038	(0.88,1.02)	
	Very good	1.026	0.022	(0.98,1.07)	
Born in This	(Ref: Born in this				0.375

Variable	Category	Risk-Ratio	logRR SE	RR 95% CI	Global p-value
country	country)				
	Born in another country	0.860	0.158	(0.63,1.17)	
How Often You Attended Religious Services or Worshiped When You Were Around 12 Years Old	(Ref: Never)				0.001
	At least once a week	1.156	0.042	(1.06,1.26)	
	Less than once a month	1.188	0.054	(1.07,1.32)	
	One to three times a month	1.143	0.045	(1.05,1.25)	
Year of birth (age group)	(Ref: 1998-2005; current age: 18-24)				2.69e-11
	1943 or earlier (current age: 80+ years)	0.801	0.083	(0.68,0.94)	
	1943-1953 (current age: 70-79 years)	0.798	0.053	(0.72,0.89)	
	1953-1963 (current age: 60-69 years)	0.839	0.036	(0.78,0.90)	
	1963-1973 (current age: 50-59 years)	0.876	0.028	(0.83,0.92)	
	1973-1983 (current age: 40-49 years)	0.852	0.023	(0.81,0.89)	
	1983-1993 (current age: 30-39 years)	0.890	0.022	(0.85,0.93)	
	1993-1998 (current age: 25-29 years)	0.977	0.028	(0.92,1.03)	
Religion when twelve years old	(Ref: No religion/Atheist/Agnostic)				0.662
	Christianity	0.988	0.065	(0.87,1.12)	
	Collapsed affiliations with prevalence<3%	1.005	0.194	(0.69,1.47)	
	Islam	0.935	0.065	(0.82,1.06)	
Race plurality (prominent race/ethnic group [0] or not [1])	(Ref: Plurality group)				0.408
	Non-plurality groups	0.849	0.190	(0.59,1.23)	

Table S20e. Tanzania: Sensitivity to unmeasured confounding of childhood predictors

Variable	Category	E-value for Estimate	E-value for 95% CI
Relationship with your mother growing up	(Ref: Very bad/somewhat bad)		
	Very good/somewhat good	1.08	1.00
Relationship with your father growing up	(Ref: Very bad/somewhat bad)		
	Very good/somewhat good	1.17	1.00
Parents married to each other when you were around 12 years old	(Ref: Parents married)		
	One or both of them had died	1.18	1.00
	Parents were divorced	1.10	1.00
	Parents were never married	1.16	1.00
	Unsure	1.41	1.00
Feelings about familys household income when growing up	(Ref: Got by)		
	Found it difficult	1.23	1.00
	Found it very difficult	1.45	1.25
	Lived comfortably	1.18	1.00
Physically or sexually abused when growing up	(Ref: No)		
		1.32	1.06
Felt like an outsider in your family when growing up	(Ref: No)		
	Yes	1.14	1.00
Your health when growing up	(Ref: Good)		
	Excellent	1.35	1.18
	Fair	1.29	1.07
	Poor	1.30	1.00
	Very good	1.19	1.00
Born in This country	(Ref: Born in this country)		
	Born in another country	1.60	1.00
How Often You Attended Religious Services or Worshiped When You Were Around 12 Years Old	(Ref: Never)		
	At least once a week	1.58	1.33
	Less than once a month	1.66	1.34
	One to three times a month	1.55	1.27
Year of birth (age group)	(Ref: 1998-2005; current age: 18-24)		
	1943 or earlier (current age: 80+ years)	1.80	1.32
	1943-1953 (current age: 70-79 years)	1.82	1.51

Variable	Category	E-value for Estimate	E-value for 95% CI
	1953-1963 (current age: 60-69 years)	1.67	1.46
	1963-1973 (current age: 50-59 years)	1.54	1.38
	1973-1983 (current age: 40-49 years)	1.63	1.49
	1983-1993 (current age: 30-39 years)	1.50	1.36
	1993-1998 (current age: 25-29 years)	1.18	1.00
Religion when twelve years old	(Ref: No religion/Atheist/Agnostic)		
	Christianity	1.12	1.00
	Collapsed affiliations with prevalence<3%	1.07	1.00
	Islam	1.34	1.00
Race plurality (prominent race/ethnic group [0] or not [1])	(Ref: Plurality group)		
	Non-plurality groups	1.64	1.00

Table S21a. Turkey: Demographic descriptive statistics

**Characteristic**	**N = 659**[1]
**Age group**	
1943 or earlier (current age: 80+ years)	10 (1.5%)
1943-1953 (current age: 70-79 years)	38 (5.8%)
1953-1963 (current age: 60-69 years)	67 (10%)
1963-1973 (current age: 50-59 years)	100 (15%)
1973-1983 (current age: 40-49 years)	124 (19%)
1983-1993 (current age: 30-39 years)	144 (22%)
1993-1998 (current age: 25-29 years)	72 (11%)
1998-2005 (current age: 18-24 years)	104 (16%)
(Missing)	0 (0%)
**Gender**	
1. Male	330 (50%)
2. Female	329 (50%)
3. Other	0 (0%)
(Missing)	0 (0%)
**Marital status**	
1. Single/Never been married	168 (26%)
2. Married	421 (64%)
3. Separated	5 (0.8%)
4. Divorced	29 (4.4%)
5. Widowed	32 (4.9%)
6. Domestic partner	0 (0%)
(Missing)	3 (0.4%)
**Employment**	
1. Employed for an employer	187 (28%)
2. Self-employed	103 (16%)
3. Retired	88 (13%)
4. Student	53 (8.0%)
5. Homemaker	157 (24%)
6. Unemployed and looking for a job	38 (5.8%)
7. None of these/Other	32 (4.9%)
(Missing)	0 (0%)
**Religious service attendance**	
1. More than once a week	224 (34%)
2. Once a week	129 (20%)
3. One to three times a month	70 (11%)
4. A few times a year	104 (16%)
5. Never	129 (20%)
(Missing)	3 (0.4%)
**Education**	
1. Up to 8	200 (30%)
2. 9-15	329 (50%)
3. 16+	130 (20%)
(Missing)	0 (0%)
**Immigration**	
1. Born in this country	621 (94%)
2. Born in another country	38 (5.8%)

Characteristic	N = 659[1]
(Missing)	0 (0%)
**Religious affiliation**	
1. Christianity	1 (0.2%)
10. Taoism	0 (0%)
11. Confucianism	0 (0%)
12. Primal, Animist, or Folk religion	0 (<0.1%)
13. Spiritism	0 (0%)
14. Umbanda, Candomblé, and other African-derived religions	0 (0%)
15. Chinese folk/traditional religion	0 (0%)
2. Islam	618 (94%)
3. Hinduism	0 (0%)
4. Buddhism	0 (<0.1%)
5. Judaism	1 (0.1%)
6. Sikhism	1 (<0.1%)
7. Baha'i	0 (0%)
8. Jainism	0 (0%)
9. Shinto	0 (0%)
96. Some other religion	0 (0%)
97. No religion/Atheist/Agnostic	30 (4.5%)
(Missing)	8 (1.2%)
**RACE ETHNICITY**	
(Missing)	4 (0.6%)
1901. Turkey: Turkish	435 (66%)
1902. Turkey: Kurdish/Zaza	112 (17%)
1903. Turkey: Arab	33 (5.0%)
1904. Turkey: Laz	21 (3.1%)
1905. Turkey: Circassian	7 (1.0%)
1906. Turkey: Bosnian	1 (0.2%)
1907. Turkey: Armenian	1 (0.1%)
1908. Turkey: Georgian	1 (0.2%)
1911. Turkey: Albanian	2 (0.4%)
1913. Turkey: Azeri	5 (0.8%)
9996. Other	36 (5.5%)

[1]n (%)

Table S21b. Turkey: Childhood descriptive statistics

**Characteristic**	**N = 659**[1]
**Age**	42 (16)
**Year of birth**	
1943 or earlier (current age: 80+ years)	10 (1.5%)
1943-1953 (current age: 70-79 years)	38 (5.8%)
1953-1963 (current age: 60-69 years)	67 (10%)
1963-1973 (current age: 50-59 years)	100 (15%)
1973-1983 (current age: 40-49 years)	124 (19%)
1983-1993 (current age: 30-39 years)	144 (22%)
1993-1998 (current age: 25-29 years)	72 (11%)
1998-2005 (current age: 18-24 years)	104 (16%)
(Missing)	0 (0%)
**Gender**	
1. Male	330 (50%)
2. Female	329 (50%)
3. Other	0 (0%)
(Missing)	0 (0%)
**Parent marital status**	
1. Parents were married	595 (90%)
2. Parents were divorced	30 (4.5%)
3. Parents were never married	2 (0.3%)
4. One or both of them had died	27 (4.1%)
5. Unsure	3 (0.4%)
(Missing)	2 (0.3%)
**Age 12 religious service attendance**	
1. At least once a week	263 (40%)
2. One to three times a month	118 (18%)
3. Less than once a month	88 (13%)
4. Never	185 (28%)
(Missing)	5 (0.7%)
**Relationship with mother**	
1. Very good	429 (65%)
2. Somewhat good	190 (29%)
3. Somewhat bad	17 (2.6%)
4. Very bad	8 (1.3%)
97. (Does not apply)	13 (2.0%)
(Missing)	2 (0.3%)
**Relationship with father**	
1. Very good	343 (52%)
2. Somewhat good	188 (29%)
3. Somewhat bad	30 (4.5%)
4. Very bad	53 (8.1%)
97. (Does not apply)	30 (4.5%)
(Missing)	15 (2.3%)
**Outsider growing up**	
1. Yes	72 (11%)
2. No	580 (88%)
(Missing)	7 (1.1%)

Characteristic	N = 659[1]
**Abuse**	
1. Yes	70 (11%)
2. No	581 (88%)
(Missing)	7 (1.1%)
**Self-rated health growing up**	
1. Excellent	144 (22%)
2. Very good	202 (31%)
3. Good	183 (28%)
4. Fair	109 (16%)
5. Poor	21 (3.2%)
(Missing)	1 (0.1%)
**Immigration status**	
1. Born in this country	621 (94%)
2. Born in another country	38 (5.8%)
(Missing)	0 (0%)
**Subjective financial status of family growing up**	
1. Lived comfortably	233 (35%)
2. Got by	287 (43%)
3. Found it difficult	84 (13%)
4. Found it very difficult	55 (8.3%)
(Missing)	0 (0%)
**Religious affiliation**	
1. Christianity	1 (0.1%)
10. Taoism	0 (0%)
11. Confucianism	0 (0%)
12. Primal, Animist, or Folk religion	0 (0%)
13. Spiritism	0 (0%)
14. Umbanda, Candomblé, and other African-derived religions	0 (0%)
15. Chinese folk/traditional religion	0 (0%)
2. Islam	647 (98%)
3. Hinduism	0 (0%)
4. Buddhism	0 (0%)
5. Judaism	1 (0.1%)
6. Sikhism	0 (0%)
7. Baha'i	0 (0%)
8. Jainism	0 (0%)
9. Shinto	0 (0%)
96. Some other religion	0 (0%)
97. No religion/Atheist/Agnostic	3 (0.5%)
(Missing)	7 (1.1%)
**Race/Ethnicity**	
(Missing)	4 (0.6%)
1901. Turkey: Turkish	435 (66%)
1902. Turkey: Kurdish/Zaza	112 (17%)
1903. Turkey: Arab	33 (5.0%)
1904. Turkey: Laz	21 (3.1%)
1905. Turkey: Circassian	7 (1.0%)

Characteristic	N = 659[1]
1906. Turkey: Bosnian	1 (0.2%)
1907. Turkey: Armenian	1 (0.1%)
1908. Turkey: Georgian	1 (0.2%)
1911. Turkey: Albanian	2 (0.4%)
1913. Turkey: Azeri	5 (0.8%)
9996. Other	36 (5.5%)

[1]Mean (SD); n (%)

Table S21c. Turkey: Proportions by demographic category

Variable	Category	Proportion	95% CI	SE	p-value
Age group	1998-2005 (current age: 18-24 years)	0.749	(0.640, 0.858)	0.055	4.89e-01
	1943 or earlier (current age: 80+ years)	0.826	*	*	
	1943-1953 (current age: 70-79 years)	0.529	(0.253, 0.805)	0.135	
	1953-1963 (current age: 60-69 years)	0.712	(0.553, 0.870)	0.079	
	1963-1973 (current age: 50-59 years)	0.688	(0.576, 0.801)	0.057	
	1973-1983 (current age: 40-49 years)	0.786	(0.704, 0.868)	0.042	
	1983-1993 (current age: 30-39 years)	0.701	(0.618, 0.783)	0.042	
	1993-1998 (current age: 25-29 years)	0.781	(0.665, 0.896)	0.058	
Gender	Male	0.706	(0.649, 0.763)	0.029	4.16e-01
	Female	0.742	(0.675, 0.809)	0.034	
	Other	*	*	*	
Marital status	Single/Never been married	0.737	(0.664, 0.811)	0.037	7.69e-01
	Divorced	0.594	(0.360, 0.828)	0.113	
	Domestic partner	*	*	*	
	Married	0.731	(0.676, 0.785)	0.028	
	Separated	0.567	*	*	
	Widowed	0.710	(0.441, 0.978)	0.130	
Employment	Employed for an employer	0.717	(0.644, 0.790)	0.037	9.06e-01
	Homemaker	0.743	(0.643, 0.842)	0.050	
	None of these/Other	0.638	(0.417, 0.859)	0.107	
	Retired	0.753	(0.621, 0.886)	0.066	
	Self-employed	0.706	(0.600, 0.812)	0.053	
	Student	0.692	(0.540, 0.844)	0.075	
	Unemployed and looking for a job	0.780	(0.634, 0.926)	0.071	
Religious service attendance	Never	0.651	(0.546, 0.757)	0.053	2.77e-02
	A few times a year	0.700	(0.591, 0.809)	0.055	
	More than once a week	0.721	(0.647, 0.795)	0.038	
	Once a week	0.838	(0.765, 0.911)	0.037	
	One to three times a month	0.693	(0.545, 0.841)	0.074	
Education	9-15	0.730	(0.667, 0.792)	0.032	5.15e-01
	16+	0.756	(0.698, 0.814)	0.030	

Variable	Category	Proportion	95% CI	SE	p-value
	Up to 8	0.694	(0.599, 0.788)	0.048	
Immigration status	Born in this country	0.725	(0.680, 0.770)	0.023	8.06e-01
	Born in another country	0.706	(0.494, 0.918)	0.104	
Religious affiliation	No religion/Atheist/Agnostic	0.634	(0.417, 0.851)	0.105	< 1e-16
	Christianity	*	*	*	
	Combined	0.985	*	*	
	Spiritism	*	*	*	
	Umbanda, Candomblé, and other African-derived religions	*	*	*	
	Buddhism	*	*	*	
	Islam	0.727	(0.682, 0.772)	0.023	
	Chinese folk/traditional religion	*	*	*	
	Hinduism	*	*	*	
	Judaism	*	*	*	
	Primal, Animist, or Folk religion	*	*	*	

Table S21d. Turkey: Childhood predictors regression analysis

Variable	Category	Risk-Ratio	logRR SE	RR 95% CI	Global p-value
Relationship with your mother growing up	(Ref: Very bad/somewhat bad)				0.507
	Very good/somewhat good	1.064	0.113	(0.85,1.33)	
Relationship with your father growing up	(Ref: Very bad/somewhat bad)				0.608
	Very good/somewhat good	0.997	0.070	(0.87,1.14)	
Parents married to each other when you were around 12 years old	(Ref: Parents married)				5.35e-06
	One or both of them had died	1.113	0.168	(0.80,1.55)	
	Parents were divorced	1.097	0.099	(0.90,1.33)	
	Parents were never married	1.352	0.062	(1.20,1.53)	
	Unsure	0.914	0.256	(0.55,1.51)	
Feelings about familys household income when growing up	(Ref: Got by)				0.060
	Found it difficult	0.896	0.070	(0.78,1.03)	
	Found it very difficult	0.809	0.090	(0.68,0.97)	
	Lived comfortably	0.988	0.047	(0.90,1.08)	
Physically or sexually abused when growing up	(Ref: No)				0.449
		1.052	0.068	(0.92,1.20)	
Felt like an outsider in your family when growing up	(Ref: No)				0.586
	Yes	1.013	0.066	(0.89,1.15)	
Your health when growing up	(Ref: Good)				0.222
	Excellent	0.992	0.060	(0.88,1.12)	
	Fair	0.866	0.070	(0.75,0.99)	
	Poor	1.008	0.114	(0.81,1.26)	
	Very good	1.015	0.058	(0.91,1.14)	
Born in This	(Ref: Born in this				0.606

Variable	Category	Risk-Ratio	logRR SE	RR 95% CI	Global p-value
country	country)				
	Born in another country	0.994	0.105	(0.81,1.22)	
How Often You Attended Religious Services or Worshiped When You Were Around 12 Years Old	(Ref: Never)				0.739
	At least once a week	0.977	0.054	(0.88,1.09)	
	Less than once a month	1.027	0.064	(0.91,1.17)	
	One to three times a month	1.011	0.061	(0.90,1.14)	
Year of birth (age group)	(Ref: 1998-2005; current age: 18-24)				0.665
	1943 or earlier (current age: 80+ years)	1.085	0.166	(0.78,1.50)	
	1943-1953 (current age: 70-79 years)	0.809	0.135	(0.62,1.05)	
	1953-1963 (current age: 60-69 years)	0.997	0.097	(0.82,1.21)	
	1963-1973 (current age: 50-59 years)	0.983	0.079	(0.84,1.15)	
	1973-1983 (current age: 40-49 years)	1.074	0.074	(0.93,1.24)	
	1983-1993 (current age: 30-39 years)	0.978	0.074	(0.85,1.13)	
	1993-1998 (current age: 25-29 years)	1.049	0.080	(0.90,1.23)	
Religion when twelve years old	(Ref:)				8.68e-04
	Collapsed affiliations with prevalence<3%	1.244	0.065	(1.10,1.41)	

Table S21e. Turkey: Sensitivity to unmeasured confounding of childhood predictors

Variable	Category	E-value for Estimate	E-value for 95% CI
Relationship with your mother growing up	(Ref: Very bad/somewhat bad)		
	Very good/somewhat good	1.33	1.00
Relationship with your father growing up	(Ref: Very bad/somewhat bad)		
	Very good/somewhat good	1.06	1.00
Parents married to each other when you were around 12 years old	(Ref: Parents married)		
	One or both of them had died	1.47	1.00
	Parents were divorced	1.42	1.00
	Parents were never married	2.04	1.68
	Unsure	1.41	1.00
Feelings about familys household income when growing up	(Ref: Got by)		
	Found it difficult	1.47	1.00
	Found it very difficult	1.77	1.23
	Lived comfortably	1.12	1.00
Physically or sexually abused when growing up	(Ref: No)		
		1.29	1.00
Felt like an outsider in your family when growing up	(Ref: No)		
	Yes	1.13	1.00
Your health when growing up	(Ref: Good)		
	Excellent	1.10	1.00
	Fair	1.58	1.08
	Poor	1.10	1.00
	Very good	1.14	1.00
Born in This country	(Ref: Born in this country)		
	Born in another country	1.09	1.00
How Often You Attended Religious Services or Worshiped When You Were Around 12 Years Old	(Ref: Never)		
	At least once a week	1.18	1.00
	Less than once a month	1.19	1.00
	One to three times a month	1.12	1.00
Year of birth (age group)	(Ref: 1998-2005; current age: 18-24)		
	1943 or earlier (current age: 80+ years)	1.39	1.00
	1943-1953 (current age: 70-79 years)	1.78	1.00

Variable	Category	E-value for Estimate	E-value for 95% CI
	1953-1963 (current age: 60-69 years)	1.06	1.00
	1963-1973 (current age: 50-59 years)	1.15	1.00
	1973-1983 (current age: 40-49 years)	1.35	1.00
	1983-1993 (current age: 30-39 years)	1.17	1.00
	1993-1998 (current age: 25-29 years)	1.27	1.00
Religion when twelve years old	(Ref:)		
	Collapsed affiliations with prevalence<3%	1.80	1.42

Table S22a. United Kingdom: Demographic descriptive statistics

Characteristic	N = 3,354[1]
**Age group**	
1943 or earlier (current age: 80+ years)	91 (2.7%)
1943-1953 (current age: 70-79 years)	451 (13%)
1953-1963 (current age: 60-69 years)	564 (17%)
1963-1973 (current age: 50-59 years)	683 (20%)
1973-1983 (current age: 40-49 years)	577 (17%)
1983-1993 (current age: 30-39 years)	541 (16%)
1993-1998 (current age: 25-29 years)	237 (7.1%)
1998-2005 (current age: 18-24 years)	209 (6.2%)
(Missing)	1 (<0.1%)
**Gender**	
1. Male	1,587 (47%)
2. Female	1,754 (52%)
3. Other	9 (0.3%)
(Missing)	4 (0.1%)
**Marital status**	
1. Single/Never been married	888 (26%)
2. Married	1,563 (47%)
3. Separated	60 (1.8%)
4. Divorced	282 (8.4%)
5. Widowed	174 (5.2%)
6. Domestic partner	363 (11%)
(Missing)	24 (0.7%)
**Employment**	
1. Employed for an employer	1,833 (55%)
2. Self-employed	268 (8.0%)
3. Retired	791 (24%)
4. Student	99 (2.9%)
5. Homemaker	126 (3.7%)
6. Unemployed and looking for a job	113 (3.4%)
7. None of these/Other	117 (3.5%)
(Missing)	7 (0.2%)
**Religious service attendance**	
1. More than once a week	130 (3.9%)
2. Once a week	277 (8.3%)
3. One to three times a month	187 (5.6%)
4. A few times a year	699 (21%)
5. Never	2,052 (61%)
(Missing)	9 (0.3%)
**Education**	
1. Up to 8	739 (22%)
2. 9-15	1,403 (42%)
3. 16+	1,210 (36%)
(Missing)	2 (<0.1%)
**Immigration**	
1. Born in this country	2,931 (87%)
2. Born in another country	418 (12%)

Characteristic	N = 3,354[1]
(Missing)	5 (0.2%)
**Religious affiliation**	
1. Christianity	1,657 (49%)
10. Taoism	4 (0.1%)
11. Confucianism	3 (<0.1%)
12. Primal, Animist, or Folk religion	16 (0.5%)
13. Spiritism	0 (0%)
14. Umbanda, Candomblé, and other African-derived religions	0 (0%)
15. Chinese folk/traditional religion	0 (0%)
2. Islam	129 (3.8%)
3. Hinduism	31 (0.9%)
4. Buddhism	16 (0.5%)
5. Judaism	27 (0.8%)
6. Sikhism	9 (0.3%)
7. Baha'i	1 (<0.1%)
8. Jainism	0 (0%)
9. Shinto	0 (0%)
96. Some other religion	53 (1.6%)
97. No religion/Atheist/Agnostic	1,393 (42%)
(Missing)	16 (0.5%)
**RACE ETHNICITY**	
(Missing)	9 (0.3%)
2001. United Kingdom: Asian	214 (6.4%)
2002. United Kingdom: Black	72 (2.2%)
2003. United Kingdom: White	2,979 (89%)
9995. Prefer not to answer	22 (0.6%)
9996. Other	58 (1.7%)

[1]n (%)

Table S22b. United Kingdom: Childhood descriptive statistics

Characteristic	N = 3,354[1]
**Age**	50 (17)
**Year of birth**	
1943 or earlier (current age: 80+ years)	91 (2.7%)
1943-1953 (current age: 70-79 years)	451 (13%)
1953-1963 (current age: 60-69 years)	564 (17%)
1963-1973 (current age: 50-59 years)	683 (20%)
1973-1983 (current age: 40-49 years)	577 (17%)
1983-1993 (current age: 30-39 years)	541 (16%)
1993-1998 (current age: 25-29 years)	237 (7.1%)
1998-2005 (current age: 18-24 years)	209 (6.2%)
(Missing)	1 (<0.1%)
**Gender**	
1. Male	1,587 (47%)
2. Female	1,754 (52%)
3. Other	9 (0.3%)
(Missing)	4 (0.1%)
**Parent marital status**	
1. Parents were married	2,731 (81%)
2. Parents were divorced	323 (9.6%)
3. Parents were never married	163 (4.8%)
4. One or both of them had died	106 (3.2%)
5. Unsure	29 (0.9%)
(Missing)	3 (<0.1%)
**Age 12 religious service attendance**	
1. At least once a week	964 (29%)
2. One to three times a month	468 (14%)
3. Less than once a month	595 (18%)
4. Never	1,310 (39%)
(Missing)	17 (0.5%)
**Relationship with mother**	
1. Very good	1,993 (59%)
2. Somewhat good	955 (28%)
3. Somewhat bad	230 (6.8%)
4. Very bad	91 (2.7%)
97. (Does not apply)	73 (2.2%)
(Missing)	13 (0.4%)
**Relationship with father**	
1. Very good	1,735 (52%)
2. Somewhat good	906 (27%)
3. Somewhat bad	286 (8.5%)
4. Very bad	196 (5.8%)
97. (Does not apply)	219 (6.5%)
(Missing)	13 (0.4%)
**Outsider growing up**	
1. Yes	635 (19%)
2. No	2,690 (80%)
(Missing)	29 (0.9%)

Characteristic	N = 3,354[1]
**Abuse**	
1. Yes	513 (15%)
2. No	2,830 (84%)
(Missing)	11 (0.3%)
**Self-rated health growing up**	
1. Excellent	1,372 (41%)
2. Very good	1,057 (32%)
3. Good	607 (18%)
4. Fair	228 (6.8%)
5. Poor	77 (2.3%)
(Missing)	13 (0.4%)
**Immigration status**	
1. Born in this country	2,931 (87%)
2. Born in another country	418 (12%)
(Missing)	5 (0.2%)
**Subjective financial status of family growing up**	
1. Lived comfortably	1,461 (44%)
2. Got by	1,288 (38%)
3. Found it difficult	417 (12%)
4. Found it very difficult	171 (5.1%)
(Missing)	17 (0.5%)
**Religious affiliation**	
1. Christianity	2,175 (65%)
10. Taoism	0 (<0.1%)
11. Confucianism	3 (<0.1%)
12. Primal, Animist, or Folk religion	10 (0.3%)
13. Spiritism	0 (0%)
14. Umbanda, Candomblé, and other African-derived religions	0 (0%)
15. Chinese folk/traditional religion	0 (0%)
2. Islam	131 (3.9%)
3. Hinduism	44 (1.3%)
4. Buddhism	6 (0.2%)
5. Judaism	31 (0.9%)
6. Sikhism	9 (0.3%)
7. Baha'i	1 (<0.1%)
8. Jainism	0 (<0.1%)
9. Shinto	0 (0%)
96. Some other religion	25 (0.7%)
97. No religion/Atheist/Agnostic	900 (27%)
(Missing)	18 (0.5%)
**Race/Ethnicity**	
(Missing)	9 (0.3%)
2001. United Kingdom: Asian	214 (6.4%)
2002. United Kingdom: Black	72 (2.2%)
2003. United Kingdom: White	2,979 (89%)
9995. Prefer not to answer	22 (0.6%)
9996. Other	58 (1.7%)

Characteristic	N = 3,354[1]

[1]Mean (SD); n (%)

Table S22c. United Kingdom: Proportions by demographic category

Variable	Category	Proportion	95% CI	SE	p-value
Age group	1998-2005 (current age: 18-24 years)	0.718	(0.603, 0.832)	0.058	1.45e-01
	1943 or earlier (current age: 80+ years)	0.825	(0.713, 0.938)	0.056	
	1943-1953 (current age: 70-79 years)	0.658	(0.592, 0.725)	0.034	
	1953-1963 (current age: 60-69 years)	0.725	(0.676, 0.774)	0.025	
	1963-1973 (current age: 50-59 years)	0.743	(0.699, 0.788)	0.022	
	1973-1983 (current age: 40-49 years)	0.706	(0.654, 0.758)	0.026	
	1983-1993 (current age: 30-39 years)	0.738	(0.681, 0.794)	0.029	
	1993-1998 (current age: 25-29 years)	0.793	(0.709, 0.876)	0.042	
Gender	Male	0.713	(0.681, 0.746)	0.017	4.83e-01
	Female	0.738	(0.707, 0.768)	0.015	
	Other	0.565	(0.000, 1.000)	0.310	
Marital status	Single/Never been married	0.707	(0.660, 0.753)	0.024	2.40e-01
	Divorced	0.691	(0.611, 0.772)	0.041	
	Domestic partner	0.748	(0.682, 0.814)	0.033	
	Married	0.733	(0.701, 0.764)	0.016	
	Separated	0.834	(0.732, 0.937)	0.051	
	Widowed	0.733	(0.638, 0.827)	0.048	
Employment	Employed for an employer	0.735	(0.706, 0.765)	0.015	3.03e-03
	Homemaker	0.661	(0.540, 0.781)	0.061	
	None of these/Other	0.628	(0.518, 0.739)	0.056	
	Retired	0.707	(0.661, 0.754)	0.024	
	Self-employed	0.838	(0.776, 0.901)	0.032	
	Student	0.698	(0.530, 0.865)	0.084	
	Unemployed and looking for a job	0.624	(0.483, 0.765)	0.071	
Religious service attendance	Never	0.674	(0.645, 0.703)	0.015	1.61e-09
	A few times a year	0.799	(0.755, 0.842)	0.022	
	More than once a week	0.910	(0.829, 0.990)	0.041	
	Once a week	0.792	(0.715, 0.868)	0.039	
	One to three times a month	0.794	(0.701, 0.887)	0.047	
Education	9-15	0.713	(0.682, 0.744)	0.016	3.77e-12
	16+	0.821	(0.793, 0.850)	0.014	

Variable	Category	Proportion	95% CI	SE	p-value
	Up to 8	0.592	(0.528, 0.656)	0.033	
Immigration status	Born in this country	0.715	(0.691, 0.738)	0.012	5.26e-03
	Born in another country	0.803	(0.745, 0.861)	0.029	
Religious affiliation	No religion/Atheist/Agnostic	0.717	(0.682, 0.751)	0.017	7.88e-01
	Christianity	0.733	(0.702, 0.764)	0.016	
	Combined	0.728	(0.648, 0.808)	0.041	
	Spiritism	*	*	*	
	Umbanda, Candomblé, and other African-derived religions	*	*	*	
	Buddhism	*	*	*	
	Islam	*	*	*	
	Chinese folk/traditional religion	*	*	*	
	Hinduism	*	*	*	
	Judaism	*	*	*	
	Primal, Animist, or Folk religion	*	*	*	

Table S22d. United Kingdom: Childhood predictors regression analysis

Variable	Category	Risk-Ratio	logRR SE	RR 95% CI	Global p-value
Relationship with your mother growing up	(Ref: Very bad/somewhat bad)				0.549
	Very good/somewhat good	1.015	0.038	(0.94,1.09)	
Relationship with your father growing up	(Ref: Very bad/somewhat bad)				0.572
	Very good/somewhat good	1.009	0.033	(0.95,1.08)	
Parents married to each other when you were around 12 years old	(Ref: Parents married)				0.737
	One or both of them had died	1.066	0.070	(0.93,1.22)	
	Parents were divorced	1.015	0.037	(0.94,1.09)	
	Parents were never married	0.992	0.063	(0.88,1.12)	
	Unsure	0.952	0.157	(0.70,1.30)	
Feelings about familys household income when growing up	(Ref: Got by)				0.041
	Found it difficult	1.026	0.035	(0.96,1.10)	
	Found it very difficult	0.994	0.056	(0.89,1.11)	
	Lived comfortably	1.063	0.024	(1.01,1.12)	
Physically or sexually abused when growing up	(Ref: No)				0.225
		1.043	0.032	(0.98,1.11)	
Felt like an outsider in your family when growing up	(Ref: No)				0.158
	Yes	0.952	0.032	(0.89,1.01)	
Your health when growing up	(Ref: Good)				0.655
	Excellent	1.014	0.032	(0.95,1.08)	
	Fair	0.946	0.052	(0.86,1.05)	
	Poor	1.045	0.078	(0.90,1.22)	
	Very good	0.980	0.033	(0.92,1.05)	
Born in This	(Ref: Born in this				0.046

Variable	Category	Risk-Ratio	logRR SE	RR 95% CI	Global p-value
country	country)				
	Born in another country	1.067	0.031	(1.00,1.13)	
How Often You Attended Religious Services or Worshiped When You Were Around 12 Years Old	(Ref: Never)				1.58e-06
	At least once a week	1.159	0.030	(1.09,1.23)	
	Less than once a month	1.162	0.031	(1.09,1.23)	
	One to three times a month	1.142	0.039	(1.06,1.23)	
Year of birth (age group)	(Ref: 1998-2005; current age: 18-24)				0.809
	1943 or earlier (current age: 80+ years)	1.069	0.080	(0.91,1.25)	
	1943-1953 (current age: 70-79 years)	0.938	0.066	(0.82,1.07)	
	1953-1963 (current age: 60-69 years)	1.008	0.062	(0.89,1.14)	
	1963-1973 (current age: 50-59 years)	1.025	0.061	(0.91,1.16)	
	1973-1983 (current age: 40-49 years)	0.982	0.063	(0.87,1.11)	
	1983-1993 (current age: 30-39 years)	1.002	0.064	(0.89,1.14)	
	1993-1998 (current age: 25-29 years)	1.061	0.071	(0.92,1.22)	
Religion when twelve years old	(Ref: No religion/Atheist/Agnostic)				0.044
	Christianity	0.939	0.028	(0.89,0.99)	
	Collapsed affiliations with prevalence<3%	0.884	0.066	(0.78,1.01)	
	Islam	0.850	0.080	(0.73,0.99)	
Race plurality (prominent race/ethnic group [0] or not [1])	(Ref: Plurality group)				0.537
	Non-plurality groups	0.981	0.045	(0.90,1.07)	

Table S22e. United Kingdom: Sensitivity to unmeasured confounding of childhood predictors

Variable	Category	E-value for Estimate	E-value for 95% CI
Relationship with your mother growing up	(Ref: Very bad/somewhat bad)		
	Very good/somewhat good	1.14	1.00
Relationship with your father growing up	(Ref: Very bad/somewhat bad)		
	Very good/somewhat good	1.10	1.00
Parents married to each other when you were around 12 years old	(Ref: Parents married)		
	One or both of them had died	1.33	1.00
	Parents were divorced	1.14	1.00
	Parents were never married	1.10	1.00
	Unsure	1.28	1.00
Feelings about familys household income when growing up	(Ref: Got by)		
	Found it difficult	1.19	1.00
	Found it very difficult	1.08	1.00
	Lived comfortably	1.32	1.13
Physically or sexually abused when growing up	(Ref: No)		
		1.26	1.00
Felt like an outsider in your family when growing up	(Ref: No)		
	Yes	1.28	1.00
Your health when growing up	(Ref: Good)		
	Excellent	1.13	1.00
	Fair	1.30	1.00
	Poor	1.26	1.00
	Very good	1.16	1.00
Born in This country	(Ref: Born in this country)		
	Born in another country	1.33	1.06
How Often You Attended Religious Services or Worshiped When You Were Around 12 Years Old	(Ref: Never)		
	At least once a week	1.59	1.41
	Less than once a month	1.59	1.41
	One to three times a month	1.54	1.31
Year of birth (age group)	(Ref: 1998-2005; current age: 18-24)		
	1943 or earlier (current age: 80+ years)	1.34	1.00
	1943-1953 (current age: 70-79 years)	1.33	1.00

Variable	Category	E-value for Estimate	E-value for 95% CI
	1953-1963 (current age: 60-69 years)	1.10	1.00
	1963-1973 (current age: 50-59 years)	1.19	1.00
	1973-1983 (current age: 40-49 years)	1.15	1.00
	1983-1993 (current age: 30-39 years)	1.05	1.00
	1993-1998 (current age: 25-29 years)	1.32	1.00
Religion when twelve years old	(Ref: No religion/Atheist/Agnostic)		
	Christianity	1.33	1.10
	Collapsed affiliations with prevalence<3%	1.52	1.00
	Islam	1.63	1.09
Race plurality (prominent race/ethnic group [0] or not [1])	(Ref: Plurality group)		
	Non-plurality groups	1.16	1.00

Table S23a. United States: Demographic descriptive statistics

Characteristic	N = 32,192[1]
**Age group**	
1943 or earlier (current age: 80+ years)	1,249 (3.9%)
1943-1953 (current age: 70-79 years)	3,919 (12%)
1953-1963 (current age: 60-69 years)	6,715 (21%)
1963-1973 (current age: 50-59 years)	6,363 (20%)
1973-1983 (current age: 40-49 years)	4,966 (15%)
1983-1993 (current age: 30-39 years)	5,751 (18%)
1993-1998 (current age: 25-29 years)	2,111 (6.6%)
1998-2005 (current age: 18-24 years)	1,119 (3.5%)
(Missing)	0 (0%)
**Gender**	
1. Male	15,188 (47%)
2. Female	16,699 (52%)
3. Other	239 (0.7%)
(Missing)	66 (0.2%)
**Marital status**	
1. Single/Never been married	6,520 (20%)
2. Married	18,448 (57%)
3. Separated	494 (1.5%)
4. Divorced	3,346 (10%)
5. Widowed	1,734 (5.4%)
6. Domestic partner	1,498 (4.7%)
(Missing)	153 (0.5%)
**Employment**	
1. Employed for an employer	16,504 (51%)
2. Self-employed	2,754 (8.6%)
3. Retired	8,510 (26%)
4. Student	625 (1.9%)
5. Homemaker	1,768 (5.5%)
6. Unemployed and looking for a job	1,014 (3.1%)
7. None of these/Other	938 (2.9%)
(Missing)	80 (0.2%)
**Religious service attendance**	
1. More than once a week	2,319 (7.2%)
2. Once a week	5,349 (17%)
3. One to three times a month	2,368 (7.4%)
4. A few times a year	7,459 (23%)
5. Never	14,604 (45%)
(Missing)	93 (0.3%)
**Education**	
1. Up to 8	71 (0.2%)
2. 9-15	18,898 (59%)
3. 16+	13,148 (41%)
(Missing)	75 (0.2%)
**Immigration**	
1. Born in this country	29,508 (92%)
2. Born in another country	2,396 (7.4%)

Characteristic	N = 32,192[1]
(Missing)	288 (0.9%)
**Religious affiliation**	
1. Christianity	20,039 (62%)
10. Taoism	40 (0.1%)
11. Confucianism	9 (<0.1%)
12. Primal, Animist, or Folk religion	130 (0.4%)
13. Spiritism	0 (0%)
14. Umbanda, Candomblé, and other African-derived religions	0 (0%)
15. Chinese folk/traditional religion	0 (0%)
2. Islam	126 (0.4%)
3. Hinduism	110 (0.3%)
4. Buddhism	271 (0.8%)
5. Judaism	619 (1.9%)
6. Sikhism	13 (<0.1%)
7. Baha'i	15 (<0.1%)
8. Jainism	10 (<0.1%)
9. Shinto	11 (<0.1%)
96. Some other religion	965 (3.0%)
97. No religion/Atheist/Agnostic	9,545 (30%)
(Missing)	290 (0.9%)
**RACE ETHNICITY**	
(Missing)	19 (<0.1%)
2201. United States: White	20,356 (63%)
2202. United States: Other	884 (2.7%)
2203. United States: Black	3,801 (12%)
2204. United States: Asian	1,572 (4.9%)
2205. United States: Hispanic	5,560 (17%)

[1]n (%)

Table S23b. United States: Childhood descriptive statistics

**Characteristic**	**N = 32,192**[1]
**Age**	52 (16)
**Year of birth**	
1943 or earlier (current age: 80+ years)	1,249 (3.9%)
1943-1953 (current age: 70-79 years)	3,919 (12%)
1953-1963 (current age: 60-69 years)	6,715 (21%)
1963-1973 (current age: 50-59 years)	6,363 (20%)
1973-1983 (current age: 40-49 years)	4,966 (15%)
1983-1993 (current age: 30-39 years)	5,751 (18%)
1993-1998 (current age: 25-29 years)	2,111 (6.6%)
1998-2005 (current age: 18-24 years)	1,119 (3.5%)
(Missing)	0 (0%)
**Gender**	
1. Male	15,188 (47%)
2. Female	16,699 (52%)
3. Other	239 (0.7%)
(Missing)	66 (0.2%)
**Parent marital status**	
1. Parents were married	24,073 (75%)
2. Parents were divorced	5,074 (16%)
3. Parents were never married	1,863 (5.8%)
4. One or both of them had died	903 (2.8%)
5. Unsure	241 (0.7%)
(Missing)	38 (0.1%)
**Age 12 religious service attendance**	
1. At least once a week	16,357 (51%)
2. One to three times a month	5,660 (18%)
3. Less than once a month	4,751 (15%)
4. Never	5,313 (17%)
(Missing)	111 (0.3%)
**Relationship with mother**	
1. Very good	17,778 (55%)
2. Somewhat good	9,679 (30%)
3. Somewhat bad	2,917 (9.1%)
4. Very bad	1,230 (3.8%)
97. (Does not apply)	489 (1.5%)
(Missing)	99 (0.3%)
**Relationship with father**	
1. Very good	13,316 (41%)
2. Somewhat good	10,692 (33%)
3. Somewhat bad	3,990 (12%)
4. Very bad	1,967 (6.1%)
97. (Does not apply)	2,191 (6.8%)
(Missing)	37 (0.1%)
**Outsider growing up**	
1. Yes	7,690 (24%)
2. No	24,226 (75%)
(Missing)	276 (0.9%)

Characteristic	N = 32,192[1]
**Abuse**	
1. Yes	7,960 (25%)
2. No	24,024 (75%)
(Missing)	208 (0.6%)
**Self-rated health growing up**	
1. Excellent	15,174 (47%)
2. Very good	10,208 (32%)
3. Good	4,877 (15%)
4. Fair	1,507 (4.7%)
5. Poor	352 (1.1%)
(Missing)	74 (0.2%)
**Immigration status**	
1. Born in this country	29,508 (92%)
2. Born in another country	2,396 (7.4%)
(Missing)	288 (0.9%)
**Subjective financial status of family growing up**	
1. Lived comfortably	13,009 (40%)
2. Got by	13,243 (41%)
3. Found it difficult	4,114 (13%)
4. Found it very difficult	1,809 (5.6%)
(Missing)	18 (<0.1%)
**Religious affiliation**	
1. Christianity	26,359 (82%)
10. Taoism	14 (<0.1%)
11. Confucianism	8 (<0.1%)
12. Primal, Animist, or Folk religion	37 (0.1%)
13. Spiritism	0 (0%)
14. Umbanda, Candomblé, and other African-derived religions	0 (0%)
15. Chinese folk/traditional religion	0 (0%)
2. Islam	152 (0.5%)
3. Hinduism	126 (0.4%)
4. Buddhism	124 (0.4%)
5. Judaism	738 (2.3%)
6. Sikhism	19 (<0.1%)
7. Baha'i	5 (<0.1%)
8. Jainism	10 (<0.1%)
9. Shinto	5 (<0.1%)
96. Some other religion	259 (0.8%)
97. No religion/Atheist/Agnostic	4,258 (13%)
(Missing)	78 (0.2%)
**Race/Ethnicity**	
(Missing)	19 (<0.1%)
2201. United States: White	20,356 (63%)
2202. United States: Other	884 (2.7%)
2203. United States: Black	3,801 (12%)
2204. United States: Asian	1,572 (4.9%)
2205. United States: Hispanic	5,560 (17%)

Characteristic	N = 32,192[1]

[1]Mean (SD); n (%)

Table S23c. United States: Proportions by demographic category

Variable	Category	Proportion	95% CI	SE	p-value
Age group	1998-2005 (current age: 18-24 years)	0.795	(0.729, 0.861)	0.034	2.33e-05
	1943 or earlier (current age: 80+ years)	0.878	(0.855, 0.901)	0.012	
	1943-1953 (current age: 70-79 years)	0.882	(0.870, 0.894)	0.006	
	1953-1963 (current age: 60-69 years)	0.884	(0.874, 0.895)	0.005	
	1963-1973 (current age: 50-59 years)	0.863	(0.850, 0.877)	0.007	
	1973-1983 (current age: 40-49 years)	0.851	(0.833, 0.868)	0.009	
	1983-1993 (current age: 30-39 years)	0.852	(0.834, 0.870)	0.009	
	1993-1998 (current age: 25-29 years)	0.819	(0.781, 0.858)	0.020	
Gender	Male	0.840	(0.830, 0.850)	0.005	1.16e-11
	Female	0.879	(0.870, 0.888)	0.005	
	Other	0.951	(0.910, 0.992)	0.021	
Marital status	Single/Never been married	0.824	(0.804, 0.844)	0.010	1.35e-04
	Divorced	0.859	(0.839, 0.878)	0.010	
	Domestic partner	0.865	(0.831, 0.898)	0.017	
	Married	0.875	(0.868, 0.883)	0.004	
	Separated	0.880	(0.833, 0.926)	0.024	
	Widowed	0.853	(0.828, 0.878)	0.013	
Employment	Employed for an employer	0.847	(0.837, 0.857)	0.005	1.86e-11
	Homemaker	0.905	(0.878, 0.932)	0.014	
	None of these/Other	0.816	(0.768, 0.863)	0.024	
	Retired	0.881	(0.872, 0.890)	0.005	
	Self-employed	0.906	(0.887, 0.926)	0.010	
	Student	0.858	(0.785, 0.930)	0.037	
	Unemployed and looking for a job	0.776	(0.711, 0.841)	0.033	
Religious service attendance	Never	0.839	(0.828, 0.849)	0.005	1.34e-10
	A few times a year	0.866	(0.852, 0.880)	0.007	
	More than once a week	0.901	(0.879, 0.922)	0.011	
	Once a week	0.892	(0.879, 0.905)	0.007	
	One to three times a month	0.878	(0.856, 0.901)	0.012	
Education	9-15	0.840	(0.830, 0.850)	0.005	1.11e-15
	16+	0.891	(0.885, 0.898)	0.003	

Variable	Category	Proportion	95% CI	SE	p-value
	Up to 8	0.926	(0.781, 1.000)	0.072	
Immigration status	Born in this country	0.861	(0.855, 0.868)	0.004	7.85e-01
	Born in another country	0.859	(0.831, 0.887)	0.014	
Religious affiliation	No religion/Atheist/Agnostic	0.853	(0.840, 0.866)	0.006	3.32e-02
	Christianity	0.863	(0.855, 0.871)	0.004	
	Combined	0.894	(0.865, 0.922)	0.015	
	Spiritism	*	*	*	
	Umbanda, Candomblé, and other African-derived religions	*	*	*	
	Buddhism	*	*	*	
	Islam	*	*	*	
	Chinese folk/traditional religion	*	*	*	
	Hinduism	*	*	*	
	Judaism	0.843	*	*	
	Primal, Animist, or Folk religion	*	*	*	

Table S23d. United States: Childhood predictors regression analysis

Variable	Category	Risk-Ratio	logRR SE	RR 95% CI	Global p-value
Relationship with your mother growing up	(Ref: Very bad/somewhat bad)				0.611
	Very good/somewhat good	1.000	0.012	(0.98,1.02)	
Relationship with your father growing up	(Ref: Very bad/somewhat bad)				0.597
	Very good/somewhat good	1.001	0.010	(0.98,1.02)	
Parents married to each other when you were around 12 years old	(Ref: Parents married)				0.154
	One or both of them had died	0.996	0.027	(0.94,1.05)	
	Parents were divorced	1.023	0.011	(1.00,1.04)	
	Parents were never married	1.002	0.024	(0.96,1.05)	
	Unsure	1.025	0.056	(0.92,1.14)	
Feelings about familys household income when growing up	(Ref: Got by)				0.027
	Found it difficult	0.999	0.012	(0.98,1.02)	
	Found it very difficult	0.992	0.019	(0.96,1.03)	
	Lived comfortably	1.019	0.007	(1.00,1.03)	
Physically or sexually abused when growing up	(Ref: No)				0.189
		1.013	0.009	(1.00,1.03)	
Felt like an outsider in your family when growing up	(Ref: No)				0.045
	Yes	0.979	0.010	(0.96,1.00)	
Your health when growing up	(Ref: Good)				0.156
	Excellent	1.024	0.011	(1.00,1.05)	
	Fair	1.017	0.022	(0.97,1.06)	
	Poor	1.012	0.043	(0.93,1.10)	
	Very good	1.012	0.012	(0.99,1.04)	
Born in This	(Ref: Born in this				0.398

Variable	Category	Risk-Ratio	logRR SE	RR 95% CI	Global p-value
country	country)				
	Born in another country	1.014	0.015	(0.98,1.04)	
How Often You Attended Religious Services or Worshiped When You Were Around 12 Years Old	(Ref: Never)				0.003
	At least once a week	1.038	0.012	(1.01,1.06)	
	Less than once a month	1.022	0.014	(0.99,1.05)	
	One to three times a month	1.044	0.014	(1.02,1.07)	
Year of birth (age group)	(Ref: 1998-2005; current age: 18-24)				0.058
	1943 or earlier (current age: 80+ years)	1.075	0.035	(1.00,1.15)	
	1943-1953 (current age: 70-79 years)	1.078	0.034	(1.01,1.15)	
	1953-1963 (current age: 60-69 years)	1.082	0.034	(1.01,1.16)	
	1963-1973 (current age: 50-59 years)	1.063	0.034	(0.99,1.14)	
	1973-1983 (current age: 40-49 years)	1.056	0.034	(0.99,1.13)	
	1983-1993 (current age: 30-39 years)	1.058	0.034	(0.99,1.13)	
	1993-1998 (current age: 25-29 years)	1.025	0.038	(0.95,1.10)	
Religion when twelve years old	(Ref: No religion/Atheist/Agnostic)				0.141
	Christianity	0.997	0.013	(0.97,1.02)	
	Collapsed affiliations with prevalence<3%	0.995	0.028	(0.94,1.05)	
	Judaism	0.957	0.021	(0.92,1.00)	
Race plurality (prominent race/ethnic group [0] or not [1])	(Ref: Plurality group)				0.006
	Non-plurality groups	0.977	0.008	(0.96,0.99)	

Table S23e. United States: Sensitivity to unmeasured confounding of childhood predictors

Variable	Category	E-value for Estimate	E-value for 95% CI
Relationship with your mother growing up	(Ref: Very bad/somewhat bad)		
	Very good/somewhat good	1.02	1.00
Relationship with your father growing up	(Ref: Very bad/somewhat bad)		
	Very good/somewhat good	1.04	1.00
Parents married to each other when you were around 12 years old	(Ref: Parents married)		
	One or both of them had died	1.07	1.00
	Parents were divorced	1.18	1.05
	Parents were never married	1.05	1.00
	Unsure	1.18	1.00
Feelings about familys household income when growing up	(Ref: Got by)		
	Found it difficult	1.03	1.00
	Found it very difficult	1.10	1.00
	Lived comfortably	1.16	1.08
Physically or sexually abused when growing up	(Ref: No)		
		1.13	1.00
Felt like an outsider in your family when growing up	(Ref: No)		
	Yes	1.17	1.03
Your health when growing up	(Ref: Good)		
	Excellent	1.18	1.05
	Fair	1.15	1.00
	Poor	1.12	1.00
	Very good	1.12	1.00
Born in This country	(Ref: Born in this country)		
	Born in another country	1.13	1.00
How Often You Attended Religious Services or Worshiped When You Were Around 12 Years Old	(Ref: Never)		
	At least once a week	1.24	1.13
	Less than once a month	1.17	1.00
	One to three times a month	1.26	1.14
Year of birth (age group)	(Ref: 1998-2005; current age: 18-24)		
	1943 or earlier (current age: 80+ years)	1.36	1.06
	1943-1953 (current age: 70-79 years)	1.37	1.11

Variable	Category	E-value for Estimate	E-value for 95% CI
	1953-1963 (current age: 60-69 years)	1.38	1.13
	1963-1973 (current age: 50-59 years)	1.32	1.00
	1973-1983 (current age: 40-49 years)	1.30	1.00
	1983-1993 (current age: 30-39 years)	1.31	1.00
	1993-1998 (current age: 25-29 years)	1.18	1.00
Religion when twelve years old	(Ref: No religion/Atheist/Agnostic)		
	Christianity	1.06	1.00
	Collapsed affiliations with prevalence<3%	1.08	1.00
	Judaism	1.26	1.05
Race plurality (prominent race/ethnic group [0] or not [1])	(Ref: Plurality group)		
	Non-plurality groups	1.18	1.09

Table S24. Summary of proportion estimates by country

Variable	Category	Argentina	Australia	Brazil	China	Egypt	Germany	Hong Kong	India	Indonesia	Israel	Japan	Kenya	Mexico	Nigeria	Philippines	Poland	South Africa	Spain	Sweden	Tanzania	Turkey	United Kingdom	United States
Age group	1998-2005 (current age: 18-24 years)	0.840	0.933	0.777	0.870	0.811	0.883	0.634	0.681	0.895	0.899	0.440	0.813	0.862	0.904	0.903	0.737	0.904	0.840	0.841	0.644	0.749	0.718	0.795
	1943 or earlier (current age: 80+ years)	0.833	0.873	0.769	1.000	1.000	0.811	*	0.549	0.695	0.801	0.636	0.459	0.669	0.972	0.525	0.651	0.765	0.917	0.874	0.419	0.826	0.825	0.878
	1943-1953 (current age: 70-79 years)	0.759	0.865	0.757	0.817	0.726	0.841	0.561	0.501	0.540	0.775	0.586	0.686	0.794	0.871	0.807	0.675	0.929	0.775	0.840	0.401	0.529	0.658	0.882
	1953-1963 (current age: 60-69 years)	0.792	0.873	0.749	0.830	0.661	0.835	0.428	0.507	0.628	0.812	0.498	0.658	0.825	0.793	0.816	0.633	0.934	0.793	0.843	0.457	0.712	0.725	0.884
	1963-1973 (current age: 50-59 years)	0.817	0.856	0.744	0.832	0.727	0.803	0.475	0.586	0.687	0.859	0.405	0.668	0.814	0.908	0.770	0.685	0.900	0.787	0.845	0.488	0.688	0.743	0.863
	1973-1983 (current age: 40-49 years)	0.811	0.864	0.723	0.812	0.707	0.824	0.473	0.641	0.746	0.847	0.379	0.707	0.811	0.906	0.840	0.691	0.869	0.822	0.829	0.464	0.786	0.706	0.851
	1983-1993 (current age: 30-39 years)	0.877	0.863	0.704	0.807	0.718	0.830	0.425	0.627	0.819	0.837	0.334	0.753	0.795	0.899	0.859	0.712	0.909	0.793	0.830	0.514	0.701	0.738	0.852
	1993-1998 (current age: 25-29 years)	0.788	0.925	0.696	0.820	0.767	0.890	0.428	0.624	0.889	0.889	0.317	0.788	0.825	0.903	0.898	0.718	0.913	0.774	0.818	0.620	0.781	0.793	0.819
Gender	Male	0.821	0.859	0.734	0.795	0.759	0.822	0.492	0.668	0.800	0.827	0.378	0.785	0.835	0.903	0.842	0.675	0.905	0.795	0.803	0.597	0.706	0.713	0.840
	Female	0.825	0.889	0.733	0.857	0.724	0.850	0.450	0.573	0.775	0.868	0.505	0.723	0.803	0.893	0.861	0.699	0.899	0.808	0.871	0.505	0.742	0.738	0.879
	Other	1.000	1.000	0.672	*	*	0.701	*	*	1.000	*	0.365	1.000	1.000	1.000	0.836	1.000	*	0.037	0.915	*	*	0.565	0.951
Marital status	Single/Never been married	0.819	0.875	0.709	0.822	0.815	0.848	0.451	0.698	0.907	0.896	0.409	0.813	0.822	0.909	0.881	0.713	0.898	0.825	0.827	0.656	0.737	0.707	0.824
	Divorced	0.902	0.866	0.804	0.905	0.867	0.820	0.284	0.871	0.781	0.886	0.466	0.719	0.868	0.889	1.000	0.651	0.979	0.711	0.842	0.370	0.594	0.691	0.859
	Domestic partner	0.840	0.881	0.753	0.827	*	0.917	0.514	0.452	0.740	0.880	0.545	0.768	0.784	0.952	0.894	0.647	0.935	0.832	0.828	0.511	*	0.748	0.865
	Married	0.800	0.879	0.746	0.822	0.716	0.827	0.487	0.617	0.764	0.828	0.440	0.728	0.843	0.893	0.828	0.685	0.903	0.802	0.848	0.513	0.731	0.733	0.875
	Separated	0.863	0.841	0.695	0.876	0.800	0.820	1.000	0.461	0.733	0.828	0.464	0.722	0.785	0.943	0.771	0.666	0.829	0.730	0.839	0.527	0.567	0.834	0.880
	Widowed	0.783	0.873	0.706	0.836	0.698	0.804	0.364	0.436	0.644	0.763	0.593	0.646	0.778	0.844	0.817	0.697	0.887	0.781	0.829	0.480	0.710	0.733	0.853
Employment	Employed for an employer	0.823	0.866	0.758	0.813	0.743	0.834	0.472	0.630	0.788	0.867	0.388	0.802	0.835	0.943	0.849	0.709	0.893	0.815	0.827	0.697	0.717	0.735	0.847
	Homemaker	0.790	0.911	0.679	0.814	0.697	0.840	0.413	0.575	0.755	0.709	0.541	0.646	0.770	0.905	0.831	0.710	0.892	0.817	0.860	0.444	0.743	0.661	0.905
	None of these/Other	0.763	0.861	0.789	0.773	0.846	0.803	0.224	0.645	0.680	0.762	0.510	0.730	0.816	0.857	0.808	0.688	0.922	0.825	0.825	0.412	0.638	0.628	0.816

Table S24. Summary of proportion estimates by country

Variable	Category	Argentina	Australia	Brazil	China	Egypt	Germany	Hong Kong	India	Indonesia	Israel	Japan	Kenya	Mexico	Nigeria	Philippines	Poland	South Africa	Spain	Sweden	Tanzania	Turkey	United Kingdom	United States
	Retired	0.735	0.865	0.783	0.843	0.678	0.821	0.484	0.510	0.737	0.823	0.531	0.657	0.790	0.875	0.832	0.641	0.908	0.768	0.846	0.404	0.753	0.707	0.881
	Self-employed	0.881	0.889	0.731	0.852	0.797	0.886	0.409	0.643	0.795	0.841	0.531	0.740	0.841	0.893	0.857	0.686	0.914	0.838	0.879	0.550	0.706	0.838	0.906
	Student	0.855	0.930	0.821	0.854	0.847	0.902	0.861	0.728	0.959	0.948	0.499	0.858	0.881	0.894	0.920	0.726	0.851	0.842	0.862	0.757	0.692	0.698	0.858
	Unemployed and looking for a job	0.787	0.958	0.662	0.721	0.741	0.750	0.444	0.675	0.857	0.760	0.325	0.763	0.827	0.901	0.830	0.605	0.911	0.724	0.755	0.598	0.780	0.624	0.776
Religious service attendance	Never	0.817	0.868	0.681	0.808	0.721	0.812	0.426	0.551	0.795	0.818	0.416	0.728	0.778	0.899	0.850	0.675	0.852	0.792	0.814	0.434	0.651	0.674	0.839
	A few times a year	0.833	0.891	0.744	0.860	0.777	0.883	0.459	0.654	0.820	0.871	0.540	0.777	0.828	0.885	0.838	0.667	0.893	0.797	0.885	0.574	0.700	0.799	0.866
	More than once a week	0.836	0.914	0.781	0.922	0.739	0.959	0.931	0.654	0.794	0.889	0.688	0.734	0.872	0.902	0.833	0.773	0.900	0.854	0.909	0.562	0.721	0.910	0.901
	Once a week	0.798	0.876	0.720	0.883	0.761	0.850	0.586	0.613	0.765	0.818	0.699	0.759	0.805	0.891	0.862	0.709	0.903	0.822	0.905	0.552	0.838	0.792	0.892
	One to three times a month	0.856	0.910	0.760	0.933	0.771	0.843	0.590	0.617	0.813	0.845	0.458	0.760	0.836	0.903	0.859	0.674	0.939	0.834	0.860	0.514	0.693	0.794	0.878
Education	9-15	0.838	0.862	0.747	0.835	0.792	0.814	0.464	0.704	0.882	0.822	0.416	0.802	0.822	0.910	0.869	0.666	0.910	0.785	0.820	0.709	0.730	0.713	0.840
	16+	0.892	0.905	0.780	0.865	0.855	0.891	0.502	0.729	0.925	0.896	0.544	0.879	0.879	0.949	0.895	0.737	0.959	0.889	0.893	0.779	0.756	0.821	0.891
	Up to 8	0.772	0.809	0.676	0.816	0.688	0.709	0.414	0.588	0.696	0.650	0.327	0.675	0.755	0.878	0.792	0.696	0.868	0.723	0.756	0.495	0.694	0.592	0.926
Immigration status	Born in this country	0.819	0.879	0.736	0.825	0.742	0.836	0.450	0.622	0.787	0.853	0.445	0.753	0.815	0.898	0.852	0.688	0.904	0.802	0.835	0.548	0.725	0.715	0.861
	Born in another country	0.925	0.862	0.456	0.880	0.829	0.844	0.649	0.577	0.877	0.830	0.421	0.742	0.969	0.941	0.726	0.705	0.861	0.795	0.866	0.443	0.706	0.803	0.859
Religious affiliation	No religion/Atheist/Agnostic	0.844	0.884	0.743	0.811	*	0.839	0.425	*	*	*	0.396	*	0.773	*	*	0.768	0.887	0.795	0.832	*	0.634	0.717	0.853
	Christianity	0.816	0.865	0.726	*	*	0.834	0.542	*	0.801	*	*	0.758	0.822	0.917	0.851	0.679	0.904	0.809	0.840	0.580	*	0.733	0.863
	Combined	0.869	0.867	0.744	0.875	0.686	0.838	0.585	0.571	0.806	0.799	0.640	0.755	0.988	0.947	0.952	0.768	0.943	0.722	0.857	0.447	0.985	0.728	0.894
	Spiritism	*	*	0.769	*	*	*	*	*	*	*	*	*	*	*	*	*	*	*	*	*	*	*	*
	Umbanda, Candomblé, and other African-derived religions	*	*	0.799	*	*	*	*	*	*	*	*	*	*	*	*	*	*	*	*	*	*	*	*
	Buddhism	*	*	*	0.863	*	*	0.440	*	*	*	0.509	*	*	*	*	*	*	*	*	*	*	*	*
	Islam	*	*	*	*	0.743	*	*	0.535	0.786	0.672	*	0.698	*	0.879	0.849	*	*	*	*	0.501	0.727	*	*
	Chinese folk/traditional	*	*	*	*	*	*	0.669	*	*	*	*	*	*	*	*	*	*	*	*	*	*	*	*

Table S24. Summary of proportion estimates by country

Variable	Category	Argentina	Australia	Brazil	China	Egypt	Germany	Hong Kong	India	Indonesia	Israel	Japan	Kenya	Mexico	Nigeria	Philippines	Poland	South Africa	Spain	Sweden	Tanzania	Turkey	United Kingdom	United States
	religion																							
	Hinduism	*	*	*	*	*	*	*	0.639	*	*	*	*	*	*	*	*	*	*	*	*	*	*	*
	Judaism	*	*	*	*	*	*	*	*	*	0.891	*	*	*	*	*	*	*	*	*	*	*	*	0.843
	Primal, Animist, or Folk religion	*	*	*	*	*	*	*	*	*	*	*	*	*	*	*	*	0.870	*	*	*	*	*	*

Forest Plots (Demographics)

Figure S1. Forest plot for `Age group`-`1998-2005 (current age: 18-24 years)`

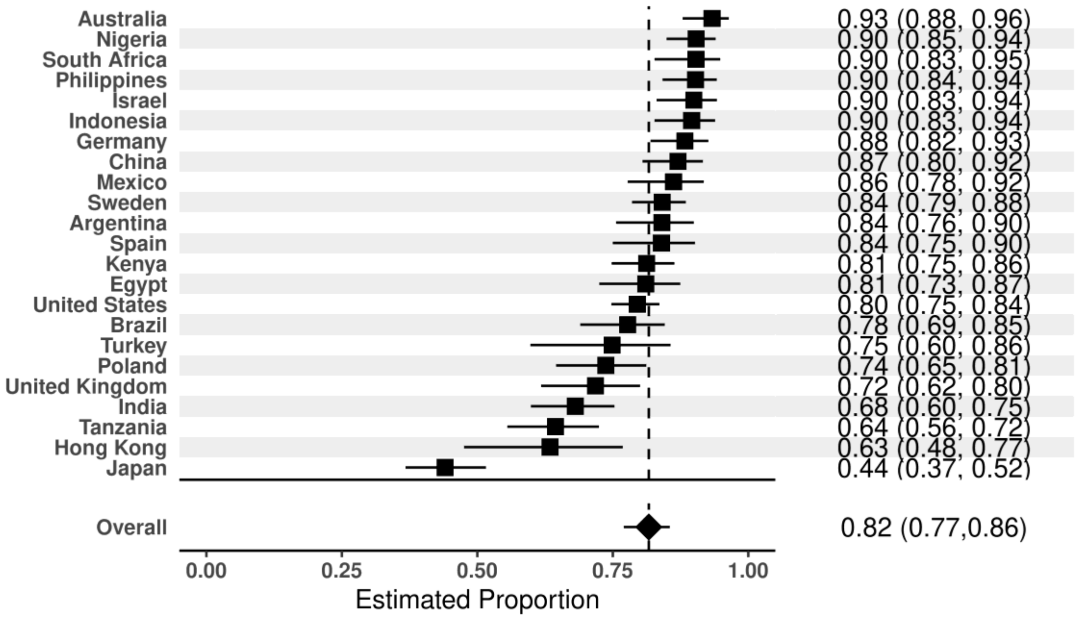

Probability-scale: τ=0.099;
Logit-scale: τ=0.658; Q(df=22)=308.61, p<.001;Q-profile 95% CI [0.478, 0.911]; I^2=91.83;
Plot is based on back transformed bounds after using approximate logit SE that aren't guarenteed to match the robust SE of a proportion.

Figure S2. Forest plot for `Age group`-`80 or older`

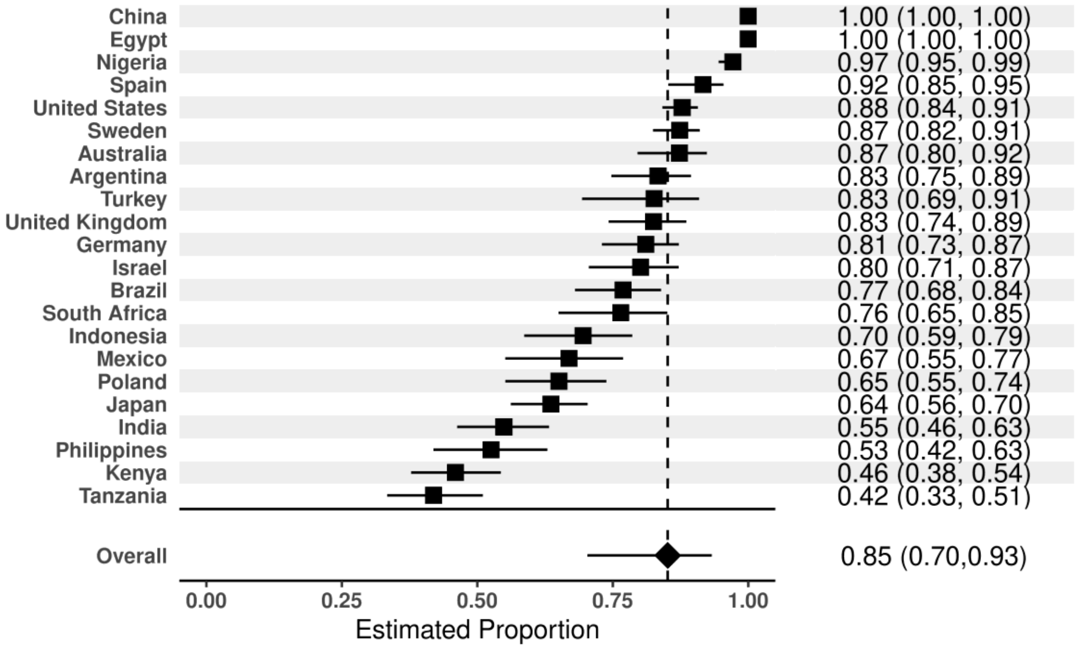

Probability-scale: τ=0.264;
Logit-scale: τ=2.087; Q(df=21)=539.84, p<.001;Q-profile 95% CI [1.278, 2.759]; I^2=99.11;
Plot is based on back transformed bounds after using approximate logit SE that aren't guarenteed to match the robust SE of a proportion.
Excluded countries: Hong Kong

Figure S3. Forest plot for `Age group`-`1943-1953 (current age: 70-79 years)`

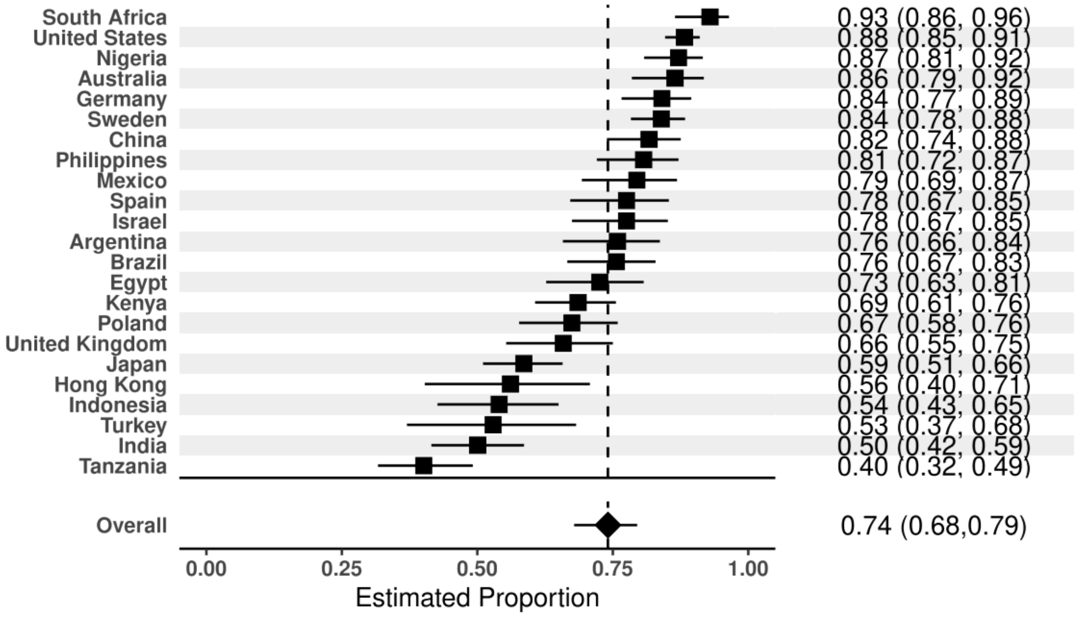

Probability-scale: τ=0.137;
Logit-scale: τ=0.714; Q(df=22)=351.94, p<.001;Q-profile 95% CI [0.515, 0.978]; I^2=93.48;
Plot is based on back transformed bounds after using approximate logit SE that aren't guarenteed to match the robust SE of a proportion.

Figure S4. Forest plot for `Age group`-`1953-1963 (current age: 60-69 years)`

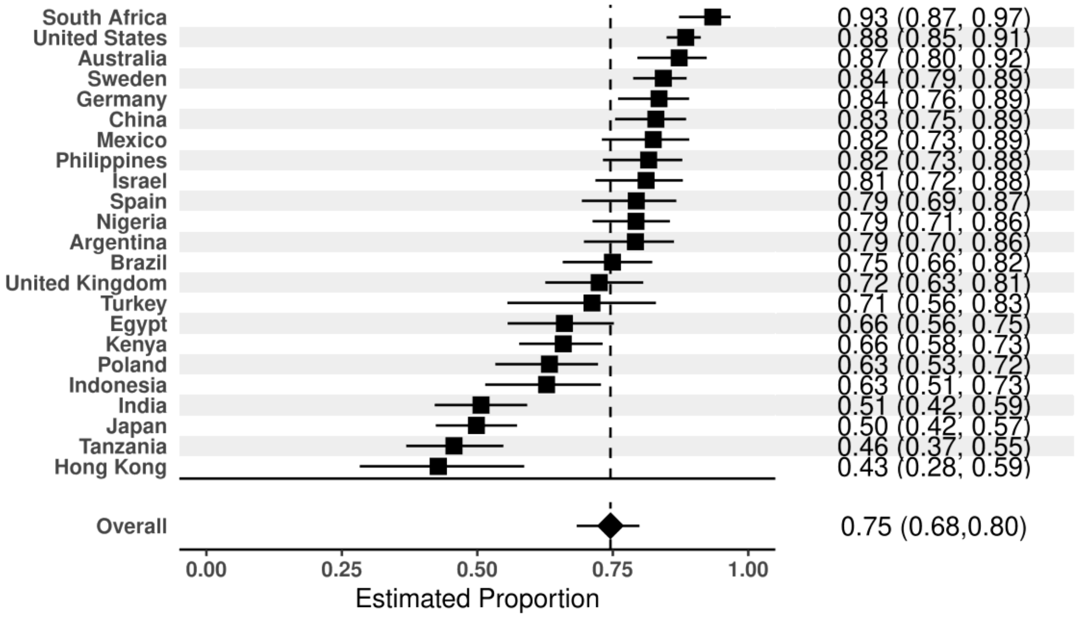

Probability-scale: τ=0.137;
Logit-scale: τ=0.720; Q(df=22)=362.70, p<.001;Q-profile 95% CI [0.519, 0.986]; I^2=93.58;
Plot is based on back transformed bounds after using approximate logit SE that aren't guarenteed to match the robust SE of a proportion.

Figure S5. Forest plot for `Age group`-`1963-1973 (current age: 50-59 years)`

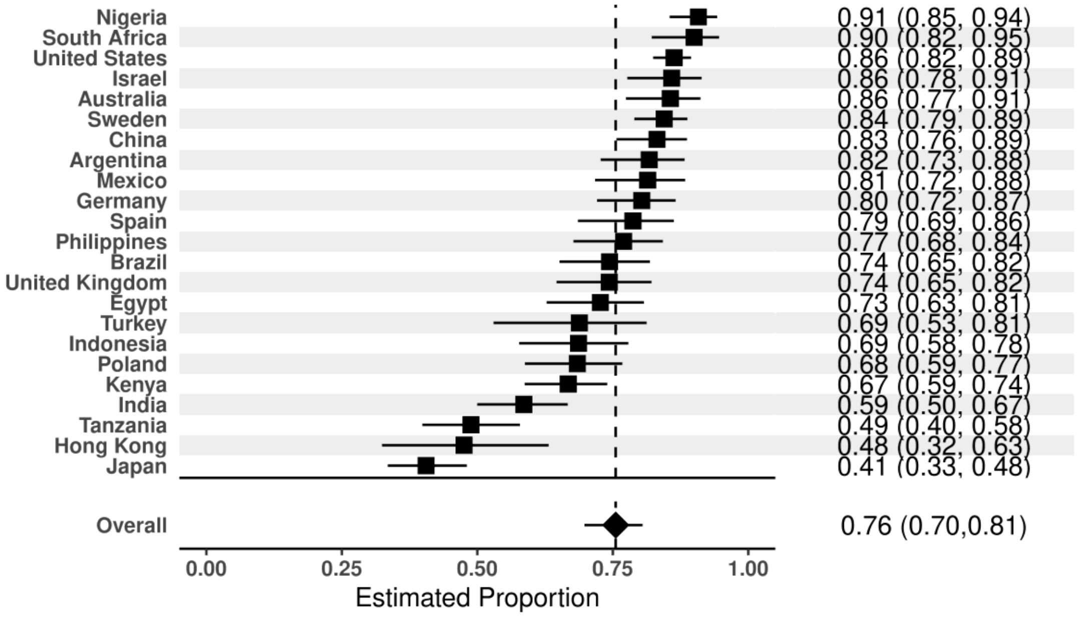

Probability-scale: τ=0.126;
Logit-scale: τ=0.682; Q(df=22)=358.40, p<.001;Q-profile 95% CI [0.496, 0.939]; I^2=92.84;
Plot is based on back transformed bounds after using approximate logit SE that aren't guarenteed to match the robust SE of a proportion.

Figure S6. Forest plot for `Age group`-`1973-1983 (current age: 40-49 years)`

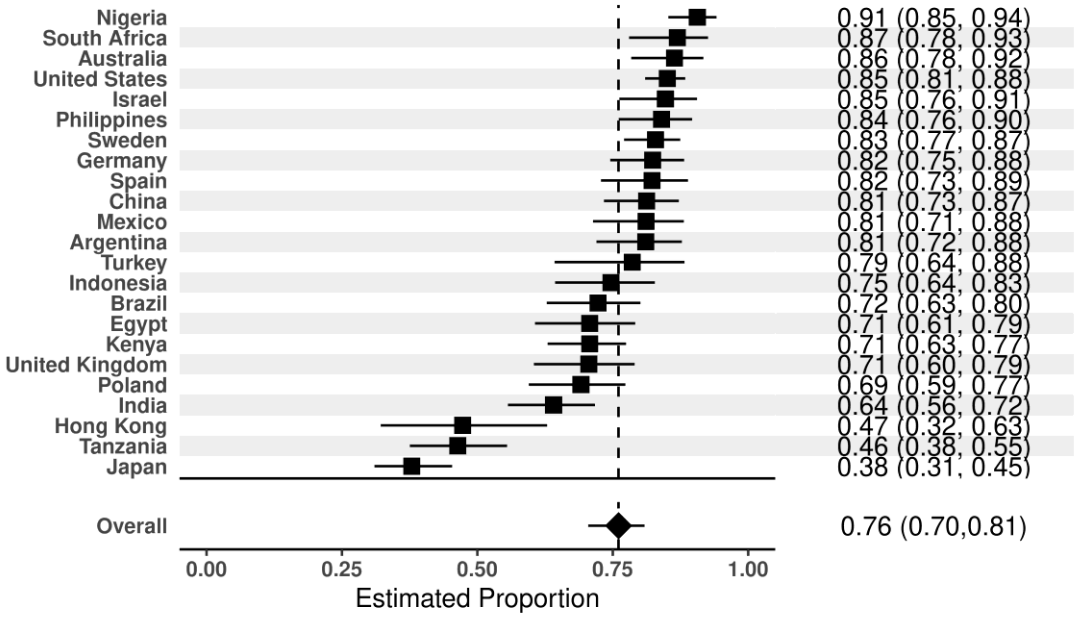

Probability-scale: τ=0.122;
Logit-scale: τ=0.668; Q(df=22)=356.47, p<.001;Q-profile 95% CI [0.488, 0.922]; I^2=92.53;
Plot is based on back transformed bounds after using approximate logit SE that aren't guarenteed to match the robust SE of a proportion.

Figure S7. Forest plot for `Age group`-`1983-1993 (current age: 30-39 years)`

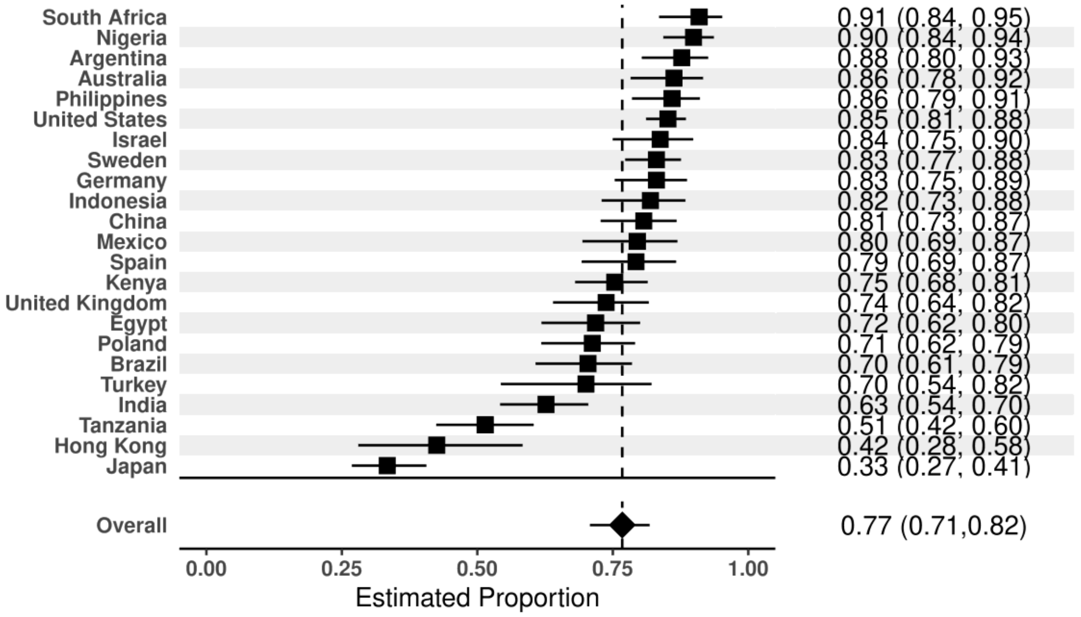

Probability-scale: τ=0.130;
Logit-scale: τ=0.727; Q(df=22)=397.63, p<.001;Q-profile 95% CI [0.530, 0.999]; I^2=93.52;
Plot is based on back transformed bounds after using approximate logit SE that aren't guarenteed to match the robust SE of a proportion.

Figure S8. Forest plot for `Age group`-`1993-1998 (current age: 25-29 years)`

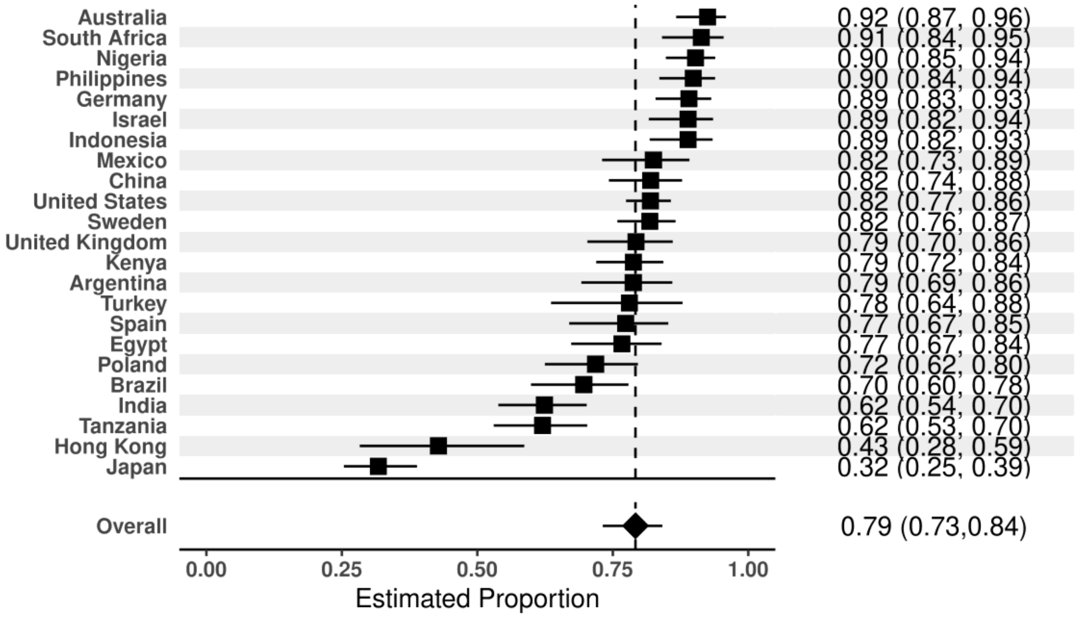

Probability-scale: τ=0.130;
Logit-scale: τ=0.787; Q(df=22)=421.05, p<.001;Q-profile 95% CI [0.574, 1.078]; I^2=94.26;
Plot is based on back transformed bounds after using approximate logit SE that aren't guarenteed to match the robust SE of a proportion.

Figure S9. Forest plot for `Gender`-`Male`

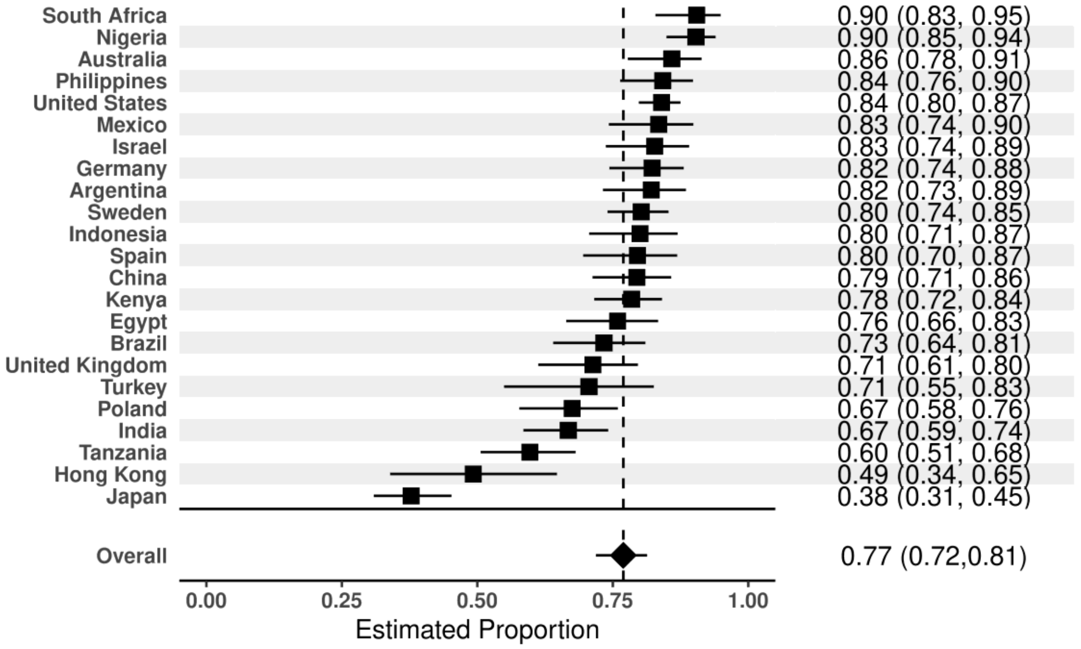

Probability-scale: τ=0.110;
Logit-scale: τ=0.619; Q(df=22)=301.66, p<.001;Q-profile 95% CI [0.447, 0.857]; I^2=91.37;
Plot is based on back transformed bounds after using approximate logit SE that aren't guarenteed to match the robust SE of a proportion.

Figure S10. Forest plot for `Gender`-`Female`

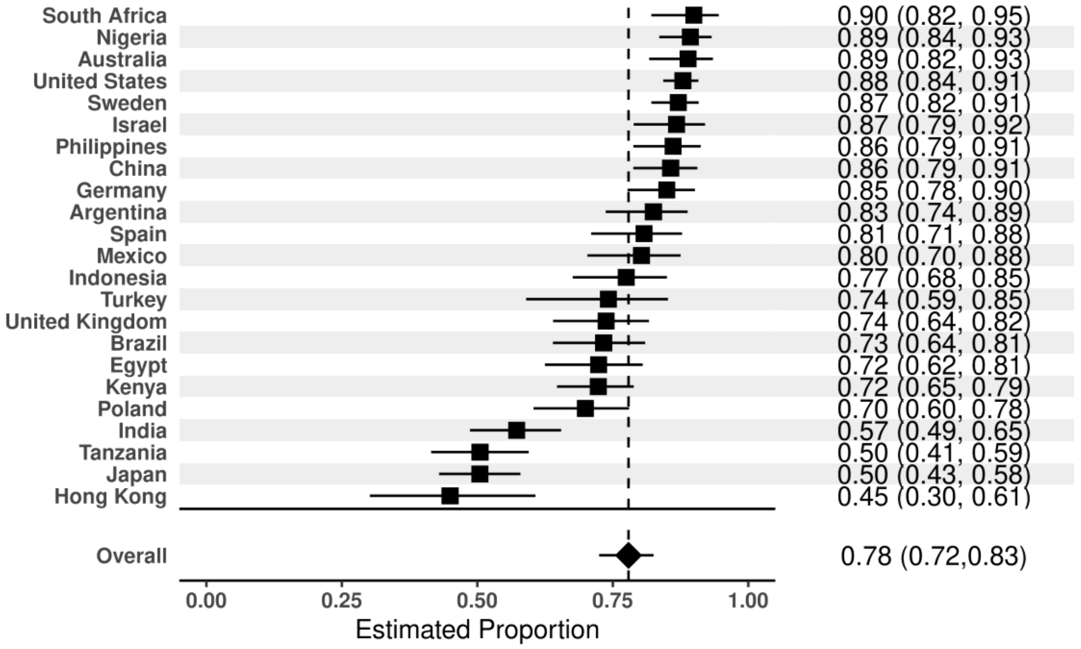

Probability-scale: τ=0.118;
Logit-scale: τ=0.684; Q(df=22)=341.41, p<.001;Q-profile 95% CI [0.497, 0.942]; I^2=92.65;
Plot is based on back transformed bounds after using approximate logit SE that aren't guarenteed to match the robust SE of a proportion.

Figure S11. Forest plot for `Gender`-`Other`

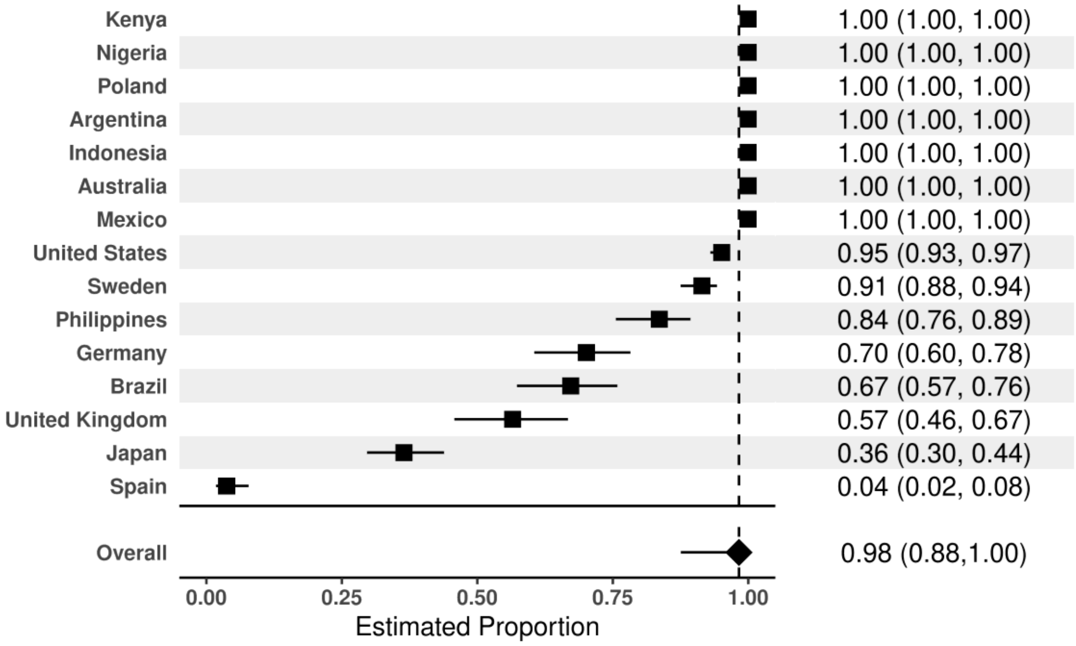

Probability-scale: τ=0.069;
Logit-scale: τ=4.088; Q(df=14)=892.34, p<.001;Q-profile 95% CI [2.815, 5.982]; I^2=99.61;
Plot is based on back transformed bounds after using approximate logit SE that aren't guarenteed to match the robust SE of a proportion.
Excluded countries: Hong Kong, India, Egypt, Israel, South Africa, Tanzania, Turkiye

Figure S12. Forest plot for `Marital status`-`Single, never married`

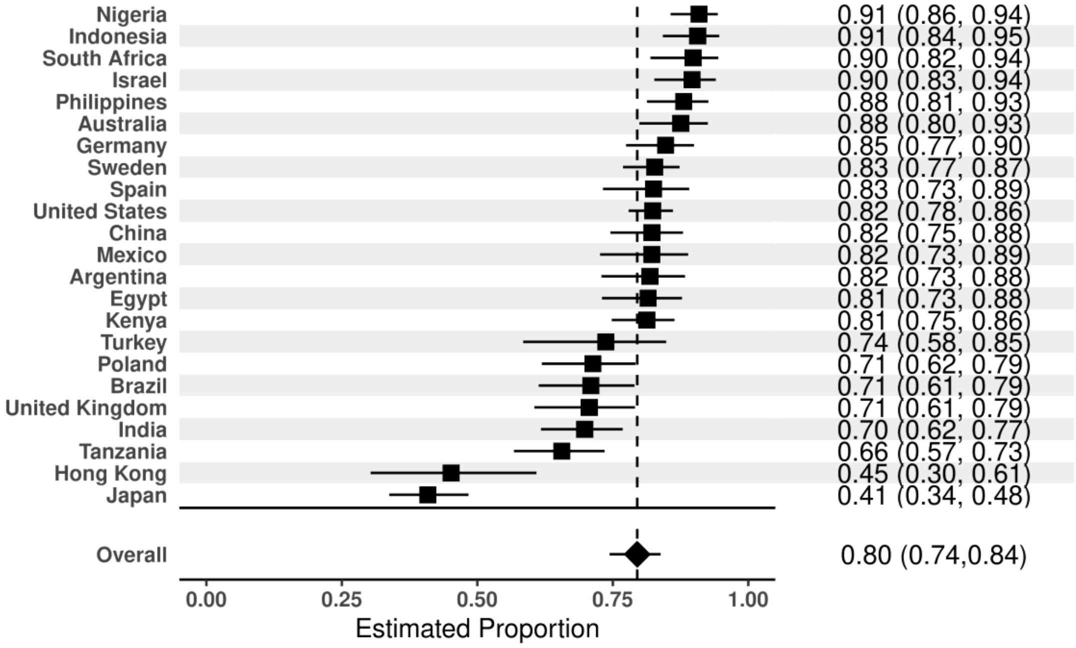

Probability-scale: τ=0.110;
Logit-scale: τ=0.676; Q(df=22)=315.78, p<.001;Q-profile 95% CI [0.487, 0.930]; I^2=92.38;
Plot is based on back transformed bounds after using approximate logit SE that aren't guarenteed to match the robust SE of a proportion.

Figure S13. Forest plot for `Marital status`-`Divorced`

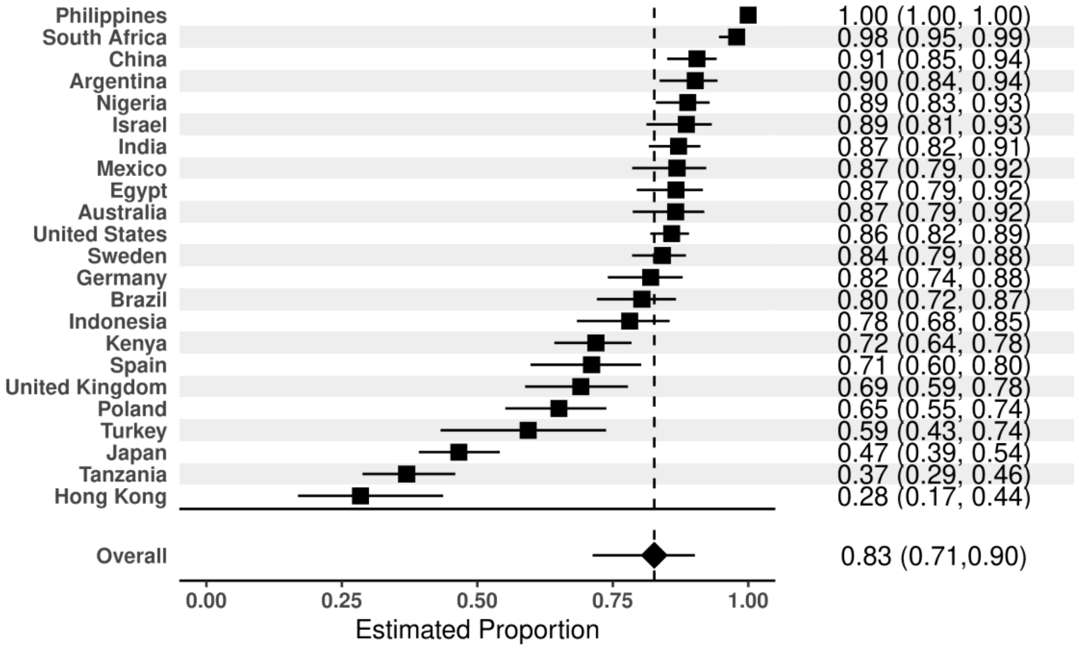

Probability-scale: τ=0.225;
Logit-scale: τ=1.573; Q(df=22)=534.06, p<.001;Q-profile 95% CI [0.936, 2.046]; I^2=98.40;
Plot is based on back transformed bounds after using approximate logit SE that aren't guarenteed to match the robust SE of a proportion.

Figure S14. Forest plot for `Marital status`-`Domestic partner`

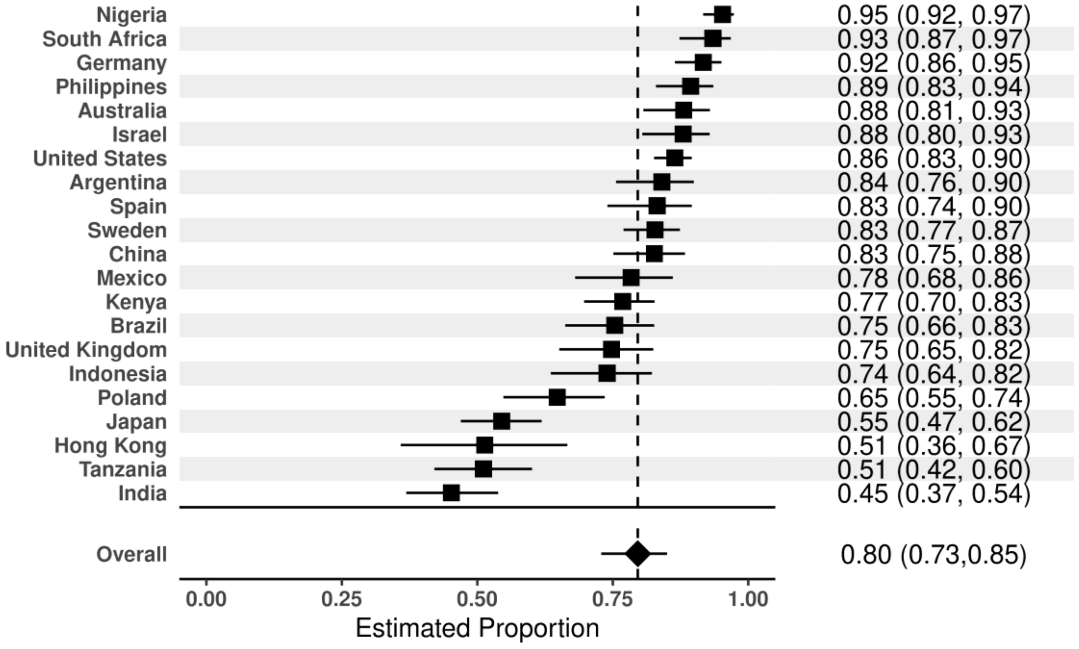

Probability-scale: τ=0.138;
Logit-scale: τ=0.849; Q(df=20)=404.83, p<.001;Q-profile 95% CI [0.610, 1.176]; I^2=95.16;
Plot is based on back transformed bounds after using approximate logit SE that aren't guarenteed to match the robust SE of a proportion.
Excluded countries: Egypt, Turkiye

Figure S15. Forest plot for `Marital status`-`Married`

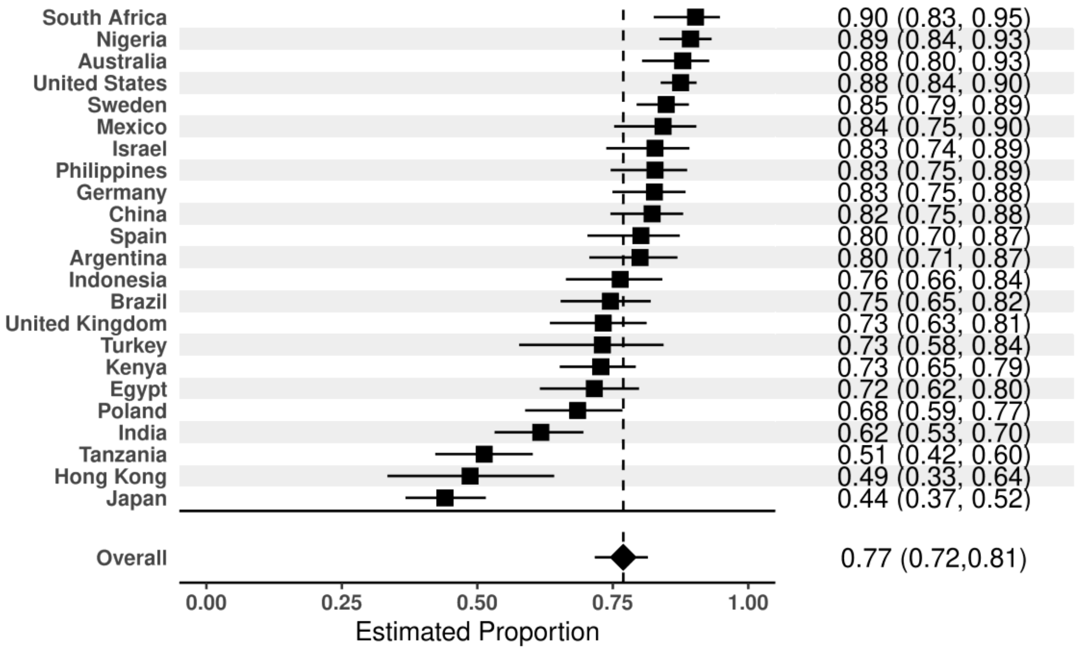

Probability-scale: τ=0.114;
Logit-scale: τ=0.644; Q(df=22)=325.96, p<.001;Q-profile 95% CI [0.468, 0.890]; I^2=91.93;
Plot is based on back transformed bounds after using approximate logit SE that aren't guarenteed to match the robust SE of a proportion.

Figure S16. Forest plot for `Marital status`-`Separated`

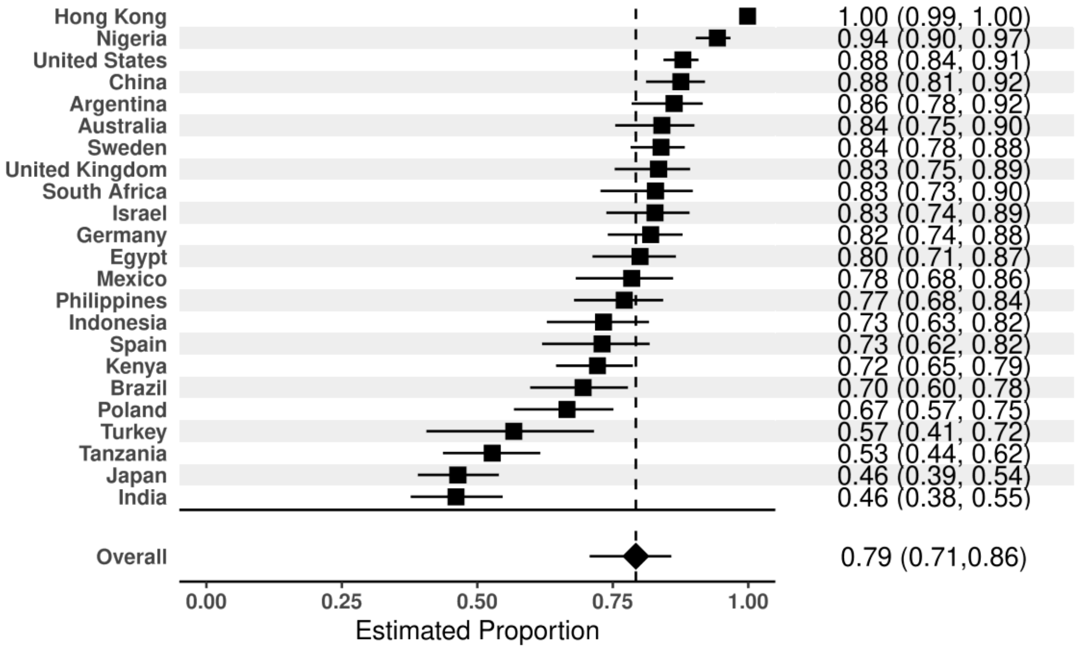

Probability-scale: τ=0.180;
Logit-scale: τ=1.093; Q(df=22)=407.26, p<.001;Q-profile 95% CI [0.566, 1.388]; I^2=96.97;
Plot is based on back transformed bounds after using approximate logit SE that aren't guarenteed to match the robust SE of a proportion.

Figure S17. Forest plot for `Marital status`-`Widowed`

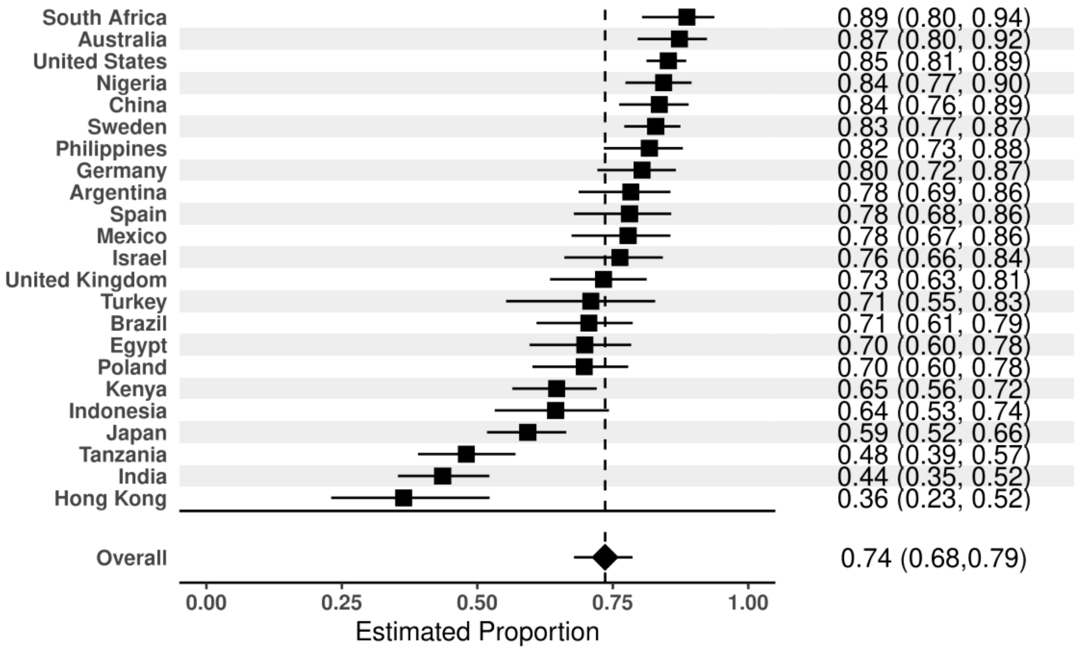

Probability-scale: τ=0.127;
Logit-scale: τ=0.652; Q(df=22)=299.94, p<.001;Q-profile 95% CI [0.468, 0.896]; I^2=92.39;
Plot is based on back transformed bounds after using approximate logit SE that
aren't guarenteed to match the robust SE of a proportion.

Figure S18. Forest plot for `Employment status`-`Employed for an employer`

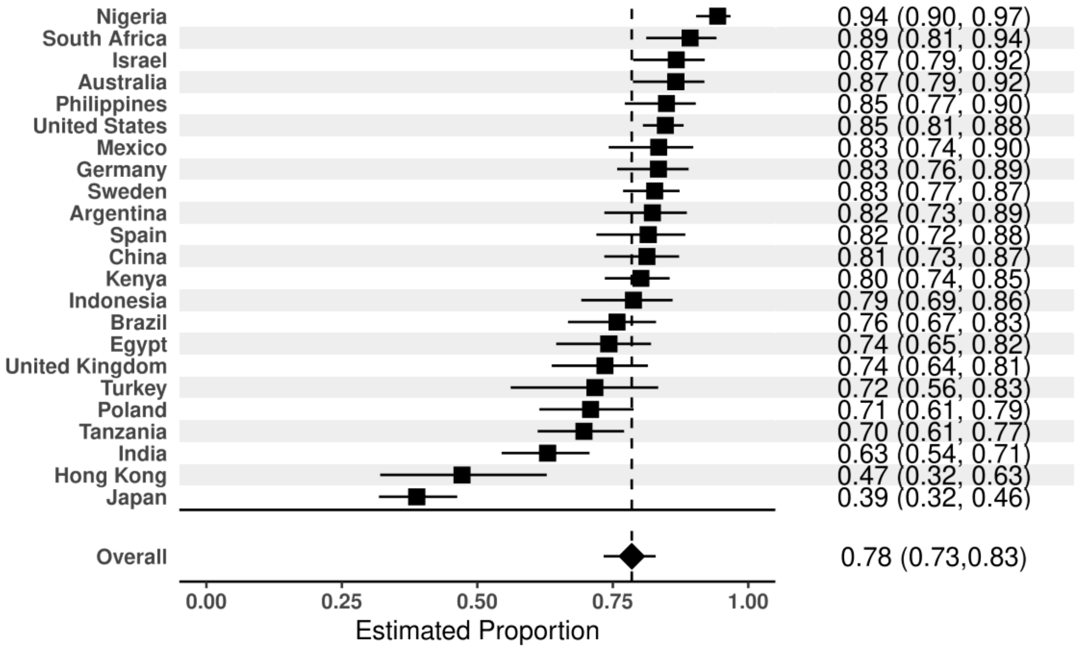

Probability-scale: τ=0.112;
Logit-scale: τ=0.664; Q(df=22)=320.58, p<.001;Q-profile 95% CI [0.479, 0.915]; I^2=92.22;
Plot is based on back transformed bounds after using approximate logit SE that
aren't guarenteed to match the robust SE of a proportion.

Figure S19. Forest plot for `Employment status`-`Homemaker`

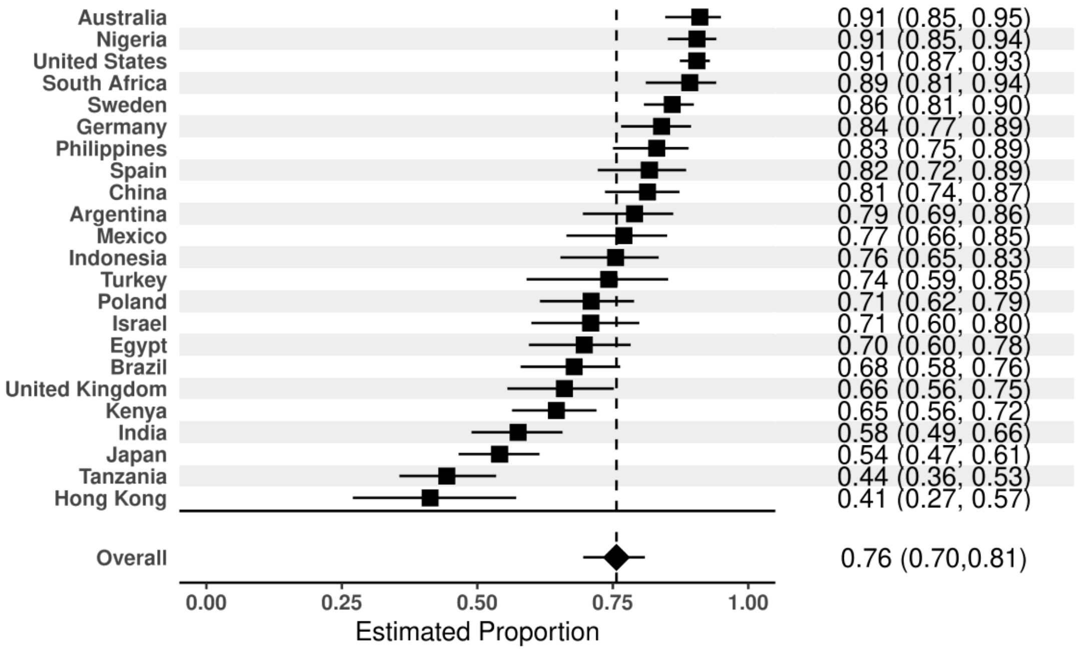

Probability-scale: τ=0.134;
Logit-scale: τ=0.729; Q(df=22)=372.13, p<.001;Q-profile 95% CI [0.530, 1.000]; I^2=93.61;
Plot is based on back transformed bounds after using approximate logit SE that
aren't guarenteed to match the robust SE of a proportion.

Figure S20. Forest plot for `Employment status`-`None of these/other`

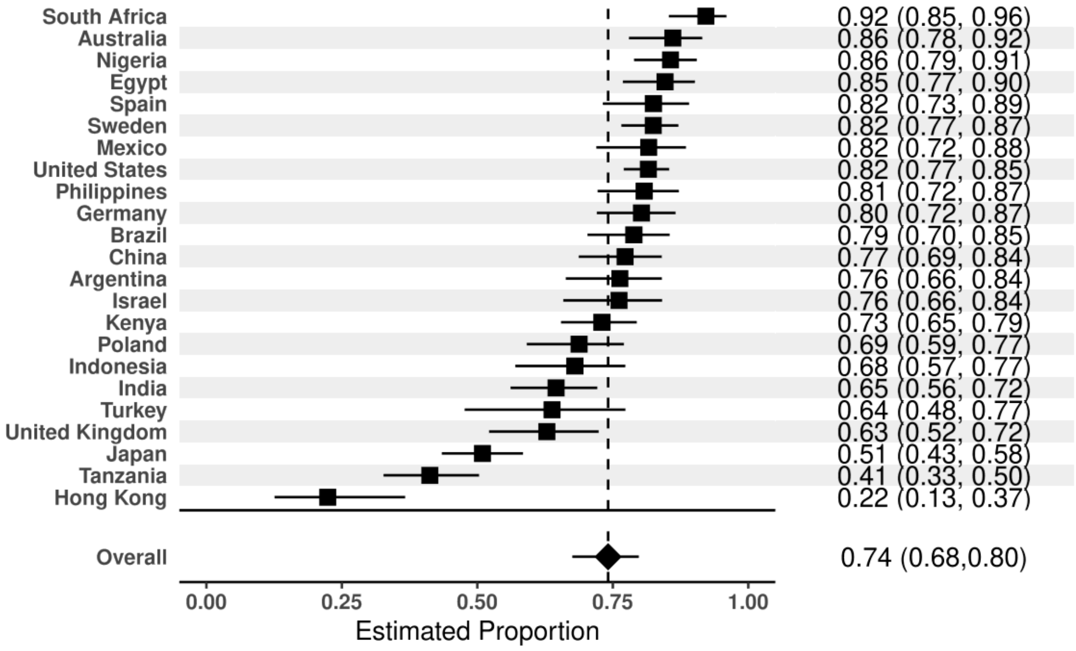

Probability-scale: τ=0.145;
Logit-scale: τ=0.757; Q(df=22)=315.06, p<.001;Q-profile 95% CI [0.535, 1.030]; I^2=94.18;
Plot is based on back transformed bounds after using approximate logit SE that
aren't guarenteed to match the robust SE of a proportion.

Figure S21. Forest plot for `Employment status`-`Retired`

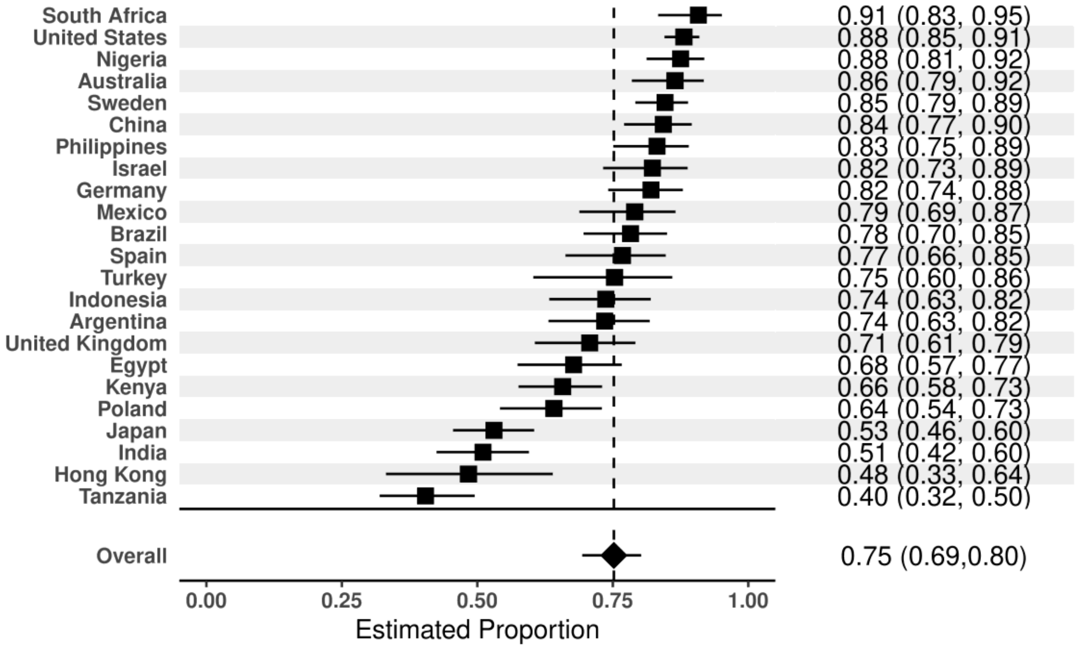

Probability-scale: τ=0.128;
Logit-scale: τ=0.685; Q(df=22)=358.77, p<.001;Q-profile 95% CI [0.500, 0.944]; I^2=92.91;
Plot is based on back transformed bounds after using approximate logit SE that
aren't guarenteed to match the robust SE of a proportion.

Figure S22. Forest plot for `Employment status`-`Self-employed`

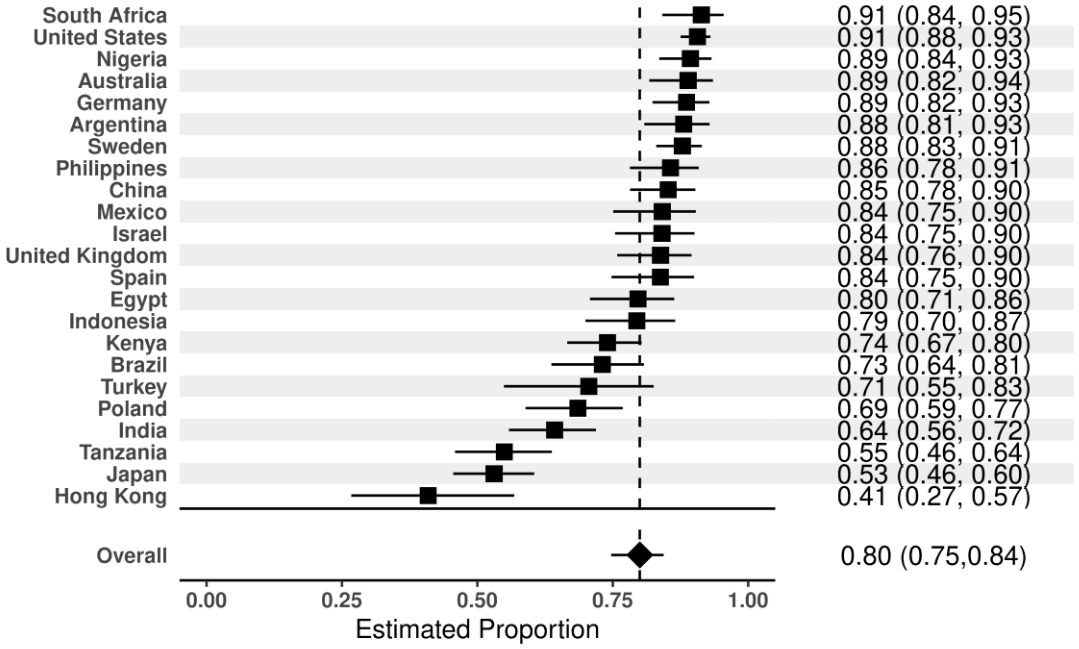

Probability-scale: τ=0.114;
Logit-scale: τ=0.710; Q(df=22)=345.60, p<.001;Q-profile 95% CI [0.514, 0.976]; I^2=92.90;
Plot is based on back transformed bounds after using approximate logit SE that
aren't guarenteed to match the robust SE of a proportion.

Figure S23. Forest plot for `Employment status`-`Student`

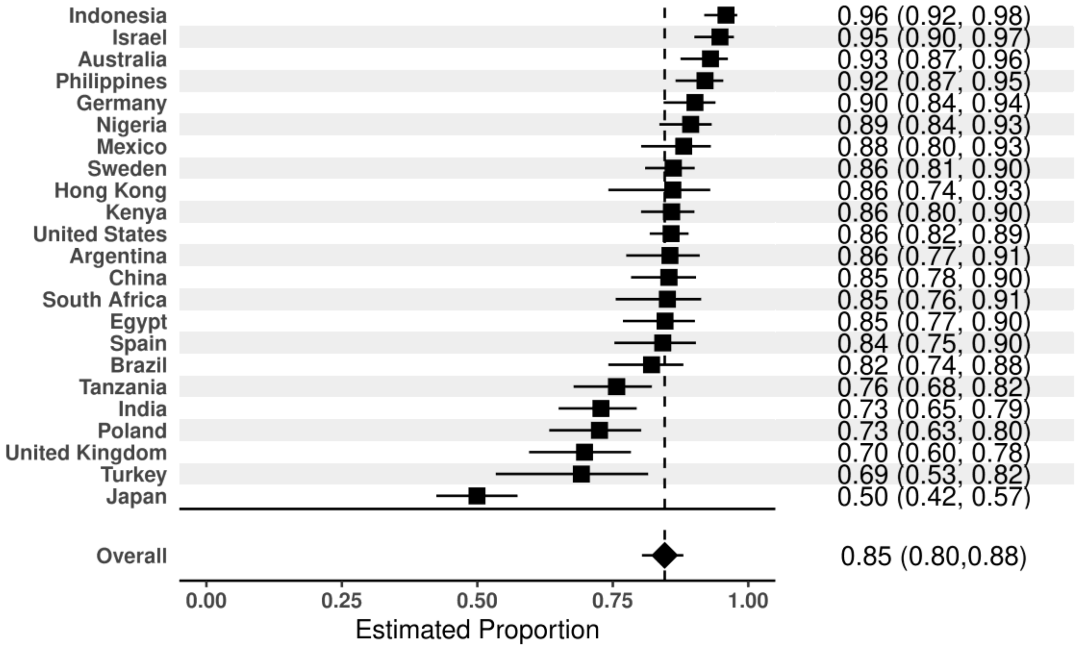

Probability-scale: τ=0.088;
Logit-scale: τ=0.678; Q(df=22)=294.12, p<.001;Q-profile 95% CI [0.486, 0.936]; I^2=91.81;
Plot is based on back transformed bounds after using approximate logit SE that
aren't guarenteed to match the robust SE of a proportion.

Figure S24. Forest plot for `Employment status`-`Unemployed and looking for a job`

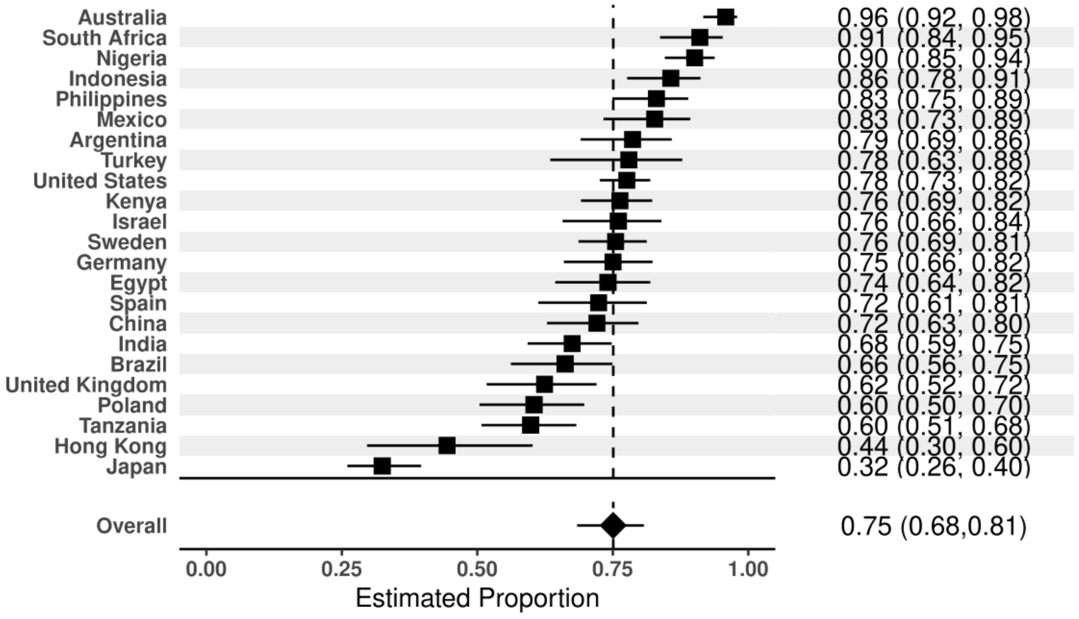

Probability-scale: τ=0.146;
Logit-scale: τ=0.779; Q(df=22)=351.74, p<.001;Q-profile 95% CI [0.556, 1.061]; I^2=94.51;
Plot is based on back transformed bounds after using approximate logit SE that
aren't guarenteed to match the robust SE of a proportion.

Figure S25. Forest plot for `Religious service attendance`-`Never`

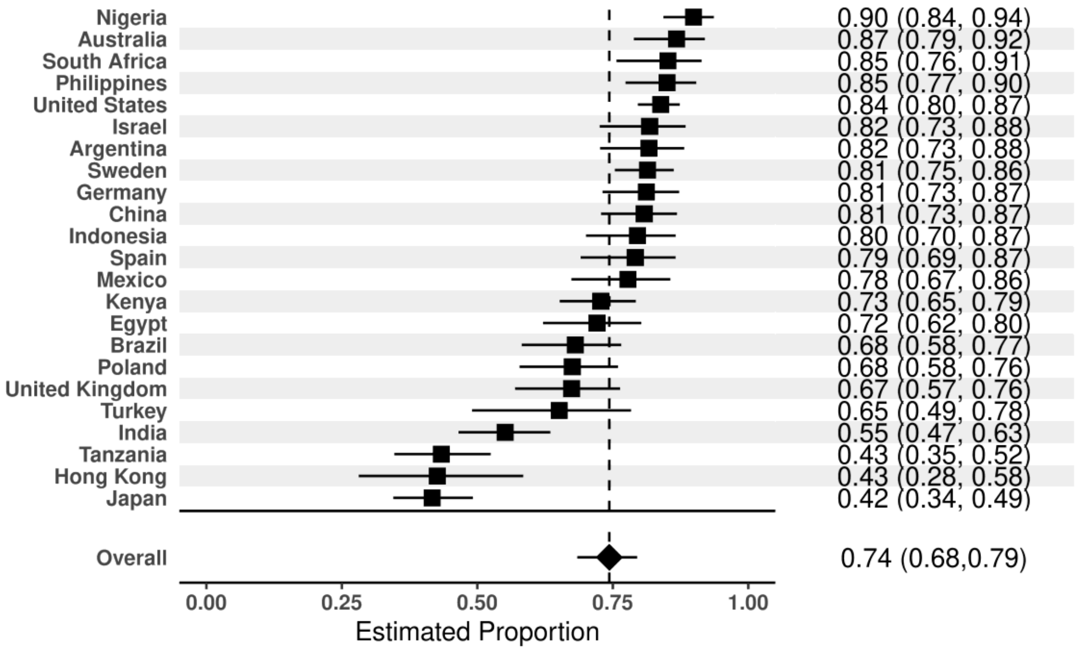

Probability-scale: τ=0.130;
Logit-scale: τ=0.680; Q(df=22)=352.00, p<.001;Q-profile 95% CI [0.495, 0.936]; I^2=92.89;
Plot is based on back transformed bounds after using approximate logit SE that aren't guarenteed to match the robust SE of a proportion.

Figure S26. Forest plot for `Religious service attendance`-`A few times a year`

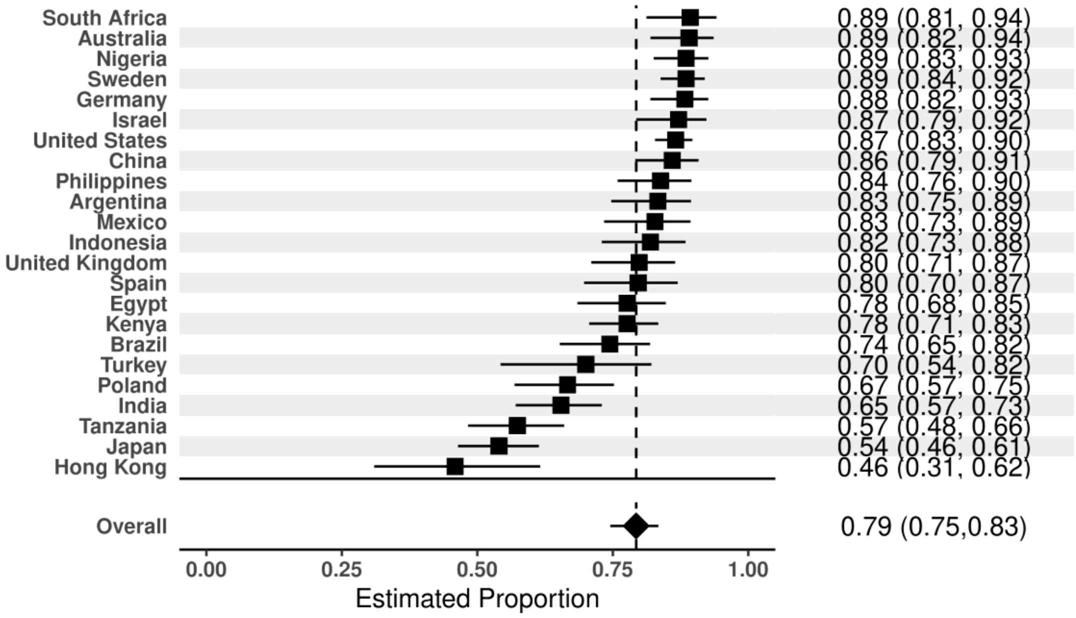

Probability-scale: τ=0.103;
Logit-scale: τ=0.628; Q(df=22)=277.57, p<.001;Q-profile 95% CI [0.451, 0.868]; I^2=91.28;
Plot is based on back transformed bounds after using approximate logit SE that aren't guarenteed to match the robust SE of a proportion.

Figure S27. Forest plot for `Religious service attendance`-`>1/week`

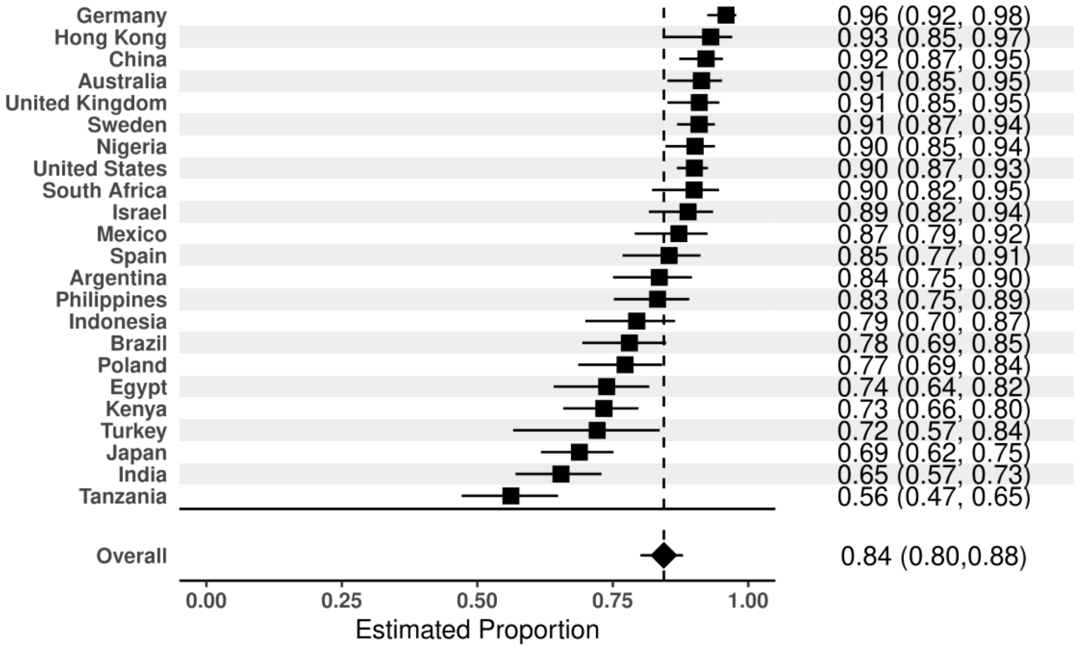

Probability-scale: τ=0.091;
Logit-scale: τ=0.694; Q(df=22)=299.52, p<.001;Q-profile 95% CI [0.502, 0.958]; I^2=92.13;
Plot is based on back transformed bounds after using approximate logit SE that
aren't guarenteed to match the robust SE of a proportion.

Figure S28. Forest plot for `Religious service attendance`-`1/week`

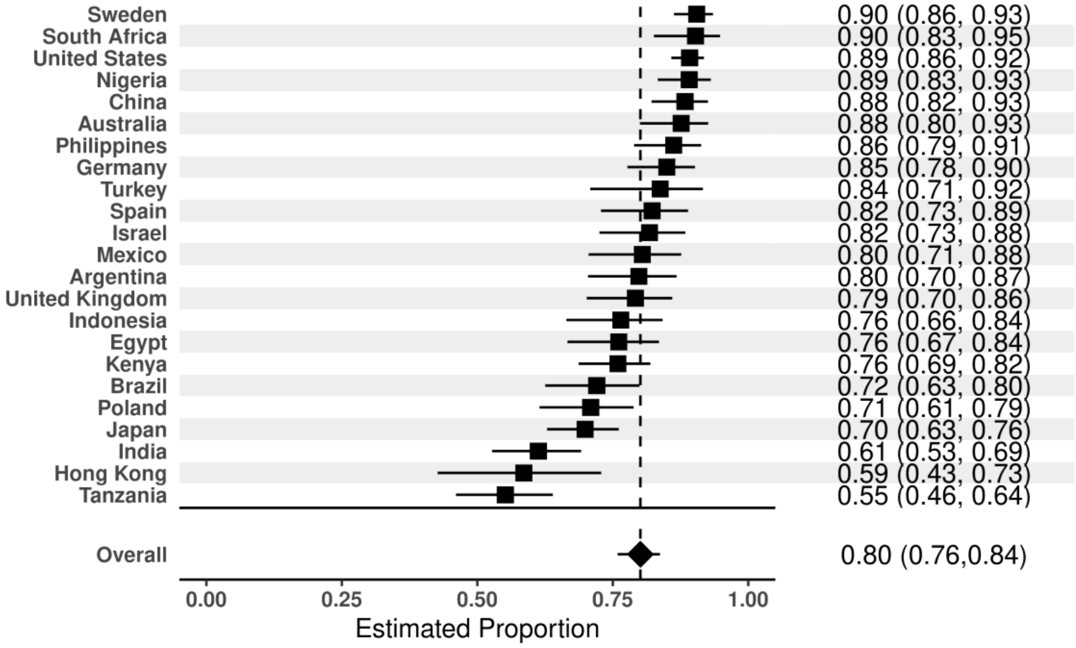

Probability-scale: τ=0.090;
Logit-scale: τ=0.562; Q(df=22)=233.97, p<.001;Q-profile 95% CI [0.405, 0.784]; I^2=89.23;
Plot is based on back transformed bounds after using approximate logit SE that
aren't guarenteed to match the robust SE of a proportion.

Figure S29. Forest plot for `Religious service attendance`-`1-3/month`

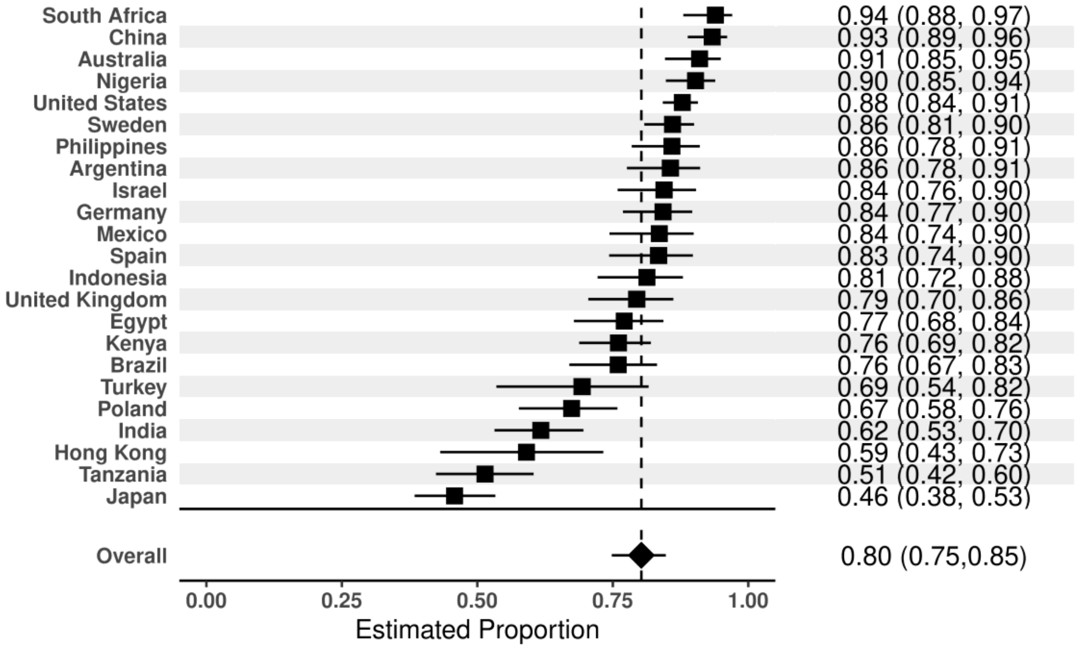

Probability-scale: τ=0.117;
Logit-scale: τ=0.740; Q(df=22)=382.25, p<.001;Q-profile 95% CI [0.538, 1.016]; I^2=93.45;
Plot is based on back transformed bounds after using approximate logit SE that aren't guarenteed to match the robust SE of a proportion.

Figure S30. Forest plot for `Education`-`9-15 years`

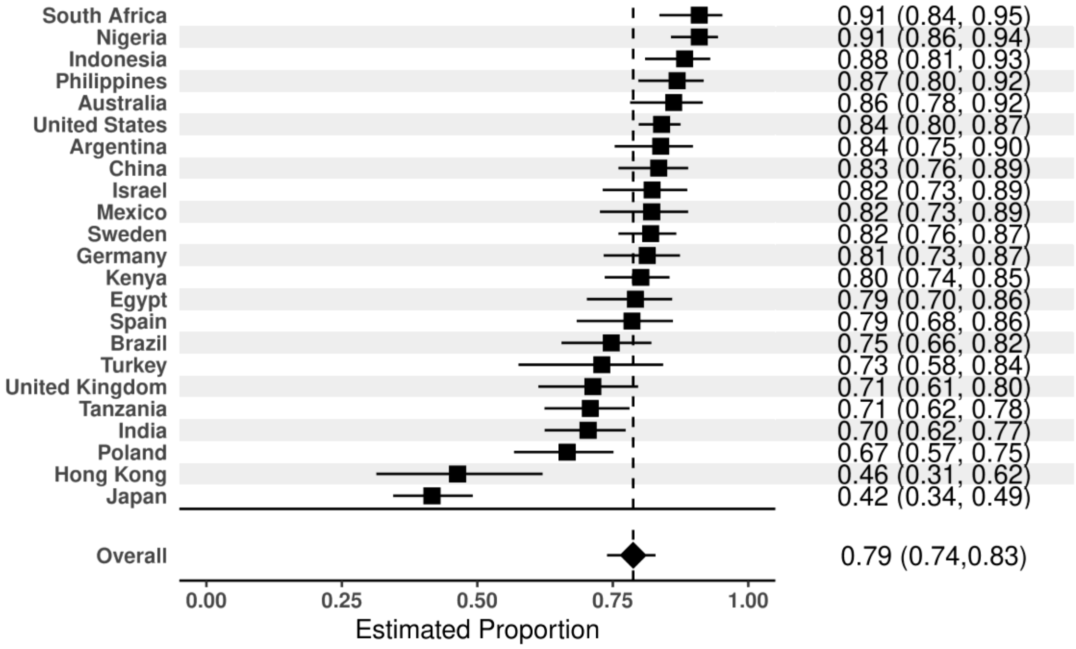

Probability-scale: τ=0.104;
Logit-scale: τ=0.621; Q(df=22)=281.33, p<.001;Q-profile 95% CI [0.445, 0.858]; I^2=91.20;
Plot is based on back transformed bounds after using approximate logit SE that aren't guarenteed to match the robust SE of a proportion.

Figure S31. Forest plot for `Education`-`16+ years`

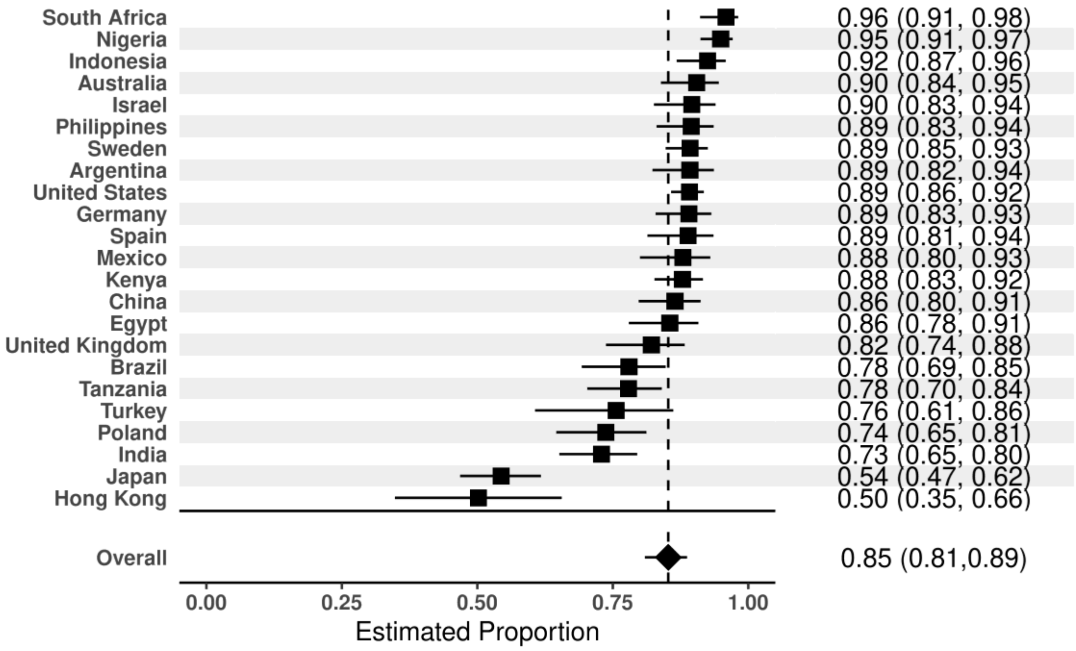

Probability-scale: τ=0.091;
Logit-scale: τ=0.720; Q(df=22)=304.87, p<.001;Q-profile 95% CI [0.513, 0.989]; I^2=92.43;
Plot is based on back transformed bounds after using approximate logit SE that
aren't guarenteed to match the robust SE of a proportion.

Figure S32. Forest plot for `Education`-`Up to 8 years`

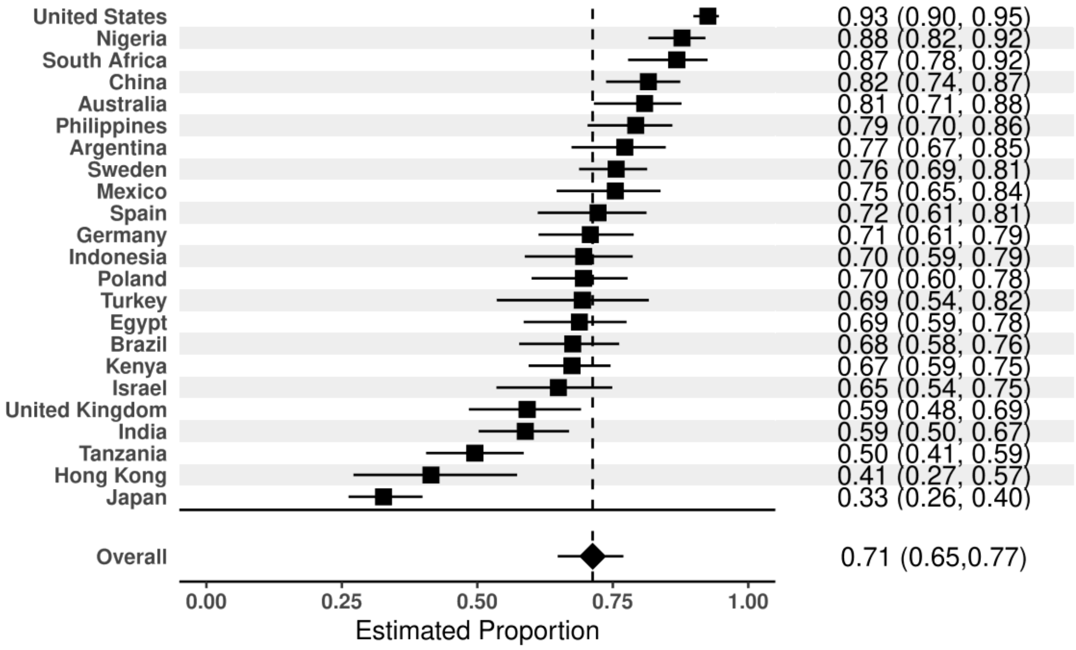

Probability-scale: τ=0.143;
Logit-scale: τ=0.699; Q(df=22)=412.37, p<.001;Q-profile 95% CI [0.513, 0.961]; I^2=93.34;
Plot is based on back transformed bounds after using approximate logit SE that
aren't guarenteed to match the robust SE of a proportion.

Figure S33. Forest plot for `Immigration status`-`Born in this country`

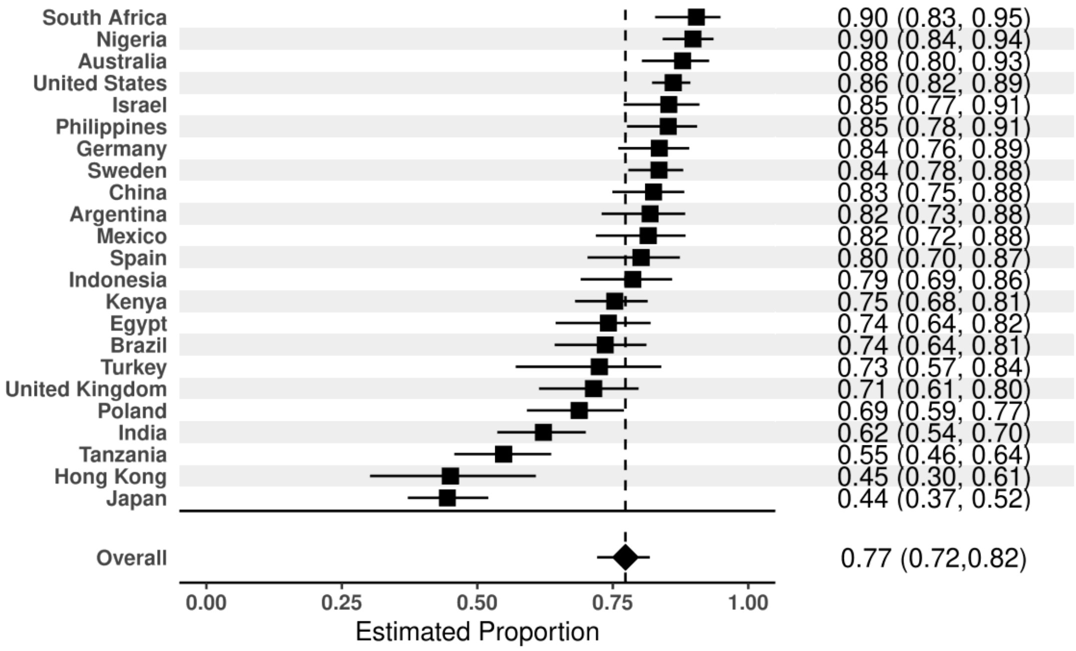

Probability-scale: τ=0.113;
Logit-scale: τ=0.647; Q(df=22)=311.25, p<.001;Q-profile 95% CI [0.467, 0.893]; I^2=91.97;
Plot is based on back transformed bounds after using approximate logit SE that
aren't guarenteed to match the robust SE of a proportion.

Figure S34. Forest plot for `Immigration status`-`Born in another country`

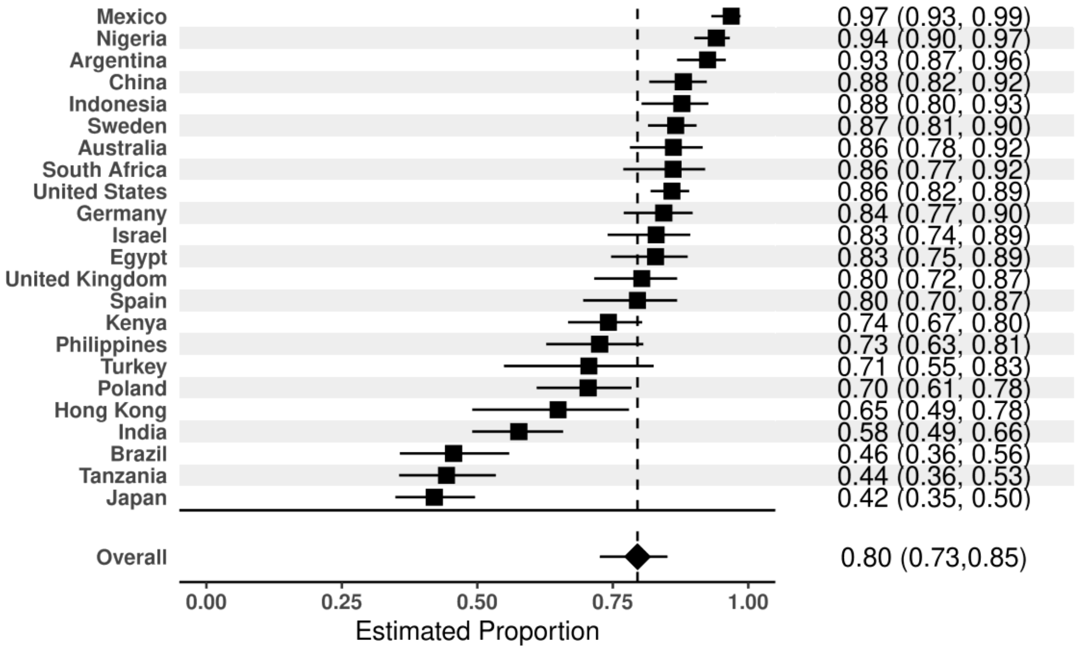

Probability-scale: τ=0.149;
Logit-scale: τ=0.913; Q(df=22)=511.80, p<.001;Q-profile 95% CI [0.666, 1.245]; I^2=95.63;
Plot is based on back transformed bounds after using approximate logit SE that
aren't guarenteed to match the robust SE of a proportion.

Pairwise differences forest plots

Figure S35. Forest plot for `Age group`-`(Ref: 1998-2005 (current age: 18-24 years)) 80 or older`

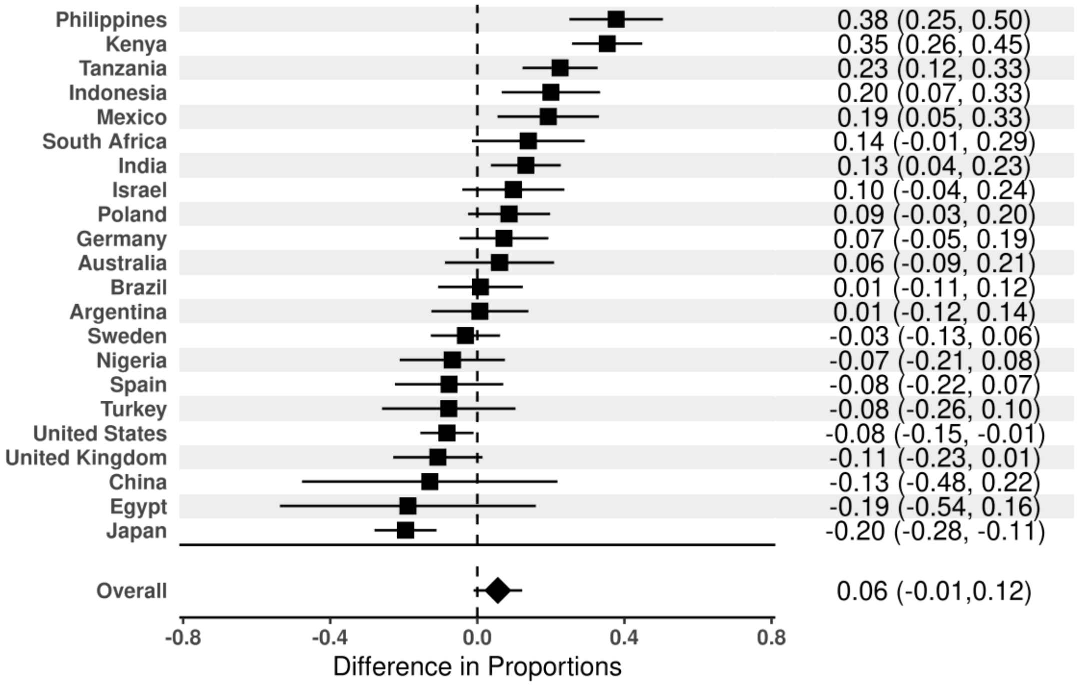

τ=0.141; Q(df=21)=155.40, p<.001;Q-profile 95% CI [0.099, 0.204]; I^2=84.21;
Excluded countries: Hong Kong

Figure S36. Forest plot for `Age group`-`(Ref: 1998-2005 (current age: 18-24 years)) 1943-1953 (current age: 70-79 years)`

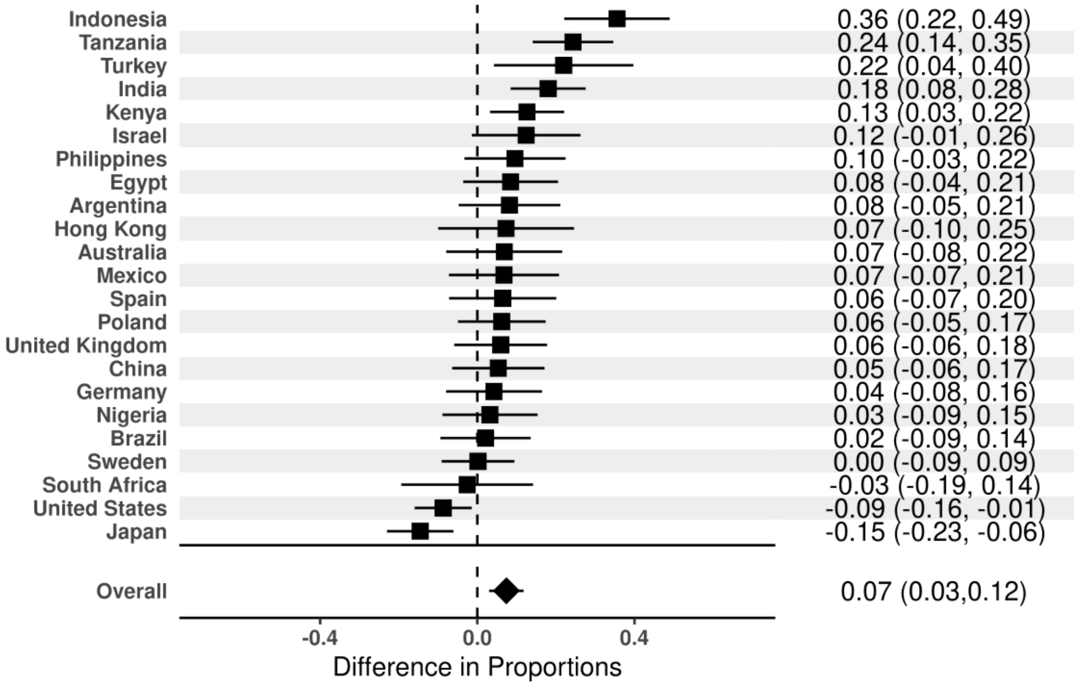

τ=0.087; Q(df=22)=85.19, p<.001;Q-profile 95% CI [0.058, 0.133]; I^2=68.72;

Figure S37. Forest plot for `Age group`-`(Ref: 1998-2005 (current age: 18-24 years)) 1953-1963 (current age: 60-69 years)`

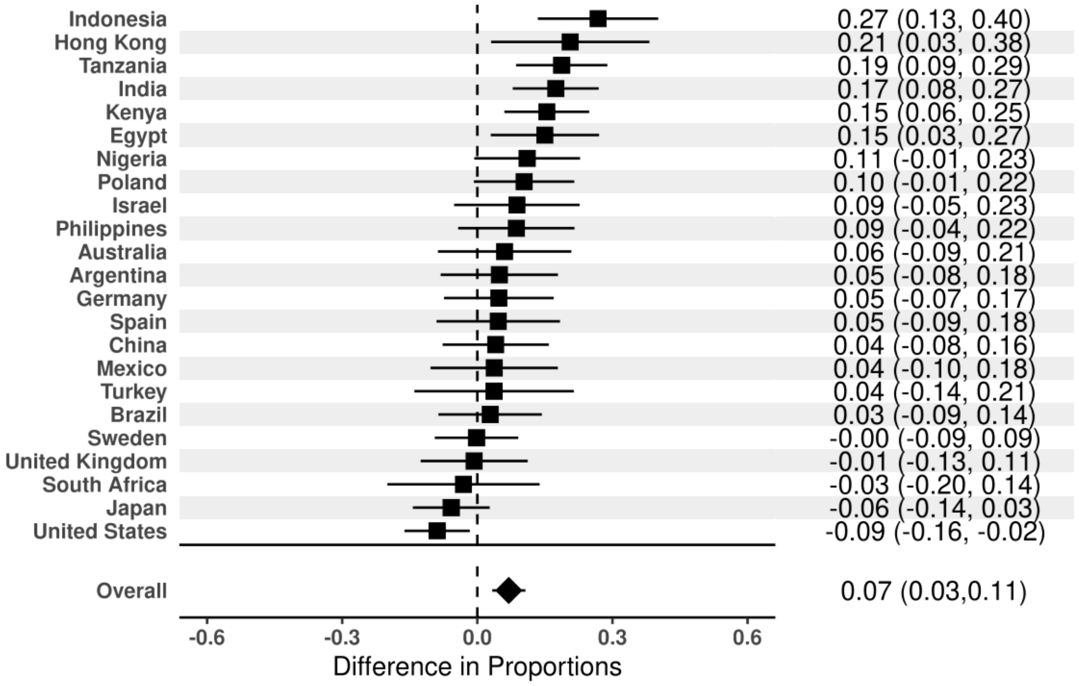

τ=0.066; Q(df=22)=59.39, p<.001;Q-profile 95% CI [0.041, 0.108]; I^2=55.44;

Figure S38. Forest plot for `Age group`-`(Ref: 1998-2005 (current age: 18-24 years)) 1963-1973 (current age: 50-59 years)`

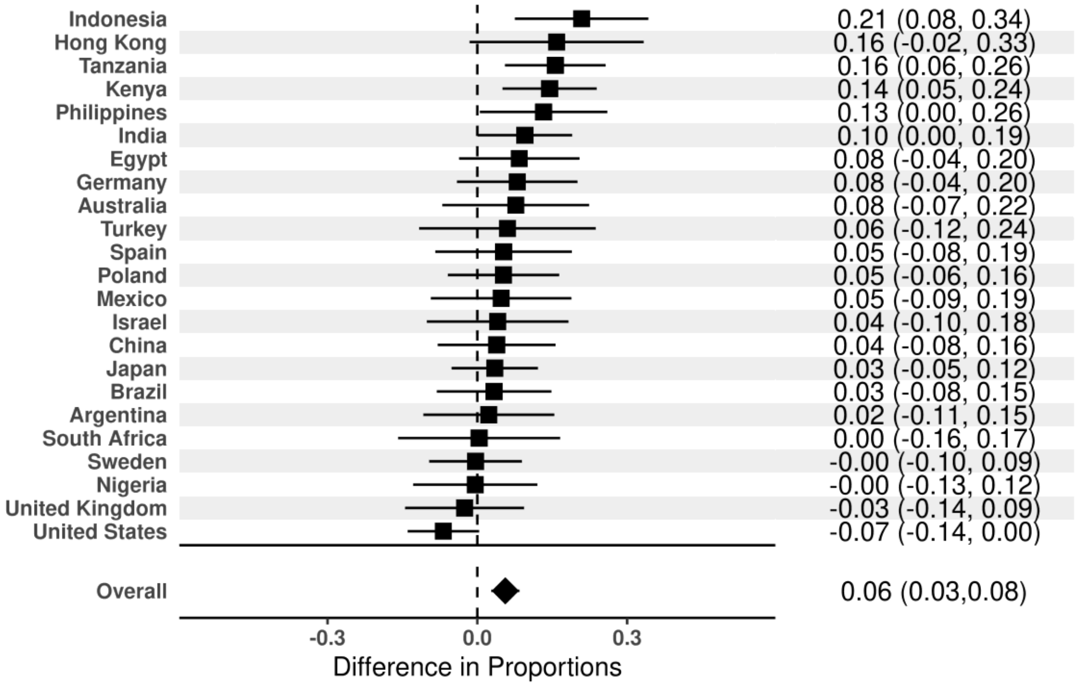

τ=0.034; Q(df=22)=32.90, p0.063;Q-profile 95% CI [0.000, 0.078]; I^2=25.26;

Figure S39. Forest plot for `Age group`-`(Ref: 1998-2005 (current age: 18-24 years)) 1973-1983 (current age: 40-49 years)`

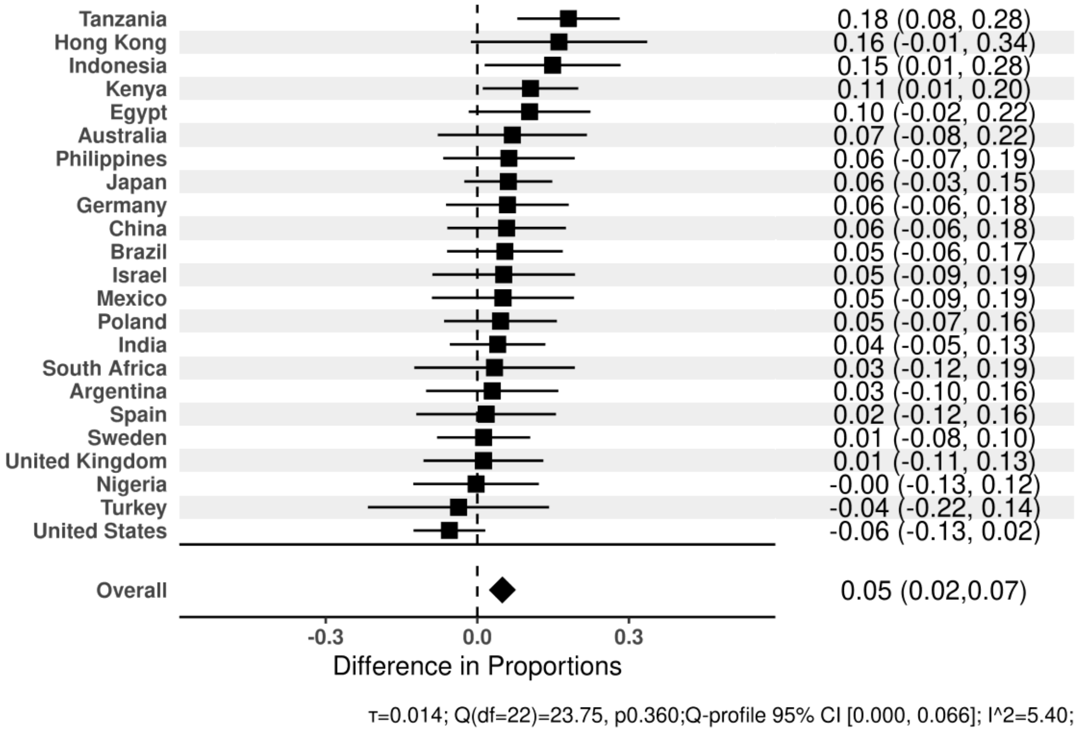

Figure S40. Forest plot for `Age group`-`(Ref: 1998-2005 (current age: 18-24 years)) 1983-1993 (current age: 30-39 years)`

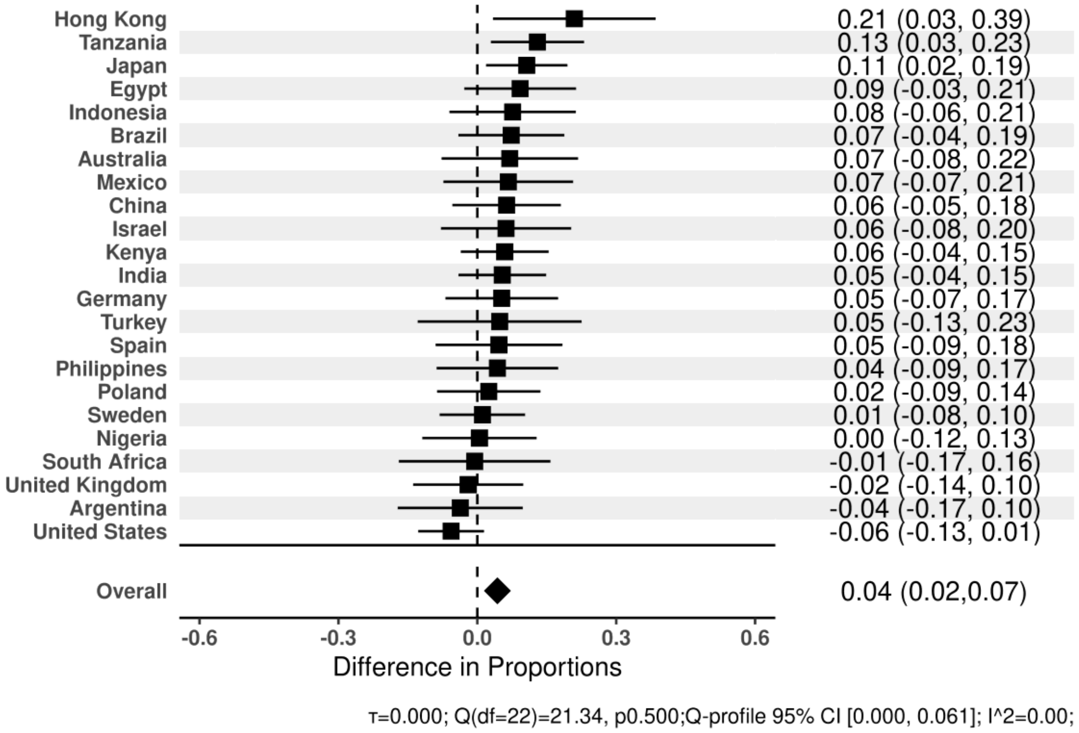

Figure S41. Forest plot for `Age group`-`(Ref: 1998-2005 (current age: 18-24 years)) 1993-1998 (current age: 25-29 years)`

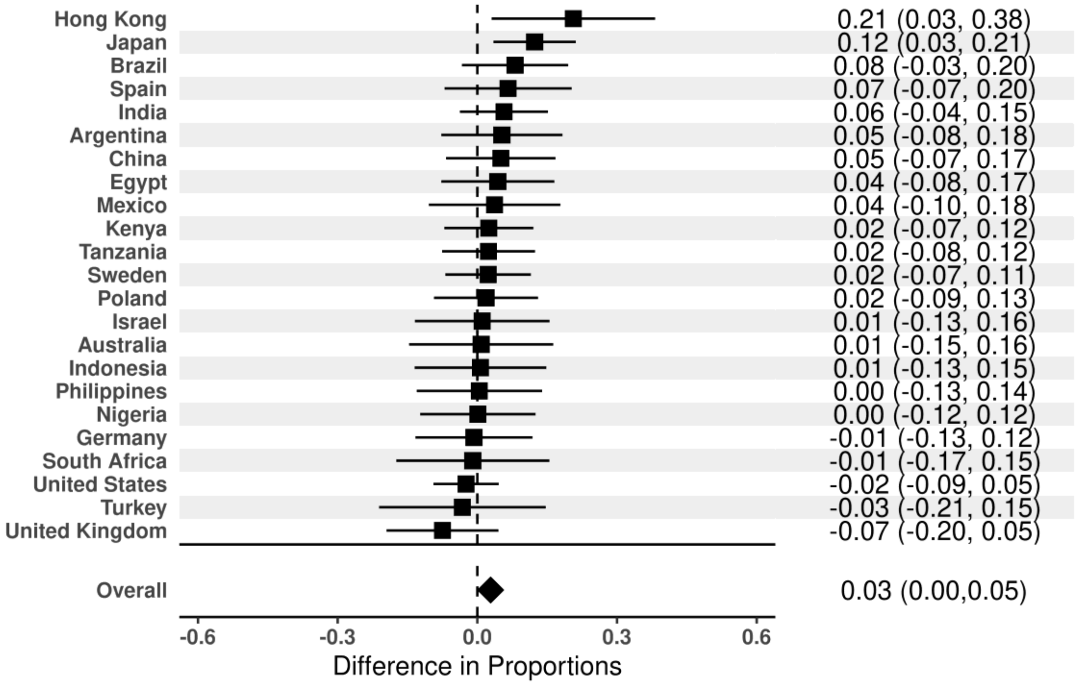

τ=0.000; Q(df=22)=16.67, p0.781;Q-profile 95% CI [0.000, 0.045]; I^2=0.00;

Figure S42. Forest plot for `Age group`-`(Ref: 80 or older) 1943-1953 (current age: 70-79 years)`

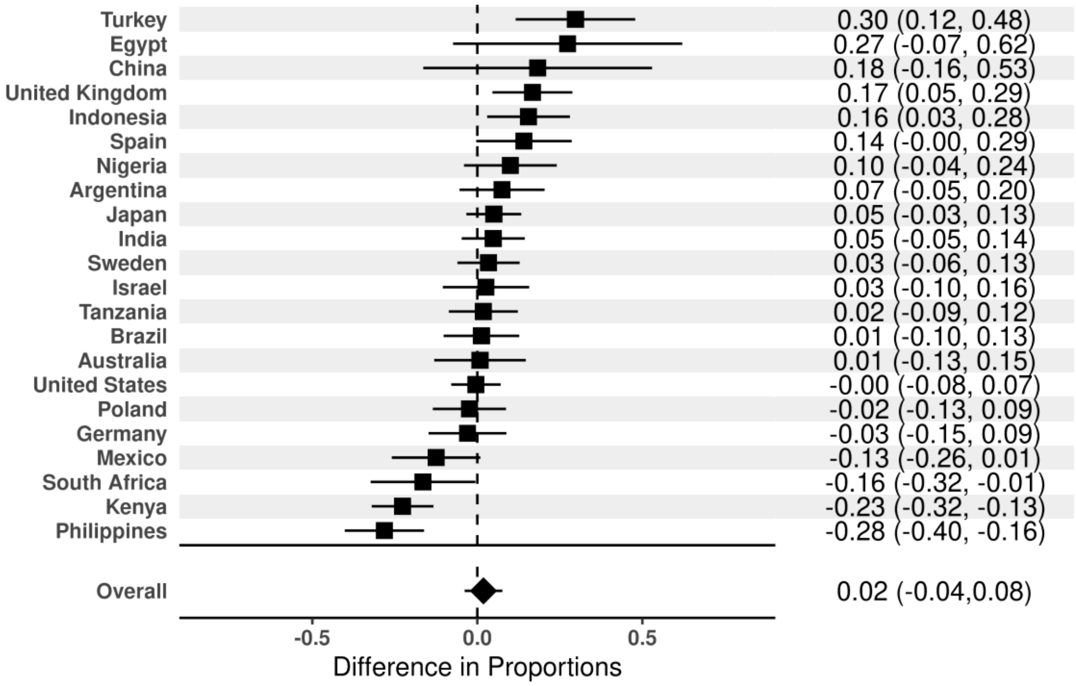

τ=0.118; Q(df=21)=88.45, p<.001;Q-profile 95% CI [0.072, 0.171]; I^2=79.26;
Excluded countries: Hong Kong

Figure S43. Forest plot for `Age group`-`(Ref: 80 or older) 1953-1963 (current age: 60-69 years)`

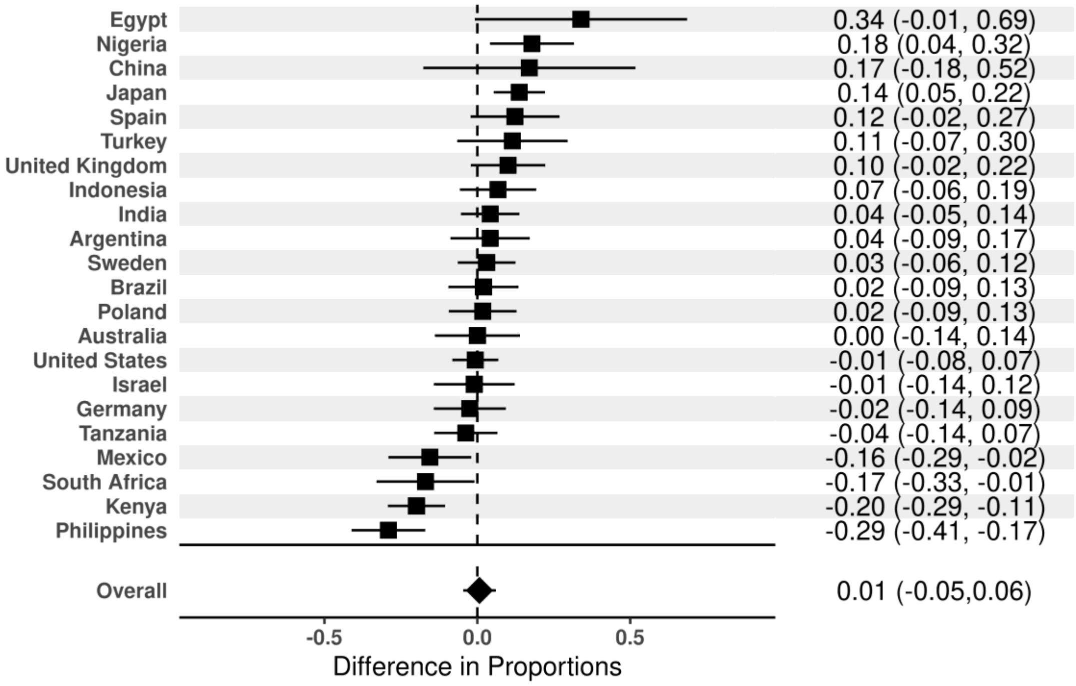

τ=0.108; Q(df=21)=81.08, p<.001;Q-profile 95% CI [0.063, 0.156]; I^2=76.03;
Excluded countries: Hong Kong

Figure S44. Forest plot for `Age group`-`(Ref: 80 or older) 1963-1973 (current age: 50-59 years)`

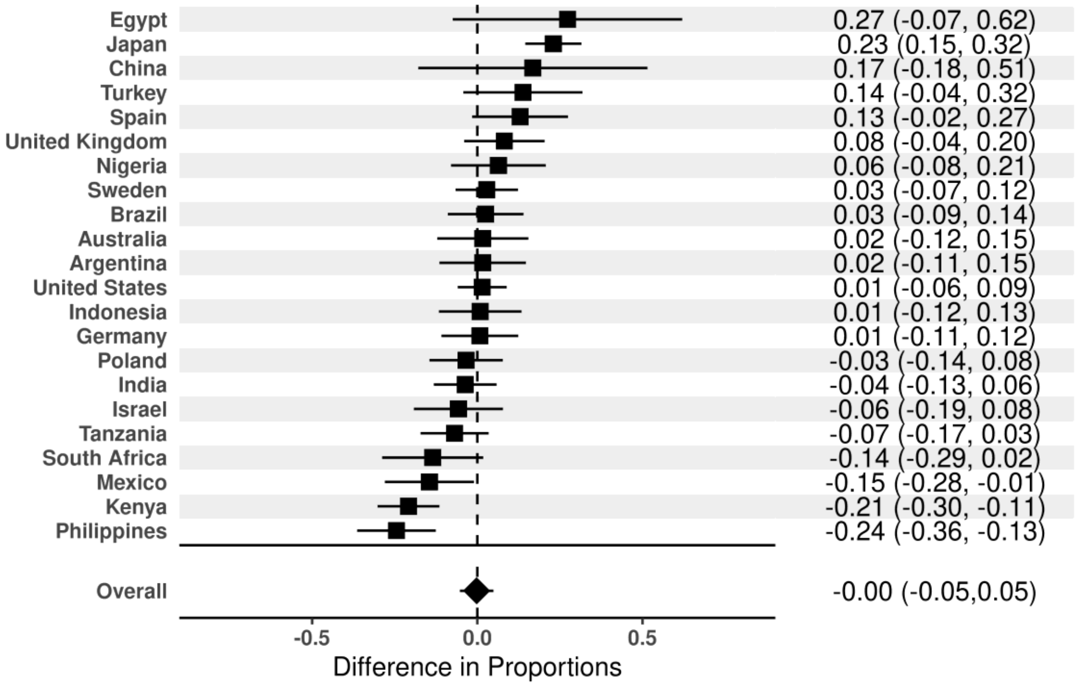

τ=0.101; Q(df=21)=86.05, p<.001;Q-profile 95% CI [0.064, 0.150]; I^2=73.53;
Excluded countries: Hong Kong

Figure S45. Forest plot for `Age group`-`(Ref: 80 or older) 1973-1983 (current age: 40-49 years)`

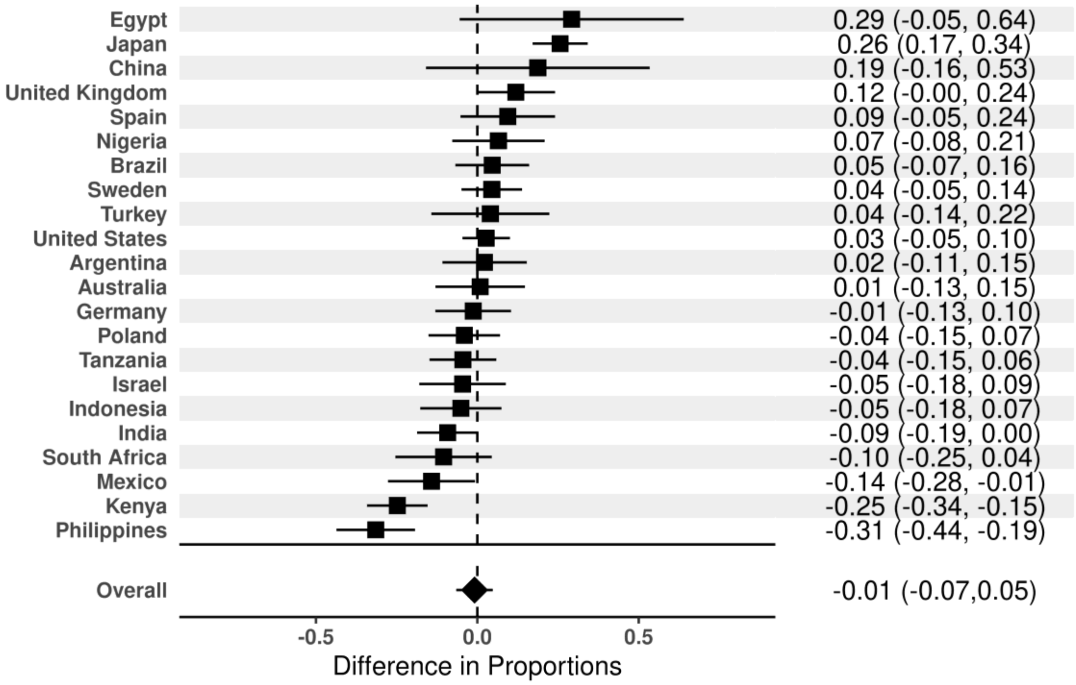

τ=0.117; Q(df=21)=111.03, p<.001;Q-profile 95% CI [0.078, 0.170]; I^2=78.81;
Excluded countries: Hong Kong

Figure S46. Forest plot for `Age group`-`(Ref: 80 or older) 1983-1993 (current age: 30-39 years)`

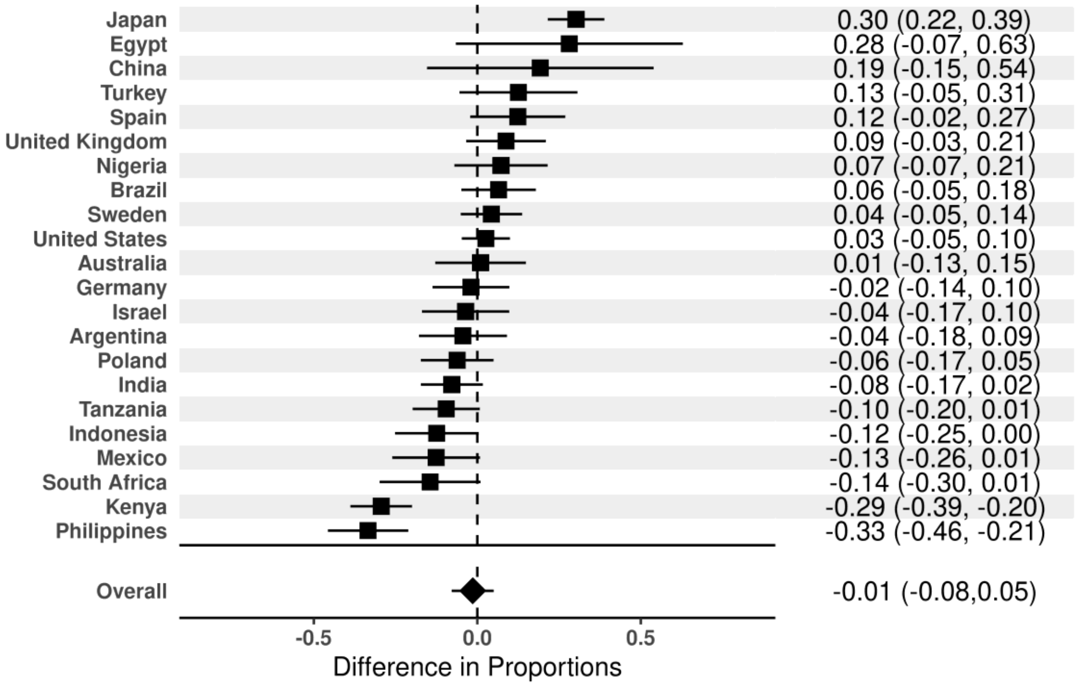

τ=0.136; Q(df=21)=143.09, p<.001;Q-profile 95% CI [0.093, 0.196]; I^2=83.49;
Excluded countries: Hong Kong

Figure S47. Forest plot for `Age group`-`(Ref: 80 or older) 1993-1998 (current age: 25-29 years)`

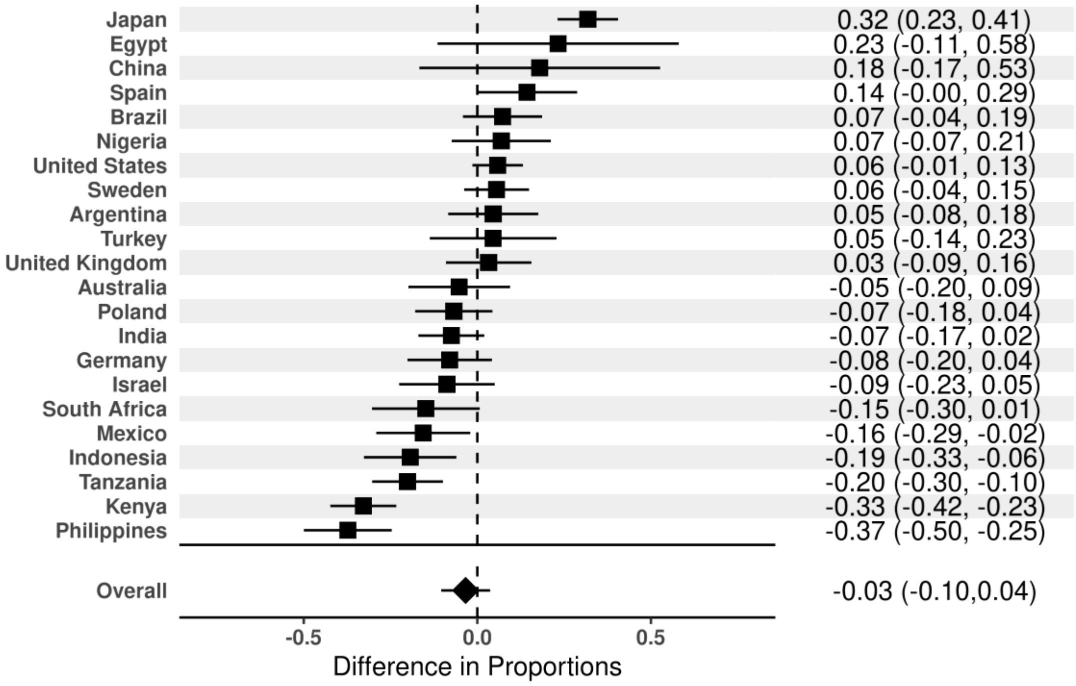

τ=0.153; Q(df=21)=178.59, p<.001;Q-profile 95% CI [0.108, 0.219]; I^2=86.22;
Excluded countries: Hong Kong

Figure S48. Forest plot for `Age group`-`(Ref: 1943-1953 (current age: 70-79 years)) 1953-1963 (current age: 60-69 years)`

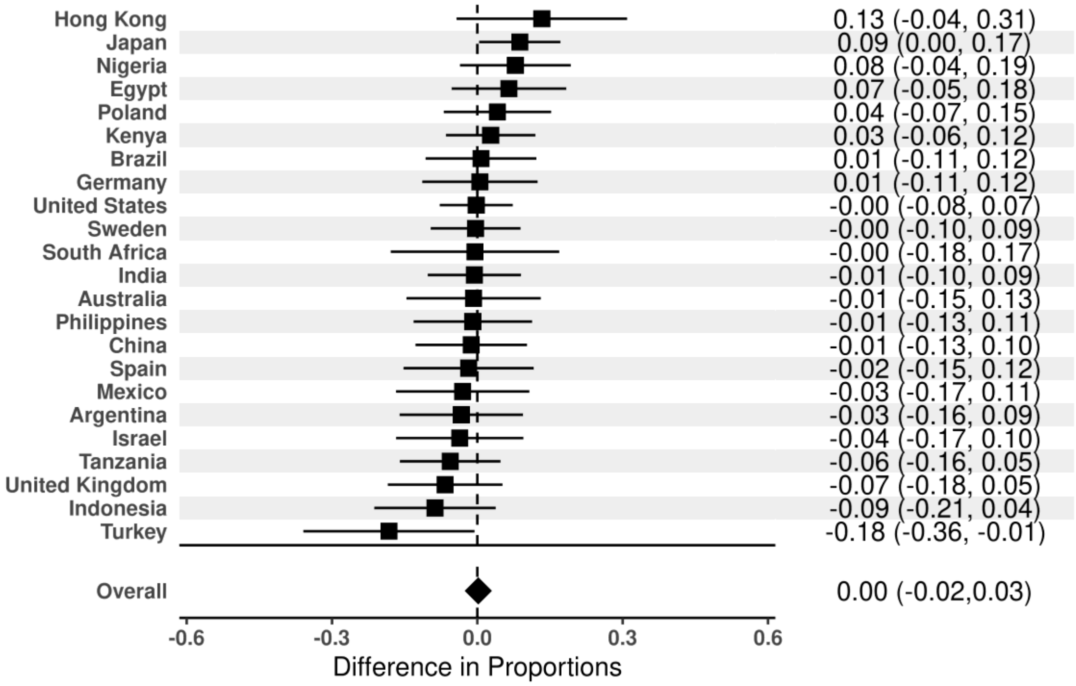

τ=0.000; Q(df=22)=19.47, p0.616;Q-profile 95% CI [0.000, 0.045]; I^2=0.00;

Figure S49. Forest plot for `Age group`-`(Ref: 1943-1953 (current age: 70-79 years)) 1963-1973 (current age: 50-59 years)`

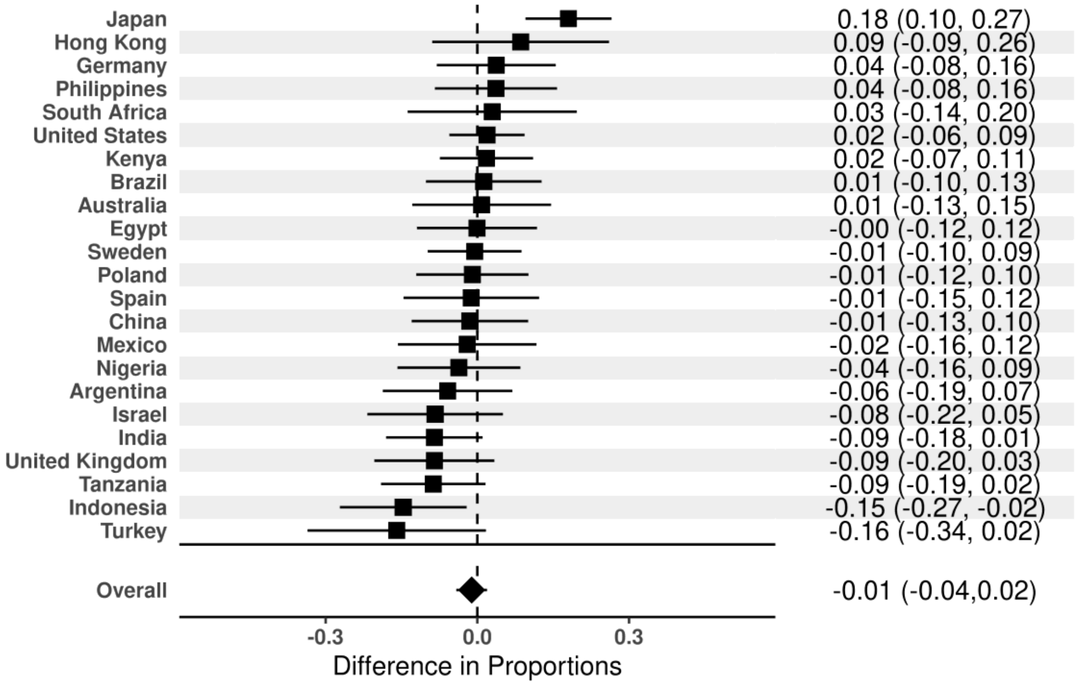

τ=0.044; Q(df=22)=38.16, p0.018;Q-profile 95% CI [0.017, 0.084]; I^2=36.61;

Figure S50. Forest plot for `Age group`-`(Ref: 1943-1953 (current age: 70-79 years)) 1973-1983 (current age: 40-49 years)`

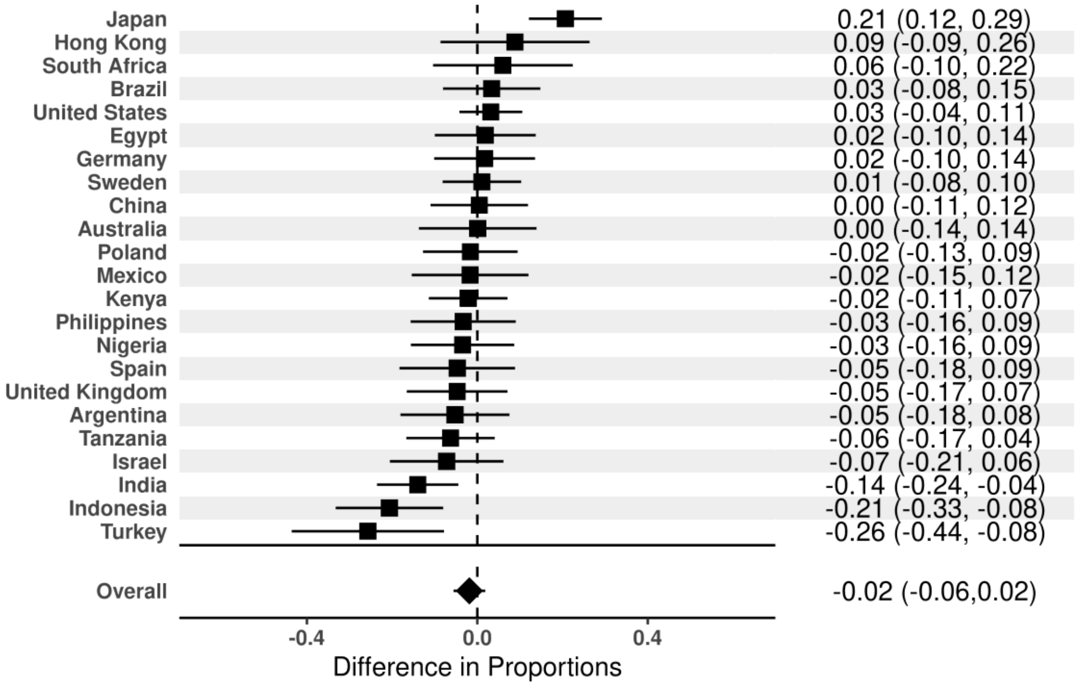

τ=0.068; Q(df=22)=56.11, p<.001;Q-profile 95% CI [0.038, 0.107]; I^2=57.57;

Figure S51. Forest plot for `Age group`-`(Ref: 1943-1953 (current age: 70-79 years)) 1983-1993 (current age: 30-39 years)`

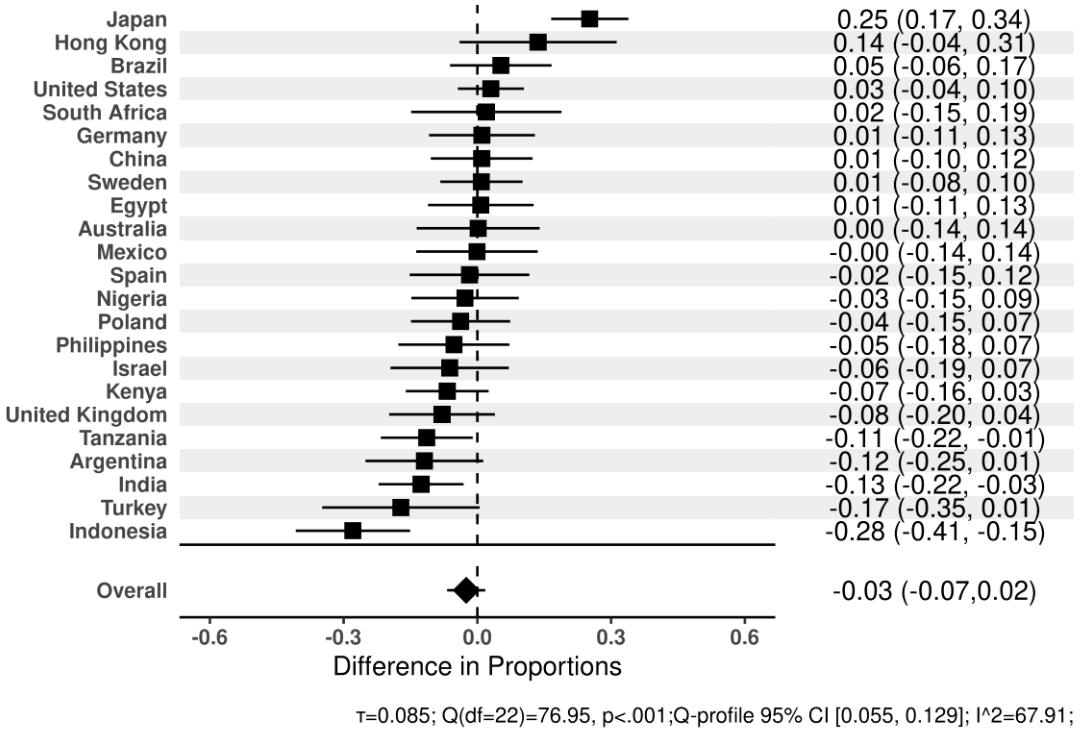

Figure S52. Forest plot for `Age group`-`(Ref: 1943-1953 (current age: 70-79 years)) 1993-1998 (current age: 25-29 years)`

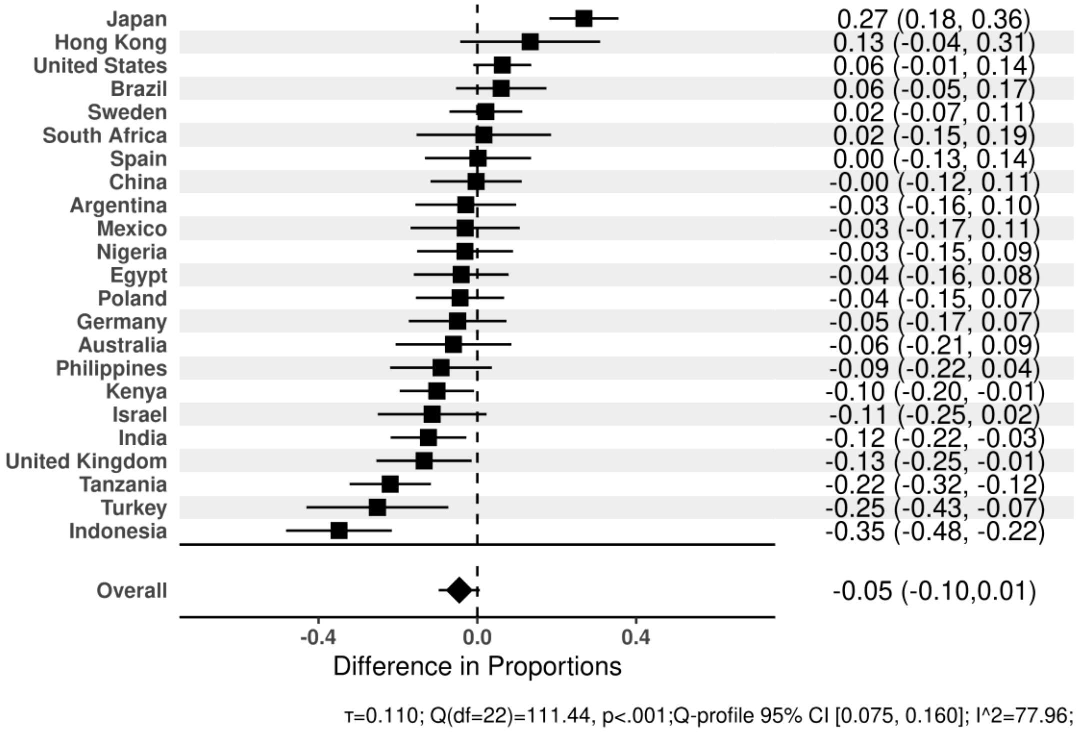

Figure S53. Forest plot for `Age group`-`(Ref: 1953-1963 (current age: 60-69 years)) 1963-1973 (current age: 50-59 years)`

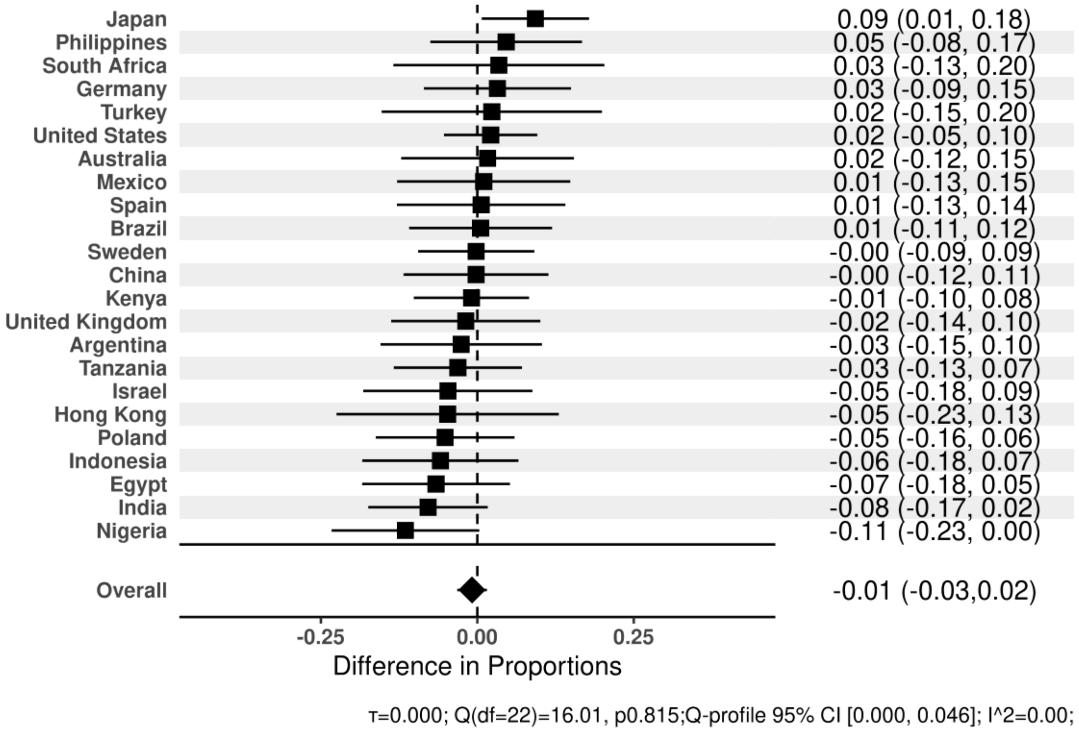

Figure S54. Forest plot for `Age group`-`(Ref: 1953-1963 (current age: 60-69 years)) 1973-1983 (current age: 40-49 years)`

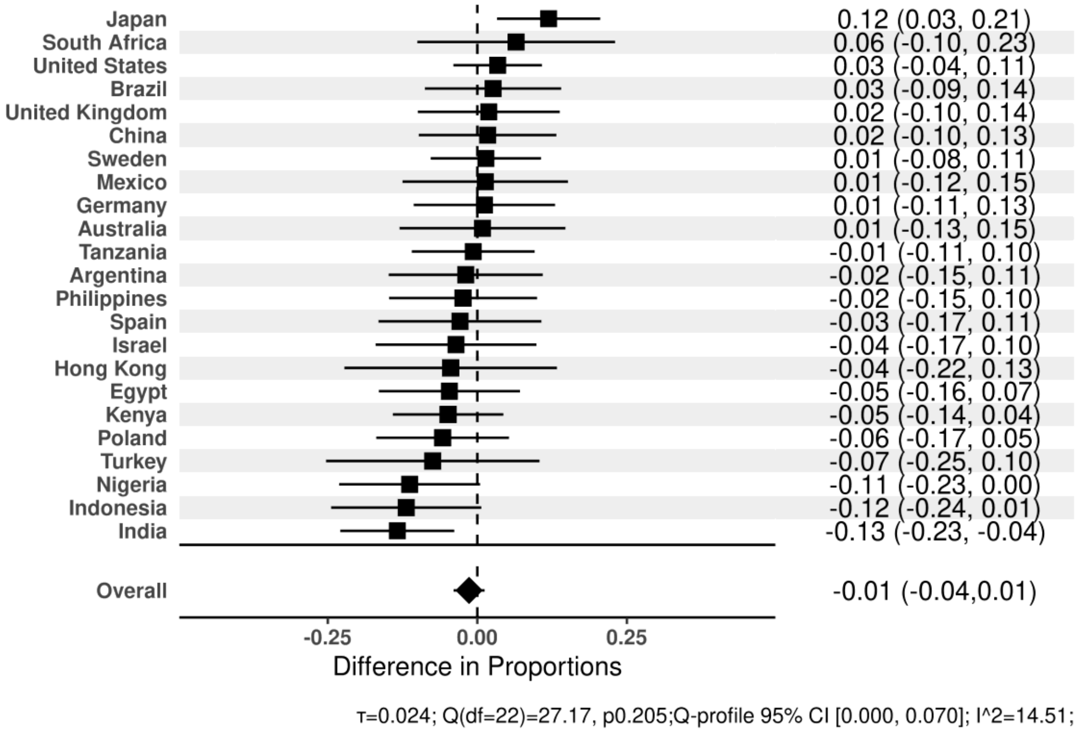

Figure S55. Forest plot for `Age group`-`(Ref: 1953-1963 (current age: 60-69 years)) 1983-1993 (current age: 30-39 years)`

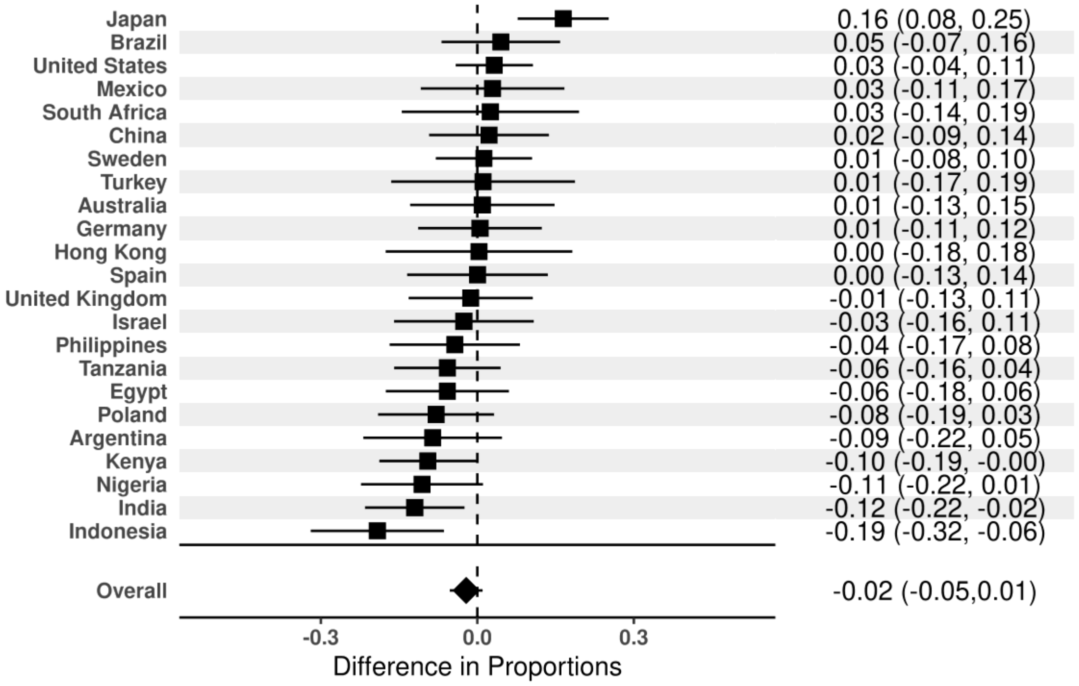

τ=0.048; Q(df=22)=41.58, p0.007;Q-profile 95% CI [0.023, 0.090]; I^2=40.35;

Figure S56. Forest plot for `Age group`-`(Ref: 1953-1963 (current age: 60-69 years)) 1993-1998 (current age: 25-29 years)`

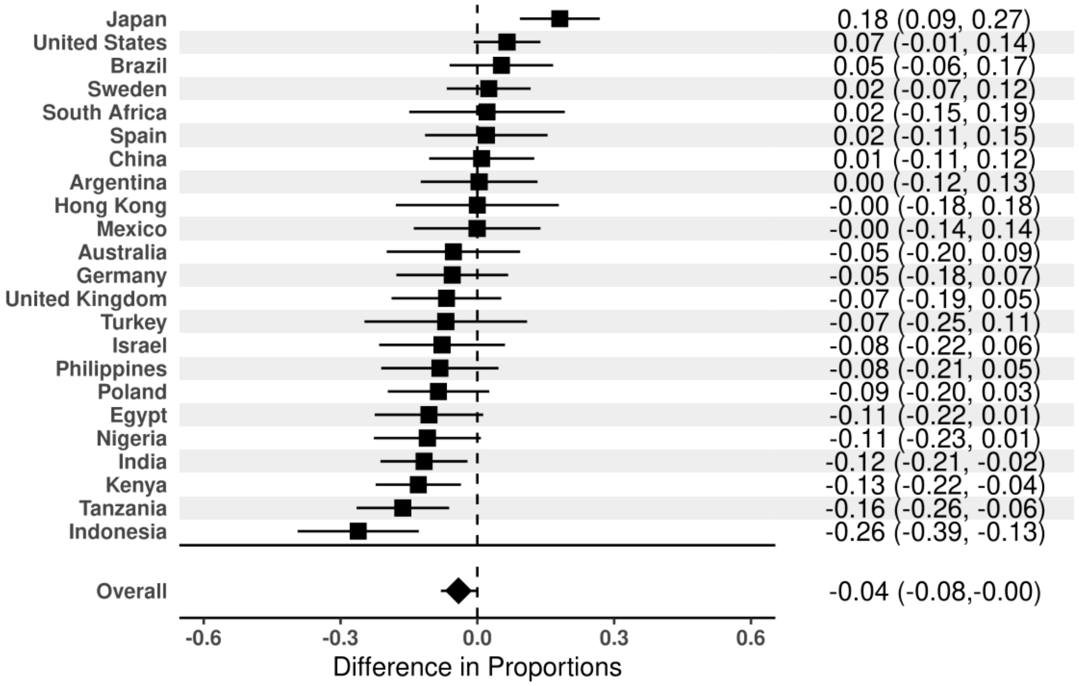

τ=0.072; Q(df=22)=65.93, p<.001;Q-profile 95% CI [0.047, 0.116]; I^2=60.10;

Figure S57. Forest plot for `Age group`-`(Ref: 1963-1973 (current age: 50-59 years)) 1973-1983 (current age: 40-49 years)`

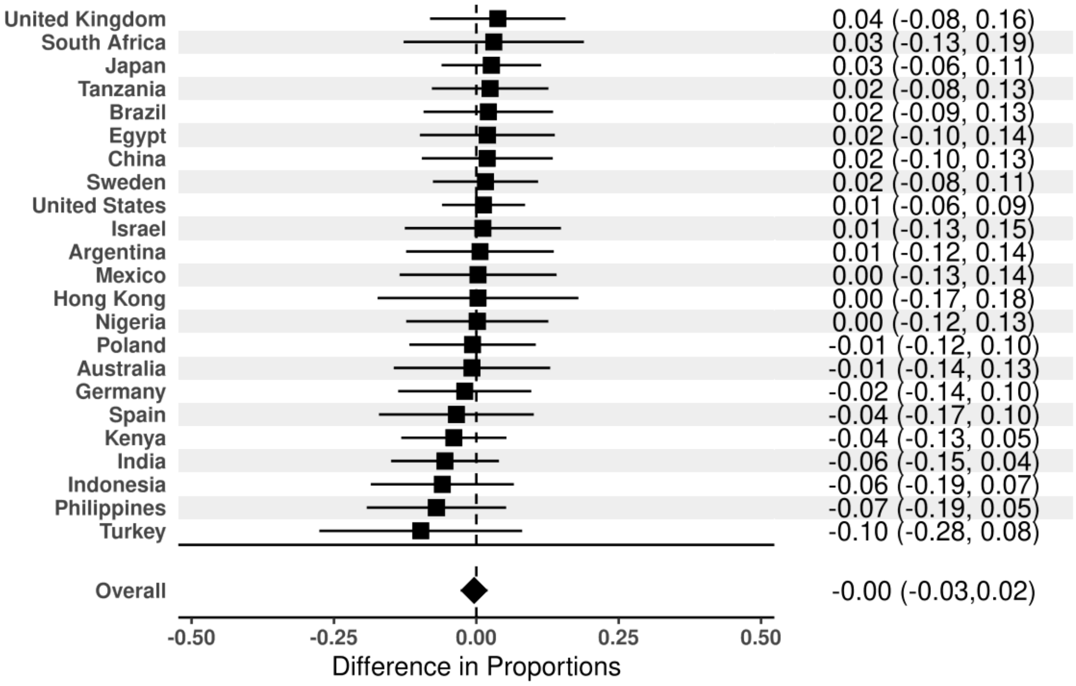

τ=0.000; Q(df=22)=7.29, p0.999;Q-profile 95% CI [0.000, 0.030]; I^2=0.00;

Figure S58. Forest plot for `Age group`-`(Ref: 1963-1973 (current age: 50-59 years)) 1983-1993 (current age: 30-39 years)`

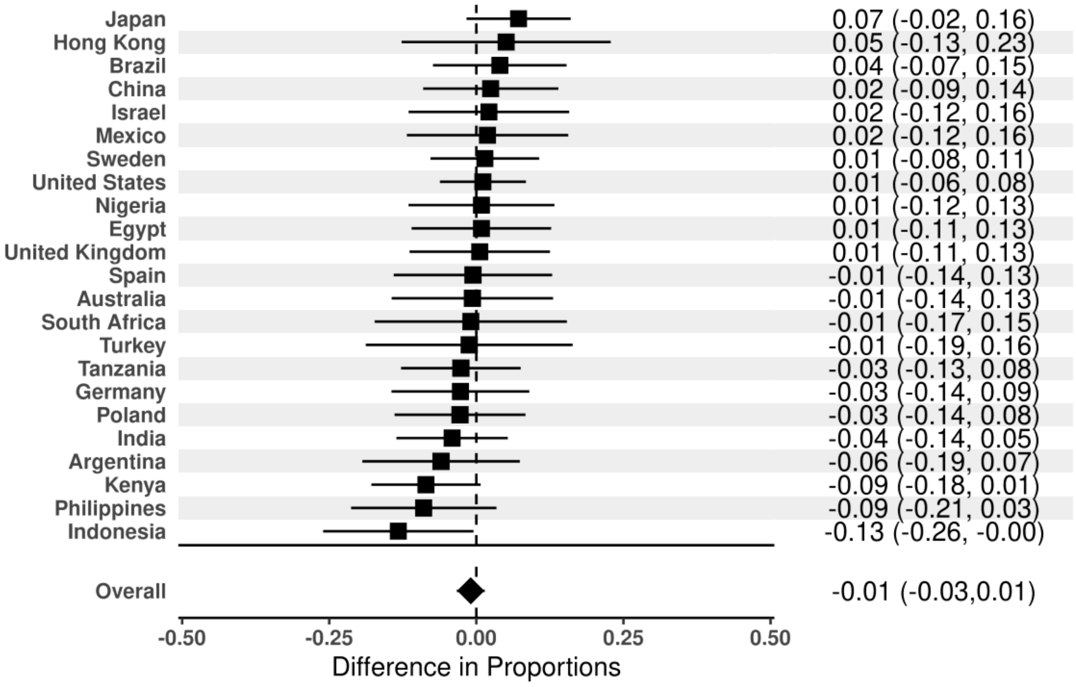

τ=0.000; Q(df=22)=14.93, p0.865;Q-profile 95% CI [0.000, 0.042]; I^2=0.00;

Figure S59. Forest plot for `Age group`-`(Ref: 1963-1973 (current age: 50-59 years)) 1993-1998 (current age: 25-29 years)`

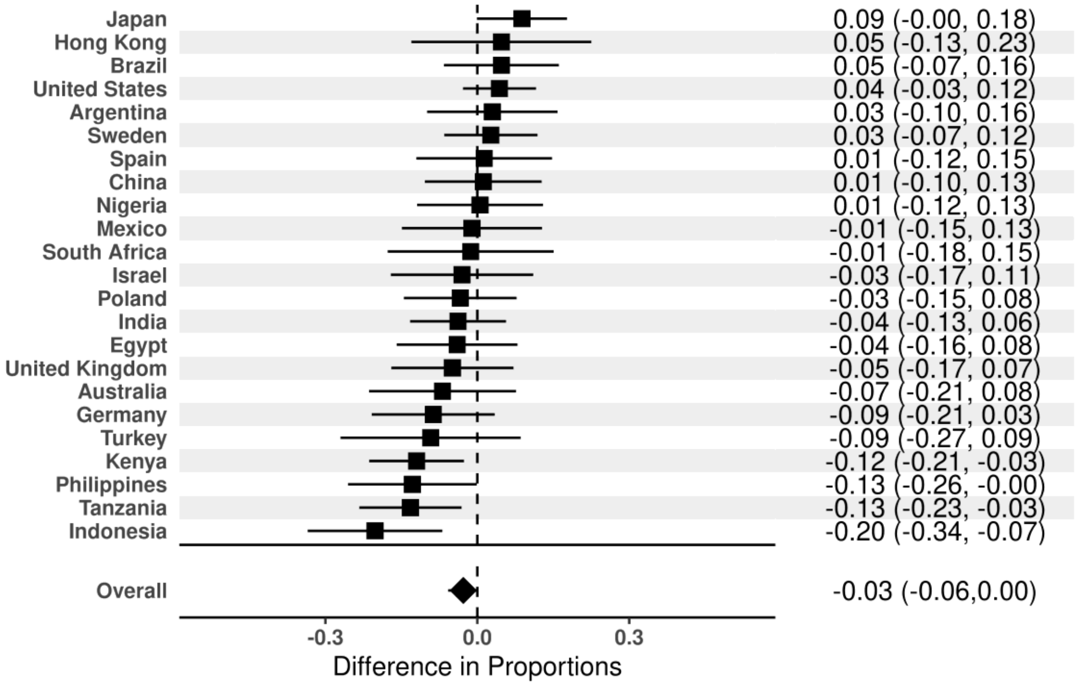

τ=0.041; Q(df=22)=34.68, p0.042;Q-profile 95% CI [0.000, 0.081]; I^2=32.38;

Figure S60. Forest plot for `Age group`-`(Ref: 1973-1983 (current age: 40-49 years)) 1983-1993 (current age: 30-39 years)`

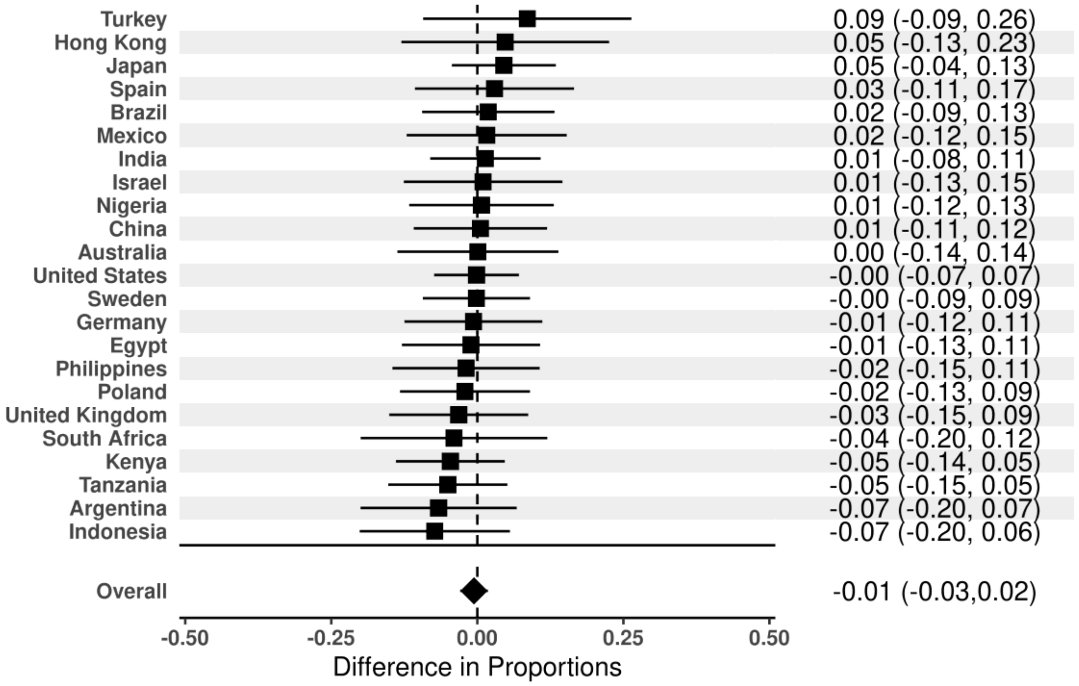

τ=0.000; Q(df=22)=7.26, p0.999;Q-profile 95% CI [0.000, 0.030]; I^2=0.00;

Figure S61. Forest plot for `Age group`-`(Ref: 1973-1983 (current age: 40-49 years)) 1993-1998 (current age: 25-29 years)`

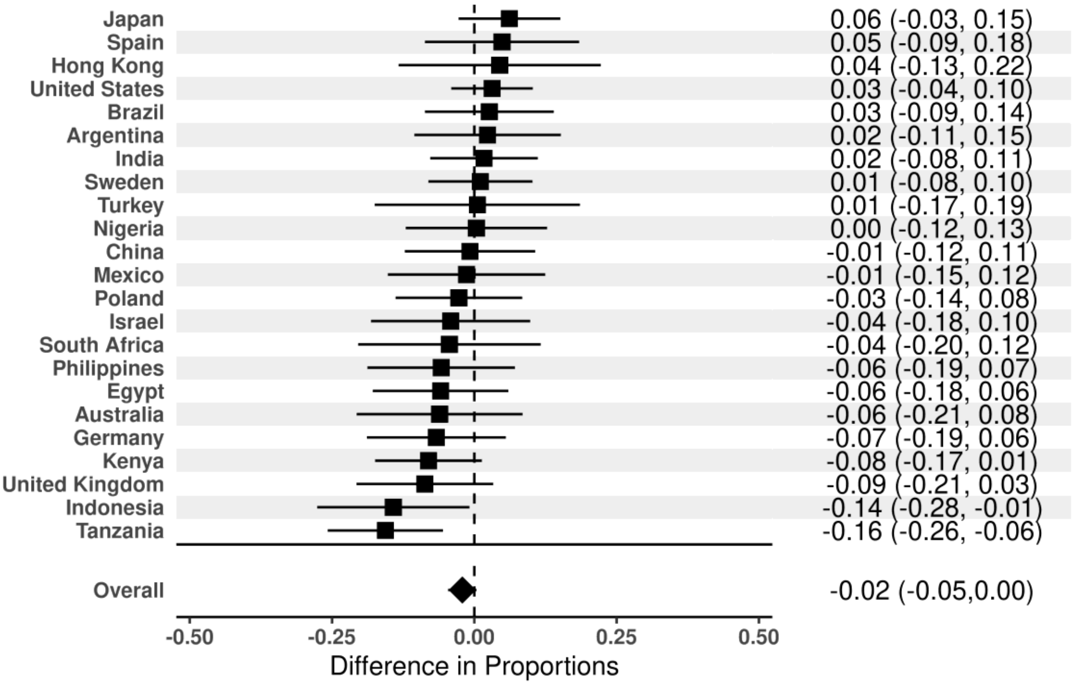

τ=0.016; Q(df=22)=23.82, p0.357;Q-profile 95% CI [0.000, 0.064]; I^2=6.71;

Figure S62. Forest plot for `Age group`-`(Ref: 1983-1993 (current age: 30-39 years)) 1993-1998 (current age: 25-29 years)`

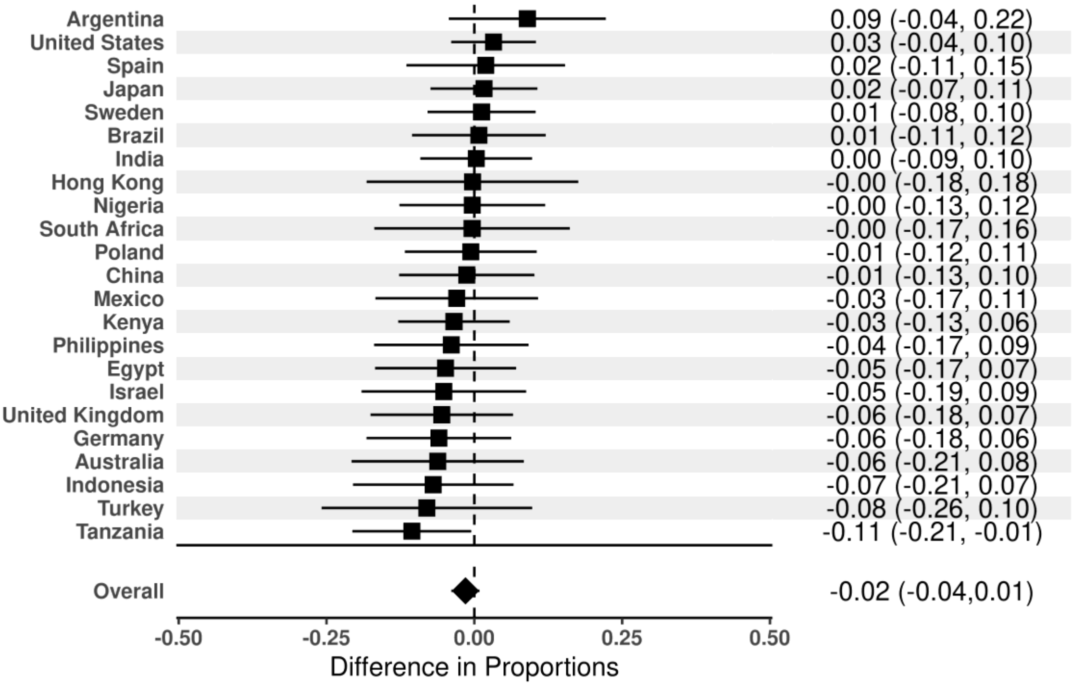

τ=0.000; Q(df=22)=12.03, p0.957;Q-profile 95% CI [0.000, 0.037]; I^2=0.00;

Figure S63. Forest plot for `Gender`-`(Ref: Male) Female`

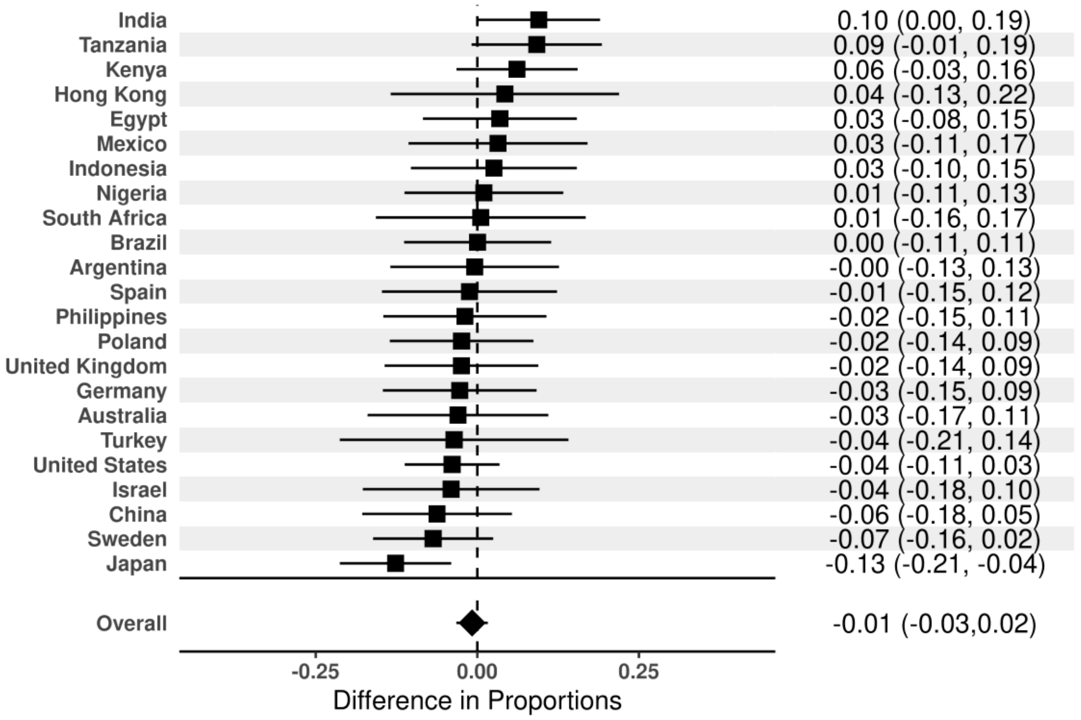

τ=0.012; Q(df=22)=23.09, p0.397;Q-profile 95% CI [0.000, 0.066]; I^2=3.93;

Figure S64. Forest plot for `Gender`-`(Ref: Male) Other`

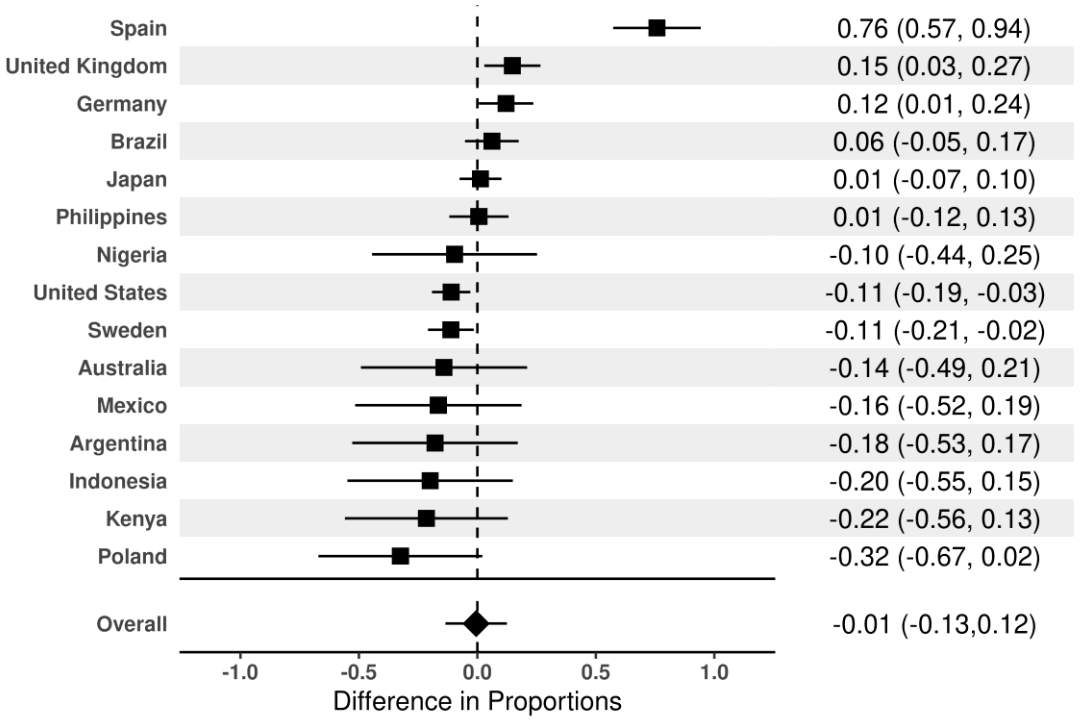

τ=0.228; Q(df=14)=96.88, p<.001;Q-profile 95% CI [0.142, 0.357]; I^2=90.48;
Excluded countries: Hong Kong, India, Egypt, Israel, South Africa, Tanzania, Turkiye

Figure S65. Forest plot for `Gender`-`(Ref: Female) Other`

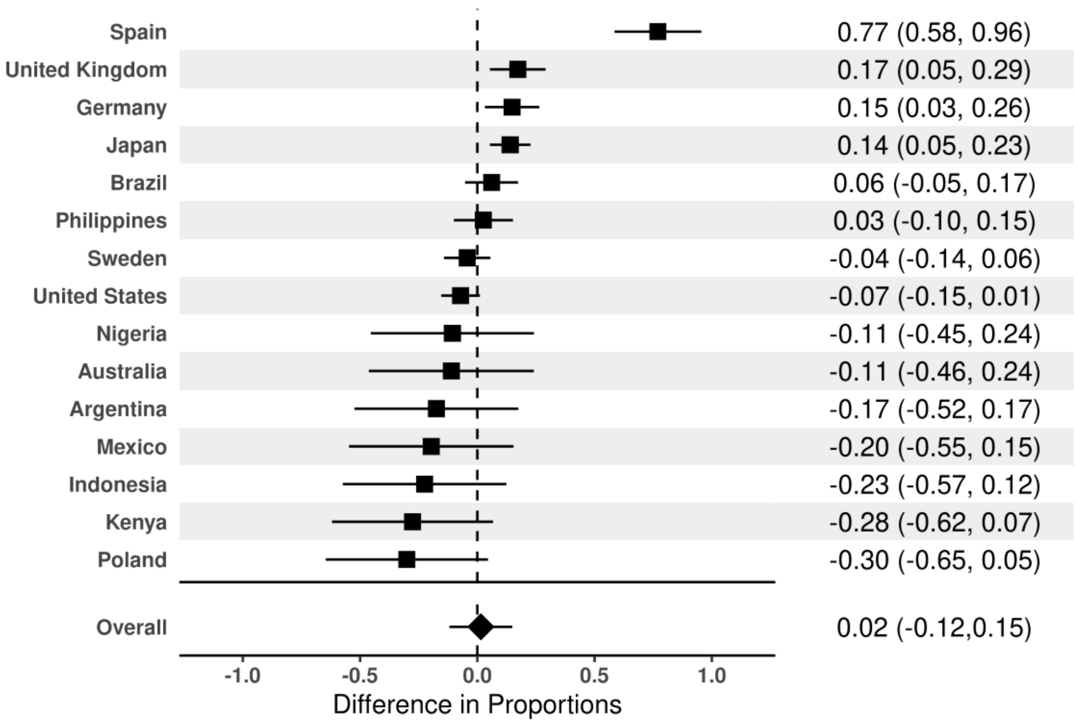

τ=0.237; Q(df=14)=95.69, p<.001;Q-profile 95% CI [0.145, 0.369]; I^2=91.05;
Excluded countries: Hong Kong, India, Egypt, Israel, South Africa, Tanzania, Turkiye

Figure S66. Forest plot for `Marital status`-`(Ref: Single, never married) Divorced`

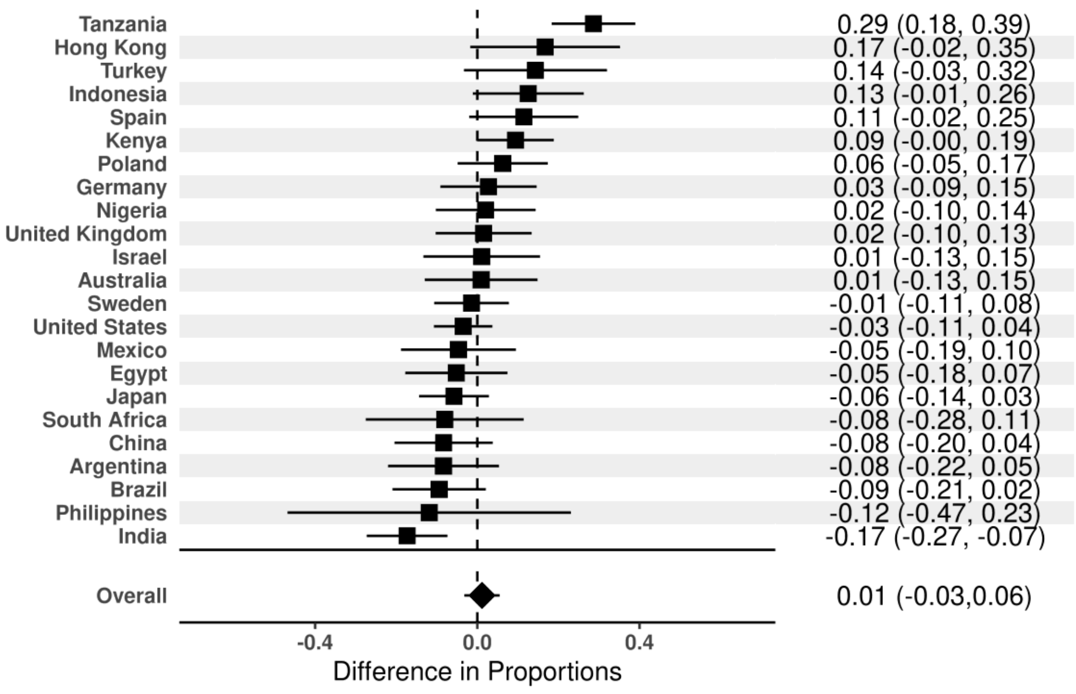

τ=0.084; Q(df=22)=68.83, p<.001;Q-profile 95% CI [0.053, 0.130]; I^2=65.82;

Figure S67. Forest plot for `Marital status`-`(Ref: Single, never married) Domestic partner`

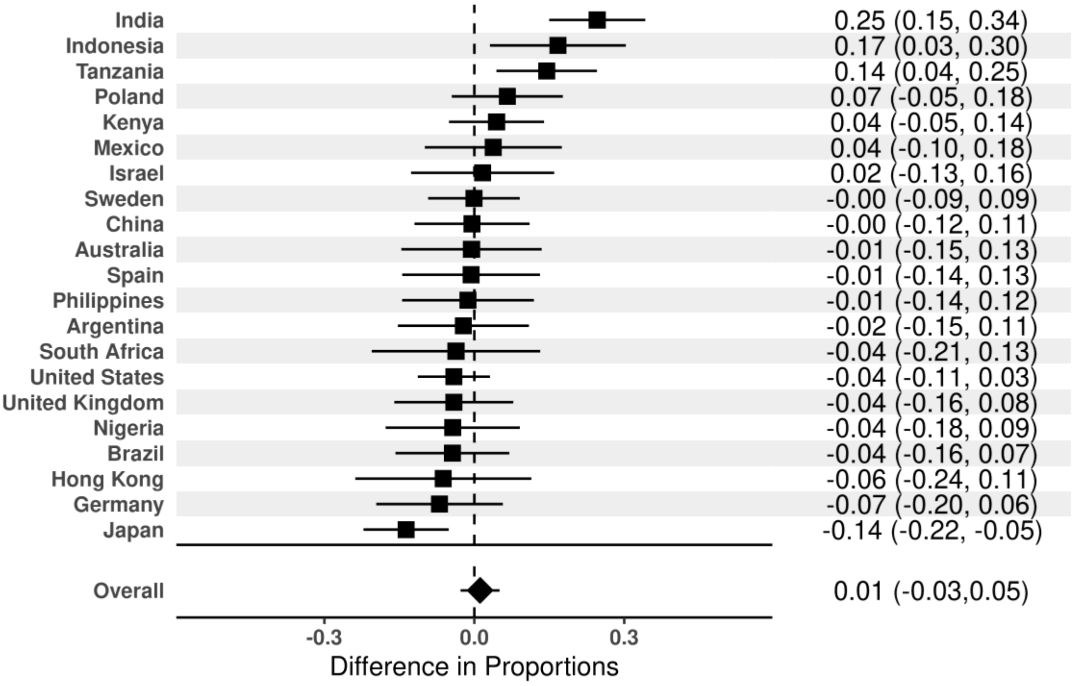

τ=0.068; Q(df=20)=55.22, p<.001;Q-profile 95% CI [0.042, 0.113]; I^2=57.46;
Excluded countries: Egypt, Turkiye

Figure S68. Forest plot for `Marital status`-`(Ref: Single, never married) Married`

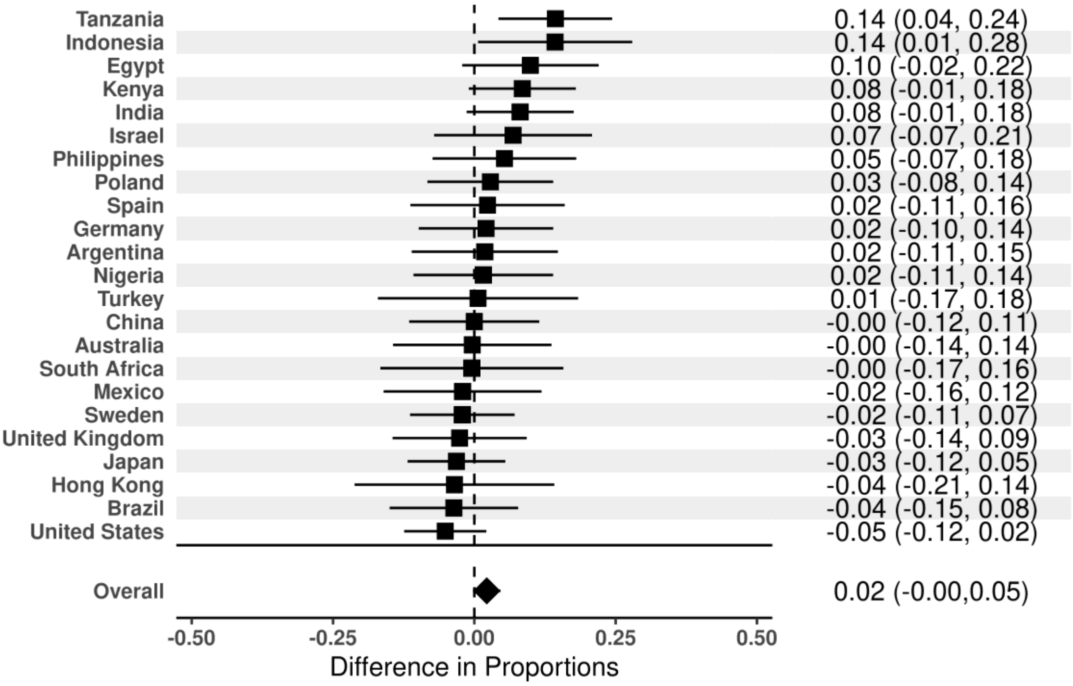

τ=0.012; Q(df=22)=23.09, p0.397;Q-profile 95% CI [0.000, 0.063]; I^2=4.20;

Figure S69. Forest plot for `Marital status`-`(Ref: Single, never married) Separated`

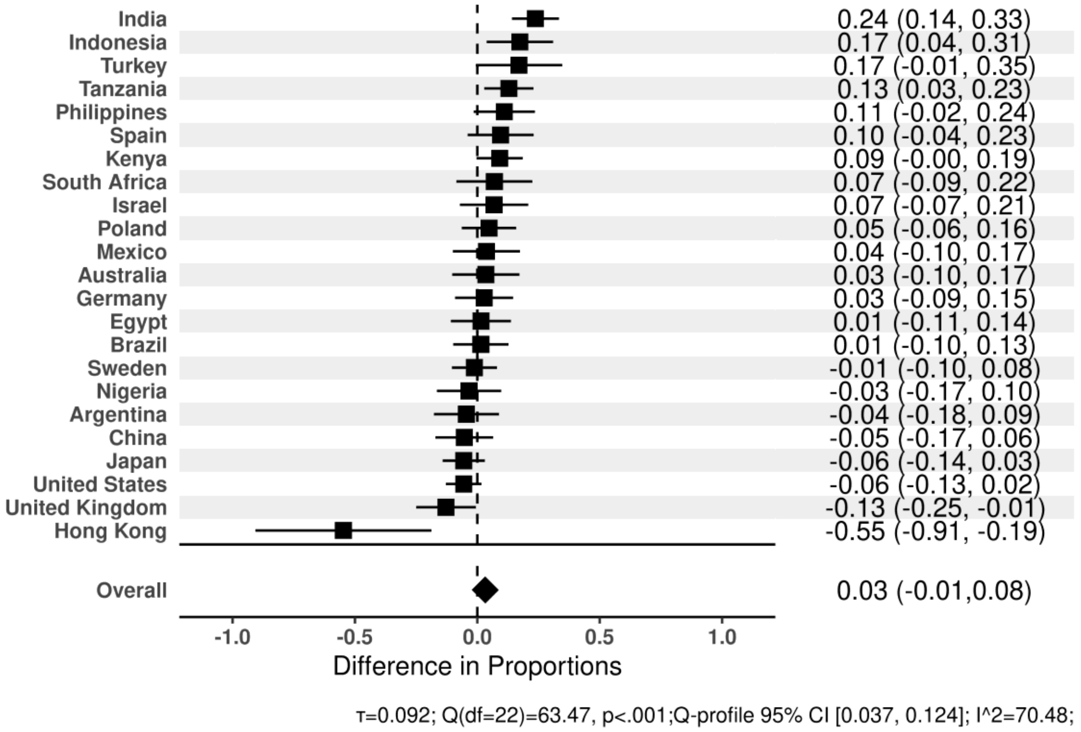

Figure S70. Forest plot for `Marital status`-`(Ref: Single, never married) Widowed`

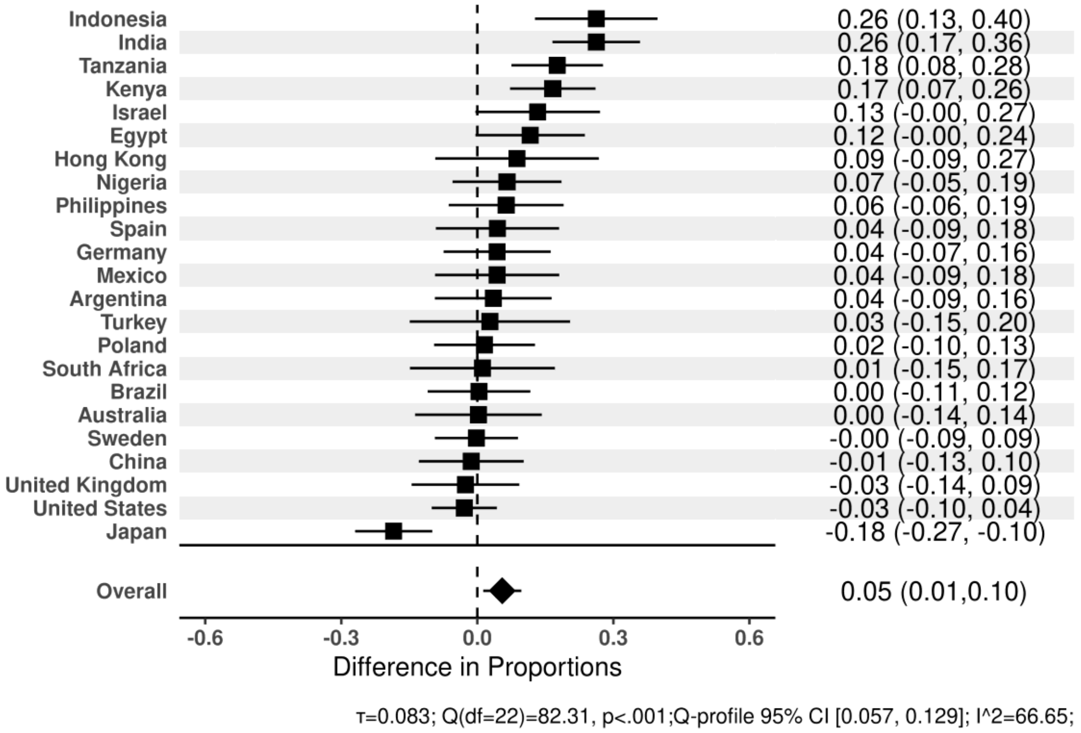

Figure S71. Forest plot for `Marital status`-`(Ref: Divorced) Domestic partner`

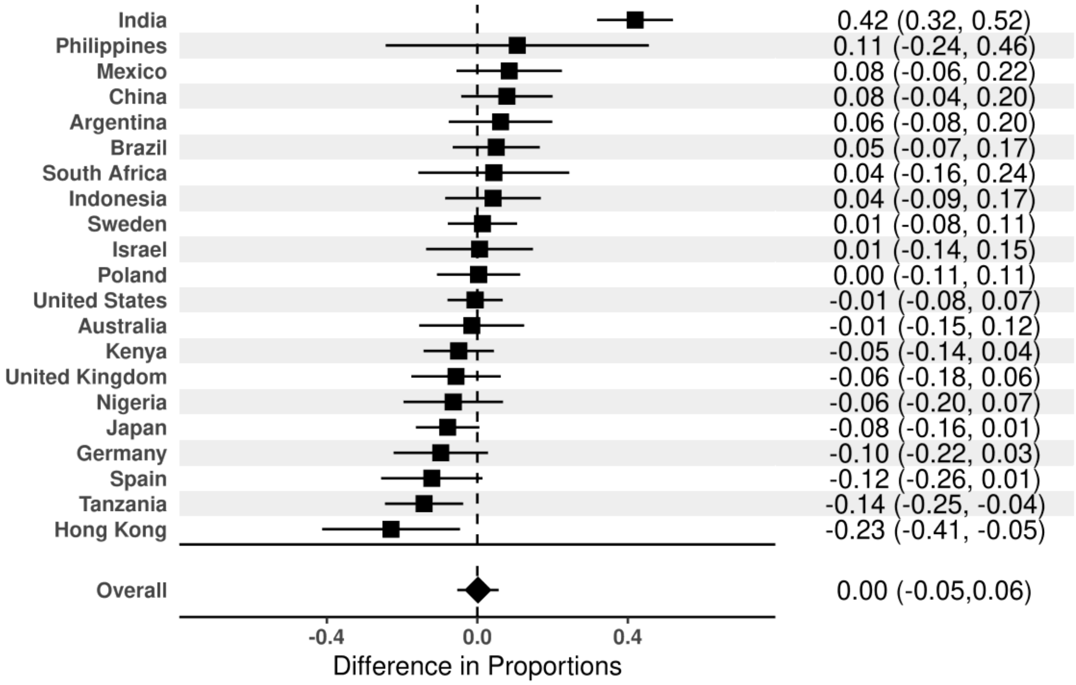

τ=0.110; Q(df=20)=96.66, p<.001;Q-profile 95% CI [0.074, 0.166]; I^2=77.13;
Excluded countries: Egypt, Turkiye

Figure S72. Forest plot for `Marital status`-`(Ref: Divorced) Married`

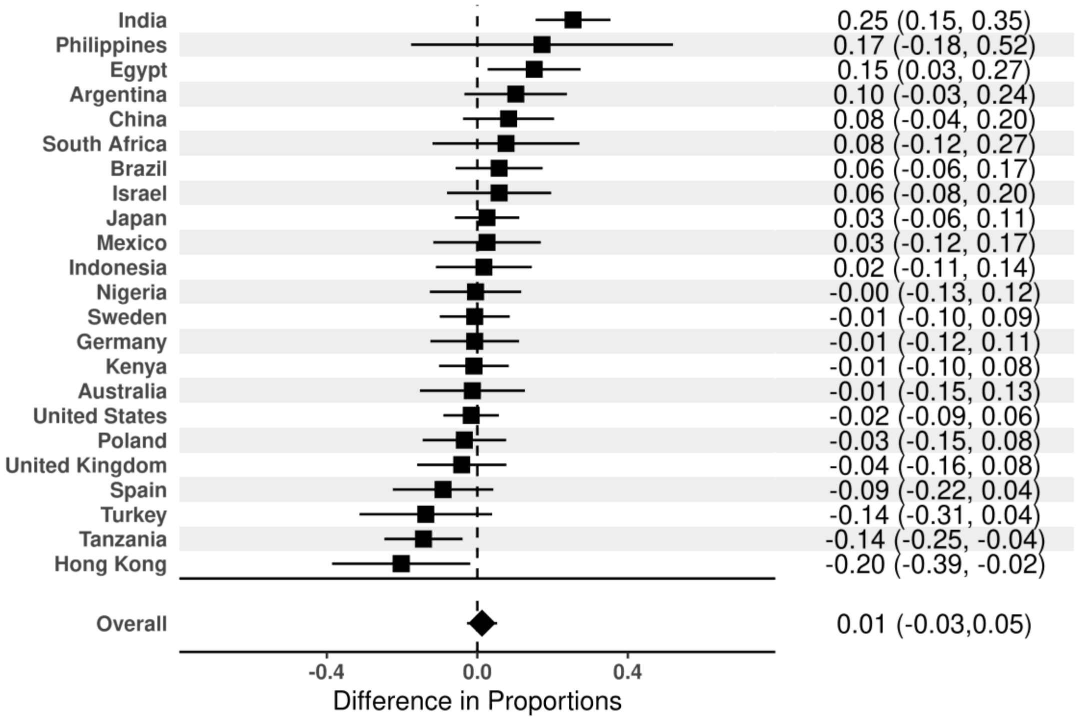

τ=0.074; Q(df=22)=54.55, p<.001;Q-profile 95% CI [0.038, 0.115]; I^2=59.88;

Figure S73. Forest plot for `Marital status`-`(Ref: Divorced) Separated`

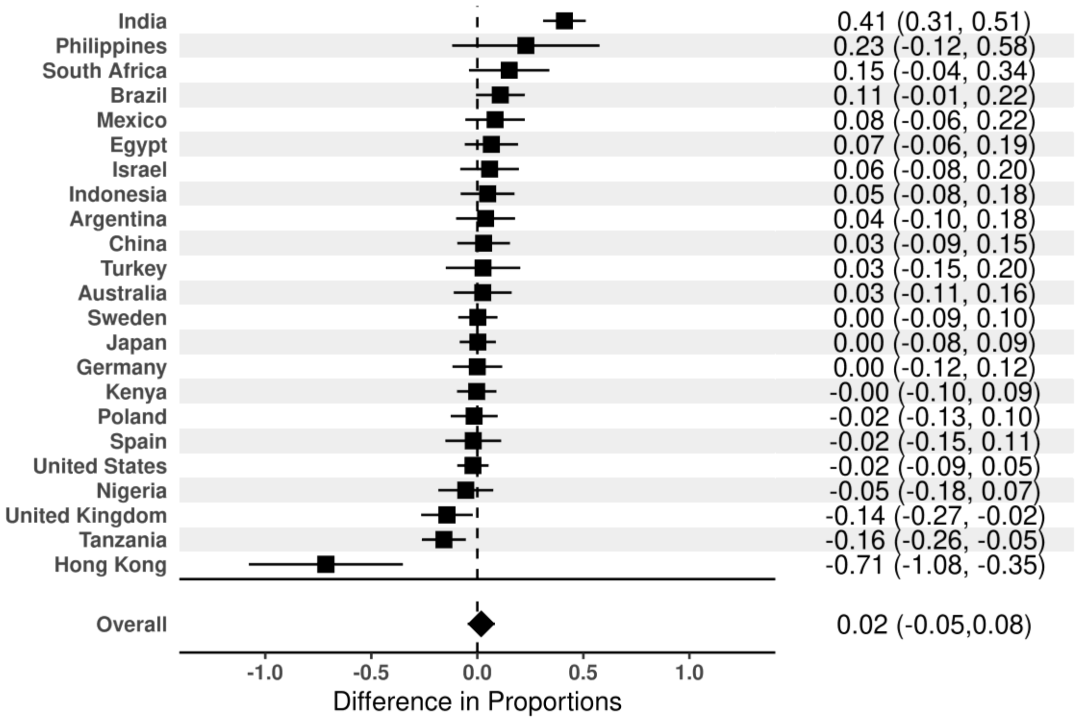

τ=0.140; Q(df=22)=102.84, p<.001;Q-profile 95% CI [0.068, 0.184]; I^2=84.11;

Figure S74. Forest plot for `Marital status`-`(Ref: Divorced) Widowed`

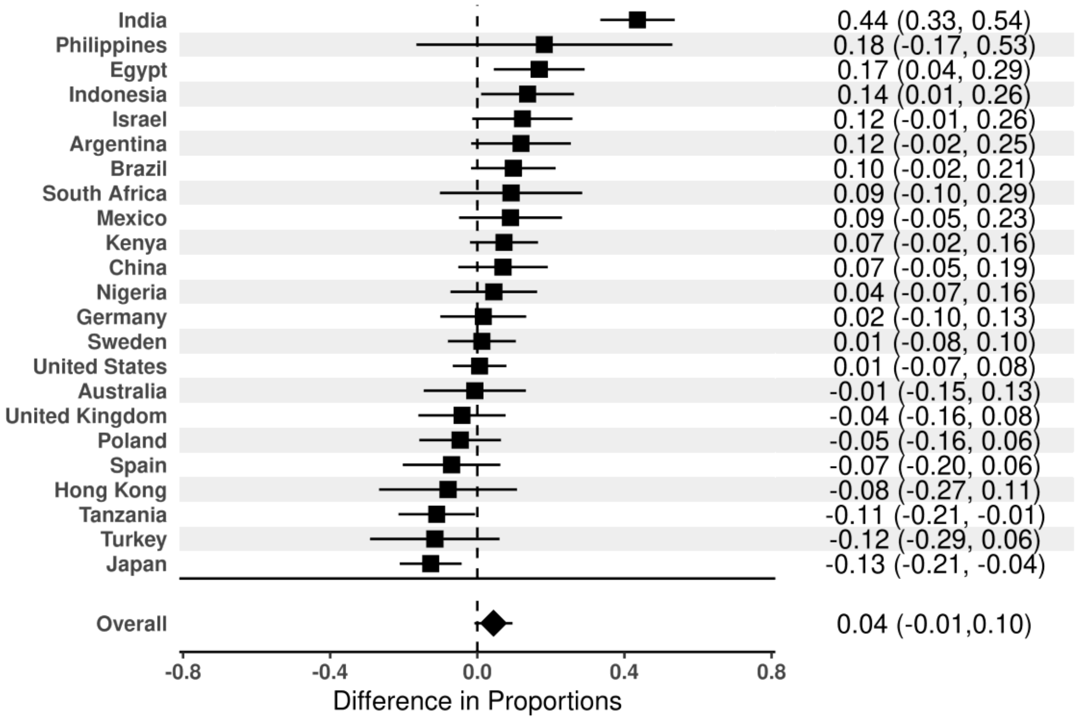

τ=0.108; Q(df=22)=107.16, p<.001;Q-profile 95% CI [0.075, 0.161]; I^2=76.39;

Figure S75. Forest plot for `Marital status`-`(Ref: Domestic partner) Married`

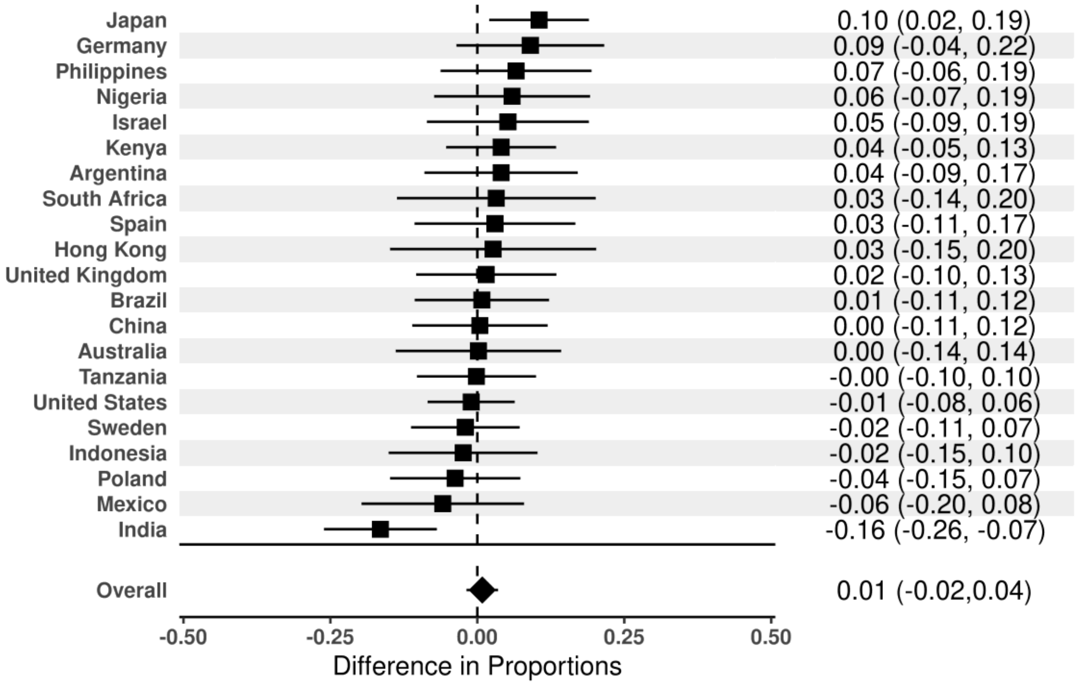

τ=0.023; Q(df=20)=24.23, p0.233;Q-profile 95% CI [0.000, 0.071]; I^2=13.59;
Excluded countries: Egypt, Turkiye

Figure S76. Forest plot for `Marital status`-`(Ref: Domestic partner) Separated`

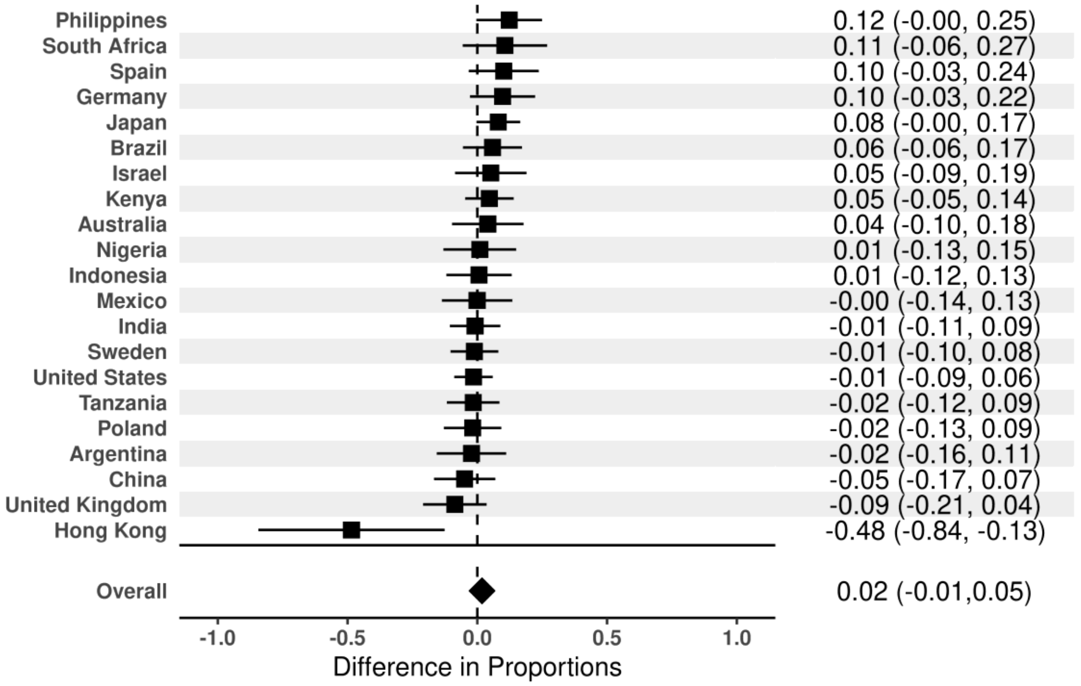

τ=0.033; Q(df=20)=24.40, p0.225;Q-profile 95% CI [0.000, 0.060]; I^2=24.02;
Excluded countries: Egypt, Turkiye

Figure S77. Forest plot for `Marital status`-`(Ref: Domestic partner) Widowed`

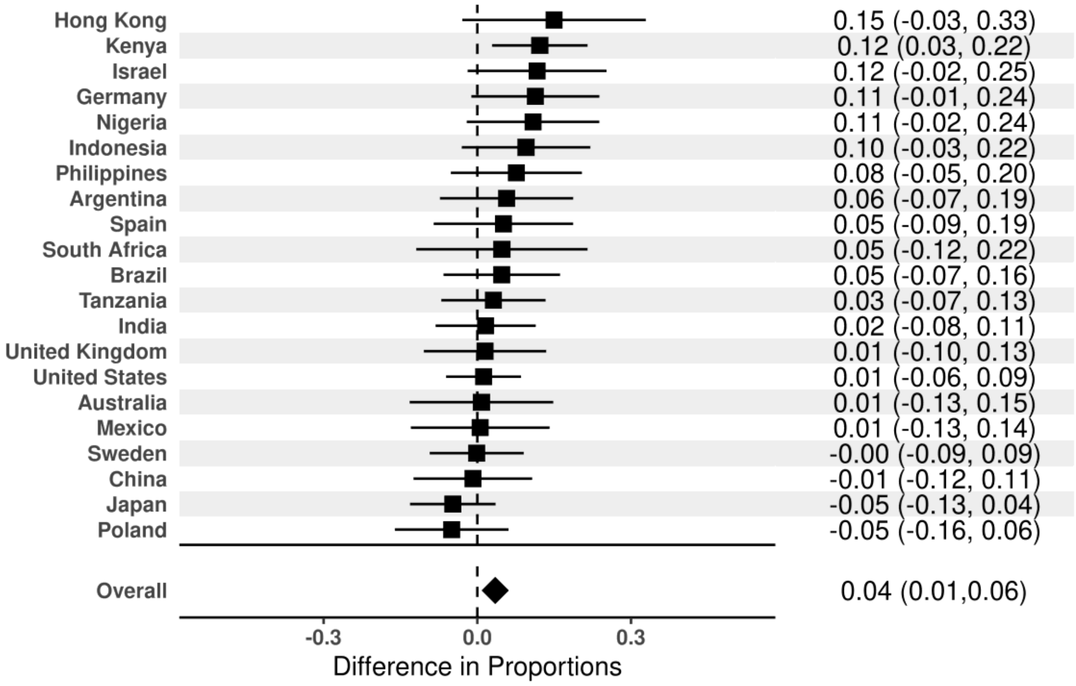

τ=0.000; Q(df=20)=18.74, p0.539;Q-profile 95% CI [0.000, 0.055]; I^2=0.00;
Excluded countries: Egypt, Turkiye

Figure S78. Forest plot for `Marital status`-`(Ref: Married) Separated`

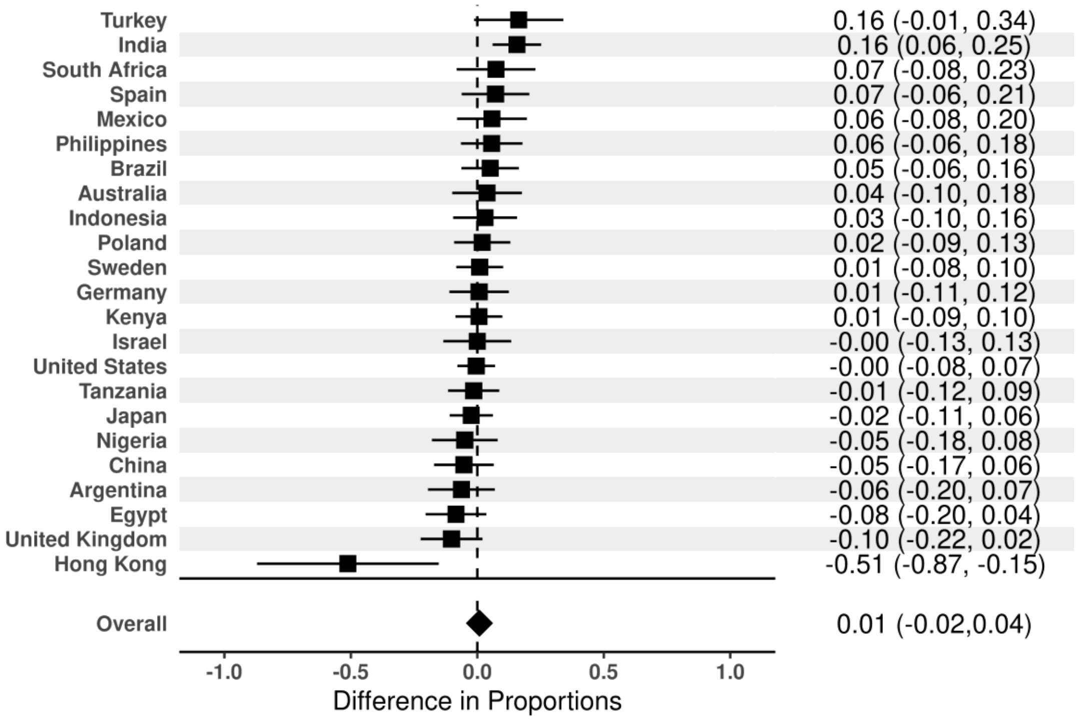

τ=0.050; Q(df=22)=32.94, p0.063;Q-profile 95% CI [0.000, 0.075]; I^2=42.09;

Figure S79. Forest plot for `Marital status`-`(Ref: Married) Widowed`

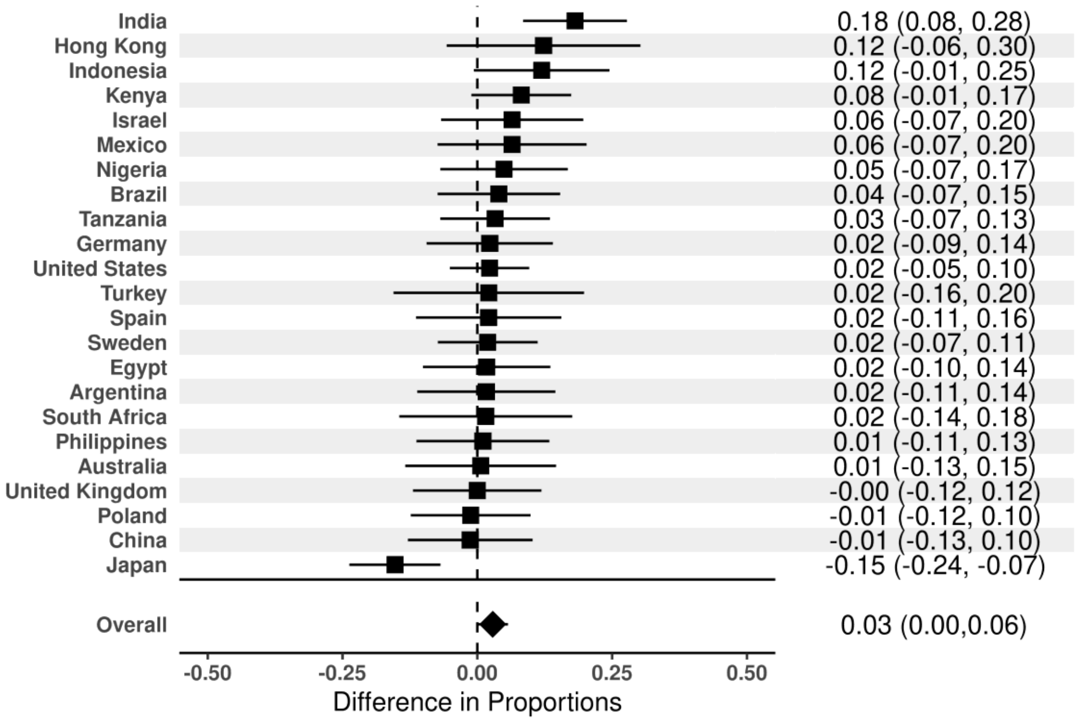

τ=0.036; Q(df=22)=33.92, p0.050;Q-profile 95% CI [0.012, 0.080]; I^2=27.26;

Figure S80. Forest plot for `Marital status`-`(Ref: Separated) Widowed`

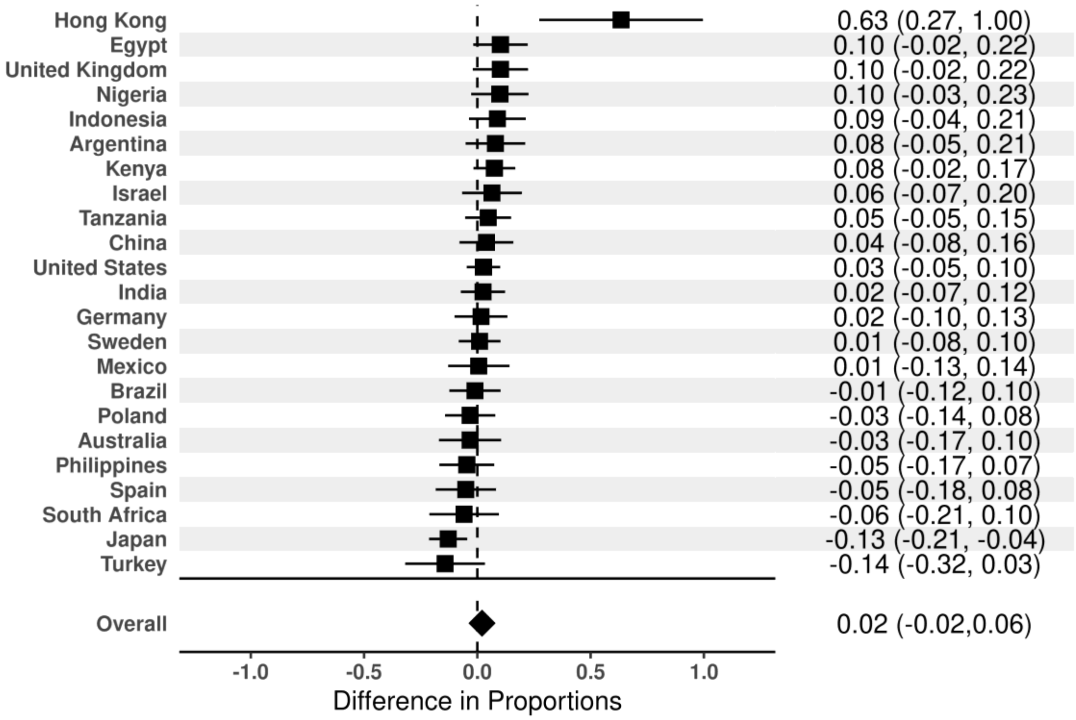

τ=0.067; Q(df=22)=40.53, p0.009;Q-profile 95% CI [0.000, 0.091]; I^2=56.66;

Figure S81. Forest plot for `Employment status`-`(Ref: Employed for an employer) Homemaker`

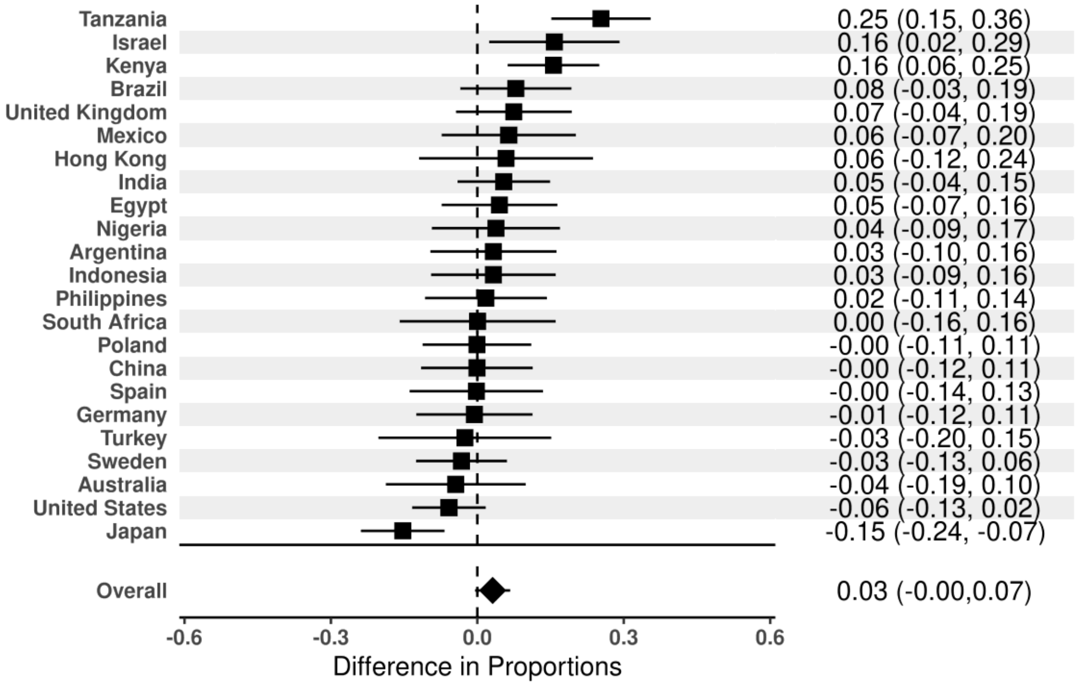

τ=0.063; Q(df=22)=58.00, p<.001;Q-profile 95% CI [0.041, 0.107]; I^2=53.71;

Figure S82. Forest plot for `Employment status`-`(Ref: Employed for an employer) None of these/other`

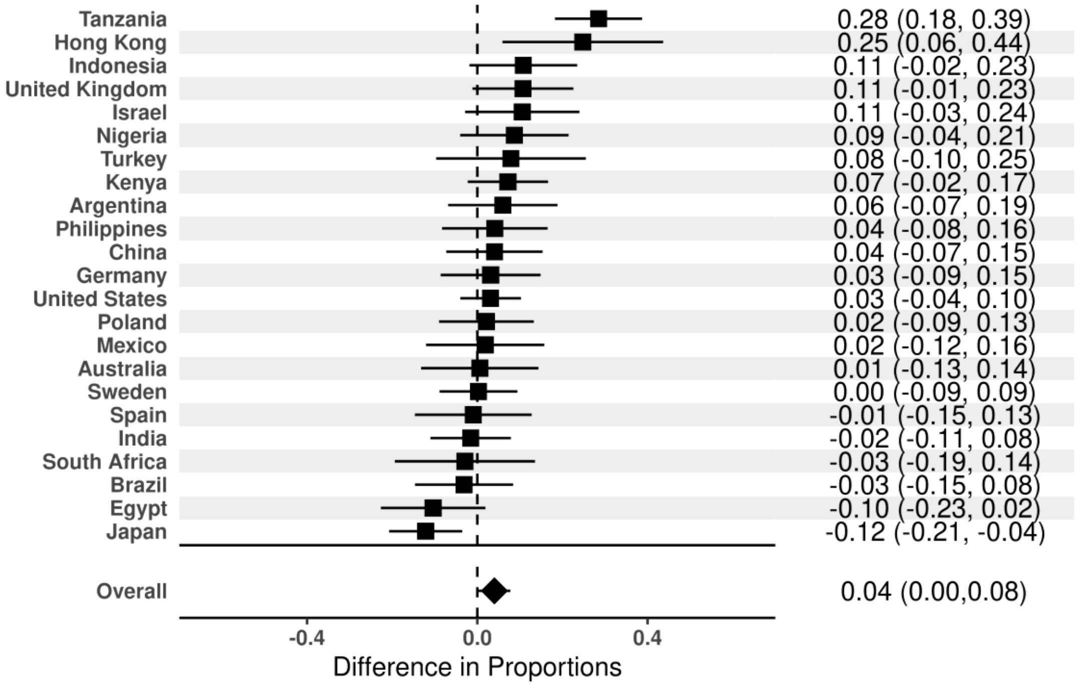

τ=0.068; Q(df=22)=54.93, p<.001;Q-profile 95% CI [0.038, 0.108]; I^2=57.33;

Figure S83. Forest plot for `Employment status`-`(Ref: Employed for an employer) Retired`

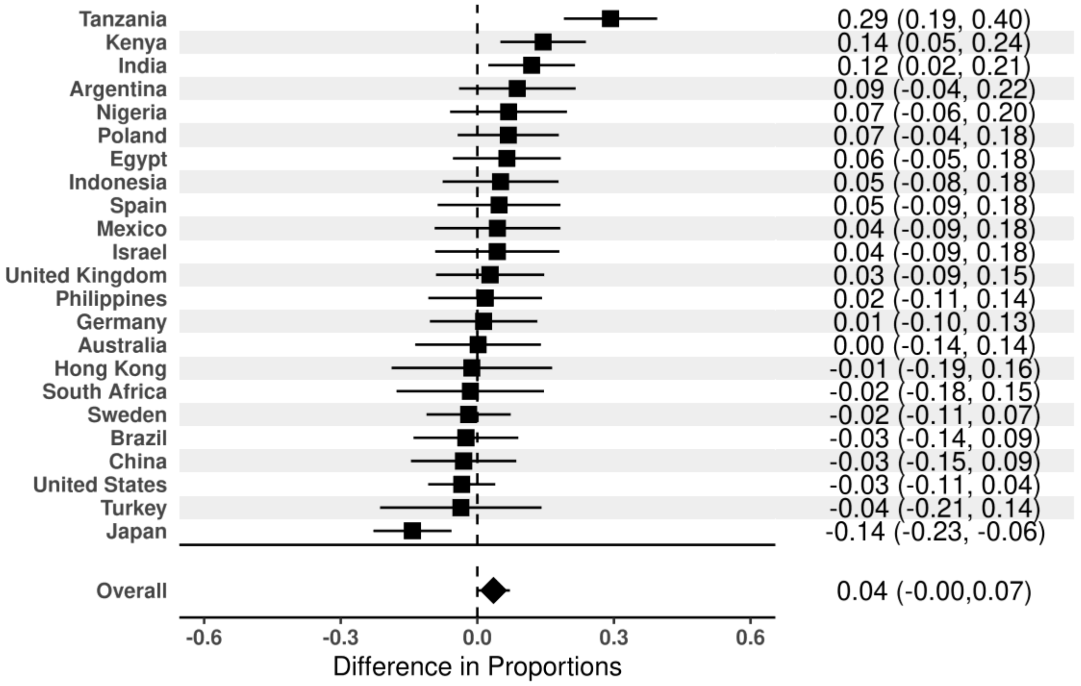

τ=0.064; Q(df=22)=59.22, p<.001;Q-profile 95% CI [0.042, 0.109]; I^2=54.79;

Figure S84. Forest plot for `Employment status`-`(Ref: Employed for an employer) Self-employed`

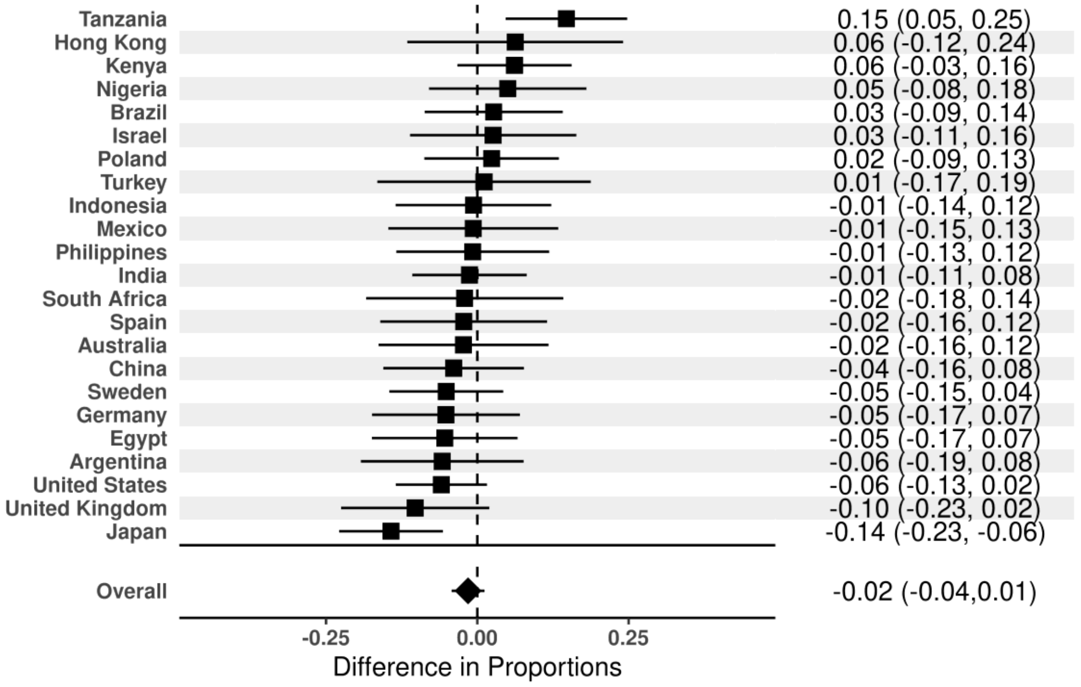

τ=0.029; Q(df=22)=29.40, p0.134;Q-profile 95% CI [0.000, 0.075]; I^2=19.59;

Figure S85. Forest plot for `Employment status`-`(Ref: Employed for an employer) Student`

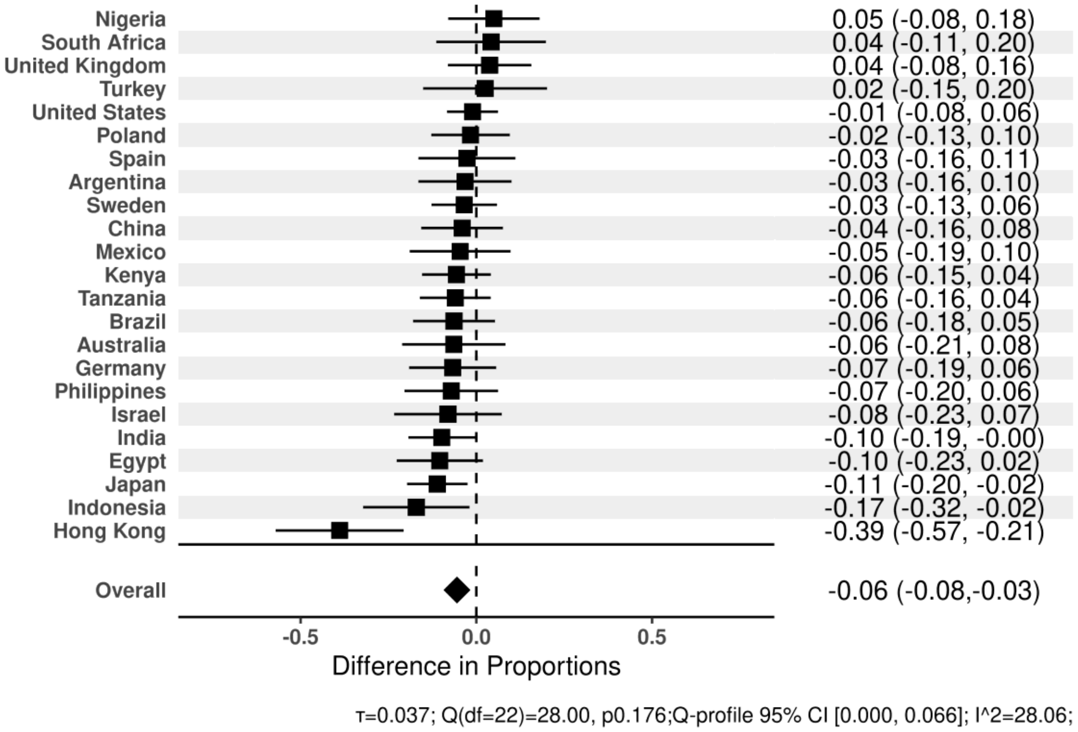

Figure S86. Forest plot for `Employment status`-`(Ref: Employed for an employer) Unemployed and looking for a job`

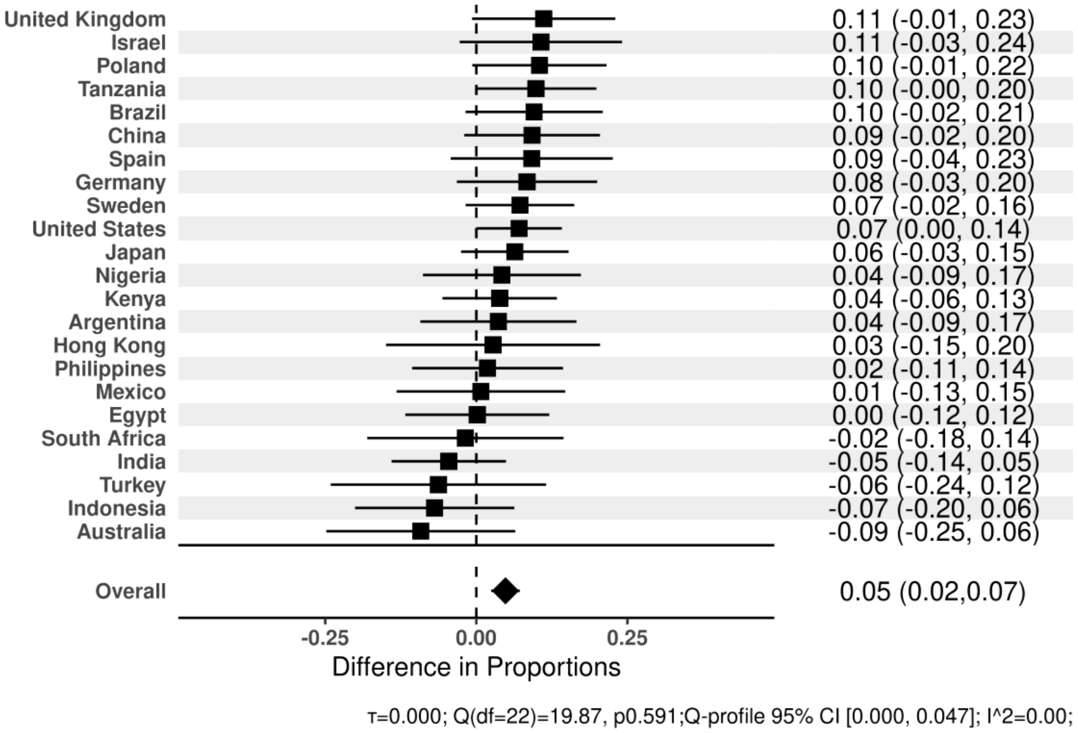

Figure S87. Forest plot for `Employment status`-`(Ref: Homemaker) None of these/other`

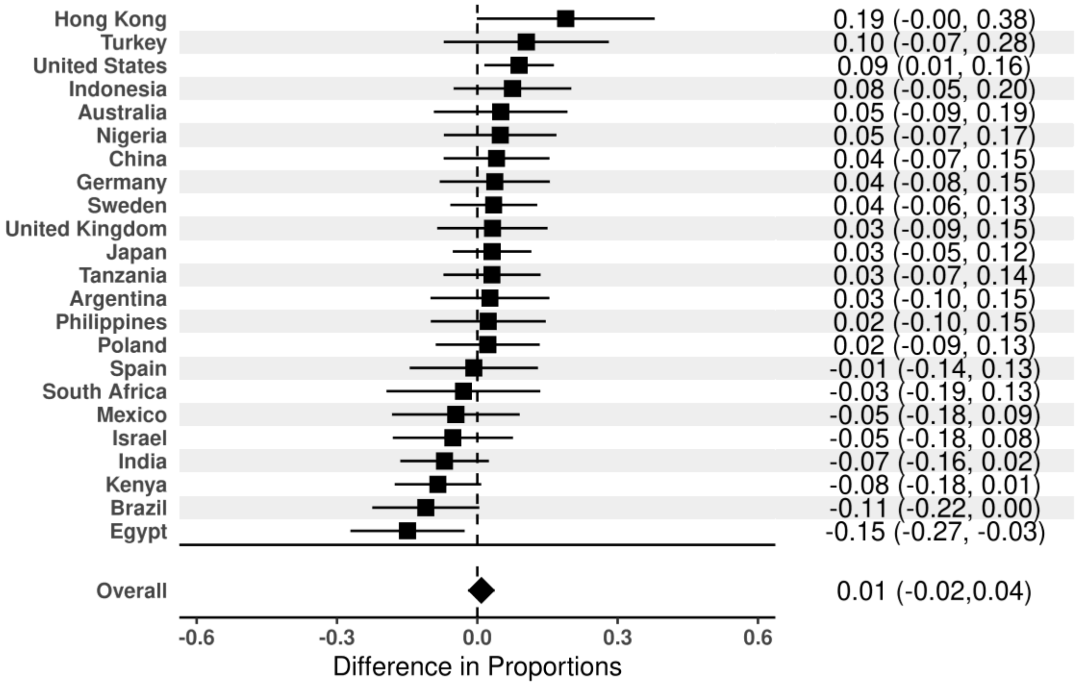

τ=0.037; Q(df=22)=31.41, p0.088;Q-profile 95% CI [0.000, 0.073]; I^2=28.35;

Figure S88. Forest plot for `Employment status`-`(Ref: Homemaker) Retired`

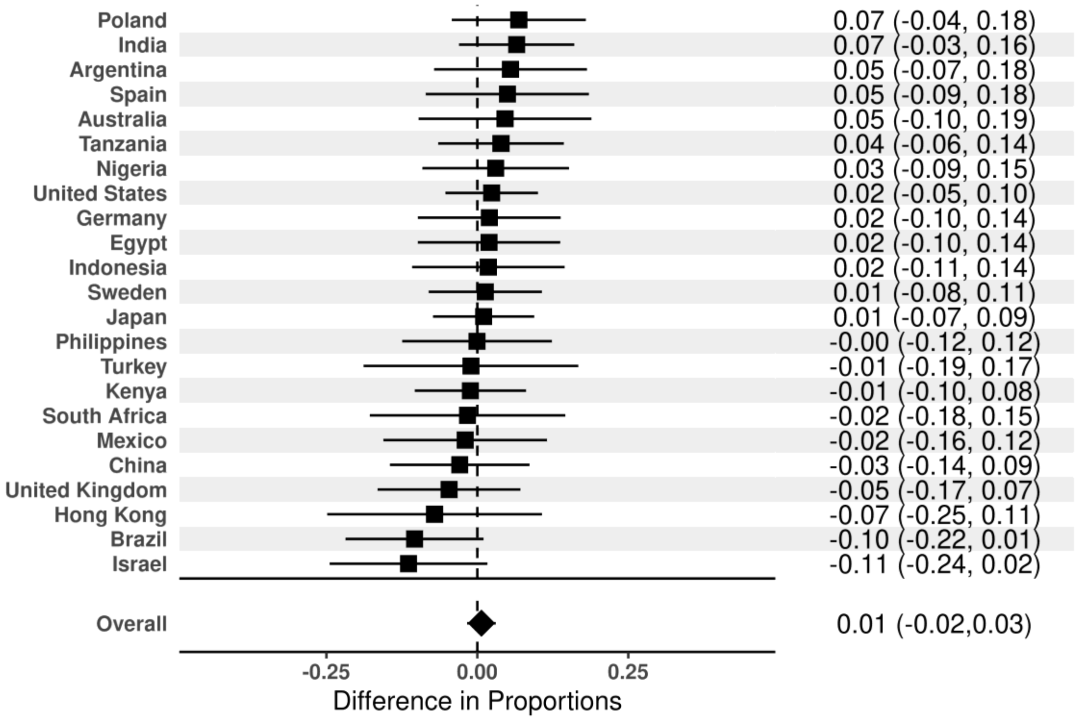

τ=0.000; Q(df=22)=13.93, p0.904;Q-profile 95% CI [0.000, 0.037]; I^2=0.00;

Figure S89. Forest plot for `Employment status`-`(Ref: Homemaker) Self-employed`

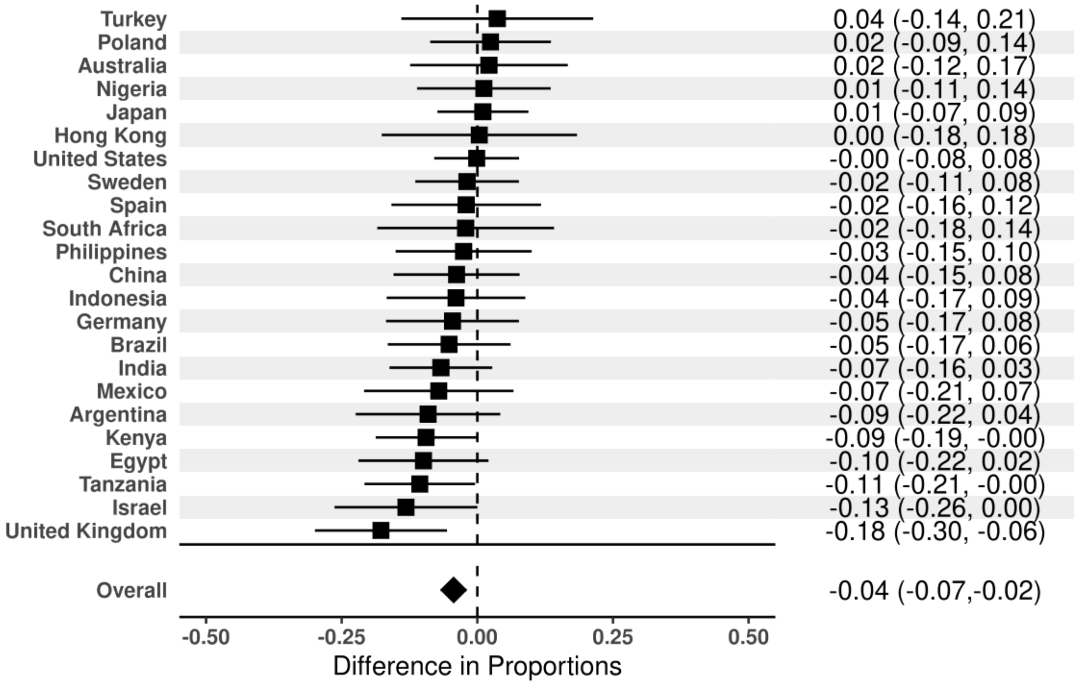

τ=0.000; Q(df=22)=17.99, p0.707;Q-profile 95% CI [0.000, 0.047]; I^2=0.00;

Figure S90. Forest plot for `Employment status`-`(Ref: Homemaker) Student`

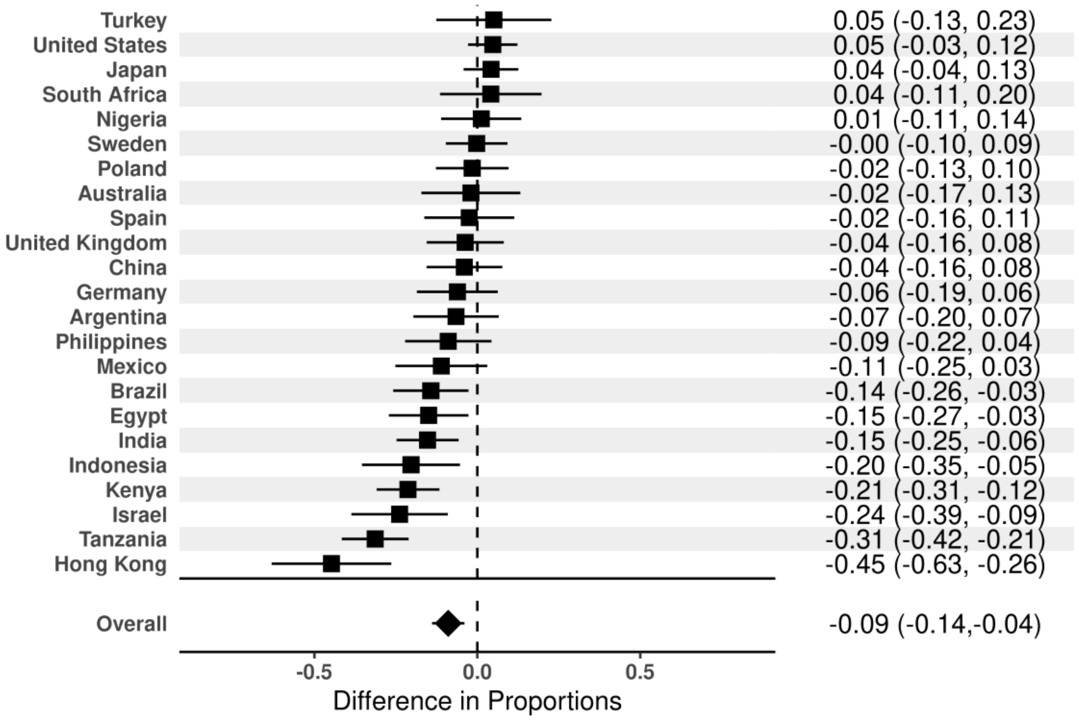

τ=0.104; Q(df=22)=86.18, p<.001;Q-profile 95% CI [0.062, 0.149]; I^2=75.05;

Figure S91. Forest plot for `Employment status`-`(Ref: Homemaker) Unemployed and looking for a job`

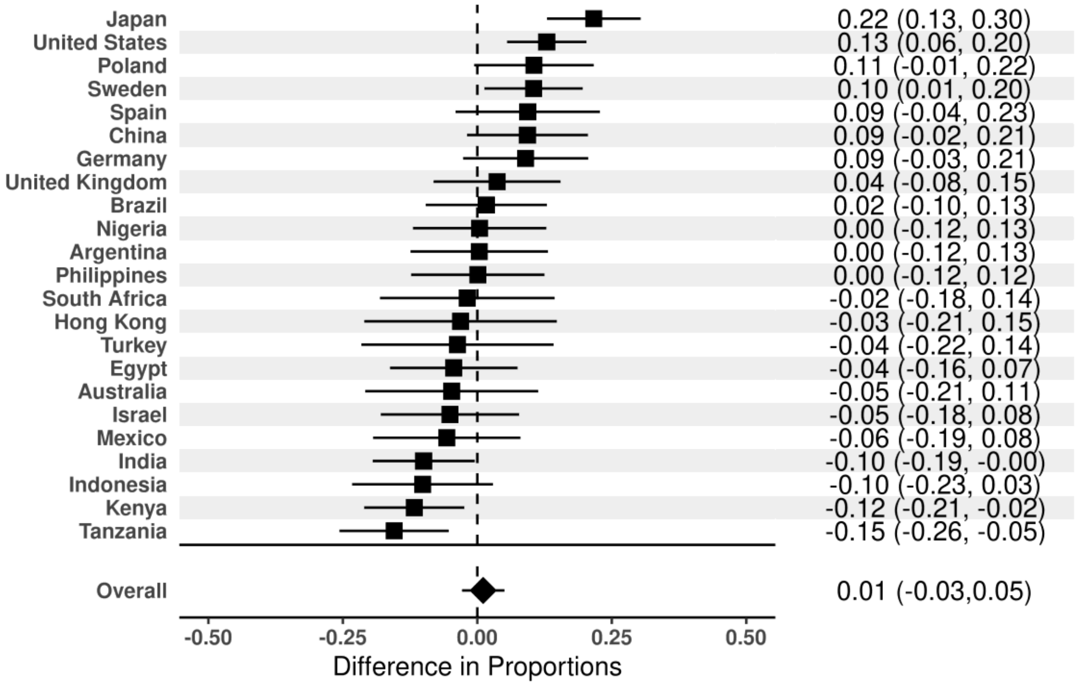

τ=0.075; Q(df=22)=72.25, p<.001;Q-profile 95% CI [0.051, 0.120]; I^2=62.03;

Figure S92. Forest plot for `Employment status`-`(Ref: None of these/other) Retired`

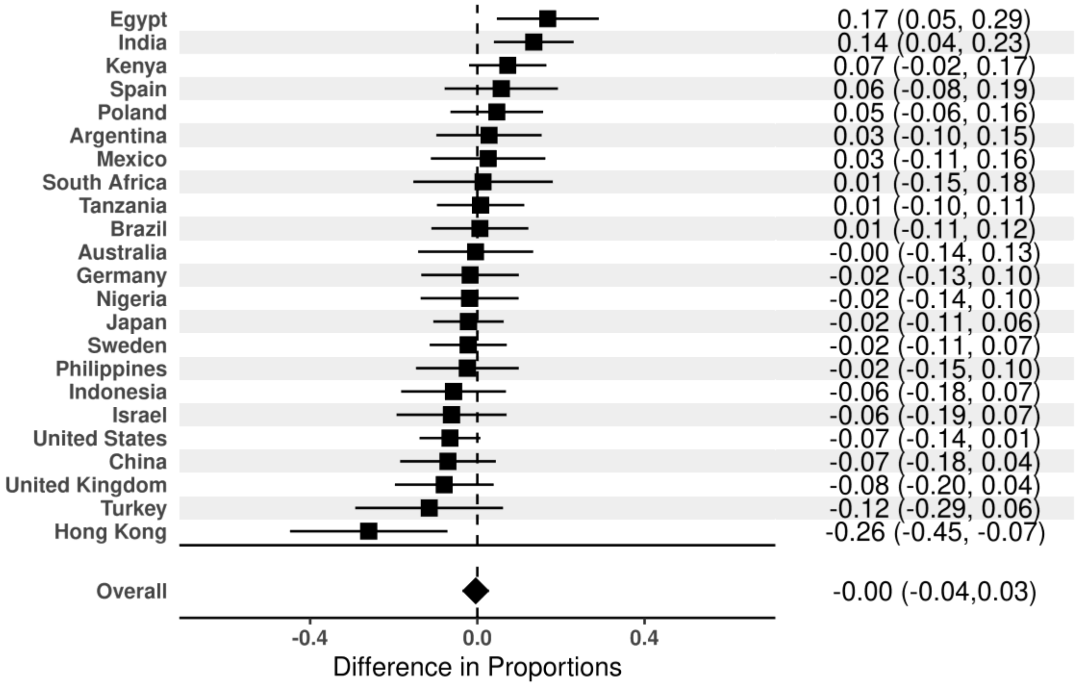

τ=0.049; Q(df=22)=36.75, p0.025;Q-profile 95% CI [0.000, 0.083]; I^2=41.82;

Figure S93. Forest plot for `Employment status`-`(Ref: None of these/other) Self-employed`

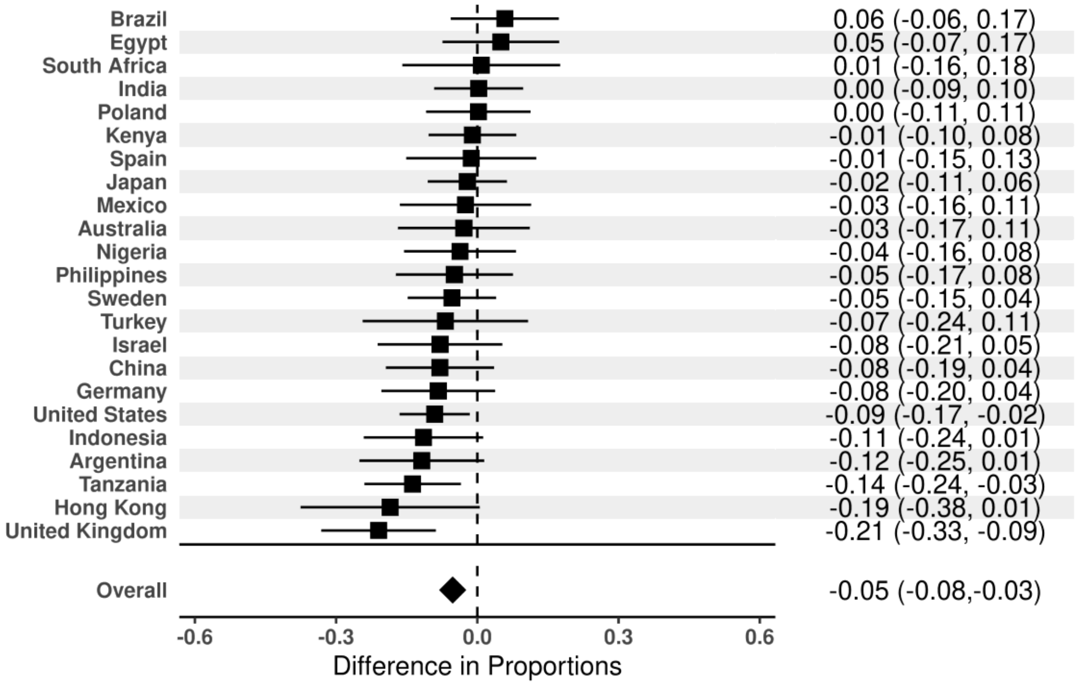

τ=0.023; Q(df=22)=25.33, p0.282;Q-profile 95% CI [0.000, 0.062]; I^2=13.09;

Figure S94. Forest plot for `Employment status`-`(Ref: None of these/other) Student`

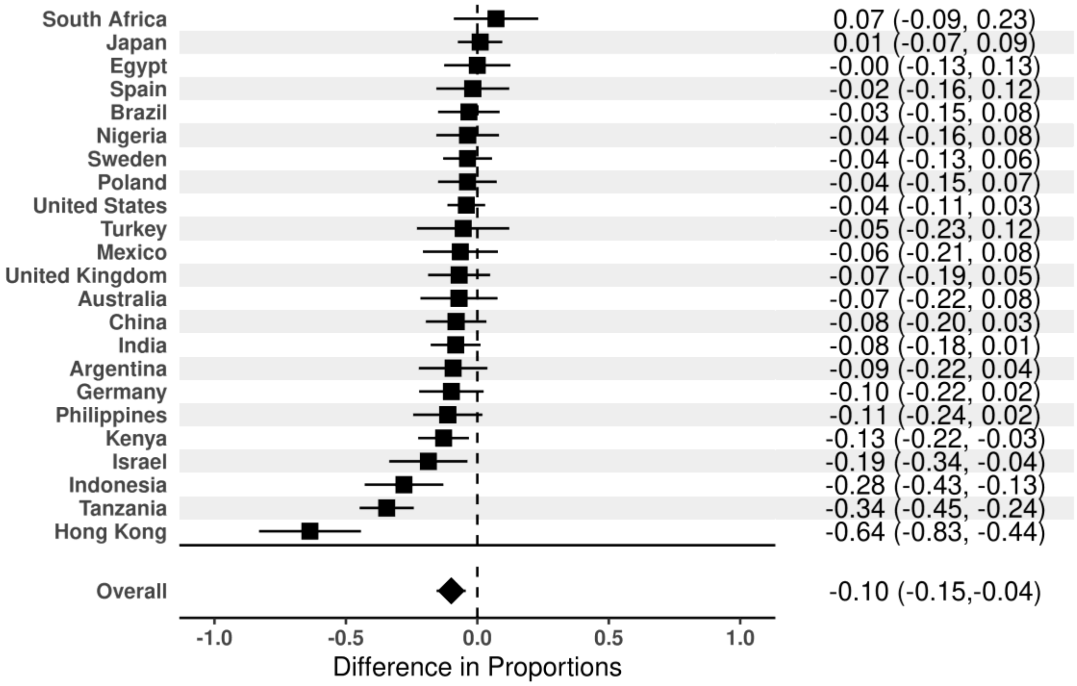

τ=0.119; Q(df=22)=80.74, p<.001;Q-profile 95% CI [0.060, 0.165]; I^2=80.17;

Figure S95. Forest plot for `Employment status`-`(Ref: None of these/other) Unemployed and looking for a job`

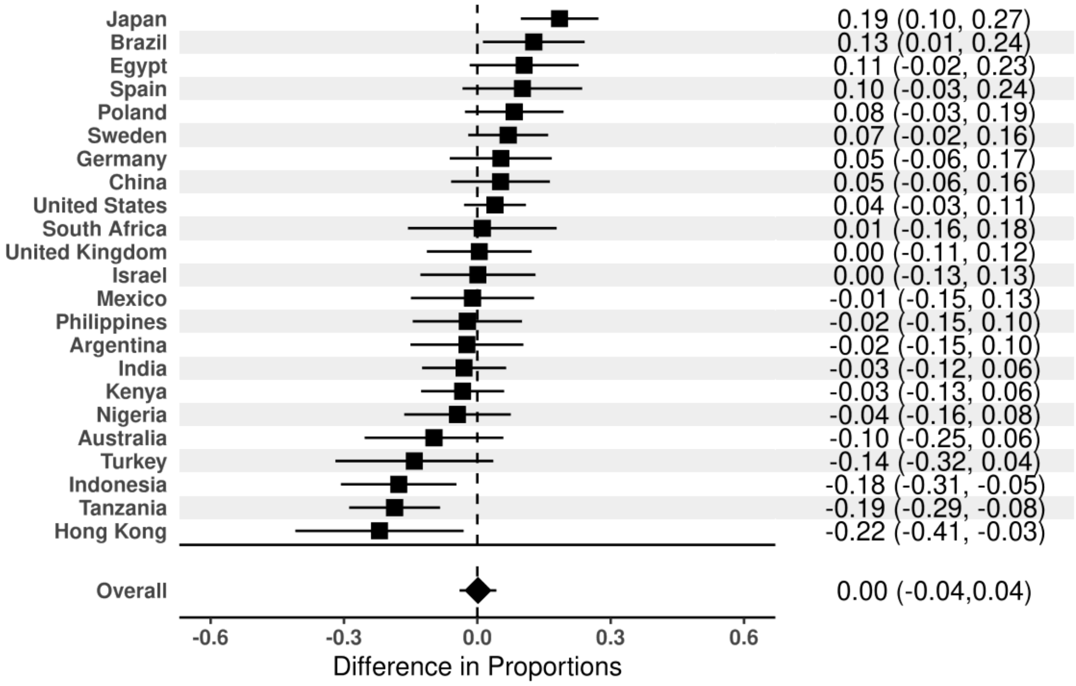

τ=0.081; Q(df=22)=63.93, p<.001;Q-profile 95% CI [0.046, 0.122]; I^2=65.90;

Figure S96. Forest plot for `Employment status`-`(Ref: Retired) Self-employed`

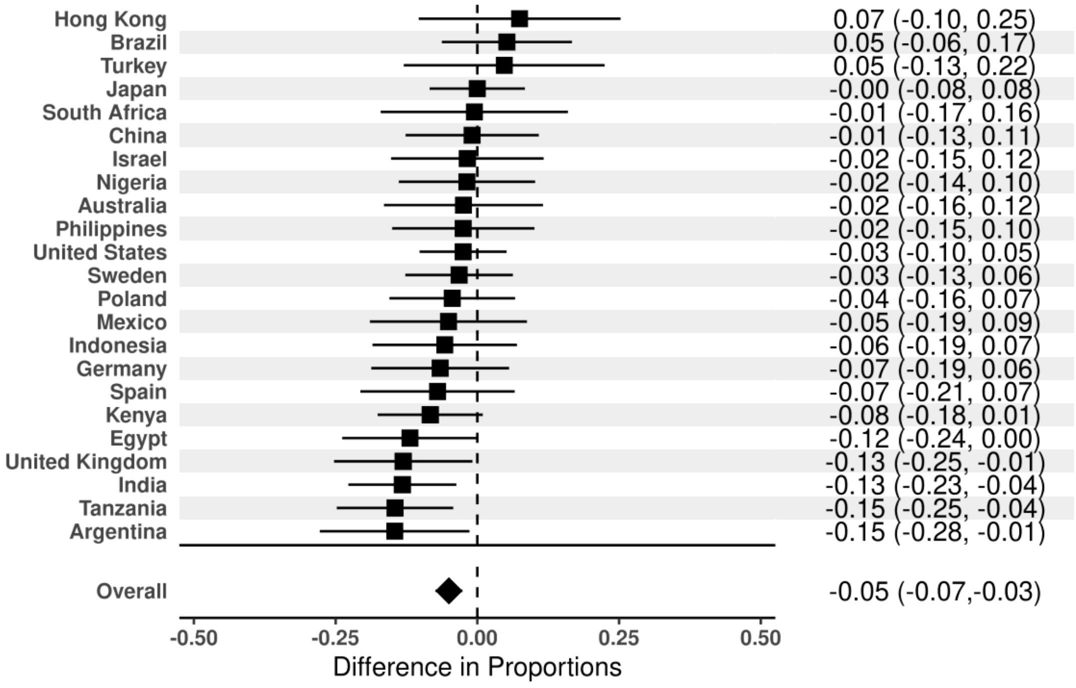

τ=0.000; Q(df=22)=21.31, p0.502;Q-profile 95% CI [0.000, 0.053]; I^2=0.00;

Figure S97. Forest plot for `Employment status`-`(Ref: Retired) Student`

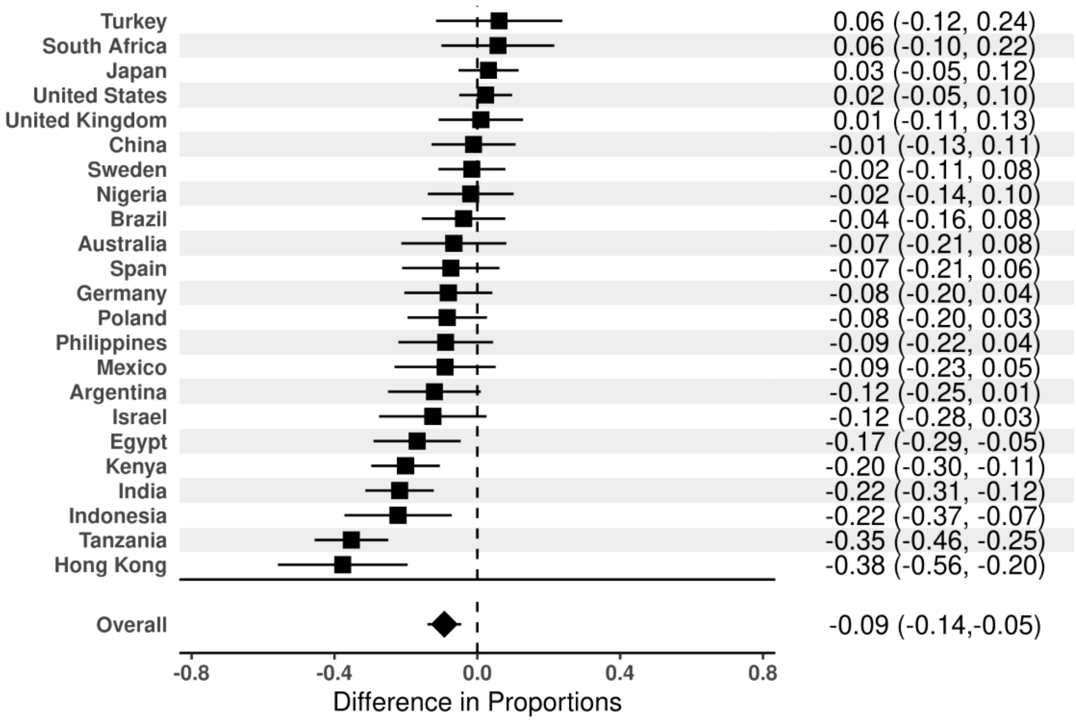

τ=0.097; Q(df=22)=83.62, p<.001;Q-profile 95% CI [0.060, 0.142]; I^2=72.56;

Figure S98. Forest plot for `Employment status`-`(Ref: Retired) Unemployed and looking for a job`

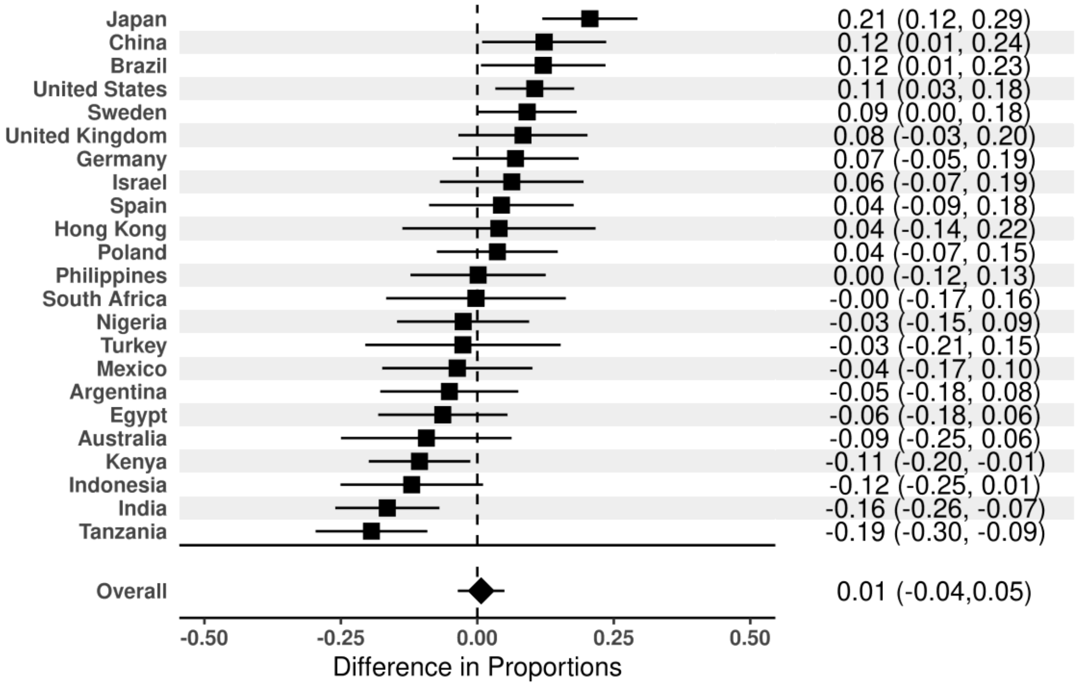

τ=0.084; Q(df=22)=83.12, p<.001;Q-profile 95% CI [0.058, 0.131]; I^2=67.77;

Figure S99. Forest plot for `Employment status`-`(Ref: Self-employed) Student`

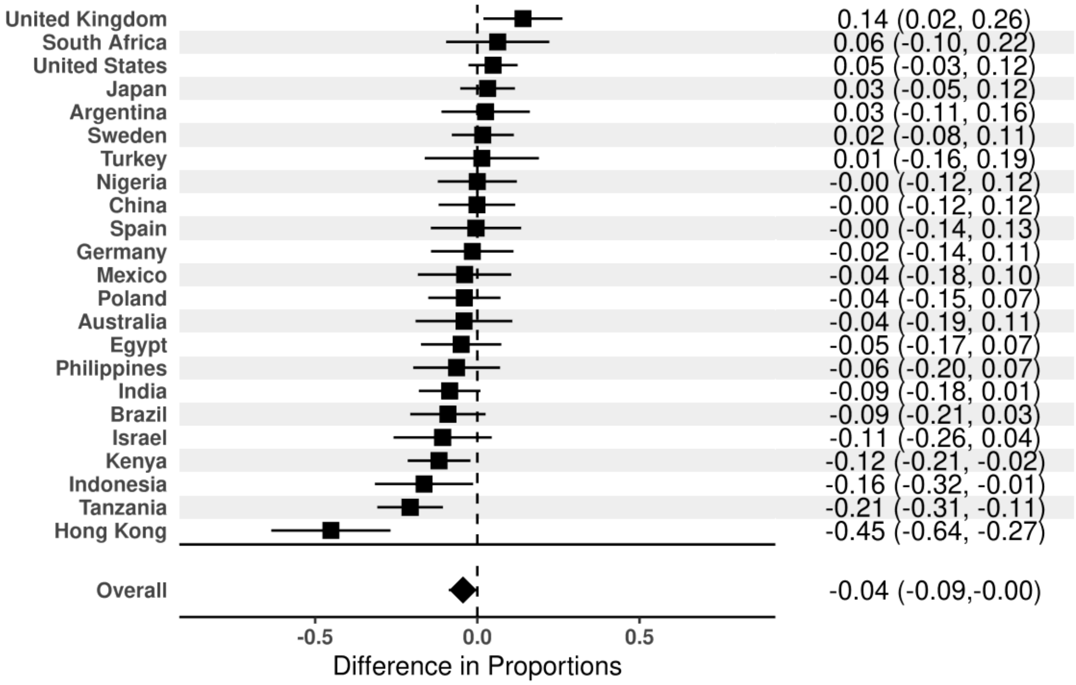

τ=0.087; Q(df=22)=59.18, p<.001;Q-profile 95% CI [0.038, 0.126]; I^2=67.50;

Figure S100. Forest plot for `Employment status`-`(Ref: Self-employed) Unemployed and looking for a job`

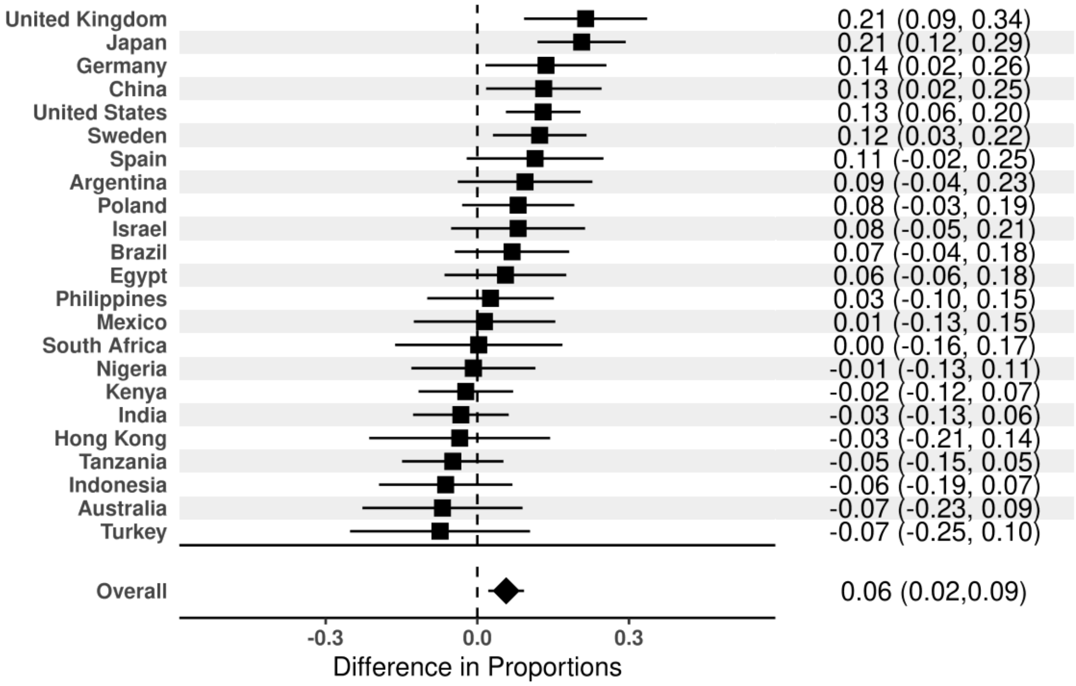

τ=0.061; Q(df=22)=48.93, p<.001;Q-profile 95% CI [0.032, 0.101]; I^2=51.84;

Figure S101. Forest plot for `Employment status`-`(Ref: Student) Unemployed and looking for a job`

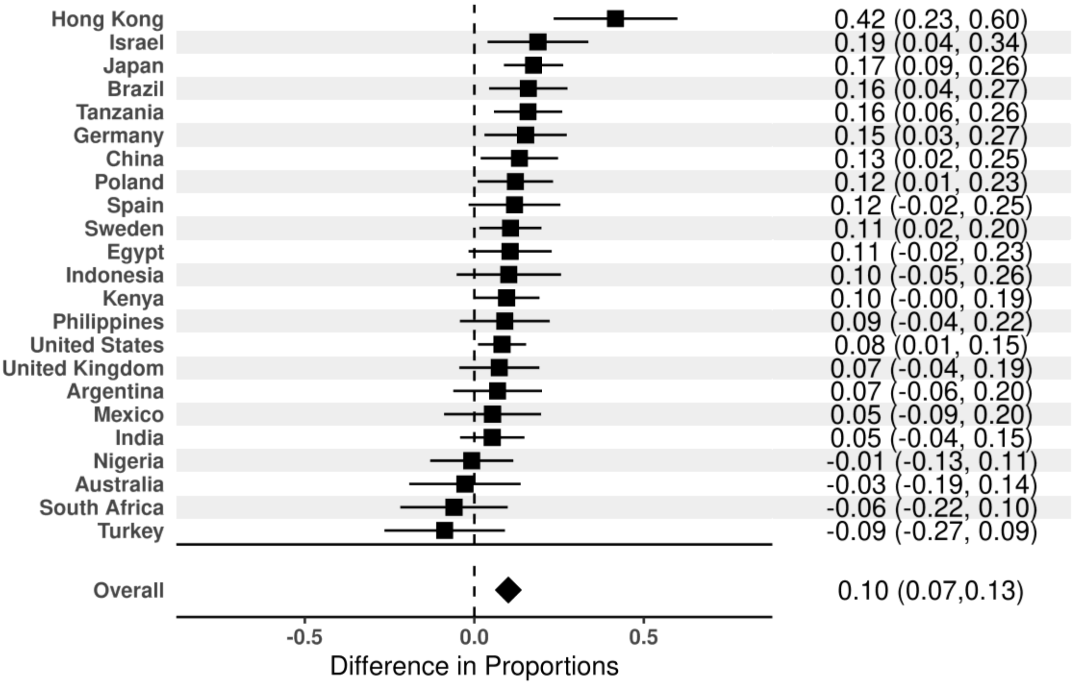

τ=0.057; Q(df=22)=34.69, p0.042;Q-profile 95% CI [0.000, 0.086]; I^2=47.67;

Figure S102. Forest plot for `Religious service attendance`-`(Ref: Never) A few times a year`

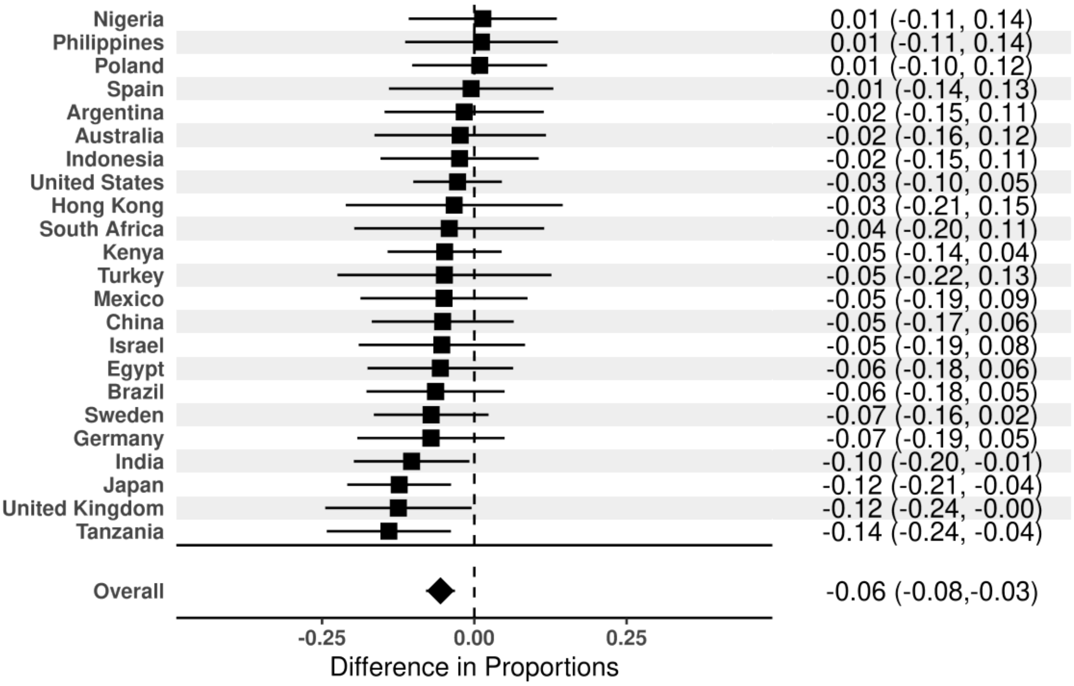

τ=0.000; Q(df=22)=13.15, p0.929;Q-profile 95% CI [0.000, 0.040]; I^2=0.00;

Figure S103. Forest plot for `Religious service attendance`-`(Ref: Never) >1/week`

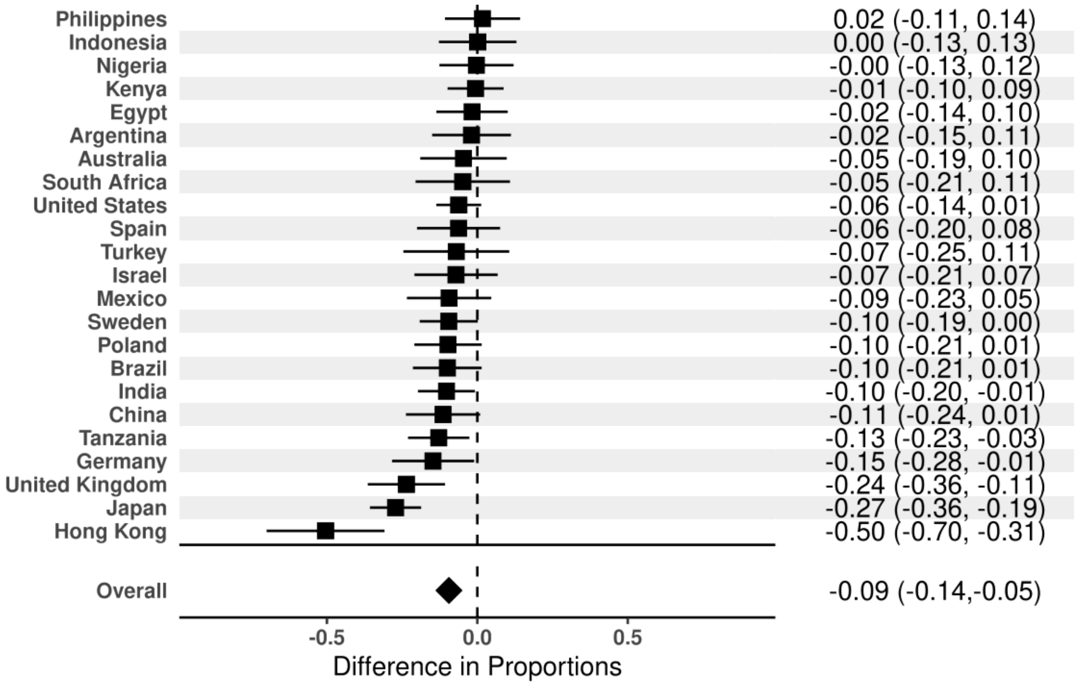

τ=0.079; Q(df=22)=54.91, p<.001;Q-profile 95% CI [0.031, 0.116]; I^2=64.07;

Figure S104. Forest plot for `Religious service attendance`-`(Ref: Never) 1/week`

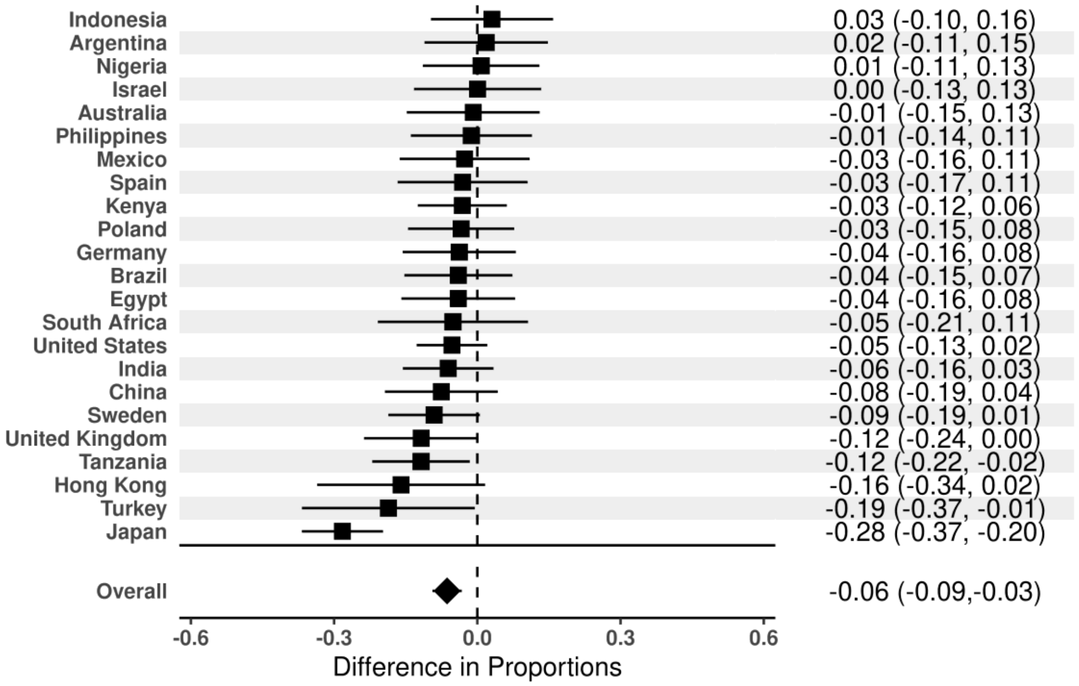

τ=0.045; Q(df=22)=39.42, p0.013;Q-profile 95% CI [0.020, 0.086]; I^2=37.10;

Figure S105. Forest plot for `Religious service attendance`-`(Ref: Never) 1-3/month`

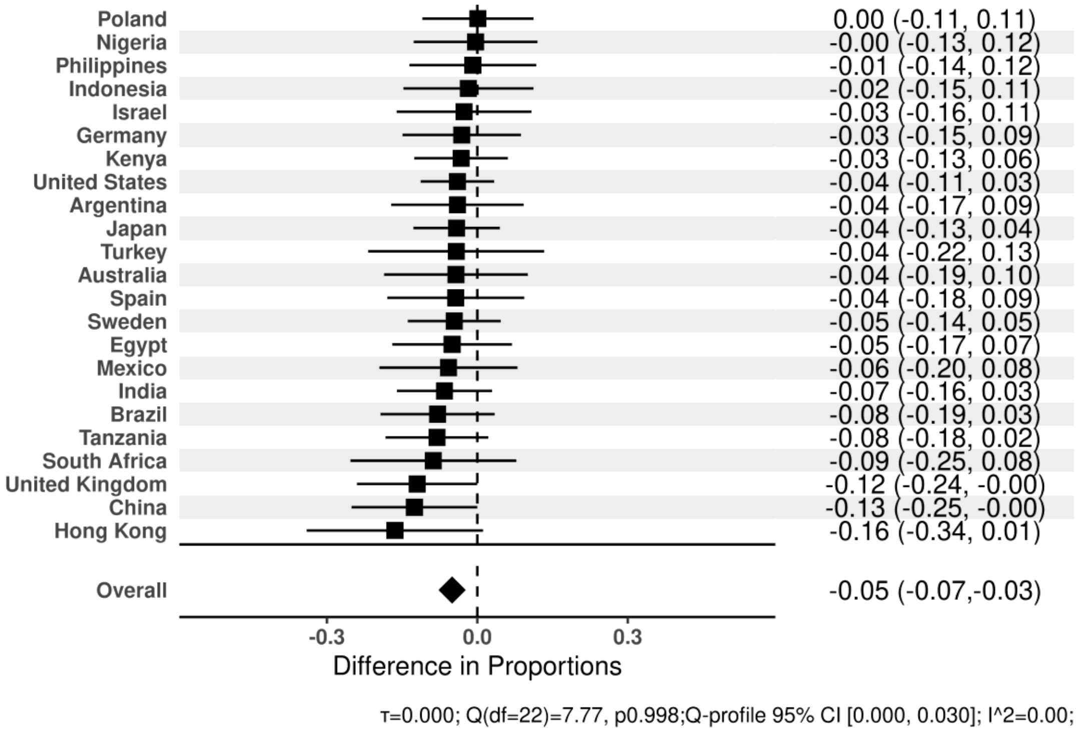

Figure S106. Forest plot for `Religious service attendance`-`(Ref: A few times a year) >1/week`

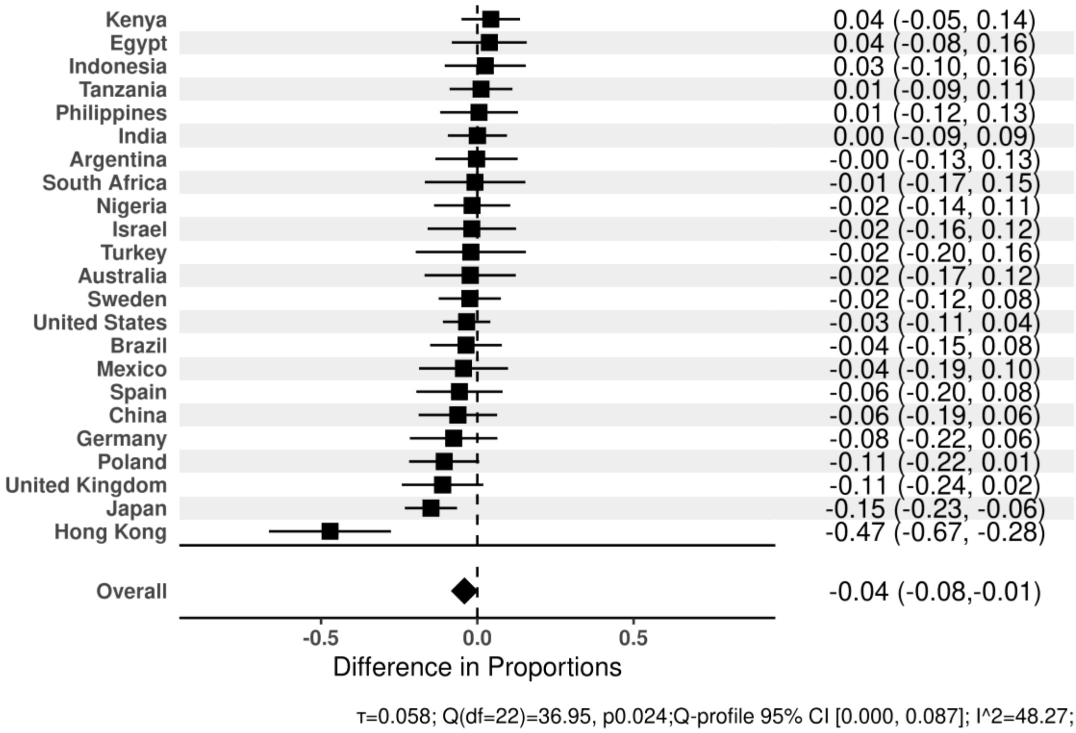

Figure S107. Forest plot for `Religious service attendance`-`(Ref: A few times a year) 1/week`

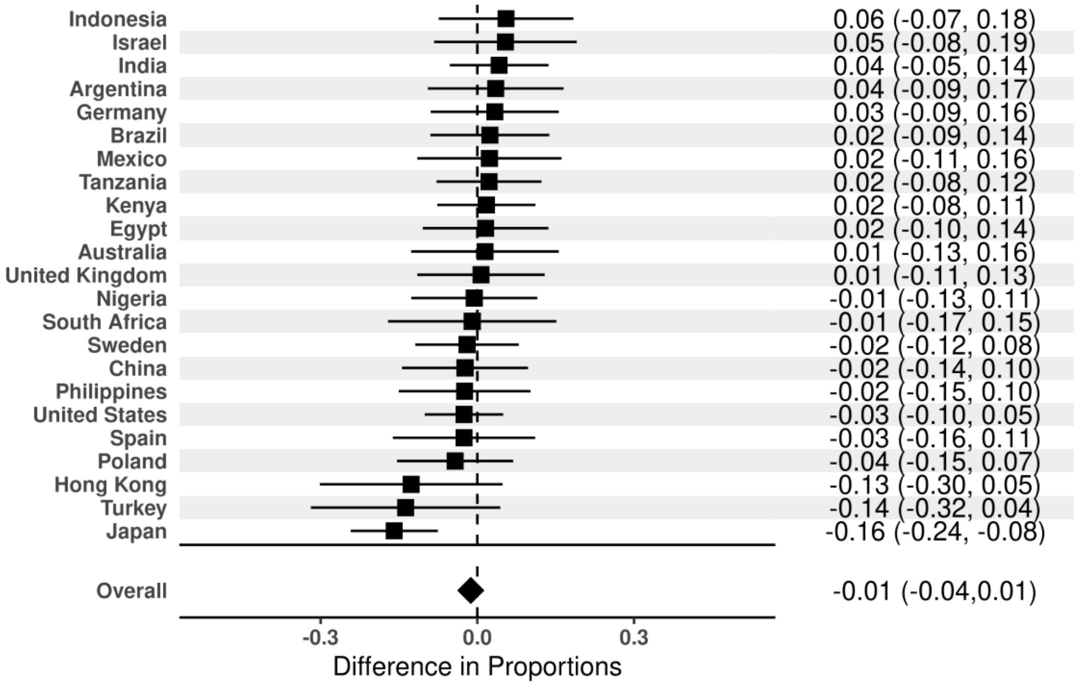

τ=0.000; Q(df=22)=22.05, p0.457;Q-profile 95% CI [0.000, 0.062]; I^2=0.00;

Figure S108. Forest plot for `Religious service attendance`-`(Ref: A few times a year) 1-3/month`

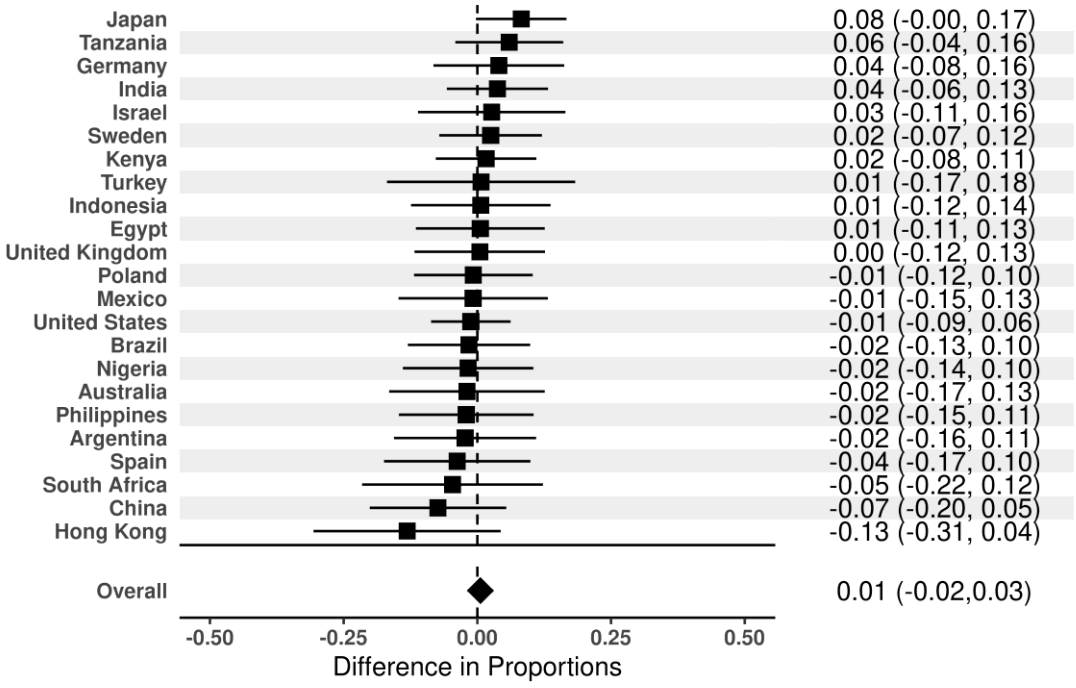

τ=0.000; Q(df=22)=10.90, p0.976;Q-profile 95% CI [0.000, 0.035]; I^2=0.00;

Figure S109. Forest plot for `Religious service attendance`-`(Ref: >1/week) 1/week`

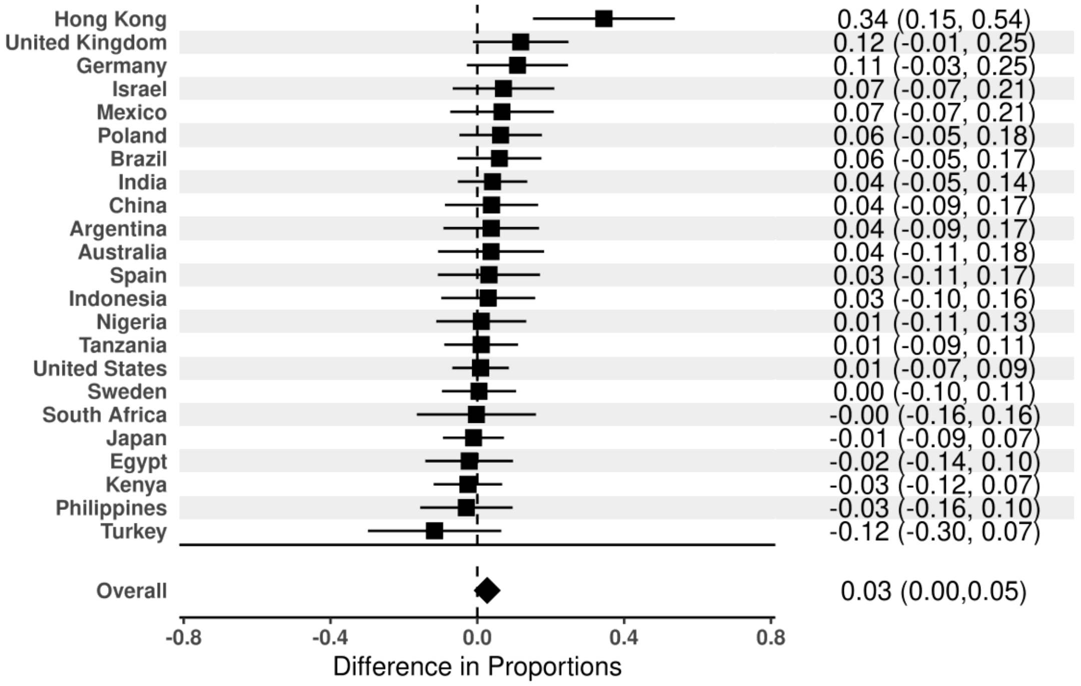

τ=0.000; Q(df=22)=21.80, p0.472;Q-profile 95% CI [0.000, 0.042]; I^2=0.00;

Figure S110. Forest plot for `Religious service attendance`-`(Ref: >1/week) 1-3/month`

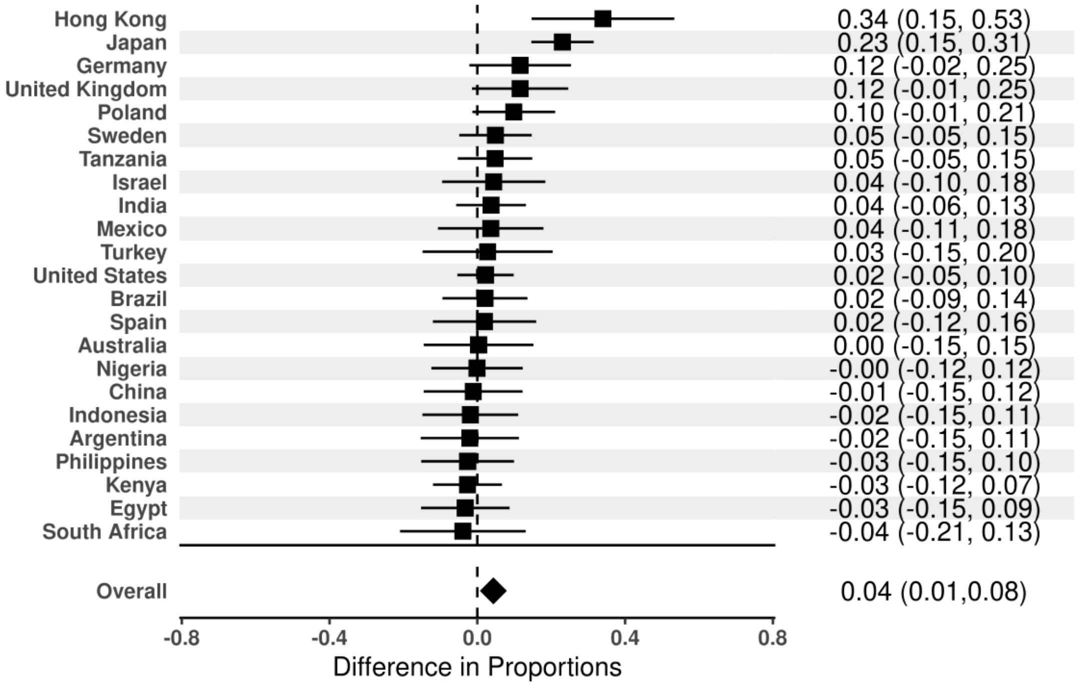

τ=0.053; Q(df=22)=40.74, p0.009;Q-profile 95% CI [0.019, 0.090]; I^2=43.27;

Figure S111. Forest plot for `Religious service attendance`-`(Ref: 1/week) 1-3/month`

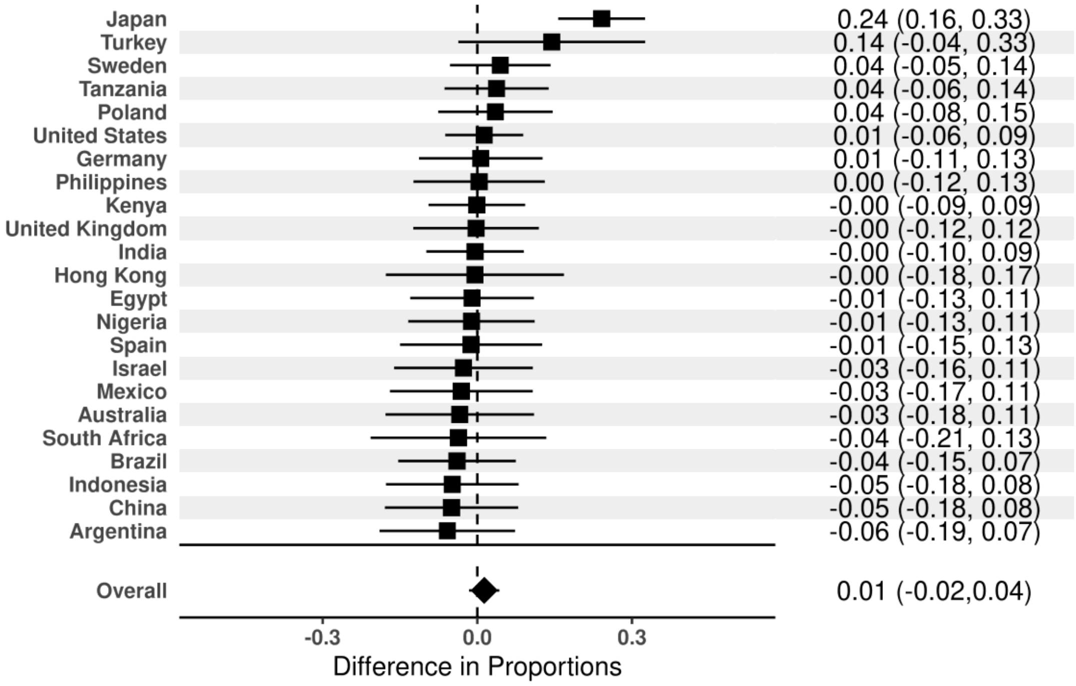

τ=0.039; Q(df=22)=36.68, p0.026;Q-profile 95% CI [0.018, 0.085]; I^2=29.97;

Figure S112. Forest plot for `Education`-`(Ref: 9-15 years) 16+ years`

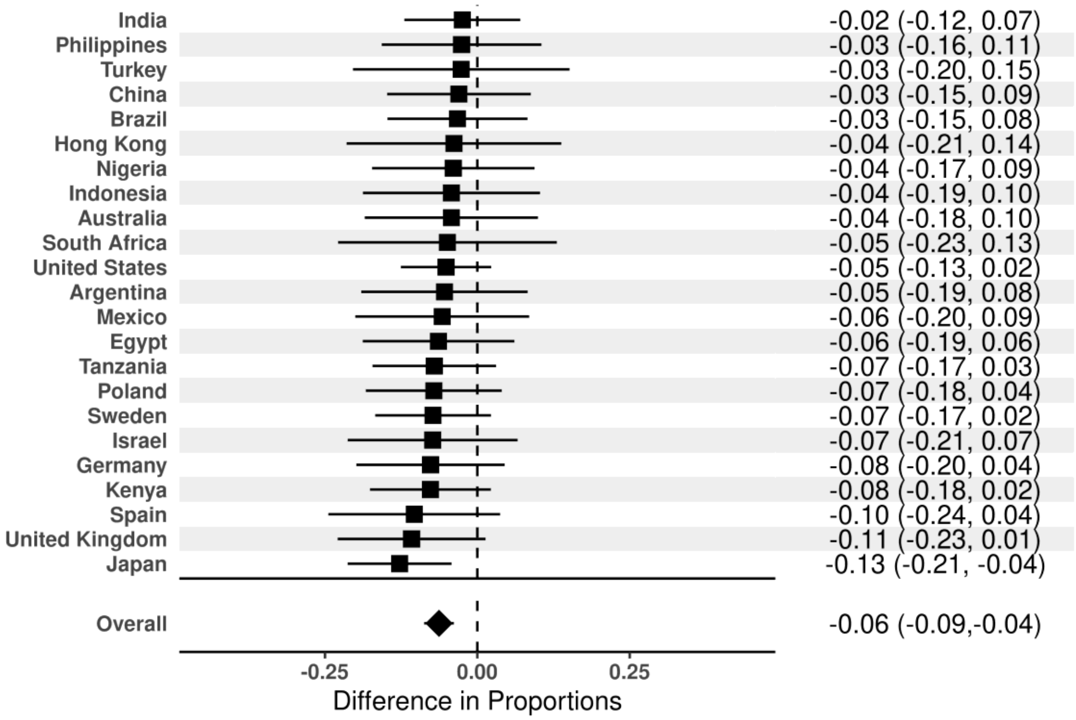

τ=0.000; Q(df=22)=5.44, p1.000;Q-profile 95% CI [0.000, 0.031]; I^2=0.00;

Figure S113. Forest plot for `Education`-`(Ref: 9-15 years) Up to 8 years`

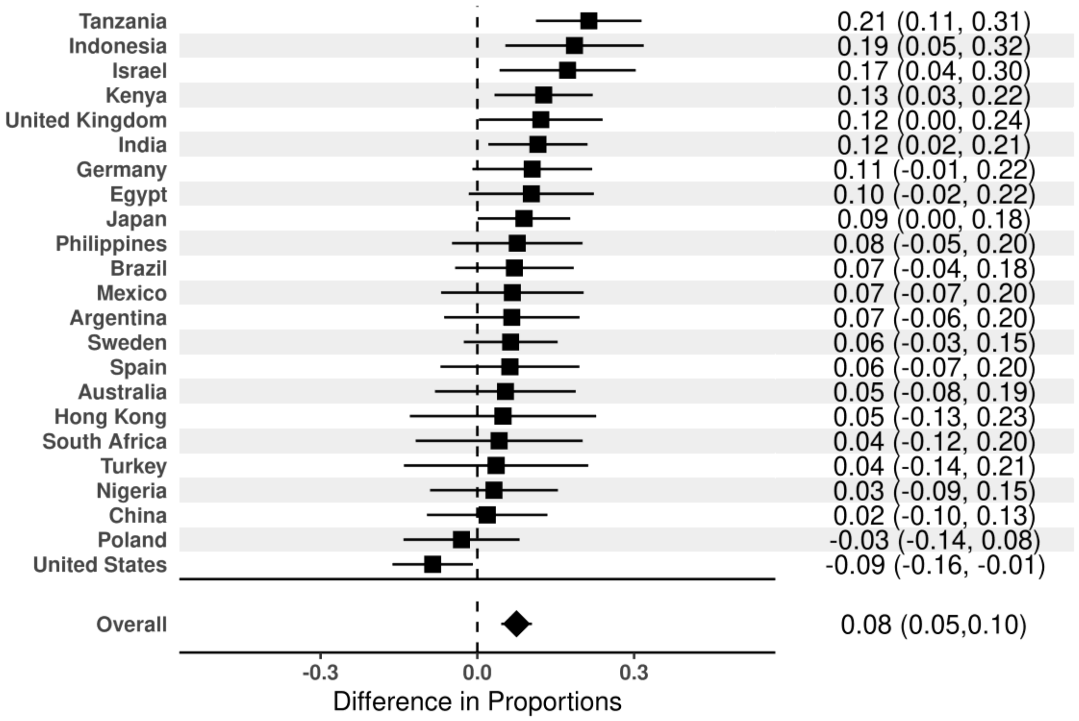

τ=0.040; Q(df=22)=37.26, p0.022;Q-profile 95% CI [0.018, 0.084]; I^2=31.98;

Figure S114. Forest plot for `Education`-`(Ref: 16+ years) Up to 8 years`

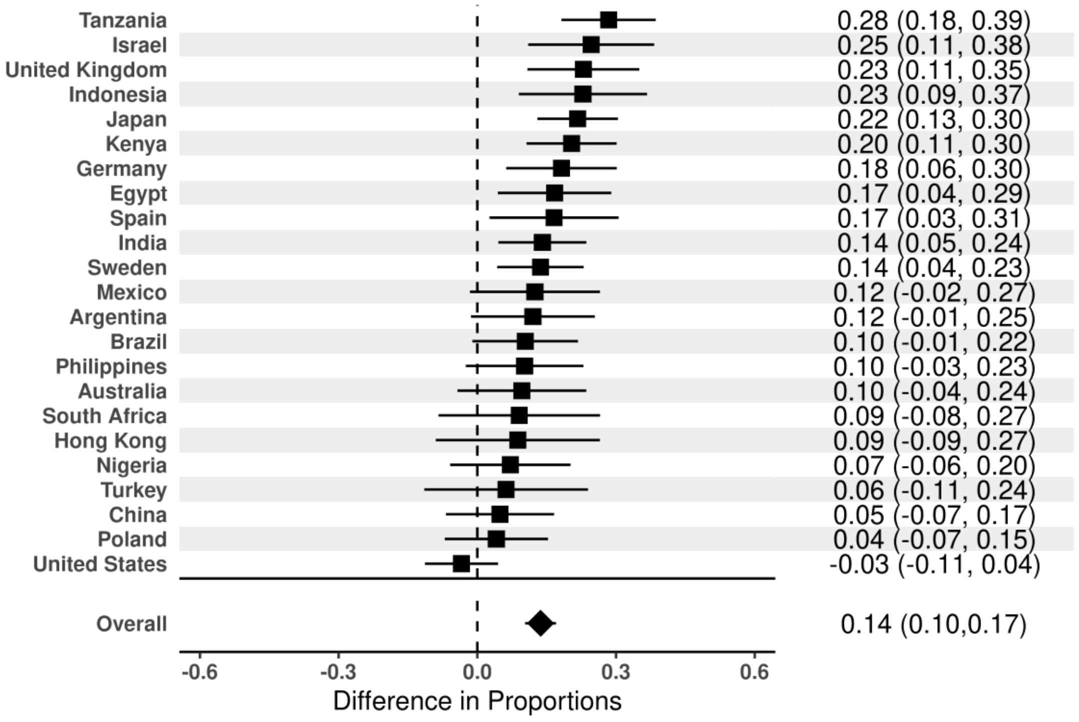

τ=0.053; Q(df=22)=46.51, p0.002;Q-profile 95% CI [0.031, 0.097]; I^2=44.09;

Figure S115. Forest plot for `Immigration status`-`(Ref: Born in this country) Born in another country`

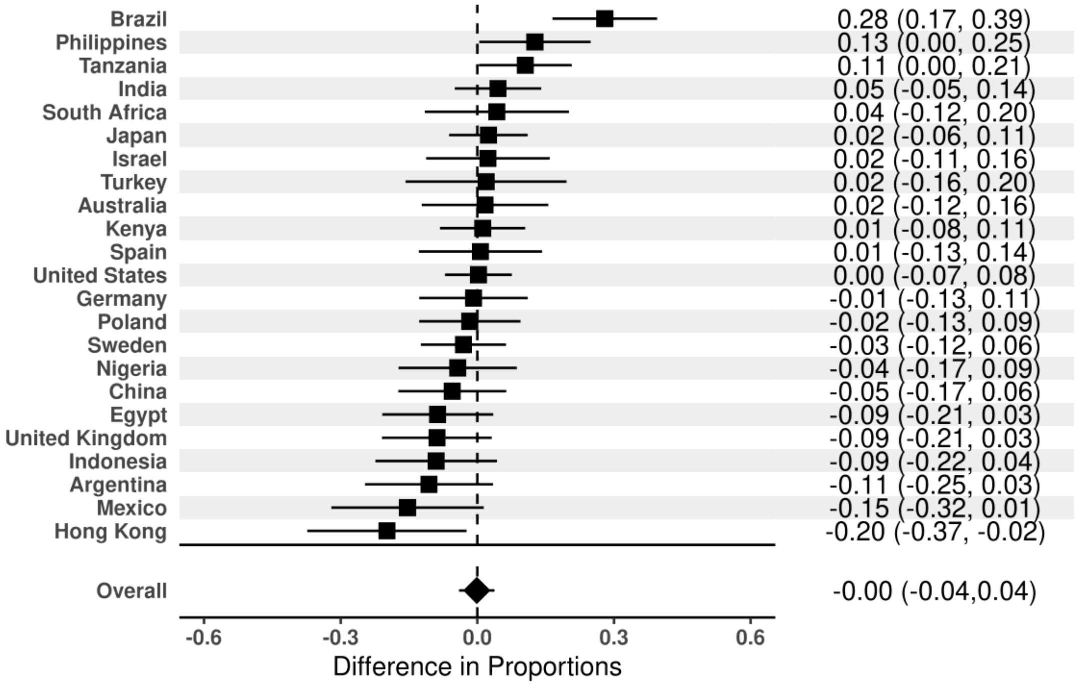

τ=0.072; Q(df=22)=50.55, p<.001;Q-profile 95% CI [0.031, 0.111]; I^2=59.97;

Forest Plots (Childhood)

Figure S1. Forest plot of the effects of Relationship with your mother growing up Very good/somewhat good (Ref: Very bad/somewhat bad) predicting experienced beauty

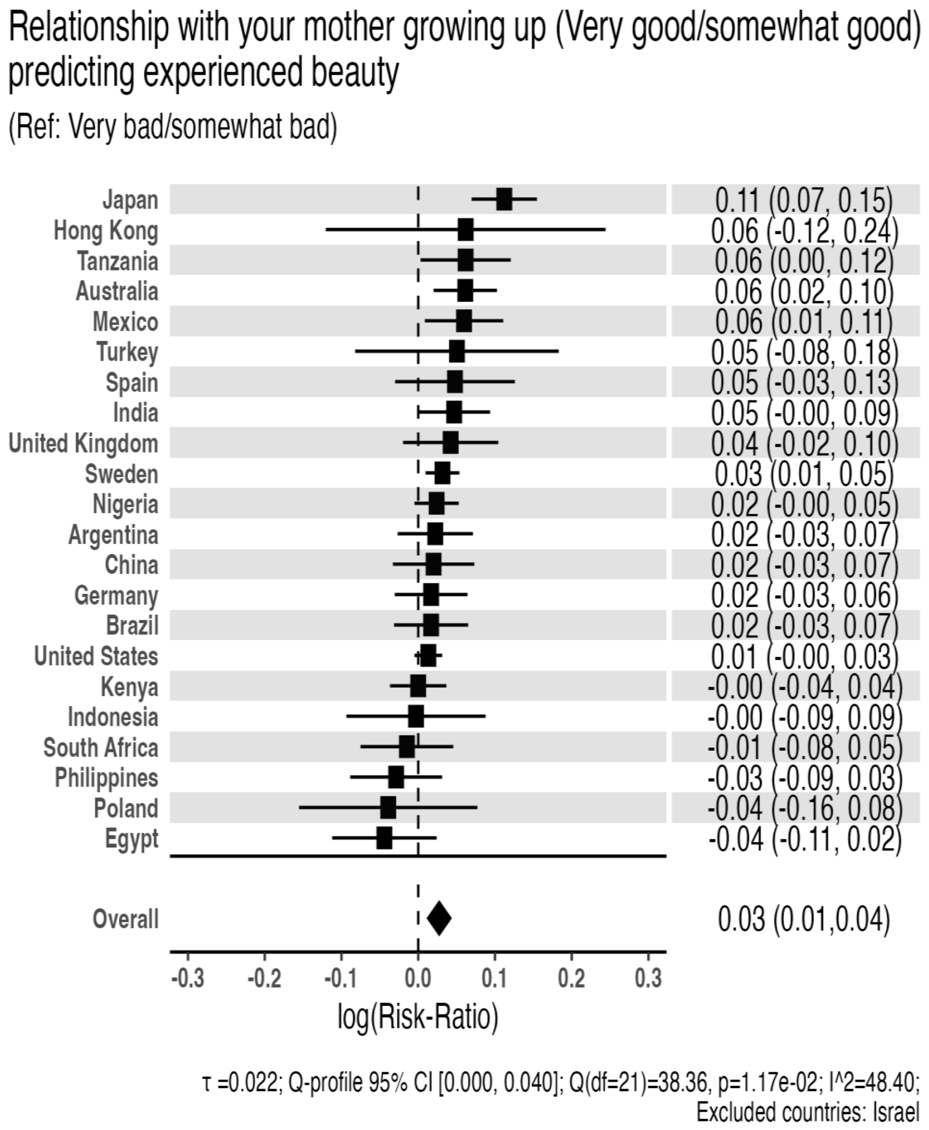

Figure S2. Forest plot of the effects of Relationship with your father growing up Very good/somewhat good (Ref: Very bad/somewhat bad) predicting experienced beauty

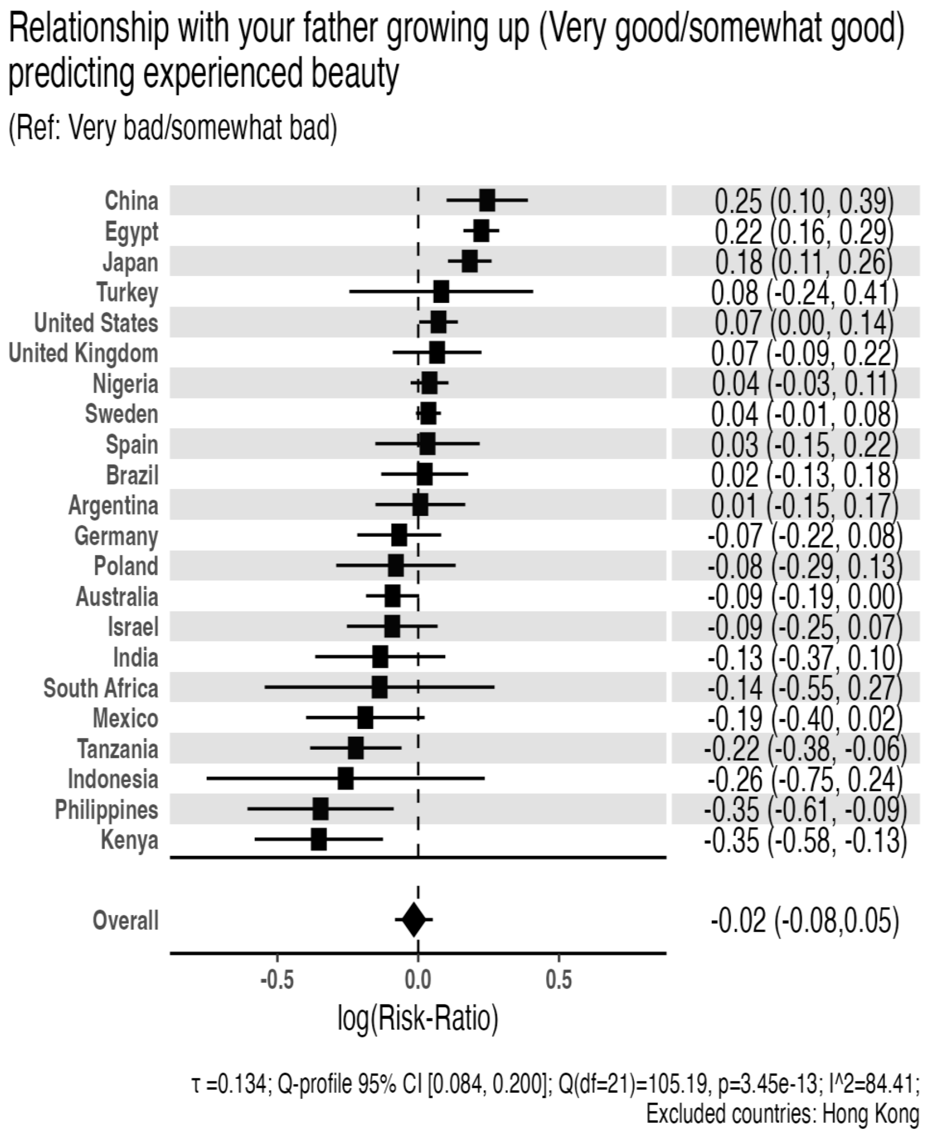

Figure S3. Forest plot of the effects of Parents married to each other when you were around 12 years old One or both of them had died (Ref: Parents married) predicting experienced beauty

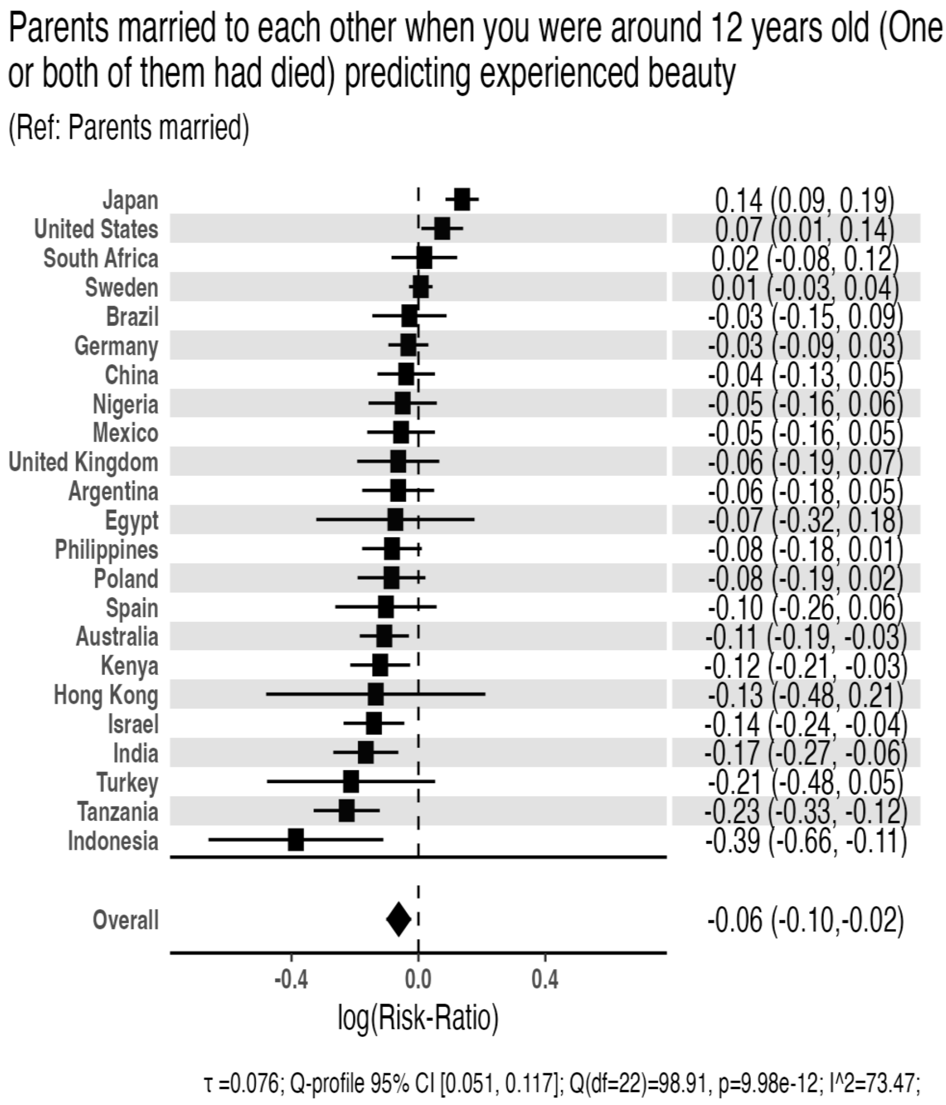

Figure S4. Forest plot of the effects of Parents married to each other when you were around 12 years old Parents were divorced (Ref: Parents married) predicting experienced beauty

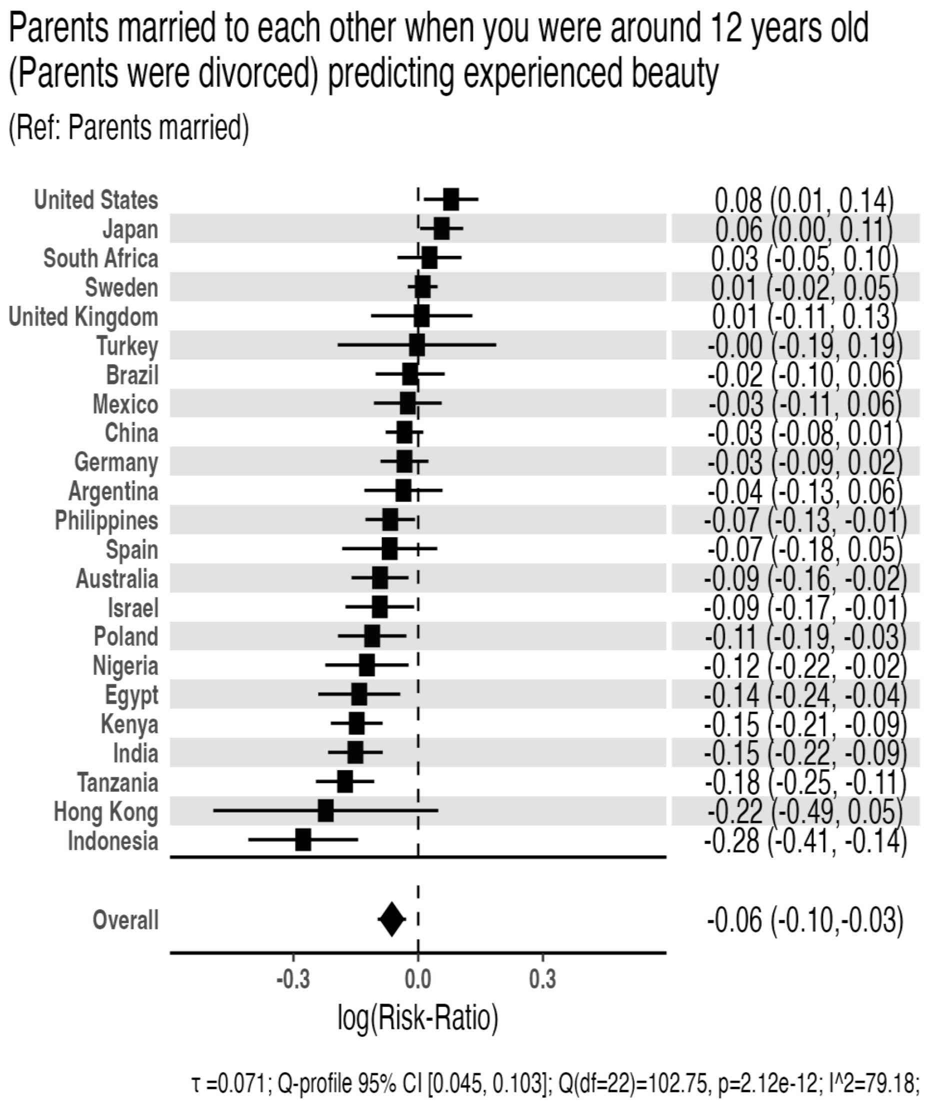

Figure S5. Forest plot of the effects of Parents married to each other when you were around 12 years old Parents were never married (Ref: Parents married) predicting experienced beauty

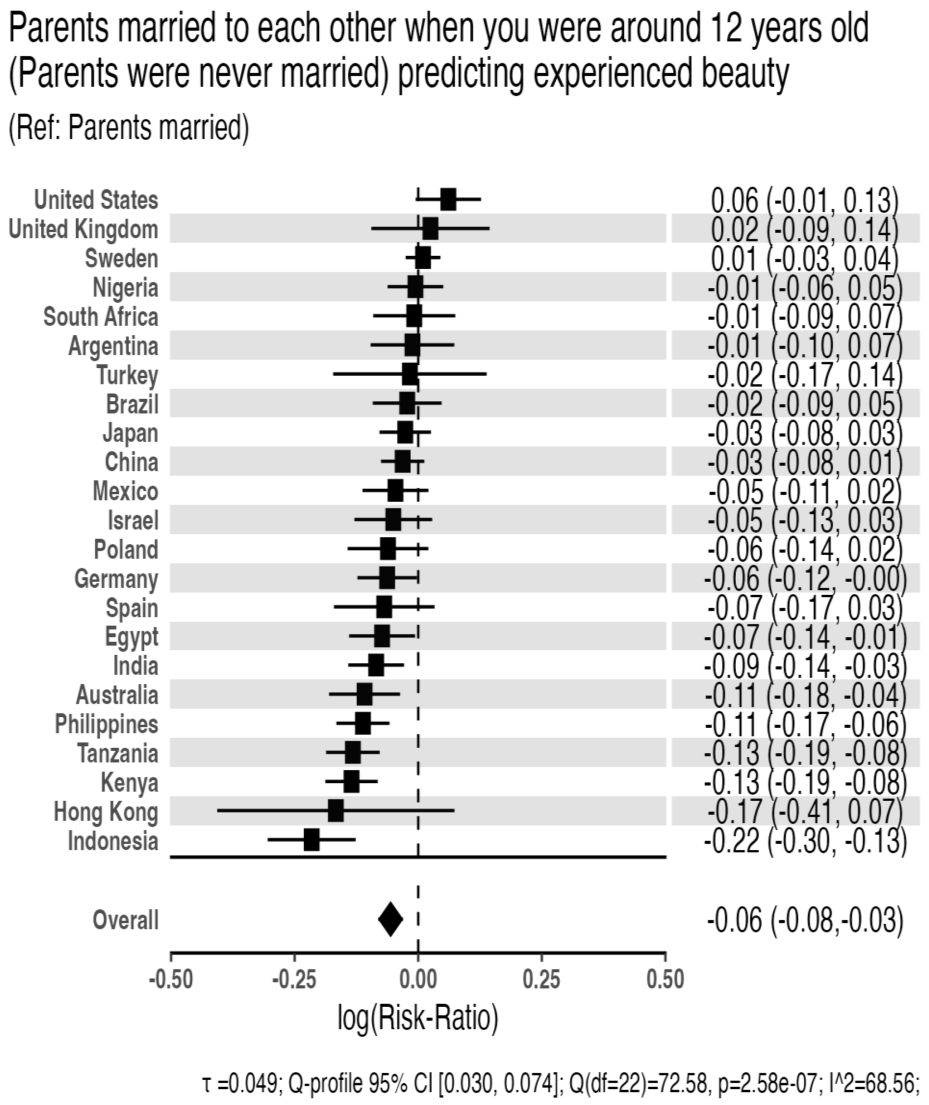

Figure S6. Forest plot of the effects of Parents married to each other when you were around 12 years old Unsure (Ref: Parents married) predicting experienced beauty

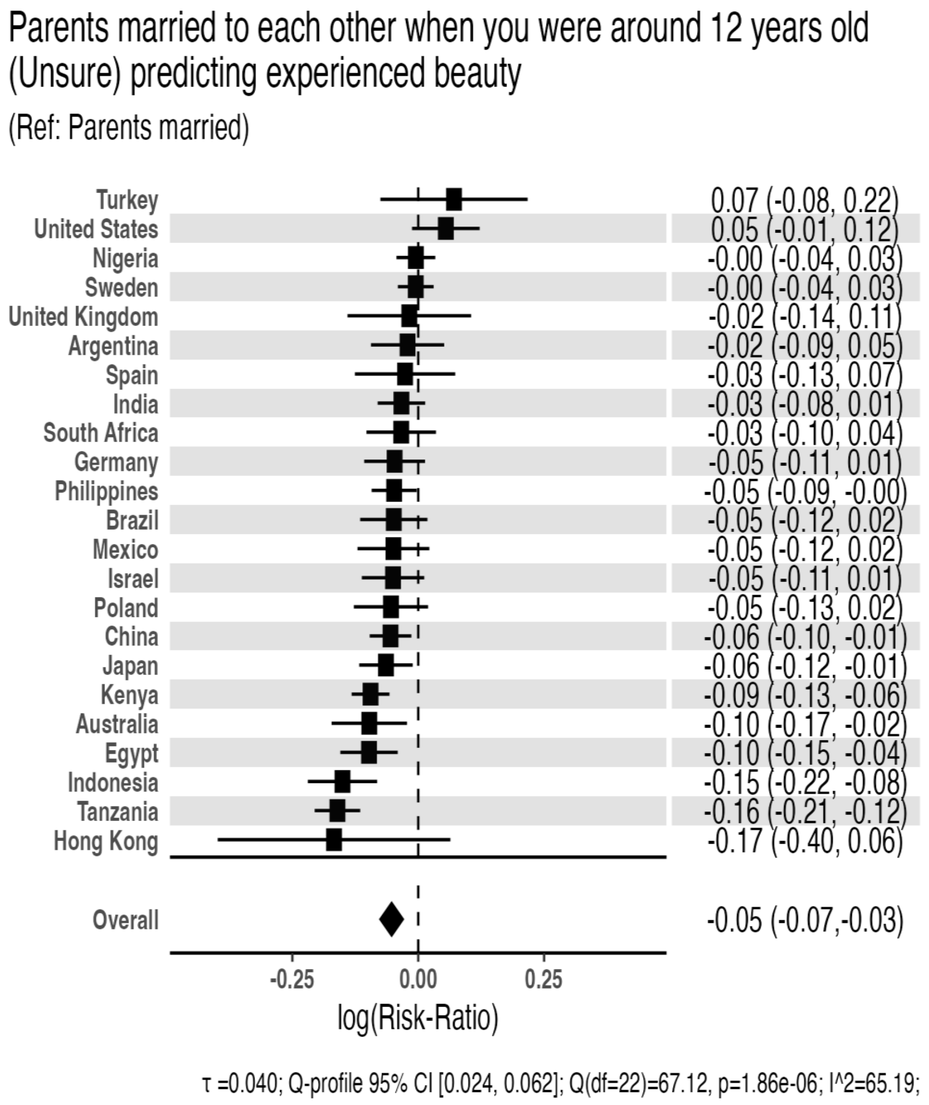

Figure S7. Forest plot of the effects of Feelings about familys household income when growing up Found it difficult (Ref: Got by) predicting experienced beauty

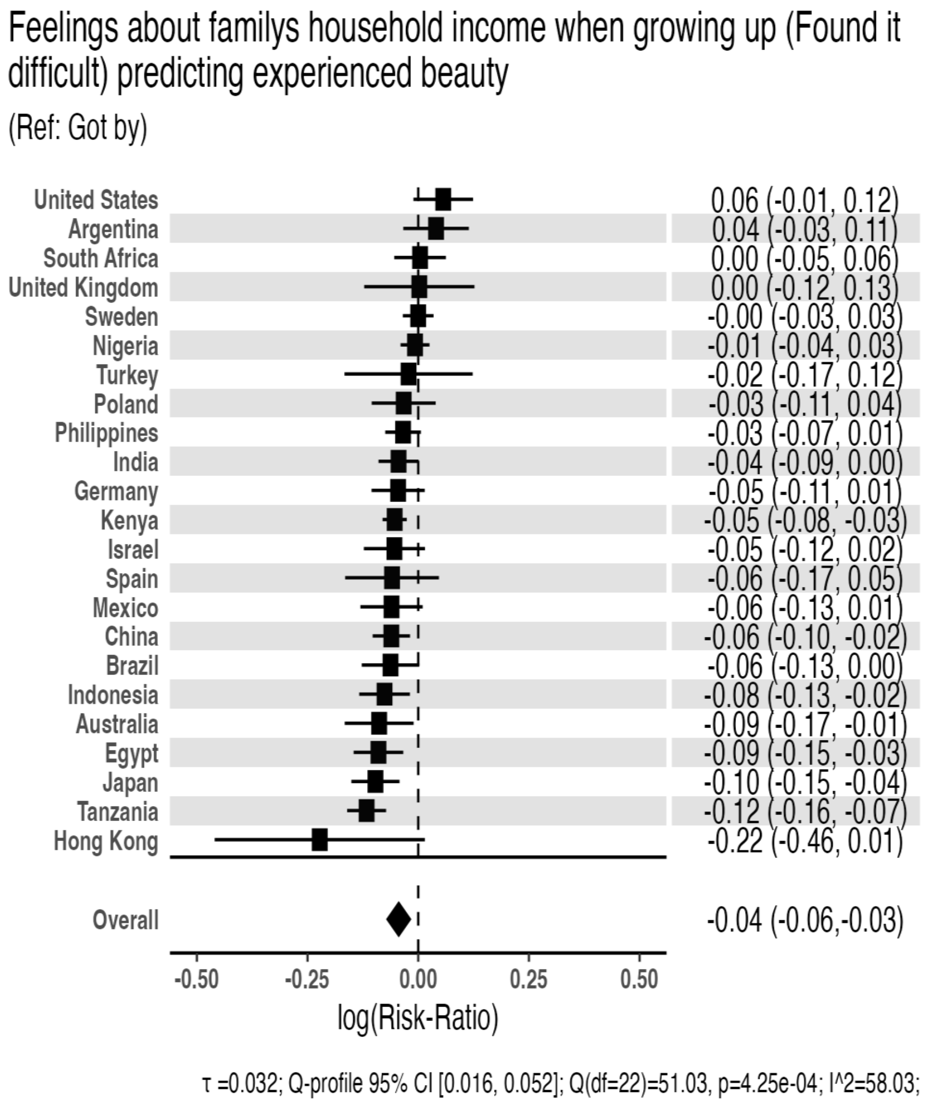

Figure S8. Forest plot of the effects of Feelings about familys household income when growing up Found it very difficult (Ref: Got by) predicting experienced beauty

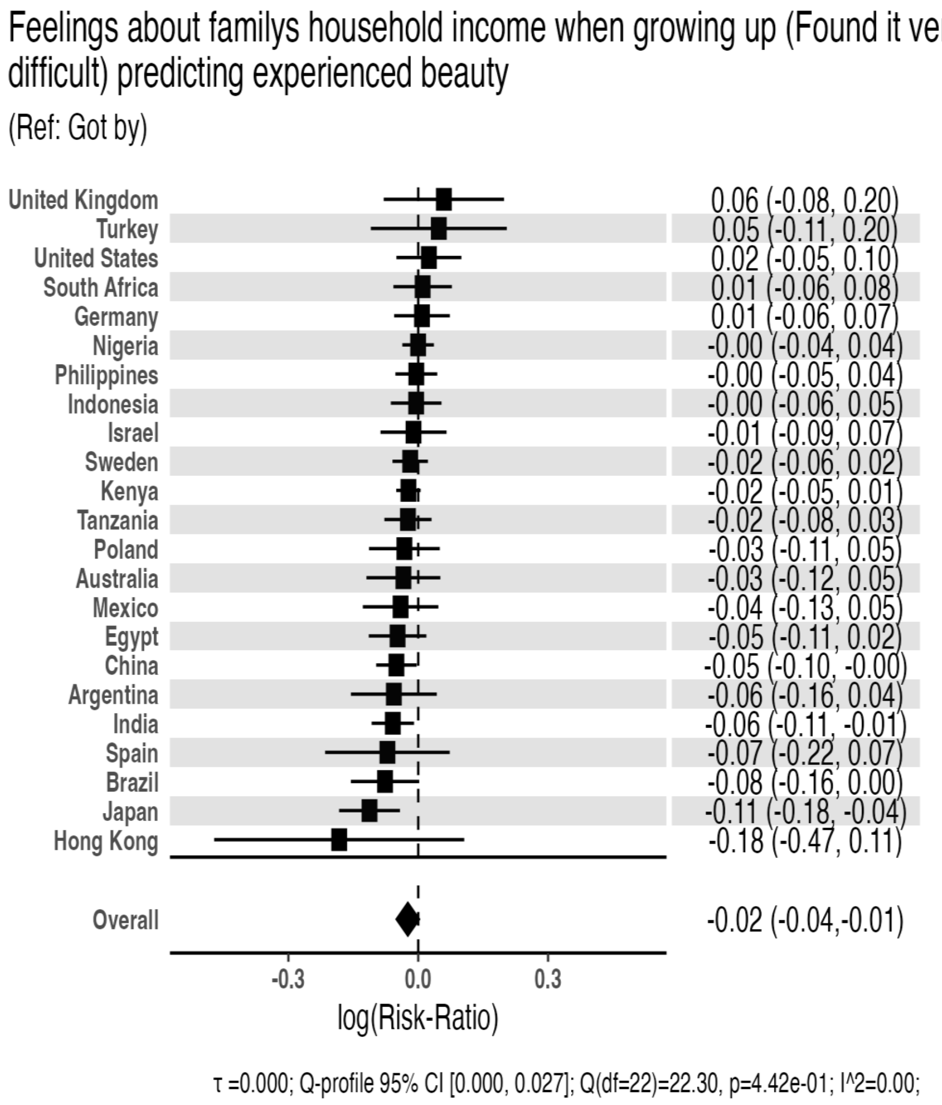

Figure S9. Forest plot of the effects of Feelings about familys household income when growing up Lived comfortably (Ref: Got by) predicting experienced beauty

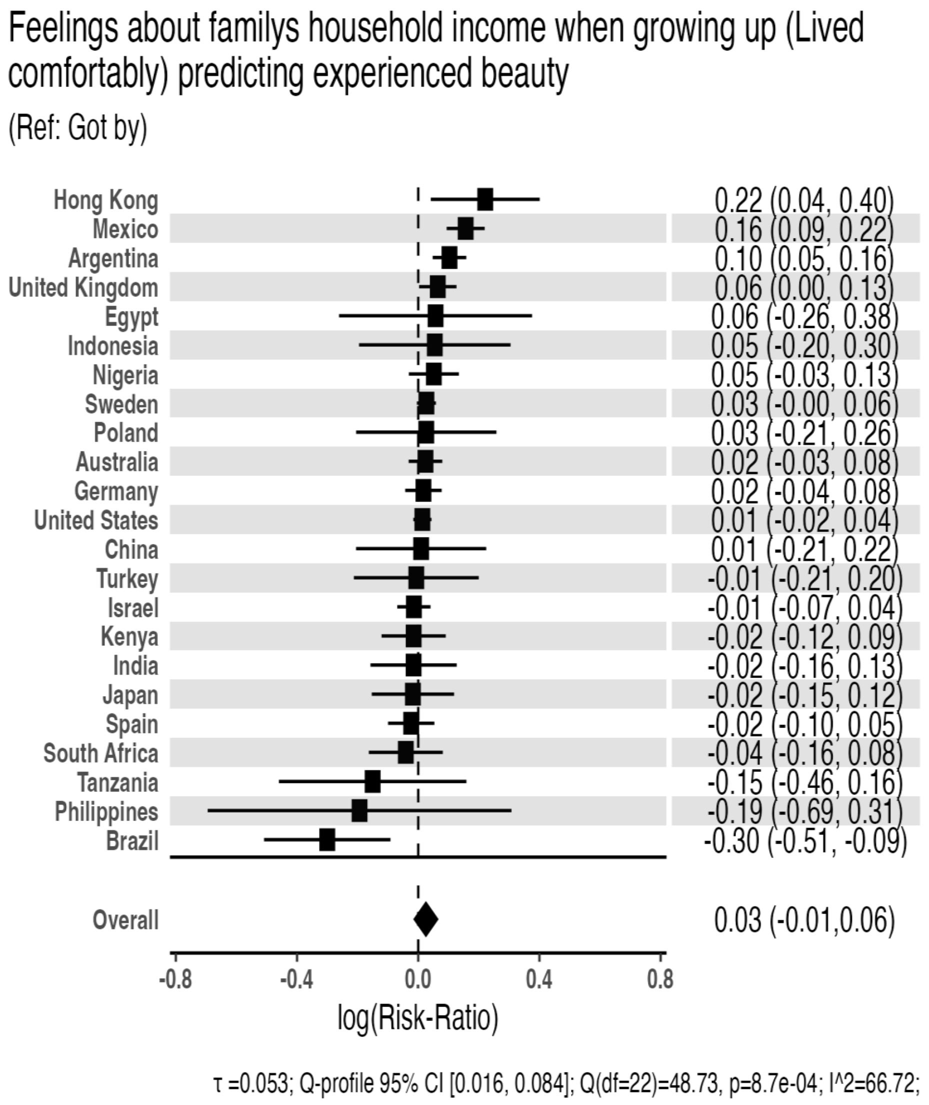

Figure S10. Forest plot of the effects of Physically or sexually abused when growing up (Ref: No) predicting experienced beauty

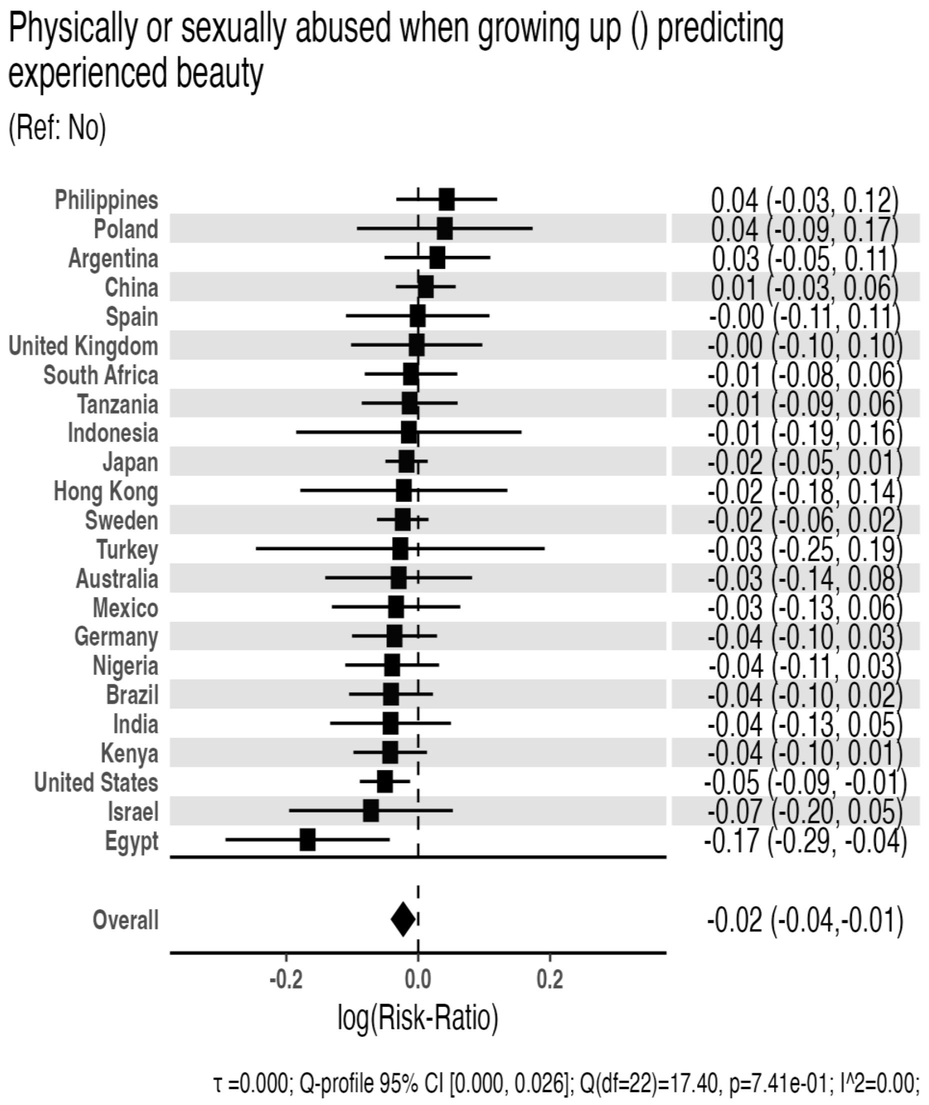

Figure S11. Forest plot of the effects of Felt like an outsider in your family when growing up Yes (Ref: No) predicting experienced beauty

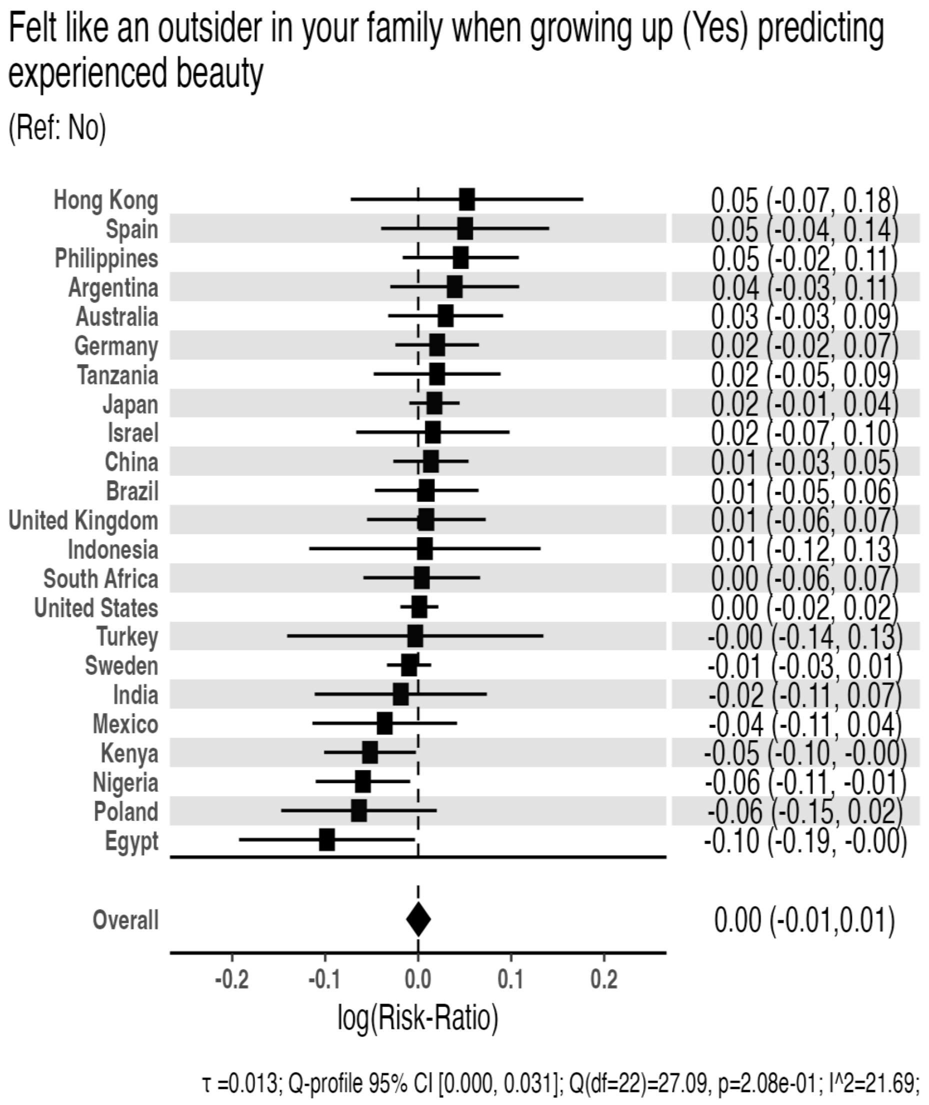

Figure S12. Forest plot of the effects of Your health when growing up Excellent (Ref: Good) predicting experienced beauty

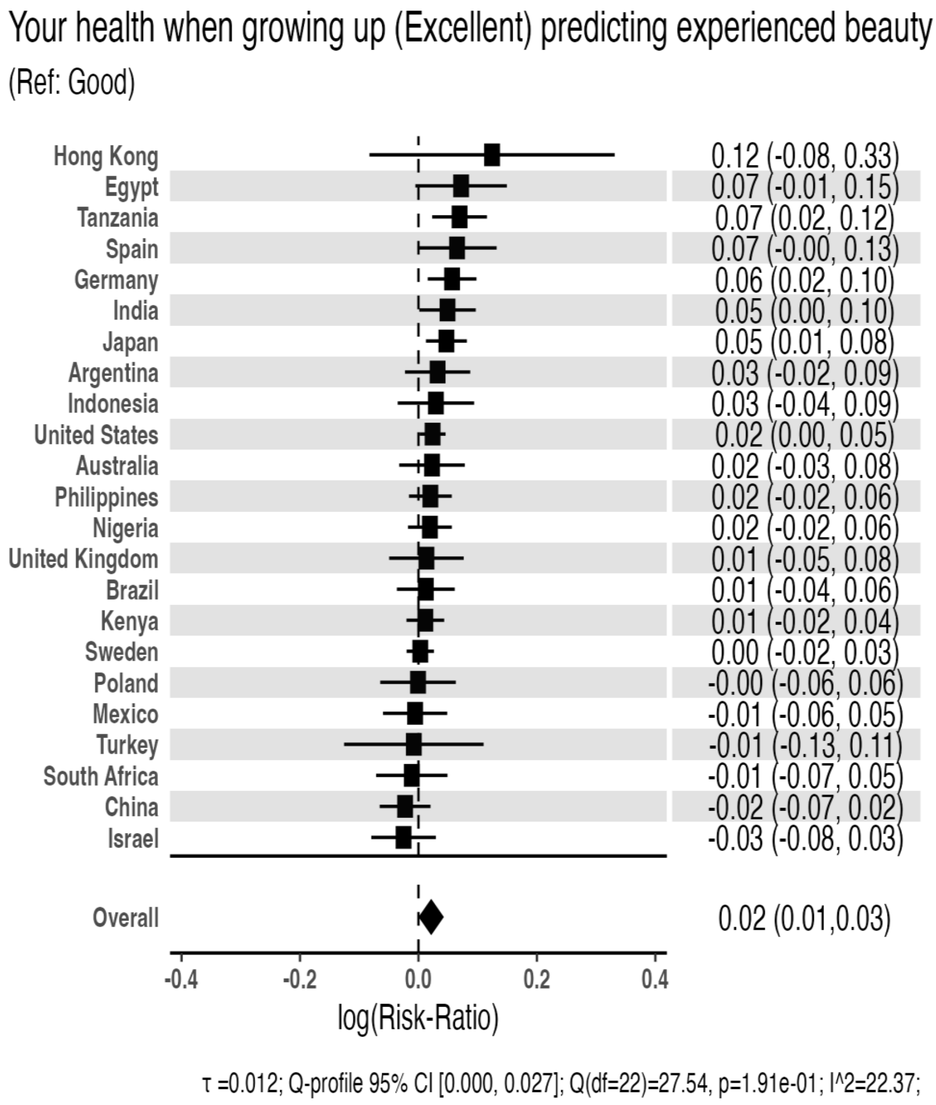

Figure S13. Forest plot of the effects of Your health when growing up Fair (Ref: Good) predicting experienced beauty

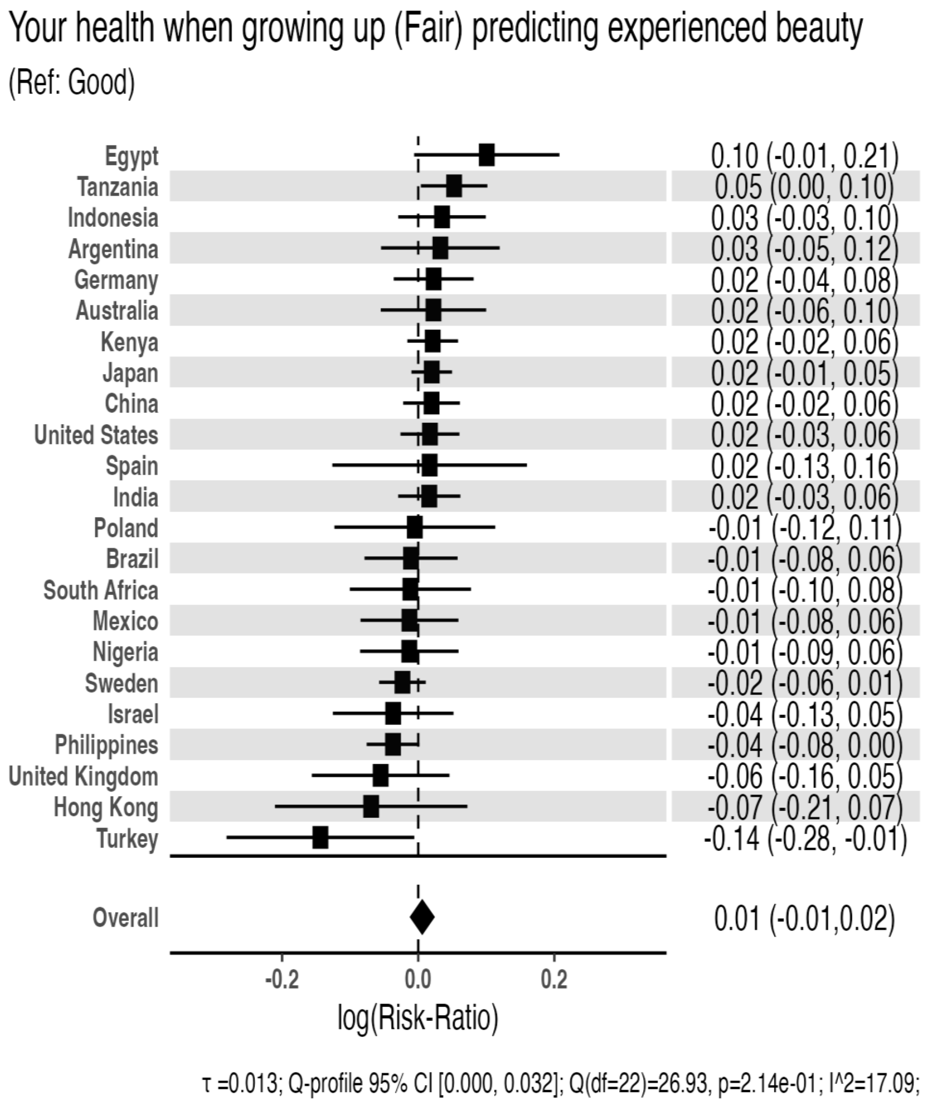

Figure S14. Forest plot of the effects of Your health when growing up Poor (Ref: Good) predicting experienced beauty

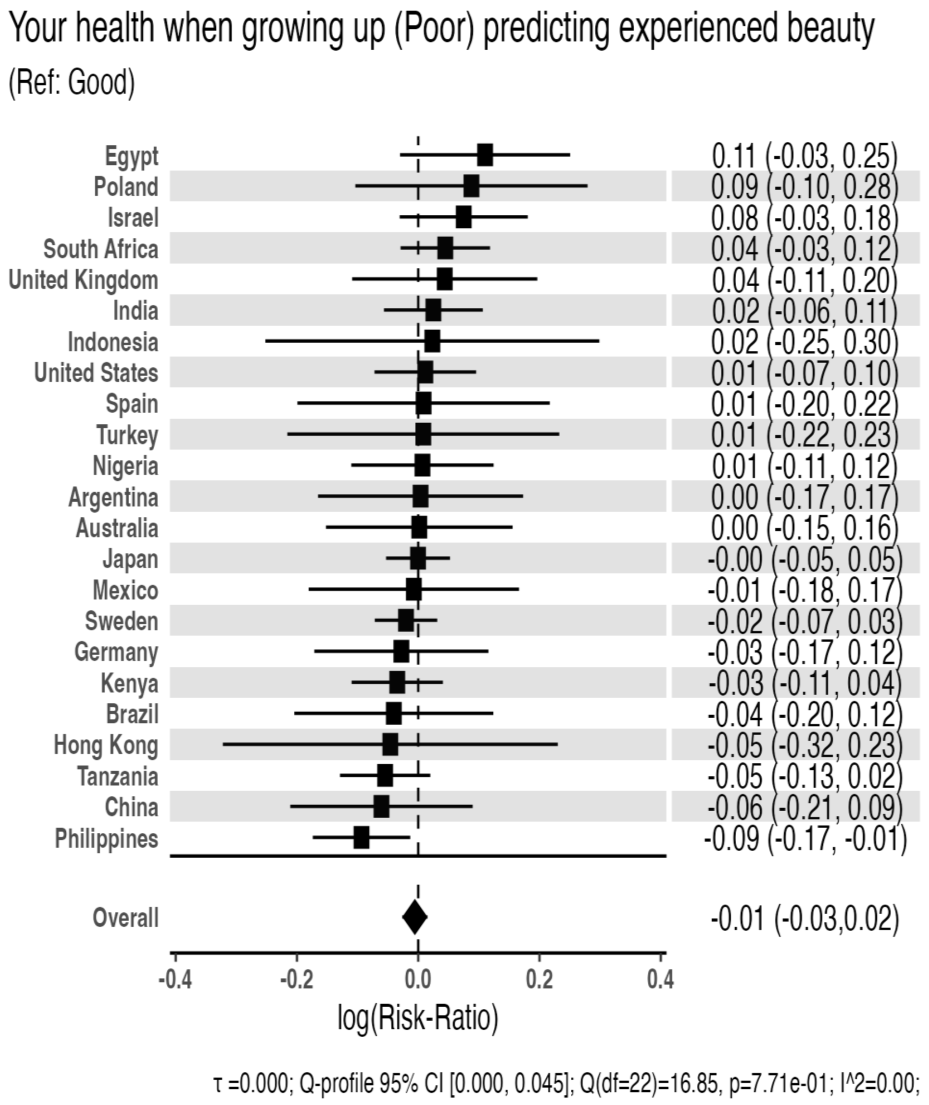

Figure S15. Forest plot of the effects of Your health when growing up Very good (Ref: Good) predicting experienced beauty

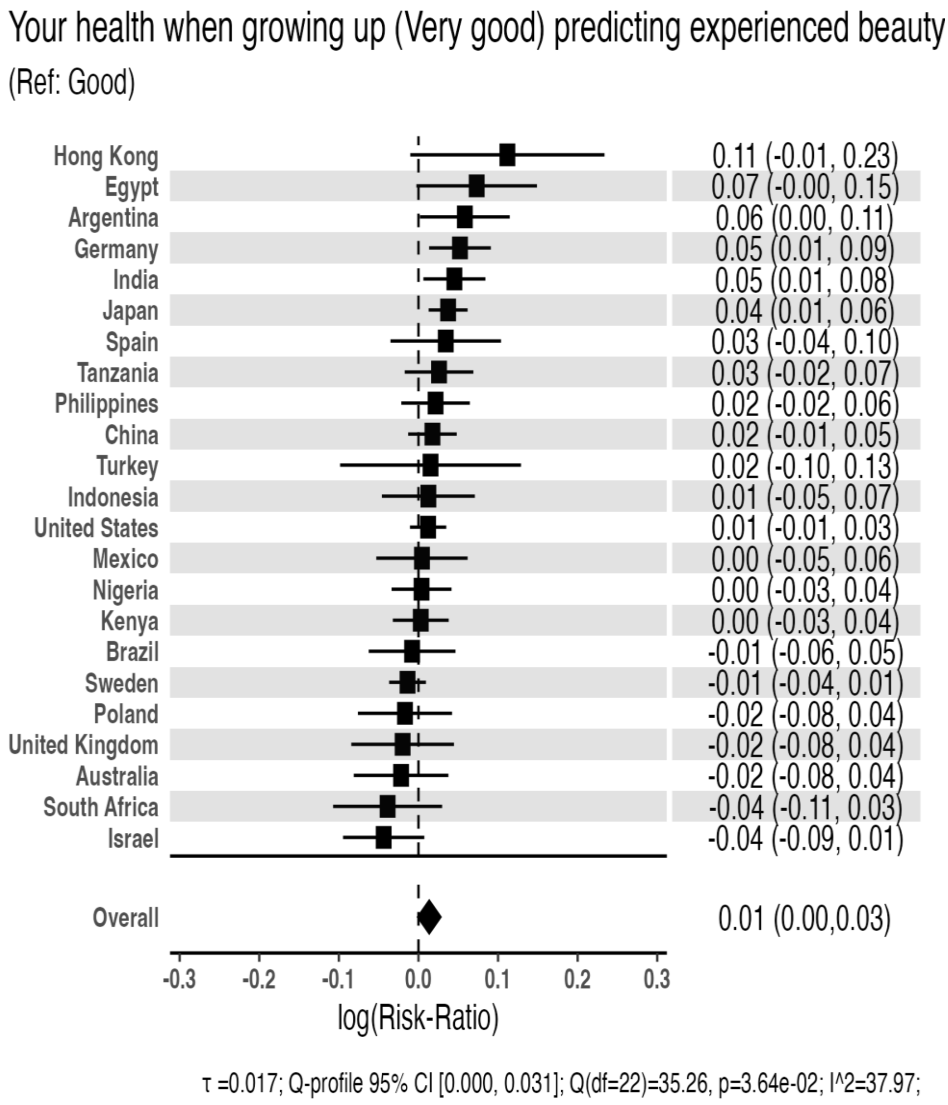

Figure S16. Forest plot of the effects of Born in This country Born in another country (Ref: Born in this country) predicting experienced beauty

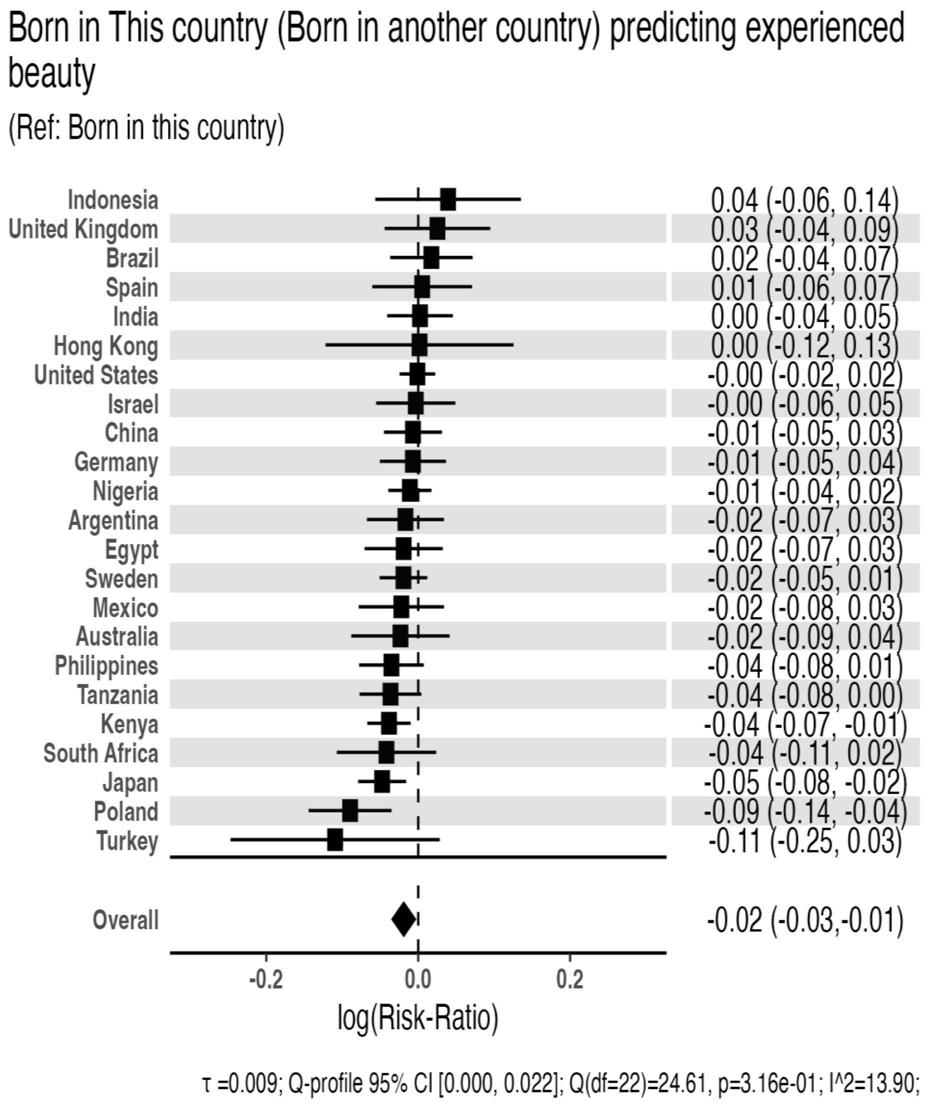

Figure S17. Forest plot of the effects of How Often You Attended Religious Services or Worshiped When You Were Around 12 Years Old At least once a week (Ref: Never) predicting experienced beauty

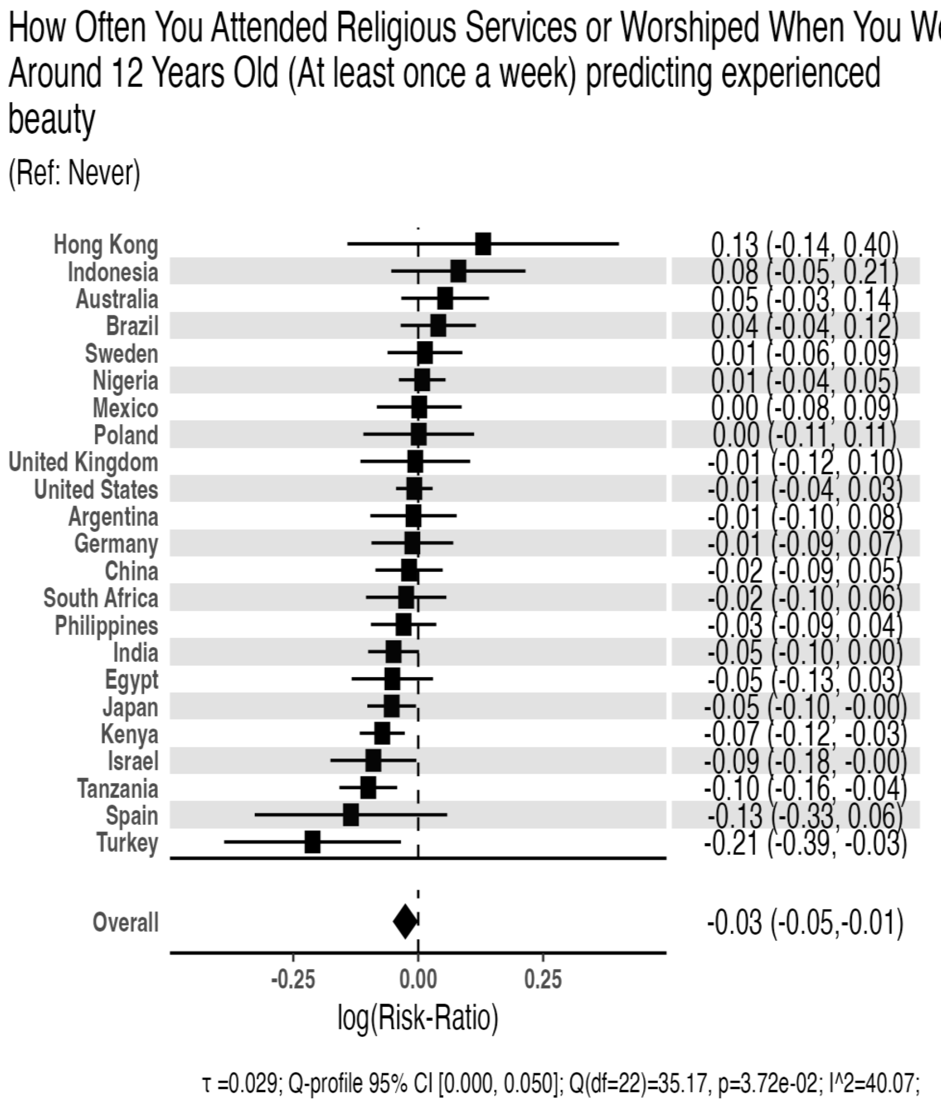

Figure S18. Forest plot of the effects of How Often You Attended Religious Services or Worshiped When You Were Around 12 Years Old Less than once a month (Ref: Never) predicting experienced beauty

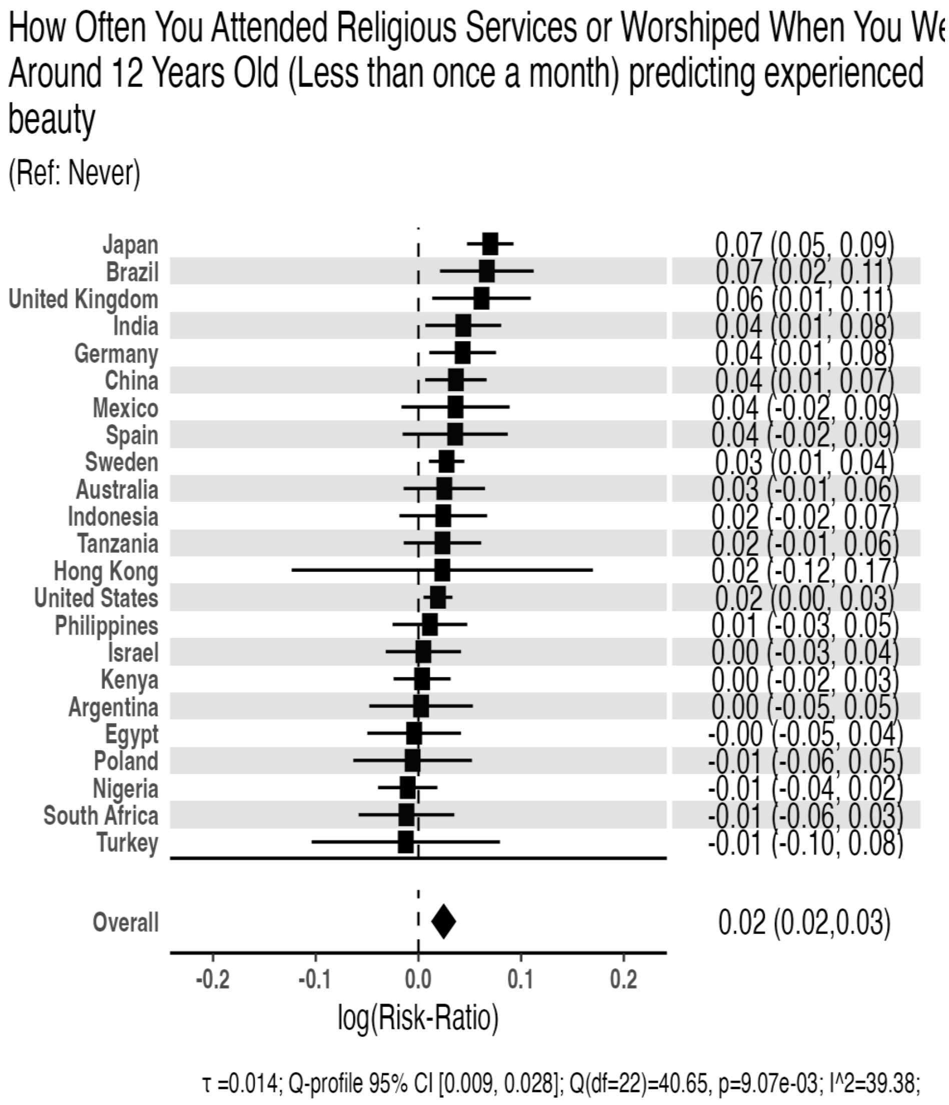

Figure S19. Forest plot of the effects of How Often You Attended Religious Services or Worshiped When You Were Around 12 Years Old One to three times a month (Ref: Never) predicting experienced beauty

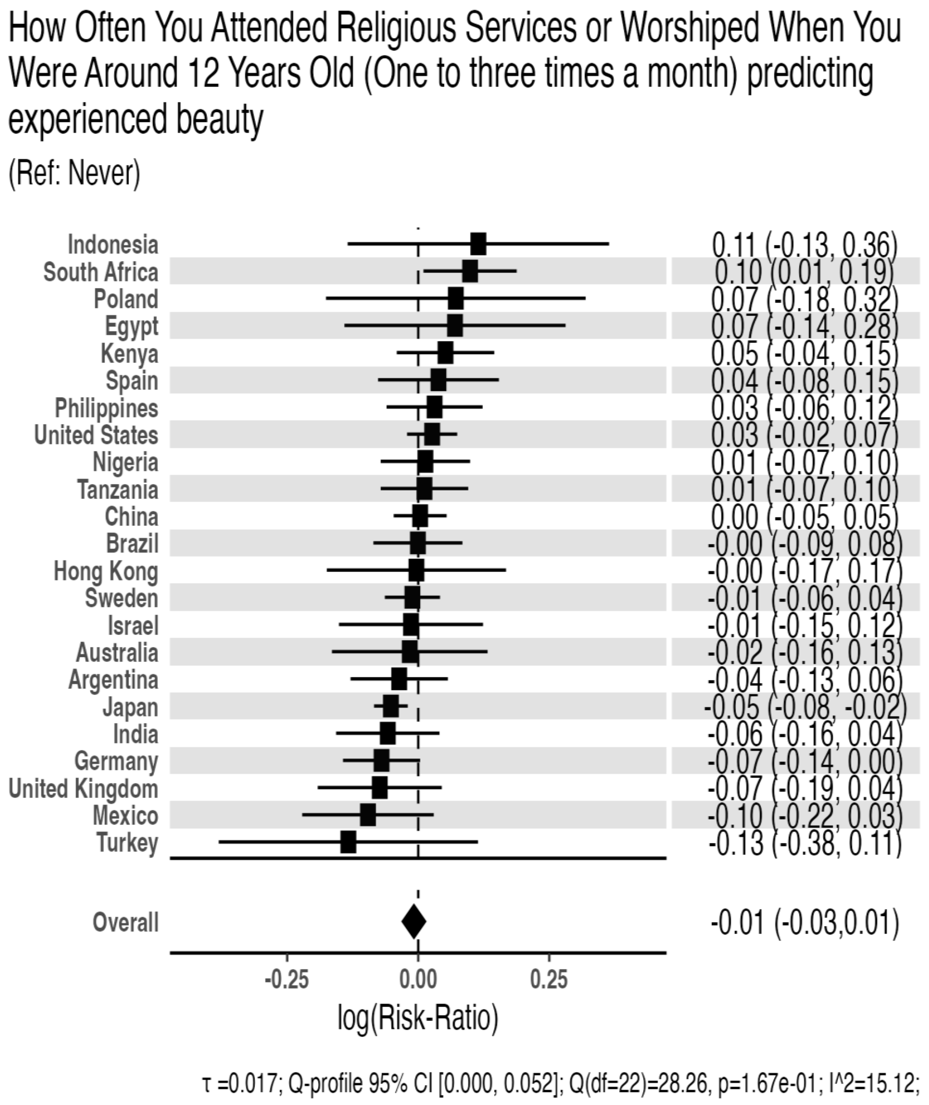

Figure S20. Forest plot of the effects of Year of birth (age group) 1943 or earlier (current age: 80+ years) (Ref: 1998-2005; current age: 18-24) predicting experienced beauty

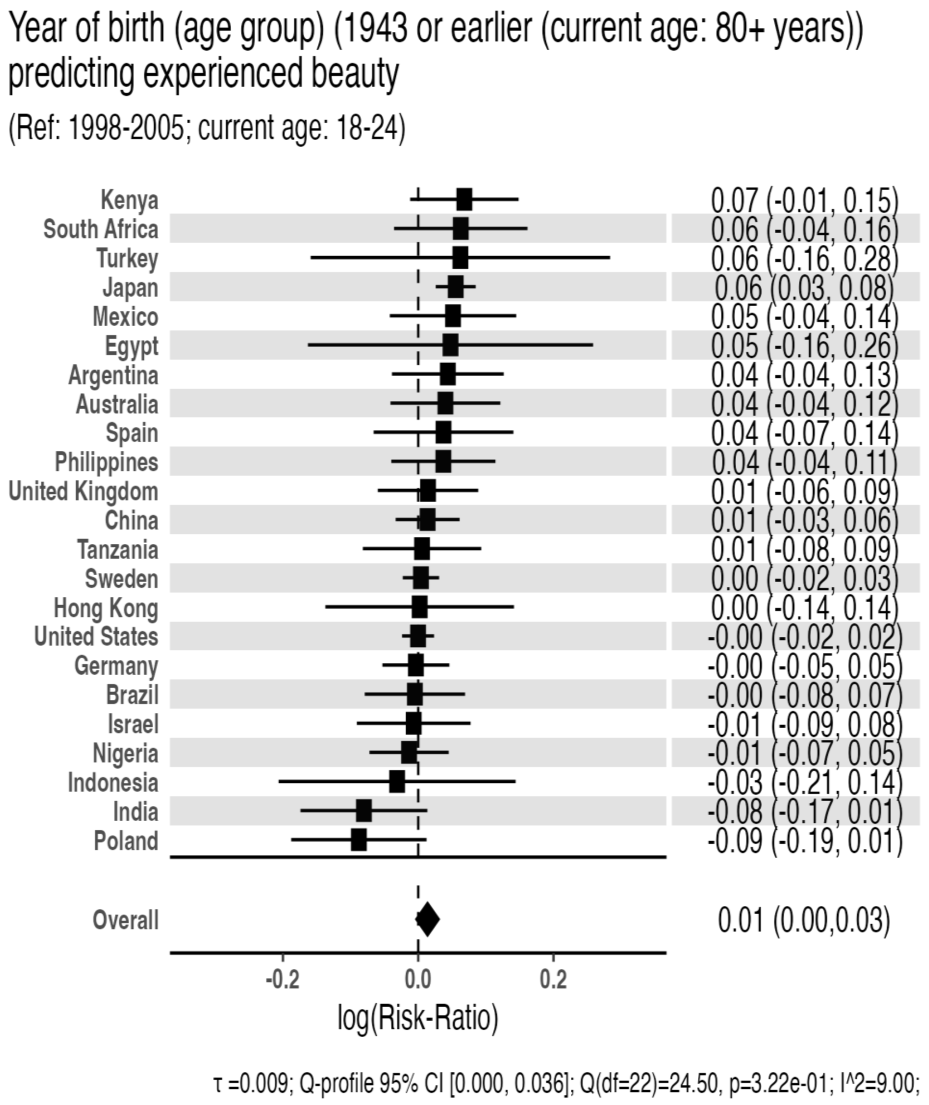

Figure S21. Forest plot of the effects of Year of birth (age group) 1943-1953 (current age: 70-79 years) (Ref: 1998-2005; current age: 18-24) predicting experienced beauty

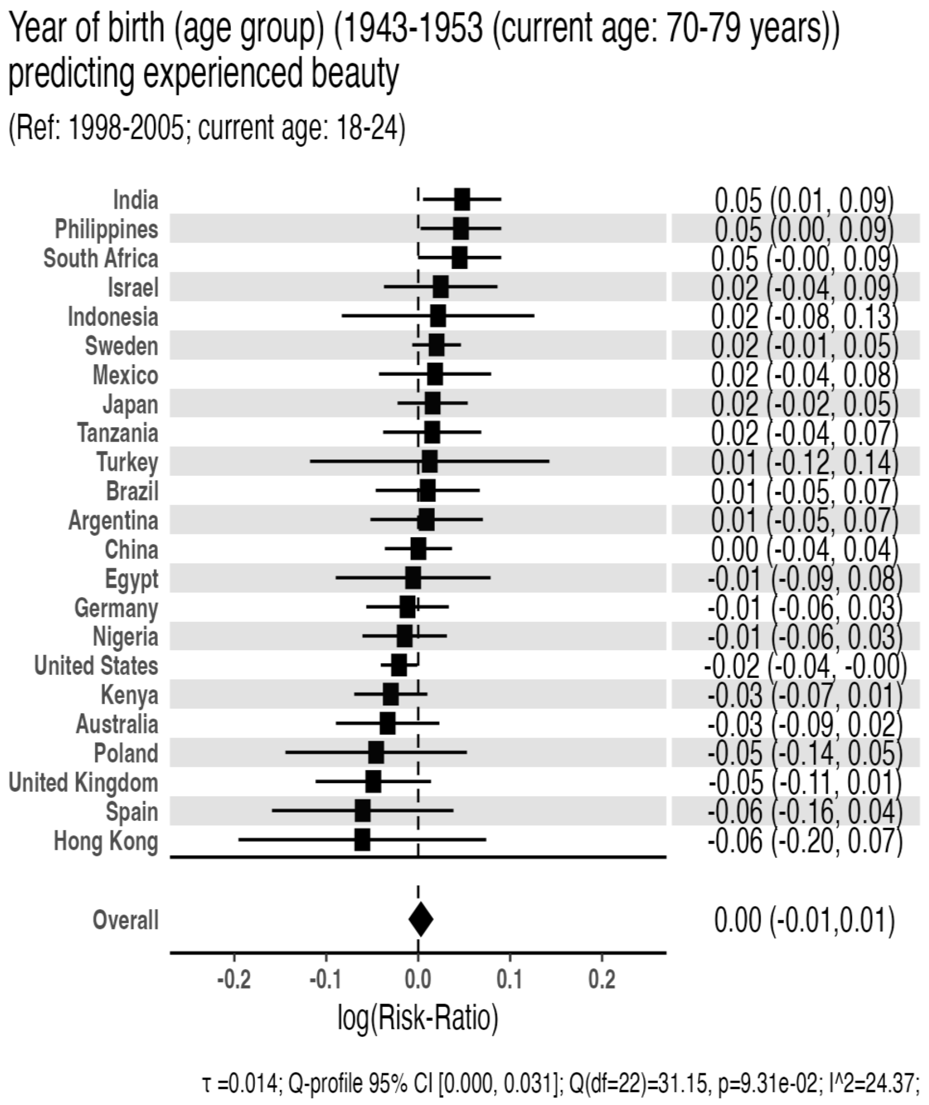

Figure S22. Forest plot of the effects of Year of birth (age group) 1953-1963 (current age: 60-69 years) (Ref: 1998-2005; current age: 18-24) predicting experienced beauty

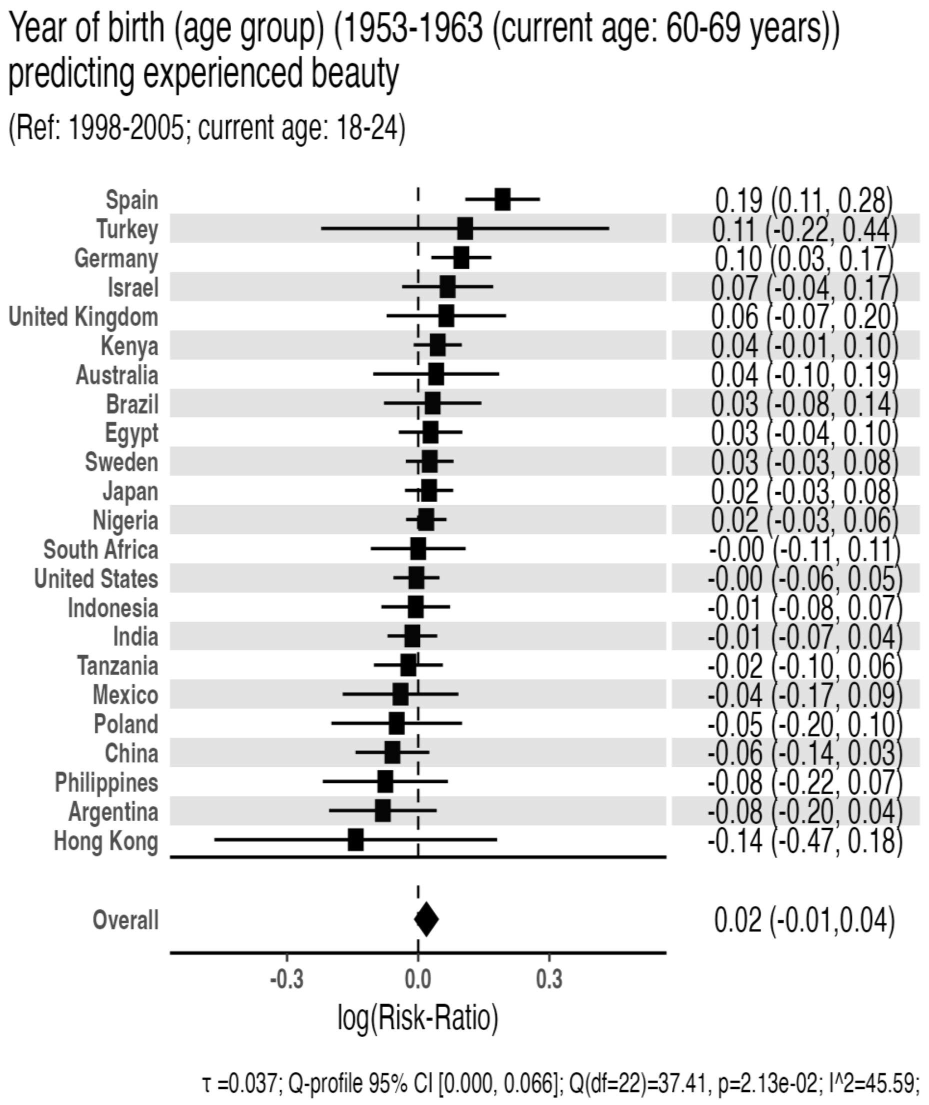

Figure S23. Forest plot of the effects of Year of birth (age group) 1963-1973 (current age: 50-59 years) (Ref: 1998-2005; current age: 18-24) predicting experienced beauty

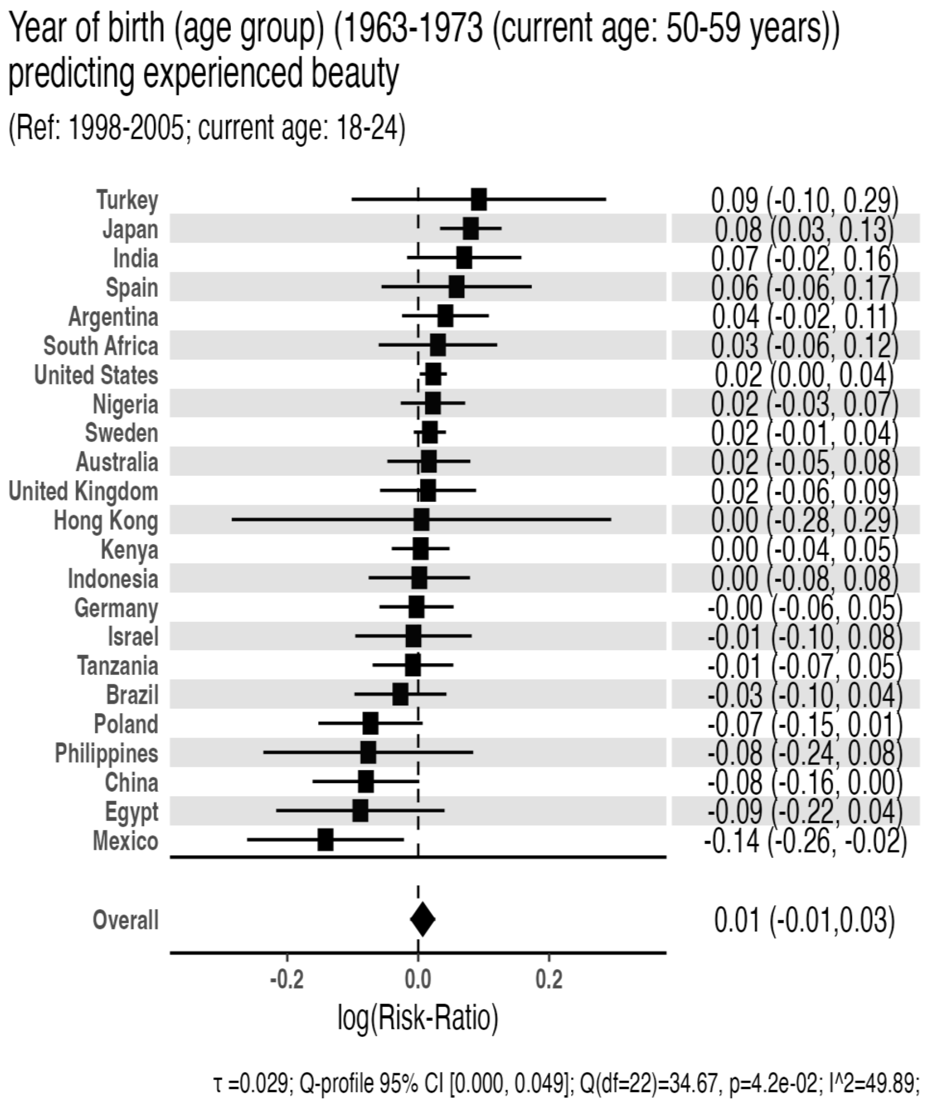

Figure S24. Forest plot of the effects of Year of birth (age group) 1973-1983 (current age: 40-49 years) (Ref: 1998-2005; current age: 18-24) predicting experienced beauty

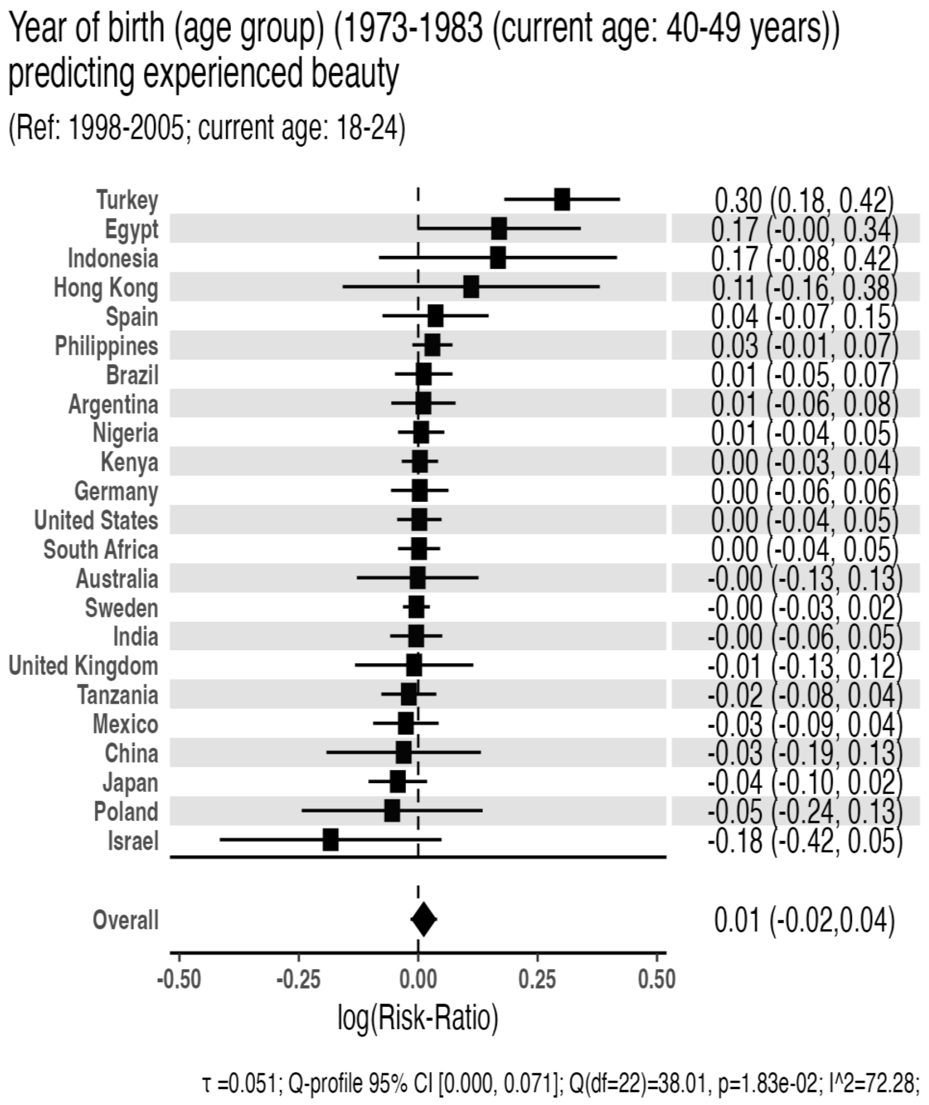

Figure S25. Forest plot of the effects of Year of birth (age group) 1983-1993 (current age: 30-39 years) (Ref: 1998-2005; current age: 18-24) predicting experienced beauty

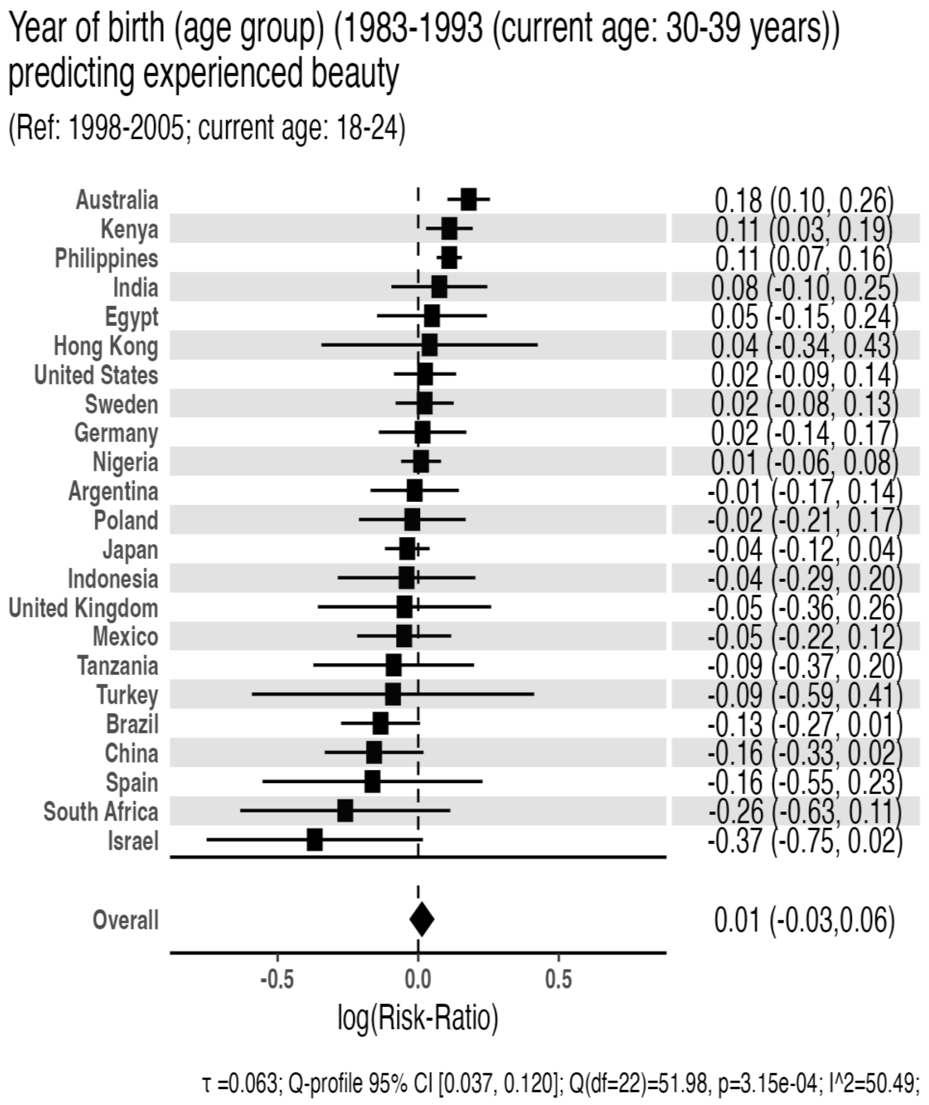

Figure S26. Forest plot of the effects of Year of birth (age group) 1993-1998 (current age: 25-29 years) (Ref: 1998-2005; current age: 18-24) predicting experienced beauty

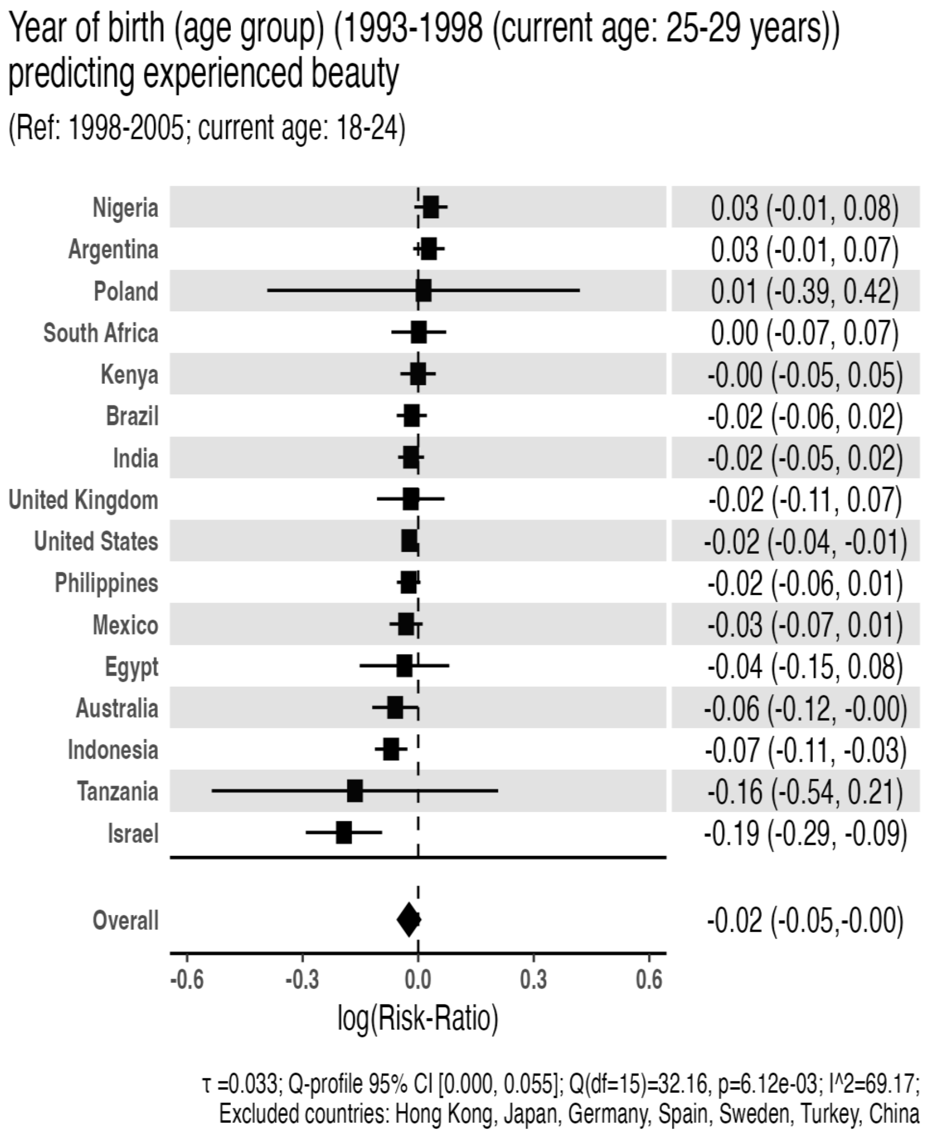

Figure S27. Forest plot of the effects of Religion when twelve years old Christianity (Ref: No religion/Atheist/Agnostic) predicting experienced beauty

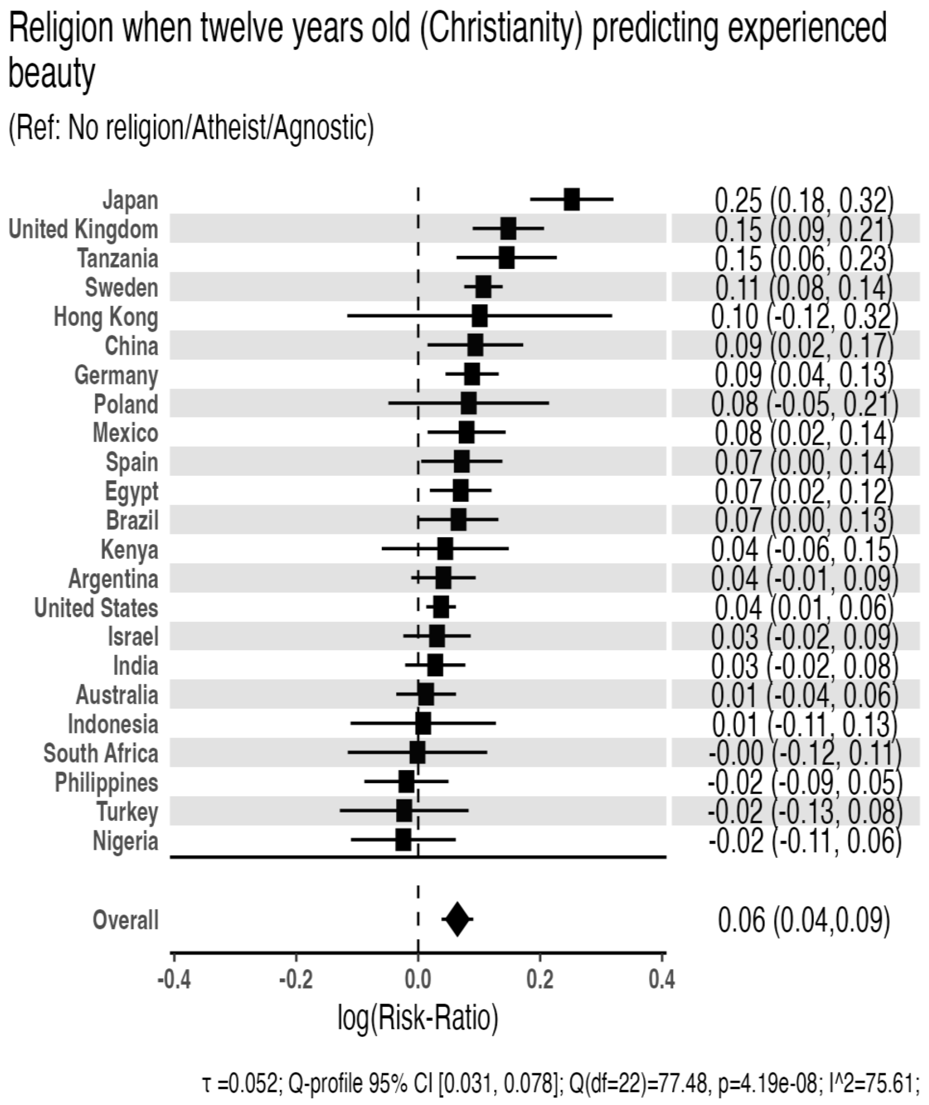

Figure S28. Forest plot of the effects of Religion when twelve years old Collapsed affiliations with prevalence<3% (Ref: No religion/Atheist/Agnostic) predicting experienced beauty

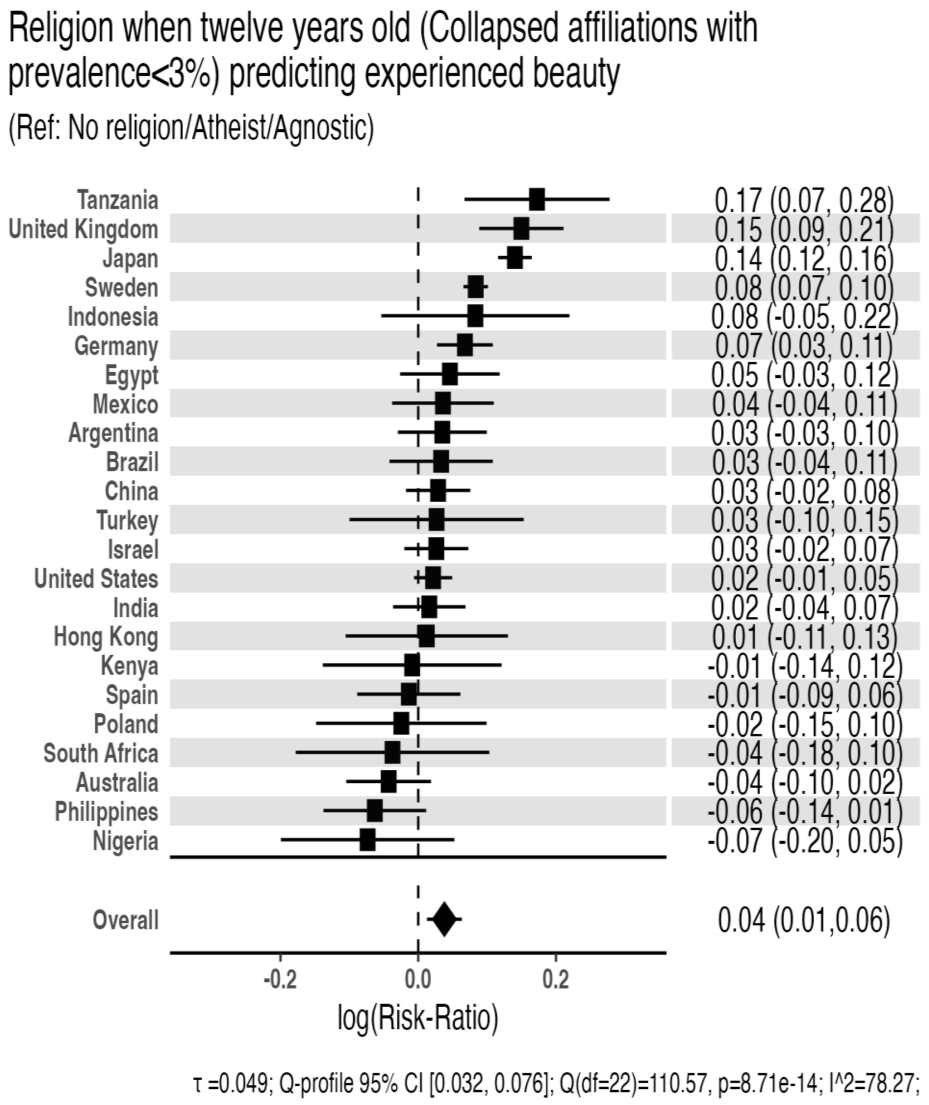

Figure S29. Forest plot of the effects of Race plurality (prominent race/ethnic group [0] or not [1]) Non-plurality groups (Ref: Plurality group) predicting experienced beauty

Race plurality (prominent race/ethnic group [0] or not [1])
(Non-plurality groups) predicting experienced beauty

(Ref: Plurality group)

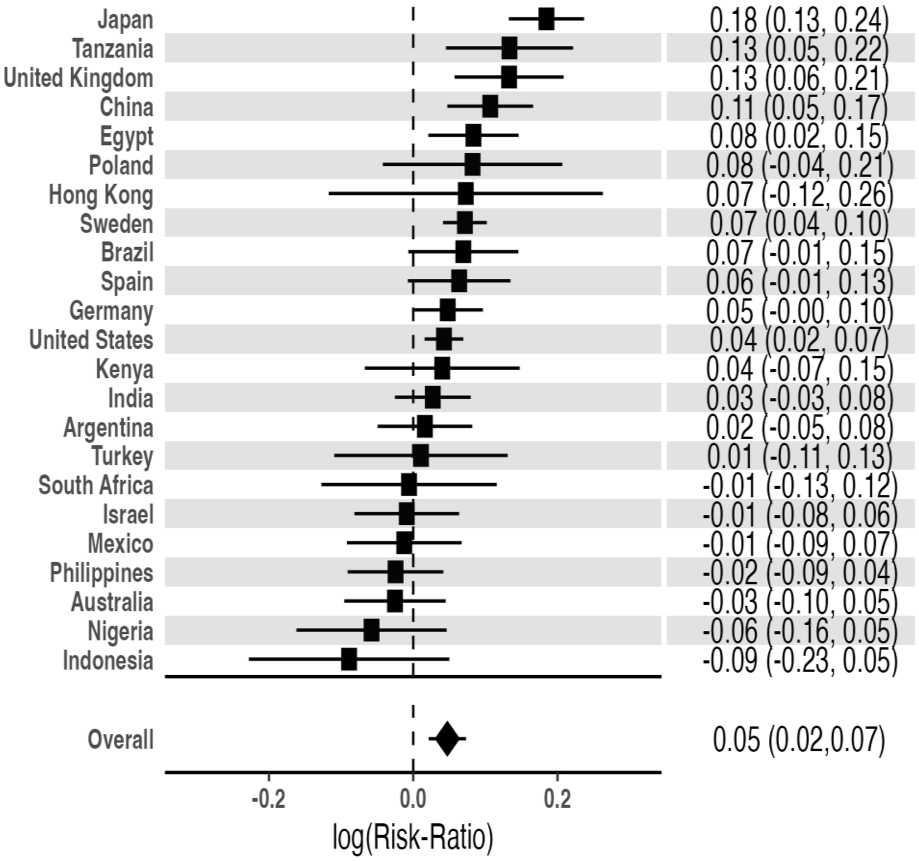

τ =0.049; Q-profile 95% CI [0.029, 0.076]; Q(df=22)=65.87, p=2.91e-06; I^2=70.58;
